# Prediction of Type III Secretion Signals in Genomes of Gram-Negative Bacteria

**DOI:** 10.1371/journal.pone.0005917

**Published:** 2009-06-15

**Authors:** Martin Löwer, Gisbert Schneider

**Affiliations:** Johann Wolfgang Goethe-University, Chair for Chem- and Bioinformatics, Frankfurt, Germany; The Research Institute for Children at Children's Hospital New Orleans, United States of America

## Abstract

**Background:**

Pathogenic bacteria infecting both animals as well as plants use various mechanisms to transport virulence factors across their cell membranes and channel these proteins into the infected host cell. The type III secretion system represents such a mechanism. Proteins transported *via* this pathway (“effector proteins”) have to be distinguished from all other proteins that are not exported from the bacterial cell. Although a special targeting signal at the N-terminal end of effector proteins has been proposed in literature its exact characteristics remain unknown.

**Methodology/Principal Findings:**

In this study, we demonstrate that the signals encoded in the sequences of type III secretion system effectors can be consistently recognized and predicted by machine learning techniques. Known protein effectors were compiled from the literature and sequence databases, and served as training data for artificial neural networks and support vector machine classifiers. Common sequence features were most pronounced in the first 30 amino acids of the effector sequences. Classification accuracy yielded a cross-validated Matthews correlation of 0.63 and allowed for genome-wide prediction of potential type III secretion system effectors in 705 proteobacterial genomes (12% predicted candidates protein), their chromosomes (11%) and plasmids (13%), as well as 213 *Firmicute* genomes (7%).

**Conclusions/Significance:**

We present a signal prediction method together with comprehensive survey of potential type III secretion system effectors extracted from 918 published bacterial genomes. Our study demonstrates that the analyzed signal features are common across a wide range of species, and provides a substantial basis for the identification of exported pathogenic proteins as targets for future therapeutic intervention. The prediction software is publicly accessible from our web server (www.modlab.org).

## Introduction

**Figure 1:**
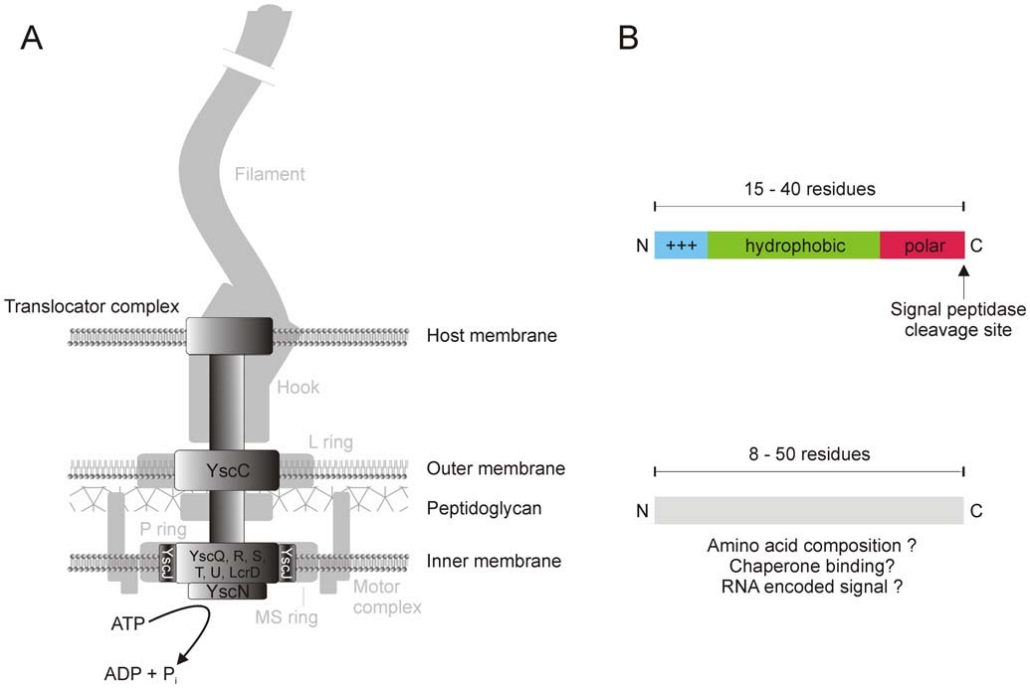
The bacterial type III secretion system (T3SS) forms a translocator complex spanning the bacterial and the host cell membranes for protein translocation. (a) Schematic T3SS structure together with a flagella apparatus (shaded in light grey). The nine components being conserved among T3SS are named in *Yersinia* nomenclature. In flagella apparati, proteins of the axial structure are exported *via* a T3SS, e.g. flagellins. Note that T3SS injection needle and translocator complex are not present in flagella (adapted from Sheng *et al.* [5] and Pallen and Matzke [4]). (b) Comparison of the features of classic signal peptides (top) [12] and the proposed features of T3SS signals (bottom). Both kinds of signals are located at the N-terminal end of a protein.

There are six known types of secretion systems in Gram-negative bacteria [1]. Among these, several prediction systems are available for the *sec* pathway that can be used to recognize N-terminal secretion signals (signal peptides) [2]. Predicting proteins that are secreted *via* other pathways has recently become a major goal of bioinformatics research [3]. The multi sub-unit type III secretion systems (T3SS) contribute to flagellar biosynthesis [4] and interaction with eukaryotic cells (Figure 1a) [5] and are therefore often involved in pathogenicity of the corresponding bacterial species, *e.g. Yersinia pestis*, *Salmonella enterica*, and *Escherichia coli* [6], [7].

Substrate specificity of the T3SS relies on two distinct signals. Most T3SS effector proteins contain an N-terminal secretion signal, which is believed to be generic for the T3SS from different species [6]. Cellular decoding of this signal is achieved by a family of cytosolic chaperones which bind the effector sequences and are recognized by the secretion machinery [6]. Usually, there is one chaperone *per* effector protein, but chaperones targeting several effectors have also been described [6]. The genes encoding the corresponding effector proteins and their chaperones are often organized in direct vicinity on the coding DNA sequence [8]. The function of these chaperones is not entirely clear; however, experimental results: support a role as antifolding factors since fully folded effector proteins are too big for the translocation channel, and stabilizers of effector proteins, which are rapidly degraded in the absence of the corresponding chaperone [5]. Also, they are thought to provide a secondary secretion signal which is somehow involved in the prioritization and order of effector secretion [5].

Analyses of known effector sequences have revealed characteristic properties, such as an overall amphipathic amino acid composition, an over-representation of serine and glutamine, and the absence of acidic residues [9]. The actual secretion signal is believed to be contained in the first 50 amino acids, although synthetic signals with as few as eight residues have been shown to promote type III secretion in *Yersinia* [10]. Furthermore, some evidence has been collected that the signal might be encoded on RNA level rather than on protein level [11]. Figure 1b presents the typical structure of a classic signal peptide [12] compared to T3SS signals.

Recent sequence-based bioinformatics approaches to finding new effector proteins utilize consensus sequence patterns of the N-terminal secretion signals [9], similarity-based comparison to known effectors [13], the genomic organization of the effectors by identifying genes in vicinity to chaperone homologues [14], and amino acid composition rules [15]. Here we present a new machine learning approach to identify potential T3SS effectors by their N-terminal amino acid sequence using a sliding window procedure in combination with artificial neural networks (ANN, feedforward type) [16] and support vector machine (SVM) classifiers [17], together with a comprehensive prediction of potential T3SS effectors for 918 bacterial genomes.

## Materials and Methods

### Data preparation

We collected a raw data set containing a total of 1,860 protein sequences (979 positive, 881 negative samples) from various literature and database sources. Included were sequences from the SwissProt [18] and *Pseudomonas syringae* Hop [19] databases and from a dataset published by Tobe and coworkers [13]. The negative data consisted of 881 cytoplasmatic sequences and secreted proteins from Gram-negative organisms. The publicly available SignalP [20] and SeretomeP [21] training sets were included. Each of the sequences of the secreted proteins contains an N-terminal secretion signal for the *sec* pathway. Possible redundancy of both datasets was reduced by using the PISCES implementation of the Hobohm algorithm [22]. Sequences with fewer than 100 amino acids were removed. The maximum pairwise identity of the sequences was 90% after the reduction, resulting in a final set of 575 positive and 685 negative sequence examples. The complete data set is available in FASTA-format [23] as Supplementary Material.

Then, sequences were analyzed using the sliding-window technique. The sliding window procedure divides a sequence in a number of overlapping subsequences. Starting from the N-terminal residue position, as many residues were read as determined by the window size, then the window was moved one residue position towards the C-terminus. The procedure was repeated until the C-terminus is reached. For each subsequence a score value (probability) was calculated by a machine learning classifier. For classifier training, the sequences were prepared by removal of the N-terminal amino acid (a methionine in most cases) and keeping only the N-terminal portions of length *L*. For each sequence stretch of length *L*, the appropriate number of windows with a width *W* was computed. Each amino acid residue of a single window was encoded into a unitary bit string of length 20, where a bit was set (value = 1) if its position in the string corresponds to the position of the amino acid residue and zero otherwise [24]. As a result, a sequence window of length *W* was encoded by a bit string containing *W*×20 bits with exactly *W* bits set to 1 and all other bits zero.

The input data for the machine learning algorithms consisted of (*L*−1)−*W* such bit vectors. Additionally, 575×(*L*−1−*W*) encoded sequence windows were randomly sampled from the C-terminal portions (starting at sequence position 51) of the positive sequence set and included as *pseudo*-negative training samples. The values of the length cut-off *L* and the window size *W* were systematically varied between 10 or 7 and 50 or 49, respectively.

### Machine learning classifiers

**Figure 2:**
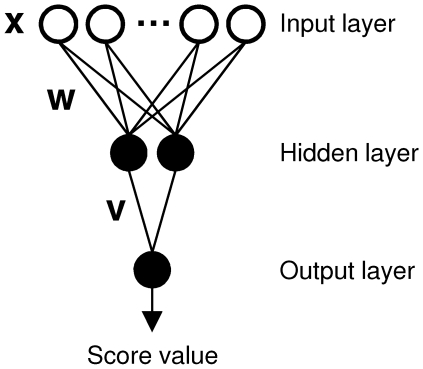
Three-layered feedforward neural networks were trained on the prediction of T3SS effector proteins. In this schematic, artificial neurons are drawn as circles (white: fan-out neuron; black: sigmoidal activation). For clarity, not all neurons are shown. The output neuron computes values between 0 and 1, which can be interpreted as the probability of an input sequence window being part of a T3SS effector signal.

We used MATLAB version R2007a [25] and SVMlight version 6.02 [26] software for training of the classifier models. The ANNs had feed-forward architecture with a single hidden neuron layer (Figure 2). All neurons in the hidden layer and the single output neuron had sigmoidal activation [16]. We used gradient descent backpropagation learning with momentum and an adaptive learning rate, as described previously [16]. Early termination of the training process was implemented by splitting the training data into two smaller training and validation sets, and stopping the training when the calculated error for the validation data rose for a predefined number of training cycles. For each set of training data, the number of hidden neurons was systematically varied from one to ten. For binary (yes/no) classification, the output of the ANN was converted to binary value using a threshold value of θ = 0.5. The overall function modelled by the implemented ANN is given by Eq. (1). 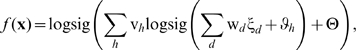
(1) where *logsig* is a sigmoidal transfer function (activation function) limiting the neuron output to the interval [0,1], *v* and *w* are the connection weights, 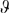 the hidden neurons' bias values, and Θ the bias of the output neuron.

The SVMs used soft margins and a radial basis function (RBF) kernel (Eq. 2). A grid search in logarithmic space was performed to find optimal values for the complexity parameter *C* and RBF parameter γ, as described [17]. The prediction of a trained SVM classifier used in this study is given by Eq. 2. 
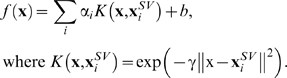
(2)


The greater *f* the higher is the probability for a compound to belong to the positive class (here: T3SS signals), **x** and **y** are sequence descriptor vectors, **x** *^sv^* are support vectors, *i.e.* data vectors that define the exact shape of the separating SVM hyperplane. The kernel function *K* defines the complexity of the surface that will be constructed. Here, we used the RBF kernel. No optimization of the choice of *K* was performed.

Training performance of both the ANNs and SVMs was evaluated by ten-fold cross-validation (leave 50% out) and calculation of the average Matthews correlation coefficient (Eq. 3) [27]

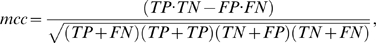
(3)where *TP, TN, FN* and *FP* denote the true-positive, true-negative, false-negative and false-positive prediction counts, respectively.

During the training process, each sequence window was considered as an individual training example and given a score, *i.e.* the ANN or SVM output. For application of the classifiers to protein sequences (obtained from bacterial genome data), an average score was computed from the individual window scores.

To compare our results to other approaches, two previously applied sets of classification rules [10], [15] were re-implemented in the programming language Python [28].

The final SVM and ANN prediction models are publicly available *via* our web server (http://www.modlab.de).

## Results and Discussion

Our study consisted of two subsequent steps: i) training of machine learning classifiers on the prediction of T3SS effectors, and ii) application of the trained classifiers on known or hypothetical proteins from available bacterial genomes, chromosomes, and plasmids.

### Machine learning and prediction performance

The starting point for both classification methods is a vector representation of the training data. Thus each training example represents a point in a vector space. During the training process, both the ANN and SVM approximate a function (hyperplane) in this vector space, which is intended to separate the positive and the negative training examples. This function can be used to classify new data points in the vector space. The multilayer perceptron used in this study employed multiple layers of artificial neurons (Figure 2) to non-linearly map the input vector to a binary classifier value. The parameters defining this mapping (weights and threshold values) are learned during the classifier training by minimizing an error function. In contrast to such ANNs, support vector machines use a so-called “kernel function” to map the training examples into a higher dimensional feature space where the examples can be separated by a hyperplane. The task of finding such a plane for a given kernel function with the constraint of maximizing the distance of the plane to the training data can be formulated as a convex optimization problem and computed efficiently [29], [30], [31].

For machine learning, it was important to realize that other transport mechanisms than T3SS also rely on N-terminal sequence signals, *e.g.* the *Sec* dependent pathway. Our dataset reflects the need to differentiate between T3SS signals and other signals, as all transportation pathways may coexist in a single species. Included are sequences with *Sec* signals, cytoplasmic proteins, and proteins exported by unknown pathways. In addition, the C-terminal sequence portions of the collected T3SS effectors were included in the negative training set. This excludes a possible general sequence bias which might be shared among the species providing the positive training data.

In order to reduce the theoretical number of 6,242,600 possible parameter sets, which results as the product of the number of sequence lengths *L*, possible window sizes *W per* sequence length, number of hidden neurons in the ANNs, and cross-validation shuffles, several attempts were made to reduce the parameter space: First, a minimal window size of *W* = 7 residues with an increment by two was used. Second, we employed a straightforward optimization protocol for the sequence length cut-off, starting with a first round of calculations using the lengths *L* = 10, 20, 30, 40 and 50 only. In the following rounds the cut-off value interval around the best performing value of the previous round was investigated in more detail. We wish to point out that due to this optimization protocol, only a single performance maximum (a “practical optimum”) can be found and it bears the risk of missing the absolute optimum.

**Figure 3:**
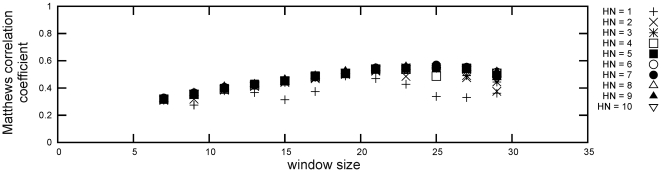
T3SS effector proteins contain a targeting signal in their N-terminal sequence portion. Performance results of the first round of neural network cross-validation for sequence length 30 and varying numbers of hidden neurons (HN) in the neural network classifiers and window sizes are shown. Values are averaged over the cross-validation folds. The data for lengths 10, 20, 40 and 50 can be found in Supplementary Figure S1.

Maximal average cross-validation performance was achieved for *L* = 30 (Figure 3), *W* = 25 and seven hidden neurons in the ANN (mcc = 0.57±0.04), although all results with more than four hidden neurons are comparable. Two more training rounds were executed (Supplementary Figures S2 and S3), using *L* = 25 and *L* = 35 for the second, and *L* = 31 to 34 for the third pass. Neither of these calculations yielded a higher performance than the maximum for *L* = 30, so the respective parameter values were employed by the final model, which was obtained by 100 training runs with randomly shuffled training data and early stop validation but no cross-validation. The performance of the best model on the complete training data is presented in Table 1. The higher accuracy likely results for three reasons: i) more data was included in the training, ii) randomized training allows for finding other performance optima, and iii) the scoring of individual sequence windows was changed to the average score over all windows.

**Table 1:** Performance of the prediction systems and sequence patterns on the complete training data (re-classification).

model	prediction for positive data (T3SS effectors)		prediction for negative data (non-effectors)		mcc
	Positive (TP)	Negative (FN)	Positive (FP)	Negative (TN)	
ANN	423 (0.74)	152 (0.26)	12 (0.02)	673 (0.98)	0.75
SVM	569 (0.99)	6 (0.01)	0 (0.0)	685 (1.0)	0.99
P1	468 (0.81)	107 (0.19)	476 (0.69)	209 (0.31)	0.14
P2	200 (0.34)	375 (0.66)	107 (0.15)	578 (0.85)	0.22

Given are absolute values and relative values in brackets. *TP*, *TN*, *FN* and *FP* denote the true-positive, true-negative, false-negative and false-positive prediction ratios, respectively. P1 and P2 indicate rule sets for prediction of type III secretion system effectors (T3SS) published by Petnicki-Ocwieja *et al.* and Vencato *et al.* [7], [12]. ANN: artificial neural network; SVM: support-vector machine.

We also studied the influence of the most N-terminal part of the training examples on the performance of ANN training. However, cleaving N-terminal parts of varying size off the training sequences reduced the performance (*cf.* Supplementary Figure S4). This suggests that the N-terminal part of the training sequences holds important information for distinguishing T3SS effectors.

**Figure 4:**
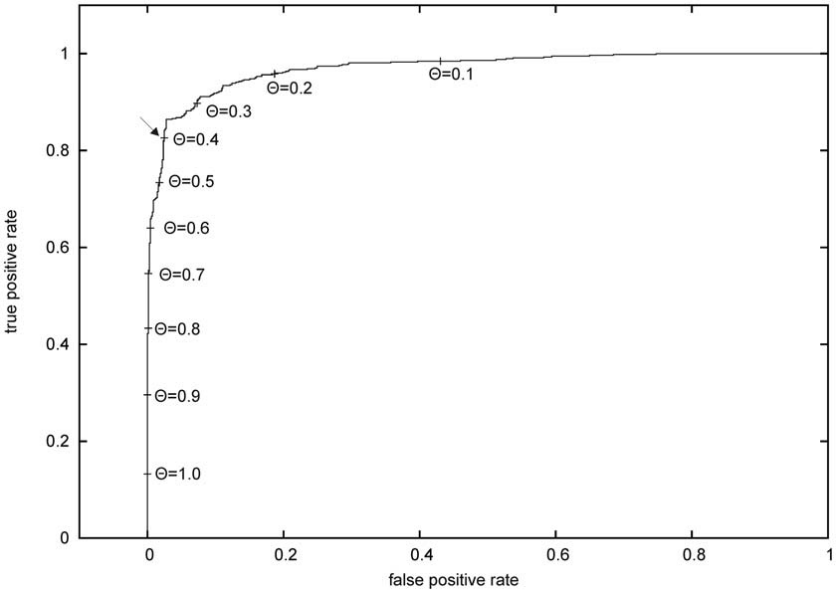
The best neural network classifier was determined by receiver-operator characteristic (ROC) analysis. The plot results from a threshold test with the final neural network model. Threshold values Θ for the predicted score ranged from 0.1 to 1.0. The threshold value of the final model (Θ  =  0.4) is marked by an arrow.

The ANN model bears an adjustable parameter, the threshold θ, which is the decision boundary for classification of the network output. It was set to 0.5 during training, but the influence of this parameter on the performance of the final model can be studied by a Receiver Operating Characteristic (ROC) threshold test [32]. The ROC curve is shown in Figure 4. The sudden flattening of the curve at a true positive ratio of about 0.85 suggests a selection of θ between 0.4 and 0.3 to optimize the true positive/false positive ratio tradeoff. For genome/predicted proteome analysis, we used the final ANN model with θ = 0.4.

Employing this parameter value for re-classification of the training data yielded an increased Matthews correlation of *mcc* = 0.82. The final classifier has a *sensitivity* of 83%, a *specificity* of 97%, and an *accuracy* of 91% [33]. As a control, we also trained neural networks on a sequence set randomly picked from the SwissProt database [18] and of the same size as our training data. A second control was done by training neural networks on the collected training data randomly divided into positive and negative examples (*Y-scrambling* test). In both experiments, no correlation between the actual and predicted class labels was observed (*mcc* = 0.0±0.0, and *mcc* = 0.003±0.018, respectively).

In addition to the neural network classifier, we trained a preliminary SVM with *L* = 30 and *W* = 25 input data. The best performing model had a complexity value of *C* = 1000 and a kernel gamma of γ = 0.01. Average cross-validation performance yielded *mcc* = 0.63±0.02. Results for the complete training data are given in Table 1. In both cases, the SVM apparently outperformed the ANN model. However, concerning its “true” predictive capabilities, it might be more appropriate to compare the SVM cross-validation performance to the ANN final model performance, as in both cases the training algorithm used only 90% of the available data (10% were employed for determination of the forced stop time point during training). In addition, the great number of support vectors (5,144 support vectors among 7,340 training vectors) in combination with the comparably large gamma value, suggest a limited generalization ability of this particular SVM model [34]. This is why we used only the ANN classifier for productive genome analysis in this study, while the SVM model served as secondary classifier.

We wish to stress that it is unlikely that the ANN will outperform an SVM solution once a good kernel will have been identified [35]. This technical optimization of the SVM kernel function was not part of our study, and is currently under investigation by us. Profile Hidden Markov Models (HMM) might also represent a method of choice for the given prediction task [36]. The present analysis was intended to provide a first cross-genomic prediction of potential T3SS effectors and certainly leaves room for future improvement. This will also have to address the interpretation of the decisive feature vector used by the machine learning classifier.

Compared to recently published residue motif rules (Table 1, rows P1 and P2) [7], [12] – whose performance was optimized by allowing for some rule violations – the performance of the ANN and SVM models is clearly superior. It should be kept in mind, however, that these rule sets were derived from a far smaller dataset and not intended for predictive purposes.

### Genome analysis and protein prediction

**Figure 5:**
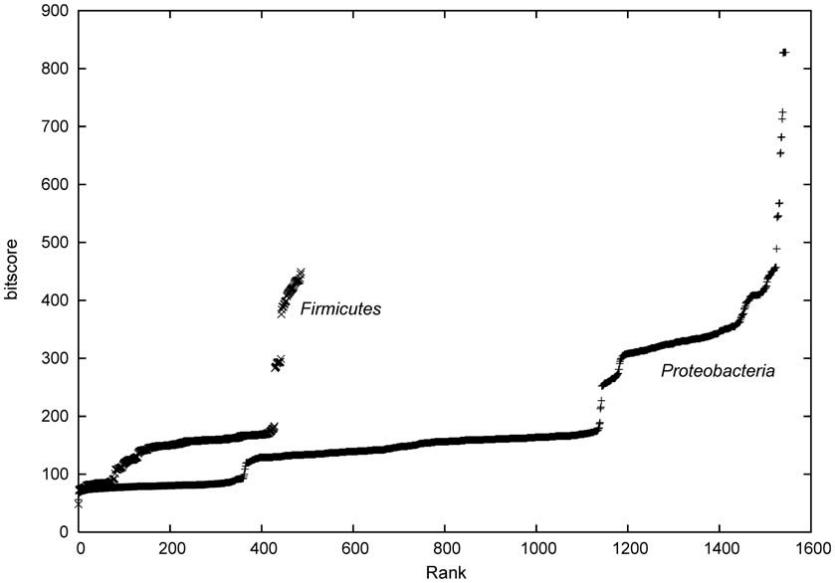
Ranking of the most significant protein alignments from all genomes was done according to their BLAST bitscore (BLOSUM62 substitution matrix, *e*-value<10^−5^). The query protein was of the *YscN* gene from *Yersinia pseudotuberculosis* (UniProt ID YSCN_YERPS).

We applied the ANN classifier to two groups of genomes collected from the RefSeq database [37]. The groups include the phylum *Proteobacteria* as Gram-negative examples and the phylum *Firmicutes* as Gram-positive examples. BLAST (BLOSUM62 substitution matrix [38], *e*-value <10^−5^) [39] was used to divide the genomes in groups depending on their possession of a homologue of the *YscN* gene from *Yersinia pseudotuberculosis* (UniProt ID YSCN_YERPS), which is known to be an integral part of a functional T3SS in *Yersinia* [40]. Notably, for all examined genomes at least one significant alignment was found, which is not expected for the Gram-positive genomes. As *YscN* is believed to be an ATPase, other enzymes with the same activity might be the reason for this finding. Consequently the BLAST bitscore threshold was set to 200 bit, as a plot of the scores suggests an inflection point around this value (Figure 5). Furthermore, sequence data from proteobacterial plasmids were separately evaluated, as only 17 plasmids seem to code for an *YscN* like protein, and these plasmids often encode virulence determinants including T3SSs, *e.g.* the *Shigella* plasmids [41].

Table 2 presents the main results of this screening exercise. All of the examined proteobacterial genomes have a comparable percentage of positive predictions (approx. 11%), which seems to be unbiased by the presence of a potential *YscN* protein, as the averages are comparable when the genomes are divided accordingly (not shown). Noticeable is a high standard deviation for the plasmid data, which might be caused by the pronounced length variation of the examined plasmids. The complete list of results shows that with regard to the relative amount of positive predictions, plasmid sequences occupy the highest ranks (*cf.* Supplementary Table S1). Many belong to genera including animal pathogenic species such as *Shigella*, *Yersinia*, *Escherichia*. Several plant pathogens are listed, *e.g. Pseudomonas syringae*, *Xanthomonas campestris*. All of the 17 plasmids holding an *YscN* homologue are present among the first 19% of the list entries. This observation clearly supports the robustness of our predictions and justifies the selection of the particular bitscore threshold applied in this study.

**Table 2:** Prediction results for the genomes (*in silico* translated sequences) of *Proteobacteria* and *Firmicutes*.

group	Number of genomes	Number of *YscN* containing genomes	Average % positive prediction (standard deviation in brackets)
*Proteobacteria*	705	284	11.5 (σ = 7.5)
proteobacterial chromosomes	405	267	10.5 (σ = 2.7)
proteobacterial plasmids	300	17	12.9 (σ = 10.8)
*Firmicutes*	213	58	6.9 (σ = 5.6)

The *Firmicutes* yield a lower overall content of *YscN* homologues relative to *Proteobacteria*. This is expected as only flagella but no standalone T3SSs exist in this phylum [42]. The average positive predictions suggest again that the T3SS signal appears to be widely spread. On the other hand, the ordering of the genomes by positive prediction content is insightful. For example, different *Clostridium* species yield a high content of positive predictions, and are also known to have flagella (*cf.* Supplementary Table S2).

The plasmid of *Yersinia* species is known to code for several virulence determinants including a T3SS and at least twelve effector proteins named “*Yersinia* outer membrane proteins” (Yops) [43]. Note that the proteins encoded on this plasmid were not included in the training data. Out of the 72 proteins encoded on the plasmid of *Yersinia enterocolitca* subsp. *enterocolitica 8081* [44], 16 are predicted to have a T3SS targeting signal (*cf.* Supplementary Table S3). Ten of these proteins are Yops and thus correctly identified. The two missing Yops are *yopQ* and *lcrV*, which received a neural network score of 0.22 and 0.3, respectively. Among the remaining six positive predictions are the chaperone of *yscN* and the *repA* and *spyB* proteins, which are involved in plasmid replication and partition [43]. These proteins are clearly false-positive predictions. Also, there are *yscP* and *yscM*, which are known to be secreted [38]. The last predicted T3SS effector is *yscW*, which is a chaperone of the T3SS component *yscC* and enables the outer membrane localization of *yscN* [45]. As *yscN* has no predicted T3SS targeting signal and *yscW* is described to be the “pilot protein” for *yscN* [45], the predicted signal of *yscW* might be responsible for the transport of both proteins.

We then took a closer look at one of the examined species, *Helicobacter pylori 26695* (RefSeq ID NC_000915), which uses flagella to propel itself and therefore has a functioning T3SS [4]. As expected, an *YscN* homologue is found, but the content of positive predictions is relatively low (6.5%). Only 93 sequences are predicted to actually contain a T3SS signal. Twelve of them are annotated as being associated to the flagellar complex, and 38 sequences are marked as “hypothetical” or lack a functional annotation (*cf.* Supplementary Table S4).

We also applied the SVM model to these *Helicobacter* data, yielding 77 candidate proteins of which 37 are annotated as “hypothetical” (not shown). 18 of these hypothetical protein sequences are shared with the ANN predictions (Table 3). BLAST [34] was used to compare these sequences with the non-redundant (nr) database of the NCBI [46]. For most of the sequences it is not possible to infer a putative function. As an exception, the sequence Hp0906 is distantly related to a putative flagellar hook protein of *Campylobacter jejuni* (alignment length = 113 residues, 36% identities).

**Table 3:** Predicted proteins from *Helicobacter pylori* strain 26695 that might be exported *via* a Type 3 Secretion System and were predicted by both ANN and SVM classifiers.

No.	Database accession codes/loci (Genbank, NCBI)	Annotation	*H. pylori* gene identifier
1	gi|15644743|ref|NP_206913.1|	Hypothetical protein	HP0113
2	gi|15644939|ref|NP_207109.1|	Hypothetical protein	HP0311
3	gi|15644995|ref|NP_207165.1|	Hypothetical protein	HP0367
4	gi|15645055|ref|NP_207225.1|	Hypothetical protein	HP0427
5	gi|15645292|ref|NP_207462.1|	Hypothetical protein	HP0668
6	gi|15645302|ref|NP_207472.1|	Hypothetical protein	HP0678
7	gi|15645498|ref|NP_207673.1|	Hypothetical protein	HP0879
8	gi|15645522|ref|NP_207698.1|	Hypothetical protein	HP0906
9	gi|15645579|ref|NP_207755.1|	Hypothetical protein	HP0963
10	gi|15645605|ref|NP_207781.1|	Hypothetical protein	HP0990
11	gi|15645679|ref|NP_207856.1|	Hypothetical protein	HP1065
12	gi|15645756|ref|NP_207933.1|	Hypothetical protein	HP1142
13	gi|15645847|ref|NP_208025.1|	Hypothetical protein	HP1233
14	gi|15646001|ref|NP_208182.1|	Hypothetical protein	HP1391
15	gi|15646018|ref|NP_208199.1|	Hypothetical protein	HP1408
16	gi|15646039|ref|NP_208221.1|	Hypothetical ATP-binding protein	HP1430
17	gi|15646108|ref|NP_208290.1|	Hypothetical protein	HP1499
18	gi|15646129|ref|NP_208311.1|	Hypothetical protein	HP1520

While the flagellum associated positive predictions can be regarded as biologically plausible and the hypothetical proteins might be effectors of a T3SS, some of the predicted signal-containing proteins are metabolic enzymes, *i.e.* the citrate synthase or biotin synthetase, which are not expected to be exported. Chromosomes of the other two strains of *Helicobacter pylori*, for which fully sequenced genomes are available (HPAG1 [47] and J99 [48]), obtain a similar predicted percentage of T3SS effectors, which also holds for the related species *Helicobacter acinonychis*, being a gastric pathogen of large felines [49]. For each of the three *Helicobacter pylori* strains ten putative flagellar components are predicted to possess a T3SS signal and share the same functional annotation. Also the obvious false-positive predictions (citrate synthase and biotin synthetase) occur for all strains.

### Conclusions

In this study we present evidence for the existence of common sequence features in the N-terminal portion (30 residues) of T3SS effectors. The existence or absence of these features can be predicted with reasonable accuracy. A low number of false positive predictions of our classifiers is an important feature, as it might help preventing unnecessary experiments when applied to selecting candidates for an experimental survey. Moreover, the predicted features seem to be universally distributed among sequences of a wide range of both Gram-negative and Gram-positive bacteria, regardless of the existence of a T3SS. Thus, we cannot be completely sure that the machine learning classifiers actually extracted directly T3SS-related and secretion-inducing features. Additional and different types of machine learning classifiers will have to be developed to address this point. In particular, we expect that thorough SVM classifier training will provide improved predictions and help understand the actually relevant sequence features. During the reviewing process of this paper two other articles [50], [51] were published which address the same problem of the prediction of T3SS effectors using a similar methodology. Interestingly, both studies lead to similar conclusions regarding the length of the putative signal on the primary protein structure and the spread of the signal among different species. Arnold *et al.* developed a naïve Bayes classifier by which up to 12% potential T3SS effectors were predicted for whole genomes [50], which is in perfect agreement with our results. These authors also demonstrate that in some cases *in silico* frame-shift mutations do not affect the predictions which might be an explanation for the hypothetical RNA encoded signal [11]. We wish to point out that our prediction system has the highest *specificity* among the presented approaches, which is an important property for prioritizing biochemical and cell biological experiments. This might be a result of the larger training data set and especially the composition of the negative training data used in our study.

Most interestingly, according to our analysis flagella T3SS and standalone T3SS seem to share the same kind of signal. Viewed from an evolutionary perspective, one might speculate that the signal evolved independently from the T3SS, maybe even without having any particular targeting function, and eventually the signal pattern was adopted by the developing T3SS for effector tagging. On the other hand, we stress that the predictions contain apparent errors, as we predict obvious cytoplasmic proteins to have a T3SS export signal. This observation leaves room for further improvements, for example by modifying the training data composition. In this context one has to keep in mind that there are certain chaperones that promote type III secretion [4], but it has not yet been determined whether both signal components (the actual sequence feature and the chaperone) are required for protein translocation or if one alone might be sufficient under certain conditions.

## Supporting Information

Figure S1 The plots present the performance results for the first round of ANN cross-validation for sequence lengths 10 (A), 20 (B), 30 (C), 40 (D) and 50(E) and varying numbers of hidden neurons and window sizes. The data values are averaged over the cross validation folds, standard deviation is not shown for clarity.
(0.05 MB PDF)

Figure S2 The graphs present performance results for the first round of ANN cross-validation for sequence lengths 25 (A) and 35 (B) and varying numbers of hidden neurons and window sizes. The data values are averaged over the cross-validation folds, standard deviations are not shown for clarity.
(0.03 MB PDF)

Figure S3 The plot presents the performance results for the first round of ANN cross-validation for sequence lengths 31 (A), 32 (B), 33 (C) and 34 (D) and varying numbers of hidden neurons and window sizes. The data values are averaged over the cross-validation folds, standard deviations are not shown for clarity.
(0.04 MB PDF)

Figure S4 The length of the N-terminal sequence portion used for classifier training has an influence on neural network performance. Results are presented forr three different lengths *L*. The *x*-axis is scaled to the fraction of removed sequence (cutoff values divided by the overall length). The performance values presented are averaged over the number of hidden neurons, the number of cross-validation shuffles, and different window sizes. Error bars denote the standard deviation. For length *L* = 30 the most N-terminal 10 and 20 residues were removed and for *L* = 40 and *L* = 50 the most N-terminal 10, 20 and 30 residues were removed. For better visualisation, this is expressed as fraction in the plot. In all cases a decrease in performance can be observed when compared to Figure S1.
(0.02 MB PDF)

Table S1 Complete list of examined protein sequence sets of *Proteobacteria*. Given is the genome name, the NCBI Refseq database identification string, the existence of an *YscN* homologue, the number of positive predictions (*P*), the number of negative predictions (*N*) and the relative number of positively predicted protein sequences (%). The list is sorted according to decreasing fractions of predicted proteins.
(0.59 MB DOC)

Table S2 Complete list of examined protein sequence sets of *Firmicutes*. Given is the genome name, the NCBI Refseq database identification string, the existence of an *YscN* homologue, the number of positive predictions (*P*), the number of negative predictions (*N*) and the relative number of positively predicted protein sequences (%). The list is sorted according to decreasing fractions of predicted proteins.
(0.20 MB DOC)

Table S3 Predicted proteins from the *Yersinia* enterocolitica strain 8081 virulence plasmid that might be exported via a Type 3 Secretion System. Higher score values indicate more reliable predictions.
(0.04 MB DOC)

Table S4 Predicted proteins from *Helicobacter pylori* strain 26695 that might be exported via a Type 3 Secretion System. Higher score values indicate more reliable predictions.
(0.08 MB DOC)
