# Correction: Prediction of Type III Secretion Signals in Genomes of Gram-Negative Bacteria

**DOI:** 10.1371/annotation/78c8fc32-b1e2-4c87-9c92-d318af980b9b

**Published:** 2009-07-15

**Authors:** Martin Löwer, Gisbert Schneider

A file was unintentionally omitted from the Supporting Information section of the published article: "Text S1. Training data." The file can be viewed here: 
