# Supplementary figures and images for: Prediction of Type III Secretion Signals in Genomes of Gram-Negative Bacteria
Source: PLoS One. 2009 Jun 15;4(6):e5917. doi: 10.1371/journal.pone.0005917 (PMC2690842; doi:10.1371/journal.pone.0005917)

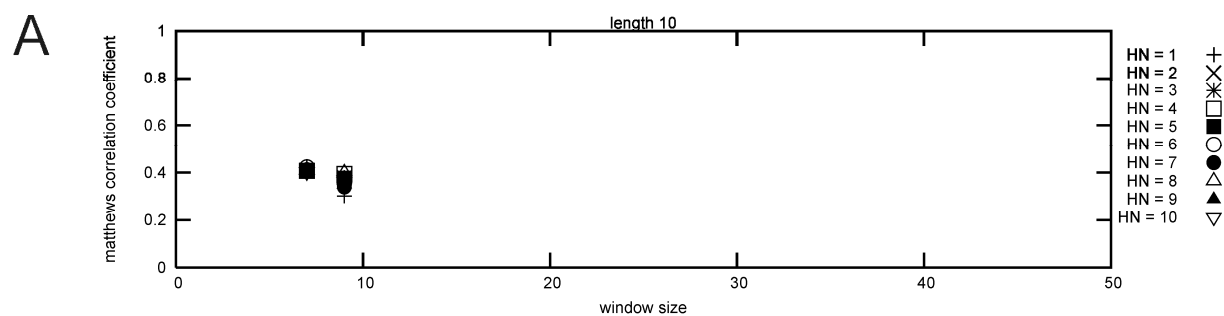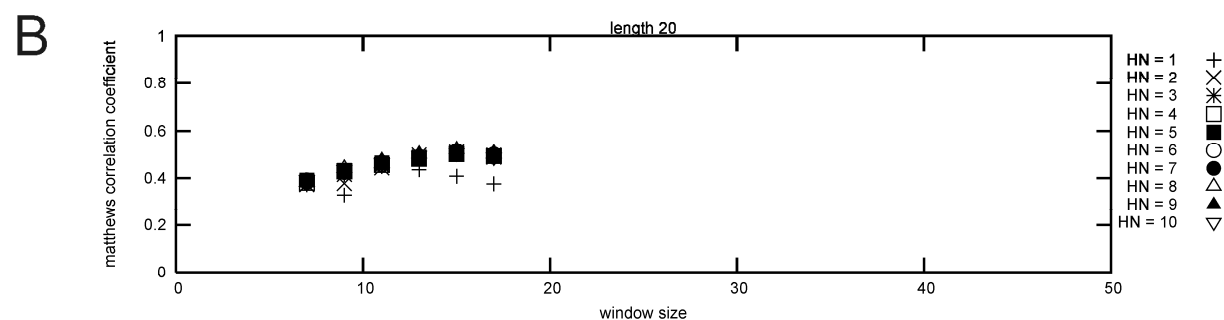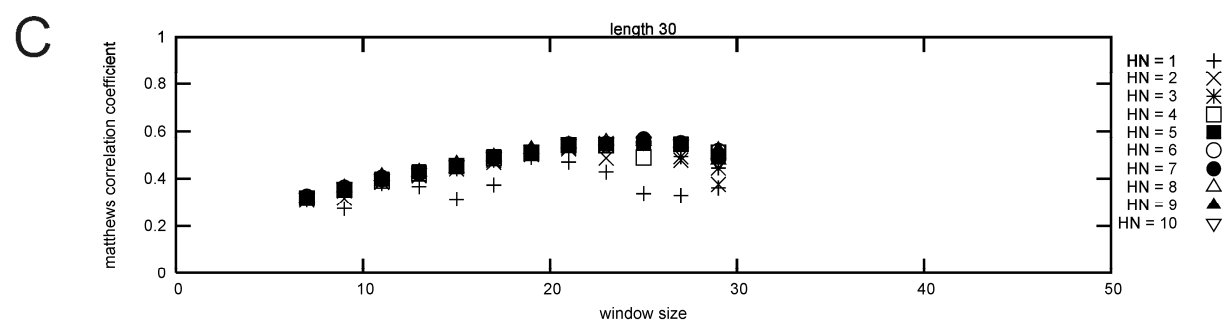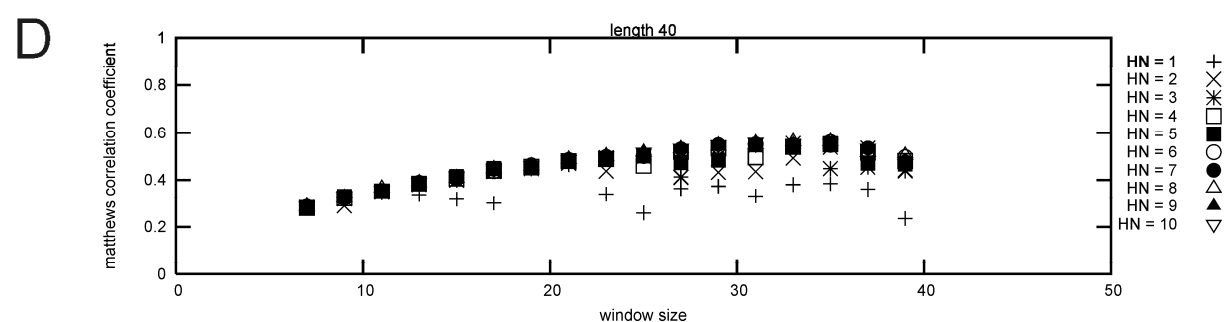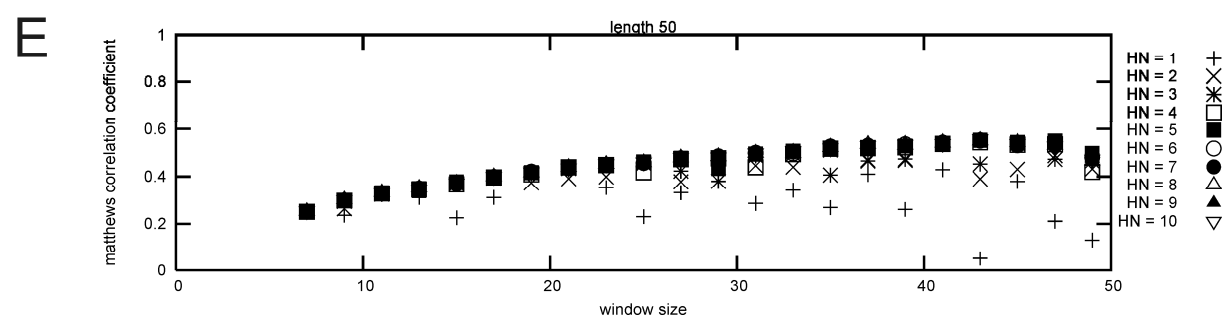

Supplement: Figure S1 — The plots present the performance results for the first round of ANN cross-validation for sequence lengths 10 (A), 20 (B), 30 (C), 40 (D) and 50(E) and varying numbers of hidden neurons and window sizes. The data values are averaged over the cross validation folds, standard deviation is not shown for clarity. (0.05 MB PDF) [file pone.0005917.s001.pdf]

A

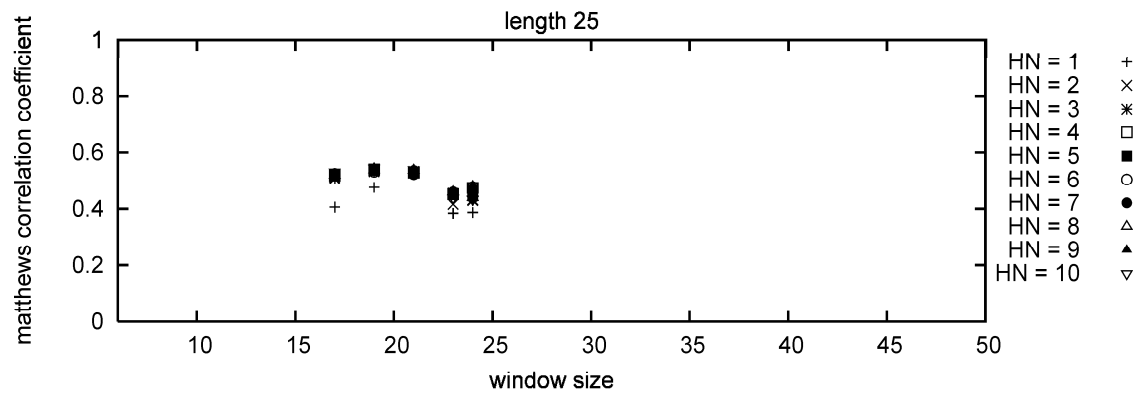

B

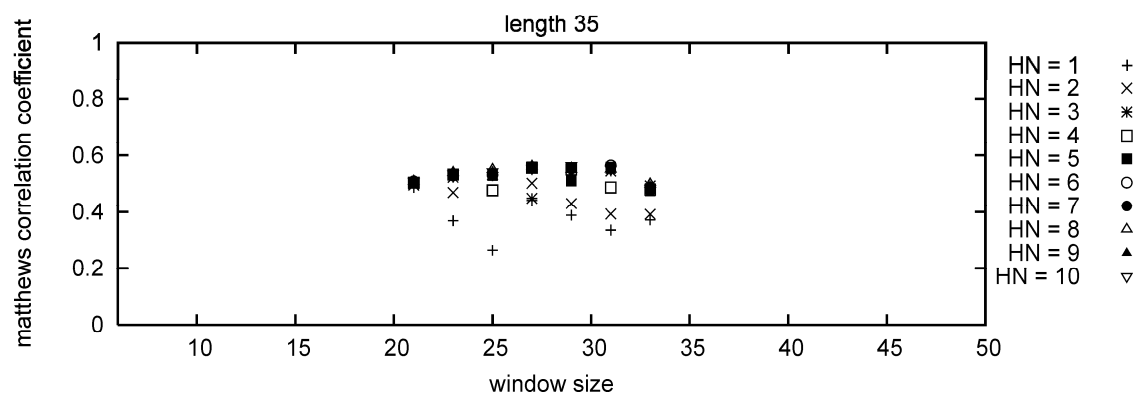

Supplement: Figure S2 — The graphs present performance results for the first round of ANN cross-validation for sequence lengths 25 (A) and 35 (B) and varying numbers of hidden neurons and window sizes. The data values are averaged over the cross-validation folds, standard deviations are not shown for clarity. (0.03 MB PDF) [file pone.0005917.s002.pdf]

A

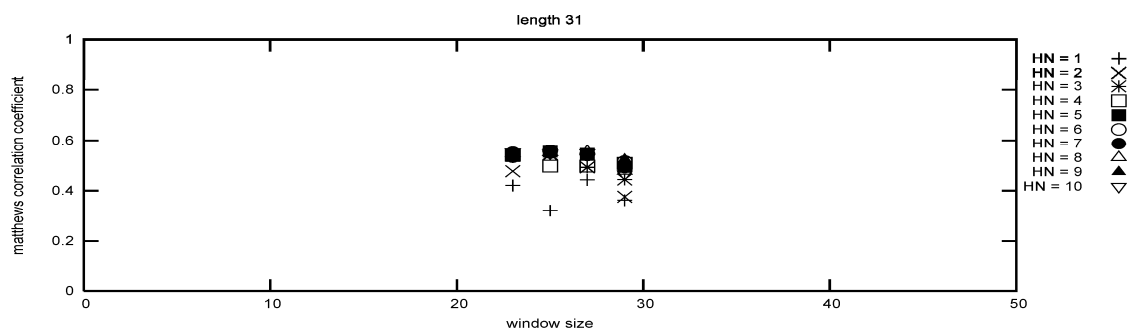

B

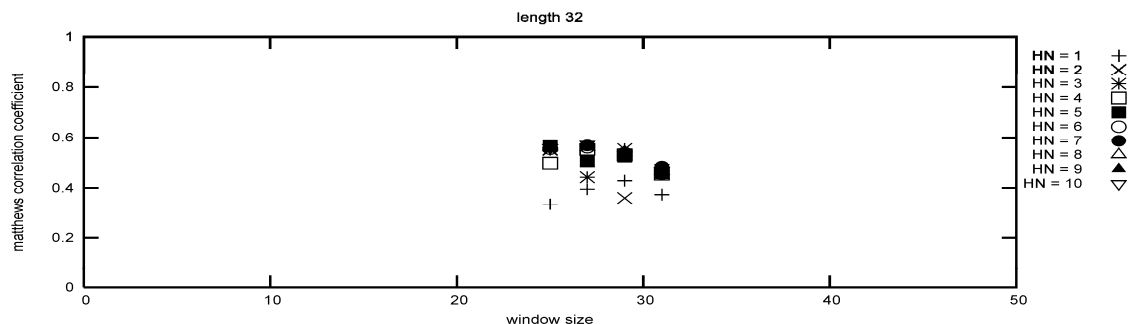

C

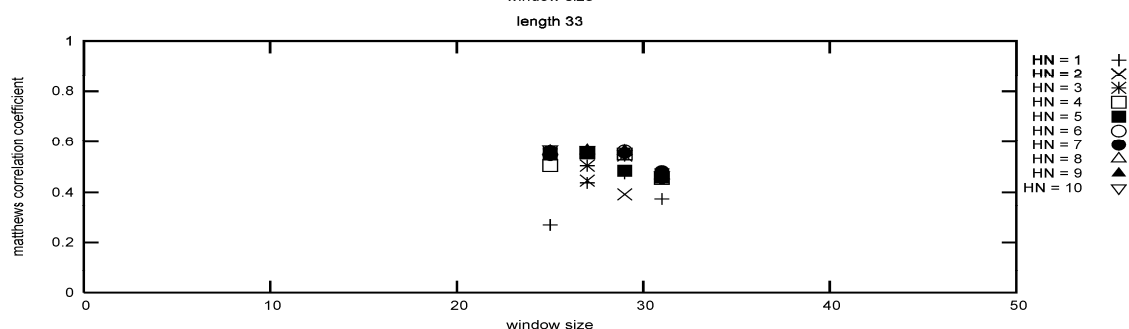

D

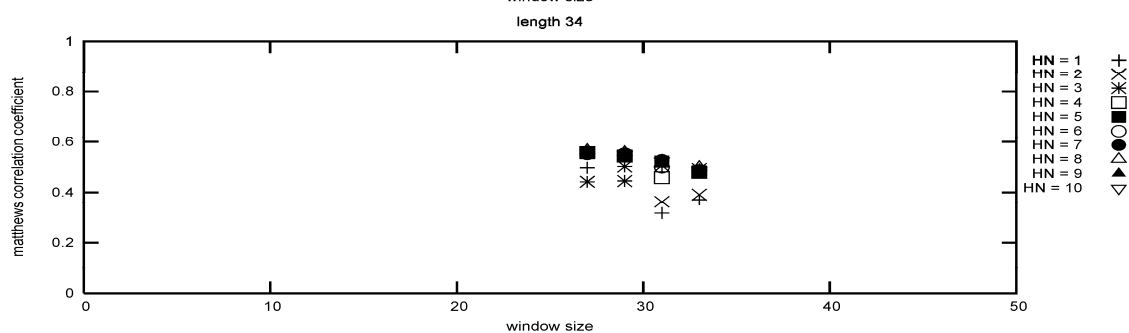

Supplement: Figure S3 — The plot presents the performance results for the first round of ANN cross-validation for sequence lengths 31 (A), 32 (B), 33 (C) and 34 (D) and varying numbers of hidden neurons and window sizes. The data values are averaged over the cross-validation folds, standard deviations are not shown for clarity. (0.04 MB PDF) [file pone.0005917.s003.pdf]

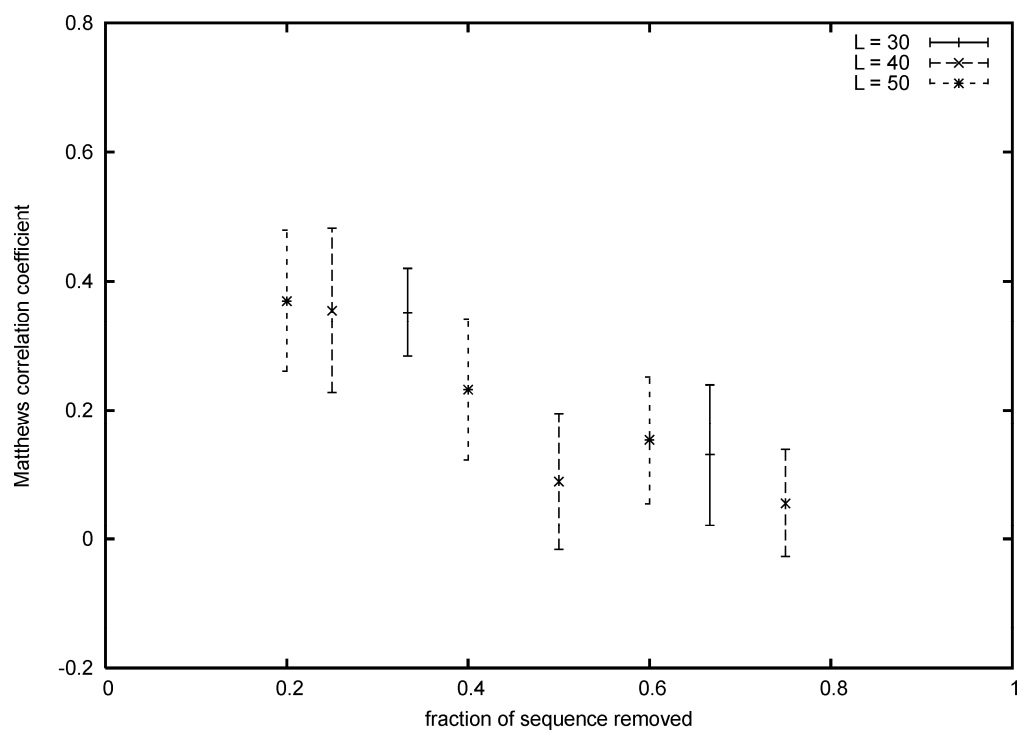

Supplement: Figure S4 — The length of the N-terminal sequence portion used for classifier training has an influence on neural network performance. Results are presented forr three different lengths L. The x-axis is scaled to the fraction of removed sequence (cutoff values divided by the overall length). The performance values presented are averaged over the number of hidden neurons, the number of cross-validation shuffles, and different window sizes. Error bars denote the standard deviation. For length L = 30 the most N-terminal 10 and 20 residues were removed and for L = 40 and L = 50 the most N-terminal 10, 20 and 30 residues were removed. For better visualisation, this is expressed as fraction in the plot. In all cases a decrease in performance can be observed when compared to Figure S1. (0.02 MB PDF) [file pone.0005917.s004.pdf]
