# Supplementary material for: Prediction of Type III Secretion Signals in Genomes of Gram-Negative Bacteria
Source: PLoS One. 2009 Jun 15;4(6):e5917. doi: 10.1371/journal.pone.0005917 (PMC2690842; doi:10.1371/journal.pone.0005917)
Supplement: Table S1 — Complete list of examined protein sequence sets of Proteobacteria. Given is the genome name, the NCBI Refseq database identification string, the existence of an YscN homologue, the number of positive predictions (P), the number of negative predictions (N) and the relative number of positively predicted protein sequences (%). The list is sorted according to decreasing fractions of predicted proteins. (0.59 MB DOC) [file pone.0005917.s005.doc]

**Table S1.** Complete list of examined protein sequence sets of *Proteobacteria*. Given is the genome name, the NCBI Refseq database identification string, the existence of an *YscN* homologue, the number of positive predictions (*P*), the number of negative predictions (*N*) and the relative number of positively predicted protein sequences (%). The list is sorted according to decreasing fractions of predicted proteins.

| **Genome name** | **NCBI ID** | ***YscN*** | ***P*** | ***N*** | **%** |
| --- | --- | --- | --- | --- | --- |
| Psychrobacter sp. PRwf-1 plasmid pRWF102 | NC_009517 | no | 1 | 0 | 1.0 |
| Xanthomonas campestris pv. vesicatoria str. 85-10 plasmid pXCV2 | NC_007504 | no | 2 | 0 | 1.0 |
| Buchnera aphidicola str. APS (Acyrthosiphon pisum) plasmid pTrp | NC_002252 | no | 2 | 1 | 0.666 |
| Roseobacter denitrificans plasmid pTB4 | NC_008389 | no | 3 | 3 | 0.5 |
| Xylella fastidiosa 9a5c plasmid pXF1.3 | NC_002489 | no | 1 | 1 | 0.5 |
| Shigella sonnei Ss046 plasmid pSS046_spA | NC_009345 | no | 2 | 3 | 0.4 |
| Escherichia coli E24377A plasmid pETEC_5 | NC_009791 | no | 1 | 2 | 0.333 |
| Buchnera aphidicola str. Bp (Baizongia pistaciae) plasmid pBBp1 | NC_004555 | no | 1 | 2 | 0.333 |
| Escherichia coli O157:H7 str. Sakai plasmid pOSAK1 | NC_002127 | no | 1 | 2 | 0.333 |
| Yersinia pestis CO92 plasmid pCD1 | NC_003131 | yes | 23 | 48 | 0.323 |
| Shigella boydii Sb227 plasmid pSB4_227 | NC_007608 | no | 43 | 106 | 0.288 |
| Sodalis glossinidius str. 'morsitans' plasmid pSG3 | NC_007715 | no | 2 | 5 | 0.285 |
| Shigella dysenteriae Sd197 plasmid pSD1_197 | NC_007607 | yes | 61 | 163 | 0.272 |
| Sphingopyxis alaskensis RB2256 F plasmid | NC_008036 | no | 8 | 22 | 0.266 |
| Yersinia pestis biovar Microtus str. 91001 plasmid pCD1 | NC_005813 | yes | 22 | 63 | 0.258 |
| Escherichia coli SMS-3-5 plasmid pSMS35_4 | NC_010486 | no | 1 | 3 | 0.25 |
| Francisella philomiragia subsp. philomiragia ATCC 25017 plasmid pFPHI01 | NC_010331 | no | 1 | 3 | 0.25 |
| Yersinia pestis Angola plasmid new_pCD | NC_010157 | yes | 22 | 66 | 0.25 |
| Shigella sonnei Ss046 plasmid pSS046_spB | NC_009346 | no | 2 | 6 | 0.25 |
| Shigella dysenteriae Sd197 plasmid pSD197_spA | NC_009344 | no | 2 | 6 | 0.25 |
| Gluconobacter oxydans 621H plasmid pGOX5 | NC_006676 | no | 1 | 3 | 0.25 |
| Shigella boydii CDC 3083-94 plasmid pBS512_211 | NC_010660 | yes | 60 | 182 | 0.247 |
| Shigella flexneri 2a str. 301 plasmid pCP301 | NC_004851 | yes | 65 | 198 | 0.247 |
| Yersinia pestis Antiqua plasmid pCD | NC_008122 | yes | 21 | 68 | 0.235 |
| Shigella sonnei Ss046 plasmid pSS_046 | NC_007385 | yes | 56 | 182 | 0.235 |
| Salmonella enterica subsp. enterica serovar Choleraesuis str. SC-B67 plasmid pSCV50 | NC_006855 | no | 12 | 39 | 0.235 |
| Yersinia pseudotuberculosis IP 32953 plasmid pYV | NC_006153 | yes | 22 | 73 | 0.231 |
| Acidiphilium cryptum JF-5 plasmid pACRY06 | NC_009472 | no | 2 | 7 | 0.222 |
| Yersinia enterocolitica subsp. enterocolitica 8081 plasmid pYVe8081 | NC_008791 | yes | 16 | 56 | 0.222 |
| Gluconobacter oxydans 621H plasmid pGOX3 | NC_006674 | no | 4 | 14 | 0.222 |
| Yersinia pestis CO92 plasmid pPCP1 | NC_003132 | no | 2 | 7 | 0.222 |
| Yersinia pestis Pestoides F plasmid CD | NC_009377 | yes | 19 | 69 | 0.215 |
| Xanthomonas axonopodis pv. citri str. 306 plasmid pXAC33 | NC_003921 | no | 9 | 33 | 0.214 |
| Escherichia coli O157:H7 EDL933 plasmid pO157 | NC_007414 | no | 21 | 78 | 0.212 |
| Burkholderia pseudomallei 1710b chromosome II | NC_007435 | yes | 546 | 2065 | 0.209 |
| Lawsonia intracellularis PHE/MN1-00 plasmid 2 | NC_008013 | no | 5 | 19 | 0.208 |
| Escherichia coli E24377A plasmid pETEC_80 | NC_009786 | no | 14 | 54 | 0.205 |
| Erwinia tasmaniensis plasmid pET46 | NC_010693 | no | 8 | 31 | 0.205 |
| Burkholderia pseudomallei 1106a chromosome II | NC_009078 | yes | 646 | 2518 | 0.204 |
| Actinobacillus pleuropneumoniae serovar 7 str. AP76 plasmid APP7_A | NC_010942 | no | 1 | 4 | 0.2 |
| Escherichia coli SMS-3-5 plasmid pSMS35_8 | NC_010485 | no | 2 | 8 | 0.2 |
| Escherichia coli E24377A plasmid pETEC_6 | NC_009789 | no | 1 | 4 | 0.2 |
| Yersinia pestis biovar Microtus str. 91001 plasmid pPCP1 | NC_005816 | no | 2 | 8 | 0.2 |
| Pseudomonas syringae pv. phaseolicola 1448A large plasmid | NC_007274 | no | 25 | 102 | 0.196 |
| Burkholderia pseudomallei 668 chromosome II | NC_009075 | yes | 644 | 2635 | 0.196 |
| Burkholderia thailandensis E264 chromosome II | NC_007650 | yes | 460 | 1898 | 0.195 |
| Desulfovibrio vulgaris subsp. vulgaris DP4 plasmid pDVUL01 | NC_008741 | yes | 29 | 121 | 0.193 |
| Methylobacterium radiotolerans JCM 2831 plasmid pMRAD07 | NC_010504 | no | 4 | 17 | 0.190 |
| Granulibacter bethesdensis CGDNIH1 | NC_008343 | no | 457 | 1980 | 0.187 |
| Psychrobacter cryohalolentis K5 | NC_007969 | no | 462 | 2005 | 0.187 |
| Psychrobacter arcticus 273-4 | NC_007204 | no | 397 | 1723 | 0.187 |
| Methylobacterium radiotolerans JCM 2831 plasmid pMRAD03 | NC_010514 | no | 8 | 35 | 0.186 |
| Burkholderia mallei SAVP1 chromosome II | NC_008784 | no | 321 | 1413 | 0.185 |
| Burkholderia pseudomallei 1710b chromosome I | NC_007434 | yes | 688 | 3048 | 0.184 |
| Pseudomonas syringae pv. phaseolicola 1448A small plasmid | NC_007275 | no | 11 | 49 | 0.183 |
| Burkholderia mallei NCTC 10247 chromosome II | NC_009079 | yes | 434 | 1942 | 0.182 |
| Xanthomonas campestris pv. vesicatoria str. 85-10 plasmid pXCV19 | NC_007505 | no | 4 | 18 | 0.181 |
| Psychrobacter sp. PRwf-1 | NC_009524 | no | 429 | 1941 | 0.181 |
| Escherichia coli APEC O1 plasmid pAPEC-O1-ColBM | NC_009837 | no | 36 | 163 | 0.180 |
| Burkholderia mallei ATCC 23344 chromosome 2 | NC_006349 | yes | 362 | 1667 | 0.178 |
| Desulfovibrio vulgaris subsp. vulgaris str. Hildenborough megaplasmid | NC_005863 | yes | 27 | 125 | 0.177 |
| Rhizobium etli CIAT 652 plasmid pB | NC_010996 | yes | 63 | 293 | 0.176 |
| Rickettsia felis URRWXCal2 plasmid pRF | NC_007110 | no | 12 | 56 | 0.176 |
| Pseudomonas syringae pv. tomato str. DC3000 plasmid pDC3000A | NC_004633 | no | 12 | 56 | 0.176 |
| Salmonella typhimurium LT2 plasmid pSLT | NC_003277 | no | 18 | 84 | 0.176 |
| Escherichia coli O157:H7 str. Sakai plasmid pO157 | NC_002128 | no | 15 | 70 | 0.176 |
| Campylobacter concisus 13826 plasmid pCCON16 | NC_009796 | no | 4 | 19 | 0.173 |
| Acidovorax avenae subsp. citrulli AAC00-1 | NC_008752 | yes | 818 | 3891 | 0.173 |
| Methylobacterium radiotolerans JCM 2831 plasmid pMRAD02 | NC_010509 | no | 9 | 43 | 0.173 |
| Rhodobacter sphaeroides 2.4.1 plasmid E | NC_009008 | no | 5 | 24 | 0.172 |
| Acinetobacter baumannii ACICU plasmid pACICU2 | NC_010606 | no | 11 | 53 | 0.171 |
| Burkholderia mallei NCTC 10229 chromosome II | NC_008835 | yes | 374 | 1803 | 0.171 |
| Escherichia coli SMS-3-5 plasmid pSMS35_130 | NC_010488 | no | 26 | 127 | 0.169 |
| Rhizobium etli CFN 42 symbiotic plasmid p42d | NC_004041 | yes | 57 | 279 | 0.169 |
| Agrobacterium tumefaciens str. C58 plasmid Ti | NC_003065 | no | 33 | 164 | 0.167 |
| Acinetobacter baumannii plasmid p2ABSDF | NC_010396 | no | 5 | 25 | 0.166 |
| Rickettsia massiliae MTU5 plasmid pRMA | NC_009897 | no | 2 | 10 | 0.166 |
| Haemophilus somnus 129PT plasmid pHS129 | NC_006298 | no | 1 | 5 | 0.166 |
| Escherichia coli UTI89 plasmid pUTI89 | NC_007941 | no | 24 | 121 | 0.165 |
| Mesorhizobium sp. BNC1 plasmid 3 | NC_008244 | no | 8 | 41 | 0.163 |
| Burkholderia xenovorans LB400 chromosome 3 | NC_007953 | no | 214 | 1098 | 0.163 |
| Rhizobium leguminosarum bv. viciae 3841 plasmid pRL7 | NC_008382 | no | 25 | 131 | 0.160 |
| Rhizobium etli CFN 42 plasmid p42a | NC_007762 | no | 28 | 147 | 0.16 |
| Xylella fastidiosa 9a5c | NC_002488 | no | 442 | 2324 | 0.159 |
| Acidiphilium cryptum JF-5 plasmid pACRY02 | NC_009468 | no | 27 | 142 | 0.159 |
| Escherichia coli E24377A plasmid pETEC_74 | NC_009790 | no | 11 | 58 | 0.159 |
| Candidatus Carsonella ruddii PV | NC_008512 | no | 29 | 153 | 0.159 |
| Rickettsia felis URRWXCal2 plasmid pRFdelta | NC_007111 | no | 7 | 37 | 0.159 |
| Acidiphilium cryptum JF-5 plasmid pACRY01 | NC_009467 | no | 27 | 144 | 0.157 |
| Dinoroseobacter shibae DFL 12 plasmid pDSHI04 | NC_009958 | no | 11 | 59 | 0.157 |
| Nitrobacter hamburgensis X14 plasmid 2 | NC_007960 | no | 27 | 145 | 0.156 |
| Gluconobacter oxydans 621H | NC_006677 | yes | 380 | 2052 | 0.156 |
| Xanthomonas oryzae pv. oryzae KACC10331 | NC_006834 | yes | 646 | 3498 | 0.155 |
| Coxiella burnetii RSA 331 plasmid QpH1 | NC_010115 | no | 7 | 38 | 0.155 |
| Burkholderia thailandensis E264 chromosome I | NC_007651 | yes | 509 | 2767 | 0.155 |
| Delftia acidovorans SPH-1 | NC_010002 | yes | 938 | 5102 | 0.155 |
| Burkholderia pseudomallei 668 chromosome I | NC_009074 | yes | 613 | 3338 | 0.155 |
| Gluconobacter oxydans 621H plasmid pGOX1 | NC_006672 | no | 25 | 138 | 0.153 |
| Nitrobacter hamburgensis X14 plasmid 3 | NC_007961 | no | 17 | 94 | 0.153 |
| Erwinia tasmaniensis plasmid pET45 | NC_010699 | no | 7 | 39 | 0.152 |
| Gluconacetobacter diazotrophicus PAl 5 plasmid pGDIPal5I | NC_010124 | no | 8 | 45 | 0.150 |
| Sphingomonas wittichii RW1 plasmid pSWIT01 | NC_009507 | no | 43 | 242 | 0.150 |
| Xanthomonas axonopodis pv. citri str. 306 plasmid pXAC64 | NC_003922 | no | 11 | 62 | 0.150 |
| Xylella fastidiosa M23 plasmid pXFAS01 | NC_010579 | no | 6 | 34 | 0.15 |
| Yersinia pestis Angola plasmid pMT-pPCP | NC_010158 | no | 18 | 102 | 0.15 |
| Burkholderia cenocepacia AU 1054 chromosome 2 | NC_008061 | yes | 370 | 2102 | 0.149 |
| Burkholderia multivorans ATCC 17616 chromosome 3 | NC_010087 | no | 123 | 700 | 0.149 |
| Burkholderia mallei SAVP1 chromosome I | NC_008785 | yes | 516 | 2939 | 0.149 |
| Xanthomonas campestris pv. campestris | NC_010688 | yes | 666 | 3801 | 0.149 |
| Burkholderia cepacia AMMD chromosome 3 | NC_008392 | yes | 151 | 862 | 0.149 |
| Sphingomonas wittichii RW1 plasmid pSWIT02 | NC_009508 | no | 31 | 179 | 0.147 |
| Burkholderia pseudomallei 1106a chromosome I | NC_009076 | yes | 592 | 3427 | 0.147 |
| Polaromonas sp. JS666 plasmid 1 | NC_007949 | no | 48 | 278 | 0.147 |
| Gluconacetobacter diazotrophicus PAl 5 | NC_010125 | yes | 556 | 3222 | 0.147 |
| Escherichia coli E24377A plasmid pETEC_73 | NC_009788 | no | 10 | 58 | 0.147 |
| Nitrobacter hamburgensis X14 plasmid 1 | NC_007959 | no | 35 | 204 | 0.146 |
| Sorangium cellulosum 'So ce 56' | NC_010162 | no | 1363 | 8021 | 0.145 |
| Burkholderia cenocepacia HI2424 chromosome 2 | NC_008543 | yes | 390 | 2296 | 0.145 |
| Vibrio vulnificus YJ016 plasmid pYJ016 | NC_005128 | no | 10 | 59 | 0.144 |
| Myxococcus xanthus DK 1622 | NC_008095 | yes | 1051 | 6280 | 0.143 |
| Burkholderia sp. 383 chromosome 3 | NC_007509 | no | 173 | 1036 | 0.143 |
| Gluconacetobacter diazotrophicus PAl 5 plasmid pGDIPal5II | NC_010123 | no | 3 | 18 | 0.142 |
| Aeromonas salmonicida subsp. salmonicida A449 plasmid pAsa5 | NC_009350 | yes | 22 | 132 | 0.142 |
| Rhodopseudomonas palustris CGA009 plasmid pRPA | NC_005297 | no | 1 | 6 | 0.142 |
| Buchnera aphidicola str. APS (Acyrthosiphon pisum) plasmid pLeu | NC_002253 | no | 1 | 6 | 0.142 |
| Burkholderia vietnamiensis G4 chromosome 3 | NC_009254 | no | 159 | 955 | 0.142 |
| Bradyrhizobium japonicum USDA 110 | NC_004463 | yes | 1186 | 7131 | 0.142 |
| Burkholderia multivorans ATCC 17616 chromosome 2 | NC_010086 | yes | 305 | 1847 | 0.141 |
| Vibrio harveyi ATCC BAA-1116 plasmid pVIBHAR | NC_009777 | no | 17 | 103 | 0.141 |
| Burkholderia phymatum STM815 chromosome 1 | NC_010622 | yes | 434 | 2638 | 0.141 |
| Burkholderia mallei NCTC 10247 chromosome I | NC_009080 | yes | 489 | 2987 | 0.140 |
| Salmonella enterica subsp. enterica serovar Typhi str. CT18 plasmid pHCM2 | NC_003385 | no | 18 | 110 | 0.140 |
| Shewanella sp. ANA-3 plasmid 1 | NC_008573 | no | 35 | 214 | 0.140 |
| Xanthomonas campestris pv. vesicatoria str. 85-10 | NC_007508 | yes | 630 | 3857 | 0.140 |
| Xylella fastidiosa Temecula1 | NC_004556 | no | 284 | 1750 | 0.139 |
| Ralstonia solanacearum GMI1000 plasmid pGMI1000MP | NC_003296 | yes | 234 | 1442 | 0.139 |
| Coxiella burnetii RSA 493 plasmid pQpH1 | NC_004704 | no | 5 | 31 | 0.138 |
| Burkholderia xenovorans LB400 chromosome 2 | NC_007952 | no | 411 | 2549 | 0.138 |
| Yersinia pestis CO92 plasmid pMT1 | NC_003134 | no | 14 | 87 | 0.138 |
| Azorhizobium caulinodans ORS 571 | NC_009937 | yes | 642 | 4075 | 0.136 |
| Burkholderia mallei NCTC 10229 chromosome I | NC_008836 | yes | 453 | 2880 | 0.135 |
| Burkholderia cepacia AMMD chromosome 1 | NC_008390 | yes | 436 | 2777 | 0.135 |
| Mesorhizobium sp. BNC1 plasmid 1 | NC_008242 | no | 43 | 274 | 0.135 |
| Zymomonas mobilis subsp. mobilis ZM4 | NC_006526 | no | 271 | 1727 | 0.135 |
| Polaromonas sp. JS666 plasmid 2 | NC_007950 | no | 42 | 268 | 0.135 |
| Orientia tsutsugamushi Boryong | NC_009488 | no | 160 | 1022 | 0.135 |
| Burkholderia xenovorans LB400 chromosome 1 | NC_007951 | yes | 599 | 3831 | 0.135 |
| Verminephrobacter eiseniae EF01-2 | NC_008786 | yes | 663 | 4245 | 0.135 |
| Xanthobacter autotrophicus Py2 plasmid pXAUT01 | NC_009717 | no | 39 | 250 | 0.134 |
| Burkholderia mallei ATCC 23344 chromosome 1 | NC_006348 | yes | 403 | 2592 | 0.134 |
| Photobacterium profundum SS9 plasmid pPBPR1 | NC_005871 | no | 9 | 58 | 0.134 |
| Xanthomonas axonopodis pv. citri str. 306 | NC_003919 | yes | 577 | 3735 | 0.133 |
| Caulobacter sp. K31 plasmid pCAUL02 | NC_010333 | no | 22 | 143 | 0.133 |
| Citrobacter koseri ATCC BAA-895 plasmid pCKO3 | NC_009793 | no | 2 | 13 | 0.133 |
| Acidovorax sp. JS42 | NC_008782 | yes | 534 | 3473 | 0.133 |
| Rhodoferax ferrireducens T118 plasmid1 | NC_007901 | no | 33 | 215 | 0.133 |
| Burkholderia sp. 383 chromosome 1 | NC_007510 | yes | 443 | 2891 | 0.132 |
| Klebsiella pneumoniae subsp. pneumoniae MGH 78578 plasmid pKPN5 | NC_009651 | no | 13 | 85 | 0.132 |
| Burkholderia sp. 383 chromosome 2 | NC_007511 | no | 421 | 2753 | 0.132 |
| Burkholderia vietnamiensis G4 chromosome 2 | NC_009255 | yes | 277 | 1819 | 0.132 |
| Xanthomonas campestris pv. campestris str. 8004 | NC_007086 | yes | 564 | 3709 | 0.131 |
| Salmonella enterica subsp. enterica serovar Typhi str. CT18 plasmid pHCM1 | NC_003384 | no | 31 | 204 | 0.131 |
| Bradyrhizobium sp. BTAi1 plasmid pBBta01 | NC_009475 | no | 30 | 198 | 0.131 |
| Xanthomonas campestris pv. campestris str. ATCC 33913 | NC_003902 | yes | 549 | 3632 | 0.131 |
| Vibrio harveyi ATCC BAA-1116 chromosome II | NC_009784 | yes | 311 | 2063 | 0.131 |
| Ralstonia eutropha JMP134 chromosome 1 | NC_007347 | no | 450 | 2989 | 0.130 |
| Polaromonas naphthalenivorans CJ2 | NC_008781 | no | 534 | 3550 | 0.130 |
| Shewanella baltica OS155 plasmid pSbal01 | NC_009035 | no | 12 | 80 | 0.130 |
| Klebsiella pneumoniae subsp. pneumoniae MGH 78578 plasmid pKPN4 | NC_009650 | no | 16 | 107 | 0.130 |
| Brucella melitensis 16M chromosome II | NC_003318 | yes | 148 | 991 | 0.129 |
| Acidiphilium cryptum JF-5 plasmid pACRY03 | NC_009469 | no | 10 | 67 | 0.129 |
| Burkholderia phymatum STM815 plasmid pBPHY02 | NC_010627 | no | 58 | 391 | 0.129 |
| Leptothrix cholodnii SP-6 | NC_010524 | yes | 563 | 3800 | 0.129 |
| Methylobacterium sp. 4-46 plasmid pM44601 | NC_010373 | no | 8 | 54 | 0.129 |
| Xylella fastidiosa M12 | NC_010513 | no | 271 | 1833 | 0.128 |
| Methylibium petroleiphilum PM1 plasmid RPME01 | NC_008826 | no | 81 | 549 | 0.128 |
| Pseudomonas syringae pv. tomato str. DC3000 plasmid pDC3000B | NC_004632 | no | 9 | 61 | 0.128 |
| Rhodobacter sphaeroides ATCC 17025 plasmid pRSPA03 | NC_009431 | no | 14 | 95 | 0.128 |
| Burkholderia ambifaria MC40-6 chromosome 3 | NC_010557 | yes | 124 | 843 | 0.128 |
| Mesorhizobium loti MAFF303099 plasmid pMLa | NC_002679 | no | 41 | 279 | 0.128 |
| Polaromonas naphthalenivorans CJ2 plasmid pPNAP01 | NC_008757 | no | 39 | 266 | 0.127 |
| Acinetobacter baumannii | NC_010400 | no | 372 | 2541 | 0.127 |
| Burkholderia vietnamiensis G4 chromosome 1 | NC_009256 | yes | 417 | 2857 | 0.127 |
| Desulfovibrio vulgaris subsp. vulgaris str. Hildenborough | NC_002937 | yes | 429 | 2950 | 0.126 |
| Ehrlichia chaffeensis str. Arkansas | NC_007799 | no | 140 | 965 | 0.126 |
| Burkholderia cepacia AMMD chromosome 2 | NC_008391 | yes | 297 | 2049 | 0.126 |
| Shigella boydii Sb227 | NC_007613 | no | 523 | 3611 | 0.126 |
| Burkholderia cenocepacia AU 1054 chromosome 1 | NC_008060 | yes | 375 | 2590 | 0.126 |
| Cupriavidus taiwanensis chromosome 1 | NC_010528 | no | 67 | 464 | 0.126 |
| Ralstonia metallidurans CH34 chromosome 1 | NC_007973 | no | 454 | 3147 | 0.126 |
| Polaromonas sp. JS666 | NC_007948 | no | 606 | 4211 | 0.125 |
| Burkholderia cenocepacia MC0-3 chromosome 2 | NC_010515 | yes | 351 | 2444 | 0.125 |
| Rhodospirillum rubrum ATCC 11170 | NC_007643 | yes | 475 | 3316 | 0.125 |
| Cupriavidus taiwanensis plasmid pRALTA | NC_010529 | no | 65 | 454 | 0.125 |
| Yersinia pestis KIM | NC_004088 | yes | 511 | 3575 | 0.125 |
| Acinetobacter baumannii plasmid p1ABSDF | NC_010395 | no | 1 | 7 | 0.125 |
| Yersinia pseudotuberculosis IP 31758 plasmid_153kb | NC_009705 | no | 17 | 119 | 0.125 |
| Burkholderia cenocepacia AU 1054 chromosome 3 | NC_008062 | no | 130 | 910 | 0.125 |
| Wolbachia pipientis | NC_010981 | no | 159 | 1116 | 0.124 |
| Ralstonia metallidurans CH34 plasmid 1 | NC_007971 | no | 30 | 211 | 0.124 |
| Mesorhizobium loti MAFF303099 plasmid pMLb | NC_002682 | no | 26 | 183 | 0.124 |
| Marinobacter aquaeolei VT8 plasmid pMAQU02 | NC_008739 | no | 25 | 176 | 0.124 |
| Azoarcus sp. EbN1 | NC_006513 | no | 514 | 3619 | 0.124 |
| Cupriavidus taiwanensis chromosome 2 | NC_010530 | no | 62 | 438 | 0.124 |
| Burkholderia pseudomallei K96243 chromosome 2 | NC_006351 | yes | 288 | 2041 | 0.123 |
| Rhodopseudomonas palustris CGA009 | NC_005296 | yes | 594 | 4219 | 0.123 |
| Shewanella baltica OS195 plasmid pS19502 | NC_009999 | no | 9 | 64 | 0.123 |
| Dinoroseobacter shibae DFL 12 plasmid pDSHI05 | NC_009959 | no | 9 | 64 | 0.123 |
| Ralstonia eutropha H16 chromosome 1 | NC_008313 | no | 450 | 3201 | 0.123 |
| Burkholderia cenocepacia HI2424 chromosome 1 | NC_008542 | yes | 389 | 2770 | 0.123 |
| Ralstonia eutropha JMP134 megaplasmid | NC_007336 | no | 63 | 449 | 0.123 |
| Orientia tsutsugamushi str. Ikeda | NC_010793 | no | 242 | 1725 | 0.123 |
| Burkholderia phymatum STM815 chromosome 2 | NC_010623 | no | 288 | 2061 | 0.122 |
| Shewanella baltica OS195 plasmid pS19503 | NC_010000 | no | 6 | 43 | 0.122 |
| Xanthomonas campestris pv. vesicatoria str. 85-10 plasmid pXCV183 | NC_007507 | no | 21 | 151 | 0.122 |
| Burkholderia multivorans ATCC 17616 chromosome 1 | NC_010084 | yes | 384 | 2762 | 0.122 |
| Ralstonia metallidurans CH34 chromosome 2 | NC_007974 | yes | 282 | 2031 | 0.121 |
| Burkholderia vietnamiensis G4 plasmid pBVIE02 | NC_009227 | no | 32 | 231 | 0.121 |
| Methylobacterium extorquens PA1 | NC_010172 | yes | 587 | 4242 | 0.121 |
| Candidatus Blochmannia pennsylvanicus str. BPEN | NC_007292 | no | 74 | 536 | 0.121 |
| Campylobacter concisus 13826 plasmid pCCON31 | NC_009795 | no | 4 | 29 | 0.121 |
| Burkholderia cenocepacia HI2424 chromosome 3 | NC_008544 | no | 111 | 807 | 0.120 |
| Yersinia pestis KIM plasmid pMT-1 | NC_004838 | no | 14 | 102 | 0.120 |
| Ralstonia eutropha H16 chromosome 2 | NC_008314 | yes | 308 | 2247 | 0.120 |
| Paracoccus denitrificans PD1222 chromosome 2 | NC_008687 | no | 200 | 1462 | 0.120 |
| Ralstonia eutropha JMP134 chromosome 2 | NC_007348 | yes | 289 | 2118 | 0.120 |
| Burkholderia pseudomallei K96243 chromosome 1 | NC_006350 | yes | 408 | 2991 | 0.120 |
| Magnetospirillum magneticum AMB-1 | NC_007626 | yes | 546 | 4013 | 0.119 |
| Coxiella burnetii Dugway 5J108-111 plasmid pQpDG | NC_009726 | no | 8 | 59 | 0.119 |
| Rhodobacter sphaeroides ATCC 17025 plasmid pRSPA02 | NC_009430 | no | 32 | 236 | 0.119 |
| Yersinia pestis Angola | NC_010159 | yes | 458 | 3379 | 0.119 |
| Nitrobacter hamburgensis X14 | NC_007964 | yes | 454 | 3350 | 0.119 |
| Yersinia pseudotuberculosis IP 32953 plasmid pYptb32953 | NC_006154 | no | 5 | 37 | 0.119 |
| Shigella flexneri 2a str. 2457T | NC_004741 | yes | 483 | 3578 | 0.118 |
| Methylibium petroleiphilum PM1 | NC_008825 | yes | 453 | 3366 | 0.118 |
| Rhodopseudomonas palustris BisB5 | NC_007958 | yes | 518 | 3879 | 0.117 |
| Desulfovibrio vulgaris subsp. vulgaris DP4 | NC_008751 | yes | 346 | 2595 | 0.117 |
| Xylella fastidiosa M23 | NC_010577 | no | 253 | 1908 | 0.117 |
| Brucella melitensis 16M chromosome I | NC_003317 | no | 241 | 1818 | 0.117 |
| Burkholderia phymatum STM815 plasmid pBPHY01 | NC_010625 | no | 190 | 1436 | 0.116 |
| Xanthomonas oryzae pv. oryzae MAFF 311018 | NC_007705 | yes | 509 | 3863 | 0.116 |
| Rhodopseudomonas palustris HaA2 | NC_007778 | yes | 545 | 4138 | 0.116 |
| Yersinia pestis biovar Microtus str. 91001 | NC_005810 | yes | 453 | 3442 | 0.116 |
| Xanthomonas campestris pv. vesicatoria str. 85-10 plasmid pXCV38 | NC_007506 | no | 5 | 38 | 0.116 |
| Stenotrophomonas maltophilia K279a | NC_010943 | yes | 509 | 3877 | 0.116 |
| Nitrobacter winogradskyi Nb-255 | NC_007406 | yes | 362 | 2760 | 0.115 |
| Shigella flexneri 2a str. 301 | NC_004337 | yes | 484 | 3693 | 0.115 |
| Desulfovibrio desulfuricans G20 | NC_007519 | yes | 437 | 3338 | 0.115 |
| Burkholderia ambifaria MC40-6 chromosome 1 | NC_010551 | yes | 355 | 2719 | 0.115 |
| Nitrosomonas eutropha C91 plasmid2 | NC_008342 | no | 6 | 46 | 0.115 |
| Yersinia pestis Nepal516 plasmid pMT | NC_008118 | no | 12 | 92 | 0.115 |
| Lawsonia intracellularis PHE/MN1-00 plasmid 3 | NC_008014 | no | 12 | 92 | 0.115 |
| Xanthobacter autotrophicus Py2 | NC_009720 | no | 547 | 4199 | 0.115 |
| Cellvibrio japonicus Ueda107 | NC_010995 | yes | 432 | 3322 | 0.115 |
| Yersinia pestis biovar Microtus str. 91001 plasmid pMT1 | NC_005815 | no | 14 | 108 | 0.114 |
| Yersinia pestis Pestoides F plasmid MT | NC_009378 | no | 15 | 116 | 0.114 |
| Pseudoalteromonas atlantica T6c | NC_008228 | yes | 490 | 3791 | 0.114 |
| Rhodopseudomonas palustris BisB18 | NC_007925 | yes | 558 | 4328 | 0.114 |
| Candidatus Vesicomyosocius okutanii HA | NC_009465 | no | 107 | 830 | 0.114 |
| Azoarcus sp. EbN1 plasmid 1 | NC_006823 | no | 31 | 241 | 0.113 |
| Ralstonia solanacearum GMI1000 | NC_003295 | no | 392 | 3048 | 0.113 |
| Acidiphilium cryptum JF-5 | NC_009484 | no | 349 | 2714 | 0.113 |
| Geobacter lovleyi SZ plasmid pGLOV01 | NC_010815 | no | 9 | 70 | 0.113 |
| Beijerinckia indica subsp. indica ATCC 9039 plasmid pBIND01 | NC_010580 | no | 20 | 156 | 0.113 |
| Burkholderia ambifaria MC40-6 chromosome 2 | NC_010552 | yes | 269 | 2113 | 0.112 |
| Shigella sonnei Ss046 | NC_007384 | yes | 476 | 3743 | 0.112 |
| Marinobacter aquaeolei VT8 plasmid pMAQU01 | NC_008738 | no | 24 | 189 | 0.112 |
| Jannaschia sp. CCS1 plasmid1 | NC_007801 | no | 8 | 63 | 0.112 |
| Methylococcus capsulatus str. Bath | NC_002977 | no | 333 | 2623 | 0.112 |
| Vibrio harveyi ATCC BAA-1116 chromosome I | NC_009783 | yes | 401 | 3160 | 0.112 |
| Pseudomonas stutzeri A1501 | NC_009434 | yes | 464 | 3664 | 0.112 |
| Shigella boydii CDC 3083-94 | NC_010658 | no | 477 | 3769 | 0.112 |
| Rickettsia rickettsii str. 'Sheila Smith' | NC_009882 | no | 151 | 1194 | 0.112 |
| Rhodobacter sphaeroides ATCC 17029 plasmid pRSPH01 | NC_009040 | no | 12 | 95 | 0.112 |
| Caulobacter crescentus CB15 | NC_002696 | yes | 419 | 3318 | 0.112 |
| Burkholderia cenocepacia MC0-3 chromosome 3 | NC_010512 | no | 118 | 935 | 0.112 |
| Bordetella petrii DSM 12804 | NC_010170 | yes | 562 | 4465 | 0.111 |
| Ehrlichia ruminantium str. Gardel | NC_006831 | no | 106 | 844 | 0.111 |
| Bartonella tribocorum CIP 105476 plasmidBtr | NC_010160 | no | 2 | 16 | 0.111 |
| Rhodopseudomonas palustris BisA53 | NC_008435 | yes | 542 | 4336 | 0.111 |
| Yersinia pestis Antiqua plasmid pPCP | NC_008121 | no | 1 | 8 | 0.111 |
| Yersinia pestis Nepal516 plasmid pPCP | NC_008119 | no | 1 | 8 | 0.111 |
| Burkholderia cenocepacia MC0-3 chromosome 1 | NC_010508 | yes | 351 | 2809 | 0.111 |
| Rhizobium leguminosarum bv. viciae 3841 plasmid pRL10 | NC_008381 | no | 49 | 393 | 0.110 |
| Silicibacter pomeroyi DSS-3 megaplasmid | NC_006569 | no | 49 | 393 | 0.110 |
| Janthinobacterium sp. Marseille | NC_009659 | yes | 409 | 3288 | 0.110 |
| Colwellia psychrerythraea 34H | NC_003910 | yes | 542 | 4368 | 0.110 |
| Ehrlichia ruminantium str. Welgevonden | NC_005295 | no | 98 | 790 | 0.110 |
| Bartonella quintana str. Toulouse | NC_005955 | no | 126 | 1016 | 0.110 |
| Polynucleobacter necessarius STIR1 | NC_010531 | no | 166 | 1342 | 0.110 |
| Rhodobacter sphaeroides 2.4.1 plasmid B | NC_007488 | no | 11 | 89 | 0.11 |
| Ochrobactrum anthropi ATCC 49188 plasmid pOANT02 | NC_009670 | no | 10 | 81 | 0.109 |
| Novosphingobium aromaticivorans DSM 12444 plasmid pNL1 | NC_009426 | no | 20 | 162 | 0.109 |
| Rhizobium etli CFN 42 plasmid p42e | NC_007765 | no | 50 | 405 | 0.109 |
| Pseudoalteromonas haloplanktis TAC125 chromosome II | NC_007482 | no | 60 | 486 | 0.109 |
| Rickettsia rickettsii str. Iowa | NC_010263 | no | 152 | 1232 | 0.109 |
| Rhodobacter sphaeroides 2.4.1 plasmid C | NC_007489 | no | 9 | 73 | 0.109 |
| Ehrlichia ruminantium str. Welgevonden | NC_006832 | no | 105 | 853 | 0.109 |
| Yersinia pseudotuberculosis IP 31758 plasmid_59kb | NC_009704 | no | 7 | 57 | 0.109 |
| Xylella fastidiosa 9a5c plasmid pXF51 | NC_002490 | no | 7 | 57 | 0.109 |
| Lawsonia intracellularis PHE/MN1-00 | NC_008011 | yes | 129 | 1051 | 0.109 |
| Neisseria gonorrhoeae NCCP11945 | NC_011035 | no | 291 | 2371 | 0.109 |
| Bradyrhizobium sp. ORS278 | NC_009445 | yes | 734 | 5983 | 0.109 |
| Bartonella bacilliformis KC583 | NC_008783 | yes | 140 | 1143 | 0.109 |
| Rhodoferax ferrireducens T118 | NC_007908 | yes | 455 | 3715 | 0.109 |
| Nitrosomonas eutropha C91 plasmid1 | NC_008341 | no | 6 | 49 | 0.109 |
| Brucella canis ATCC 23365 chromosome II | NC_010104 | yes | 125 | 1024 | 0.108 |
| Anaplasma marginale str. St. Maries | NC_004842 | no | 103 | 846 | 0.108 |
| Rhizobium etli CIAT 652 | NC_010994 | yes | 470 | 3873 | 0.108 |
| Shigella flexneri 5 str. 8401 | NC_008258 | yes | 445 | 3670 | 0.108 |
| Burkholderia vietnamiensis G4 plasmid pBVIE05 | NC_009226 | no | 12 | 99 | 0.108 |
| Herminiimonas arsenicoxydans | NC_009138 | yes | 359 | 2966 | 0.107 |
| Yersinia pseudotuberculosis IP 31758 | NC_009708 | yes | 444 | 3680 | 0.107 |
| Chromohalobacter salexigens DSM 3043 | NC_007963 | yes | 354 | 2944 | 0.107 |
| Mesorhizobium loti MAFF303099 | NC_002678 | yes | 723 | 6020 | 0.107 |
| Acinetobacter baumannii ACICU plasmid pACICU1 | NC_010605 | no | 3 | 25 | 0.107 |
| Roseobacter denitrificans plasmid pTB2 | NC_008387 | no | 6 | 50 | 0.107 |
| Cupriavidus necator megaplasmid pHG1 | NC_005241 | no | 45 | 375 | 0.107 |
| Ehrlichia canis str. Jake | NC_007354 | no | 99 | 826 | 0.107 |
| Agrobacterium tumefaciens str. C58 plasmid At | NC_003064 | no | 58 | 484 | 0.107 |
| Ochrobactrum anthropi ATCC 49188 chromosome 2 | NC_009668 | yes | 181 | 1512 | 0.106 |
| Yersinia pseudotuberculosis YPIII | NC_010465 | yes | 448 | 3744 | 0.106 |
| Desulfotalea psychrophila LSv54 | NC_006138 | yes | 333 | 2783 | 0.106 |
| Burkholderia vietnamiensis G4 plasmid pBVIE01 | NC_009230 | no | 43 | 360 | 0.106 |
| Methylobacterium radiotolerans JCM 2831 plasmid pMRAD01 | NC_010510 | no | 54 | 453 | 0.106 |
| Buchnera aphidicola str. Cc (Cinara cedri) | NC_008513 | yes | 38 | 319 | 0.106 |
| Rhizobium leguminosarum bv. viciae 3841 plasmid pRL12 | NC_008378 | no | 83 | 697 | 0.106 |
| Brucella ovis ATCC 25840 chromosome II | NC_009504 | yes | 102 | 860 | 0.106 |
| Rhodobacter sphaeroides ATCC 17025 plasmid pRSPA01 | NC_009429 | no | 85 | 717 | 0.105 |
| Baumannia cicadellinicola str. Hc (Homalodisca coagulata) | NC_007984 | no | 63 | 532 | 0.105 |
| Rhizobium leguminosarum bv. viciae 3841 plasmid pRL11 | NC_008384 | no | 67 | 567 | 0.105 |
| Klebsiella pneumoniae subsp. pneumoniae MGH 78578 plasmid pKPN3 | NC_009649 | no | 19 | 161 | 0.105 |
| Shigella dysenteriae Sd197 | NC_007606 | no | 450 | 3821 | 0.105 |
| Bradyrhizobium sp. BTAi1 | NC_009485 | yes | 779 | 6615 | 0.105 |
| Rhizobium leguminosarum bv. viciae 3841 | NC_008380 | yes | 494 | 4200 | 0.105 |
| Rhizobium etli CIAT 652 plasmid pA | NC_010998 | no | 40 | 341 | 0.104 |
| Rickettsia conorii str. Malish 7 | NC_003103 | no | 144 | 1230 | 0.104 |
| Pseudomonas syringae pv. syringae B728a | NC_007005 | yes | 533 | 4556 | 0.104 |
| Yersinia pestis Pestoides F | NC_009381 | yes | 403 | 3447 | 0.104 |
| Wolbachia endosymbiont of Drosophila melanogaster | NC_002978 | no | 125 | 1070 | 0.104 |
| Brucella suis ATCC 23445 chromosome II | NC_010167 | yes | 138 | 1182 | 0.104 |
| Shewanella baltica OS195 plasmid pS19501 | NC_009998 | no | 7 | 60 | 0.104 |
| Salmonella enterica subsp. enterica serovar Paratyphi B str. SPB7 | NC_010102 | yes | 584 | 5008 | 0.104 |
| Yersinia pestis Antiqua | NC_008150 | yes | 435 | 3732 | 0.104 |
| Brucella ovis ATCC 25840 chromosome I | NC_009505 | no | 201 | 1727 | 0.104 |
| Beijerinckia indica subsp. indica ATCC 9039 | NC_010581 | yes | 372 | 3197 | 0.104 |
| Bordetella parapertussis 12822 | NC_002928 | yes | 436 | 3749 | 0.104 |
| Aeromonas salmonicida subsp. salmonicida A449 plasmid pAsa4 | NC_009349 | no | 18 | 155 | 0.104 |
| Pseudomonas syringae pv. phaseolicola 1448A | NC_005773 | yes | 518 | 4466 | 0.103 |
| Vibrio parahaemolyticus RIMD 2210633 chromosome II | NC_004605 | yes | 182 | 1570 | 0.103 |
| Nitrosospira multiformis ATCC 25196 chromosome 1 | NC_007614 | yes | 286 | 2471 | 0.103 |
| Sphingopyxis alaskensis RB2256 | NC_008048 | yes | 328 | 2837 | 0.103 |
| Polynucleobacter sp. QLW-P1DMWA-1 | NC_009379 | no | 215 | 1862 | 0.103 |
| Gluconobacter oxydans 621H plasmid pGOX2 | NC_006673 | no | 3 | 26 | 0.103 |
| Photobacterium profundum SS9 chromosome 1 | NC_006370 | yes | 353 | 3063 | 0.103 |
| Polaromonas naphthalenivorans CJ2 plasmid pPNAP03 | NC_008759 | no | 16 | 139 | 0.103 |
| Photobacterium profundum SS9 chromosome 2 | NC_006371 | no | 207 | 1799 | 0.103 |
| Azoarcus sp. EbN1 plasmid 2 | NC_006824 | no | 20 | 174 | 0.103 |
| Yersinia pestis Nepal516 | NC_008149 | yes | 410 | 3571 | 0.102 |
| Polaromonas naphthalenivorans CJ2 plasmid pPNAP04 | NC_008760 | no | 14 | 122 | 0.102 |
| Rhodopseudomonas palustris TIE-1 | NC_011004 | yes | 540 | 4706 | 0.102 |
| Buchnera aphidicola str. APS (Acyrthosiphon pisum) | NC_002528 | yes | 58 | 506 | 0.102 |
| Bartonella henselae str. Houston-1 | NC_005956 | no | 153 | 1335 | 0.102 |
| Photorhabdus luminescens subsp. laumondii TTO1 | NC_005126 | yes | 481 | 4202 | 0.102 |
| Methylobacterium radiotolerans JCM 2831 | NC_010505 | yes | 584 | 5102 | 0.102 |
| Brucella abortus S19 chromosome 2 | NC_010740 | yes | 106 | 927 | 0.102 |
| Beijerinckia indica subsp. indica ATCC 9039 plasmid pBIND02 | NC_010578 | no | 4 | 35 | 0.102 |
| Verminephrobacter eiseniae EF01-2 plasmid pVEIS01 | NC_008771 | no | 4 | 35 | 0.102 |
| Burkholderia cenocepacia HI2424 plasmid 1 | NC_008545 | no | 16 | 140 | 0.102 |
| Sinorhizobium meliloti 1021 plasmid pSymB | NC_003078 | no | 161 | 1409 | 0.102 |
| Rhizobium etli CFN 42 plasmid p42f | NC_007766 | no | 58 | 509 | 0.102 |
| Burkholderia ambifaria MC40-6 plasmid pBMC401 | NC_010553 | no | 28 | 246 | 0.102 |
| Dinoroseobacter shibae DFL 12 plasmid pDSHI02 | NC_009956 | no | 14 | 123 | 0.102 |
| Nitrosomonas eutropha C91 | NC_008344 | yes | 249 | 2195 | 0.101 |
| Brucella melitensis biovar Abortus 2308 chromosome II | NC_007624 | yes | 105 | 929 | 0.101 |
| Erwinia carotovora subsp. atroseptica SCRI1043 | NC_004547 | yes | 454 | 4018 | 0.101 |
| Wigglesworthia glossinidia endosymbiont of Glossina brevipalpis | NC_004344 | yes | 62 | 549 | 0.101 |
| Yersinia pseudotuberculosis IP 32953 | NC_006155 | yes | 395 | 3506 | 0.101 |
| Anaeromyxobacter sp. Fw109-5 | NC_009675 | yes | 452 | 4014 | 0.101 |
| Brucella suis 1330 chromosome II | NC_004311 | yes | 116 | 1032 | 0.101 |
| Syntrophus aciditrophicus SB | NC_007759 | yes | 320 | 2848 | 0.101 |
| Pseudomonas fluorescens Pf-5 | NC_004129 | yes | 620 | 5518 | 0.101 |
| Methylobacterium sp. 4-46 | NC_010511 | yes | 667 | 5942 | 0.100 |
| Shewanella pealeana ATCC 700345 | NC_009901 | yes | 428 | 3813 | 0.100 |
| Rhizobium etli CFN 42 | NC_007761 | yes | 407 | 3628 | 0.100 |
| Sinorhizobium meliloti 1021 plasmid pSymA | NC_003037 | no | 130 | 1160 | 0.100 |
| Sinorhizobium medicae WSM419 plasmid pSMED03 | NC_009622 | no | 15 | 134 | 0.100 |
| Brucella abortus biovar 1 str. 9-941 chromosome II | NC_006933 | yes | 106 | 949 | 0.100 |
| Candidatus Pelagibacter ubique HTCC1062 | NC_007205 | no | 136 | 1218 | 0.100 |
| Candidatus Ruthia magnifica str. Cm (Calyptogena magnifica) | NC_008610 | no | 98 | 878 | 0.100 |
| Erwinia tasmaniensis | NC_010694 | yes | 344 | 3083 | 0.100 |
| Shewanella sp. MR-7 | NC_008322 | yes | 402 | 3604 | 0.100 |
| Acinetobacter sp. ADP1 | NC_005966 | no | 333 | 2992 | 0.100 |
| Bordetella bronchiseptica RB50 | NC_002927 | yes | 500 | 4494 | 0.100 |
| Shigella boydii CDC 3083-94 plasmid pBS512_7 | NC_010672 | no | 1 | 9 | 0.1 |
| Pseudomonas syringae pv. tomato str. DC3000 | NC_004578 | yes | 547 | 4928 | 0.099 |
| Rhodobacter sphaeroides ATCC 17029 chromosome 2 | NC_009050 | no | 105 | 947 | 0.099 |
| Silicibacter sp. TM1040 | NC_008044 | yes | 302 | 2728 | 0.099 |
| Polaromonas naphthalenivorans CJ2 plasmid pPNAP02 | NC_008758 | no | 16 | 145 | 0.099 |
| Roseobacter denitrificans OCh 114 | NC_008209 | yes | 392 | 3554 | 0.099 |
| Shewanella frigidimarina NCIMB 400 | NC_008345 | yes | 400 | 3629 | 0.099 |
| Pelobacter propionicus DSM 2379 | NC_008609 | yes | 355 | 3221 | 0.099 |
| Shewanella sp. MR-4 | NC_008321 | yes | 389 | 3535 | 0.099 |
| Caulobacter sp. K31 plasmid pCAUL01 | NC_010335 | no | 21 | 191 | 0.099 |
| Pseudoalteromonas haloplanktis TAC125 chromosome I | NC_007481 | yes | 290 | 2650 | 0.098 |
| Pseudomonas putida KT2440 | NC_002947 | yes | 527 | 4823 | 0.098 |
| Jannaschia sp. CCS1 | NC_007802 | yes | 414 | 3798 | 0.098 |
| Shewanella sp. W3-18-1 | NC_008750 | yes | 397 | 3647 | 0.098 |
| Silicibacter pomeroyi DSS-3 | NC_003911 | yes | 374 | 3436 | 0.098 |
| Shewanella denitrificans OS217 | NC_007954 | yes | 368 | 3386 | 0.098 |
| Escherichia coli CFT073 | NC_004431 | yes | 521 | 4818 | 0.097 |
| Paracoccus denitrificans PD1222 chromosome 1 | NC_008686 | yes | 273 | 2526 | 0.097 |
| Novosphingobium aromaticivorans DSM 12444 | NC_007794 | no | 324 | 3000 | 0.097 |
| Sodalis glossinidius str. 'morsitans' | NC_007712 | yes | 237 | 2195 | 0.097 |
| Rhizobium etli CIAT 652 plasmid pC | NC_010997 | no | 95 | 881 | 0.097 |
| Shewanella halifaxensis HAW-EB4 | NC_010334 | yes | 416 | 3862 | 0.097 |
| Shewanella sediminis HAW-EB3 | NC_009831 | yes | 437 | 4060 | 0.097 |
| Vibrio cholerae O1 biovar eltor str. N16961 chromosome II | NC_002506 | no | 106 | 987 | 0.096 |
| Sinorhizobium medicae WSM419 plasmid pSMED02 | NC_009621 | no | 106 | 988 | 0.096 |
| Yersinia pestis CO92 | NC_003143 | yes | 376 | 3509 | 0.096 |
| Methylobacterium radiotolerans JCM 2831 plasmid pMRAD05 | NC_010518 | no | 3 | 28 | 0.096 |
| Syntrophobacter fumaroxidans MPOB | NC_008554 | no | 393 | 3671 | 0.096 |
| Sinorhizobium medicae WSM419 | NC_009636 | yes | 341 | 3188 | 0.096 |
| Magnetococcus sp. MC-1 | NC_008576 | yes | 359 | 3357 | 0.096 |
| Bartonella tribocorum CIP 105476 | NC_010161 | no | 200 | 1874 | 0.096 |
| Marinomonas sp. MWYL1 | NC_009654 | yes | 428 | 4011 | 0.096 |
| Burkholderia vietnamiensis G4 plasmid pBVIE03 | NC_009229 | no | 24 | 225 | 0.096 |
| Maricaulis maris MCS10 | NC_008347 | yes | 295 | 2768 | 0.096 |
| Shigella boydii CDC 3083-94 plasmid pBS512_33 | NC_010657 | no | 5 | 47 | 0.096 |
| Silicibacter sp. TM1040 mega plasmid | NC_008043 | no | 70 | 658 | 0.096 |
| Pseudomonas fluorescens PfO-1 | NC_007492 | yes | 551 | 5185 | 0.096 |
| Shewanella baltica OS195 | NC_009997 | yes | 432 | 4067 | 0.096 |
| Enterobacter sp. 638 plasmid pENTE01 | NC_009425 | no | 12 | 113 | 0.096 |
| Aeromonas hydrophila subsp. hydrophila ATCC 7966 | NC_008570 | yes | 395 | 3727 | 0.095 |
| Alcanivorax borkumensis SK2 | NC_008260 | no | 264 | 2491 | 0.095 |
| Rhodobacter sphaeroides 2.4.1 chromosome 2 | NC_007494 | no | 80 | 755 | 0.095 |
| Brucella suis ATCC 23445 chromosome I | NC_010169 | no | 184 | 1737 | 0.095 |
| Chromobacterium violaceum ATCC 12472 | NC_005085 | yes | 422 | 3985 | 0.095 |
| Shewanella putrefaciens CN-32 | NC_009438 | yes | 380 | 3592 | 0.095 |
| Brucella canis ATCC 23365 chromosome I | NC_010103 | no | 201 | 1901 | 0.095 |
| Rickettsia akari str. Hartford | NC_009881 | no | 120 | 1139 | 0.095 |
| Buchnera aphidicola str. Sg (Schizaphis graminum) | NC_004061 | yes | 52 | 494 | 0.095 |
| Halorhodospira halophila SL1 | NC_008789 | yes | 229 | 2178 | 0.095 |
| Vibrio vulnificus YJ016 chromosome II | NC_005140 | no | 161 | 1535 | 0.094 |
| Shewanella sp. ANA-3 chromosome 1 | NC_008577 | yes | 390 | 3721 | 0.094 |
| Agrobacterium tumefaciens str. C58 chromosome circular | NC_003062 | yes | 262 | 2503 | 0.094 |
| Escherichia coli O157:H7 EDL933 | NC_002655 | yes | 503 | 4809 | 0.094 |
| Escherichia coli UTI89 | NC_007946 | yes | 475 | 4546 | 0.094 |
| Methylobacillus flagellatus KT | NC_007947 | yes | 260 | 2493 | 0.094 |
| Neisseria gonorrhoeae FA 1090 | NC_002946 | no | 189 | 1813 | 0.094 |
| Sinorhizobium medicae WSM419 plasmid pSMED01 | NC_009620 | no | 136 | 1305 | 0.094 |
| Citrobacter koseri ATCC BAA-895 | NC_009792 | yes | 470 | 4510 | 0.094 |
| Acinetobacter baumannii ACICU | NC_010611 | no | 346 | 3321 | 0.094 |
| Candidatus Blochmannia floridanus | NC_005061 | no | 55 | 528 | 0.094 |
| Nitrosomonas europaea ATCC 19718 | NC_004757 | yes | 231 | 2230 | 0.093 |
| Bordetella avium 197N | NC_010645 | yes | 317 | 3064 | 0.093 |
| Aeromonas salmonicida subsp. salmonicida A449 | NC_009348 | yes | 383 | 3703 | 0.093 |
| Salmonella enterica subsp. enterica serovar Choleraesuis str. SC-B67 | NC_006905 | yes | 413 | 4000 | 0.093 |
| Shewanella baltica OS185 | NC_009665 | yes | 404 | 3919 | 0.093 |
| Shewanella baltica OS155 | NC_009052 | yes | 401 | 3906 | 0.093 |
| Erythrobacter litoralis HTCC2594 | NC_007722 | no | 280 | 2731 | 0.092 |
| Acinetobacter baumannii ATCC 17978 | NC_009085 | no | 311 | 3041 | 0.092 |
| Vibrio fischeri ES114 chromosome II | NC_006841 | no | 109 | 1066 | 0.092 |
| Escherichia coli APEC O1 | NC_008563 | yes | 410 | 4018 | 0.092 |
| Bordetella pertussis Tohama I | NC_002929 | yes | 318 | 3118 | 0.092 |
| Escherichia coli O157:H7 str. Sakai | NC_002695 | yes | 484 | 4746 | 0.092 |
| Vibrio fischeri ES114 chromosome I | NC_006840 | yes | 239 | 2347 | 0.092 |
| Shewanella woodyi ATCC 51908 | NC_010506 | yes | 451 | 4429 | 0.092 |
| Rickettsia canadensis str. McKiel | NC_009879 | no | 101 | 992 | 0.092 |
| Vibrio vulnificus YJ016 chromosome I | NC_005139 | yes | 301 | 2958 | 0.092 |
| Pelobacter propionicus DSM 2379 plasmid pPRO1 | NC_008607 | no | 18 | 177 | 0.092 |
| Marinobacter aquaeolei VT8 | NC_008740 | yes | 356 | 3502 | 0.092 |
| Dinoroseobacter shibae DFL 12 | NC_009952 | yes | 329 | 3248 | 0.091 |
| Rhodobacter sphaeroides 2.4.1 plasmid A, partial sequence | NC_009007 | no | 8 | 79 | 0.091 |
| Sinorhizobium meliloti 1021 | NC_003047 | yes | 307 | 3034 | 0.091 |
| Rhizobium leguminosarum bv. viciae 3841 plasmid pRL9 | NC_008379 | no | 28 | 277 | 0.091 |
| Coxiella burnetii RSA 493 | NC_002971 | no | 184 | 1832 | 0.091 |
| Helicobacter hepaticus ATCC 51449 | NC_004917 | yes | 171 | 1704 | 0.091 |
| Saccharophagus degradans 2-40 | NC_007912 | yes | 365 | 3643 | 0.091 |
| Acinetobacter baumannii plasmid p2ABAYE | NC_010402 | no | 1 | 10 | 0.090 |
| Vibrio cholerae O395 chromosome 1 | NC_009456 | no | 103 | 1030 | 0.090 |
| Polaromonas naphthalenivorans CJ2 plasmid pPNAP07 | NC_008763 | no | 1 | 10 | 0.090 |
| Yersinia pestis Antiqua plasmid pMT | NC_008120 | no | 9 | 90 | 0.090 |
| Coxiella burnetii Dugway 5J108-111 | NC_009727 | no | 187 | 1871 | 0.090 |
| Pseudomonas putida F1 | NC_009512 | yes | 477 | 4775 | 0.090 |
| Shewanella oneidensis MR-1 | NC_004347 | yes | 392 | 3926 | 0.090 |
| Rhizobium etli CFN 42 plasmid p42c | NC_007764 | no | 21 | 211 | 0.090 |
| Alkalilimnicola ehrlichei MLHE-1 | NC_008340 | yes | 259 | 2606 | 0.090 |
| Pseudomonas aeruginosa PA7 | NC_009656 | yes | 568 | 5718 | 0.090 |
| Rickettsia bellii RML369-C | NC_007940 | no | 129 | 1300 | 0.090 |
| Coxiella burnetii RSA 331 | NC_010117 | no | 174 | 1756 | 0.090 |
| Escherichia coli HS | NC_009800 | yes | 394 | 3984 | 0.089 |
| Acinetobacter baumannii AYE | NC_010410 | no | 324 | 3283 | 0.089 |
| Brucella suis 1330 chromosome I | NC_004310 | no | 190 | 1933 | 0.089 |
| Salmonella enterica subsp. arizonae serovar 62:z4,z23:-- | NC_010067 | yes | 402 | 4096 | 0.089 |
| Vibrio cholerae O1 biovar eltor str. N16961 chromosome I | NC_002505 | yes | 245 | 2497 | 0.089 |
| Escherichia coli APEC O1 plasmid pAPEC-O1-R | NC_009838 | no | 20 | 204 | 0.089 |
| Legionella pneumophila str. Lens plasmid pLPL | NC_006366 | no | 5 | 51 | 0.089 |
| Buchnera aphidicola str. Bp (Baizongia pistaciae) | NC_004545 | yes | 45 | 459 | 0.089 |
| Psychromonas ingrahamii 37 | NC_008709 | yes | 316 | 3229 | 0.089 |
| Salmonella enterica subsp. enterica serovar Typhi str. CT18 | NC_003198 | yes | 391 | 4004 | 0.088 |
| Burkholderia cepacia AMMD plasmid 1 | NC_008385 | no | 4 | 41 | 0.088 |
| Azoarcus sp. BH72 | NC_008702 | yes | 354 | 3635 | 0.088 |
| Hahella chejuensis KCTC 2396 | NC_007645 | yes | 601 | 6177 | 0.088 |
| Agrobacterium tumefaciens str. C58 chromosome linear | NC_003063 | no | 164 | 1687 | 0.088 |
| Brucella abortus S19 chromosome 1 | NC_010742 | no | 174 | 1793 | 0.088 |
| Pelobacter carbinolicus DSM 2380 | NC_007498 | yes | 296 | 3056 | 0.088 |
| Vibrio parahaemolyticus RIMD 2210633 chromosome I | NC_004603 | yes | 271 | 2809 | 0.087 |
| Pseudomonas entomophila L48 | NC_008027 | yes | 451 | 4683 | 0.087 |
| Mesorhizobium sp. BNC1 | NC_008254 | yes | 357 | 3707 | 0.087 |
| Campylobacter fetus subsp. fetus 82-40 | NC_008599 | yes | 151 | 1568 | 0.087 |
| Rickettsia massiliae MTU5 | NC_009900 | no | 85 | 883 | 0.087 |
| Vibrio fischeri ES114 plasmid pES100 | NC_006842 | no | 5 | 52 | 0.087 |
| Salmonella enterica subsp. enterica serovar Typhi Ty2 | NC_004631 | yes | 377 | 3941 | 0.087 |
| Pseudomonas putida W619 | NC_010501 | yes | 452 | 4730 | 0.087 |
| Brucella abortus biovar 1 str. 9-941 chromosome I | NC_006932 | no | 177 | 1853 | 0.087 |
| Hyphomonas neptunium ATCC 15444 | NC_008358 | yes | 305 | 3200 | 0.087 |
| Burkholderia multivorans ATCC 17616 plasmid pBMUL01 | NC_010070 | no | 12 | 126 | 0.086 |
| Ochrobactrum anthropi ATCC 49188 plasmid pOANT01 | NC_009669 | no | 14 | 147 | 0.086 |
| Sodalis glossinidius str. 'morsitans' plasmid pSG2 | NC_007714 | no | 2 | 21 | 0.086 |
| Wolbachia endosymbiont strain TRS of Brugia malayi | NC_006833 | no | 70 | 735 | 0.086 |
| Pseudomonas aeruginosa UCBPP-PA14 | NC_008463 | yes | 512 | 5380 | 0.086 |
| Brucella melitensis biovar Abortus 2308 chromosome I | NC_007618 | no | 173 | 1827 | 0.086 |
| Rhodobacter sphaeroides ATCC 17025 | NC_009428 | yes | 268 | 2843 | 0.086 |
| Anaeromyxobacter dehalogenans 2CP-C | NC_007760 | yes | 373 | 3973 | 0.085 |
| Roseobacter denitrificans plasmid pTB1 | NC_008386 | no | 9 | 96 | 0.085 |
| Neisseria meningitidis 053442 | NC_010120 | no | 173 | 1847 | 0.085 |
| Parvibaculum lavamentivorans DS-1 | NC_009719 | yes | 311 | 3325 | 0.085 |
| Salmonella enterica subsp. enterica serovar Paratyphi A str. ATCC 9150 | NC_006511 | yes | 350 | 3743 | 0.085 |
| Rhodobacter sphaeroides 2.4.1 chromosome 1 | NC_007493 | yes | 258 | 2764 | 0.085 |
| Ralstonia metallidurans CH34 plasmid 2 | NC_007972 | no | 14 | 150 | 0.085 |
| Francisella philomiragia subsp. philomiragia ATCC 25017 | NC_010336 | no | 163 | 1748 | 0.085 |
| Yersinia enterocolitica subsp. enterocolitica 8081 | NC_008800 | yes | 339 | 3640 | 0.085 |
| Shewanella baltica OS155 plasmid pSbal02 | NC_009036 | no | 6 | 65 | 0.084 |
| Nitrosococcus oceani ATCC 19707 | NC_007484 | yes | 251 | 2723 | 0.084 |
| Escherichia coli E24377A | NC_009801 | yes | 400 | 4349 | 0.084 |
| Pseudomonas mendocina ymp | NC_009439 | yes | 386 | 4208 | 0.084 |
| Dechloromonas aromatica RCB | NC_007298 | yes | 350 | 3821 | 0.083 |
| Vibrio cholerae O395 chromosome 2 | NC_009457 | yes | 230 | 2512 | 0.083 |
| Neisseria meningitidis MC58 | NC_003112 | no | 173 | 1890 | 0.083 |
| Rhodobacter sphaeroides ATCC 17029 chromosome 1 | NC_009049 | yes | 249 | 2724 | 0.083 |
| Pseudomonas putida GB-1 | NC_010322 | yes | 453 | 4956 | 0.083 |
| Escherichia coli SMS-3-5 | NC_010498 | yes | 397 | 4346 | 0.083 |
| Rickettsia felis URRWXCal2 | NC_007109 | no | 117 | 1283 | 0.083 |
| Neisseria gonorrhoeae NCCP11945 plasmid pNGK | NC_011034 | no | 1 | 11 | 0.083 |
| Acinetobacter baumannii plasmid p3ABSDF | NC_010398 | no | 2 | 22 | 0.083 |
| Rhodobacter sphaeroides ATCC 17025 plasmid pRSPA05 | NC_009433 | no | 1 | 11 | 0.083 |
| Shewanella baltica OS155 plasmid pSbal03 | NC_009037 | no | 1 | 11 | 0.083 |
| Sphingomonas wittichii RW1 | NC_009511 | yes | 403 | 4447 | 0.083 |
| Salmonella typhimurium LT2 | NC_003197 | yes | 367 | 4058 | 0.082 |
| Paracoccus denitrificans PD1222 plasmid 1 | NC_008688 | no | 51 | 565 | 0.082 |
| Vibrio vulnificus CMCP6 chromosome II | NC_004460 | no | 128 | 1429 | 0.082 |
| Francisella tularensis subsp. tularensis WY96-3418 | NC_009257 | no | 134 | 1500 | 0.082 |
| Pseudomonas aeruginosa PAO1 | NC_002516 | yes | 454 | 5114 | 0.081 |
| Haemophilus influenzae 86-028NP | NC_007146 | no | 146 | 1646 | 0.081 |
| Campylobacter jejuni subsp. doylei 269.97 | NC_009707 | yes | 141 | 1590 | 0.081 |
| Enterobacter sp. 638 | NC_009436 | yes | 335 | 3780 | 0.081 |
| Caulobacter sp. K31 | NC_010338 | yes | 412 | 4649 | 0.081 |
| Ochrobactrum anthropi ATCC 49188 plasmid pOANT03 | NC_009671 | no | 7 | 79 | 0.081 |
| Thiobacillus denitrificans ATCC 25259 | NC_007404 | yes | 230 | 2597 | 0.081 |
| Vibrio vulnificus CMCP6 chromosome I | NC_004459 | yes | 238 | 2689 | 0.081 |
| Escherichia coli 536 | NC_008253 | yes | 375 | 4245 | 0.081 |
| Ochrobactrum anthropi ATCC 49188 plasmid pOANT04 | NC_009672 | no | 3 | 34 | 0.081 |
| Haemophilus somnus 2336 | NC_010519 | no | 160 | 1820 | 0.080 |
| Shewanella oneidensis MR-1 plasmid pMR-1 | NC_004349 | no | 12 | 137 | 0.080 |
| Rhodobacter sphaeroides 2.4.1 plasmid D | NC_007490 | no | 7 | 80 | 0.080 |
| Haemophilus somnus 129PT | NC_008309 | no | 144 | 1648 | 0.080 |
| Escherichia coli ATCC 8739 | NC_010468 | yes | 336 | 3864 | 0.08 |
| Rhodospirillum rubrum ATCC 11170 plasmid unnamed | NC_007641 | no | 4 | 46 | 0.08 |
| Rickettsia typhi str. Wilmington | NC_006142 | no | 67 | 771 | 0.079 |
| Ochrobactrum anthropi ATCC 49188 chromosome 1 | NC_009667 | no | 218 | 2513 | 0.079 |
| Rhizobium etli CFN 42 plasmid p42b | NC_007763 | no | 13 | 150 | 0.079 |
| Haemophilus influenzae Rd KW20 | NC_000907 | no | 132 | 1525 | 0.079 |
| Mesorhizobium sp. BNC1 plasmid 2 | NC_008243 | no | 9 | 104 | 0.079 |
| Neisseria meningitidis Z2491 | NC_003116 | no | 163 | 1886 | 0.079 |
| Ralstonia eutropha JMP134 plasmid 1 | NC_007337 | no | 7 | 81 | 0.079 |
| Campylobacter jejuni subsp. jejuni 81-176 | NC_008787 | yes | 131 | 1522 | 0.079 |
| Desulfotalea psychrophila LSv54 plasmid large | NC_006139 | no | 8 | 93 | 0.079 |
| Serratia proteamaculans 568 | NC_009832 | yes | 387 | 4504 | 0.079 |
| Enterobacter sakazakii ATCC BAA-894 plasmid pESA2 | NC_009779 | no | 3 | 35 | 0.078 |
| Legionella pneumophila str. Corby | NC_009494 | yes | 253 | 2953 | 0.078 |
| Geobacter sulfurreducens PCA | NC_002939 | yes | 271 | 3175 | 0.078 |
| Legionella pneumophila str. Paris | NC_006368 | yes | 238 | 2789 | 0.078 |
| Rickettsia bellii OSU 85-389 | NC_009883 | no | 116 | 1360 | 0.078 |
| Legionella pneumophila subsp. pneumophila str. Philadelphia 1 | NC_002942 | yes | 231 | 2711 | 0.078 |
| Shewanella loihica PV-4 | NC_009092 | yes | 303 | 3556 | 0.078 |
| Serratia proteamaculans 568 plasmid pSPRO01 | NC_009829 | no | 4 | 47 | 0.078 |
| Anaplasma phagocytophilum HZ | NC_007797 | no | 99 | 1165 | 0.078 |
| Acidovorax sp. JS42 plasmid pAOVO02 | NC_008766 | no | 5 | 59 | 0.078 |
| Escherichia coli str. K-12 substr. DH10B | NC_010473 | yes | 322 | 3804 | 0.078 |
| Francisella tularensis subsp. tularensis Schu 4 | NC_006570 | no | 125 | 1478 | 0.077 |
| Francisella tularensis subsp. tularensis FSC198 | NC_008245 | no | 125 | 1480 | 0.077 |
| Rickettsia prowazekii str. Madrid E | NC_000963 | no | 65 | 770 | 0.077 |
| Legionella pneumophila str. Lens | NC_006369 | yes | 224 | 2654 | 0.077 |
| Escherichia coli str. K-12 substr. MG1655 | NC_000913 | yes | 320 | 3812 | 0.077 |
| Neisseria meningitidis FAM18 | NC_008767 | no | 148 | 1769 | 0.077 |
| Arcobacter butzleri RM4018 | NC_009850 | yes | 174 | 2085 | 0.077 |
| Francisella tularensis subsp. mediasiatica FSC147 | NC_010677 | no | 108 | 1298 | 0.076 |
| Idiomarina loihiensis L2TR | NC_006512 | yes | 201 | 2427 | 0.076 |
| Salmonella enterica subsp. enterica serovar Choleraesuis str. SC-B67 plasmid pSC138 | NC_006856 | no | 13 | 157 | 0.076 |
| Enterobacter sakazakii ATCC BAA-894 | NC_009778 | yes | 324 | 3931 | 0.076 |
| Rhizobium leguminosarum bv. viciae 3841 plasmid pRL8 | NC_008383 | no | 10 | 122 | 0.075 |
| Shewanella amazonensis SB2B | NC_008700 | yes | 276 | 3369 | 0.075 |
| Campylobacter jejuni RM1221 | NC_003912 | yes | 139 | 1699 | 0.075 |
| Geobacter metallireducens GS-15 | NC_007517 | yes | 265 | 3254 | 0.075 |
| Francisella tularensis subsp. holarctica | NC_007880 | no | 132 | 1622 | 0.075 |
| Dichelobacter nodosus VCS1703A | NC_009446 | no | 96 | 1184 | 0.075 |
| Geobacter lovleyi SZ | NC_010814 | yes | 270 | 3336 | 0.074 |
| Mannheimia succiniciproducens MBEL55E | NC_006300 | no | 178 | 2202 | 0.074 |
| Bdellovibrio bacteriovorus HD100 | NC_005363 | yes | 268 | 3319 | 0.074 |
| Campylobacter jejuni subsp. jejuni NCTC 11168 | NC_002163 | yes | 122 | 1512 | 0.074 |
| Francisella tularensis subsp. holarctica OSU18 | NC_008369 | no | 116 | 1439 | 0.074 |
| Haemophilus influenzae PittGG | NC_009567 | no | 124 | 1543 | 0.074 |
| Methylobacterium radiotolerans JCM 2831 plasmid pMRAD08 | NC_010507 | no | 2 | 25 | 0.074 |
| Dinoroseobacter shibae DFL 12 plasmid pDSHI03 | NC_009957 | no | 10 | 125 | 0.074 |
| Sodalis glossinidius str. 'morsitans' plasmid pSG1 | NC_007713 | no | 4 | 50 | 0.074 |
| Methylobacterium radiotolerans JCM 2831 plasmid pMRAD04 | NC_010517 | no | 3 | 38 | 0.073 |
| Acinetobacter baumannii plasmid p3ABAYE | NC_010404 | no | 6 | 76 | 0.073 |
| Sulfurimonas denitrificans DSM 1251 | NC_007575 | yes | 152 | 1944 | 0.072 |
| Thiomicrospira crunogena XCL-2 | NC_007520 | yes | 159 | 2037 | 0.072 |
| Pasteurella multocida subsp. multocida str. Pm70 | NC_002663 | no | 145 | 1870 | 0.071 |
| Candidatus Desulfococcus oleovorans Hxd3 | NC_009943 | no | 234 | 3031 | 0.071 |
| Haemophilus ducreyi 35000HP | NC_002940 | no | 123 | 1594 | 0.071 |
| Francisella tularensis subsp. holarctica FTNF002-00 | NC_009749 | no | 113 | 1467 | 0.071 |
| Erwinia tasmaniensis plasmid pET35 | NC_010696 | no | 3 | 39 | 0.071 |
| Psychrobacter sp. PRwf-1 plasmid pRWF101 | NC_009516 | no | 1 | 13 | 0.071 |
| Acidiphilium cryptum JF-5 plasmid pACRY05 | NC_009471 | no | 2 | 26 | 0.071 |
| Acidovorax sp. JS42 plasmid pAOVO01 | NC_008765 | no | 6 | 78 | 0.071 |
| Campylobacter jejuni subsp. jejuni 81116 | NC_009839 | yes | 116 | 1510 | 0.071 |
| Haemophilus influenzae PittEE | NC_009566 | no | 115 | 1504 | 0.071 |
| Enterobacter sakazakii ATCC BAA-894 plasmid pESA3 | NC_009780 | no | 9 | 118 | 0.070 |
| Shewanella baltica OS185 plasmid pS18501 | NC_009661 | no | 5 | 66 | 0.070 |
| Actinobacillus pleuropneumoniae serovar 3 str. JL03 | NC_010278 | no | 143 | 1893 | 0.070 |
| Klebsiella pneumoniae subsp. pneumoniae MGH 78578 | NC_009648 | no | 331 | 4445 | 0.069 |
| Helicobacter pylori J99 | NC_000921 | yes | 103 | 1386 | 0.069 |
| Escherichia coli E24377A plasmid pETEC_35 | NC_009787 | no | 2 | 27 | 0.068 |
| Lawsonia intracellularis PHE/MN1-00 plasmid 1 | NC_008012 | no | 2 | 27 | 0.068 |
| Psychrobacter cryohalolentis K5 plasmid 1 | NC_007968 | no | 3 | 41 | 0.068 |
| Helicobacter acinonychis str. Sheeba | NC_008229 | yes | 109 | 1503 | 0.067 |
| Francisella tularensis subsp. novicida U112 | NC_008601 | no | 116 | 1603 | 0.067 |
| Campylobacter concisus 13826 | NC_009802 | yes | 130 | 1799 | 0.067 |
| Geobacter uraniireducens Rf4 | NC_009483 | yes | 293 | 4064 | 0.067 |
| Actinobacillus pleuropneumoniae serovar 7 str. AP76 | NC_010939 | no | 143 | 1988 | 0.067 |
| Nitrosospira multiformis ATCC 25196 plasmid 3 | NC_007617 | no | 1 | 14 | 0.066 |
| Actinobacillus pleuropneumoniae L20 | NC_009053 | no | 134 | 1878 | 0.066 |
| Silicibacter sp. TM1040 plasmid unnamed | NC_008042 | no | 7 | 99 | 0.066 |
| Campylobacter hominis ATCC BAA-381 | NC_009714 | no | 111 | 1571 | 0.065 |
| Erwinia tasmaniensis plasmid pET49 | NC_010697 | no | 4 | 57 | 0.065 |
| Burkholderia vietnamiensis G4 plasmid pBVIE04 | NC_009228 | no | 7 | 100 | 0.065 |
| Rhodobacter sphaeroides ATCC 17025 plasmid pRSPA04 | NC_009432 | no | 2 | 29 | 0.064 |
| Neorickettsia sennetsu str. Miyayama | NC_007798 | no | 60 | 872 | 0.064 |
| Actinobacillus succinogenes 130Z | NC_009655 | no | 133 | 1946 | 0.063 |
| Campylobacter curvus 525.92 | NC_009715 | yes | 121 | 1810 | 0.062 |
| Acidiphilium cryptum JF-5 plasmid pACRY04 | NC_009470 | no | 2 | 30 | 0.062 |
| Roseobacter denitrificans plasmid pTB3 | NC_008388 | no | 1 | 15 | 0.062 |
| Nitrosospira multiformis ATCC 25196 plasmid 2 | NC_007616 | no | 1 | 15 | 0.062 |
| Dinoroseobacter shibae DFL 12 plasmid pDSHI01 | NC_009955 | no | 12 | 183 | 0.061 |
| Wolinella succinogenes DSM 1740 | NC_005090 | yes | 121 | 1921 | 0.059 |
| Helicobacter pylori 26695 | NC_000915 | yes | 93 | 1483 | 0.059 |
| Nitrosospira multiformis ATCC 25196 plasmid 1 | NC_007615 | no | 1 | 16 | 0.058 |
| Desulfotalea psychrophila LSv54 plasmid small | NC_006140 | no | 1 | 16 | 0.058 |
| Novosphingobium aromaticivorans DSM 12444 plasmid pNL2 | NC_009427 | no | 25 | 406 | 0.058 |
| Polaromonas naphthalenivorans CJ2 plasmid pPNAP05 | NC_008761 | no | 3 | 49 | 0.057 |
| Helicobacter pylori HPAG1 | NC_008086 | yes | 87 | 1449 | 0.056 |
| Gluconobacter oxydans 621H plasmid pGOX4 | NC_006675 | no | 1 | 17 | 0.055 |
| Polaromonas naphthalenivorans CJ2 plasmid pPNAP06 | NC_008762 | no | 1 | 19 | 0.05 |
| Sulfurovum sp. NBC37-1 | NC_009663 | no | 121 | 2317 | 0.049 |
| Methylobacterium sp. 4-46 plasmid pM44602 | NC_010374 | no | 1 | 20 | 0.047 |
| Nitrosococcus oceani ATCC 19707 plasmid A | NC_007483 | no | 2 | 41 | 0.046 |
| Nitratiruptor sp. SB155-2 | NC_009662 | yes | 82 | 1761 | 0.044 |
| Legionella pneumophila str. Paris plasmid pLPP | NC_006365 | no | 6 | 133 | 0.043 |
| Campylobacter jejuni subsp. jejuni 81-176 plasmid pTet | NC_008790 | no | 2 | 50 | 0.038 |
| Yersinia pestis biovar Microtus str. 91001 plasmid pCRY | NC_005814 | no | 1 | 29 | 0.033 |
| Pelobacter propionicus DSM 2379 plasmid pPRO2 | NC_008608 | no | 1 | 32 | 0.030 |
| Campylobacter jejuni subsp. jejuni 81-176 plasmid pVir | NC_008770 | no | 1 | 52 | 0.018 |
| Burkholderia cenocepacia chromosome 3 | NC_011002 | no | 0 | 1 | 0.0 |
| Actinobacillus pleuropneumoniae serovar 7 str. AP76 plasmid ABB7_B | NC_010941 | no | 0 | 4 | 0.0 |
| Actinobacillus pleuropneumoniae serovar 7 str. AP76 plasmid APP7_C | NC_010940 | no | 0 | 2 | 0.0 |
| Erwinia tasmaniensis plasmid pET09 | NC_010695 | no | 0 | 7 | 0.0 |
| Shigella boydii CDC 3083-94 plasmid pBS512_5 | NC_010659 | no | 0 | 4 | 0.0 |
| Shigella boydii CDC 3083-94 plasmid pBS512_2 | NC_010656 | no | 0 | 3 | 0.0 |
| Methylobacterium radiotolerans JCM 2831 plasmid pMRAD06 | NC_010502 | no | 0 | 23 | 0.0 |
| Escherichia coli SMS-3-5 plasmid pSMS35_3 | NC_010487 | no | 0 | 3 | 0.0 |
| Acinetobacter baumannii plasmid p4ABAYE | NC_010403 | no | 0 | 5 | 0.0 |
| Acinetobacter baumannii plasmid p1ABAYE | NC_010401 | no | 0 | 7 | 0.0 |
| Citrobacter koseri ATCC BAA-895 plasmid pCKO2 | NC_009794 | no | 0 | 13 | 0.0 |
| Campylobacter hominis ATCC BAA-381 plasmid pCH4 | NC_009713 | no | 0 | 5 | 0.0 |
| Klebsiella pneumoniae subsp. pneumoniae MGH 78578 plasmid pKPN7 | NC_009653 | no | 0 | 5 | 0.0 |
| Klebsiella pneumoniae subsp. pneumoniae MGH 78578 plasmid pKPN6 | NC_009652 | no | 0 | 5 | 0.0 |
| Acidiphilium cryptum JF-5 plasmid pACRY08 | NC_009474 | no | 0 | 5 | 0.0 |
| Acidiphilium cryptum JF-5 plasmid pACRY07 | NC_009473 | no | 0 | 5 | 0.0 |
| Shigella sonnei Ss046 plasmid pSS046_spC | NC_009347 | no | 0 | 1 | 0.0 |
| Acinetobacter baumannii ATCC 17978 plasmid pAB2 | NC_009084 | no | 0 | 5 | 0.0 |
| Acinetobacter baumannii ATCC 17978 plasmid pAB1 | NC_009083 | no | 0 | 11 | 0.0 |
| Shewanella baltica OS155 plasmid pSbal04 | NC_009038 | no | 0 | 7 | 0.0 |
| Polaromonas naphthalenivorans CJ2 plasmid pPNAP08 | NC_008764 | no | 0 | 5 | 0.0 |
| Shewanella sp. MR-7 plasmid1 | NC_008320 | no | 0 | 8 | 0.0 |
| Helicobacter acinonychis str. Sheeba plasmid pHac1 | NC_008230 | no | 0 | 6 | 0.0 |
| Helicobacter pylori HPAG1 plasmid pHPAG1 | NC_008087 | no | 0 | 8 | 0.0 |
| Geobacter metallireducens GS-15 plasmid unnamed | NC_007515 | no | 0 | 13 | 0.0 |
| Aeromonas salmonicida salmonicida A449 plasmid pAsa2 | NC_004925 | no | 0 | 7 | 0.0 |
| Aeromonas salmonicida salmonicida A449 plasmid pAsa3 | NC_004924 | no | 0 | 9 | 0.0 |
| Aeromonas salmonicida salmonicida A449 plasmid pAsa1 | NC_004923 | no | 0 | 8 | 0.0 |
| Xylella fastidiosa Temecula1 plasmid pXFPD1.3 | NC_004554 | no | 0 | 2 | 0.0 |
| Wigglesworthia glossinidia endosymbiont of Glossina brevipalpis plasmid pWb1 | NC_003425 | no | 0 | 6 | 0.0 |
