# Supplementary material for: Prediction of Type III Secretion Signals in Genomes of Gram-Negative Bacteria
Source: PLoS One. 2009 Jun 15;4(6):e5917. doi: 10.1371/journal.pone.0005917 (PMC2690842; doi:10.1371/journal.pone.0005917)
Supplement: Table S2 — Complete list of examined protein sequence sets of Firmicutes. Given is the genome name, the NCBI Refseq database identification string, the existence of an YscN homologue, the number of positive predictions (P), the number of negative predictions (N) and the relative number of positively predicted protein sequences (%). The list is sorted according to decreasing fractions of predicted proteins. (0.20 MB DOC) [file pone.0005917.s006.doc]

**Table S2.** Complete list of examined protein sequence sets of *Firmicutes*. Given is the genome name, the NCBI Refseq database identification string, the existence of an *YscN* homologue, the number of positive predictions (*P*), the number of negative predictions (*N*) and the relative number of positively predicted protein sequences (%). The list is sorted according to decreasing fractions of predicted proteins.

| **Name** | **NCBI ID** | ***YscN*** | ***P*** | ***N*** | **%** |
| --- | --- | --- | --- | --- | --- |
| Exiguobacterium sibiricum 255-15 plasmid pEXIG02 | NC_010550 | no | 1 | 2 | 0.333 |
| Staphylococcus aureus subsp. aureus USA300 plasmid pUSA02 | NC_007791 | no | 1 | 2 | 0.333 |
| Staphylococcus aureus subsp. aureus COL plasmid pT181 | NC_006629 | no | 1 | 2 | 0.333 |
| Lactobacillus plantarum WCFS1 plasmid pWCFS101 | NC_006375 | no | 1 | 2 | 0.333 |
| Staphylococcus epidermidis ATCC 12228 plasmid pSE-12228-01 | NC_005008 | no | 1 | 2 | 0.333 |
| Staphylococcus epidermidis ATCC 12228 plasmid pSE-12228-06 | NC_005003 | no | 3 | 8 | 0.272 |
| Lactobacillus plantarum WCFS1 plasmid pWCFS102 | NC_006376 | no | 1 | 3 | 0.25 |
| Natranaerobius thermophilus JW/NM-WN-LF plasmid pNTHE02 | NC_010724 | no | 2 | 8 | 0.2 |
| Lactococcus lactis subsp. cremoris SK11 plasmid 1 | NC_008503 | no | 2 | 8 | 0.2 |
| Clostridium perfringens SM101 plasmid 2 | NC_008264 | no | 2 | 8 | 0.2 |
| Staphylococcus aureus subsp. aureus USA300 plasmid pUSA01 | NC_007790 | no | 1 | 4 | 0.2 |
| Bacillus cereus E33L plasmid pE33L9 | NC_007107 | no | 2 | 8 | 0.2 |
| Staphylococcus epidermidis ATCC 12228 plasmid pSE-12228-02 | NC_005007 | no | 1 | 5 | 0.166 |
| Bacillus weihenstephanensis KBAB4 plasmid pBWB402 | NC_010181 | no | 9 | 54 | 0.142 |
| Staphylococcus saprophyticus subsp. saprophyticus ATCC 15305 plasmid pSSP2 | NC_007352 | no | 3 | 20 | 0.130 |
| Lactococcus lactis subsp. cremoris SK11 plasmid 5 | NC_008507 | no | 1 | 7 | 0.125 |
| Bacillus cereus E33L plasmid pE33L8 | NC_007106 | no | 1 | 7 | 0.125 |
| Geobacillus thermodenitrificans NG80-2 plasmid pLW1071 | NC_009329 | no | 6 | 47 | 0.113 |
| Staphylococcus aureus subsp. aureus JH9 plasmid pSJH901 | NC_009477 | no | 3 | 26 | 0.103 |
| Clostridium perfringens SM101 plasmid 1 | NC_008263 | no | 1 | 9 | 0.1 |
| Lactobacillus salivarius UCC118 plasmid pSF118-44 | NC_006530 | no | 5 | 46 | 0.098 |
| Enterococcus faecalis V583 plasmid pTEF2 | NC_004671 | no | 6 | 56 | 0.096 |
| Geobacillus kaustophilus HTA426 plasmid pHTA426 | NC_006509 | no | 4 | 38 | 0.095 |
| Staphylococcus epidermidis ATCC 12228 | NC_004461 | no | 224 | 2195 | 0.092 |
| Clostridium botulinum A3 str. Loch Maree plasmid pCLK | NC_010418 | no | 30 | 299 | 0.091 |
| Staphylococcus aureus subsp. aureus JH1 plasmid pSJH101 | NC_009619 | no | 3 | 30 | 0.090 |
| Clostridium difficile 630 plasmid pCD630 | NC_008226 | no | 1 | 10 | 0.090 |
| Bacillus thuringiensis serovar konkukian str. 97-27 plasmid pBT9727 | NC_006578 | no | 7 | 73 | 0.087 |
| Symbiobacterium thermophilum IAM 14863 | NC_006177 | yes | 288 | 3050 | 0.086 |
| Leuconostoc citreum KM20 plasmid pLCK2 | NC_010466 | no | 3 | 33 | 0.083 |
| Bacillus anthracis str. 'Ames Ancestor' plasmid pXO1 | NC_007322 | no | 17 | 187 | 0.083 |
| Bacillus cereus ATCC 10987 plasmid pBc10987 | NC_005707 | no | 20 | 221 | 0.082 |
| Staphylococcus epidermidis RP62A | NC_002976 | no | 205 | 2289 | 0.082 |
| Staphylococcus aureus subsp. aureus NCTC 8325 | NC_007795 | no | 237 | 2655 | 0.081 |
| Leuconostoc citreum KM20 plasmid pLCK1 | NC_010470 | no | 4 | 45 | 0.081 |
| Heliobacterium modesticaldum Ice1 | NC_010337 | yes | 236 | 2764 | 0.078 |
| Leuconostoc citreum KM20 plasmid pLCK4 | NC_010469 | no | 1 | 12 | 0.076 |
| Staphylococcus aureus subsp. aureus USA300_TCH1516 plasmid pUSA300HOUMR | NC_010063 | no | 2 | 24 | 0.076 |
| Natranaerobius thermophilus JW/NM-WN-LF | NC_010718 | yes | 218 | 2664 | 0.075 |
| Staphylococcus aureus subsp. aureus USA300 | NC_007793 | no | 191 | 2369 | 0.074 |
| Oenococcus oeni PSU-1 | NC_008528 | no | 126 | 1565 | 0.074 |
| Clostridium botulinum B str. Eklund 17B plasmid pCLL | NC_010680 | no | 4 | 50 | 0.074 |
| Clostridium novyi NT | NC_008593 | yes | 171 | 2144 | 0.073 |
| Clostridium acetobutylicum ATCC 824 plasmid pSOL1 | NC_001988 | no | 13 | 163 | 0.073 |
| Leuconostoc citreum KM20 | NC_010471 | no | 125 | 1577 | 0.073 |
| Clostridium botulinum B str. Eklund 17B | NC_010674 | yes | 255 | 3218 | 0.073 |
| Lactobacillus plantarum WCFS1 | NC_004567 | no | 219 | 2788 | 0.072 |
| Staphylococcus saprophyticus subsp. saprophyticus ATCC 15305 | NC_007350 | no | 178 | 2268 | 0.072 |
| Staphylococcus haemolyticus JCSC1435 | NC_007168 | no | 194 | 2482 | 0.072 |
| Clostridium botulinum E3 str. Alaska E43 | NC_010723 | yes | 233 | 3023 | 0.071 |
| Lactobacillus helveticus DPC 4571 | NC_010080 | no | 114 | 1496 | 0.070 |
| Clostridium kluyveri DSM 555 | NC_009706 | yes | 271 | 3567 | 0.070 |
| Lactobacillus plantarum WCFS1 plasmid pWCFS103 | NC_006377 | no | 3 | 40 | 0.069 |
| Staphylococcus aureus subsp. aureus COL | NC_002951 | no | 182 | 2433 | 0.069 |
| Staphylococcus aureus subsp. aureus MW2 | NC_003923 | no | 183 | 2449 | 0.069 |
| Lactobacillus gasseri ATCC 33323 | NC_008530 | no | 122 | 1633 | 0.069 |
| Staphylococcus aureus subsp. aureus USA300_TCH1516 | NC_010079 | no | 183 | 2474 | 0.068 |
| Staphylococcus aureus subsp. aureus str. Newman | NC_009641 | no | 180 | 2434 | 0.068 |
| Lactobacillus reuteri F275 | NC_009513 | no | 130 | 1770 | 0.068 |
| Staphylococcus aureus subsp. aureus MSSA476 | NC_002953 | no | 175 | 2404 | 0.067 |
| Clostridium tetani E88 plasmid pE88 | NC_004565 | no | 4 | 55 | 0.067 |
| Staphylococcus aureus RF122 | NC_007622 | no | 170 | 2339 | 0.067 |
| Bacillus cereus E33L plasmid pE33L466 | NC_007103 | no | 29 | 401 | 0.067 |
| Clostridium perfringens SM101 | NC_008262 | no | 171 | 2387 | 0.066 |
| Clostridium beijerinckii NCIMB 8052 | NC_009617 | yes | 335 | 4685 | 0.066 |
| Staphylococcus aureus subsp. aureus Mu3 | NC_009782 | no | 180 | 2518 | 0.066 |
| Desulfitobacterium hafniense Y51 | NC_007907 | yes | 337 | 4723 | 0.066 |
| Clostridium tetani E88 | NC_004557 | yes | 158 | 2215 | 0.066 |
| Staphylococcus aureus subsp. aureus N315 | NC_002745 | no | 172 | 2416 | 0.066 |
| Staphylococcus aureus subsp. aureus MRSA252 | NC_002952 | no | 176 | 2480 | 0.066 |
| Staphylococcus aureus subsp. aureus Mu50 | NC_002758 | no | 178 | 2519 | 0.065 |
| Clostridium acetobutylicum ATCC 824 | NC_003030 | yes | 242 | 3430 | 0.065 |
| Bacillus weihenstephanensis KBAB4 plasmid pBWB401 | NC_010180 | no | 19 | 270 | 0.065 |
| Staphylococcus aureus subsp. aureus JH9 | NC_009487 | no | 177 | 2520 | 0.065 |
| Lactobacillus brevis ATCC 367 | NC_008497 | no | 143 | 2042 | 0.065 |
| Oceanobacillus iheyensis HTE831 | NC_004193 | yes | 229 | 3271 | 0.065 |
| Streptococcus pyogenes MGAS2096 | NC_008023 | no | 123 | 1775 | 0.064 |
| Staphylococcus aureus subsp. aureus JH1 | NC_009632 | no | 176 | 2571 | 0.064 |
| Lactobacillus fermentum IFO 3956 | NC_010610 | no | 117 | 1726 | 0.063 |
| Clostridium thermocellum ATCC 27405 | NC_009012 | yes | 202 | 2987 | 0.063 |
| Pediococcus pentosaceus ATCC 25745 | NC_008525 | no | 111 | 1644 | 0.063 |
| Clostridium perfringens ATCC 13124 | NC_008261 | no | 181 | 2695 | 0.062 |
| Clostridium perfringens str. 13 | NC_003366 | no | 167 | 2493 | 0.062 |
| Clostridium botulinum A3 str. Loch Maree | NC_010520 | yes | 229 | 3426 | 0.062 |
| Lactobacillus johnsonii NCC 533 | NC_005362 | no | 114 | 1707 | 0.062 |
| Staphylococcus epidermidis RP62A plasmid pSERP | NC_006663 | no | 2 | 30 | 0.062 |
| Streptococcus thermophilus LMG 18311 | NC_006448 | no | 117 | 1772 | 0.061 |
| Clostridium botulinum A str. Hall | NC_009698 | yes | 210 | 3194 | 0.061 |
| Streptococcus pyogenes MGAS10750 | NC_008024 | no | 122 | 1857 | 0.061 |
| Lysinibacillus sphaericus C3-41 | NC_010382 | yes | 281 | 4303 | 0.061 |
| Streptococcus thermophilus CNRZ1066 | NC_006449 | no | 117 | 1798 | 0.061 |
| Clostridium botulinum A str. ATCC 19397 | NC_009697 | yes | 217 | 3335 | 0.061 |
| Bacillus subtilis subsp. subtilis str. 168 | NC_000964 | yes | 250 | 3855 | 0.060 |
| Streptococcus pyogenes MGAS9429 | NC_008021 | no | 114 | 1763 | 0.060 |
| Finegoldia magna ATCC 29328 | NC_010376 | no | 99 | 1532 | 0.060 |
| Clostridium phytofermentans ISDg | NC_010001 | yes | 235 | 3667 | 0.060 |
| Streptococcus pneumoniae CGSP14 | NC_010582 | no | 132 | 2074 | 0.059 |
| Clostridium difficile 630 | NC_009089 | yes | 223 | 3519 | 0.059 |
| Bacillus amyloliquefaciens FZB42 | NC_009725 | yes | 220 | 3473 | 0.059 |
| Desulfotomaculum reducens MI-1 | NC_009253 | yes | 194 | 3082 | 0.059 |
| Streptococcus suis 98HAH33 | NC_009443 | no | 129 | 2056 | 0.059 |
| Staphylococcus aureus subsp. aureus Mu50 plasmid VRSAp | NC_002774 | no | 2 | 32 | 0.058 |
| Clostridium botulinum A str. ATCC 3502 | NC_009495 | yes | 209 | 3363 | 0.058 |
| Streptococcus pyogenes MGAS315 | NC_004070 | no | 109 | 1756 | 0.058 |
| Lactobacillus reuteri F275 | NC_010609 | no | 106 | 1714 | 0.058 |
| Clostridium botulinum F str. Langeland | NC_009699 | yes | 211 | 3424 | 0.058 |
| Streptococcus pyogenes MGAS10270 | NC_008022 | no | 114 | 1872 | 0.057 |
| Streptococcus pyogenes MGAS10394 | NC_006086 | no | 108 | 1778 | 0.057 |
| Streptococcus suis 05ZYH33 | NC_009442 | no | 125 | 2061 | 0.057 |
| Clostridium botulinum B1 str. Okra | NC_010516 | yes | 209 | 3448 | 0.057 |
| Leuconostoc mesenteroides subsp. mesenteroides ATCC 8293 plasmid pLEUM1 | NC_008496 | no | 2 | 33 | 0.057 |
| Leuconostoc mesenteroides subsp. mesenteroides ATCC 8293 | NC_008531 | no | 112 | 1858 | 0.056 |
| Bacillus pumilus SAFR-032 | NC_009848 | yes | 209 | 3472 | 0.056 |
| Alkaliphilus oremlandii OhILAs | NC_009922 | yes | 161 | 2675 | 0.056 |
| Syntrophomonas wolfei subsp. wolfei str. Goettingen | NC_008346 | yes | 142 | 2362 | 0.056 |
| Clostridium botulinum B1 str. Okra plasmid pCLD | NC_010379 | no | 11 | 184 | 0.056 |
| Streptococcus agalactiae NEM316 | NC_004368 | no | 118 | 1976 | 0.056 |
| Streptococcus agalactiae A909 | NC_007432 | no | 112 | 1884 | 0.056 |
| Streptococcus pneumoniae TIGR4 | NC_003028 | no | 118 | 1987 | 0.056 |
| Streptococcus pyogenes MGAS8232 | NC_003485 | no | 103 | 1736 | 0.056 |
| Lactobacillus sakei subsp. sakei 23K | NC_007576 | no | 105 | 1774 | 0.055 |
| Clostridium botulinum A str. ATCC 3502 plasmid pBOT3502 | NC_009496 | no | 1 | 17 | 0.055 |
| Staphylococcus aureus subsp. aureus USA300 plasmid pUSA03 | NC_007792 | no | 2 | 34 | 0.055 |
| Bacillus cereus E33L plasmid pE33L54 | NC_007105 | no | 3 | 51 | 0.055 |
| Bacillus cereus ATCC 14579 | NC_004722 | yes | 290 | 4944 | 0.055 |
| Lactobacillus casei ATCC 334 | NC_008526 | no | 151 | 2600 | 0.054 |
| Streptococcus pneumoniae G54 | NC_011072 | no | 116 | 1999 | 0.054 |
| Moorella thermoacetica ATCC 39073 | NC_007644 | yes | 135 | 2330 | 0.054 |
| Bacillus weihenstephanensis KBAB4 | NC_010184 | yes | 282 | 4873 | 0.054 |
| Streptococcus agalactiae 2603V/R | NC_004116 | no | 116 | 2008 | 0.054 |
| Streptococcus mutans UA159 | NC_004350 | no | 107 | 1853 | 0.054 |
| Streptococcus pyogenes str. Manfredo | NC_009332 | no | 95 | 1650 | 0.054 |
| Streptococcus pyogenes MGAS6180 | NC_007296 | no | 103 | 1791 | 0.054 |
| Alkaliphilus metalliredigens QYMF | NC_009633 | yes | 251 | 4374 | 0.054 |
| Lactobacillus acidophilus NCFM | NC_006814 | no | 101 | 1761 | 0.054 |
| Streptococcus pyogenes MGAS5005 | NC_007297 | no | 101 | 1764 | 0.054 |
| Bacillus licheniformis ATCC 14580 | NC_006322 | yes | 227 | 3969 | 0.054 |
| Lactobacillus salivarius UCC118 plasmid pMP118 | NC_007930 | no | 12 | 210 | 0.054 |
| Streptococcus pyogenes SSI-1 | NC_004606 | no | 100 | 1761 | 0.053 |
| Lysinibacillus sphaericus C3-41 plasmid pBsph | NC_010381 | no | 10 | 177 | 0.053 |
| Clostridium kluyveri DSM 555 plasmid pCKL555A | NC_009466 | no | 4 | 71 | 0.053 |
| Streptococcus thermophilus LMD-9 | NC_008532 | no | 91 | 1619 | 0.053 |
| Streptococcus pneumoniae R6 | NC_003098 | no | 108 | 1935 | 0.052 |
| Geobacillus thermodenitrificans NG80-2 | NC_009328 | yes | 178 | 3214 | 0.052 |
| Candidatus Desulforudis audaxviator MP104C | NC_010424 | yes | 113 | 2044 | 0.052 |
| Bacillus cereus subsp. cytotoxis NVH 391-98 | NC_009674 | yes | 200 | 3633 | 0.052 |
| Lactococcus lactis subsp. lactis Il1403 | NC_002662 | no | 121 | 2200 | 0.052 |
| Bacillus cereus ATCC 10987 | NC_003909 | yes | 292 | 5311 | 0.052 |
| Exiguobacterium sibiricum 255-15 | NC_010556 | yes | 156 | 2851 | 0.051 |
| Streptococcus pyogenes M1 GAS | NC_002737 | no | 88 | 1609 | 0.051 |
| Bacillus anthracis str. Ames | NC_003997 | yes | 275 | 5036 | 0.051 |
| Bacillus anthracis str. 'Ames Ancestor' | NC_007530 | yes | 274 | 5035 | 0.051 |
| Streptococcus pneumoniae Hungary19A-6 | NC_010380 | no | 111 | 2044 | 0.051 |
| Bacillus licheniformis ATCC 14580 | NC_006270 | yes | 215 | 3963 | 0.051 |
| Bacillus halodurans C-125 | NC_002570 | yes | 209 | 3857 | 0.051 |
| Pelotomaculum thermopropionicum SI | NC_009454 | yes | 150 | 2770 | 0.051 |
| Lactobacillus delbrueckii subsp. bulgaricus ATCC BAA-365 | NC_008529 | no | 88 | 1633 | 0.051 |
| Bacillus anthracis str. Sterne | NC_005945 | yes | 270 | 5017 | 0.051 |
| Bacillus clausii KSM-K16 | NC_006582 | yes | 209 | 3887 | 0.051 |
| Bacillus thuringiensis serovar konkukian str. 97-27 | NC_005957 | yes | 260 | 4857 | 0.050 |
| Streptococcus pneumoniae D39 | NC_008533 | no | 96 | 1818 | 0.050 |
| Bacillus thuringiensis str. Al Hakam | NC_008600 | yes | 237 | 4499 | 0.050 |
| Lactobacillus casei ATCC 334 plasmid 1 | NC_008502 | no | 1 | 19 | 0.05 |
| Geobacillus kaustophilus HTA426 | NC_006510 | yes | 174 | 3324 | 0.049 |
| Streptococcus gordonii str. Challis substr. CH1 | NC_009785 | no | 101 | 1950 | 0.049 |
| Bacillus cereus E33L | NC_006274 | yes | 251 | 4883 | 0.048 |
| Bacillus anthracis str. 'Ames Ancestor' plasmid pXO2 | NC_007323 | no | 5 | 99 | 0.048 |
| Lactobacillus delbrueckii subsp. bulgaricus ATCC 11842 | NC_008054 | no | 75 | 1487 | 0.048 |
| Lactobacillus salivarius UCC118 | NC_007929 | no | 82 | 1635 | 0.047 |
| Bacillus cereus ATCC 14579 plasmid pBClin15 | NC_004721 | no | 1 | 20 | 0.047 |
| Clostridium perfringens str. 13 plasmid pCP13 | NC_003042 | no | 3 | 60 | 0.047 |
| Lactococcus lactis subsp. cremoris SK11 | NC_008527 | no | 113 | 2271 | 0.047 |
| Listeria monocytogenes EGD-e | NC_003210 | yes | 134 | 2712 | 0.047 |
| Lactobacillus casei | NC_010999 | no | 142 | 2902 | 0.046 |
| Caldicellulosiruptor saccharolyticus DSM 8903 | NC_009437 | yes | 124 | 2555 | 0.046 |
| Lactococcus lactis subsp. cremoris MG1363 | NC_009004 | no | 112 | 2322 | 0.046 |
| Streptococcus equi subsp. zooepidemicus str. MGCS10565 | NC_011134 | no | 85 | 1808 | 0.044 |
| Listeria monocytogenes str. 4b F2365 | NC_002973 | yes | 126 | 2695 | 0.044 |
| Streptococcus sanguinis SK36 | NC_009009 | no | 101 | 2169 | 0.044 |
| Staphylococcus saprophyticus subsp. saprophyticus ATCC 15305 plasmid pSSP1 | NC_007351 | no | 2 | 43 | 0.044 |
| Enterococcus faecalis V583 | NC_004668 | no | 138 | 2975 | 0.044 |
| Thermoanaerobacter tengcongensis MB4 | NC_003869 | yes | 113 | 2475 | 0.043 |
| Listeria innocua Clip11262 | NC_003212 | yes | 129 | 2839 | 0.043 |
| Clostridium botulinum F str. Langeland plasmid pCLI | NC_009700 | no | 1 | 23 | 0.041 |
| Listeria welshimeri serovar 6b str. SLCC5334 | NC_008555 | yes | 113 | 2661 | 0.040 |
| Thermoanaerobacter pseudethanolicus ATCC 33223 | NC_010321 | yes | 90 | 2153 | 0.040 |
| Listeria innocua Clip11262 plasmid pLI100 | NC_003383 | no | 3 | 72 | 0.04 |
| Carboxydothermus hydrogenoformans Z-2901 | NC_007503 | yes | 102 | 2518 | 0.038 |
| Finegoldia magna ATCC 29328 plasmid pFMC | NC_010371 | no | 7 | 175 | 0.038 |
| Lactobacillus salivarius UCC118 plasmid pSF118-20 | NC_006529 | no | 1 | 26 | 0.037 |
| Thermoanaerobacter sp. X514 | NC_010320 | yes | 86 | 2263 | 0.036 |
| Lactococcus lactis subsp. cremoris SK11 plasmid 3 | NC_008505 | no | 2 | 59 | 0.032 |
| Bacillus thuringiensis str. Al Hakam plasmid pALH1 | NC_008598 | no | 2 | 60 | 0.032 |
| Staphylococcus aureus subsp. aureus N315 plasmid pN315 | NC_003140 | no | 1 | 30 | 0.032 |
| Lactococcus lactis subsp. cremoris SK11 plasmid 4 | NC_008506 | no | 1 | 34 | 0.028 |
| Enterococcus faecalis V583 plasmid pTEF1 | NC_004669 | no | 2 | 70 | 0.027 |
| Bacillus weihenstephanensis KBAB4 plasmid pBWB403 | NC_010182 | no | 2 | 73 | 0.026 |
| Bacillus weihenstephanensis KBAB4 plasmid pBWB404 | NC_010183 | no | 1 | 70 | 0.014 |
| Natranaerobius thermophilus JW/NM-WN-LF plasmid pNTHE01 | NC_010715 | no | 0 | 14 | 0.0 |
| Exiguobacterium sibiricum 255-15 plasmid pEXIG01 | NC_010549 | no | 0 | 5 | 0.0 |
| Leuconostoc citreum KM20 plasmid pLCK3 | NC_010467 | no | 0 | 20 | 0.0 |
| Bacillus cereus subsp. cytotoxis NVH 391-98 plasmid pBC9801 | NC_009673 | no | 0 | 11 | 0.0 |
| Lactococcus lactis subsp. cremoris SK11 plasmid 2 | NC_008504 | no | 0 | 6 | 0.0 |
| Streptococcus thermophilus LMD-9 plasmid 2 | NC_008501 | no | 0 | 2 | 0.0 |
| Streptococcus thermophilus LMD-9 plasmid 1 | NC_008500 | no | 0 | 4 | 0.0 |
| Lactobacillus brevis ATCC 367 plasmid 2 | NC_008499 | no | 0 | 22 | 0.0 |
| Lactobacillus brevis ATCC 367 plasmid 1 | NC_008498 | no | 0 | 11 | 0.0 |
| Bacillus cereus E33L plasmid pE33L5 | NC_007104 | no | 0 | 5 | 0.0 |
| Staphylococcus aureus subsp. aureus MSSA476 plasmid pSAS | NC_005951 | no | 0 | 19 | 0.0 |
| Staphylococcus epidermidis ATCC 12228 plasmid pSE-12228-03 | NC_005006 | no | 0 | 8 | 0.0 |
| Staphylococcus epidermidis ATCC 12228 plasmid pSE-12228-04 | NC_005005 | no | 0 | 16 | 0.0 |
| Staphylococcus epidermidis ATCC 12228 plasmid pSE-12228-05 | NC_005004 | no | 0 | 22 | 0.0 |
| Enterococcus faecalis V583 plasmid pTEF3 | NC_004670 | no | 0 | 18 | 0.0 |
