# Supplementary material for: Prediction of Type III Secretion Signals in Genomes of Gram-Negative Bacteria
Source: PLoS One. 2009 Jun 15;4(6):e5917. doi: 10.1371/journal.pone.0005917 (PMC2690842; doi:10.1371/journal.pone.0005917)
Supplement: Table S3 — Predicted proteins from the Yersinia enterocolitica strain 8081 virulence plasmid that might be exported via a Type 3 Secretion System. Higher score values indicate more reliable predictions. (0.04 MB DOC) [file pone.0005917.s007.doc]

**Table S3.** Predicted proteins from the *Yersinia enterocolitica* strain 8081 virulence plasmid that might be exported *via* a Type 3 Secretion System. Higher score values indicate more reliable predictions.

| **Gene name, protein name** | **ANN score** |
| --- | --- |
| gi|122815811|ref|YP_001004077.1| type III secretion outer membrane protein | 1.0 |
| gi|122815800|ref|YP_001004066.1| translocator protein | 1.00 |
| gi|122815839|ref|YP_001004105.1| putative outer membrane virulence protein | 1.00 |
| gi|122815795|ref|YP_001004061.1| plasmid type III secretion system effector protein | 0.98 |
| gi|122815798|ref|YP_001004064.1| yop type III secretion system effector protein | 0.95 |
| gi|122815854|ref|YP_001004120.1| putative targeted effector protein | 0.87 |
| gi|122815829|ref|YP_001004095.1| putative type III secretion protein | 0.87 |
| gi|122815842|ref|YP_001004108.1| plasmid-partitioning protein | 0.81 |
| gi|122815853|ref|YP_001004119.1| putative targeted effector protein kinase | 0.78 |
| gi|122815801|ref|YP_001004067.1| virulence plasmid YopB transmembrane effector protein | 0.67 |
| gi|122815834|ref|YP_001004100.1| putative type III secretion regulatory protein | 0.63 |
| gi|122815809|ref|YP_001004075.1| YopN chaperone SycN | 0.58 |
| gi|122815850|ref|YP_001004116.1| putative resolvase/recombinase | 0.56 |
| gi|122815836|ref|YP_001004102.1| putative protein-tyrosine phosphatase Yop effector | 0.52 |
| gi|122815820|ref|YP_001004086.1| putative type III secretion effector targeting lipoprotein | 0.49 |
| gi|122815814|ref|YP_001004080.1| putative type III secretion protein | 0.46 |
