# Supplementary material for: Prediction of Type III Secretion Signals in Genomes of Gram-Negative Bacteria
Source: PLoS One. 2009 Jun 15;4(6):e5917. doi: 10.1371/journal.pone.0005917 (PMC2690842; doi:10.1371/journal.pone.0005917)
Supplement: Table S4 — Predicted proteins from Helicobacter pylori strain 26695 that might be exported via a Type 3 Secretion System. Higher score values indicate more reliable predictions. (0.08 MB DOC) [file pone.0005917.s008.doc]

**Table S4.** Predicted proteins from *Helicobacter pylori* strain 26695 that might be exported *via* a Type 3 Secretion System. Higher score values indicate more reliable predictions.

| Gene name, protein name | ANN score |
| --- | --- |
| gi|15645522|ref|NP_207698.1| hypothetical protein HP0906 | 1.00 |
| gi|15644743|ref|NP_206913.1| hypothetical protein HP0113 | 0.98 |
| gi|15645579|ref|NP_207755.1| hypothetical protein HP0963 | 0.90 |
| gi|15645509|ref|NP_207684.1| hypothetical protein HP0891 | 0.89 |
| gi|15645733|ref|NP_207910.1| flagellar hook-associated protein 1 (HAP1) (flgK) | 0.88 |
| gi|15645370|ref|NP_207544.1| polar flagellin (flaG) | 0.88 |
| gi|15646164|ref|NP_208348.1| flagellar basal-body protein (fliE) | 0.87 |
| gi|15644764|ref|NP_206934.1| 3-deoxy-D-arabino-heptulosonate 7-phosphate synthase (dhs1) | 0.86 |
| gi|15646192|ref|NP_208376.1| flagellar basal-body rod protein (flgG) | 0.84 |
| gi|15644995|ref|NP_207165.1| hypothetical protein HP0367 | 0.82 |
| gi|15646039|ref|NP_208221.1| conserved hypothetical ATP-binding protein | 0.80 |
| gi|15645055|ref|NP_207225.1| hypothetical protein HP0427 | 0.79 |
| gi|15646018|ref|NP_208199.1| hypothetical protein HP1408 | 0.79 |
| gi|15646016|ref|NP_208197.1| biotin synthetase (bioB) | 0.72 |
| gi|15645469|ref|NP_207643.1| type I restriction enzyme M protein (hsdM) | 0.72 |
| gi|15645157|ref|NP_207327.1| cag pathogenicity island protein (cag11) | 0.68 |
| gi|15645809|ref|NP_207986.1| translation elongation factor EF-G (fusA) | 0.67 |
| gi|15645756|ref|NP_207933.1| hypothetical protein HP1142 | 0.67 |
| gi|15645034|ref|NP_207204.1| hypothetical protein HP0406 | 0.66 |
| gi|15645173|ref|NP_207343.1| cag pathogenicity island protein (cag26) | 0.66 |
| gi|15645496|ref|NP_207671.1| Holliday junction endodeoxyribonuclease (ruvC) | 0.66 |
| gi|15646041|ref|NP_208223.1| histidine and glutamine-rich protein | 0.64 |
| gi|15644762|ref|NP_206932.1| L-serine deaminase (sdaA) | 0.63 |
| gi|15644659|ref|NP_206828.1| citrate synthase (gltA) | 0.63 |
| gi|15646103|ref|NP_208285.1| UDP-MurNac-tripeptide synthetase (murE) | 0.62 |
| gi|15645928|ref|NP_208107.1| ribosomal protein S19 (rps19) | 0.61 |
| gi|15645605|ref|NP_207781.1| hypothetical protein HP0990 | 0.61 |
| gi|15645367|ref|NP_207541.1| cell division protein (ftsE) | 0.61 |
| gi|15645286|ref|NP_207456.1| ribonuclease III (rnc) | 0.60 |
| gi|15645679|ref|NP_207856.1| hypothetical protein HP1065 | 0.60 |
| gi|15644745|ref|NP_206915.1| flagellin B (flaB) | 0.60 |
| gi|15644939|ref|NP_207109.1| hypothetical protein HP0311 | 0.60 |
| gi|15645818|ref|NP_207996.1| ribosomal protein L33 (rpL33) | 0.57 |
| gi|15644876|ref|NP_207046.1| hypothetical protein HP0248 | 0.56 |
| gi|15645292|ref|NP_207462.1| hypothetical protein HP0668 | 0.56 |
| gi|15646061|ref|NP_208243.1| thiophene and furan oxidizer (tdhF) | 0.56 |
| gi|15645854|ref|NP_208032.1| hypothetical protein HP1240 | 0.55 |
| gi|15645245|ref|NP_207415.1| DNA mismatch repair protein (MutS) | 0.55 |
| gi|15645524|ref|NP_207700.1| flagellar hook (flgE) | 0.54 |
| gi|15645847|ref|NP_208025.1| hypothetical protein HP1233 | 0.54 |
| gi|15645648|ref|NP_207824.1| ATP-binding protein (ylxH) | 0.53 |
| gi|15645510|ref|NP_207685.1| hypothetical protein HP0892 | 0.53 |
| gi|15644790|ref|NP_206960.1| hypothetical protein HP0161 | 0.52 |
| gi|15646174|ref|NP_208358.1| conserved hypothetical GTP-binding protein | 0.51 |
| gi|15645238|ref|NP_207408.1| ABC transporter, ATP-binding protein | 0.51 |
| gi|15646175|ref|NP_208359.1| hypothetical protein HP1568 | 0.51 |
| gi|15645296|ref|NP_207466.1| solute-binding signature and mitochondrial signature protein (aspB) | 0.50 |
| gi|15645664|ref|NP_207841.1| homoserine kinase (thrB) | 0.50 |
| gi|15645491|ref|NP_207666.1| alkylphosphonate uptake protein (phnA) | 0.49 |
| gi|15644923|ref|NP_207093.1| flagellin B homolog (fla) | 0.49 |
| gi|15645489|ref|NP_207664.1| flagellar hook (flgE) | 0.49 |
| gi|15646108|ref|NP_208290.1| hypothetical protein HP1499 | 0.49 |
| gi|15644992|ref|NP_207162.1| ribonucleoside diphosphate reductase, beta subunit (nrdB) | 0.48 |
| gi|15645118|ref|NP_207288.1| ribosomal protein L28 (rpL28) | 0.48 |
| gi|15645589|ref|NP_207765.1| phosphoglycerate mutase (pgm) | 0.48 |
| gi|15644996|ref|NP_207166.1| hypothetical protein HP0368 | 0.48 |
| gi|15646084|ref|NP_208266.1| lipopolysaccharide core biosynthesis protein (kdtB) | 0.48 |
| gi|15645194|ref|NP_207364.1| GTP-binding protein (gtp1) | 0.47 |
| gi|15644662|ref|NP_206831.1| dethiobiotin synthetase (bioD) | 0.47 |
| gi|15645472|ref|NP_207647.1| ABC transporter, ATP-binding protein (yheS) | 0.47 |
| gi|15644757|ref|NP_206927.1| outer membrane protein (omp4) | 0.47 |
| gi|15645226|ref|NP_207396.1| flagellin A (flaA) | 0.47 |
| gi|15644901|ref|NP_207071.1| hypothetical protein HP0273 | 0.46 |
| gi|15645980|ref|NP_208160.1| type IIS restriction enzyme M2 protein (mod) | 0.46 |
| gi|15644878|ref|NP_207048.1| oligopeptide ABC transporter, ATP-binding protein (oppD) | 0.46 |
| gi|15644989|ref|NP_207159.1| pseudouridylate synthase I (hisT) | 0.46 |
| gi|15646151|ref|NP_208335.1| toxR-activated gene (tagE) | 0.46 |
| gi|15645757|ref|NP_207934.1| hypothetical protein HP1143 | 0.45 |
| gi|15645542|ref|NP_207718.1| hypothetical protein HP0926 | 0.45 |
| gi|15646001|ref|NP_208182.1| hypothetical protein HP1391 | 0.45 |
| gi|15646104|ref|NP_208286.1| transaldolase (tal) | 0.45 |
| gi|15645424|ref|NP_207598.1| lipooligosaccharide 5G8 epitope biosynthesis-associated protein (lex2B) | 0.45 |
| gi|15645498|ref|NP_207673.1| hypothetical protein HP0879 | 0.45 |
| gi|15645147|ref|NP_207317.1| cag pathogenicity island protein (cag1) | 0.44 |
| gi|15645959|ref|NP_208138.1| glyceraldehyde-3-phosphate dehydrogenase (gap) | 0.44 |
| gi|15644759|ref|NP_206929.1| hypothetical protein HP0129 | 0.44 |
| gi|15645104|ref|NP_207274.1| glutamyl-tRNA synthetase (gltX) | 0.44 |
| gi|15644665|ref|NP_206834.1| hypothetical protein HP0032 | 0.43 |
| gi|15645580|ref|NP_207756.1| hypothetical protein HP0964 | 0.43 |
| gi|15646149|ref|NP_208333.1| hypothetical protein HP1542 | 0.43 |
| gi|15645459|ref|NP_207633.1| flaA1 protein | 0.42 |
| gi|15646068|ref|NP_208250.1| hypothetical protein HP1459 | 0.42 |
| gi|15645388|ref|NP_207562.1| molybdopterin-guanine dinucleotide biosynthesis protein A (mobA) | 0.42 |
| gi|15645484|ref|NP_207659.1| deoxyuridine 5'-triphosphate nucleotidohydrolase (dut) | 0.42 |
| gi|15645045|ref|NP_207215.1| methionyl-tRNA synthetase (metS) | 0.41 |
| gi|15646129|ref|NP_208311.1| hypothetical protein HP1520 | 0.41 |
| gi|15646165|ref|NP_208349.1| flagellar basal-body rod protein (flgC) (proximal rod protein) | 0.41 |
| gi|15645567|ref|NP_207743.1| hypothetical protein HP0951 | 0.41 |
| gi|15645893|ref|NP_208071.1| anthranilate isomerase (trpC) | 0.41 |
| gi|15645327|ref|NP_207498.1| hypothetical protein HP0704 | 0.40 |
| gi|15644981|ref|NP_207151.1| flagellar export protein (fliH) | 0.40 |
| gi|15645302|ref|NP_207472.1| hypothetical protein HP0678 | 0.40 |
| gi|15645680|ref|NP_207857.1| hypothetical protein HP1066 | 0.40 |
