# Supplementary material for: Correction: Prediction of Type III Secretion Signals in Genomes of Gram-Negative Bacteria
Source: PLoS One. 2009 Jul 15;4(7):10.1371/annotation/78c8fc32-b1e2-4c87-9c92-d318af980b9b. doi: 10.1371/annotation/78c8fc32-b1e2-4c87-9c92-d318af980b9b (PMC2727526; doi:10.1371/annotation/78c8fc32-b1e2-4c87-9c92-d318af980b9b)
Supplement: Supplementary file 1 [file pone.78c8fc32-b1e2-4c87-9c92-d318af980b9b.s001.pdf]

Negative Training Data

```
> Q46M54|TFDS_RALEJ HTH -type transcriptional regulator tfdS  - Ralstonia
eutropha (strain JMP134) (Alcaligenes eutrophus).
MEFRQLRYFVAAAEEGNVGAAARRLHISQPPVTRQIHALEQHLGVLLFER
SARGVQLTPAGAAFLEDARRMLELGRTSVDRSRAASRGEIGQLDIGYLGT
AIYQTVPALLHAFTQAVPGATLSLALMPKVRQIEALRAGTIHLGVGRFYP
QEPGITVEHLHYERLYIAAGSSIARQLRQDPTLLRLKSESLVLFPKEGRP
SFADEVIALMRRAGVEPRVTAIVEDVNAALGLVAAGAGVTLVPASVAAIR
RPFVRTMEMADASAKVPVSLTYLTDSRVPVLRAFLDVARRGKGQK
> P0A921|PA1_ECOLI Phospholipase A1  - Escherichia coli.
MRTLQGWLL PVFMLPMAVYAQEATVKEVHDAPAVRGSIIANMLQEHDNPF
TLYPYDTNYLIYTQTSDLNKEAIASYDWAENARKDEVKFQLSLAFPLWRG
ILGPNSVLGASYTQKSWWQLSNSEESSPFRETNYEPQLFLGFATDYRFAG
WTLRDVEMGYNHDSNGRSDPTSRSWNRLYTRLMAENGNWLVEVKPWYVVG
NTDDNPDITKYMGYYQLKIGYHLGDAVLSAKGQYNWNTGYGGAELGLSYP
ITKHVRLYTQ VYSGYGESLIDYNFNQTRVGVGVMLNDLF
> CHEA_SALTY P09384 CHEMOTAXIS PROTEIN CHEA (EC 2.7.3. -). - S
MSMDISDFYQTFFDEADELLADMEQHLLDLVPESPDAEQLNAIFRAAHSI
KGGAGTFGFTILQETTHLMENLLDEARRGEMQLNTDIINLFLETKDIMQE
QLDAYKNSEEPDAASFEYICNALRQLALEAKGETTPAVVETAALSAAIQE
ESVAETESPRD ESKLRIVLSRLKANEVDLLEEELGNLATLTDVVKGADSL
SATLDGSVAEDDIVAVLCFVIEADQIAFEKVVAAPVEKAQEKTEVAPVAP
PAVVAPAAKSAAHEHHAGREKPARERESTSIRVAVEKVDQLINLVGELVI
TQSMLAQRSNELDPVNHGDLITSMGQLQRNARDLQESVMSIRMMPMEYVF
SRFPRLVRDLAGKLGKQVELTLVGSSTELDKSLIERIIDPLTHLVRNSLD
HGIEMPEKRLEA GKNVVGNLILSAEHQGGNICIEVTDDGAGLNRERILAK
AMSQGMAVNENMTDDEVGMLIFAPGFSTAEQVTDVSGRGVGMDVVKRNIQ
EMGGHVEIQSKQGSGTTIRILLPLTLAILDGMSVRVAGEVFILPLNAVME
SLQPREEDLHPLAGGERVLEVRGEYLPLVELWKVFDVDGAKTEATQGIVV
ILQSAGRRYALLVDQLIGQHQVVVKNLESNYRKVPGISAATILGDGSVAL
IVDVSALQGLNRE QRMAITAA
> CHEW_ENTAE P21821 PURINE -BINDING CHEMOTAXIS PROTEIN.  - ENTE
MAGLATVSKLAGETVGQAFLIFTLGNEEYGIDILKVQKIRGYDQVTRIAN
TPDFIKGVTNLRGVIVPIIDLRVKYAQQGVSYDENTVVIVLNFGQRVVGI
VVDGVSDVLSLTAEQIRPAPEFAVTMATEYLTGLGALGALLILVDIEKLL
STEEMALVDNVAKSH
> P32557|DSBA_VI BCH Thiol:disulfide interchange protein dsbA  - Vibrio
cholerae.
MKKLFALVATLMLSVSAYAAQFKEGEHYQVLKTPASSSPVVNEFFSFYCP
HCNTFEPIIAQLKQQLPEGAKFQKNHVSFMGGNMGQAMSKAYATMIALEV
EDKMVPVMFNRIHTLRKPPKDEQELRQIFLDEGIDAAKFDAAYNGFAVDS
MVRRFDKQFQDSGLTGVPAVVVNNRYLVQGQSVKSLDEY FDLVNYLLTLK

> P05149|GALM_ACICA Aldose 1 -epimerase - Acinetobacter calcoaceticus.
MKKLAILGVTVYSFAQLANAATLNVKSYGTTQNGQKVDLYTMSNNNGVSV
SFISFGGVITQILTPDAQGKQNNIVLGFDDLKGYEVTDTKEGIHFGGLIG
RYANRIGNAKFSLDGKTYNLEKNNGPNSLHSGNPGFDKRVWQVKPLVSKG
ETVKASLKLTSPNGDQGFPG KLDVEVIYSLSDQNEFKIEYKAKTDQPTVV
NLTNHSYFNLSGAGNNPYGVLDHVVQLNAGRILVTDQNSLPTGEIASVAG
TPFDFRMPKAIVKDIRANNQQLAYGYGYDQTWVINQKSQGKLNLAAIVVD
PKSKRTMQVLTTEPSVQMYTADHLLGNIVGANGVLYRQADALALETQHFP
DSPNQPTFPSTRLNPNQTYNSVTVFKFGVQK
> P14212|HIFA2_HAEIN Major fimbrial subu nit - Haemophilus influenzae.
MKKTLLGSLILLAFAGNVQADINTETSGKVTFFGKVVENTCKVKTEHKNL
SVVLNDVGKNSLSTKVNTAMPTPFTITLQNCDPTTANGTANKANKVGLYF
YSWKNVDKENNFTLKNEQTTADYATNVNIQLMESNGTKAISVVGKETEDF
MHTNNNGVALNQTHPNNAHISGSTQLTTGTNELPLHFIAQYYATNKATAG
KVQSSVDFQIAYE
> P22340|SCRY_SALTY Sucrose porin  - Salmonella typhimurium.
MYRKSTLAMLIALLTSAASAHAQTDISTIEARLNALEKRLQEAENRAQTA
ENRAGAAEKKVQQLTAQQQKNQNSTQEVAQRTARLEKKADDKSGFEFHGY
```

ARSGVIMNDSGASTKSGAYITPAGETGGAIGRLGNQADTYVEMNLEHKQT
LDNGATTRFKVMVADGQTSYNDWTASTSDLNVRQAFVELGNLPTFAGPFK
GSTLWAGKRFDRDNFDIHWIDSDVVFLAGTGGGIYDVKWNDGLRSNFSLY
GRNFGDIDDSSNSVQNYILTMNHFAGPLQMMVSGLRAKDNDERKDSNGNL
AKGDAANTGVHALLGLHNDSFYGLRDGSSKTALLYGHGLGAEVKGIGSDG
ALRPGADTWRIASYGTTPLSENWSVAPAMLAQRSKDRYADGDSYQWATFN
LRLIQAINQNFALAYEGSYQYMDLKPEGYNDRQAVNGSFYKLTFAPTFKV
GSIGDFFSRPEIRFYTSWMDWSKKLNNYASDDALGSDGFNSGGEWSFGVQ
METWF
> P43822|SYGB_HAEIN Glycyl -tRNA synthetase beta chain - Haemophilus influenzae.
MTTQNFLVEIGTEELPPKALKTLATSFADNVEAELNQAGLSFDKIEWFAA
PRRLAVKVLNLATQQPSKEIEKRGPAVSAAFDAEGKPTKAAEGWARGCGI
TVEQAERIATDKGEWLIH RAKIEGQPTKNLLNDIVANALAKLPIPKPMRW
ADKTVQFIRPVHTVTMLLGDELIEGEILGVASARTIRGHRFLGEKEFYIQ
HADQYPQLLREKGSVVADFNERKAEILAKSQAKATALGGVADIEESLLEE
VTSLVEYPNVLAAKFEEHFLAVPAEALVYTMKGDQKYFPIYDKDGKLLPH
FIFVSNINPEDPTAIIEGNEKVVRPRLTDAEFFFKTDLKQKLVDRLPRLE
TVLFQQQLGTLKDKTDRIE QLAGEIAKQIGADEAKAKRAGLLSKCDLMTN
MVFEFTDTQGVMGMHYARHDGEDEEVAVALNEQYMPRFAGDELPKSLVAS
AVALADKFDTLTGIFGIGQAPKGSADPFALRRAALGALRIIVEKNLPLDL
EDLVKKSAALFGDKLTNQNVVADVVDFMLGRFRAWYQDEGIAVDVIQAVL
ARRPTRPADFDARVRAVSHFRTLDSAEALAAANKRVSNILAKAGAAIGEI
NLTACVEPAEKALAEAVLAL RTEVQPLIAQGDYTTVLDKLANLRAPVDSF
FDNVMVNAEDPALRQNRLAILNTLQGLFLQVADISVLQ
> P0A2S9|RHAS_SALTY HTH -type transcriptional activator rhaS - Salmonella typhimurium.
MTVLHSVDFFPSGKAPVAIEPRLPQAAFPEHHHDFHEIVIVEHGTGIHVF
NGQPYTISGGTVCFVRDHDRHLYEHTDNLCLTNVLWRSPDAFQFLAGLD Q
LLPQEQDGYYPSHWRVNQSVLQQVRQLVGLMERAGDGMDAPAVANREILF
MQLLVLLRRSSLMEGATNNDAKLNQLMAWLEDHFAEEVCWEAVAEQFSLS
LRTLHRQLKQHTGLTPQRYLNRLRLIKARHLLRHSDHSVTEIAYRCGFGD
SNHFSTLFRREFNWSPRDIRQGRDAIIQ
> P62088|PRSX_ECOLI HTH -type transcriptional regulator prsX - Escherichi a coli.
MNNTDTLEKIIRHQKNKDPAYPFQEHLLMQLCIRANKRMQDNISEFLGAY
GINHSVYMVLTTLFTAESHCLSPSEISQKLQFTRTNITRITDFLEKTGYV
KRTDSREDRRAKKISLTSEGMFFIQRLTLAQSMYLKEIWGYLTHDEQELF
EVINKKLLAHLDDVSS
> P22541|GUNA_BUTFI Endoglucanase A - Butyrivibrio fibrisolvens.
MVSKKQKFLTVIL VIVLAIVIVGGVFGISFVKGRVTFPWQLQNSEAKTEQ
VKEPAKEEPKLVIKEKKQDESAKKEQELKKAKEEAEAAVEKETEKTEEEP
VDNLLNDMKLKYYGKLAVEGSHLVDADGHEVLLMGVSTHGINWYPEYASA
ETIKSLRDTWGINVIRLAMYTSDYNGYCVAGKENQEKLKDIIDDAVEAAT
DNDMYVIIDWHTLNDADPNEYKADAIQFFGEMVRKYKDNENVIYEICNEP
NGDTTWNDVRRYAN EVIPVIRNVDAIILVGTPKWATDLDSVLDKPLDFDN
IMYTYHFYAGTHHKAERNALRDALDEGLPVFISEYGLVDADGDGNLNEKE
ADYWYDMIRKEYGVSSCMWNLSNKDEGSAMINADCDKLSDFTEEDLSESA
MWLIDQISQLKHSDLEQGVDWITPENNNR
> P32722|PORD_PSEAE Porin D - Pseudomonas aeruginosa.
MKVMKWSAIALAVSAGSTQFAVADAFVSDQAEA KGFIEDSSLDLLLRNYY
FNRDGKSGSGDRVDWTQGFLTTYESGFTQGTVGFGVDAFGYLGLKLDGTS
DKTGTGNLPVMNDGKPRDDYSRAGGAVKVRISKTMLKWGEMQPTAPVFAA
GGSRLFPQTATGFQLQSSEFEGLDLEAGHFTEGKEPTTVKSRGELYATYA
GETAKSADFIGGRYAITDNLSASLYGAELEDIYRQYYLNSNYTIPLASDQ
SLGFDFNIYRTNDEGKAKAGDISNTTWSLAAAYT LDAHTFTLAYQKVHGD
QPFDYIGFGRNGSGAGGDSIFLANSVQYSDFNGPGEKSWQARYDLNLASY
GVPGLTFMVRYINGKDIDGTKMSDNNVGYKNYGYGEDGKHHETNLEAKYV
VQSGPAKDLSFRIRQAWHRANADQGEGDQNEFRLIVDYPLSIL
> P17052|RURE_PSEOL Rubredoxin -NAD(+) reductase - Pseudomonas oleovorans.
MAIVVVGAGTAGVNAAFWL RQYGYKGEIRIFSRESVAPYQRPPLSKAFLT
SEIAESAVPLKPEGFYTNNNITISLNTPIVSIDVGRKIVSSKDGKEYAYE
KLILATPASARRLTCEGSELSGVCYLRSMEDAKNLRRKLVESASVVVLGG

GVIGLEVASAAVGLGKRVTVIEATPRVMARVVTPAAANLVRARLEAEGIE
FKLNAKLTSIKGRNGHVEQCVLESGEEIQADLIVVGIGAIPELELATEAA
LEVSNGVVVDDQMCTSDTSI YAIGDCAMARNPFWGTMVRLETIHNAVTHA
QIVASSICGTSTPAPTPPRFWSDLKGMALQGLGALKDYDKLVVAINNETL
ELEVLAYKQERLIATETINLPKRQGALAGSIKLPD
> P15713|PHLN_PSEAE Non -hemolytic phospholipase C - Pseudomonas aeruginosa.
MISKSRRSFIRLAAGTVGATVATSMLPSSIQAALAIPAHRRHGNLKDVEH
VVILMQENRSF DHYFGTLKGVRGFGDRMAIPLPDGQRVWHQKGSKGEILP
YHFDTSTTSAQRVDGTPHTWPDAQQAWNEGRMDKWLPAKTERSLGYYKEQ
DIAFQFAMANAFTICDAYHCSFQGGTNPNRLFLWTGTNDPLGQHGGPVTT
NDHDSNGPVEQGYTWTTYPERLQAAGITWRVYQDMADNFSDNPLIGFRQY
RAAAPDSPLIVNGLSTWKLDALKRDVLANSLPQVSWIVAPAKYSEHPGPS
SPIWGAEYTSWV LDALTANPEVWSKTALLVMFDENDGFFDHVAPPAAPSL
NKDGTLRGKTTADATLEWHTKGDIRYRNQPYGLGPRVPMYVISPWSKGGW
VNSQVFDHTSVIRFLEQRFGVMEPNISPWRRAVCGDLTSAFNFANPNNEP
FPELPDTSQADAIVASQIKLPKPKPPAVAAMPKQEMGIRPARALPYELGV
HARYRSGGDALSLTFANTGKAGAVFQVFDLLDSENPPKRYTVGARKRLHD
SFQGDASGDYHLE VHGPNGFLRVFRGNLRRDLAERKAPLPEVRIDYEPLF
GNLRVQLINRGRHPVKLTVKDNVYRQGERRTVNVPPGQRREVRYSLRSSG
NWYDFSVSAQGADSFLRRFSGRMEDGRSGFSDPGMGLGTLTF
> P43827|SYL_HAEIN Leucyl -tRNA synthetase - Haemophilus influenzae.
MQEQYRPDMIEPKVQQYWAENKVFKAIKDESKEKYYCLSMFPYPSGRLHM
GHVRNYTIGDVISRYQRMLGKNVLQPFGWDAFGLPAEGAAIKNKTAPAKW
TYENIAYMKKQLQLLGFGFDWDREIATCKPEYYKWEQWFFTELYKKGLVY
KKTSTVNWCPNDETVLANEQVHEGCCWRCDTPVEQKEIPQWFIKITDYAE
QLLGGLDTLPQWPDMVKTMQRNWIGRSEGVEITFDVANTNEKVAVYTTRP
DTFYGVSYLGIAAAHPLASLAAQNNSELAAFIQEAKNAKVAEADLATMEK
KGMATGLFAIHPLTGDKLPIWVANFVLMHYGTGAVMAVPAHDQRDFEFAQ
KYSLPIKQVIAPLADEEIDLTKQAFVEHGKLVNSDEFDGKNFDGAFNGIA
DKLEKLGVGKRQVNYRLRDWGVSRQRYWGAPIPMLTLENGDVVPAPMEDL
PIILPEDVVMDGVKSPINADPNWAKTTFNDAPALKETDTFDTFMESSWYY
ARYTCPQYQNGMLDAEEANYWLPVDQYIGGIEHATMHLLYFRFFHKLLRD
AGFVTSEEPADKLLCQGMVLADAFYYTSPTNERIWVSPTQVTLERDEKGR
IIKATDPEGRELVHSGMTKMSKSKNNGIDPQEMVEKYGADTVRLFMMFAS
PAEMTLEWQESGVEGAKRFLGRVWNLVYQYQQNPAKTSLDLTALSAEQKV
LRREVHKTIAKVSDDIGRRQTFNTAIAAVMELMNKLTKASLDSEQDRAVM
AEALSAVVRMLYPITPHICFELWQALGNESAIDTAEWVKADEAAMVEDEK
LIVVQVNG KVRGKVTVATDADEDTVKTIAFADENVKKFIDNQHIVKVIYV
VGKLLNVVVKP
> P0A7A9|IPYR_ECOLI Inorganic pyrophosphatase - Escherichia coli.
MSLLNVPAGKDLPEDIYVVIEIPANADPIKYEIDKESGALFVDRFMSTAM
FYPCNYGYINHTLSLDGDPVDVLVPTPYPLQPGSVIRCRPVGVLKMTDEA
GEDAKLVAVPHSKLSKEYDHIKDVNDLPELLKA QIAHFFEHYKDLEKGKW
VKVEGWENAEAAKAEIVASFERAKNK
> P06971|FHUA_ECOLI Ferrichrome -iron receptor - Escherichia coli.
MARSKTAQPKHSLRKIAVVVATAVSGMSVYAQAAVEPKEDTITVTAAPAP
QESAWGPAATIAARQSATGTKTDTPIQKVPQSISVVTAEEMALHQPKSVK
EALSYTPGVSVGTRGASNTYDHLIIRGFAAEGQSQNNYLNGLK LQGNFYN
DAVIDPYMLERAEIMRGPVSVLYGKSSPGGLLNMVSKRPTTEPLKEVQFK
AGTDSLFQTGFDFSDSLDDDGVYSYRLTGLARSANAQQKGSEEQRYAIAP
AFTWRPDDKTNFTFLSYFQNEPETGYYGWLPKEGTVEPLPNGKRLPTDFN
EGAKNNTYSRNEKMVGYSFDHEFNDTFTVRQNLRFAENKTSQNSVYGYGV
CSDPANAYSKQCAALAPADKGHYLARKYVVDDEKLQNFSVDTQL QSKFAT
GDIDHTLLTGVDFMRMRNDINAWFGYDDSVPLLNLYNPVNTDFDFNAKDP
ANSGPYRILNKQKQTGVYVQDQAQWDKVLVTLGGRYDWADQESLNRVAGT
TDKRDDKQFTWRGGVNYLFDNGVTPYFSYSESFEPSSQVGKDGNIFAPSK
GKQYEVGVKYVPEDRPIVVTGAVYNLTKTNNLMADPEGSFFSVEGGEIRA
RGVEIEAKAALSASVNVVGSYTYTDAEYTTDTTYKGNTPAQVPKH MASLW
ADYTFFDGPLSGLTLGTGGRYTGSSYGDPANSFKVGSYTVVDALVRYDLA
RVGMAGSNVALHVNNLFDREYVASCFNTYGCFWGAERQVVATATFRF
> P45375|PHBB_CHRVI Acetoacetyl -CoA reductase - Chromatium vinosum
(Allochromatium vinosum).
MARIALVTGGIGGIGTSICTRLAKDGCTVVANCHPSEAAAAEEWKQARAA

EGFDIAVFTADVSSFDDSARMVREITEQVGPIDILVNCAGITRDKTFKKM
EQAHWEAVINVNLNSVFNVTRQVWDGMLERGFGRIINISSVNGQRGQFGQ
ANYSAAKAGMHGFTMALAQEGASKGVTVNTISPGYVETAMTLAMNDDVRN
SIISGIPMRRMAQPNEIAAAIAFLAGDESGYMTGANLPVNGGLFMH
> Q51883|PLAS_PHOLA Plastocyanin - Phormidium laminosum.
MKLIAQISRSLSLALFALVLMVGSFVAVMSPAAAETFTVKMGADSGLLQF
EPANVTVHPGDTVKWVNNKLPPHNILFDDKQVPGASKELADKLSHSQLMF
SPGESYEITFSSDFPAGTYTYYCAPHRGAGMVGKITVEG
> Q01551|TBUD_BURPI Phenol 2 -monooxygenase - Burkholderia pickettii (Ralstonia pickettii) (Pseudomonas pickettii).
MTKYNEAYCDVLIVGAGPAGVMAAAHLLSYGTTARPHRVRIFDATKEVNG
SDESTESLSTDVIADALNSGASGPEKDAASTTEDLPMLVTTLQVSDVLHD
TGDDTKIAYRETATEQQVLLLADTTANTSSTMNPRSMCEAGCRFHQIYQG
HCFPEYELDSERLRSVDGRAQVLEDEHETGQLRLERLGRPEELLELDEEN
SMSVVTNLKAAPYKFLMKDVDENFPGELSTSGGKTTSISADESAIDAALH
AVWDADDLGAAWHLDEASGLRAVDWNAAQWFKSGQPWTPDAAKSLQEGRV
FLAGDARHRHPPLTGIGKNTSIADCYNLTWKLLGVLLGVARADPARTYVA
ERVYIRMRAATDIAVDAEMESLAAKWITVQLTLSRSWISSAKEAERWDAV
LRDSAMSASKPMWTTSDMRASFDAGLMGHGHAHDHVTPTIKEFASSSISR
SISELASTSWWESRGWGNGGPFESLMEDARWTGAVESNCRYAAYDRDAPV
LHEHVAWVTRFTSRARTAVLEAAVGQAHVVDCWDVGLVEPALDDLDSAGA
GLHVAHHADQWPAQLDEAVWPRESLSDWRIVTDTSATGEGYQTSPREAPG
DYADLNADNAKAHFNGQFAGHKAYGDAAAADGGGCHGRILVGPAVRGRHL
HREIPLGEECQRAAQPLFKEV
> P43828|SYM_HAEIN Methionyl -tRNA synthetase - Haemophilus influenzae.
MTTQPRKILVTCALPYA NGAIHLGHMLEHIQADIWVRFQRMRGNKIHFVC
ADDAHGTPIMLNADKLGITPEELIAKAKADHIRDFAGFNISFDNYHSTHS
EENKQLTAEIYNKLKANGFIKSKVISQLFDPEKNMFLPDRFVKGTCPKCK
AEDQYGDNCEVCASTYSPMDLINPRSAVSGTTPIVKESEHFFFDLPAFEG
MLKEWTRSGSLQSEIANKMQEWFESDLQQWDISRDAPYFGFEIPGAKDKF
FYVWLDAPIGYMASFKNL CNREGIDFNEFWAEGSDAELYHFIGKDIVYFH
SLFWPAMLEGSGYRKPTNVFAHGYVTVDGAKMSKSRGTFIQASTYLNHID
PECLRYYYAAKLNDRIEDLDFNLEDFVQRVNTDIVNKLVNLASRNAGFIA
KRFEGKLADKLEDKSLFAEFTAQAEQIAAYYESREYNKTIREIMALTDKA
NKYIDEKAPWVIAKEEGKEAELQAVCSMGIELFRVLMSYLKPVLPKLAER
AETFLQAELRWDNIHQPLL GHTLAPFKALFSRLEKKQIDAVVEETKALFA
AANKAAEKTEAKPTALSAVEPIAETITIDDFAKLDMRVAKVLKCEAVPES
NKLLRFELDLGDHTRQVFSGIKAAYNKPEELEGRFVIMVANLAPRKMKFG
VSEGMILSAGTGGSDLFLLSADSGVTAGMQVK
> P16700|CYSP_ECOLI Thiosulfate -binding protein - Escherichia coli.
MAVNLLKKNSLALVASLLLAG HVQATELLNSSYDVSRELFAALNPPFEQQ
WAKDNGGDKLTIKQSHAGSSKQALAILQGLKADVVTYNQVTDVQILHDKG
KLIPADWQSRLPNNSSPFYSTMGFLVRKGNPKNIHDWNDLVRSDVKLIFP
NPKTSGNARYTYLAAWGAADKADGGDKGKTEQFMTQFLKNVEVFDTGGRG
ATTTFAERGLGDVLISFESEVNNIRKQYEAQGFEVVIPKTNILAEFPVAW
VDKNVQANGTEKAAKAYLNWLY SPQAQTIITDYYYRVNNPEVMDKLKDKF
PQTELFRVEDKFGSWPEVMKTHFTSGGELDKLLAAGRN
> P0AG80|UGPB_ECOLI Glycerol -3-phosphate-binding periplasmic protein - Escherichia coli.
MKPLHYTASALALGLALMGNAQAVTTIPFWHSMEGELGKEVDSLAQRFNA
ENPDYKIVPTYKGNYEQNLSAGIAAFRTGNAPAILQVYEVGTATMMAS KA
IKPVYDVFKEAGIQFDESQFVPTVSGYYSDSKTGHLLSQPFNSSTPVLYY
NKDAFKKAGLDPEQPPKTWQDLADYAAKLKASGMKCGYASGWQGWIQLEN
FSAWNGLPFASKNNGFDGTDAVLEFNKPEQVKHIAMLEEMNKKGDFSYVG
RKDESTEKFYNGDCAMTTASSGSLANIREYAKFNYGVGMMPYDADAKDAP
QNAIIGGASLWVMQGKDKETYTGVAKFLDFLAKPENAAEWHQKTGYLPI T
KAAYDLTREQGFYEKNPGADTATRQMLNKPPLPFTKGLRLGNMPQIRVIV
DEELESVWTGKKTPQQALDTAVERGNQLLRRFEKSTKS
> Q55806|SYT_SYNY3 Threonyl -tRNA synthetase - Synechocystis sp. (strain PCC 6803).
MAKTVVSPDTIALPRTSESEQLKKIRHTTSHVMAMAVQKLFPKAQVTIGP
WTETGFYYDFDVAEPFTEADLKAIKKEMVK IINKKLPVIREEISREEAKQ
RIESIQEPYKLEILDSIHEPITVYHLGDQWWDLCAGPHLESTADINPKAI

ALESVAGAYWRGDANKAQLQRIYGTAWETPEQLAEYQRRKEEALKRDHRK
LGKELGLFIFADPVGPGLPLWTPKGTIIRTILEDFLKQEQIKRGYLPVVT
PHIARVDLFKQSGHWQKYQEDMFPMMAESPEEAAQEMGFVLKPMNCPFHI
QIYKSELRSYRDLPLRLAEFGTVYRYEQSGE LGGLTRVRGFTVDDSHLFV
TPDQLDEEFLSVVDLILTVFKSLQLKNFKARLSFRDPESDKYIGSDEAWE
KAQGAIRRAVQELEMDYFEAPGEAAFYGPKLDFIFQDALEREWQLGTVQV
DYNLPERFDLEYIAADGSRQRPVMIHRAPFGSLERLIGILIEEYAGDFPL
WLAPIQVRLLAVSDEFLPFAQSIVQQMQSLGLRAEVDTSGDRLGKMIRNA
EKQKIPVMAVIGAKEVEANALNIRTRASGELG EIPVTEVLEKLQATVTNH
DTW
> NRDG_HAEIN P45080 ANAEROBIC RIBONUCLEOSIDE -TRIPHOSPHATE RED
MNYLQYYPTDVINGEGTRCTLFVSGCTHACKGCYNQKSWSFSAGVLFDDV
MEQQIINDLKDTRIKRQGLTLSGGDPLHPLNVETLLPFVQRVKRECPDKD
IWVWTGYKLDELDKQQRAMLPYIDVLIDGKFIQEQADPSLVWRGSANQII
HRFKL
> P27032|GUNY_DICD3 Minor endoglucanase Y - Dickeya dadantii (strain 3937) (Erwinia chrysanthemi (strain 3937)).
MGKPMWRCWALMLMVWFSASATAANGWEIYKSRFMTTDGRIQDTGNKNVS
HTEGQGFAMLMAVHYDDRIAFDNLWNWTQSHLRNTTSGLFYWRYDPSAAN
PVVDKNNASDGDVLIAWALLKAGNKWQDNRYLQASDSIQKAIIASNIIQF
AGRTVMLPGAYGFNKNSYVILNPSYFLFPAWRDFANRSHLQVWRQLIDDS
LSLVGEMRFGQVGLPTDWAALNADGSMAPATAWPSRFSYDAIRIPLYLYW
YDAKTTALVPFQLYWRNYPRLTTPAWVDVLSSNTATYNMQGGLLAVRDLT
MGNLDGLSDLPGASEDYYSSSLRLLVMLARGK
> P14532|CCPR_PSEAE Cytochrome c551 peroxidase - Pseudomonas aeruginosa.
MQSSQLLPLGSLLLSFATPLAQADALHDQASALFKPIPEQVTELRGQPIS
EQQRELGKKLFFDPRLSRSHVLSCNTCHNVGTGGADNVPTSVGHGWQKGP
RNSPTVFNAVFNAAQFWDGRAKDLGEQAKGPIQNSVEMHSTPQLVEQTLG
SIPEYVDAFRKAFPKAGKPVSFDNMALAIEAYEATLVTPDSPFDLYLKGD
DKALDAQQKKGLKAFMDSGCSACHNGINLGGQAYFPFGLVKKPDASVLPS
GDKGRFAVTKTQSDEYVFRAAPLRNVALTAPYFHSGQVWELKDAVAIMGN
AQLGKQLAPDDVENIVAFLHSLSGKQPRVEYPLLPASTETTPRPAE
> P27477|THTR_SYNP7 Putative thiosulfate sulfurtransferase - Synechococcus sp. (strain PCC 7942) (Anacystis nidulans R2).
MSVRSLRWPRQKAFLAVISLVVAVLLAVPGWLTPAT AASQATVQFVAPTW
AAERLNNKQLKILDVRTNPLAYIEGHLPGAVNIADAAYRGPNGFLPVQIW
DPEKLASLFGRAGVSNNDTVLVYSDGNDVLGATLVAYLLERSGVQNIAVL
DGGYKGYKDAGLPVTKEYPRYQAARFAPKDNRAFRVDIKQVEQLTGKSTF
VDPRPPALFSGEQQVFIRNGHIPGARNIPWPTFTEANNANESLKNPHKLK
PLSELKAILEAKGVTPDKDVIVTCSTGREASLQYLVL KHLLKYPKVRIYE
GSWTEYSASNLPVETGPDRV
> P0A948|RIMJ_ECOLI Ribosomal -protein-alanine acetyltransferase - Escherichia coli.
MFGYRSNVPKVRLTTDRLVVRLVHDRDAWRLADYYAENRHFLKPWEPVRD
ESHCYPSGWQARLGMINEFHKQGSAFYFGLFDPDEKEIIGVANFSNVVRG
SFHACYLGYSIGQKWQGKGLMFEALTAAIRYMQRT QHIHRIMANYMPHNK
RSGDLLARLGFEKEGYAKDYLLIDGQWRDHVLTALTTPDWTPGR
> P32520|FLAA1_TREHY Flagellar filament outer layer protein flaA1 - Treponema hyodysenteriae (Serpulina hyodysenteriae).
MKKLFVVLTSIFIAASAYGLTNSTLIDFALTGNADNLQAGEGDTNEVVPV
AENLYNDNWVVWLNESARLTENR RNSYVTNVDSKGNNGAWEAGKVLGVRV
HFPLAAWNSYALVKPVYELEMYGGADGTKYTEGKGVIHNVGEIKSISSWV
YGRNYLISYFVNLQNEFGELKSYPMGTVYFNGWRQVRWENREYLPNVRDR
VLVREPLYPRMIPSVKLDSLGFYRTKDTKGGDFITYVKDVTLEYDVVVVD
FEEDIDDEATWQLLKTENDRKQAIESARIREQAELRDLEQRRIGDGTAAD
QGAAANTGAADTGAAQEQAQ
> P28718|G6PI_ZYMMO Glucose -6-phosphate isomerase - Zymomonas mobilis.
MARIANKAAIDAAWKQVSACSEKTLKQLFEEDSNRLSGLVVETAKLRFDF
SKNHLDSQKLTAFKKLLEACDFDARRKALFAGEKINITEDRAVEHMAERG
QGAPASVARAKEYHARMRTLIEAIDAGAFGEVKHLLHIGIGGSALGPKLL
IDALTRESGRYDVAVVSNVDGQALEEVFKKFNPHK TLIAVASKTFTTAET
MLNAESAMEWMKKHGVEDPQGRMIALTANPAKASEMGIDDTRILPFAESI
GGRYSLWSSIGFPAALALGWEGFQQLLEGGAAMDRHFLEAAPEKNAPILA

AFADQYYSAVRGAQTHGIFAYDERLQLLPFYLQQLEMESNGKRVDLDGNL
IDHPSAFITWGGVGTDAQHAVFQLLHQGTRLVPIEFIAAIKADDTLNPVH
HKTLLTNAFAQGAALMSGRDNKDPARSYPGDRPSTT ILMEELRPAQLGAL
IAFYEHRTFTNGVLLGINSFDQFGVELGKEMAHAIADHPENSDFDPSTKA
LIAAALK
> P0A9A6|FTSZ_ECOLI Cell division protein ftsZ - Escherichia coli.
MFEPMELTNDAVIKVIGVGGGGGNAVEHMVRERIEGVEFFAVNTDAQALR
KTAVGQTIQIGSGITKGLGAGANPEVGRNAADEDRDALRAALEGADMVFI
AAGMGGGTGTGAA PVVAEVAKDLGILTVAVVTKPFNFEGKKRMAFAEQGI
TELSKHVDSLITIPNDKLLKVLGRGISLLDAFGAANDVLKGAVQGIAELI
TRPGLMNVDFADVRTVMSEMGYAMMGSGVASGEDRAEEAAEMAISSPLLE
DIDLSGARGVLVNITAGFDLRLDEFETVGNTIRAFASDNATVVIGTSLDP
DMNDELRVTVVATGIGMDKRPEITLVTNKQVQQPVMDRYQQHGMAPLTQE
QKPVAKVVNDNAPQ TAKEPDYLDIPAFLRKQAD
> P0A705|IF2_ECOLI Translation initiation factor IF -2 - Escherichia coli.
MTDVTIKTLAAERQTSVERLVQQFADAGIRKSADDSVSAQEKQTLIDHLN
QKNSGPDKLTLQRKTRSTLNIPGTGGKSKSVQIEVRKKRTFVKRDPQEAE
RLAAEEQAQREAEEQARREAEESAKREAQQKAEREAAEQAKREAAEQAKR
EAAEKDKVS NQQDDMTKNAQAEKARREQEAAELKRKAEEEARRKLEEEAR
RVAEEARRMAEENKWTDNAEPTEDSSDYHVTTSQHARQAEDESDREVEGG
RGRGRNAKAARPKKGNKHAESKADREEARAAVRGGKGGKRKGSSLQQGFQ
KPAQAVNRDVVIGETITVGELANKMAVKGSQVIKAMMKLGAMATINQVID
QETAQLVAEEMGHKVILRRENELEEAVMSDRDTGAAAEPRAPVVTIMGHV
DHGKTSLLDY IRSTKVASGEAGGITQHIGAYHVETENGMITFLDTPGHAA
FTSMRARGAQATDIVVLVVAADDGVMPQTIEAIQHAKAAQVPVVVAVNKI
DKPEADPDRVKNELSQYGILPEEWGGESQFVHVSAKAGTGIDELLDAILL
QAEVLELKAVRKGMASGAVIESFLDKGRGPVATVLVREGTLHKGDIVLCG
FEYGRVRAMRNELGQEVLEAGPSIPVEILGLSGVPAAGDEVTVVRDEKKA
REVALYRQGKF REVKLARQQKSKLENMFANMTEGEVHEVNIVLKADVQGS
VEAISDSLLKLSTDEVKVKIIGSGVGGITETDATLAAASNAILVGFNVRA
DASARKVIEAESLDLRYYSVIYNLIDEVKAAMSGMLSPELKQQIIGLAEV
RDVFKSPKFGAIAGCMVTEGVVKRHNPIRVLRDNVVIYEGELESLRRFKD
DVNEVRNGMECGIGVKNYNDVRTGDVIEVFEIIEIQRTIA
> Q55013|CY550_SYNY3 C ytochrome c-550 - Synechocystis sp. (strain PCC 6803).
MKRFFLVAIASVLFFFNTMVGSANAVELTESTRTIPLDEAGGTTTLTARQ
FTNGQKIFVDTCTQCHLQGKTKTNNNVSLGLADLAGAEPRRDNVLALVEF
LKNPKSYDGEDDYSELHPNISRPDIYPEMRNYTEDDIFDVAGYTLIAPKL
DERWGGTIYF
> O05947|SYT_RICPR Threonyl -tRNA synthetase - Rickettsia prowazekii.
MINISFPDGSVKQFAQNITAFEIVNAISMSLAKAAIVVEINGELKDLSTV
IENDCKLRILTAQDYECLEIIRHDAAHLTAEAVKELFPETQVTIGPAIEN
GYYYDFARDKPFSIDDLANIEAKMQELSKKNEKITRELWDRDKAVEFFLS
IGEHYKAKIIASIPEGEPITLYRQGNFIDLCRGPHSPSTGFVKHFKLMKV
AGAYWRGNSRNEMLQRIYGT AWATKEQLDNYLFMLEEAEKRDHRKIGKEL
DLFHFQEEAQGMVFWHDKGWSIYNTIEQYIRKKIRKNGYIEVKTPVLVDK
SLWEASGHWAKFRCDMFTLETDDKTLALKPMNCPCHVQIFKQGIKSYRDL
PLRMSEFGLCHRNEASGALHGLMRVRSLVQDDAHIFCAEEQITDETVRFC
KLLTEVYKDFGFTDIKVKFSDRSEICAGNDEVWDKAENALKTAIEKAGFI
YTLNPGEAAFYGPKIEFVLTD AIGRQWQCGTLQMDFVLPERLDANYIAAS
GEKKRPVMLHRAILGSLERFIGILIEEYAGKFPIWLAPVQVAIATITNDL
NDYALEVQKTLIDNGVRTDINISPDKINYKIREFSNQKIPIIAVIGKQEQ
ANKQVTIRKLGTIEQEILSIEQLIAMIKKENSNYL
> P56075|NDK_HELPY Nucleoside diphosphate kinase - Helicobacter pylori
(Campylobacter py lori).
MKQRTLSIIKPDALKKKVVGKIIDRFESNGLEVVAMKRLHLSVKDAENFY
AIHRERPFFKDLIEFMVSGPVVVMVLEGKDAVAKNRDLMGATDPKLAQKG
TIRADFAESIDANAVHGSDSLENAHNEIAFFFAARDL
> NEUA_NEIME Q57385 ACYLNEURAMINATE CYTIDYLYLTRANSFERASE (EC
MEKQNIAVILARQNSKGLPLKNLRKMNGISLLGHTINAAISSKCFDR IIV
STDGGLIAEEAKNFGVEVVLRPAELASDTASSISGVIHALETIGSNSGTV
TLLQPTSPLRTGAHIREAFSLFDEKIKGSVVSACPMEHHPLKTLLQINNG
EYAPMRHLSDLEQPRQQLPQAFRPNGAIYINDTASLIANNCFFIAPTKLY
IMSHQDSIDIDTELDLQQAENILNHKES

> P15930|FLGI_SALTY Flagellar P -ring protein - Salmonella typhimurium.
MFKALAGIVLALVATLAHAERIRDLTSVQGVRENSLIGYGLVVGLDGTGD
QTTQTPFTTQTLNNMLSQLGITVPTGTNMQLKNVAAVMVTASYPPFARQG
QTIDVVVSSMGNAKSLRGGTLLMTPLKGVDSQVYALAQGNILVGGAGASA
GGSSVQVNQLNGGRITNGAIIERELPTQFGAGNTINLQLNDEDFTMAQQI
TDAINRARGYGSATALDARTVQVRVPSGNSSQVRFLADIQNMEVNVTPQD
AKVVINSRTGSVVMNREVTLDSCAVAQGNLSVTVNRQLNVNQPNTPFGGG
QTVVTPQTQIDLRQSGGSLQSVRSSANLNSVVRALNALGATPMDLMSILQ
SMQSAGCLRAKLEII
> Q55267|HISZ_SYNP7 ATP phosphoribosyltransferase regulatory subunit - Synechococcus sp. (strain PCC 7942) (Anacystis nidulans R2).
MVHQPPAGTRDLLPQDVTQKRWIESRLQQVFQQWGYQRIITPTLERLDTL
VAGGAVQRSAVIQVQSDEESGLGLRPELTASIARAAVTRLAGSSLPLRLY
YLANVFRPAFQGDRLQQRELFQAGVELLGVGGTLADAEVLHVLADALAEL
GFGQPPLGSWHLVVGEASLTRSLLQPFPKDLREKVRQAIAQLDRVTLESL
PLESQLRDRALLLHDLRGQPDQVFAKLQQLTLTPLEQTLRDRLAQLVELY
NASAGPQDSPLLLDLSLLRSFDYYTGIVFEVVYETPTGPWVLAQGGRYDR
LLDVYDPQAAGQPGIGFSCNIENLQQVLLAANRLPHRPPAIDQLVIPVDS
EAYGAALAEAQRLQRQDQLRVELYLDSDRRPEVVQAFAQRRRIGRIVWVS
SGSAPQSEAVAVAERATTTC
> P07024|USHA_ECOLI Protein ushA [Includes: UDP -sugar hydrolase - Escherichia coli.
MKLLQRGVALALLTTFTLASETALAYEQDKTYKITVLHTNDHHGHFWRNE
YGEYGLAAQKTLVDGIRKEVAAEGGSVLLLSGGDINTGVPESDLQDAEPD
FRGMNLVGYDAMAIGNHEFDNPLTVLRQQEKWAKFPLLSANIYQKSTGER
LFKPWALFKRQDLKIAVIGLTTDDTAKIGNPEYFTDIEFRKPADEAKLVI
QELQQTEKPDIIIAATHMGHYDNGEHGSNAPGDVEMARALPAGSLAMIVG
GHSQDPVCMAAENKKQVDYVPGTPCKPDQQNGIWIVQAHEWGKYVGRADF
EFRNGEMKMVNYQLIPVNLKKKVTWEDGKSERVLYTPEIAENQQMISLLS
PFQNKGKAQLEVKIGETNGRLEGDRDKVRFVQTNMGRLILAAQMDRTGAD
FAVMSGGGIRDSIEAGDISYKNVLKVQPFGNVVVYADMTGKEVIDYLTAV
AQMKPDSGAYPQFANVSFVAKDGKLNDLKIKGEPVDPAKTYRMATLNFNA
TGGDGYPRLDNKPGYVNTGFIDAEVLKAYIQKSSPLDVSVYEPKGEVSWQ

> P27195|PPCF_ELIMR Prolyl endopeptidase - Elizabethkingia miricola (Chryseobacterium miricola).
MKYKKLSVAVAAFAFAAVSAQNSNSLKYPETKKVNHTDTYFGNQVSDPYR
WLEDDRAEDTKAWVQQEVKFTQDYLAQIPFRGQIKKQLLDIWNYEKISAP
FKKGKYTYFYKN DGLQAQSVLYRKDASGKTEVFLDPNKFSDKGTTSLANL
SFNKKGTLVAYSISEGGSDWNKIIILDAETKKQIDETLLDVKFSGISWLG
DEGFFYSSYDKPKDGSVLSGMTDKHKVYFHKLGTKQSQDELIIGGDKFPR
RYLSGYVTEDQRYLVVSAANATNGNELYIKDLKNKTDFIPIITGFESNVG
LVDTDGDTLFLHTDKNAPNMRMVKTTIQNPKPETWKDVIAETSEPMRVNS
GGGYFFATYMKDA LSQIKQYDKTGKLVREIKLPGSGTAGGFGGEKTEKEL
YYSFTNYITPPTIFKFSIDSGKSEVYQKPKVKFNPENYVSEQVFYTSADG
TKIPMMISNKKGLKKDGKNPTILYSYGGFNISLQPAFSVVNAIWMENGGI
YAVPNIRGGGEYGKKWHDAGTKQQKKNVFNDFIAAGEYLQKNGYTSKDYM
ALSGRSNGGLLVGATMTMRPDLAKVAFPGVGVLDMLRYNKFTAGAGWAYD
YGTAEDSKEMFEYL KSYSPVHNVKAGTCYPSTMVITSDHDDRVVPAHSFK
FGAELQAKQACKNPVLIRIETNAGHGAGRSTEQVVMENADLLSFALYEMG
IKNLK
> CYPB_HAEIN P44499 PEPTIDYL -PROLYL CIS-TRANS ISOMERASE B (EC
MVTLHTNFGDIKIKLDFDKAPVTAENFLNYCKDGFYNNTIFHRVIDGFMI
QGGGMESGMREKATKAPIQNEANNRLSNKRGTIAMARTSDPHSATAQFF I
NVADNDFLNYRSKEMFGREVVQEWGYAVFGEVVEGMDVVDKIKKVKTGNK
GFHQDVPTEDVVITSVSIE
> Q01911|TETX_BACFR Tetracycline resistance protein from transposon Tn4351/Tn4400 - Bacteroides fragilis.
MTMRIDTDKQMNLLSDKNVAIIGGGPVGLTMAKLLQQNGIDVSVYERDND
REARIFGGTLDLHKGSGQEAMKKAGL LQTYYDLALPMGVNIADKKGNILS
TKNVKPENRFDNPEINRNDLRAILLNSLENDTVIWDRKLVMLEPGKKKWT
LTFENKPSETADLVILANGGMSKVRKFVTDTEVEETGTFNIQADIHQPEI
NCPGFFQLCNGNRLMASHQGNLLFANPNNNGALHFGISFKTPDEWKNQTQ

VDFQNRNSVVDFLLKEFSDWDERYKELIHTTLSFVGLATRIFPLEKPWKS
KRPLPITMIGDAAHLMPPFAGQGVNSG LVDALILSDNLADGKFNSIEEAV
KNYEQQMFMYGKEAQEESTQNEIEMFKPDFTFQQLLNV
> P22390|BLAC_CITDI Beta -lactamase - Citrobacter diversus.
MFKKRGRQTVLIAAVLAFFTASSPLLARTQGEPTQVQQKLAALEKQSGGR
LGVALINTADRSQILYRGDERFAMCSTSKTMVAAAVLKQSETQHDILQQK
MVIKKADLTNWNPVTEKYVDKEMTLAELSAAT LQYSDNTAMNKLLEHLGG
TSNVTAFARSIGDTTFRLDRKEPELNTAIPGDERDTTCPLAMAKSLHKLT
LGDALAGAQRAQLVEWLKGNTTGGQSIRAGLPEGWVVGDKTGAGDYGTTN
DIAVIWPEDRAPLILVTYFTQPQQDAKGRKDILAAAAKIVTEGL
> P32823|CHIA_ALTSO Chitinase A - Alteromonas sp. (strain O -7).
MKLNKITSYIGFALLSGGALAAPSTP TLDWQPQQYSFVEVNVDGLGSYKQ
LVKAKDVVDISIKWNAWSGSGGDNYKVYFDDLLVNQGSLPAGTKSGVVQF
PYTKSGRHQLYLELCEGTVCARSAGKEIVIADTDGAHLAPLPMNVDPNNR
NNGTIPGRVTGAYFVEWGIYGRNYDVTKIPAHNLSHILYGFIPICGPNES
LKSIEIGNSWRALQTACADSQDYEVVIHDPWAAVQKSMPGVDAKDPIRGV
YSQLMALKQRYPDLKILPSVGGWTLSD PFHGFTNKANRDTFVASVKQFLK
TWKFYDGVDIDWEFPGGDGPNPDLGDPINDGPAYVALMQELRAMLDELEA
ETGRQYELTSAIGAGYDKIEDVDYQAAQQYMDYIFAMTYDFYGAWNNETG
HQTGIYCGSHLSTDECNGTGVDDNGVPRKGPAYTGDHAIQLLLQQGVQPS
KLVMGVAMYGRGWEGVLDANAAIPGNPMTAPGNGPLTGSTSEGVWEPGIM
DYKAIAANAVGQGGSGVNGYEVGYDEQA QAAYVWNRSNGKLITYDSPRSV
IAKGQYANTHQLAGLFGWEIDADNGDILNAMYDGLTAGEIPNRAPTIGVS
GPINVTSGQVVNVDAQASDLDNDPLTYSWVAAPGLALSANNTAAVAVTAP
SVAQQTSYDLTVTVNDGALSTTKTIVVVVNPEGANAAPVVTPVSDISVNE
GASATVNVSATDPEGAALSYSWSVPAELSVANGSSATITAANVTADTTVP
VTVTVSDGVNAVDTTFNVTIKDGAEYPTW DRSTVYVGGDRVIHNSNVFEA
KWWTQGEEPGTADVWKAVTN
> P27603|PHEA_PSEST P -protein [Includes: Chorismate mutase - Pseudomonas stutzeri (Pseudomonas perfectomarina).
MSEADQLKALRVRIDSLDERILDLISERARCAQEVARVKTASWPKAEEAV
FYRPEREAWVLKHIMELNKGPLDNEEMARLFREIMSSCLALEQPLRVAYL
GPEGTFSQAAALKHFGHSVISKPMAAIDEVFREVVAGAVNFGVVPVENST
EGAVNHTLDSFLEHDIVICGEVELRIHHHLLVGETTKTDRITRIYSHAQS
LAQCRKWLDAHYPNVERVAVSSNADAAKRVKSEWNSAAIAGDMAAQLYGL
SKLAEKIEDRPVNSTRFLIIGSQEVPPTGDDKTSIIVSMRNKPGALHELL
MPFHSNGIDLTRIETRPSRSGKWTYVFFIDCMGHHQDPLIKNVLEKIGHE
AVALKVLGSYPKAVL
> P0A9F9|METR_ECOLI HTH -type transcriptional regulator metR - Escherichia coli.
MIEVKHLKTLQALRNCGSLAAAAATLHQTQSALSHQFSDLEQRLGFRLFV
RKSQPLRFTPQGEILLQLANQVLPQISQALQACNEPQQTRLRIAIECHSC
IQWLTPALENFHKNWPQVEMDFKSGVTFDPQPALQQGELDLVMTSDILPR
SGLHYSPMFDYEVRLVLAPDHPLAAKTRITPEDLASETLLIYPVQRSRLD
VWRHFLQPAGVSPSLKSVDNTLLLIQMVAARMGIAALPHWVVESFERQGL
VVTKTLGEGLWSRLYAAVRDGEQRQPVTEAFIRSARNHACDHLPFVKSAE
RPTYDAPTVRPGSPARL
> P46244|SYT_BUCAP Threonyl -tRNA synthetase - Buchnera aphidicola subsp. Schizaphis graminum .
MPVIRFCDGSQQVYKHSVSLREIIENKKPNIIRSLIAISVNNSFSNFNTL
ITEDSSISFISKKDCEALNIIRYSCIQLLNYAAKKTWPSCKIGESEITKS
GFYCDIDFENSITEEDFFILENNMKTLIKREYFISHQNISFDHAYEMFKK
KSEIYKIHLIKKYINKKNKISLYYHENYFDIDMGMQVFNIKFCKYFKLQK
IGGIYWKGDHKNKMLQRIYGTAWSTKKELDKHLSYINELKKRDHRKIGKL
LNLYHMQKESPGMIFWHNNGWIIFNELEIFVREKLKEYKYKEVKTPLLID
KSIWQKSGHWDNYQDAIFTTSSENREYCIKPMNCPGHVQIFNCGLKSYRD
LPIRMAEFGSCHRNESSGSLHGLMRIRNFTQDDAHIFCTQEQLRYEINNC
IKMIYDLYSTFNFKKILVKFSTRPKKRIGDESVWDQAEKDLSDVLIENNL
KFEHQEGEGAFYGPKIEFVLQDSLDRNWQCGTIQLDFYLPIRLRSFYIDE
HNHQKIPIIIHRAILGSIERFIGILIEEFSGKLPTWLSPIQVVILSITDS
HINYVKKIVQHFSDINIRVESDLRNEKIGFKIREHTLRQIPYILICGEKE
IKSKKISVRTRNGYNLGIIDIDCFIKKLQKEIFTRSFYQMEE
> Q00964|VIUA_VIBCH Vibriobactin receptor - Vibrio cholerae.

MAVLCPARVSVAENKKFKLHTLSAMMMGLFTGSFAYAETQNTSNQEQEMP
VLVVIGEKTQRSIYETSASVEVFDQDTIERTPGATEIDDLLQLIPNLVDS
GQSNNMPTIRGIDGSGPSVGGLASFAGTSPRLNMSIDGRSLTYSEIAFGP
RSLWDMQQVEIYLGPQSYIQGRNTSAGAIVMKSNDPTHHFESAVKAGIGE
SDYSQTAGMISAPIIQDELAFRLSFDQQKRDSFVDLAAFEPAGDPKKIEM
NSVRGKLLYEPSALDGFKTTLTLSHMDSRGPQTENINVAGNEAFRPVYET
ASFTTAWDIIWHLNDLFTFENNLVYADFSYDRYTNPNSRGDFNTDGKEFH
IEPLLRYIALDGSVNTLIGARYYQSSQDDMYIDAASAYPMDGRTKAKSVF
AEVTYALTPSINVNLAGRFEREQVKRNVSHPRYKLDYDETSSVFLPKLDV
AYTPVQGQTYGIKAAKGYNASGAGLAFNSMQFTGFRPYEFEQESIWNYEF
YTRHRFSHSVEVLTNLFYNDFDSMQMTQTTSSGDVFIANLDEASTYGAEI
GSRWYATSSLELFANLGLLKTEFKETTGNTKELPRAPKMSANVGLLYDFG
QGFEFSSNAAYTGSYFSESGNSEKFAIDSYWVANAQLAYVFEHGRATLYA
TNLLDSDKTTLYLSTNNTLDQLKQQPRMIGASVQLNF
> P13430|SFAS_ECOL5 S-fimbrial adhesin protein sfaS - Escherichia coli O6:K15:H31 (strain 536 / UPEC).
MKLKAIILATGLINCIAFSAQAVDTTITVTGNVLQRTCNVPGNVDVSLGN
LYVSDFPNAGSGSPWVNFDLSLTGCQNMNTVRATFSGTADGQTYYANTGN
AGGIKIEIQDRDGSNASYHNGMFKTLNVQNNNATFNLKARAVSKGQVTPG
NISSVITVTYTYA
> P77754|SPY_ECOLI Spheroplast protein Y - Escherichia coli.
MRKLTALFVASTLALGAANLAHAADTTTAAPADAKPMMHHKG KFGPHQDM
MFKDLNLTDAQKQQIREIMKGQRDQMKRPPLEERRAMHDIIASDTFDKVK
AEAQIAKMEEQRKANMLAHMETQNKIYNILTPEQKKQFNANFEKRLTERP
AAKGKMPATAE
> P11439|TOXA_PSEAE Exotoxin A - Pseudomonas aeruginosa.
MHLTPHWIPLVASLGLLAGGSFASAAEEAFDLWNECAKACVLDLKDGVRS
SRMSVDPAIADTNGQGVLHYSMVLEGGNDALKLAIDNALSITSDGLTIRL
EGGVEPNKPVRYSYTRQARGSWSLNWLVPIGHEKPSNIKVFIHELNAGNQ
LSHMSPIYTIEMGDELLAKLARDATFFVRAHESNEMQPTLAISHAGVSVV
MAQAQPRREKRWSEWASGKVLCLLDPLDGVYNYLAQQRCNLDDTWEGKIY
RVLAGNPAKHDLDIKPTVISHRLHFPEGGSLAALTAHQACHLPLETFTRH
RQPRGWEQLEQCGYPVQRLVALYLAARLSWNQVDQVIRNALASPGSGGDL
GEAIREQPEQARLALTLAAAESERFVRQGTGNDEAGAASADVVSLTCPVA
AGECAGPADSGDALLERNYPTGAEFLGDGGDISFSTRGTQNWTVERLLQA
HRQLEERGYVFVGYHGTFLEAAQSIVFGGVRARSQDLDAIWRGFYIAGDP
ALAYGYAQDQEPDARGRIRNGALLRVYVPRSSLPGFYRTGLTLAAPEAAG
EVERLIGHPLPLRLDAITGPEEEGGRLETILGWPLAERTVVIPSAIPTDP
RNVGGDLDPSSIPDKEQAISALPDYASQPGKPPREDLK
> P13628|PHFS_DESVO Periplasmic [Fe] hydrogenase small subunit - Desulfovibrio vulgaris (strain Oxamicus Monticello).
MQIVNLTRRGFLKAACVVTGGALISIRMTGKAVAAAKQLKDYMMDRINGV
YGADAKFPVRASQDNVQVQKLYADFLEKPMSHKAEQLLHTHWVDRSKAIE
RMKAQGAYPNPRAKEFEGNTYPYE
> P27217|SCRB_KLEPN Sucrose-6-phosphate hydrolase - Klebsiella pneumoniae.
MSLPSRLPAILQAVMQGQPQALADSHYPQWHLAPVNGLLNDPNGFCQVAG
RYHLFYQWNPLACDHTYKCWGHWSSADLLHWRHEPIALMPDEEYDRNGCY
SGSAVEFEGALTLCYTGNVKFPDGGRTAWQCLATENADGTFRKLGPVLPL
PEGYTGHVRDPKVWRQDGRWYMVLGAQDVQQRGKVLLFTASDLREWRLVG
EIAGHDVNGLANAGYMWECPDLFPLADTHLLICCPQGLAREAQRFLNTYP
AVWMAGRFDAERGIFDHGPLHELDSGFEFYAPQTMQADDGRRLLVGWMGV
PDGDEMHQPTRAQGWIHQMTCVRELEWQAGTLYQRPLRELVALRGEAQGW
CGQTLPLAPMELAFDLSPDSTLGLDFAGALQLTVNRDGLRLSRRGLQTAE
MHHRYWRGEARRLRIFIDRSSVEIFINDGEGVMSSRFFPGYPGQLIFSGA
TPVAFCRWLLRPCMVE
> P15644|GSPD_KLEPN General secretion pathway protein D - Klebsiella pneumoniae.
MIIANVIRSFSLTLLIFAALLFRPAAAEEFSASFKGTDIQEFINTVSKNL
NKTVIIDPSVRGTITVRSYDMLNEEQYYQFFLSVLDVYGFAVINMNNGVL
KVVRSKDAKTAAVPVASDAAPGIGDEVVTRVVPLTNVAARDLAPLLRQLN
DNAGVGSVVHYEPSNVLLMTGRAAVIKRLLTIVERVDNAGDRSVVTVPLS
WASAADVVKLVTELNKDTSKSALPGSMVANVVADERTNAVLVSGEPNSRQ
RIIAMIKQLDRQQATQGNTKVIYLKYAKASDLVEVLTGISSTMQSEKQAA

KPVAALDKNIIIKAHGQTNALIVTAAPDVMNDLE RVIAQLDIRRPQVLVE
AIIAEVQDADGLNLGIQWANKNAGMTQFTNSGLPISTAIAGANQYNKDGT
VSSSLASALSSFNGIAAGFYQGNWAMLLTALSSSTKNDILATPSIVTLDN
MEATFNVGQEVPVLTGSQTTSGDNIFNTVERKTVGIKLKVKPQINEGDSV
LLEIEQEVSSVADAASSTSSDLGATFNTRTVNNAVLVGSGETVVVGGLLD
KSVSDTADKVPLLGDIPVIGALFRSTSKKVSKRNL MLFIRPTVIRDRDEY
RQASSGQYTAFNDAQSKQRGKENNDAMLNQDLLEIYPRQDTAAFRQVSAA
IDAFNLGGNL
> P46445|CYC6_SYNY3 Cytochrome c6 - Synechocystis sp. (strain PCC 6803).
MFKLFNQASRIFFGIALPCLIFLGGIFSLGNTALAADLAHGKAIFAGNCA
ACHNGGLNAINPSKTLKMADLEANGKNSVAAIVAQITNGNGAMPGFKGRI
SDSDMEDVAAYVLDQAEKGW
> P94845|GLNA_HELPY Glutamine synthetase - Helicobacter pylori
(Campylobacter pylori).
MIVRTQNSESKIKEFFEFCKENEVEFVDFRFSDIKGTWNHIAYSFGALTH
GMLKEGIPFDASCFKGWQGIEHSDMILTPDLVRYFIDPFSADVSVVVFCD
VYDVYKNQPYEKCPRSIAKKALQHLKDSGLGDVAYFGAENEFFIFDSIK I
KDASNSQYYEVDSEEGEWNRDRSFENGVNFGHRPGKQGGYMPVPPTDTMM
DIRTEIVKVLNQVGLETFVVHHEVAQAQGEVGVKFGDLVEAADNVQKLKY
VVKMVAHLNGKTATFMPKPLYGDNGSGMHTHVSVWKNNENLFSGETYKGL
SEFALHFLGGVLRHARGLAAFTNASTNSYKRLIPGYEAPSILTYSANNRS
ASVRIPYGISKNSARFEFRFPDSSSNPYLAFAAILMAGMDGVKNKIDPGE
AMDINLFKLTLDEIREKGIKQMPHTLRRSLEEMLADKQYLKESQVFSEEF
IQAYQSLKFNAEVFPWESKPHPFEFITTYSC
> KDSA_HAEIN P45251 2-DEHYDRO-3-DEOXYPHOSPHOOCTONATE ALDOLASE
MQNKIVKIGNIDVANDKPFVLFGGMNVLESRDMAMQVCEAYVKVTEKLGV
PYVFKASFDKANRSSIHSYRGPGMEEGLKIFQELKDTFGVKIITDVHEIY
QCQPVADVVDIIQLPAFLARQTDLVEAMAKTGAVINVKKPQFLSPSQMGN
IVEKIEECGNDKIILCDRGTNFGYDNLIVDMLGFSVMKKASKGSPVIFDV
THSLQCRDPFGAASSGRRAQVTELARSGLAVGIAGLFLEAHPNPNQAKCD
GPSALPLSALEGFVSQMKAIDDLVKSFPELDTSI
> Q04681|PMFA_PROMI Major fimbrial subunit - Proteus mirabilis.
MKLSKIALAAAL VFGINSVATAENETPAPKVSSTKGEIQLKGEIVNSACG
LAASSSPVIVDFSEIPTSALANLQKAGNIKKDIELQDCDTTVAKTATVSY
TPSVVNAVNKDLASFVSGNASGAGIGLMDAGSKAVKWNTATTPVQLINGV
SKIPFVAYVQAESADAKVTPGEFQAVINFQVDYQ
> P23135|CY1_RHORU Cytochrome c1 - Rhodospirillum rubrum.
MTTIVKRALVAAGMVLAIGGAA QANEGGVSLHKQDWSWKGIFGRYDQPQL
QRGFQVFHEVCSTCHGMKRVAYRNLSALGFSEDGIKELAAEKEFPAGPDD
NGDMFTRPGTPADHIPSPFANDKAAAAANGGAAPPDLSLLAKARPGGPNY
IYSLLEGYASDSPGEPAEWWVKQQQEKGLEVAFNEAKYFNDYFPGHAISM
PPPLMDDLITYEDGTAATKDQMAQDVVAYLNWAAEPELDARKSLGLKVLL
FLGVLTAMLLALKLAIWRDVKH
> P10858|YADA_YERPS Adhesin yadA - Yersinia pseudotuberculosis.
MTKDFKISVSAALISALFSSPYAFAEEPEDGNDGIPRLSAVQISPNVDPK
LGVGLYPAKPILRQENPKLPPRGPQGPEKKRARLAEAIQPQVLGGLDARA
KGIHSIAIGATAEAAKPAAVAVGAGSIATGVNSVAIGPLSKALGDSAVTY
GASSTAQKDGVAIGARASASDTGVAVGFNSKVDAQNSVA IGHSSHVAADH
GYSIAIGDLSKTDRENSVSIGHESLNRQLTHLAAGTKDNDAVNVAQLKKE
MAETLENARKETLAQSNDVLDAAKKHSNSVARTTLETAEEHANKKSAEAL
VSAKVYADSNSSHTLKTANSYTDVTVSSSTKKAISESNQYTDHKFSQLDN
RLDKLDKRVDKGLASSAALNSLFQPYGVGKVNFTAGVGGYRSSQALAIGS
GYRVNESVALKAGVAYAGSSNVMYNASFNIEW
> P00083|CYC2_RHOVI Cytochrome c2 - Rhodopseudomonas viridis.
MRKLVFGLFVLAASVAPAAAQDAASGEQVFKQCLVCHSIGPGAKNKVGPV
LNGLFGRHSGTIEGFAYSDANKNSGITWTEEVFREYIRDPKAKIPGTKMI
FAGVKDEQKVSDLIAYIKQFNADGSKK
> P09378|RHAR_ECOLI HTH-type transcriptional activator rhaR - Escherichia
coli.
MAFCNNANLLNVFVRHIANNQLRSLAEVATVAHQLKLLKDDFFASDQQAV
AVADRYPQDVFAEHTHDFCELVIVWRGNGLHVLNDRPYRITRGDLFYIHA
DDKHSYASVNDLVLQNIIYCPERLKLNLDWQGAIPGFNASAGQPHWRLGS
MGMAQARQVIGQLEHESSQHVPFANEMAELLFGQLVMLLNRHRYTSDSLP

PTSSETLLDKLITRLAASLKSPFALDKFCDEASCSERVLRQQF RQQTGMT
INQYLRQVRVCHAQYLLQHSRLLISDISTECGFEDSNYFSVVFTRETGMT
PSQWRHLNSQKD
> Q05202|FCUA_YEREN Ferrichrome receptor fcuA - Yersinia enterocolitica.
MNQTISSRAPQKRLAPRLLCVMIGAALGTLSASSWAAAATDSTAENAKKT
SATAATAKAEDSKTNDTITVVGAQETFRAGGNDLIPTYLDGQVANGGRIG
FLGQQDARNVPFNVIGYTSKMIEDQQANSIADVVKNDASVQNVRGYGNPS
QNYRIRGYNLDGDDISFGGLFGVLPRQIVSTSMVERVEVFKGANAFINGI
SPSGSGVGGMINLEPKRAGDTPLTRVTVDYGSASQVGGALDVGRRYGDDD
QFGVRVNVLHREGESAIHDQKERTTAVSTGLDYRGDRARTSLDVGYQKQT
IHHMRTDVAIGGATVIPEPPSSTLNYGQSWVYTDMETTFGMLRSEYDVSQ
NWTVYGSVGA SRNEETGQYGAPMLTNNNGDATISRLYVPYVADSVAGLGG
IRGHFDTGPITHKVNLGYAANYRTTKSAWNMSGQEDTNIYNPGVIGFPQT
VMGSDSQDPQLTSQVRASGLSLSDTLSMMDDKVSLMLGVRRQEVTIRNFD
SGVPNSAGSLDAMKVTPIYGIMVKPWEKVSLYANHIEALGPGKSAPYQYN
GKPVVNAGQIPGIIHSKQNEIGVKFDNQRYGGTLALFEITRPTGMVDPAT
NVYGFYGEQRN RGIELNVFGEPVFGTRLLASATWLDPKLTKAADSANNGN
DAVGVANYQLVFGGEYDIPVVEGLTATGTVVRSGSQYANEANTLKLKPWT
RLDLGVRYTMPMKDTSLTWRANIENVTNERYWESVEDSGTYIYQGDPRAL
KLSVSMDF
> P00101|CY551_PSEST Cytochrome c -551 - Pseudomonas stutzeri (Pseudomonas perfectomarina).
MKKILIPMLALGGA LAMQPALAQDGEALFKSKPCAACHSVDTKMVGPALK
EVAAKNAGVEGAADTLALHIKNGSQGVWGPIPMPPNPVTEEEAKILAEWV
LSLK
> P00935|METB_ECOLI Cystathionine gamma -synthase - Escherichia coli.
MTRKQATIAVRSGLNDDEQYGCVVPPIHLSSTYNFTGFNEPRAHDYSRRG
NPTRDVVQRALAELEGGAGAVLTNTGMSAIHLVTTVFLKPGDL LVAPHDC
YGGSYRLFDSLAKRGCYRVLFVDQGDEQALRAALAEKPKLVLVESPSNPL
LRVVDIAKICHLAREVGAVSVVDNTFLSPALQNPLALGADLVLHSCTKYL
NGHSDVVAGVVIAKDPDVVTELAWWANNIGVTGGAFDSYLLLRGLRTLVP
RMELAQRNAQAIVKYLQTQPLVKKLYHPSLPENQGHEIAARQQKGFGAML
SFELDGDEQTLRRFLGGLSLFTLAESLGGVESLISHAATMTHAG MAPEAR
AAAGISETLLRISTGIEDGEDLIADLENGFRAANKG
> P74941|SYA_THET8 Alanyl -tRNA synthetase - Thermus thermophilus (strain HB8 / ATCC 27634 / DSM 579).
MRTAEIREKFLSFFEGKGHLRLPSFSLIPEDDPSLLFTSAGMAPLKPYFL
GAKPIFGGREWRRVTTCQECLRVGDIENVGRTSRHNTYFEMLGNFSFGDY
FKKEAILWAWEFLTEHLKLDPGRLWVTVFEDDDEAYEIWRDLVGVPEERI
GRFGEDENYWPGGAITHGPNGPSGPCSEIFYDRGPAYGTPDETGPNTGSG
DRFVEIWNLVFTQYDRQGPIPGPGILKPLPQKNIDTGMGLYRVAAILQDV
EDFYRTDTFFPIIQEVARMSGRPYEGKTSVSHRVIADHVRAVVAALSDGA
TFSNTGRGYVIRRLLRRALRHGYLLGLSDPFLHRLAPLVAELLGDFYPEM
RENLPAVEKQIRLEEERFLETLEGGLKRLDALLSGLKPGEVLPGKEAFRL
YDTYGFPLDLTVEIAAERGYGVDTEGFQKAMEEQQSRSRAAMAFEREIFK
KGAQVLEELYAERGATEFLGYNALEAEAEVLALLAGDQSLLEAGPGTEVQ
VVLDKTPFYAEGGGQIGDFGLLEWPGGRARVETTRKTERGIFLHKARVEE
GVLRVGERVRAVVDPRRRDTERNHTATHLLHAALRAVLGPHVRQAGSLVA
PDRLRFDFTH PEPLKPEELERVELLVNRWIMADFPVTWRYMPLEEARKEG
AMALFGEKYGEVVRVVRVEGSPLEGLESKELCGGCHVRRTGEIGAFLIRS
EEAVSAGVRRIEAVTGEEAIRFARGSLNRLKALAERLEVGEAALEERLEK
LLAELKEKEREVESLKARLVQAALGGGGGASLEEKGGLRWTVAELPGLDA
KALRQAADDLVARGADVALVLSGGQAVLKLSPKAQGMGLEAGALFRALAE
KAGGRGGGKGA LAQGGGLDPRKAREALPGLLP
> P15452|CY552_HYDTH Cytochrome c -552 - Hydrogenobacter thermophilus.
MKKFLLVAVVGLAGITFANEQLAKQKGCMACHDLKAKKVGPAYADVAKKY
AGRKDAVDYLAGKIKKGGSGVWGSVPMPPQNVTDAEAKQLAQWILSIK
> P0AD60|IVY_ECO57 Inhibitor of vertebrate lysozyme - Escherichia coli O157:H7.
MGRISSGGMMFKAITTVAALVIATSAMAQDDLTISSLAKGETTKAAFNQM
VQGHKLPAWVMKGGTYTPAQTVTLGDETYQVMSACKPHDCGSQRIAVMWS
EKSNQMTGLFSTIDEKTSQEKLTWLNVNDALSIDGKTVLFAALTGSLENH
PDGFNFK

> P42517|CHMU_ENTAG Monofunctional chorismate mutase - Enterobacter agglomerans (Erwinia herbicola) (Pantoea agglomerans).
MTHFVAIFFSSLFMCSNVFAGSVSSVSLGSLSSALNERMQVMKAVAGYKA
LHHLPIEDLPREQVVLDHMLQNAQQAGLEPHSVEPFVHALMNASKTIQYR
YRADWLSSPDSAVPVRDLTETRQQIQQLDTQLLTAISQRLMTGAFSQEDK
EFLMSHLTAPHLSESDKNSLFASLSRIQRQH
> AMY2_SALTY P26613 CYTOPLA SMIC ALPHA-AMYLASE (EC 3.2.1.1) (1
MKNPTLLQYFHWYYPDGGKLWSELAERADGLNDIGINMVWLPPACKGASG
GYSVGYDTYDLFDLGEFDQKGTIATKYGDKRQLLTAIDALKKNNIAVLLD
VVVNHKMGADEKERIRVQRVNQDDRTQIDDNIIECEGWTRYTFPARAGQY
SNFIWDYHCFSGIDHIENPDEDGIFKIVNDYTGDGWNDQVDDEMGNFDYL
MGENIDFRNHAVTEEIK YWARWVMEQTHCDGFRLDAVKHIPAWFYKEWIE
HVQAVAPKPLFIVAEYWSHEVDKLQTYIDQVDGKTMLFDAPLQMKFHEAS
RQGAEYDMRHIFTGTLVEADPFHAVTLVANHDTQPLQALEAPVEPWFKPL
AYALILLRENGVPSVFYPDLYGASYEDSGENGETCRVDMPVINQLDRLIL
ARQRFAHGIQTLFFDHPNCIAFSRSGTEENPGCVVVLSNGDDGEKTLLLG
DNYANKTWRDFSGNRDEY VVTNDQGEATFFCNAGSVSVWVIEDV
> P20862|FANH_ECOLI Protein fanH - Escherichia coli.
MIKKVPVLLFFMASISITHASQTATKSLGVSITLSKAQCKINNRAGISGS
FVLPMISTSGQIISSKKFTTVPIIIDCTAGGNVNQLEITFGDNSSKKIDS
TTWYTTNKDLGLRFSWTKDKTQGFNLGVAHNINKSIWLEGNKKFNASVDV
SPVVIRNTVQGGQYTSALPVTVT FI
> P0A9E0|ARAC_ECOLI Arabinose operon regulatory protein - Escherichia coli.
MAEAQNDPLLPGYSFNAHLVAGLTPIEANGYLDFFIDRPLGMKGYILNLT
IRGQGVVKNQGREFVCRPGDILLFPPGEIHHYGRHPEAREWYHQWVYFRP
RAYWHEWLNWPSIFANTGFFRPDEAHQPHFSDLFGQIINAGQGEGRYSEL
LAINLLEQLLLRRMEAINESLHPP MDNRVREACQYISDHLADSNFDIASV
AQHVCLSPSRLSHLFRQQLGISVLSWREDQRISQAKLLLSTTRMPIATVG
RNVGFDDQLYFSRVFKKCTGASPSEFRAGCEEKVNDVAVKLS
> P37902|GLTI_ECOLI Glutamate/aspartate periplasmic -binding protein - Escherichia coli.
MQLRKPATAILALALSAGLAQADDAAPAAGSTLDKIAKNGVIVVGHR ESS
VPFSYYDNQQKVVGYSQDYSNAIVEAVKKKLNKPDLQVKLIPITSQNRIP
LLQNGTFDFECGSTTNNVERQKQAAFSDTIFVVGTRLLTKKGGDIKDFAN
LKDKAVVVTSGTTSEVLLNKLNEEQKMNMRIISAKDHGDSFRTLESGRAV
AFMMDDALLAGERAKAKKPDNWEIVGKPQSQEAYGCMLRKDDPQFKKLMD
DTIAQVQTSGEAEKWFDKWFKNPIPPKNLNMNFELSDEMKALFKEPND KA
LN
> P04960|PELE1_ERWCH Pectate lyase E - Erwinia chrysanthemi.
MKNTRVRSIGTKSLLAAVVTAALMATSAYAAVETDAATTGWATQNGGTTG
GAKAAKAVEVKNISDFKKALNGTDSSAKIIKVTGPIDISGGKAYTSFDDQ
KARSQISIPSNTTIIGVGSNGKFTNGSLVIKGVKNVILRNLYIETPVDVA
PHYESGDGWNAEWDAAVIDNSTNVWVDHVTISDGSF TDDKYTTKDGEKYV
QHDGALDIKKGSDYVTISYSRFELHDKTILIGHSDSNGSQDSGKLRVTFH
NNVFDRVTERAPRVRFGSIHAYNNVYLGDVKHSVYPYLYSFGLGTSGSIL
SESNSFTLSNLKSIDGKNPECSIVKQFNSKVFSDKGSLVNGSTTTKLDTC
GLTAYKPTLPYKYSAQTMTSSLATSINNNAGYGKL
> P36267|GGT_PSEUA Gamma-glutamyltranspeptidase - Pseudomonas sp. (strain A14).
MKNQTFSKALLATALSCALFNVHAASQAPVGAENGMVVTAQHIASKVGVE
VLKSGGNAIDAAVAVGYALAVVYPAAGNIGGGGFMTIQLADGRKTFLDFR
EKAPLAATANMYLDKDGNVIKGASTTGYLAVGVPGTVSGMEYAREKYGTK
TRQQLISPAITLADKGFVLEQGDVDMLWTSTKDFEKDRANSGAIFMNKGQ
PFQPGERLVQKDLARTLRLISAKG TDGFYKGEVADKLVASMKAGGGIITQ
ADLDQYKTRELAPVECDYRGYHVVSAPPPSSGGVVICEIMNILEGYPMKE
LGYHSAQGVHYTIEAMRHAYVDRNSYLGDPDFVKNPLAHLLDKDYAAKIR
AAINPQKAGISQEIKPGVPPHEGSNTTHYSIVDKDGNAVSVTYTLNDWFG
AKVMANGTGVLLNDEMDDFTSKVGVPNMYGLIQGEANAIGPGRRPLSSMS
PTIVTKDGKTVMVVGTPGGSRIITA TLLTMLNMIDYGMNLQEAVDAPRFH
QQWMPESTNIEAFALSPDTQKILESWGQKFAGPQPANHIAAILVGAPSLG
GKPIGKNRFYGANDPRRNTGLALGY
> FUMC_BRAJA P28894 FUMARATE HYDRATASE C (EC 4.2.1.2) (FUMARA
MKRPRGISRGGMDVLMAKSARTKTARPATRTETDSFGPIEVPSDRYWGAQ

TERSRQNFRIGTDRMPISLVHALGIVKLAAAQSNRELGLL DQRRASAIIR
AAREVSTADLDDHFPLVVWQTGSGTQTNMNLNEVIANRANELLGGELGAK
KPVHPNDHVNMSQSSNDSFPTAMHIAAASRITADLVPALGELLRALRKKE
KEFAKIVKIGRTHTQDATPLTLGQEFSGYAAQVERGIARLKVAVKELYPL
AQGGTAVGTGLNAKPRFARLFAKHVAGITKLPFTSAANKFEALASNDAYV
LAHGAISSVATGLFKIANDIRLLGSGPRSGLGELILPENEP GSSIMPGKV
NPTQCEAMTMVCCQVFGNHTAITVAGSQGHFELNVYKPVLAYNMLHSIRL
MADAARSFTEHCVSGIRADEKRISELMQRSLMLVTALAPKIGYDNAAKVA
KTAHANGTTLKEEALRLGFVTADEFDRLVQPEKMTKPG
> Q03011|MRPA_PROMI Major MR/P fimbria protein - Proteus mirabilis.
MKLNKLALVLGLGLSVVAGSALAADQGHGTVKFVGSI IDAPCSITPDTEN
QTVPLGQISTAALKDGGRSNSRDFKISLENCTTETYKTVQTTFTGSEATE
VLEGSLGIEGIAKNAAVVITDAGGKQIKLGTPSAAQNLRDGNNDLNFAAY
LQGSASEAAVPGDFTAIATFALTYQ
> P45605|PHOB_KLEPN Phosphate regulon transcriptional regulatory protein phoB - Klebsiella pneumoniae.
MARRILVVEDE APIREMVCFVLEQNGFQPVEAEDYDSAVNQLNEPWPDLI
LLDWMLPGGSGLQFIKLLKREAMTRDIPVVMLTARGEEEDRVRGLETGAD
DYITKPFSPKELVARIKAVMRRISPMAVEEVIEMQGLSLDPSSHRVMTGD
SPLDMGPTEFKLLHFFMTHPERVYSREQLLNHVWGTNVYVEDRTVDVHIR
RLRKALEHSGHDRMVQTVRGTGYRFSARF
> P0A0Y4|FBPA_NEIMB Major ferric iron-binding protein - Neisseria meningitidis serogroup B.
MKTSIRYALLAAALTAATPALADITVYNGQHKEAAQAVADAFTRATGIKV
KLNSAKGDQLAGQIKEEGSRSPADVFYSEQIPALATLSAANLLEPLPAST
INETRGKGVPVAAKKDWVALSGRSRVVVYDTRKLSEKDLEKSVLNYATPK
WKNRIGYAPTSGAFLEQVVAIVKLKGEAAALKWLKGLKEYGKPY AKNSVA
LQAVENGEIDAALINNYYWHAFAREKGVQNVHTRLNFVRHRDPGALVTYS
GAAVLKSSQNKDEAKKFVAFLASKEGQRALTAVRAEYPLNPHVVSTFNLE
PIAKLEAPQVSATTVSEKEHATRLLEQAGMK
> Q06006|NIR_PSECL Copper -containing nitrite reductase - Pseudomonas chlororaphis (Pseudomonas aureofaciens).
MSVFRSVLGACVLLGSCASSLALAGGAEGLQRVKVDLVAPPLVHPHEQVV
SGPPKVVQFRMSIEEKKMVIDDQGTTLQAMTFNGSMPGPTLVVHEGDYIE
LTLVNPATNSMPHNVDFHAATGALGGAGLTQVVPGQEVVLRFKADRSGTF
VYHCAPQGMVPWHVVSGMNGALMVLPRDGLRDPQGKLLHYDRVYTIGESD
LYIPKDKDGHYKDYPDLASSYQDTRAVMRTLTPSHVVFNGRVGALTGANA
LTSKVGESVLFIHSQANRDSRPHLIGGHGDWVWTTGKFANPPQRNMETWF
IPGGSAVAALYTFKQPGTYVYLSHNLIEAMELGALAQIKVEGQWDDDLMT
QVKAPGPIVEPKQ
> P0AEE5|DGAL_ECOLI D -galactose-binding periplasmic protein - Escherichia coli.
MNKKVLTLSAVMASMLFGAAAHAADTRIGVTIYKYDDNFMSVVRKAIEQD
AKAAPDVQLLMNDSQ NDQSKQNDQIDVLLAKGVKALAINLVDPAAAGTVI
EKARGQNVPVVFFNKEPSRKALDSYDKAYYVGTDSKESGIIQGDLIAKHW
AANQGWDLNKDGQIQFVLLKGEPGHPDAEARTTYVIKELNDKGIKTEQLQ
LDTAMWDTAQAKDKMDAWLSGPNANKIEVVIANNDAMAMGAVEALKAHNK
SSIPVFGVDALPEALALVKSGALAGTVLNDANNQAKATFDLAKNLADGKG
AADGTNWKIDNKVVRV PYVGVDKDNLAEFSKK
> P44715|PTFAH_HAEIN Multiphosphoryl transfer protein - Haemophilus influenzae.
MLELSESNIHLNANAIDKQQAIEMAVSALVQAGNVENGYLQGMLARELQT
STFLGNGIAIPHGTLDTRLMVKKTGVQVFQFPQGIEWGEGNIAYVVIGIA
ARSDEHLSLLRQLTHVLSDEDTAAKLAKITDVAEFCAILMGETIDPFEIP
AANISLDVNTQSLLTLVAINAGQLQVQSAVENRFISEVINNAALPLGKGL
WVTDSVVGNVKNALAFSRAKTIFSHNGKAVKGVITVSAVGDQINPTLVRL
LDDDVQTTLLNGNSTEILTALLGSSSDVETQSVEGAVVGTFTIRNEHGLH
ARPSANLVNEVKKFTSKITMQNLTRESEVVSAKSLMKIVALGVTQGHRLR
FVAEGEDAKQAIESLGKAIANGLGENVSAVPPSEPDTIEIMGDQIHTPAV
TEDDNLPANAIEAVFVIKNEQGLHARPSAILVNEVKKYNASVAVQNLDRN
SQLVSAKSLMKIVALGVVKGTRLRFVATGEEAQQAIDGIGAVIESGLGE
> P13036|FECA_ECOLI Iron(III) dicitrate transport protein fecA - Escherichia coli.

MTPLRVFRKTTPLVNTIRLSLLPLAGLSFSAFAAQVNIAPGSLDKALNQY
AAHSGFTLSVDASLTRGKQSNGLHGDYD VESGLQQLLDGSGLQVKPLGNN
SWTLEPAPAPKEDALTVVGDWLGDARENDVFEHAGARDVIRREDFAKTGA
TTMREVLNRIPGVSAPENNGTGSHDLAMNFGIRGLNPRLASRSTVLMDGI
PVPFAPYGQPQLSLAPVSLGNMDAIDVVRGGGAVRYGPQSVGGVVNFVTR
AIPQDFGIEAGVEGQLSPTSSQNNPKETHNLMVGGTADNGFGTALLYSGT
RGSDWREHSATRIDDLMLKSKYAPDEVHT FNSLLQYYDGEADMPGGLSRA
DYDADRWQSTRPYDRFWGRRKLASLGYQFQPDSQHKFNIQGFYTQTLRSG
YLEQGKRITLSPRNYWVRGIEPRYSQIFMIGPSAHEVGVGYRYLNESTHE
MRYYTATSSGQLPSGSSPYDRDTRSGTEAHAWYLDDKIDIGNWTITPGMR
FEHIESYQNNAITGTHEEVSYNAPLPALNVLYHLTDSWNLYANTEGSFGT
VQYSQIGKAVQSGNVEPEKARTWELGTRYD DGALTAEMGLFLINFNNQYD
SNQTNDTVTARGKTRHTGLETQARYDLGTLTPTLDNVSIYASYAYVNAEI
REKGDTYGNLVPFSPKHKGTLGVDYKPGNWTFNLNSDFQSSQFADNANTV
KESADGSTGRIPGFMLWGARVAYDFGPQMADLNLAFGVKNIFDQDYFIRS
YDDNNKGIYAGQPRTLYMQGSLKF
> P35823|SLAP_AERSA S -layer protein - Aeromonas salmonici da.
MFKKTLIAAAIVVGSAAPAFADVVISPNDNTFVTTSLASVTKQPVLDFST
AQQNLTLNFSEVGDLKNNGFIVLEIQGEGQFNDAEIRQWLSNGFWRRPFT
GLLVNPNDHGNFANSGEVNDVRKFFKIISDGTQLTIVHTIDSNGKRLRLA
LASDVEETINFADAEVELKLNLANQAFKLTSGSQGTVALTAGALWNASYT
ADPVATKPLFKLGKLFQLSLTNAGKATALVSEGFLKLNIGDANISATD FA
ITNVTTNQTIQRDKVNLTLTGDVSAFKKDANGNLVNKAGASIGWKAAADG
QSATAVLGAGNMAGGVQNALAAFGTLYVAADNTVPVPAVNFNVKAEIQGD
SQATYNYFKDELADLFILTRDGMKFDTITTGTTSANLIHIRDVSNILPTE
GGKIFVTITEYADHAANGRGEGTVLVTRKALSVTLPSGGAVTLKPADVAA
DVGASITAGRQARLVFEVETNQGEVAVKKSNAEGVDIQNGTRGTAPLVD F
TL
> HXKG_ECOLI P46880 GLUCOKINASE (EC 2.7.1.2) (GLUCOSE KINASE)
MTKYALVGDVGGTNARLALCDIASGEISQAKTYSGLDYPSLEAVIRVYLE
EHKVEVKDGCIAIACPITGDWVAMTNHTWAFSIAEMKKNLGFSHLEIIND
FTAVSMANPMLKKEHLIQFGGAEPVEGKPIAVYGAGTGLGVAHLVHVDKR
WVSLPGEGGHVDFAPNSEEEAIILEILRAEIGHVSA EACLSGPGLVNLYR
AIVKADNRLPENLKPKDITERALADSCTDCRRALSLFCVIMGRFGGNLAL
NLGTFGGVFIAGGIVPRFLEFFKGSGFRAAFEDKGRFKEYVHDIPVYLIV
HDNPGLLGSGAHLRQTLGHIL
> P73443|SYK_SYNY3 Lysyl -tRNA synthetase - Synechocystis sp. (strain PCC 6803).
MADSHSHSSLEEIRATRLEKAEQLRQLGLNPYAYTWE ITHQAQDLQETYR
DLSNGEEVDLKVAIAGRILARRVMGKLAFFTLQDESGTIQLYLEKQRLTE
HMPELENAFNLLKKITDVGDILGVTGTLKRTEKGELSVYVQTYAVLTKSL
LPLPDKWHGLTDTEKRYRQRYVDLIVNPTVRQTFRRRAQITAAIRRYLDK
QGFIEIETPVLQGESGGAEARPFITHHNTLGMPLYLRIATELHLKRLVVG
GFEKVFELGRIFRNEGVSTRHNPEFTSIEVYQAYVDYN EMMALTEALVTT
AAQAVLGTLKITYQGEEIDLTPPWKRITMHEAVQLETGIDFSQFTDLETA
KQAATKAGIGVPEDCPSLGHLLNHAFEQKVEGTLMQPTFIIDFPVEISPL
AKPHRSKPGLVERFELFVYGRELANSFSELTDPIDQRSRLEAQAAKKAAG
DLEAHSVDEDFLTALEYGMPPTGGLGIGIDRLVMLLTDSPSIRDVIAFPL
LKNQEAGSDG
> P04172|AMCY_METEX Amicyani n-alpha - Methylobacterium extorquens (Protomonas extorquens).
MRALAFAAALAAFSATAALAAGALEAVQEAPAGSTEVKIAKMKFQTPEVR
IKAGSAVTWTNTEALPHNVHFKSGPGVEKDVEGPMLRSNQTYSVKFNAPG
TYDYICTPHPFMKGKVVVE
> P43763|PROB_HAEIN Glutamate 5 -kinase - Haemophilus influenzae.
MNKKTIVVKFGTSTLTQGSPKLNSPHMMEIVRQIAQLHNDGFRIVIVTSG
AIAAGRHYLNHPQLPPTIASKQLLAAVGQSQLIQAWEKLFAIYDIHIGQL
LLTRADIEDRERFLNARDTLYALLDNHIIPVINENDAVATAEIKVGDNDN
LSALVAILVQAEQLYLLTDQQGLFDSDPRKNPEAKLIPVVEQITDHIRSI
AGGSGTNLGTGGMMTKIIAADVATRSGIETIIAPGNRPNVIADLAYEQNI
GTKFIAHQSDRLESRKQWLFAAPSAGIITIDNGAQNAILEQNKSLLPAGI
INVEGRFSRGEVVKIRTQSGKDIALGMPRYNSDALQLIKGRKSADIENVL
GYEYGAVAMHRDDMIILS

> P0AGD1|SODC_ECOLI Superoxide dismutase [Cu -Zn] - Escherichia coli.
MKRFSLAILALVVATGAQAASEKVEMNLVTSQGVGQSIGSVTITETDKGL
EFSPDLKALPPGEHGFHIHAKG SCQPATKDGKASAAESAGGHLDPQNTGK
HEGPEGAGHLGDLPALVVNNDGKATDAVIAPRLKSLDEIKDKALMVHVGG
DNMSDQPKPLGGGGERYACGVIK
> P51837|RNC_COXBU Ribonuclease III - Coxiella burnetii.
MNHLNKLMERLGHQFNNLELLKIALTHCSSGADNNERLEFLGDSVLGFII
ASELYQRRPQAREGDLSRMRASMVNGDELAQMSTKLGINEYLQL GVGEQK
SGGKRRRSILADALEAIVGAIYIDAGLETCRRCVLNWYGERVDDLSKLSP
KKDAKSLLQEWLQARRLPLPTYEVKITGEAHAQTFTVNCYVKGLPHKTEG
VNTTRRRAEQIAAKRFLELLDDGKGDGITERDQ
> P04977|TOX1_BORPE Pertussis toxin subunit 1 - Bordetella pertussis.
MRCTRAIRQTARTGWLTWLAILAVTAPVTSPAWADDPPATVYR YDSRPPE
DVFQNGFTAWGNNDNVLDHLTGRSCQVGSSNSAFVSTSSSRRYTEVYLEH
RMQEAVEAERAGRGTGHFIGYIYEVRADNNFYGAASSYFEYVDTYGDNAG
RILAGALATYQSEYLAHRRIPPENIRRVTRVYHNGITGETTTTEYSNARY
VSQQTRANPNPYTSRRSVASIVGTLVRMAPVIGACMARQAESSEAMAAWS
ERAGEAMVLVYYESIAYSF
> Q02760|CY1_RHOSH Cytoc hrome c1 - Rhodobacter sphaeroides
(Rhodopseudomonas sphaeroides).
MIRKLTLTAATALALSGGAAMAAGGGHVEDVPFSFEGPFGTFDQHQLQRG
LQVYTEVCAACHGMKFVPIRSLSEPGGPELPEDQVRAYATQFTVTDEETG
EDREGKPTDHFPHSALENAADLSLMAKARAGFHGPMGTGISQLFNGIGGP
EYIYSVLTGFPEEPPKCAEGHEPDGFYYNRAFQNGS VPDTCKDANGVKTT
AGSWIAMPPPLMDDLVEYADGHDASVHAMAEDVSAFLMWAAEPKLMARKQ
AGFTAVMFLTVLSVLLYLTNKRLWAGVKGKKKTNV
> P44305|RIMI_HAEIN Ribosomal -protein-alanine acetyltransferase -
Haemophilus influenzae.
MSIISQIEACDFERLYEIEQQAHLVPWSFGTLKNNQGERYLNLKLIENNQ
IIGFAICQTVLDE ATLFNIAILPTYQGCGFGKLLLGKLIFQLKEKGVQTL
WLEVRESNSARFLYEKIGFNEVDIRKNYYPKPSGGRENAVVMACYL
> GLN1_RHILV P09826 GLUTAMINE SYNTHETASE I (EC 6.3.1.2) (GLUT
MATASEILKQIKENDVKFVDLRFTDRRASLQHVTMDVVCVDEDMFADGVM
FDGFSIGGWKAINESDMVLMPDTETVHMDPFFAQSTMVIVCDILDPVSGE
AYNRDRRGTAKKAEAYLKASGIGDTVFVGREAEFFVFDDVKYKADPYNTG
FKLDSTELPSNDDTDYETGNLGHRPRVKGGYFPVPPVDSAQDMRSEMLTV
LSEMGVVVEKHHHEVAAAQHDTLVRNADELGIKFKMQIYKYVVHQVANAY
GKTATFMPKPIFGDNGSGMHVHQSIWKGGKPTFAGDEYAGLSESCLFYIG
GIIKHAKAINAFTNPSTNSYKRFVPGYEAPVLLAYSARNRSASCRIPFGS
NPKAKRVE VRFPDPTANPYLAFAAMLMAGLDGIKNKIHPGKAMDKDLYDL
PPKELKKIPTVCGSLRQALESLDKDRKFLTAGGVFDDDQIDAFIELKMAE
VMRFEMTPHPVEYDMYYSA
> APT_PSEAE Q04633 PROBABLE ADENINE PHOSPHORIBOSYLTRANSFERASE
MIFDEFTLKSQIRAVPDFPKPGVVFRDITPLFQSPRALRMTVDSFVQRYI
EADFSHIGAMDARGFLIGSAVAYALNKPL VLFRKQGKLPADVLAEGYQTE
YGEAFLEVHADSLCEGDSVLIFDDLIATGGTLLAAASLVRRLGARVFEAA
AIIDLPELGGSTRLQDAGISTFSLTAFALDER
> Q07408|MSP4_ANAMA Major surface antigen 4 - Anaplasma marginale.
MNYRELFTGGLSAATVCACSLLVSGAVVASPMSHEVASEGGVMGGSFYVG
AAYSPAFPSVTSFDMRESSKETSYVRGYDKSI ATIDVSVPANFSKSGYTF
AFSKNLITSFDGAVGYSLGGARVELEASYRRFATLADGQYAKSGAESLAA
ITRDANITETNYFVVKIDEITNTSVMLNGCYDVLHTDLPVSPYVCAGIGA
SFVDISKQVTTKLAYRGKVGISYQFTPEISLVAGGFYHGLFDESYKDIPA
HNSVKFSGEAKASVKAHIADYGFNLGARFLFS
> GLN2_RHILP Q02154 GLUTAMINE SYNTHETASE II (EC 6.3 .1.2) (GLU
MTKFKLEYIWLDGYTPVPNLRGKTQIKEFDEFPTLEQLPLWGFDGSSTMQ
AEGSSDCVLKPVAIYPDPARTNGALVMCEVMMPDGHAHASNARATILDDE
DAWFGFEQEYFFYQNGRPLGFPEQGYPAPQPYYTGVGYSNVGDVAREIVE
EHLDLCLAAGINHEGINAEVAKGQWEFQIFGKGSKKAADQIWMARYLLQR
LTEKYGIDIEYHCKPLGDTDWNGSGMHCNFSTKYLREVGGK EYFEALMAS
SDKNLMDHIAVYGPDNDKRLTGKHETAPWNKFSYGVADRGASIRVPHSFI
KNDYKGYLEDRRPNSQGDPYQIVRRF
> LYSR_ECOLI P03030 TRANSCRIPTIONAL ACTIVATOR PROTEIN LYSR. -

MAAVNLRHIEIFHAVMTAGSLTEAAHLLHTSQPTVSRELARFEKVIGLKL
FERVRGRLHPTVQGLRLFEEVQRSWYGLDRIVSAAESLREFRQGELSIAC
LPVFSQSFLPQLLQPFLARYPDVSLNIVPQESPLLEEWLSAQRHDLGLTE
TLHTPAGTERTELLSLDEVCVLPPGHPLAVKKVLTPDDFQGENYISLSRT
DSYRQLLDQLFTEHQVKRRMIVETHSAASVCAMVRAGVGISVVNPLTALD
YAASGLVVRRFSIAVPFTVSLIRPLHRPSSALVQAFSGHLQAGLPKLVTS
LDAILSSATTA
> P06191|ALR2_SALTY Alanine racemase, catabo lic - Salmonella typhimurium.
MTRPIQASLDLQVMKQNLAIVRRAAPEARVWSVVKANAYGHGIERVWSAL
GATDGFAMLNLEEAITLRERGWKGPILMLEGFFHAQDLEAYDTYRLTTCI
HSNWQLKALQNARLNAPLDIYVKVNSGMNRLGFQPERAQTVWQQLRAMRN
VGEMTLMSHFAQADHPEGIGEAMRRIALATEGLQCAYSLSNSAATLWHPQ
AHYDWVRPGIILYGASPSGQWR DIADTGLKPVMTLSSEIIGVQTLSAGER
VGYGGGYSVTQEQRIGIVAAGYADGYPRHAPTGTPVLVDGIRTRTVGTVS
MDMLAVDLTPCPQAGIGTPVELWGKEIKVDDVASAAGTLGYELLCAVAPR
VPFVTT
> Q55729|SYM_SYNY3 Methionyl -tRNA synthetase - Synechocystis sp. (strain PCC 6803).
MLDSSVPTFSVTTPLYYVNDVPHLGSAYTTVVAD TLARFKRLQGYDVLMI
TGTDEHGQKIQRTAEAQELDPQTHCDRTVVKFKELWRSLNILYDRFSRTT
DPRHLAIVKDFFQRVWDKGDIYLAQQQGWYCVACEEFKEKRDLLEDNHCP
LHPNRKAEWRDEENYFFRLSRYQHPLEELYAQRPEFIQPSSRRNEVLNFV
AQGLQDFSISRVNLDWGFPLPNDPNHTIYVWFDALLGYVTALLDEDEEPN
LTNALVKWWPINLHLIGKDILRFHAVYWPAMLMSA ELAIPAQVFGHGFLT
KDGQKMGKSLGNTVDPLDLINRYGEDAFRYYFLKEIEFGKDGDFNEQRFV
NVLNADLANDLGNLLNRTLGMVKKYCQGQGPQVMATDLAPDNPLKALGSH
LGEEVSSAYERLSFTDACEAIFTLVRAGNKYIDDMAPWKLFKQGSQKEVE
DVLYSVLESIRLSAYLLSPIVPRLSTKIYQQLGFTWDFDQWRSPLEQAEE
FNRHQSWGQLGANQNLPPAQPIFTKLELPAEE
> P50176|PHBC_RHIME Poly -beta-hydroxybutyrate polymerase - Rhizobium meliloti (Sinorhizobium meliloti).
MTAEKAEGATGFAGFDPKSVEPYIVKDPESLAINMARAAEQLGKAASAWL
APREAGEKTDSFAEPVSDMVKTLSKVSEYWLSDPRRTLEAQTHLLGSFFD
MWSRTLQRMAGDAVEDPANLQRNDKRFADEDWVKNPFFDFIRQAYFVTSD
WAERMVRDAEGLDDHTRHKAAFYVRQIASALSPTNFITTNPQLYRETVAS
SGANLVKGMQMLAEDIAAGRGELRLRQTDTSKFAIGENIAITPGKVIAQN
DVCQVLQYEASTETVLKRPLLICPPWINKFYVLDLNPEKSFIKWAVDQGQ
TVFVISWVNPDERHASKDWEAYAREGIGFALDIIEQATGEREVNSIGYCV
GGTLLAATLALHAAEGDERIRSATLFTTQVDFTHAGDLKVFVDDDQIRHL
EANMSATGYLEGSKMASAFNMLRASELIWPYFVNNYLKGQDPLPFDLLYW
NSDSTRMPAANHSFYLRNCYLENRLSKGEMVLAGRRVSLGDVKIPIYNLA
TKEDHIAPAKSVFLGSSSFGGKVTFVLSGSGHIAGVVNPPARSKYQYWTG
GAPKGDIETWMGKAKETAGSWWPHWQGWVERLDKRRVPARKAGGPLNSIE
EAPGSYVRVRA
> P37080|PTRA_KLEPN Sorbose -specific phosph otransferase enzyme IIA component - Klebsiella pneumoniae.
MVHAIFCAHGQLAGAMLDSVCMVYGEVNVSAVAFVPGENAADIAINLEKL
VSAHTDEEWVIAVDLQCGSPWNAAAGLAMRHPQIRVISGLSLPLALELVD
NQHTLSADDLCQHLQAIASQCCVVWQQPETVEEEF
> P18815|MALE_ENTAE Maltose -binding periplasmic protein - Enterobacter aerogenes (Aerobacter aerogenes).
MKIKTGARILALSALTTMMFSASALAKIEEGKLVIWINGDKGYNGLAEVG
KKFEKDTGIKVSVEHPDKLEEKFPQVAATGDGPDIIFWAHDRFGAYAQSG
LLAEITPDKAFQDKLYPFTWDAVRYNGKLIAYPVAVEALSLIYNKDLVPN
PPKTWEEIPALDKALKAKGKSALMFNLQEPYFTWPLIAADGGYAFKFENG
KYDVKNVGVDSAGAKAGLTFLVDLIKNKHMNADTDYSIAEAAFNKGETAM
TINGPWAWSNIDKSKVNYGVTLLPTFKGKPSKPFVGVLSAGINAASPNKE
LAKEFLENYLMTDPGLEAVNNDKPLGAVAVKSFQEKLEKDPRIAATMANA
QKGEIMPNIPQMSAFWYAVRTAVINAASARQTVDAALKDAQGRITK
> P15320|HLYA_SERMA Hemolysin - Serratia marcescens.
MKNNNFRLSAAGKLAAALAIILAASAGAYAAEIVAANGANGPGVSTAATG
AQVVDIVAPNGNGLSHNQYQDFNVNQPGAVLNNSREAGLSQLAGQLGANP
NLGGREASVILNEVIGRNPSLLHGQQEIFGMAADYVLANPNGISCQSCGF

INTSHSSLVVGNPLVENGVLQGYSTFGNRNTLSLNGTLNAGGVLDLIAPK
IDSRGEVIVQDFKQSNGKVTSAAINAISGLNRVARDGTVQASQQMPTALD
SYYLGSMQAGRINIINTAQGSGVKLAGSLNAGDELKVKAYDIRSESRVDD
ASSNKNGGDNYQNYRGGIYVNDRSSSQTLTRTELKGKNISLVADNHAHLT
ATDIRGEDITLQGGKLTLDGQQLKQTQGHTDDRWFYSWQYDVTREREQLQ
QAGSTVAASGSAKLISTQEDVKLLGANVSADRALSVKAARDVHLAGLVEK
DKSSERGYQRNHTSSLRTGRWSNSDESESLKASELRSEGELTLKAGRNVS
TQGAKVHAQRDLTIDADNQIQVGVQKTANAKAVRDDKTSWGGIGGGDNKN
NSNRREISHASELTSGGTLRLNGQQGVTITGSKARGQKGGEVTATHGGLR
IDNALSTTVDKIDARTGTAFNITSSSHKADNSYQSSTASELKSDTNLTLV
SHKDADVIGSQVASGGELSVESKTGNINVKAAERQQNIDEQKTALTVNGY
AKEAGDKQYRAGLRIEHTRDSEKTTRTENSASSLSGGSVKLKAEKDVTFS
GSKLVADKGDASVSGNKVSFLAADDKTASNTEQTKIGGGFYYTGGIDKLG
SGVEAGYENNKTQAQSSKAITSGSDVKGNLTINARDKLTQQGAQHSVGGA
YQENAAGVDHLAAADTASTTTTKTDVGVNIGANVDYSAVTRPVERAVGKA
AKLDATGVINDIGGIGAPNVGLDIGAQGGSSEKRSSSSQAVVSSVQAGSI
DINAKGEVRDQGTQYQASKGAVNLTADSHRSEAAANRQDEQSRDTRGSAG
VRVYTTTGSDLTVDAKGEGGTQRSNSSASQAVTGSIDAANGINVNVKKDA
IYQGTALNGGRGKTAVNAGGDIRLDQASDKQSESRSGFNVKASAKGGFTA
DSKNFGAGFGGGTHNGESSSSTAQVGNISGQQGVELKAGRDLTLQGTDVK
SQGDVSLSAGNKVALQAAESTQTRKESKLSGNIDLGAGSSDSKEKTGGNL
SAGGAFDIAKVNESATERQGATIASDGKVTLSANGKGDDALHLQGAKVSG
GSAALEAKNGGILLESAKNEQHKDNWSLGIKANAKGGQTFNKDAGGKVDP
NTGKDTHTLGAGLKVGVEQQDKTTHANTGITAGDVTLNSGKDTRLAGARV
DADSVQGKVGGDLHVESRKDVENGVKVDVDAGLSHSNDPGSSITSKLSKV
GTPRYAGKVKEKLEAGVNKVADATTDKYNSVARRLDPQQDTTGAVSFSKA
EGKVTLPATPAGEKPQGPLWDRGARTVGGAVKDSITGPAGRQGHLKVNAD
VVNNNAVGEQSAIAGKNGVALQVGGQTQLTGGEIRSQQGKVELGGSQVSQ
QDVNGQRYQGGGRVDAAATVGGLLGGAAKQSVAGNVPFASGHASTQQADA
KAGVFSGK
> P0AD64|BLA1_KLEPN Beta-lactamase SHV-1 - Klebsiella pneumoniae.
MRYIRLCIISLLATLPLAVHASPQPLEQIKLSESQLSGRVGMIEMDLASG
RTLTAWRADERFPMMSTFKVVLCGAVLARVDAGDEQLERKIHYRQQDLVD
YSPVSEKHLADGMTVGELCAAAITMSDNSAANLLLATVGGPAGLTAFLRQ
IGDNVTRLDRWETELNEALPGDARDTTTPASMAATLRKLLTSQRLSARSQ
RQLLQWMVDDRVAGPLIRSVLPAGWFIADKTGAGERGARGIVALLGPNNK
AERIVVIYLRDTPASMAERNQQIAGIGAALIEHWQR
> Q05489|LIP_BURGL Lipase - Burkholderia glumae (Pseudomonas glumae).
MVRSMRSRVAARAVAWALAVMPLAGAAGLTMAASPAAVAADTYAATRYPV
ILVHGLAGTDKFANVVDYWYGIQSDLQSHGAKVYVANLSGFQSDDGPNGR
GEQLLAYVKQVLAATGATKVNLIGHSQGGLTSRYVAAVAPQLVASVTTIG
TPHRGSEFADFVQDVLKTDPTGLSSTVIAAFVNVFGTLVSSSHNTDQDAL
AALRTLTTAQTATYNRNFPSAGLGAPGSCQTGAATETVGGSQHLLYSWGG
TAIQPTSTVLGVTGATDTSTGTLDVANVTDPSTLALLATGAVMINRASGQ
NDGLVSRCSSLFGQVISTSYHWNHLDEINQLLGVRGANAEDPVAVIRTHV
NRLKLQGV
> P73141|SYY_SYNY3 Tyrosyl-tRNA synthetase - Synechocystis sp. (strain PCC 6803).
MVVNSDPDWLWRGTAEIFPHNADSNNPQEHLGILTQQDRPLRVKLGIDPT
GTDIHLGHSIPFRKLRAFQDAGHTAVVIIGDFTAQIGDPTGKSEVRKQLT
ADQVKRNAESYLAQLKPILDFDTPGRLEIRYNSEWLSNLDLNKILELLAT
MTVGQMLAKEGFAERFQKENPIFLHEFLYPLMQGYDSVAVNADVELGGTD
QKFNIAVGRDLQRHFGKTPQFGLLLPILLGTDGQQKMSKSLNNYVGLQED
ALSMYSKLEKTPDRLLGEYFELLTNLDLAELPENPRECQKLLAKEVTAQF
HGVAGAIAAQKTAEDIVTQGKAGNTDSVPEFSLAEITFPVKLAYLLSASG
LCPSSSEGRRQIKGGAVRLDGDRLEDVNQEYADPKMLINKVLQMGKKKFI
RLIS
> P04338|NODA_RHILV Nodulation protein A - Rhizobium leguminosarum bv. viciae.
MSSEVRWKICWENELEASDHAELADFFCKTYGPTGAFNAKPFETGRSWGG
ARPERRAIAYDSHGVASHMGLLRRFIKVGTTDLLVAELGLYGVRPDLEGL
GIAHSVRAMFPILRELSVPFAFGTVRHAMRNHMERYCRDGTANIMTGLRV
RSTLPDAHSDLPATRTEDVLVLVVPVDRPMTEWPAGSLIERNGSEL

> GT_HAEIN P44521 GLUTATHIONE S -TRANSFERASE (EC 2.5.1.18). -
MKLYGLIGACSFVPHVALEWVKIRENADYEFEPVTRELIKSPEFLSLNPR
GAVPVLVDGDLVLSQ NQAILHYLDELYPNSKLFGSKTVRDKAKAARWLAF
FNSDVHKSFVPLFRLPNYAKDNETLAHTIRQQAVEQILDQLAVANEHLES
HIYFGENISVADAYLYIMLNWCKAVKIDFSHLTQLSAFMQRVETDQAVEN
VRKSEELKV
> P06717|ELAP_ECOLI Heat -labile enterotoxin A chain - Escherichia coli.
MKNITFIFFILLASPLYANGDRLYRADSRPPDEIKR SGGLMPRGHNEYFD
RGTQMNINLYDHARGTQTGFVRYDDGYVSTSLSLRSAHLAGQSILSGYST
YYIYVIATAPNMFNVNDVLGVYSPHPYEQEVSALGGIPYSQIYGWYRVNF
GVIDERLHRNREYRDRYYRNLNIAPAEDGYRLAGFPPDHQAWREEPWIHH
APQGCGNSSRTITGDTCNEETQNLSTIYLREYQSKVKRQIFSDYQSEVDI
YNRIRDEL
> P52276|SYN_SYNY3 Asparagin yl-tRNA synthetase - Synechocystis sp. (strain PCC 6803).
MNKRRIIEVLRHGQADDQVMVQGWLRTKRTLKDFSFVEVNDGSSLANLQV
VLDGSLADYDRLLSQLQTGAALVVEGKLAPSPGKGQRVELKATKLELLGG
ADPGSYPLQKKRHSFEFLRTIGHLRPRTNTIGAVMRVRNACATAIHQFFQ
ERGFLWVHTPIITASDCEGAGDLFKVTTLDLQRVPKNGEGIDYSQ DFFGK
QAYLTVSGQLEAEVMALAFQNVYTFGPTFRAENSNTSRHLAEFWMVEPEM
AFCDLEGDRQWAEEFLKYIFKFVLEKCPEDMEFFDQRIDNTVLATADNII
NNEFAWLTYSEAIKLLEKADQKFEYPVAWGVDLQSEHERYLAETVFKRPT
IVTDYPKDIKAFYMRLNDDGKTVAAMDILAPKIGEIIGGSQREERLDILT
QRMQEQGVPEEDLWWYLDLRRYGSVPHAGFGLGFERIVQFMTGMAN IRDV
IPFPRTPMNAEF
> P55875|IF2_STIAU Translation initiation factor IF -2 - Stigmatella aurantiaca.
MSKKRVHEIAKELKGHGIELDNKEVVTELAGLGYDVKSHSSSLDDDQATA
AVQKILDKRKPKQAAAPVTAKGFVVRRKVGPPTGSGVYDASQEPSQAASD
VSSPPSEPVHEASGAEAAASERVPEAAAVQEPVAEAPRAAASEPAAEAPK
ATAPVAPEPTVEAPKAAAPVAPEPTVEAPKTEAPVAAAPIAEAPTPPART
EVPVTSGRRAASCRGAAPLPCSGKDPLALNSSPQSSAAFCPDARNPGDCD
FPSTSGRWHAWPSRGSSGRFAHGAGRPSGWTFARWTSGRPRAASRRTAVQ
RPSGRAGAGASHGLQRRKGFGAGAQASGQPQNVTMVGGIPHAPTAPDARA
LRPTATQAVVISRPLIQVRRVTPTTSSAKQYPMAPGKKAIGEVREFKVVP
DHAGRGRELVDVSKNKDKSPRKRGGPNDTSISKQELTDLAWGRVNIPLRG
KKKKPTKKGAKTQITQMAEDKKVIKLQEGISVSDLGQRMGVRTSDIIKKL
MGLGKMATANQMVDADTVELIASDYGWKVDRVGFEVEDYLPEVVARPEDA
RTRPPVVTVMGHVDHGKTSLLDAIRAANVASGEAGGITQHIGAYSVTTAR
GDITFLDTPGHEAFTSMRARGANVTDIVILVVAADDGVMPQTIEAIKHAK
AAEVPIVVALNKMDVPGANPDRVKKDLANHELVPEEWGGETIMVPVSAKQ
KMGIDLLLENVVLQAEVLELTSNPSRPAVGAIIEGELDRGRGPVATVLVQ
EGTLRVGDAVVTGTDYGRVRAMNNSRGESVKEVLPGYCAEVIGLSGVPSA
GDTINVVADEKAAKQIAEHRGMKERQSELSKVSRETLDQLFAKTKAGGGP
KELRVVIKADVQGSAEAVKQAVQKLTTHKVKVEVIDTGVGAITESDVMRA
AASKGVVLGFNVKPESGAESAAKAEGVMLRSFSIIYELIDGVRSSMEELL
EPIRTERKLGRAEVRNTFNVPKLGTIAGAAVLDGVIKRGAFVRLMRENKQ
LFAGKMASLRRFKDDVKEVAQGFECGIGIENFNDLKAGDIIEAYEIEETR
QSLT
> Q03961|KPSD1_ECOLI Polysialic acid transport protein kpsD - Escherichia coli.
MKLFKSILLIAACHAAQASAAIDINA DPNLTGAAPLTGILNGQQSDTQNM
SGFDNTPPPSPPVVMSRMFGAQLFNGTSADSGATVGFNPDYILNPGDSIQ
VRLWGAFTFDGALQVDPKGNIFLPNVGPVKVAGVSNSQLNALVTSKVKEV
YQSNVNVYASLLQAQPVKVYVTGFVRNPGLYGGVTSDSLLNYLIKAGGVD
PERGSYVDIVVKRGNRVRSNVNLYDFLLNGKLGLSQFADGDTIIVGPRQH
TFSVQGDVFNSYDFEFRESSIPVTEAL SWARPKPGATHITIMRKQGLQKR
SEYYPISSAPGRMLQNGDTLIVSTDRYAGTIQVRVEGAHSGEHAMVLPYG
STMRAVLEKVRPNSMSQMNAVQLYRPSVAQRQKEMLNLSLQKLEEASLSA
QSSTKEEASLRMQEAQLISRFVAKARTVVPKGEVILNESNIDSVLLEDGD
VINIPEKTSLVMVHGEVLFPNAVSWQKGMTTEDYIEKCGGLTQKSGNARI
IVIRQNGAAVNAEDVDSLKPGDEIMVLP KYESKNIEVTRGISTILYQLAV
GAKVILSL

> P37696|GUNA_ACEXY Probable endoglucanase - Acetobacter xylinus (Gluconacetobacter xylinus).
MSVMAAMGGAQVLSSTGAFADPAPDAVAQQWAIFRAKYLRPSGRVVDTGN
GGESHSEGQGYGMLFAASAGDLASFQSMWMWARTNLQHTNDKLFSWRFLK
GHQPPVPDKNNATDGDLLIALALGRAGK RFQRPDYIQDAMAIYGDVLNLM
TMKAGPYVVLMPGAVGFTKKDSVILNLSYYVMPSLLQAFDLTADPRWRQV
MEDGIRLVSAGRFGQWRLPPDWLAVNRATGALSIASGWPPRFSYDAIRVP
LYFYWAHMLAPNVLADFTRFWNNFGANALPGWVDLTTGARSPYNAPPGYL
AVAECTGLDSAGELPTLDHAPDYYSAALTLLVYIARAEETIK
> P0ADU5|YGIW_ECOLI Protein ygiW - Escherichia coli.
MKKFAAVIAVMALCSAPVMAAEQGGFSGPSATQSQAGGFQGPNGSVTTVE
SAKSLRDDTWVTLRGNIVERISDDLYVFKDASGTINVDIDHKRWNGVTVT
PKDTVEIQGEVDKDWNSVEIDVKQIRKVNP
> P37735|DCTP_RHOCA C4-dicarboxylate-binding periplasmic protein - Rhodobacter capsulatus (Rhodopseudomonas capsulata).
MLTRRILGALVGATALSLALSVPALAEPIVIKFSHVVAPDTPKGKGAAKF
EELAEKYTNGAVDVEVYPNSQLYKDKEELEALQLGAVQMLAPSLAKFGPL
GVQDFEVFDLPYIFKDYEALHKVTQGEAGKMLLSKLEAKGITGLAFWDNG
FKIMSANTPLTMPDDFLGLKMRIQSSKVLEAEMNALGAVPQVMAFSEVYQ
ALQTGVVDGTENPPSNMFTQKMNEVQKHATVSNHGYLGY AVIVNKQFWDG
LPADVRTGLEKAMAESTDYANGIAKEENEKALQAMKDAGTTEFHELTAEE
RAAWEEVLTPVHDEMAERIGAETIAAVKAATAE
> P0A402|PSAF_SYNEN Photosystem I reaction center subunit III - Synechococcus elongatus naegeli.
MRRFLALLLVLTLWLGFTPLASADVAGLVPCKDSPAFQKRAAAAVNTTAD
PASGQKRFERY SQALCGEDGLPHLVVDGRLSRAGDFLIPSVLFLYIAGWI
GWVGRAYLIAVRNSGEANEKEIIIDVPLAIKCMLTGFAWPLAALKELASG
ELTAKDNEITVSPR
> 26KD_HELPY P21762 26 KD ANTIGEN. - HELICOBACTER PYLORI (CAM
MLVTKLAPDFKAPAVLGNNEVDEHFELSKNLGKNGAILFFWPKDFTFVCP
TEIIAFDKRVKDFQEKGFNVIGVSIDSEQVHFAWKNT PVEKGGIGQVTFP
MVADITKSISRDYDVLFEEAIALRGAFLIDKNMKVRHAVINDLPLGRNAD
EMLRMVDALLHFEEHGEVCPAGWRKGDKGMKATHQGVAEYLKENSIKL
> P42454|RURE_ACIAD Rubredoxin-NAD(+) reductase - Acinetobacter sp. (strain ADP1).
MHPIVIIGSGMAGYTLAREFRKLNPEHELVMICADDAVNYAKPTLSNALS
GNKAPEQI PLGDAEKMSTQLKLQILSETWVKAINPETHELKLEKNGQETI
QPYSKLVLAVGANPTRLAIAGDGSDDIHVVNSLIDYRAFRENLAKRQDKR
VVILGAGLIGCEFANDLQHTGHQVTVIDLSPRPLGRLLPAHIADAFQKNL
EESGIHFVLSTTVEKVSKINDGQDYAVTLANGQTLVADIVLSAIGLQPNI
DLAKHAGVHTSRGILTNSLLETNLEDIYAIGDCAEVNGTLLPYVMPIMQQ
ARALAKTLS GETTHVHYPAMPVAVKTPAAPLTVLPVPVDVDVNWETEEFE
DGMLAKAIDNTDTLRGFVLLGATAGKQRLTLTKLVPDLIPAQL
> P17543|PAPJ_ECOLI Protein papJ - Escherichia coli.
MVVNKTTAVLYLIALSLSGFIHTFLRAEERGIYDDVFTADALRHYRINER
GGRTGSLTCSGALLSSPCTLVSNEVPLSLRPENHSAAAGAPLMLRLAGCG
DGGALQPGKRGVAMT VSGSLVTGPGSGSALLPDRKLSGCDHLVIHDGDTF
LLCRPDRRQEEMLAAWRKRATQEGEYSDARSNPAMLRLSIKYE
> P31133|POTF_ECOLI Putrescine-binding periplasmic protein - Escherichia coli.
MTALNKKWLSGLVAGALMAVSVGTLAAEQKTLHIYNWSDYIAPDTVANFE
KETGIKVVYDVFDSNEVLEGKLMAGSTGFDLVVPSASFLERQLTAG VFQP
LDKSKLPEWKNLDPELLKLVAKHDPDNKFAMPYMWATTGIGYNVDKVKAV
LGENAPVDSWDLILKPENLEKLKSCGVSFLDAPEEVFATVLNYLGKDPNS
TKADDYTGPATDLLLKLRPNIRYFHSSQYINDLANGDICVAIGWAGDVWQ
ASNRAKEAKNGVNVSFSIPKEGAMAFFDVFAMPADAKNKDEAYQFLNYLL
RPDVVAHISDHVFYANANKAATPLVSAEVRENPGIYPPADVRAKLFT LKV
QDPKIDRVRTRAWTKVKSGK
> P33012|GYRI_ECOLI DNA gyrase inhibitory protein - Escherichia coli.
MNYEIKQEEKRTVAGFHLVGPWEQTVKKGFEQLMMWVDSKNIVPKEWVAV
YYDNPDETPAEKLRCDTVVTVPGYFTLPENSEGVILTEITGGQYAVAVAR
VVGDDFAKPWYQFFNSLLQDSAYEMLPKPCFEVYLNNGAEDGYWDIEMYV
AVQPKHH

> P09403|PGK_THET8 Phosphoglycerate kinase - Thermus thermophilus (strain HB8 / ATCC 27634 / DSM 579).
MRTLLDLDPKGKRVLVRVDYNVPVQDGKVQDETRILESLPTLRHLLAGGA
SLVLLSHLGRPKGPDPKYSLAPVGEALRAHLPEARFAPFPPGSEEARREA
EALRPGEVLLLENVRFEPGEEKNDPELSARYARLGEAFVLDAFGSAHRAH
ASVVGVARLLPAYAGFLMEKEVRALSRLLKDPERPYAVVLGGAKVSDKIG
VIESLLPRIDRLLIGGAMAFTFLKALGGEVGRSLVEEDRLDLAKDLLGRA
EALGVRVYLPEDVVAAERIEAGVETRVFPARAIPVPYMGLDIGPKTREAF
ARALEGARTVFWNGPMGVFEVPPFDEGTLAVGQAIAALEGAFTVVGGGDS
VAAVNRLGLKERFGHVSTGGGASLEFLEKGTLPGLEVLEG
> DEOC_ECOLI P00882 DEOXYRIBOSE-PHOSPHATE ALDOLASE (EC 4.1.2.
MTDLKASSLRALKLMDLTTLNDDDTDEKVIALCHQAKTPVGNTAAICIYP
RFIPIARKTLKEQGTPEIRIATVTNFPHGNDDIDIALAETRAAIAYGADE
VDVVFPYRALMAGNEQVGFDLVKACKEACAAANVLLKVIIETGELKDEAL
IRKASEISIKAGADFIKTSTGKVAVNATPESARIMMEVIRDMGVEKTVGF
KPAGGVRTAEDAQKYLAIADELFGADWADARHYRFGASSLLASLLKALGH
GDGKSASSY
> P44322|IF1_HAEIN Translation initiation factor IF-1 - Haemophilus influenzae.
MAKEDCIEMQGTILETLPNTMFRVELENGHVVTAHISGKMRKNYIRILTG
DKVTVEMTPYDLSKGRIIFRSR
> P0A9P4|TRXB_ECOLI Thioredoxin reductase - Escherichia coli.
MGTTKHSKLLILGSGPAGYTAAVYAARANLQPVLITGMEKGGQLTTTTEV
ENWPGDPNDLTGPLLMERMHEHATKFETEIIFDHINKVDLQNRPFRLNGD
NGEYTCDALIIATGASARYLGLPSEEAFKGRGVSACATCDGFFYRNQKVA
VIGGGNTAVEEALYLSNIASEVHLIHRRDGFRAEKILIKRLMDKVENGNI
ILHTNRTLEEVTGDQMGVTGVRLRDTQNSDNI ESLDVAGLFVAIGHSPNT
AIFEGQLELENGYIKVQSGIHGNATQTSIPGVFAAGDVMDHIYRQAITSA
GTGCMAALDAERYLDGLADAK
> LPSZ_RHIME P31858 LIPOPOLYSACCHARIDE PROCESSING PROTEIN LPS
MKNVGIKKAALQMSRYVLASKQVQRALKLLGLPASRANEVSGIPKPADTS
LSFPGYEAFALHGEAWQAAGEQKPIILMFGVHPWKRDVLARYFSDFRVAY
VRTNTSWTKVQTSFCQFTPQAFVFWGMTEIRAAKNYAIKSSIPLWRVEDG
FLRSVGLGAQHVLPLSLAVDTTGIYFDPSRPSTLETLISEIGVTENATLI
ERARRCMSMISAFGLSKYNVGQDVPLKRLPPSDRRRVLVVGQVEDDASIV
MGCAARYTNNDIVRITQKENPEAEVIYRPHPDVLGGHRKEFSNPRDVANI
CTILSGDYDLGSLLDSVDHVYTITSLLGFEALIRRKKVTVFGAPFYSGWG
LTDDRQPTPRRTRKPSLDELFAAAYILYPRYCVGSLGSSAEIEHAIMSLA
LEKNGVPRELAEGVSSALPAEAINVDCVSQTLEGLKSIPS
> P74936|PROB_TREPA Glutamate 5-kinase - Treponema pallidum.
MIRALFAAAKKIVIKIGSNTLAQADGTPDEEFLAECARACAALMRDGKQI
VVVSSGAQVAGISALHCLSSPPQGAGLERHESRGVIPGDGASCKQALCAV
GQAELISRWRSAFAAHQQCVGQFLCTKEDFTDSDRAAQVRYTLSFLLERR
VVPILNENDALCCSDVPSVPADRRVSLSPQKRIGDNDSLSAFVALLWQAD
LLLLLSDIDGVYDKDPKAHTDAQHVPLVTDVSALVGKTSMGSSNVFGTGG
IATKLDAARLVTRAGIPLVLANGRHLDPILSLMRGDARGTLFVPVS
> P17266|OMPW_VIBCH Outer membrane protein W - Vibrio cholerae.
MKQTICGLAVLAALSSAPVFAHQEGDFIVRAGIASVVPNDSSDKVLNTQS
ELAVNSNTQLGLTLGYMFTDNISFEVLAATPFSHKISTSGGELGSLGDIG
ETKHLPPTFMVQYYFGEANSTFRPYVGAGLNYTTFFDESFNGTGTNAGLS
DLKLDDSWGLAANVGFDYMLNDSWFLNASVWYANIETTATYKAGADAKST
DVEINPWVFMIAGGYKF
> P0A3T3|BCSP_BRUAB 31 kDa immunogenic protein - Brucella abortus.
MKFGSKIRRLAVAAVAGAIALGASFAVAQAPTFFRIGTGGTAGTYYPIGG
LIANAISGAGEKGVPGLVATAVSSNGSVANINAIKSGALESGFTQSDVAY
WAYNGTGLYDGKGKVEDLRLLATLYPETIHIVARKDANIKSVADLKGKRV
SLDEPGSGTIVDARIVLEAYGLTEDDIKAEHLKPGPAGERLKDGALDAYF
FVGGYPTGAISELAISNGISLVPISGPEADKILEKYSFFSKDVVPAGAYK
DVAETPTLAVAAQWVTSAKQPDDLIYNITKVLWNEDTRKALDAGHAKGKL
IKLDSATSSLGIPLHPGAERFYKEAGVLK
> Q03155|AIDA_ECOLI Adhesin AIDA-I - Escherichia coli.
MNKAYSIIWSHSRQAWIVASELARGHGFVLAKNTLLVLAVVSTIGNAFAV
NISGTVSSGGTVSSGETQIVYSGRGNSNATV NSGGTQIVNNGGKTTATTV

NSSGSQNVGTSGATISTIVNSGGIQRVSSGGVASATNLSGGAQNIYNLGH
ASNTVIFSGGNQTIFSGGITDSTNISSGGQQRVSSGGVASNTTINSSGAQ
NILSEEGAISTHISSGGNQYISAGANATETIVNSGGFQRVNSGAVATGTV
LSGGTQNVSSGGSAISTSVYNSGVQTVFAGATVTDTTVNSGGNQNISSGG
IVSETTVNVSGTQNIYSGGSALSANIKGSQIV NSEGTAINTLVSDGGYQH
IRNGGIASGTIVNQSGYVNISSGGYAESTIINSGGTLRVLSDGYARGTIL
NNSGRENVSNGGVSYNAMINTGGNQYIYSDGEATAAIVNTSGFQRINSGG
TAPVQNSVVVTRTVSSAAKPFDAEVYSGGKQTVYLWRGIWYSNFLTAVWS
MFPGTASGANVNLSGRLNAFAGNVVGTILNQEGRQYVYSGATATSTVGNN
EGREYVLSGGITDGTVLNSGGLQAVSSGGKASA TVINEGGAQFVYDGGQV
TGTNIKNGGTIRVDSGASALNIALSSGGNLFTSTGATLPELTTMAALSVS
QNHASNIVLENGGLLRVTSGGTATDTTVNSAGRLRIDDGGTINGTTTINA
DGIVAGTNIQNDGNFILNLAENYDFETELSGSGVLVKDNTGIMTYAGTLT
QAQGVNVKNGGIIFDSAVVNADMAVNQNAYINISDQATINGSVNNNGSIV
INNSIINGNITNDADLSFGTAKLLSATVNGSLVN NKNIILNPTKESAGNT
LTVSNYTGTPGSVISLGGVLEGDNSLTDRLVVKGNTSGQSDIVYVNEDGS
GGQTRDGINIISVEGNSDAEFSLKNRVVAGAYDYTLQKGNESGTDNKGWY
LTSHLPTSDTRQYRPENGSYATNMALANSLFLMDLNERKQFRAMSDNTQP
ESASVWMKITGGISSGKLNDGQNKTTTNQFINQLGGDIYKFHAEQLGDFT
LGIMGGYANAKGKTINYTSNKAARNTLDGYSVGVY GTWYQNGENATGLFA
ETWMQYNWFNASVKGDGLEEEKYNLNGLTASAGGGYNLNVHTWTSPEGIT
GEFWLQPHLQAVWMGVTPDTHQEDNGTVVQGAGKNNIQTKAGIRASWKVK
STLDKDTGRRFRPYIEANWIHNTHEFGVKMSDDSQLLSGSRNQGEIKTGI
EGVITQNLSVNGGVAYQAGGHGSNAISGALGIKYSF
> P30860|ARTJ_ECOLI Arginine -binding periplasmic p rotein 2 - Escherichia coli.
MKKLVLAALLASFTFGASAAEKINFGVSATYPPFESIGANNEIVGFDIDL
AKALCKQMQAECTFTNHAFDSLIPSLKFRKYDAVISGMDITPERSKQVSF
TTPYYENSAVVIAKKDTYKTFADLKGKRIGMENGTTHQKYIQDQHPEVKT
VSYDSYQNAFIDLKNGRIDGVFGDTAVVNEWLKTNPQLGVATEKVTDPQY
FGTGLGIAVRPDNKALLEKLNNA LAAIKADGTYQKISDQWFPQ
> P23537|PTHP_RALEH Phosphocarrier protein HPr - Ralstonia eutropha (strain ATCC 17699 / H16 / DSM 428 / Stanier 337) (Cupriavidus necator (strain ATCC 17699 / H16 / DSM 428 / Stanier 337)).
MLQRDTTIINKLGLHARASAKLTQLAGNFVSQVKMSRNGRQVDA KSIMGV
MMLAAGIGSTVTLETDGPDEQEAMDALLALIANRFGEGE
> P14899|AMY3_DICTH Alpha -amylase 3 - Dictyoglomus thermophilum.
MKIKFFIKRTLIFIFILVTFLTYIHGYNEPWYKNAIFYEVFVRSFADSDG
DRVGDLNGLIDKLDYFKNLNITALWLMPIFPSVSYHGYDVTDYYDIHPGY
GTMEDFENLIRKAHEKNIKIILDLVVNHTSSRHPWFVSSASS YNSPYRDY
YIWSTEKPEKNSNLWYKKPTGYYYALFWSEMPDLNFDNPKVREEVKKIAK
FWIEKGVDGFRLDAAKHIYDDDSKNIQWWKEFYSYLKSIKPDVYLVGEVW
DNEYKIAEYYKGLPSNFNFPLSDKIMNSSSKSKRLRNYRISRLKRLFGEN
NTDFADAIFLRNHDQVRVRTFFGGSIDKSILAGSIYLTLAGNTFIYYGEE
IGMEGSKPDEYIREPFKWTDDMKSKYQTYWIIPRYNLPGNGIA LDTEEKD
PNSIYNHYKKLLEIRVKCRALSNGKIERIKTQDRSILAYKLELEDEKIMV
VHNLNRIENTFNFNNEIKEKDILYIRNAKTEKNKIILGPYSTVIVKIP
> P31522|PSAA_YERPE pH 6 antigen - Yersinia pestis.
MKMKCFAKNALAVTTLMIAACGMANASTVINSKDVSGEVTVKQGNTFHVD
FAPNTGEIFAGKQPGDVTMFTLTMGDTAPHGGWRLIPTGDSKGGY MISAD
GDYVGLYSYMMSWVGIDNNWYINDDSPKDIKDHLYVKAGTVLKPTTYKFT
GRVEEYVF
> P13415|OMPA1_NEIMC Major outer membrane protein P.IA - Neisseria meningitidis serogroup C.
MRKKLTALVLSALPLAAVADVSLYGEIKAGVEGRNIQAQLTEQPQVTNGV
QGNQVKVTKAKSRIRTKISDFGSFIGFKGSEDLGEGLKAVWQLEQD VSVA
GGGASQWGNRESFIGLAGEFGTLRAGRVANQFDDASQAINPWDSNNDVAS
QLGIFKRHDDMPVSVRYDSPEFSGFSGSVQFVPAQNSKSAYKPAYYTKDT
NNNLTLVPAVVGKPGSDVYYAGLNYKNGGFAGNYAFKYARHANVGRNAFE
LFLIGSATSDEAKGTDPLKNHQVHRLTGGYEEGGLNLALAAQLDLSENGD
KAKTKNSTTEIAATASYRFGNAVPRISYAHGFDLIERGKKGENTSYD QII
AGVDYDFSKRTSAIVSGAWLKRNTGIGNYTQINAASVGLRHKF
> P26602|UBIC_ECOLI Chorismate --pyruvate lyase - Escherichia coli.

MSHPALTQLRALRYCKEIPALDPQLLDWLLLEDSMTKRFEQQGKTVSVTM
IREGFVEQNEIPEELPLLPKESRYWLREILLCADGEPWLAGRTVVPVSTL
SGPELALQKLGKTPLGRYLFTSSTLTRDFIEIGRDAGLW GRRSRLRLSGK
PLLLTELFLPASPLY
> P08704|CDGT_KLEOX Cyclomaltodextrin glucanotransferase - Klebsiella oxytoca.
MKRNRFFNTSAAIAISIALNTFFCSMQTIAAEPEETYLDFRKETIYFLFL
DRFSDGDPSNNAGFNSATYDPNNLKKYTGGDLRGLINKLPYLKSLGVTSI
WITPPIDNVNNTDAAGNTGYHGYWGRDYFRIDEHFGNLDDFKELTSL MHS
PDYNMKLVLDYAPNHSNANDENEFGALYRDGVFITDYPTNVAANTGWYHH
NGGVTNWNDFFQVKNHNLFNLSDLNQSNTDVYQYLLDGSKFWIDAGVDAI
RIDAIKHMDKSFIQKWTSDIYDYSKSIGREGFFFFGEWFGASANTTTGVD
GNAIDYANTSGSALLDFGFRDTLERVLVGRSGNTMKTLNSYLIKRQTVFT
SDDWQVVFMDNHDMARIGTALRSNATTFGPGNNETGGSQSEAFAQKRI DL
GLVATMTVRGIPAIYYGTEHYAANFTSNSFGQVGSDPYNREKMPGFDTES
EAFSIIKTLGDLRKSSPAIQNGTYTELWVNDDILVFERRSGNDIVIVALN
RGEANTINVKNIAVPNGVYPSLIGNNSVSVANKRTTLTLMQNEAVVIRSQ
SDDAENPTVQSINFTCNNGYTISGQSVYIIGNIPQLGGWDLTKAVKISPT
QYPQWSASLELPSDLNVEWKCVKRNETNPTANVEWQSGANNQFNSNDTQ T
TNGSF
> P43817|SYD_HAEIN Aspartyl -tRNA synthetase - Haemophilus influenzae.
MMRTHYCGALNRNNIGQDVTLSGWVHRRRDLGGLIFIDMRDRDGIVQVCF
DPKYQDALTAAAGLRNEFCIQIKGEVIARPDNQINKNMATGEVEVLAKEL
RIYNASDVLPLDFNQNNTEEQRLKYRYLDLRRPEMAQRLKTRAKITSFVR
RFMDDNGFLDIETPMLTKATPEGAR DYLVPSRVHKGKFYALPQSPQLFKQ
LLMMSGFDRYYQIVKCFRDEDLRADRQPEFTQIDVETSFLTAPEVREIME
RMVHGLWLDTIGVDLGKFPVMTWQEAMRRFGSDKPDLRNPLEMVDVADIV
KDVEFKVFNEPANNPNGRVAVIRVPNGAEITRKQIDEYTQFVGIYGAKGL
AWAKVNDINAGLEGVQSPIAKFLNEDVWKGLAERVNAQTGDILFFGADKW
QTTTDAMGALRLKLGRDLGLTRLDEW QPLWVIDFPMFERDEEGNLAAMHH
PFTSPKDFSPEQLEADPTSAVANAYDMVINGYEVGGGSVRIFDPKMQQTV
FRILGIDEEQQREKFGFLLDALKFGTPPHAGLAFGLDRLTMLLTGTENIR
DVIAFPKTTAAACLMTEAPSFANPQALEELAISVVKAE
> P00120|CY553_DESVM Cytochrome c -553 - Desulfovibrio vulgaris (strain Miyazaki).
MKRILVVMSICAALAFGVSAAMAADGAALYKSCVGCHGADGSKQAMGVGH
AVKGQKADELFKKLKGYADGSYGGEKKAVMTNLVKRYSDEEMKAMADYMS
KL
> P23608|PHBC_RALEH Poly -beta-hydroxybutyrate polymerase - Ralstonia eutropha (strain ATCC 17699 / H16 / DSM 428 / Stanier 337) (Cupriavidus necator (strain AT CC 17699 / H16 / DSM 428 / Stanier 337)).
MATGKGAAASTQEGKSQPFKVTPGPFDPATWLEWSRQWQGTEGNGHAAAS
GIPGLDALAGVKIAPAQLGDIQQRYMKDFSALWQAMAEGKAEATGPLHDR
RFAGDAWRTNLPYRFAAAFYLLNARALTELADAVEADAKTRQRIRFAISQ
WVDAMSPANFLATNPEAQRLLIESGGESLRAGVRNMMEDLTRGKISQTDE
SAFEVGRNVA VTEGAVVFENEYFQLLQYKPLTDKVHARPLLMVPPCINKY
YILDLQPESSLVRHVVEQGHTVFLVSWRNPDASMAGSTWDDYIEHAAIRA
IEVARDISGQDKINVLGFCVGGTIVSTALAVLAARGEHPAASVTLLTTLL
DFADTGILDVFVDEGHVQLREATLGGGAGAPCALLRGLELANTFSFLRPN
DLVWNYVVDNYLKGNTPVPFDLLFWNGDATNLPGPWYCWYLRHTYLQNEL
KVPGKLTVCGV PVDLASIDVPTYIYGSREDHIVPWTAAYASTALLANKLR
FVLGASGHIAGVINPPAKNKRSHWTNDALPESPQQWLAGAIEHHGSWWPD
WTAWLAGQAGAKRAAPANYGNARYRAIEPAPGRYVKAKA
> P43830|SYP_HAEIN Prolyl -tRNA synthetase - Haemophilus influenzae.
MRTSQYLFSTLKETPNDAQVVSHQLMLRAGMIRPMASGLYNWLPTGIRVL
KKVEKVVREEMNKGGAIEVLMPVVQPAELWEESGRWDQYGPELLRFEDRG
NRNFVLGPTHEEVITDLVRREVSSYKQLPLNLYQIQTKFRDEVRPRFGVM
RSREFIMKDAYSFHTTQESLQATYDVMYQVYSNIFNRLGLDFRAVQADTG
SIGGSASHEFQVLASSGEDDVVFSTESDFAANIELAEAIAIGERQAPTAE
MCLVDTPNAKTIAELVEQFNLPIEKTVKTLIVKGADENQPLVALIIRGDH
ELNEIKAQKHPLVADPLEFADETEIKAKIGSGVGSLGAVNLNIPAIIDRT
VALMSDFSCGANIDGKHYFNVNWERDVAIPKVFDLRNVVEGDPSPDGKGT
LQIKRGIEVGHIFQLGKKYSEAMKATVQGEDGKPLVMTMGCYGIGVTRVV

ASAIEQHHDERGIIWPSDEIAPFTVAIVPMNMHKSEAVQKYAEELYRTLQ
SQGVDVIFDDRKERPGVMFADMELIGVPHMVVIGEKNLDNGEIEYKNRRT
GEKEMISKDKLLSVLNEKLGNL
> HOXF_ALCEU P22317 NAD-REDUCING HYDROGENASE HOXS ALPHA SUBUN
MDSRITTILERYRSDRTRLIDILWDVQHEYGHIPDAVLPQLGAGLKLSPL
DIRETASFYHFFLDKPSGKYRIYLCNSVIAKINGYQAVREALERETGIRF
GETDPNGMFGLFDTPCIGLSDQEPAMLIDKVVFTRLRPGKITDIIAQLKQ
GRSPAEIANPAGLPSQDIAYVDAMVE SNVRTKGPVFFRGRTDLRSLLDQC
LLLKPEQVIETIVDSRLRGRGGAGFSTGLKWRLCRDAESEQKYVICNADE
GEPGTFKDRVLLTRAPKKVFVGMVIAAYAIGCRKGIVYLRGEYFYLKDYL
ERQLQELREDGLLGRAIGGRAGFDFDIRIQMGAGAYICGDESALIESCEG
KRGTPRVKPPFPVQQGYLGKPTSVNNVETFAAVSRIMEEGADWFRAMGTP
DSAGTRLLSVAGDCSKPGIYEVEWGVT LNEVLAMVGARDARAVQISGPSG
ECVSVAKDGERKLAYEDLSCNGAFTIFNCKRDLLEIVRDHMQFFVEESCG
ICVPCRAGNVDLHRKVEWVIAGKACQKDLDDMVSWGALVRRTSRCGLGAT
SPKPILTTLEKFPEIYQNKLVRHEGPLLPSFDLDTALGGYEKALKDLEEV
TR
> P50205|PHBB_RHIME Acetoacetyl-CoA reductase - Rhizobium meliloti (Sinorhizobium meliloti).
MSRVALVTGGSRGIGAAICVALKAAGYKVAANYAGNDERAKAFEQESGIP
VYKWDVSSYQACVDGIARVEADLGPVDILVNNAGITRDAMFHKMTPEQWG
EVIGTNLTGVFNMTHPLWSGMRDRGFGRIVNISSINGQKGQMGQVNYSAA
KAGDLGLTKALAQEGAAKGITVNAICPGYIGTEMVRAVPEKVLNERIIPQ
IPVGRLGEPEEVARCVVFLASDDAGFITGSTISAN GGQYFA
> P62593|BLAT_ECOLI Beta-lactamase TEM - Escherichia coli.
MSIQHFRVALIPFFAAFCLPVFAHPETLVKVKDAEDQLGARVGYIELDLN
SGKILESFRPEERFPMMSTFKVLLCGAVLSRVDAGQEQLGRRIHYSQNDL
VEYSPVTEKHLTDGMTVRELCSAAITMSDNTAANLLLTTIGGPKELTAFL
HNMGDHVTRLDRWEPELNEAIPNDERDTTMPAAMATT LRKLLTGELLTLA
SRQQLIDWMEADKVAGPLLRSALPAGWFIADKSGAGERGSRGIIAALGPD
GKPSRIVVIYTTGSQATMDERNRQIAEIGASLIKHW
> P52977|LON_CAUCR ATP-dependent protease La - Caulobacter crescentus (Caulobacter vibrioides).
MSELRTLPVLPLRDIVVFPHMVVPLFVGRDKSVRALEEVMRGDKQILLVT
QKNSADDDPAPGDIFEVGVLATVLQLLKLPDGTVKVLVEGKARAAVVSFT
DQESYYEAQIGEVSEDDGAGPEAEALSRAVVEQFENYVKLNKKVPPEALA
SIPQIAEPGKLADSIAAHLSVKIGDKQNLLEIFDVVKRLEKVFALMEGEI
SVLQVEKKIRSRVKRQMEKTQREYYLNEQMKAIQRELGDPDDARDELIDL
EKRIKKTKLSKEARTKAESELKKLRNMSPMSAESTVVRNYLDWLLSIPWG
KAKTKKIDLVESEGILDADHYGLEKVKERILEYLAVQARTNSLKGPILCL
VGPPGVGKTSLGKSIAKATGREFVRMSLGGVRDEAEIRGHRRTYIGSMPG
KVVQSMKKAKTTNAFVLLDEIDKMGSDYRGDPASALLEVLDPSQNSTFGD
HYLEVDYDLSQVMFVTTANSLNMPQPLLDRMEIIRIPGYTEDEKLEIAKR
HILPKLAKDHGLKPAEFIVPDKAIRDLIRYYTREAGVRSLERELGALARK
TVRDLAREKVASITIDDERLAKYAGVKKYRYGETDEVDQVGIVTGLAWTE
FGGDILTIEAVKMPGKGRMQITGNLKDVMKESIAAANSYVRSRALQFGIK
PPVFEKTDVHIHVPDGATPKDGPSAGIAMALAMVSVLTGIPIRKDIAMTG
EITLRGRVTAIGGLKEKLLAALRSGVKTVLIPQENEKDLADVPQTVKDGL
EIIPVSTVDEVLKHALTGPLTPVEWNEAEEPITTSAKKDDGDSDAMLTH
> P37918|TSAT_RICTS 56 kDa type-specific antigen - Rickettsia tsutsugamushi.
MKKIMLIASAMSALSLPFSASAIELGDEGGLECGPYAKVGVVGGMITGVE
STRLDPADAGGKKQLPLTTSMPFGGTLAAGMTIAPGFRAELGVMYLANVK
AEVESGKTGSDADIRSGADSPMPQRYKLTPPQPTIMPISIADRDLGVDIP
NVPQGGANHLGDNLGANDIRRADDRITWLKNYAGVDY MVPDPNNPQARIV
NPVLLNIPQGPPNANPRQAMQPCSILNHDHWRHLVVGITAMSNANKPSVS
PIKVLSEKIVQIYRDVKPFARVAGIEVPSDPLPNSASVEQIQNKMQELND
ILDEIRDSFDGCIGGNAFANQIQLNFRIPQAQQQGQGQQQQQAQATAQEA
AAAAAVRVLNNNDQIIKLYKDLVKLKRHAGIKKAMEELAAQDGGCNGGGD
NKKKRGASEDSDAGGASKGGKGKETKETEFDLSMIVGQ VKLYADLFTTES
FSIYAGLGAGLAYTSGKIDGVDIKANTGMVASGALGVAINAAEGVYVDIE
GSYMHSFSKIEEKYSINPLMASFGVRYNF
> P0A2D5|CHEY_SALTY Chemotaxis protein cheY - Salmonella typhimurium.
MADKELKFLVVDDFSTMRRIVRNLLKELGFNNVEEAEDGVDALNKLQAGG

FGFIISDWNMPNMDGLELLKTIRADSAMSALPVLMVTAEAK KENIIAAAQ
AGASGYVVKPFTAATLEEKLNKIFEKLGM
> P69441|KAD_ECOLI Adenylate kinase - Escherichia coli.
MRIILLGAPGAGKGTQAQFIMEKYGIPQISTGDMLRAAVKSGSELGKQAK
DIMDAGKLVTDELVIALVKERIAQEDCRNGFLLDGFPRTIPQADAMKEAG
INVDYVLEFDVPDELIVDRIVGRRVHAPSGRVYHVKFNPPKVEGKDDVTG
EELTTRKDDQEETVRKRLVEYHQMTAPLIGYYSKEAEAGNTKYAKVDGTK
PVAEVRADLEKILG
> P44312|G6PI_HAEIN Glucose-6-phosphate isomerase - Haemophilus influenzae.
MKNINPTHTHAWKSLEAHKAELSNITIQDLFKQEKNRFDDYSLTFNNQIL
IDFSKNNINQTTLSHLRQLAQECALDSAKEAMFTGEKINRTENRAVLHTA
LRNRTNTPVLVDGKDVMPE VNAVLAKMKDFCQRIISGEWKGYTGKAITDV
VNIGIGGSDLGPYMVTEALRPYKNHLNMHFVSNVDGTHIAETLKKVNPET
TLFLVASKTFTTQETMTNAQSARDWLLKAAKDESAVAKHFAALSTNAKDV
EKFGIDTNNMFEFWDWVGGRYSLWSAIGLSIALSIGFENFEALLNGAHEM
DKHFLSTPIEQNIPTTLALVGLWNTNFLGAQTEAILPYDQYLHRFAAYFQ
QGNMESNGKYVDRDGNVIKN YQTGPIIWGEPGTNGQHAFYQLIHQGTTLI
PCDFIAPAQRHNPLADHHNKLLSNFFAQTEALAFGKTKEEVEAEFVKAGK
SLDDVKNIVPFKVFTGNKPTNSILVQKITPFTLGALIAMYEHKIFVQGVI
FNIFSFDQWGVELGKQLANRILPELTDSEKVASHDSSTNGLINQFKAWR
> P02924|ARAF_ECOLI L-arabinose-binding periplasmic protein - Escherichia coli.
MHKFTKALAAIGLAAVMSQSAMAENLKLGFLVKQPEEPWFQTEWKFADKA
GKDLGFEVIKIAVPDGEKTLNAIDSLAASGAKGFVICTPDPKLGSAIVAK
ARGYDMKVIAVDDQFVNAKGKPMDTVPLVMMAATKIGERQGQELYKEMQK
RGWDVKESAVMAITANELDTARRRTTGSMDALKAAGFPEKQIYQVPTKSN
DIPGAFDAANSMLVQHPEVKHWLIVGMNDSTVLGGVRATEGQGFK AADII
GIGINGVDAVSELSKAQATGFYGSLLPSPDVHGYKSSEMLYNWVAKDVEP
PKFTEVTDVVLITRDNFKEELEKKGLGGK
> P09169|OMPT_ECOLI Protease 7 - Escherichia coli.
MRAKLLGIVLTTPIAISSFASTETLSFTPDNINADISLGTLSGKTKERVY
LAEEGGRKVSQLDWKFNNAAIIKGAINWDLMPQISIGAAGWTTLGSRGGN
MVDQDWMDSSNPGTWT DESRHPDTQLNYANEFDLNIKGWLLNEPNYRLGL
MAGYQESRYSFTARGGSYIYSSEEGFRDDIGSFPNGERAIGYKQRFKMPY
IGLTGSYRYEDFELGGTFKYSGWVESSDNDEHYDPGKRITYRSKVKDQNY
YSVAVNAGYYVTPNAKVYVEGAWNRVTNKKGNTSLYDHNNNTSDYSKNGA
GIENYNFITTAGLKYTF
> P44420|TYSY_HAEIN Thymidylate synthase - Haemophilus influenzae.
MKQYLELCRRIVSEGEWVANERTGKHCLTVINADLEYDVANNQFPLITTR
KSYWKAAIAEFLGYIRGYDNAADFRALGTKTWDANANENAAWLANPHRRG
VDDMGRVYGVQGRAWRKPNGETIDQLRKIVNNLTKGIDDRGEILTFFNPG
EFDLGCLRPCMHTHTFSLVGDTLHLTSYQRSCDVPLGLNFNQIQVFTFLA
LMAQITGKKAGKAYHKIVNAHIYEDQLELMRDVQL KREPFPLPKLEINPD
IKTLEDLETWVTMDDFKVVGYQSHEPIKYPFSV
> RFFE_ECOLI P27828 UDP-N-ACETYLGLUCOSAMINE 2-EPIMERASE (EC 5
MKVLTVFGTRPEAIKMAPLVHALAKDPFFEAKVCVTAQHREMLDQVLKLF
SIVPDYDLNIMQPGQGLTEITCRILEGLKPILAEFKPDVVLVHGDTTTTL
ATSLAAFYQRIPVGHVEAGLRTGDLYSPWPEEANRTLTGHLA MYHFSPTE
TSRQNLLRENVADSRIFITGNTVIDALLWVRDQVMSSDKLRSELAANYPF
IDPDKKMILVTGHRRESFGRGFEEICHALADIATTHQDIQIVYPVHLNPN
VREPVNRILGHVKNVILIDPQEYLPFVWLMNHAWLILTDSGGIQEEAPSL
GKPVLVMRDTTERPEAVTAGTVRLVGTDKQRIVEEVTRLLKDENEYQAMS
RAHNPYGDGQACSRILEALKNNRISL
> P19843|NOSD_PSEST Copper-binding periplasmic protein - Pseudomonas stutzeri (Pseudomonas perfectomarina).
MFKAQATFSRYSAAVSLLLLFSGAAQAAPQSITTLPLQPDGENRWRLPAG
EYQGQFTIEQPMQLRCEPGAVIQSQGQGSSLLISAPDVLVEGCTLYEWGS
DLTAMDSAVFILPAAERAQISNNRMRGPGFGVFVDGTRDVQVIGNEIDGD
AGVRSQDRGNGIHLFAVSGARVLHNHVRNARDGIYIDTSNGNHLEGNVIE
DVRYGVHYMFANENSLIDNVTRRTRTGYALMQSRKLTVTGNRSEQDQNYG
ILMNYITYSTITGNFVSDVQRGDTGGDSMISGGEGKALFIYNSLFNTIEN
NHFEKSSLGIHLTAGSEDNRISGNAFVGNQQQVKYVASRTQEWSVDGRGN
YWSDYLGWDRNNDGLGDIAYEPNDNVDRLLWLYPQVRLLMNSPSIEVLRW

VQRAFPVIKSPG VQDSHPLMKLPTEKLLTEKQEPTS
> P20713|ATSA_KLEAE Arylsulfatase - Klebsiella aerogenes.
MNKKAMAAAVSMILAGGAHAAQQERPNVIVIIADDMGYSDISPFGGEIPT
PNLQAMAEQGMRMSQYYTSPMSAPARSMLLTGNSNQQAGMGGMWWYDSTI
GKEGYELRLTDRVTTMAERFKDAGYNTLMAGKWHLGFVPGATPKDRGFNH
AFAFMGGGTSHFNDAIPLGT VEAFHTYYTRDGERVSLPDDFYSSEAYARQ
MNSWIKATPKEQPVFAWLAFTAPHDPLQAPDEWIKRFKGQYEQGYAEVYR
QRIARLKALGIIHDDTPLPHLELDKEWEALTPEQQKYTAKVMQVYAAMIA
NMDAQIGTLMETLKQTGRDKNTLLVFLTDNGANPAQGFYYESTPEFWKQF
DNSYDNVGRKGSFVSYGPHWANVSNAPYANYHKTTSAQGGINTDFMISGP
GITRHGKIDASTMAVYDVAPT LYEFAGIDPNKSLAKKPVLPMIGVSLSAI
SPAKYRSRRAELRG
> P43819|SYFA_HAEIN Phenylalanyl -tRNA synthetase alpha chain - Haemophilus influenzae.
MQHLNELVEKAKLAIESIQDKSLTALDEIRVEYFGKKGHFTQLMQELRNV
SAEERPAMGAKINEAKQAALEFLNAKKTEWEQAELNSKLEKERVDVSLPG
RKVETGGLHPVTMTINRVTKFF SELGFSVENGPEIESDYYNFDALNIPKH
HPARADHDTFWFNPELLLRTQTSGVQIRTMEKMQPPIRIMAPGRVYRNDY
DQTHTPMFHQIELLYVDKKANFTELKGLLHDFLRAFFEEDLQVRFRPSYF
PFTEPSAEVDVMGKNGKWLEVLGCGMVHPNVLRNVGIDPNEYSGFAVGMG
VERLTMLRYNVTDLRSFFENDLRFLKQFK
> O05542|DHET_GLUOX Alcohol dehydrogenase [a cceptor] - Gluconobacter oxydans (Gluconobacter suboxydans).
MTSGLLTPIKVTKKRLLSCAAALAFSAAVPVAFAQEDTGTAITSSDNGGH
PGDWLSYGRSYSEQRYSPLDQINTENVGKLKLAWHYDLDTNRGQEGTPLI
VNGVMYATTNWSKMKALDAATGKLLWSYDPKVPGNIADRGCCDTVSRGAA
YWNGKVYFGTFDGRLIALDAKTGKLVWSVYTIPKEAQLGHQR SYTVDGAP
RIAKGKVLIGNGGAEFGARGFVSAFDAETGKLDWRFFTVPNPENKPDGAA
SDDILMSKAYPTWGKNGAWKQQGGGGTVWDSLVYDPVTDLVYLGVGNGSP
WNYKFRSEGKGDNLFLGSIVAINPDTGKYVWHFQETPMDEWDYTSVQQIM
TLDMPVNGEMRHVIVHAPKNGFFYIIDAKTGKFITGKPYTYENWANGLDP
VTGRPNYVPDALWTLTGKPWLGIPGELGGHNFAAMAYSPKTKL VYIPAQQ
IPLLYDGQKGGFKAYHDAWNLGLDMNKIGLFDDNDPEHVAAKKDFLKVLK
GWTVAWDPEKMAPAFTINHKGPWNGGLLATAGNVIFQGLANGEFHAYDAT
NGNDLYSFPAQSAIIAPPVTYTANGKQYVAVEVGWGGIYPFLYGGVARTS
GWTVNHSRVIAFSLDGKDSLPPKNELGFTPVKPVPTYDEARQKDGYFMYQ
TFCSACHGDNAISGGVLPDLRWSGAPRGRESFYKLVGRGALTAY GMDRFD
TSMTPEQIEDIRNFIVKRANESYDDEVKARENSTGVPNDQFLNVPQSTAD
VPTADHP
> P35811|XYNC_FIBSU Endo -1,4-beta-xylanase C - Fibrobacter succinogenes (Bacteroides succinogenes).
MKTFSVTKSSVVFAMALGMASTAFAQDFCSNAQHSGQKVTITSNQTGKIG
DIGYELWDENGHGGSATFYSDGSMDCNITGAKDYLCRAG LSLGSNKTYKE
LGGDMIAEFKLVKSGAQNVGYSYIGIYGWMEGVSGTPSQLVEYYVIDNTL
ANDMPGSWIGNERKGTITVDGGTYIVYRNTRTGPAIKNSGNVTFYQYFSV
RTSPRDCGTINISEHMRQWEKMGLTMGKLYEAKVLGEAGNVNGEVRGGHM
DFPHAKVYVKNGSDPVSSSSVKSSSSTDAPKSSSSKGNGNVSGKIDACKD
VMGHEGKETRTQGQNNSSVTGNVGSSPYHYEIWYQGGNNS MTFYDNGTYK
ASWNGTNDFLARVGFKYDEKHTYEELGPIDAYYKWSKQGSAGGYNYIGIY
GWTVDPLVEYYIVDDWFNKPGANLLGQRKGEFTVDGDTYEIWQNTRVQQP
SIKGTQTFPQYFSVRKSARSCGHIDITAHMKKWEELGMKMGKMYEAKVLV
EAGGGSGSFDVTYFKMTDKAHPLAQPEPESSSSEAKVESSSSTVALHAAP
KMELKSGNFQVFDMQGRFLGTVKLDAGASVAQVLKANFKNA GIYMVKQGN
FMQRVAVK
> PROB_THETH Q60050 GLUTAMATE 5 -KINASE (EC 2.7.2.11) (GAMMA -G
MEFFGLPLRVEEGVLIPRPETEGLVELALGLPLPPAPRIARQVAALREEG
REVVLVSSGAVAAGMRRLGLKERPKDMPKKQALAALGQPLLMAFWQEAFA
PFGLPVAQVLLTAEDLSSRSRYLNAKATLRALLDLGAIPVINENDTVAFE
EIRFGDNDQLSARVAALVEAGL LALLSDVDALYEEDPKKNPQARPIPEVE
SVEAVLAHAGEENPLGSGGMKSKLLAARIAGRVGIPTLLLPGKRPGVLLQ
ALSGAPLGTYFHARRRYRGEKAWLFGLLRPKGELVLDRGAVRALKERGAS
LLPAGVKEVRGRFSRGEAVRLLSEEGEEVGVGLANYASEEIARIKGRRSA
EIEAVLGYRYTEEVVHRDHLALKEEA

> P26501|ISOA_PSEUM Isoamylase - Pseudomonas sp. (strain SMP1).
MKCPKILAALLGCAVLAGVPAMPAHAAINSMSLGASYDAQQANITFRVYS
SQATRIVLYLYSAGYGVQESATYTLSPAGSGVWAVTVPVSSIKAAGITGA
VYYGYRAWGPNWPYASNWGKGSQAGFVSDVDANGDRFNPNKLLLDPYAQE
VSQDPLNPSNQNGNVFASGASYRTTDSGIYAPKGVVLVPSTQSTGTKPTR
AQKDDVIYEVHVRGFTEQDTSIPAQYRGTYYGAGL KASYLASLGVTAVEF
LPVQETQNDANDVVPNSDANQNYWGYMTENYFSPDRRYAYNKAAGGPTAE
FQAMVQAFHNAGIKVYMDVVYNHTAEGGTWTSSDPTTATIYSWRGLDNTT
YYELTSGNQYFYDNTGIGANFNTYNTVAQNLIVDSLAYWANTMGVDGFRF
DLASVLGNSCLNGAYTASAPNCPNGGYNFDAADSNVAINRILREFTVRPA
AGGSGLDLFAEPWAIGGNSYQLGGFPQGWSEWNGLF RDSLRQAQNELGSM
TIYVTQDANDFSGSSNLFQSSGRSPWNSINFIDVHDGMTLKDVYSCNGAN
NSQAWPYGPSDGGTSTNYSWDQGMSAGTGAAVDQRRAARTGMAFEMLSAG
TPLMQGGDEYLRTLQCNNNAYNLDSSANWLTYSWTTDQSNFYTFAQRLIA
FRKAHPALRPSSWYSGSQLTWYQPSGAVADSNYWNNTSNYAIAYAINGPS
LGDSNSIYVAYNGWSSSVTFTLPAPPSGTQWYRVTDT CDWNDGASTFVAP
GSETLIGGAGTTYGQCGQSLLLLISK
> P31554|OSTA_ECOLI Organic solvent tolerance protein - Escherichia coli.
MKKRIPTLLATMIATALYSQQGLAADLASQCMLGVPSYDRPLVQGDTNDL
PVTINADHAKGDYPDDAVFTGSVDIMQGNSRLQADEVQLHQKEAPGQPEP
VRTVDALGNVHYDDNQVILKGPKGWANLNTKDTNVWEGD YQMVGRQGRGK
ADLMKQRGENRYTILDNGSFTSCLPGSDTWSVVGSEIIHDREEQVAEIWN
ARFKVGPVPIFYSPYLQLPVGDKRRSGFLIPNAKYTTTNYFEFYLPYYWN
IAPNMDATITPHYMHRRGNIMWENEFRYLSQAGAGLMELDYLPSDKVYED
EHPNDDSSRRWLFYWNHSGVMDQVWRFNVDYTKVSDPSYFNDFDNKYGSS
TDGYATQKFSVGYAVQNFNATVSTKQFQVFSEQNTSSYSA EPQLDVNYYQ
NDVGPFDTRIYGQAVHFVNTRDDMPEATRVHLEPTINLPLSNNWGSINTE
AKLLATHYQQTNLDWYNSRNTTKLDESVNRVMPQFKVDGKMVFERDMEML
APGYTQTLEPRAQYLYVPYRDQSDIYNYDSSLLQSDYSGLFRDRTYGGLD
RIASANQVTTGVTSRIYDDAAVERFNISVGQIYYFTESRTGDDNITWEND
DKTGSLVWAGDTYWRISERWGLRGGIQYDTRLDNVATSNSS IEYRRDEDR
LVQLNYRYASPEYIQATLPKYYSTAEQYKNGISQVGAVASWPIADRWSIV
GAYYYDTNANKQADSMLGVQYSSCCYAIRVGYERKLNGWDNDKQHAVYDN
AIGFNIELRGLSSNYGLGTQEMLRSNILPYQNTL
> P18473|TRBC_ECOLI Periplasmic protein trbC - Escherichia coli.
MKLSMKSLAALLMMLNGAVMASENVNTPENRQFLKQQENLSRQL REKPDH
QLKAWAEKQVLENPLQRSDNHFLDELVRKQQASQDGKPRQGALYFVSFSI
PEEGLKRMLGETRHFGIPATLRGMVNNDLKTTAEAVLSLVKDGATDGVQI
DPTLFSQYGIRTVPALVVFCSQGYDIIRGNLRVGQALEKVAATGDCRQVA
HDLLAGKGDSGK
> P12293|DHM1_PARDE Methanol dehydrogenase subunit 1 - Paracoccus
denitrificans.
MNRNTPKARGASSLAMAVAMGLAVLTTAPATANDQLVELAKDPANWVMTG
RDYNAQNYSEMTDINKENVKQLRPAWSFSTGVLHGHEGTPLVVGDRMFIH
TPFPNTTFALDLNEPGKILWQNKPKQNPTARTVACCDVVNRGLAYWPGDD
QVKPLIFRTQLDGHIVAMDAETGETRWIMENSDIKVGSTLTIAPYVIKDL
VLVGSSGAELGVRGYVTAYDVKSGEMRWRAFATGPDEELLLAEDFNAPNP
HYGQKNLGLETWEGDAWKIGGGTNWGWYAYDPEVDLFYYGSGNPAPWNET
MRPGDNKWTMAIWGREATTGEAKFAYQKTPHDEWDYAGVNVMMLSEQEDK
QGQMRKLLTHPDRNGIVYTLDRTNGDLISADKMDDTVNWVKEVQLDTGLP
VRDPEFGTRMDHKARDICPSAMGYHNQGHDSYDPERKVFMLGINHICMDW
EPFMLPYRAGQFFVGATLTMYPGPKATAERAGAGQIKAYDAISGEMKWEK
MERFSVWGGTMATAGGLTFYVTLDGFIKARDSDTGDLLWKFKLPSGVIGH
PMTYKHDGRQYVAIMYGVGGWPGVGLVFDLADPTAGLGSVGAFKRLQEFT
QMGGGVMVFSLDGESPYSDPNVGEYAPGEPT
> P19487|GUNA_XANCP Major extracellular endoglucanase - Xanthomonas
campestris pv. campestris.
MSIFRTASTLALATALALAAGPAFSYSINNS RQIVDDSGKVVQLKGVNVF
GFETGNHVMHGLWARNWKDMIVQMQGLGFNAVRLPFCPATLRSDTMPASI
DYSRNADLQGLTSLQILDKVIAEFNARGMYVLLDHHTPDCAGISELWYTG
SYTEAQWLADLRFVANRYKNVPYVLGLDLKNEPHGAATWGTGNAATDWNK
AAERGSAAVLAVAPKWLIAVEGITDNPVCSTNGGIFWGGNLQPLACTPLN
IPANRLLLAPHVYGPDVFVQSYFNDSNFPNNM PAIWERHFGQFAGTHALL

LGEFGGKYGEGDARDKTWQDALVKYLRSKGINQGFYWSWNPNSGDTGGIL
RDDWTSVRQDKMTLLRTLWGTAGNTTPTPTPTPTPTPTPTPTPTPTPTPG
TSTFSTKVIVDNSWNGGYCNRVQVTNTGTASGTWSIAVPVTGTVNNAWNA
TWSQSGSTLRASGVDFNRTLAAGATAEFGFCAAS
> P12634|FLAD_CAUCR Distal basal body ring compon ent protein - Caulobacter crescentus (Caulobacter vibrioides).
MKAFLFAAAATLVITALSAPAFAGTPVTLRMDTTDADGRITLGDLFDGVS
GPAANVVVAARMSATAVLEAGQVQMSARRAGYVWTNANGVRRIIVREGVD
NGGVSSSAAPGAQLAGARLAGAPRANVEVLAYARSLSAGEIVQPQDLIWV
KMAGAPADAPRDADAVIGLAAKRPLREGAPVGMKDVAAAQ VIKSGDLITI
TYEDGGISLSLQGKAMAAAAAGDVFAVQNTLSKKIIQAVAVGPGAAAVGP
QAQSLQARSQPLRFAAR
> P38501|NIR_ALCFA Copper -containing nitrite reductase - Alcaligenes faecalis.
MAEQMQISRRTILAGAALAGALAPVLATTSAWGQGAVRKATAAEIAALPR
QKVELVDPPFVHAHSQVAEGGPKVVEFTMVIEEKKIVIDDAGTEVH AMAF
NGTVPGPLMVVHQDDYLELTLINPETNTLMHNIDFHAATGALGGGGLTEI
NPGEKTILRFKATKPGVFVYHCAPPGMVPWHVVSGMNGAIMVLPREGLHD
GKGKALTYDKIYYVGEQDFYVPRDENGKYKKYEAPGDAYEDTVKVMRTLT
PTHVVFNGAVGALTGDKAMTAAVGEKVLIVHSQANRDTRPHLIGGHGDYV
WATGKFNTPPDVDQETWFIPGGAAGAAFYTFQQPGIYAYVNHNLIEA FEL
GAAAHFKVTGEWNDDLMTSVLAPSGT
> P37081|PTRB_KLEPN Sorbose -specific phosphotransferase enzyme IIB component - Klebsiella pneumoniae.
MQITLARIDDRLIHGQVTTVWSKVANAQRIIICNDDVFNDEVRRTLLRQA
APPGMKVNVVSLEKAVAVYHNPQYQDETVFYLFTNPHDVLTMVRQGVQIA
TLNIGGMAWRPGKKQLTKAVS LDPQDIQAFRELDKLGVKLDLRVVASDPS
VNILDKINETAFCE
> P41256|SYY_THIFE Tyrosyl -tRNA synthetase - Thiobacillus ferrooxidans (Acidithiobacillus ferrooxidans).
MTMKHQDAFEQIAFGTVDMLPEGEMLARLAAAQRDNRPLRIKLGMDPTAP
DLHLGAYVLLHKARQFQDLGHRLLFVIGDFTAMIGDPTGKSVTRKALSRE
EVVANAATYRPQVFKILDPERTEVMFNSEWLGALRPEELIQIAACYTVAR
MLERDDFNKRYSANQPIAIHEFLYPLLQGYDSVAIKADVELGGTDQRFNL
LVGRELQREYGQKPQLVLTMPILEGLDGVQKMSKSLGNFIAVEDPPAEMF
GKIMSISDFLMWRYYALLSRVPAVEQTRLQKEAASGARNPRDIKLDLAGE
LVRRFHGTAAAQEAHIAFLARFQRHETPEDLPLQAIKLSEAPRLSQLLVQ
VHLAASTSEAMRKMKEGAVRVDWRRVVDPATILALDAVYLLQFGKRHFAR
VALQKGE
> P77202|DSBG_ECOLI Thiol:disulfide interchange protein dsbG - Escherichia coli.
MLKKILLLALLPAIAFAEELPAPVKAIEKQGITIIKTFDAPGGMKGYLGK
YQDMGVTIYLTPDGKHAISGYMYNEKGENLSNTLIEKEIYAPAGREMWQR
MEQSHWLLDGKKDAPVIVY VFADPFCPYCKQFWQQARPWVDSGKVQLRTL
LVGVIKPESPATAAAILASKDPAKTWQQYEASGGKLKLNVPANVSTEQMK
VLSDNEKLMDDLGANVTPAIYYMSKENTLQQAVGLPDQKTLNIIMGNK
> P62722|VIRG_AGRT9 Regulatory protein virG - Agrobacterium tumefaciens (strain 15955).
MIVHPSRENFSSAVNKGSDFRLKGEPLKHVLLVDDD VAMRHLIIEYLTIH
AFKVTAVADSTQFTRVLSSATVDVVVVDLNLGREDGLEIVRNLAAKSDIP
IIIISGDRLEETDKVVALELGASDFIAKPFSIREFLARIRVALRVRPNVV
RSKDRRSFCFTDWTLNLRQRRLMSEAGGEVKLTAGEFNLLLAFLEKPRDV
LSREQLLIASRVRDEEVYDRSIDVLILRLRRKLEADPSSPQLIKTARGAG
YFFDADVQVSHGGTMAA
> Q07739|NODA_AZOCA Nodulation protein A - Azorhizobium caulinodans.
MMVHNYCAPENVTAPPHVSRVTQYNKRGIRMISKVTWRVAWESDLTNGDH
AELSDFFKSVYGATGAFNALPFAGGRSWAGARPELRGIAYDESGVAAHMG
VLRRFIKVGGEQIAVAELGLYGVRRDLEGLGIGHSTLAMLPVLKALGVPF
AFGCFRNELRIHFQRFCRNGKGAIVDNVNIKSTQPDIYPDLPPTKIEKKA
AVILPLTETLDRWPEGVDIERNGPEL
> HEM6_PSEAE P43898 COPROPORPHYRINOGEN III OXIDASE, AEROBIC (
MTDRIAAVKTYLLDLQDRICAALEAEDGKARFAEDAWERPAGGGGRTRVI
GDGALIEKGGVNFSHVFGDSLPPSASAHRPELAGRGFQALGVSLVIHPEN

PHVPTSHANVRFFCAEKEGEEPVWWFGGGFDLTPYYAHEEDCVHWHRVAR
DACAPFGADVYPRYKE WCDRYFHLKHRNEPRGIGGLFFDDLNQWDFDTCF
AFIRAIGDAYIDAYLPIVQRRKHTPFDERQREFQAYRRGRYVEFNLVFDR
GTLFGLQSGGRTESILMSLPPQVRWGYDWKPEPGSEEARLTEYFLADRDW
LAGQP
> P09961|AMY1_DICTH Alpha -amylase 1 - Dictyoglomus thermophilum.
MTKSIYFSLGIHNHQPVGNFDFVIERAYEMSYKPLINFFFKHPDFPIN VH
FSGFLLLWLEKNHPEYFEKLKIMAERGQIEFVSGGFYEPILPIIPDKDKV
QQIKKLNKYIYDKFGQTPKGMWLAERVWEPHLVKYIAEAGIEYVVVDDAH
FFSVGLKEEDLFGYYLMEEQGYKLAVFPISMKLRYLIPFADPEETITYLD
KFASEDKSKIALLFDDGEKFGLWPDTYRTVYEEGWLETFVSKIKENFLLV
TPVNLYTYMQRVKPKGRIYLPTASYREMMEWVLFPEAQKELEELVEKLK T
ENLWDKFSPYVKGGFWRNFLAKYDESNHMQKKMLYVWKKVQDSPNEEVKE
KAMEEVFQGQANDAYWHGIFGGLYLPHLRTAIYEHLIKAENYLENSEIRF
NIFDFDCDGNDEIIVESPFFNLYLSPNHGGSVLEWDFKTKAFNLTNVLTR
RKEAYHSKLSYVTSEAQGKSIHERWTAKEEGLENILFYDNHRRVSFTEKI
FESEPVLEDLWKDSSRLEVDSFYENYDYEINKDENKIRVLFSGVFRGFEL
CKSYILYKDKSFVDVVYEIKNVSETPISLNFGWEINLNFLAPNHPDYYFL
IGDQKYPLSSFGIEKVNNWKIFSGIGIELECVLDVEASLYRYPIETVSLS
EEGFERVYQGSALIHFYKVDLPVGSTWRTTIRFWVK
> P15321|HLYB_SERMA Hemolysin activator protein - Serratia marcescens.
MIKKITALTLLVSTALSAETLPDSHMMQDMSMGESRRALQDSTRE VNQLI
EQRRYQQLKQQRLLAEPAAPALPQSAQCLPIAGVYLQGVTLLSPADLSAL
SGLPEQCISSNDINRLTRELTRLYVQKGYITARVQIVRPNSQGELGLSVT
EGFIEKIEGGDRWVNSRLLFPGLEGKPLKLTELDQGLDQANRLQSNTTKL
DILPGRQVGGSVIRLRNQHAKPWLITAGTDNYGQKSTGRWLARATATLDS
PFGLSDFVSLNANSTLENPAHRYNRAYTLLYSLPYGAFTFSGFASF SSYE
NHQQLPHNVVKLHGQTQQYGLRSDYVFYRDHDQIDSLSGQLTYKRIDNYF
ESVRLEVSSPTLTLAELSASHLQILPNGVFSANLSVEQGMPWLGAGRHPS
SVHLDSQFTKGKLFANLSQRLRLGDATYQLNNLFYGQYSRDPLPGVEWLS
LTDRSAVRGFSRSTQSGDNGWYLQNTLSRSFNLGATTLTPRLGADVGRIL
PRQDNSGWRSSAGISTGATLRYQRALVDLEVSRGWILSNHATPEDPV QVL
ARFSYTF
> P16114|RNS_ECOLI Regulatory protein rns - Escherichia coli.
MDFKYTEEKETIKINNIMIHKYTVLYTSNCIMDIYSEEEKITCFSNRLVF
LERGVNISVRMQKQILSEKPYVAFRLNGDMLRHLKDALMIIYGMSKIDTN
ACRSMSRKIMTTEVNKTLLDELKNINSHDNSAFISSLIYLISKLENNEKI
IESIYISSVSFFSDKVRNLIEKDLSRKWT LGIIADAFNASEITIRKRLES
ENTNFNQILMQLRMSKAALLLLENSYQISQISNMIGISSASYFIRIFNKH
YGVTPKQFFTYFKGG
> P0A1Z2|SKP_SALTY Chaperone protein skp - Salmonella typhimurium.
MKKWLLAAGLGLAMVTSAQAADKIAIVNMGNLFQQVAQKTGVSNTLENEF
KGRAAELQKMETDLQSKMQRLQSMKAGSDRTKLEKDVMSQRQTFAQKAQ A
FEKDRARRSNEERNKLVTRIQTAVKKVANDQSIDLVVDANTVAYNSSDVK
DITADVLKQVK
> IDH_SYNY3 P80046 ISOCITRATE DEHYDROGENASE (NADP) (EC 1.1.1.
MYEKLQPPSVGSKITFVAGKPVVPNDPIIPYIRGDGTGVDIWPATELVIN
AAIAKAYGGREEINWFKVYAGDEACELYGTYQIFPEDTLTAIKEYGVAIK
GPLTTPVGGGIRSLNVALRQIFDLYTC VRPCRYYPGTPSPHKTPEKLDII
VYRENTEDIYLGIEWAEGTEGAKKLIAYLNDELIPTTPALGKKQIRLDSG
IGIKPISKTGSQRLVRRAILHAKRLPKAKQMVTLVHKGNIMKFTEGPFRD
WGYELATTEFRAECVTERESWICGNKESNPDLTIEANAHMIDPGYDTLTE
EKQAVIKQEVEQVLNSIWESHGNGQWKEKVMVNDRIADSIFQQIQTRPDE
YSILATMNLNGDYLSDAAAAVVGGLGMG PGANIGDSAAIFEATHGTAPKH
AGLDRINPGSVILSGVMMLEFMGWQEAADLIKKGIGAAIANREVTYDLAR
LMEPKVDKPLKCSEFAQAIVSHFDD
> P0AEQ3|GLNH_ECOLI Glutamine -binding periplasmic protein - Escherichia
coli.
MKSVLKVSLAALTLAFAVSSHAADKKLVVATDTAFVPFEFKQGDKYVGFD
VDLWAAIAKELKLDYELKPMDFSGIIP ALQTKNVDLALAGITITDERKKA
IDFSDGYYKSGLLVMVKANNNDVKSVKDLDGKVVAVKSGTGSVDYAKANI
KTKDLRQFPNIDNAYMELGTNRADAVLHDTPNILYFIKTAGNGQFKAVGD
SLEAQQYGIAFPKGSDELRDKVNGALKTLRENGTYNEIYKKWFGTEPK

> P43900|PHEA_HAEIN P-protein [Includes: Chorismate mutase - Haemophilus influenzae.
MALELSDIRQQITQIDRSLLKLLSERHRLAFDVVRSKEISQKSLRDVERE
QQLLQELVQFAENENYQLEAQYITSIFQKIIEDSVLTQQVYLQNKLNEQR
NQNLHIAFLGKRGSYSNLAARNYAARYQKQFVELGCQSFEQVFEKVQTGE
ADFGVLPLENTTSGAINEVYDLLQHTDLSLVGELAYPIKHCVLVNDKTDL
NQIDTLYSHPQVIQQCSQFIHSLDRVHIEYCESSSHAMQLVASLNKPN IA
ALGNEDGGKLYGLSVLKTNIANQENNITRFIVVAKEPREVSSQIPTKTLL
LMTTSQQAGALVDALLVFKKHQINMTKLESRPIYGKPWEEMFYLEIEANI
HHPDTKQALEELKNYSNYLKILGCYPSEIVKPVSV
> P0A9N4|PFLA_ECOLI Pyruvate formate-lyase 1-activating enzyme - Escherichia coli.
MSVIGRIHSFESCGTVDGPGIRFITFFQGCLM RCLYCHNRDTWDTHGGKE
VTVEDLMKEVVTYRHFMNASGGGVTASGGEAILQAEFVRDWFRACKKEGI
HTCLDTNGFVRRYDPVIDELLEVTDLVMLDLKQMNDEIHQNLVGVSNHRT
LEFAKYLANKNVKVWIRYVVVPGWSDDDDSAHRLGEFTRDMGNVEKIELL
PYHELGKHKWVAMGEEYKLDGVKPPKKETMERVKGILEQYGHKVMF
> P14768|XYNA_PSEFL Endo-1,4-beta-xylanase A - Pseudomonas fluorescens.
MRTAMAKSLGAAAFLGAALFAHTLAAQTATCSYNITNEWNTGYTGDITIT
NRGSSAINGWSVNWQYATNRLSSSWNANVSGSNPYSASNLSWNGNIQPGQ
SVSFGFQVNKNGGSAERPSVGGSICSGSVASSSAPASSVPSSIASSSPSS
VASSVISSMASSSPVSSSSVASSTPGSSSGNQQCNWYGTLYPLCVTTTNG
WGWEDQRSCIARSTCAA QPAPFGIVGSGSSTPVSSSSSSLSSSSVVSSIR
SSSSSSSSSVATGNGLASLADFPIGVAVAASGGNADIFTSSARQNIVRAE
FNQITAENIMKMSYMYSGSNFSFTNSDRLVSWAAQNGQTVHGHALVWHPS
YQLPNWASDSNANFRQDFARHIDTVAAHFAGQVKSWDVVNEALFDSADDP
DGRGSANGYRQSVFYRQFGGPEYIDEAFRRARAADPTAELYYNDFNTEEN
GAKTTALVNLVQRLLNNG VPIDGVGFQMHVMNDYPSIANIRQAMQKIVAL
SPTLKIKITELDVRLNNPYDGNSSNDYTNRNDCAVSCAGLDRQKARYKEI
VQAYLEVVPPGRRGGITVWGIADPDSWLYTHQNLPDWPLLFNDNLQPKPA
YQGVVEALSGR
> P0A9B6|E4PD_ECOLI D-erythrose-4-phosphate dehydrogenase - Escherichia coli.
MTVRVAINGFGRIGRNVVRALYESGRRAEIT VVAINELADAAGMAHLLKY
DTSHGRFAWEVRQERDQLFVGDDAIRVLHERSLQSLPWRELGVDVVLDCT
GVYGSREHGEAHIAAGAKKVLFSHPGSNDLDATVVYGVNQDQLRAEHRIV
SNASCTTNCIIPVIKLLDDAYGIESGTVTTIHSAMHDQQVIDAYHPDLRR
TRAASQSIIPVDTKLAAGITRFFPQFNDRFEAIAVRVPTINVTAIDLSVT
VKKPVKANEVNLLLQKAAQGAFHGIVDYTELP LVSVDFNHDPHSAIVDGT
QTRVSGAHLIKTLVWCDNEWGFANRMLDTTLAMATVAFR
> P44440|RNB_HAEIN Exoribonuclease 2 - Haemophilus influenzae.
MFQDNPLLAQLKQQIHDSKEQVEGVVKSTDKAYGFLECDKKTYFIAPPSM
KKVMHGDKIKATIEKQGDKEQAEPEALIEPMLTRFIAKVRFNKDKKLQVL
VDHPSINQPIGAQQAKSVKEELQEGDWVVANL KTHPLRDDRFFYATINQL
ICRADDELAPWWVTLARHEQSRYPVRGAEPYEMLDQKTRENLTALHFVTI
DSESTMDMDDALYIEPIAQNSTQTGWKLVVAIADPTAYIALDSQIEQEAK
QRCFTNYLPGFNIPMLPRELSDELCSLIANETRPALVCYIETDLTGNITA
KPHFVSAYVQSKAKLAYNKVSDYLEQADNAWQPEMPETAQQIHWLHQFTK
ARIQWRKTHSLFFKEKPDYAFVLAENGKVQEIK AEYRRIANQIVEEAMII
ANICAAQFLHEQAKTGIFNTHSGFDKKFLENAHNFLMANLANEQNQTELA
ERYSVENLATLNGYCQMRHDIEPIESDYLELRLRRYLTFAEFKSELAPHF
GLGLEGYATWTSPIRKYSDMVNHRLIKAVLAKQPYEKPQNDVLARLQEAR
RQNRLVERDIADWLYCRYLADKVASNAEFEAEVQDVMRAGLRVQLLENGA
SLFIPAATLHNNKEEIQLNPDELALYIKGERTYK IGDMVKVKLTEVKEAT
RSIVGEILQ
> P52111|GLPD_PSEAE Glycerol-3-phosphate dehydrogenase - Pseudomonas aeruginosa.
MSQAHTPSAPLAEVYDVAVVGGGINGVGIAADAAGRGLSVFLCEQHDLAQ
HTSSASSKLIHGGLRYLEHYEFRLVREALAEREVLLAKAPHIVKPLRFVL
PHRPHLRPAWMIRAGLFLYDHLGKREKLPASRGLRFTGSSPLKAEI RRGF
EYSDCAVDDARLVVLNAISAREHGAHVHTRTRCVSARRSKGLWHLHLERS
DGSLYSIRARALVNAAGPWVARFIQDDLKQKSPYGIRLIQGSHIIVPKLY
EGEHAYILQNEDRRIVFAIPYLDRFTMIGTTDREYQGDPAKVAISEEETA

YLLQVVNAHFKQQLAAADILHSFAGVRPLCDDESDEPSAITRDYTLSLSA
GNGEPPLLSVFGGKLTTYRKLAESALTQLQPFFANLGPAWTAKAPLP GGE
QMQSVEALTEQLANRYAWLDRELALRWARTYGTRVWRLLDGVNGEADLGE
HLGGGLYAREVDYLCKHEWAQDAEDILWRRSKLGLFLSPSQQVRLGQYLQ
SEHPHRPRVHAA
> P74368|RNC_SYNY3 Ribonuclease III - Synechocystis sp. (strain PCC 6803).
MNHPDFPPIGDPQLKLEALTHRSYCNEHPGTPSYDRLEFLGDAVLGFVVG
RILFERYPHFTEAELTRLRSQLVNQNQLAYLARFLHIAPEIRLSQSLARD
DGQSSPSILADVFESLLGAALLDRGLTAVEDFIQELFVPILEQWEKSQDG
RSPKLVPTMDVKSMLQQWALAKTKQLPEYELINTSGPPHAQEFTFTVKVA
GKIHGQGSGPSKQIATKQAALEALKSLGLLQ
> P19926|AGP_ECOLI Glucose-1-phosphatase - Escherichia coli.
MNKTLIAAAVAGIVLLASNAQ AQTVPEGYQLQQVLMMSRHNLRAPLANNG
SVLEQSTPNKWPEWDVPGGQLTTKGGVLEVYMGHYMREWLAEQGMVKSGE
CPPPYTVYAYANSLQRTVATAQFFITGAFPGCDIPVHHQEKMGTMDPTFN
PVITDDSAAFSEQAVAAMEKELSKLQLTDSYQLLEKIVNYKDSPACKEKQ
QCSLVDGKNTFSAKYQQEPGVSGPLKVGNSLVDAFTLQYYEGFPMDQVAW
GEIKSDQQWKVLSKLKNGYQDS LFTSPEVARNVAKPLVSYIDKALVTDRT
SAPKITVLVGHDSNIASLLTALDFKPYQLHDQNERTPIGGKIVFQRWHDS
KANRDLMKIEYVYQSAEQLRNADALTLQAPAQRVTLELSGCPIDADGFCP
MDKFDSVLNEAVK
> P0AES9|HDEA_ECOLI Protein hdeA - Escherichia coli.
MKKVLGVILGGLLLLPVVSNAADAQKAADNKKPVNSWTCEDFLAVDESFQ
PTAVGFAEALNNKDKPEDAVLDVQGIATVTPAIVQACTQDKQANFKDKVK
GEWDKIKKDM
> P73201|SYS_SYNY3 Seryl-tRNA synthetase - Synechocystis sp. (strain PCC
6803).
MLDLKQIRENPTAIQNRLNQRGGGASYDLEPILAIAAEQKAKESERTVLQ
SRSNEIGKLIGQKIGQGADPKGEEIQTLREEGNSLKIQLADLEPQEKDLK
EQLQKLLLELPNLPCETTP IGASEADNIEVKRWGDQYLKAETVGILPHWE
IGEKLGIIDSERGVKVAQSRFISLMKAGAALERALINFMLERHIGVGYQE
IMPPILVNSDSLLGTGQLPKFAEESFQCRGDDLWLIPTAEVPVTNLYRDE
VLDLEQLPIKHCAYTPCFRREAGSYGRDTKGLIRLHQFNKVELVKLVKPE
ESAAEHQALVADAEAILQALELPYRVVELCTGDLGFGAAKCYDLEVWLPS
ANTYREISSCSNFHDFQARR ANIRYKEKGKKGTQFVHTLNGSGLAIGRTM
AAILENYYEPSSGQVKVPVVLQDFLKRDYL
> FUCI_ECOLI P11552 L-FUCOSE ISOMERASE (EC 5.3.1.-). - ESCHER
MKKISLPKIGIRPVIDGRRMGVRESLEEQTMNMAKATAALLTEKLRHACG
AAVECVISDTCIAGMAEAAACEEKFSSQNVGLTITVTPCWCYGSETIDMD
PTRPKAIWGFNGTERPGAVYLAAALAAHSQ KGIPAFSIYGHDVQDADDTS
IPADVEEKLLRFARAGLAVASMKGKSYLSLGGVSMGIAGSIVDHNFFESW
LGMKVQAVDMTELRRRIDQKIYDEAELEMALAWADKNFRYGEDENNKQYQ
RNAEQSRAVLRESLLMAMCIRDMMQGNSKLADIGRVEESLGYNAIAAGFQ
GQRHWTDQYPNGDTAEAILNSSFDWNGVREPFVVATENDSLNGVAMLMGH
QLTGTAQVFADVRTYWSPEAIERVTGHKLDG LAEHGIIHLINSGSAALDG
SCKQRDSEGNPTMKPHWEISQQEADACLAATEWCPAIHEYFRGGGYSSRF
LTEGGVPFTMTRVNIIKGLGPVLQIAEGWSVELPKDVHDILNKRTNSTWP
TTWFAPRLTGKGPFTDVYSVMANWGANHGVLTIGHVGADFITLASMLRIP
VCMHNVEETKVYRPSAWAAHGMDIEGQDYRACQNYGPLYKR
> P48638|GSHR_ANASP Glutathione reductase - Anabaena sp. (strain PCC 7120).
MTFDYDLFVIGAGSGGLAASKRAASYGAKVAIAENDLVGGTCVIRGCVPK
KLMVYGSHFPALFEDAAGYGWQVGKAELNWEHFITSIDKEVRRLSQLHIS
FLEKAGVELISGRATLVDNHTVEVGERKFTADKILIAVGGRPIKPELPGM
EYGITSNEIFHLKTQPKHIAIIGSGYIGTEFAGIMRGLGSQVTQITRGDK
ILKGFDEDIRTEIQEGM TNHGIRIIPKNVVTAIEQVPEGLKISLSGEDQE
PIIADVFLVATGRVPNVDGLGLENAGVDVVDSSIEGPGYSTMNAIAVNEY
SQTSQPNIYAVGDVTDRLNLTPVAIGEGRAFADSEFGNNRREFSHETIAT
AVFSNPQASTVGLTEAEARAKLGDDAVTIYRTRFRPMYHSFTGKQERIMM
KLVVDTKTDKVLGAHMVGENAAEIIQGVAIAVKMGATKKDFDATVGIHPS
SAEEFVTMR
> ALKK_PSEOL Q00594 MEDIUM-CHAIN-FATTY-ACID--COA LIGASE (EC 6
MLGQMMRNQLVIGSLVEHAARYHGAREVVSVETSGEVTRSCWKEVELRAR
KLASALGKMGLTPSDRCATIAWNNIRHLEVYYAVSGAGMVCHTINPRLFI

EQITYVINHAEDKVVLLDDTFLPIIAEIHGSLPKVKAFVLMAHNNSNASA
QMPGLIAYEDLIGQGDDNYIWPDVDENEASSLCYTSGTTGNPKGVLYSH R
STVLHSMTTAMPDTLNLSARDTILPVVPMFHVNAWGTPYSAAMVGAKLVL
PGPALDGASLSKLIASEGVSIALGVPVVWQGLLAAQAGNGSKSQSLTRVV
VGGSACPASMIREFNDIYGVEVIHAWGMTELSPFGTANTPLAHHVDLSPD
EKLSLRKSQGRPPYGVELKIVNDEGIRLPEDGRSKGNLMARGHWVIKDYF
HSDPGSTLSDGWFSTGDVATIDSDGFMTICDRAKDIIKSGGEWISTVELE
SIAIAHPHIVDAAVIAARHEKWDERPLLIAVKSPNSELTSGEVCNYFADK
VARWQIPDAAIFVEELPRNGTGKILKNRLREKYGDILLRSSSSVCE
> P24059|CY552_BRAJA Cytochrome c -552 - Bradyrhizobium japonicum.
MHLHLRGICLVLAVASSSSSALAADAGHGADLAKRWCASCHVVANGQAVA
SADVPSFASVARRPDFSSEKLAFFLLDPHPKMPSFPLSRT EAGDIAAYIG
SLRP
> P08506|DACC_ECOLI Penicillin -binding protein 6 - Escherichia coli.
MTQYSSLLRGLAAGSAFLFLFAPTAFAAEQTVEAPSVDARAWILMDYASG
KVLAEGNADEKLDPASLTKIMTSYVVGQALKADKIKLTDMVTVGKDAWAT
GNPALRGSSVMFLKPGDQVSVADLNKGVIIQSGNDACIALADYVAGSQES
FIGLMNGYAKKLGLTNTT FQTVHGLDAPGQFSTARDMALLGKALIHDVPE
EYAIHKEKEFTFNKIRQPNRNRLLWSSNLNVDGMKTGTTAGAGYNLVASA
TQGDMRLISVVLGAKTDRIRFNESEKLLTWGFRFFETVTPIKPDATFVTQ
RVWFGDKSEVNLGAGEAGSVTIPRGQLKNLKASYTLTEPQLTAPLKKGQV
VGTIDFQLNGKSIEQRPLIVMENVEEGGFFGRVWDFVMMKFHQWFGSWFS

> P0AED0|USPA_ECOL I Universal stress protein A - Escherichia coli.
MAYKHILIAVDLSPESKVLVEKAVSMARPYNAKVSLIHVDVNYSDLYTGL
IDVNLGDMQKRISEETHHALTELSTNAGYPITETLSGSGDLGQVLVDAIK
KYDMDLVVCGHHQDFWSKLMSSARQLINTVHVDMLIVPLRDEEE
> P0AFI5|PBP7_ECOLI Penicillin -binding protein 7 - Escherichia coli.
MLIMPKFRVSLFSLALMLAVPFAPQAVAKTAAATTASQPEIASGSAMIVD
LNTNKVIYSNHPDLVRPIASISKLMTAMVVLDARLPLDEKLKVDISQTPE
MKGVYSRVRLNSEISRKDMLLLALMSSENRAAASLAHHYPGGYKAFIKAM
NAKAKSLGMNNTRFVEPTGLSVHNVSTARDLTKLLIASKQYPLIGQLSTT
REDMATFSNPTYTLPFRNTNHLVYRDNWNIQLTKTGFTNAAGH CLVMRTV
INNKPVALVVMDAFGKYTHFADASRLRTWIETGKVMPVPAAALSYKKQKA
AQMAAAGQTAQND
> P24228|PBP4_ECOLI Penicillin -binding protein 4 - Escherichia coli.
MRFSRFIIGLTSCIAFSVQAANVDEYITQLPAGANLALMVQKVGASAPAI
DYHSQQMALPASTQKVITALAALIQLGPDFRFTTTLETKGNVENGVLKGD
LVARFGADPTLK RQDIRNMVATLKKSGVNQIDGNVLIDTSIFASHDKAPG
WPWNDMTQCFSAPPAAAIVDRNCFSVSLYSAPKPGDMAFIRVASYYPVTM
FSQVRTLPRGSAEAQYCELDVVPGDLNRFTLTGCLPQRSEPLPLAFAVQD
GASYAGAILKDELKQAGITWSGTLLRQTQVNEPGTVVASKQSAPLHDLLK
IMLKKSDNMIADTVFRMIGHARFNVPGTWRAGSDAVRQILRQQAGVDIGN
TIIADGSGLSRHN LIAPATMMQVLQYIAQHDNELNFISMLPLAGYDGSLQ
YRAGLHQAGVDGKVSAKTGSLQGVYNLAGFITTASGQRMAFVQYLSGYAV
EPADQRNRRIPLVRFESRLYKDIYQNN
> P06111|OMPV_VIBCH Outer membrane protein ompV - Vibrio cholerae.
MKKIALFITASLIAGNALAAQTYIRNGNIYTHEGQWAAEVGAFGSTDLLK
DQDKSYGALLNFGYHGEDFNA DLSGLNYRFFGNTGDIVNLGTYLTGSGVA
YDQDSANSVKGMDKRKATVDLGLNADIALGDGTVSTYFQHDILNENKGYK
TGVNYFHIIDLGVADLVPFAGISYQSSDYNNYYFGVKDKEATAQRKAYHA
GGDFSYNLGYKLVYPINDRWEITQTSAYTRLGSDIAHSPIVDSANQWLVG
ATVAYHF
> P45523|FKBA_ECOLI FKBP -type peptidyl -prolyl cis-trans isomerase fkpA -
Escherichia coli.
MKSLFKVTLLATTMAVALHAPITFAAEAAKPATAADSKAAFKNDDQKSAY
ALGASLGRYMENSLKEQEKLGIKLDKDQLIAGVQDAFADKSKLSDQEIEQ
TLQAFEARVKSSAQAKMEKDAADNEAKGKEYREKFAKEKGVKTSSTGLVY
QVVEAGKGEAPKDSDTVVVNYKGTLIDGKEFDNSYTRGEPLSFRLDGVIP
GWTEGLKNIKKGGKIKLVIPPELAYG KAGVPGIPPNSTLVFDVELLDVKP
APKADAKPEADAKAADSAKK
> P19449|BCSA1_ACEXY Cellulose synthase catalytic subunit [UDP -forming] -
Acetobacter xylinus (Gluconacetobacter xylinus).

MSEVQSPVPAESRLDRFSNKILSLRGANYIVGALGLCALIAATTVTLSIN
EQLIVALVCVLVFFIVGRGKSRRTQIFLEVLSALVS LRYLTWRLTETLDF
DTWIQGGLGVTLLMAELYALYMLFLSYFQTIQPLHRAPLPLPDNVDDWPT
VDIFIPTYDEQLSIVRLTVLGALGIDWPPDKVNVYILDDGVRPEFEQFAK
DCGALYIGRVDSSHAKAGNLNHAIKRTSGDYILILDCDHIPTRAFLQIAM
GWMVADRKIALMQTPHHFYSPDPFQRNLAVGYRTPPEGNLFYGVIQDGND
FWDATFFCGSCAILRREAIESIGGFAVETVTEDAHTA LRMQRRGWSTAYL
RIPVASGLATERLTTHIGQRMRWARGMIQIFRVDNPMLGGGLKLGQRLCY
LSAMTSFFFAIPRVIFLASPLAFLFFGQNIIAASPLAVLAYAIPHMFHSI
ATAAKVNKGWRYSFWSEVYETTMALFLVRVTIITLMFPSKGKFNVTEKGG
VLEEEEFDLGATYPNIIFAGIMTLGLLIGLFELTFHFNQLAGIAKRAYLL
NCIWAMISLIILLAAIAVGRETKQVRYNHRVEAHIPVT VYEAPVAGQPNT
YHNATPGMTQDVSMGGVAVHMPWPDVSTGPVKTRIHAVLDGEEIDIPATM
LRCKNGKAVFTWDNNDLDTERDIVRFVFGRADAWLQWNNYEDDRPLRSLW
SLLLSIKALFRKKGKMMANSRPKRKPLALPVERREPTTIQSGQTQEGKIS
RAAS
> CHEZ_SALTY P07800 CHEMOTAXIS PROTEIN CHEZ. - SALMONELLA TYP
MMQPSIKPADEGSAGDIIARIGS LTRMLRDSLRELGLDQAIAEAAEAIPD
ARDRLDYVVQMTAQAAERALNSVEASQPHQDAMEKEAKALTQRWDEWFDN
PIELSDARELVTDTRQFLRDVPGHTSFTNAQLLDIMMAQDFQDLTGQVIK
RMMDVIQEIERQLLMVLLENIPEQSARPKRENESLLNGPQVDTSKAGVVA
SQDQVDDLLDSLGF
> P0AG82|PSTS_ECOLI Phosphate -binding periplasmic protein - Escherichia coli.
MKVMRTTVATVVAATLSMSAFSVFAEASLTGAGATFPAPVYAKWADTYQK
ETGNKVNYQGIGSSGGVKQIIANTVDFGASDAPLSDEKLAQEGLFQFPTV
IGGVVLAVNIPGLKSGELVLDGKTLGDIYLGKIKKWDDEAIAKLNPGLKL
PSQNIAVVRRADGSGTSFVFTSYLAKVNEEWKNNVGTGSTVKWPIGLGGK
GNDGIAAFVQRLPGAIGYVEYAYAKQNNLAYTKL ISADGKPVSPTEENFA
NAAKGADWSKTFAQDLTNQKGEDAWPITSTTFILIHKDQKKPEQGTEVLK
FFDWAYKTGAKQANDLDYASLPDSVVEQVRAAWKTNIKDSSGKPLY
> P27755|PRU_MYXXA Protein U - Myxococcus xanthus.
MNAIKTAVAAVTAAASLVAFSPAEAATATANLNVTANVGGACSIGSGAGG
GTLNFGTYDPVVVNSALGVDLFGTGSLSVQCTLLSTAVI TLGQGLYPAAG
STAAVPLRRMRNAASTDYLSYFLYMDVTRLIAWGNTSGTGLPFLGLGLPV
PVQVYGTVPRGQNVPSGTYNDTVVATITF
> P0C1A8|PMEA_ERWCH Pectinesterase A - Erwinia chrysanthemi.
MLKTISGTLALSLIIAASVHQAQAATTYNAVVSKSSSDGKTFKTIADAIA
SAPAGSTPFVILIKNGVYNERLTITRNNLLLKGESRNGAVIAAATAAGTL
KSDGSKWGTAGSSTITISAKDFSAQSLTIRNDFDFPANQAKSDSDSSKIK
DTQAVALYVTKSGDRAYFKDVSLVGYQDTLYVSGGRSFFSDCRISGTVDF
IFGDGTALFNNCDLVSRYRADVKSGNVSGYLTAPSTNINQKYGLVITNSR
VIRESDSVPAKSYGLGRPWHPTTTFSDGRYADPNAIGQTVFLNTSMDNHI
YGWDKMSGKDKNGNTIWFNPEDSRFFEYKSYGAGAAVSKDRRQLTDAQAA
EYTQSKVLGDWTPTLP
> P0AFM2|PROX_ECOLI Glycine betaine -binding periplasmic protein - Escherichia coli.
MRHSVLFATAFATLISTQTFAADLPGKGITVNPVQSTITEETFQTLLVSR
ALEKLGYTVNKPSEVDYNVGYTSLASGDATFTAVNWTPLHDNMYEAAGGD
KKFYREGVFVNGAAQGYLIDKKTADQYKITNIAQLKDPKIAKLFDTNGDG
KADLTGCNPGWGCEGAINHQLAAYELTNTVTHNQGNYAAMMADTISRYKE
GKPVFYYTWTPYWVSNELKPGKDVVWLQVPFSALPGDKNADTKLPNGANY
GFPVSTMHIVANKAWAEKNPAAAKLFAIMQLPVADINAQNAIMHDGKASE
GDIQGHVDGWIKAHQQQFDGWVNEALAAQK
> P14775|DHM2_METEX Methanol dehydrogenase subunit 2 - Methylobacterium extorquens (Protomonas extorquens).
MKTTLIAAAIVALSGLAAPALAYDGTKCKAAGNCWEPKPGFPEKIAGSKY
DPKHDPKELNKQADSIKQMEERNKKRVENFKKTGKFEYDVAKISAN
> P37921|FIMA1_SALTY Type -1 fimbrial protein, A chain - Salmonella typhimurium.
MKHKLMTSTIASLMFVAGAAVAADPTPVSVSGGTIHFEGKLVNAA CAVST
KSADQTVTLGQYRTASFTAIGNTTAQVPFSIVLNDCDPKVAANAAVAFSG
QADNTNPNLLAVSSADNSTTATGVGIEILDNTSSPLKPDGATFSAKQSLV

EGTNTLRFTARYKATAAATTPGQANADATFIMKYE
> P13717|NUCA_SERMA Nuclease - Serratia marcescens.
MRFNNKMLALAALLFAAQASADTLESIDNCAVGCPTGGSSNVSIVRHAYT
LNNNSTTKFANWVAYHITKDTPASGKTRNWKTDPALNPADTLAPADYTGA
NAALKVDRGHQAPLASLAGVSDWESLNYLSNITPQKSDLNQGAWARLEDQ
ERKLIDRADISSVYTVTGPLYERDMGKLPGTQKAHTIPSAYWKVIFINNS
PAVNHYAAFLFDQNTPKGADFCQFRVTVDEIEKRTGLIIWAGLPDDVQAS
LKSKPGVLPELMGCKN
> P32099|LPLA_ECOLI Lipoate-protein ligase A - Escherichia coli.
MSTLRLLISDSYDPWFNLAVEECIFRQMPATQRVLFLWRNADTVVIGRAQ
NPWKECNTRRMEEDNVRLARRSSGGGAVFHDLGNTCFTFMAGKPEYDKTI
STSIVLNALNALGVSAEASGRNDLVVKTVEGDRKVSGSAYRETKDRGFHH
GTLLLNADLSRLANYLNPDKKKLAAKGITSVRSRVTNLTELLPGITHEQV
CEAITEAFFAHYGERVEAEIISPNKTPDLPN FAETFARQSSWEWNFGQAP
AFSHLLDERFTWGGVELHFDVEKGHITRAQVFTDSLNPAPLEALAGRLQG
CLYRADMLQQECEALLVDFPEQEKELRELSAWMAGAVR
> P56154|PGK_HELPY Phosphoglycerate kinase - Helicobacter pylori
(Campylobacter pylori).
MLAKMSFMQNVKNIQEVEVSHKRVLIRVDFNVPLDENLNITDDTRIRESL
PTIQYCIDNKAKDIILVSHLGRPKGVEEKLSLKPFLKRLERLLNHEVVFS
QNIVQLKQALNENAPTRIFLLENIRFLRGEEENDENLAKDLASLCDVFVN
DAFGTSHRKHASTYGTAKFAPIKVSGFLLKKEIDSFYQAFNHPLRPLLLI
VGGAKVSSKLTLLKNILDLIDKLIIAGAMSNTFLKALGYDVQDSSVEDAL
INDALELLQSAKEKKVKVYLPIDAVTTDDILNPKHIKISPVQDIEPKHKI
ADIGPASLKLFSEVIESAPTILWNGPLGVHEKQEFARGTTFLAHKIADTY
AFSLIGGGDTIDAINRAGEKDNMSFISTGGGASLELLEGKILPCFEVLDK
RH
> P04164|CY552_THETH Cytochrome c-552 (Fragment) - Thermus thermophilus.
QADGAKIYAQCAGCHQQNGQGIPGAFPPLAGHVAEILAKEGGREYLILVL
LYGLQGQIEVKGMKYNGVMSSFAQLKDEEIAAVL NHIATAWGDAKKVKGF
KPFTAEEVKKLRAKKLTPQQVLAERKKLGLK
> Q55210|CY550_SYNP2 Cytochrome c-550 - Synechococcus sp. (strain PCC 7002)
(Agmenellum quadruplicatum).
MNKILGIDPLKKFIFGISAFVLLFWQLNVGAANATALREVDRTVNLNETE
TVVLSDQQVAKGERIFINTCSTCHNSGRTKSNPNVTLSLVDLEGAEPRRD
NILAMVDYLKNPTSYDVELDLSQLHPNTVRADIWSSMRNLNEEDLQNVSG
YVLVQAQVRGVAWGGGKTVN
> HMC6_DESVH P33393 52.7 KD PROTEIN IN HMC OPERON (ORF 6). -
MPEGKFCNRKPVNTEEDLKALLGDKGGAQYYKEMEELEVDQEALWANIEK
TCQSRTKTWLEICAHCGMCADSCFLYRVNDRDPKQVPAYKIQSTLGEIIR
RKGKVDTQFMLHAMEVAWSQ CTCCNRCGQYCPHGIDMGVMFSYLRGLLYS
QGFVPWELKIGSGMHRVYGAQMDVTTEDWVETCEWMAEEQQEEWPGLEIP
VDVENADIMYVLNAREPKHYPEDVAEAAILFHIAGENWTVPSEGWEQTSL
AMFAGDWAACKMQVERVYAAIERLKPKCVVGTECGHAHRASAIEGPYWAG
YEDGKTPAPWLHYVEWVAMALRTGKIKIDPEKRIKEPVTLQDSCNYIRNH
GLAKCTREIMSYIADDFREMT PNREHNYCCGGGGGFNGIGKFRKQRNKAL
QTKRDQILATGAKLVVAPCHNCWDAIRDLEEEYRIGIRWSFLKPLIIKMA
IIPEHLRPEEE
> FABB_HAEIN P43710 3-OXOACYL-[ACYL-CARRIER-PROTEIN] SYNTHASE
MRRTVITGFGIISSIGNNKEEVLASLKAGKSGIEVVPEFVEMNMRSHVAG
TIKLNPSEHIDRKVFRFMGDAAAYAYLSMREAIEDAGLTEDQVSNDRTGL
VIGAGTGSAHNQLVACDAVRGPRGVKAIGPYAVTKTMASSVSACLATPYK
IRGVNYSMSSACATSAHCIGHAVELIQLGKQDVVFAGGAEELSWECATEF
DAMGAVSTKYNETPEKASRAYDANRDGFVIAGGGAVVVVEELEHALARGA
KIYAEIVGYGATSDGYDMVAPSGEGAERCMKQAMATVDTPIDYINVHGTS
TPVGDVKELGAIKNVFGDKIPAISSTKSMTGHSLGAAGAHEAIYTLLMLD
NDFIAPSINIETLDEAAEGCNIVTETKENAGLQTVMSNSFGFGGTNATLI
FKRYNG
> HPRT_HAEIN P45078 HYPOXANTHINE PHOSPHORIBOSYLTRANSFERASE (E
MKKHHVDVLISENDVHARIAELGAQITKFYQEKQIDNLVVVGLLRGSFMF
MADIVRQINLPVEIEFMTTSSYGTGMTTNHDVRITKDLDGDIKGKHVLIV
EDIIDTGYTLEKVRDILNLREPASLTICTLLDKP SRREVEVPVEWVGFEI
PDEFVVGYGIDYAQRHRNLGYIGKVVLEE

> P07254|CHIA_SERMA Chitinase A - Serratia marcescens.
MRKFNKPLLALLIGSTLCSAAQAAAPGKPTIAWGNTKFAIVEVDQAATAY
NNLVKVKNAADVSVSWNLWNGDAGTTAKILLNGKEAWSGPSTGSSGTANF
KVNKGGRYQMQVALCNADGCTASDATEIVVADTDGSHLAPLKEPLLEKNK
PYKQNSGKVVGSYFVEWGVYGRNFTVDKIPAQNLTHLLYGFIPICGGNGI
NDSLKEIEGSFQALQRSCQGREDFKVSIHDPFAALQKAQKGVTAWDDPYK
GNFGQLMALKQAHPDLKILPSIGGWTLSDPFFFMGDKVKRDRFVGSVKEF
LQTWKFFDGVDIDWEFPGGKGANPNLGSPQDGETYVLLMKELRAMLDQLS
AETGRKYELTSAISAGKDKIDKVAYNVAQNSMDHIFLMSYDFYGPFDLKN
LGHQTALNAPAWKPDTAYTTVNGVNALLAQGVKPGKVVVGTAMYGRGWTG
VNGYQNNIPFTGTATGPVKGTWKNGIVDYRQIAGQFMSGEWQYTYDATAE
APYVFKPSTGDLITFDDARSVQAKGKYVLDKQLGGLFSWEIDADNGDILN
SMNASLGNSAGVQ
> P56118|RNC_HELPY Ribonuclease III - Helicobacter pylori (Campylobacter pylori).
MKNKRSQNSPYITPNSSYTTLEKALGYSFKDKRLLEQALTHKSCKLALNN
ERLEFLGDAVLGLVIGELLYHKFYQYDEGKLSKLRASIVSAHGFTKLAKA
IALQDYLRVSSSEEISNGREKPSILSSAFEALMAGVYLEAGLAKVQKIMQ
NLLNRAYKRLDLEHLFMDYKTALQELTQAQFCVIPTYQLLKEKGPDHHKE
FEMALYIQDKMYATAKGKSKKEAEQQCAYQALQKLKEAK
> Q56268|LEU3_THIFE 3-isopropylmalate dehydrogenase - Thiobacillus ferrooxidans (Acidithiobacillus ferrooxidans).
MKKIAIFAGDGIGPEIVAAARQVLDAVDQAAHLGLRCTEGLVGGAALDAS
DDPLPAASLQLAMAADAVILGAVGGPRWDAYPPAKRPEQGLLRLRKGLDL
YANLRPAQIFPQLLDASPLRPELVRDVDILVVRELTGDIYFGQPRGLEVI
DGKRRGFNTMVYDEDEIRRIAHVAFRAAQGRRKQLCSVDKANVLETTRLW
REVVTEVARDYPDVRLSHMYVDNAAMQLIRAPAQFDVLLTGNMFGDILSD
EASQLTGSIGMLPSASLGEGRAMYEPIHGSAPDIAGQDKANPLATILSVA
MMLRHSLNAEPWAQRVEAAVQRVLDQGLRTADIAAPGTPVIGTKAMGAAV
VNALNLKD
> P43902|TYRA_HAEIN T-protein [Includes: Chorismate mutase - Haemophilus influenzae.
MSFMEALKDLRSEIDSLDRELIQLFAKRLELVSQVGKVKHQHGLPIYAPE
REIAMLQARRLEAEKAGISADLIEDVLRRFMRESYANENQFGFKTINSDI
HKIVIVGGYGKLGGLFARYLRASGYPISILDREDWAVAESILANADVVIV
SVPINLTLETIERLKPYLTENMLLADLTSVKREPLAKMLEVHTGAVLGLH
PMFGADIASMAKQVVVRCDGRFPERYEWLLEQIQIWGAKIYQTNATEHDH
NMTYIQALRHFSTFANGLHLSKQPINLANLLALSSPIYRLELAMIGRLFA
QDAELYADIIMDKSENLAVIETLKQTYDEALTFFENNDRQGFIDAFHKVR
DWFGDYSEQFLKESRQLLQQANDLKQG
> P43834|SYV_HAEIN Valyl-tRNA synthetase - Haemophilus influenzae.
MTQKFEMADRFNPSAVEQALYQRWEESGYFKPSENENAPSYCIAIPPPNV
TGSLHMGHAFQQTLMDTLIRFNRMEGHNTLWQTGTDHAGIATQMVVERKI
AAEEGKTRHDYGREAFINKIWDWKAYSGGTISQQMRRLGNSIDWERERFT
MDDGLSNAVKEVFVRLHEEGLIYRGKRLVNWDPKLHTAISDLEVENKESK
GSLWHFRYPLANDAKTADGKDYLVVATTRPETMLGDTAVAVHPEDERYQS
LIGKTVVLPLANREIPIIADEYVDREFGTGVVKITPAHDFNDYEVGKRHN
LPMVNVLTLNANIRDEAEIIGTDGKPLAGYEATIPADYRGLERFAARKKI
VADFEALGLLDEIKPHDLKVPYGDRGGVPIEPMLTDQWYVSVKPLADVAI
KAVEDGEIQFVPKQYENLYFSWMRDIQDWCISRQLWWGHRIPAWYDAEGN
VYVARNEEEVRSKYNLDSAVELKQDEDVLDTWFSSGLWTFSTLGWPEQTK
ELKMFHPTDVLITGFDIIFFWVARMIMFTMHFVKDENGKPQVPFKTVYVT
GLIRDEQGQKMSKSKGNVLDPIDMIDGISLEDLLEKRTGNMMQPQLAEKI
AKATRKEFAEGIAAHGTDALRFTLAALASNGRDINWDMKRLEGYRNFCNK
LWNASRFVLTNEKLDLSQGEIEFSLADRWIQSEFNRTVETFRSSLSQYRF
DLCANAIYEFTWNQFCDWYLELTKPVFANGNAAQIRAASQTLVHVLEKLL
RLAHPLIPFITEEIWQKVKGFVGITADSIMLQPFPQVEESGFDPEAEAEI
EWLKEVIVAVRNIRAESNIAPSKGLDLLFRNLSAENAKILEKQTALLKAM
AKLDNVQVLATNETAPLAVAKLVGNAELLVPMAGFINKEAELARLTKEIE
KYQNEVKRIENKLSNEAFVAKAPEAVIAKEREKQAEYQSGLEKIQEQYKA
IEAL
> Q07703|METC_BORAV Cystathionine beta-lyase - Bordetella avium.
MSDTSAKHIDTLLQHLGSAPFNPDTGAAPVNLPSVRASTVRFQSLAKLED

AQRRKAAGERASTYGRMGMDTHAALEQVFAELEGGTHCYLASSGLAGISM
VFLSLLSAGEHALVADCAYGPVHELHEAVLSRLGIDVTFFDAKADLASLV
RPTTRLIFAEAPGSLLFEMLDMPALARFAKQHDLILATDNTWGSGYIYRP
LTLGAQVSVIAGTKYVGGHSDLMLGAVVTNDEAIAKRLNRTQYALGYSVS
ADDAWLALRGVRTM PVRMAQHARHALEVCEFLQNRPEVVRLYHPAWPADP
GHALWQRDCSGSNGMLAVQLGLSPQAARDFVNALTLFGIGFSWGGFESLV
QLVTPGELARHQYWQGGSDALVRLHIGLESPADLIADLAQALDRAA
> P0A9X9|CSPA_ECOLI Cold shock protein cspA - Escherichia coli.
MSGKMTGIVKWFNADKGFGFITPDDGSKDVFVHFSAIQNDGYKSLDEGQK
VSFTIESGAKGPAAGNVTSL
> P11797|CHIB_SERMA Chitinase B - Serratia marcescens.
MSTRKAVIGYYFIPTNQINNYTETDTSVVPFPVSNITPAKAKQLTHINFS
FLDINSNLECAWDPATNDAKARDVVNRLTALKAHNPSLRIMFSIGGWYYS
NDLGVSHANYVNAVKTPAARTKFAQSCVRIMKDYGFDGVDIDWEYPQAAE
VDGFIAALQEIRTLLNQQTIADGRQALPYQLTI AGAGGAFFLSRYYSKLA
QIVAPLDYINLMTYDLAGPWEKITNHQAALFGDAAGPTFYNALREANLGW
SWEELTRAFPSPFSLTVDAAVQQHLMMEGVPSAKIVMGVPFYGRAFKGVS
GGNGGQYSSHSTPGEDPYPNADYWLVGCDECVRDKDPRIASYRQLEQMLQ
GNYGYQRLWNDKTKTPYLYHAQNGLFVTYDDAESFKYKAKYIKQQQLGGV
MFWHLGQDNRNGDLLAALDRYFNAADYDDSQLDM GTGLRYTGVGPGNLPI
MTAPAYVPGTTYAQGALVSYQGYVWQTKWGYITSAPGSDSAWLKVGRLA
> P37060|NANH_VIBCH Sialidase - Vibrio cholerae.
MRFKNVKKTALMLAMFGMATSSNAALFDYNATGDTEFDSPAKQGWMQDNT
NNGSGVLTNADGMPAWLVQGIGGRAQWTYSLSTNQHAQASSFGWRMTTEM
KVLSGGMITNYYANGTQRVLPIISLDSSGNLVVEFEGQ TGRTVLATGTAA
TEYHKFELVFLPGSNPSASFYFDGKLIRDNIQPTASKQNMIVWGNGSSNT
DGVAAYRDIKFEIQGDVIFRGPDRIPSIVASSVTPGVVTAFAEKRVGGGD
PGALSNTNDIITRTSRDGGITWDTELNLTEQINVSDEFDFSDPRPIYDPS
SNTVLVSYARWPTDAAQNGDRIKPWMPNGIFYSVYDVASGNWQAPIDVTD
QVKERSFQIAGWGGSELYRRNTSLNSQQDWQSNAKIRIV DGAANQIQVAD
GSRKYVVTLSIDESGGLVANLNGVSAPIILQSEHAKVHSFHDYELQYSAL
NHTTTLFVDGQQITTWAGEVSQENNIQFGNADAQIDGRLHVQKIVLTQQG
HNLVEFDAFYLAQQTPEVEKDLEKLGWTKIKTGNTMSLYGNASVNPGPGH
GITLTRQQNISGSQNGRLIYPAIVLDRFFLNVMSIYSDDGGSNWQTGSTL
PIPFRWKSSSILETLEPSEADMVELQNGDLLLTARLDFNQ IVNGVNYSPR
QQFLSKDGGITWSLLEANNANVFSNISTGTVDASITRFEQSDGSHFLLFT
NPQGNPAGTNGRQNLGLWFSFDEGVTWKGPIQLVNGASAYSDIYQLDSEN
AIVIVETDNSNMRILRMPITLLKQKLTLSQN
> AMPL_RICPR P27888 CYTOSOL AMINOPEPTIDASE (EC 3.4.11.1) (LEU
MLNINFVNEESSTNQGLIVFIDEQLKLNNNLIALDQQHYELISKTIQNK L
QFSGNYGQITVVPSVIKSCAVKYLIIVGLGNVEKLTEAKIEELGGKILQH
ATCAKIATIGLKIINRINRFTSPTFTSLIASGAFLASYRFHKYKTTLKEV
EKFAVESIEILTDNNSEAMKLFEVKKLIAEAVFFTRDISNEPSNIKTPQV
YAERIVEILEPLGVNIDVIGEHDIKNLGMGALLGVGQGSQNESKLVVMEY
KGGSRDDSTLALVGKGVIFDTGGISLKPSSNMHLMRYDMAGSAAVVGTII
ALASQKVPVNVVGVVGLVENMQSGNAQRPGDVVVTMSGQTAEVLNTDAEG
RLVLADTVWYVQEKFNPKCVIDVATLTGAITVALGSTYAGCFSNNDELAD
KLIKAGEAVNEKLWRMPLHDDYDAMINSDIADIANIGNVPGAAGSCTAAH
FIKRFIKDGVDWAHLDIAGVANSNNASALCPKGAVGYGVRLLEKFIKEYN

> P00131|CYC3_DESVH Cytochrome c3 - Desulfovibrio vulgaris (strain Hildenborough / ATCC 29579 / NCIMB 8303).
MRKLFFCGVLALAVAFALPVVAAPKAPADGLKMEATKQPVVFNHSTHKSV
KCGDCHHPVNGKEDYRKCGTAGCHDSMDKKDKSAKGYYHVMHDKNTKFKS
CVGCHVEVAGADAAKKKDLTGCKKSKCHE
> P54737|PKN5_MYXXA Serine/threonine -protein kinase pkn5 - Myxococcus xanthus.
MPLKVIGPYRVLETLGSGGAGTVYRALDRRTTDEVALKLLSAGPARDARA
ARRLAREFDTLVDLSHPNVVKVFESGVHQGVPYLAMELIEGLTLRHYLDL
SSGDRQTPPGSHTPRSPLSVLRTADDDFGPLSRSFSDSMDDSEDSPFDGT
FGLEAFAEEAPSEDLESFASSASPHVGIGSDDSLEGFDLPPPMPRPAEPE
EEPGRVVREEDLNRPERMGRLKDAMLQICEALAYIHGH GLVHRDLKPSNI
MVDDDRQVRLMDFGLAKFLADDAAITEAGKLVGTYRYMAPEQILGEPLDG

RADLYSLGVILYELLSGRPPFDAKTPHELWRQVLETEPPPVLALNLHGDP
QLARVAHRLIRKEPDDRFQTAEEVYEALSE
> MASY_ECOLI P08997 MALATE SYNTHASE A (EC 4.1.3.2) (MSA). - E
MTEQATTTDELAFTRPYGEQEKQILTAEAVEFLTELVTHFTPQRNKLL AA
RIQQQQDIDNGTLPDFISETASIRDADWKIRGIPADLEDRRVEITGPVER
KMVINALNANVKVFMADFEDSLAPDWNKVIDGQINLRDAVNGTISYTNEA
GKIYQLKPNPAVLICRVRGLHLPEKHVTWRGEAIPGSLFDFALYFFHNYQ
ALLAKGSGPYFYLPKTQSWQEAAWWSEVFSYAEDRFNLPRGTIKATLLIE
TLPAVFQMDEILHALRDHIVGLNCGRWDYIFSYIKTLKNYPDRVLPDRQ A
VTMDKPFLNAYSRLLIKTCHKRGAFAMGGMAAFIPSKDEEHNNQVLNKVK
ADKSLEANNGHDGTWIAHPGLADTAMAVFNDILGSRKNQLEVMREQDAPI
TADQLLAPCDGERTEEGMRANIRVAVQYIEAWISGNGCVPIYGLMEDAAT
AEISRTSIWQWIHHQKTLSNGKPVTKALFRQMLGEEMKVIASELGEERFS
QGRFDDAARLMEQITTSDELIDFLTLPGYRLLA
> P11073|PELC_ERWCH Pectate lyase C - Erwinia chrysanthemi.
MKSLITPITAGLLLALSQPLLAATDTGGYAATAGGNVTGAVSKTATSMQD
IVNIIDAARLDANGKKVKGGAYPLVITYTGNEDSLINAAAANICGQWSKD
PRGVEIKEFTKGITIIGANGSSANFGIWIKKSSDVVVQNMRIGYLPGGAK
DGDMIRVDDSPNVWVDHNELFAANHECDGTPDNDTTFESAVDIKGASNTV
TVSYNYIHGVKKVGLDGSSSSDTGRNITYHHNYYNDVNARLPLQRGGLVH
AYNNLYTNITGSGLNVRQNGQALIENNWFEKAINPVTSRYDGKNFGTWVL
KGNNITKPADFSTYSITWTADTKPYVNADSWTSTGTFPTVAYNYSPVSAQ
CVKDKLPGYAGVGKNLATLTSTACK
> P33225|TORA_ECOLI Trimethylamine-N-oxide reductase 1 - Escherichia coli.
MNNNDLFQASRRRFLAQLGGLTVAGMLGPSLLTPRRATAAQAATDAVISK
EGILTGSHWGAIRATVKDGRFVAAKPFELDKYPSKMIAGLPDHVHNAARI
RYPMVRVDWLRKRHLSDTSQRGDNRFVRVSWDEALDMFYEELERVQKTHG
PSALLTASGWQSTGMFHNASGMLAKAIALHGNSVGTGGDYSTGAAQVILP
RVVGSMEVYEQQTSWPLVLQNSKTIVLWGSDLLKNQQANWWCPDHDVYEY
YAQLKAKVAAGEIEVISIDPVVTSTHEYLGREHVKHIAVNPQTDVPLQLA
LAHTLYSENLYDKNFLANYCVGFEQFLPYLLGEKDGQPKDAAWAEKLTGI
DAETIRGLARQMAANRTQIIAGWCVQRMQHGEQWAWMIVVLAAMLGQIGL
PGGGFGFGWHYNGAGTPGRKGVILSGFSGSTSIPPVHDNSDYKGYSSTIP
IARFIDAILEPGKVINWNGKSVKLPPLKMCIFAGTNPFHRHQQINRIIEG
LRKLETVIAIDNQWTSTCRFADIVLPATTQFERNDLDQYGNHSNRGIIAM
KQVVPPQFEARNDFDIFRELCRRFNREEAFTEGLDEMGWLKRIWQEGVQQ
GKGRGVHLPAFDDFWNNKEYVEFDHPQMFVRHQAFREDPDLEPLGTPSGL
IEIYSKTIADMNYDDCQGHPMWFEKIERSHGGPGSQKYPLHLQSVHPDFR
LHSQLCESETLRQQYTVAGKEPVFINPQDASARGIRNGDVVRVFNARGQV
LAGAVVSDRYAPGVARIHEGAWYDPDKGGEPGALCKYGNPNVLTIDIGTS
QLAQATSAHTTLVEIEKYNGTVEQVTAFNGPVEMVAQCEYVPASQVKS
> P74917|CY552_THIFE Cytochrome c-552 - Thiobacillus ferrooxidans
(Acidithiobacillus ferrooxidans).
MTTYLSQDRLRNKENDTMTYQHSKMYQSRTFLLFSALLLVAGQASAAVGS
ADAPAPYRVSSDCMVCHGMTGRDTLYPIVPRLAGQHKSYMEAQLKAYKDH
SRADQNGEIYMWPVAQALDSAKITALADYFNAQKPPMQSSGIKHAGAKEG
KAIFNQGVTNEQIPACMECHGSDGQGAGPFPRLAGQRYGYIIQQLTYFHN
GTRVNTLMNQIAKNITVAQMKDVAAYLSSL
> P43856|APT_HAEIN Adenine phosphoribosyltransferase - Haemophilus
influenzae.
MTTQLDLIKSSIKSIPNYPKEGIIFRDITTLLEVPAAFKATIDLIVEQYR
DKGITKVLGTESRGFIFGAPVALALGLPFELVRKPKKLPRETISQSYQLE
YGQDTLEMHVDAISEGDNVLIIDDLLATGGTVEATVKLVQRLGGAVKHAA
FVINLPELGGEKRLNNLGVDCYTLVNFEGH
> P18539|AMPC_SERMA Beta-lactamase - Serratia marcescens.
MTKMNRCAALIAALILPTAHAAQQQDIDAVIQPLMKKYGVPGMAIAVSVD
GKQQIYPYGVASKQTGKPITEQTLFEVGSLSKTFTATLAVYAQQQSKLSF
KDPASHYLPDVRGSAFDGVSLLNLATHTSGLPLFVPDDVTNNAQLMAYYR
AWQPKHPAGSYRVYSNLGIGMLGMIAAKSLDQPFIQAMEQGMLPALGMSH
TYVQVPAAQMANYAQGYSKDDKPVRVNPGPLDAESYGIKSNARDLIRYLD
ANLQQVKVASVARRWPRRTSVITSAGAFTQDLMWENYPYPVKLSRLIEGN
NAGMIMNGTPATAITPPQPELRAGWYNKTGSTGGFSTYAVFIPAKNIAVE
MLANKWFPNDDRVEAAYHIIQALEKR

> P0ABW5|SFMA_ECOLI Sfm fimbrial protein, A chain - Escherichia coli.
MKLRFISSALAAALFAATGSYAAVVDGGTIHFEGELVNAACSVNTDSADQ
VVTLGQYRTDIFNAVGNTSALIPFTIQLNDCD PVVAANAAVAFSGQADAI
NDNLLAIASSTNTTTATGVGIEILDNTSAILKPDGNSFSTNQNLIPGTNV
LHFSARYKGTGTSASAGQANADATFIMRYE
> P56071|SYT_HELPY Threonyl -tRNA synthetase - Helicobacter pylori
(Campylobacter pylori).
MSAELIAVYKDEQIIDLESAKVLGLSDGIKALNGTEPIYFDDSPLALEVI
RHSCAHLLAQSLKA LYPDAKFFVGPVVEEGFYYDFKTSSKISEEDLPKIE
AKMKEFAKLKLAITKETLTREQALERFKGDELKHAVMSKIGGDAFGVYQQ
GEFEDLCKGPHLPNTRFLNHFKLTKLAGAYLGGDENNEMLIRIYGIAFAT
KEGLKDYLFQIEEAKKRDHRKLGVELGLFSFDDEIGAGLPLWLPKGARLR
KRIEDLLSQALLLRGYEPVKGPEILKSDVWKISGHYDNYKENMYFTTIDE
QEYGIKPMNCVGHIK VYQSALHSYRDLPLRFYEYGVVHRHEKSGVLHGLL
RVREFTQDDAHIFCSFEQIQSEVSAILDFTHKIMQAFDFSYEMELSTRPA
KSIGDDKVWEKATNALKEALKEHRIDYKIDEGGGAFYGPKIDIKITDALK
RKWQCGTIQVDMNLPERFKLAFTNEYNHAEQPVMIHRAILGSFERFIAIL
SEHFGGNFPFFVAPTQIALIPINEEHHVFALKLKEALKKRDIFVEVLDKN
DSLNKKVRLAEKQKIP MILVLGNEEVETEILSIRDREKQDQYKMPLKEFL
NMVESKMQEVSF
> P00282|AZUR_PSEAE Azurin - Pseudomonas aeruginosa.
MLRKLAAVSLLSLLSAPLLAAECSVDIQGNDQMQFNTNAITVDKSCKQFT
VNLSHPGNLPKNVMGHNWVLSTAADMQGVVTDGMASGLDKDYLKPDDSRV
IAHTKLIGSGEKDSVTFDVSKLKEGEQYMFFCTFPGHSALMKGTLTLK
> P0C0V0|DEGP_ECOLI Protease do - Escherichia coli.
MKKTTLALSALALSLGLALSPLSATAAETSSATTAQQMPSLAPMLEKVMP
SVVSINVEGSTTVNTPRMPRNFQQFFGDDSPFCQEGSPFQSSPFCQGGQG
GNGGGQQQKFMALGSGVIIDADKGYVVTNNHVVDNATVIKVQLSDGRKFD
AKMVGKDPRSDIALIQIQNPKNLTAIKMADSDALRVGDYTVAIGNPFGLG
ETVTSGIVSALGRSGLNAENYENFIQTDAAINRGNSGGALVNLNGELIGI
NTAILAPDGGNIGIGFAIPSNMVKNLTSQMVEYGQVKRGELGIMGTELNS
ELAKAMKVDAQRGAFVSQVLPNSSAAKAGIKAGDVITSLNGKPISSFAAL
RAQVGTMPVGSKLTLGLLRDGKQVNVNLELQQSSQNQVDSSSIFNGIEGA
EMSNKGKDQGVVVNNVKTGTPAAQIGLKKGDVIIGANQQAVKNIAELRKV
LDSKPSVLALNIQRGDSTIYLLMQ
> Q55690|SYGB_SYNY3 Glycyl -tRNA synthetase beta chain - Synechocystis sp.
(strain PCC 6803).
MPLQTFLFEIGTEELPADFVRSAITQWQGLIPPGLAAEFLQPESVEIYGT
PRRLAVLIKGLPECQPDRLEEIKGPPASAAFKDGQPTPAALGFAKKQGVN
PEDFEIRSTPKGDFIFVNKQLQGQASRDLLPKLALSWLKAL DGRRFMRWG
DGDWRFPRPIRWLVCLLDDQVLPLQIDNGSTTLVGDRLSRGHRILHPADV
SLEHGQNYLAQLKTAGVVVDPQERRAMIEQQITTQAATLEGEAIIYEDLL
DEVEQLVEYPTAVLGKFDQEFLSLPREVTTTVMVTHQRYFPVVDKDGRLL
PHFITIANGDPSKGDIIAAGNGRVIRARLADAKFFYQADCDDSLDSYLPQ
LETVTFQEELGTMRDKVDRIMEMAAAIADQLGVTEQQRGEID STAMLCKA
DLVTQMVYEFPELQGIMGEKYALVSGESAAVAQGIVEHYLPRHQDDDLPQ
GLPGQVVGMADRLDTLVSIFGLGLLPTGSSDPFALRRAANAVINVAWAAS
LEINLLELLTQGCRDFVTSHPDKTSPLQALKTFFLQRLQTLLQDEQGIDY
DLVNAVLGNGETNCDEAQSRLHDRLLADLQDVKERAQYLQELRDNGHLDA
IYPTVNRSAKLASKGTLPTDQLDPRPVIQAPQLVQDSEKAVYQ ALLAIYP
KAVEVQESRDYETLVNALHELAPTVAEFFDGPDSVLVMAENDELRQNRLN
LLGLIRNYALILGDFGAIVKGI
> Q57142|C554_NITEU Cytochrome c -554 - Nitrosomonas europaea.
MKIMIACGLVAAALFTLTSGQSLAADAPFEGRKKCSSCHKAQAQSWKDTA
HAKAMESLKPNVKKEAKQKAKLDPAKDYTQDKDCVGCHVDGFGQKGGYTI
ESPKPMLTGV GCESCHGPGRNFRGDHRKSGQAFEKSGKKTPRKDLAKKGQ
DFHFEERCSACHLNYEGSPWKGAKAPYTPFTPEVDAKYTFKFDEMVKEVK
AMHEHYKLEGVFEGEPKFKFHDEFQASAKPAKKGK
> P32966|UVRC_PSEFL UvrABC system protein C - Pseudomonas fluorescens.
MTDPFDPSAFLSTCSGRPGVYRMFDSDTRLLYVGKAKNLKSRLASYFRKT
GLAPKTAALVGRIAQIETTITANETEALLLEQTLIKEWRPPYNILLRDDK
SYPYVFLSDGEFPRFSIHRGAKKQKGKYFGPYPSAGAIRESLSLLQKTFM
VRQCEDSYYKNRTRPCLQYQIKRCKAPCVGLVEAEVYAEDVRHSVMFLEG

RSNALTDELSAGMEQAASTLDFEKAAELRDQISLLRRVQDQQSMEGGSGD
VDVVAAFVNPGGACVHLISVRGGRVLGSKNFFPQVGIEEEVAEVMSAFLG
QYFLSSPERDLPSELIVNVVHEDFPALIAAIQELRGRELSISHRVRGTRA
RWQQLAVTNAEQALSARLANRQHVAARFEALAEVLNLDEPPQRLECYDIS
HSSGEATVASCVVFGPEGPLKSDYRRYNIEGVTAGDDYAAMHQALTRRFS
KLKDGEGKLPDILLVDGGKGQLSMARDVLNELAVPDLILLGVAKGATRKT
GFETLYLNDAAHEFTLKGDSPALHLIQQIRDEAHRFAITGHRARRGKTRR
TSTLEDVA GVGPKRRRDLLKHFGGLQELSRASIEEIAKAPGISKKLAELI
YANLHSE
> P20861|FANG_ECOLI Protein fanG - Escherichia coli.
MKKLYKAITVICILMSNLQSAQGATKSVQVPIRTEVKIPTCQLEIDSSID
FSFVKIEDIISSRATSKEANLNFRCDAHVDNVRIMFVPGSNRTSSDKRVM
HSGTTGLGYSLQWSRASSGYSDIGFNTQYQWSDSDAYQNLLSGKLRLKPV
SFPGESLSKEGKVSSTINIEVTYD
> Q52369|CYC4_PSEST Cytochrome c4 - Pseudomonas stutzeri (Pseudomonas perfectomarina).
MNKVLVSLLLTLGITGMAHAAGDAEAGQGKVAVCGACHGVDGNSPAPNFP
KLAGQGERYLLKQLQDIKAGSTPGAPEGVGRKVLEMTGMLDPLSDQDLED
IAAYFSSQKGSVGYADPALAKQGEKLFRGGKLDQGMPACTG CHAPNGVGN
DLAGFPKLGGQHAAYTAKQLTDFREGNRTNDGDTMIMRGVAAKLSNKDIE
ALSSYIQGLH
> P45069|FTSZ_HAEIN Cell division protein ftsZ - Haemophilus influenzae.
MLYPEYPEYDNFNESGALIKVVGVGGGGGNAVNHMVMNMVKQEMGGTFVG
ESSLTSEEHGRIVFYAVNTDAQALRKSQVQQTVQIGGETTKGLGAGANPN
IGRKAAEDDQDEIRKMLEGADMVFIAAGMGGGTGTGAAPVVAKIAKELGI
LTVAVVTKPFTFEGKKRMQFAELGIKDLSQYVDSMIIIPNQQIQKVLPKN
AKLIDAFAAANDVLRNSVMGISDMITSPGLINVDFADVRTVMSVQGQAMI
GFGSAVGEPGAGRAEEAARLAVRNDLLEKIDLSNAQGILVNITAGMDLVF
EEFNIIGETIGSFASEEATVVVGTSLVPEMSDEIRVTIVATGLGEIAGNE
PIQVVRQGLS TQNIEGEGRVNIVPELHRRESVEVSRTASEEYQRPLDKPI
TDRLEAFKKNNFFNPAQREEN
> P0ABZ6|SURA_ECOLI Chaperone surA - Escherichia coli.
MKNWKTLLLGIAMIANTSFAAPQVVDKVAAVVNNGVVLESDVDGLMQSVK
LNAAQARQQLPDDATLRHQIMERLIMDQIILQMGQKMGVKISDEQLDQAI
ANIAKQNNMTLDQMRSRLAYDGLNYNTYRNQIRKEM IISEVRNNEVRRRI
TILPQEVESLAQQVGNQNDASTELNLSHILIPLPENPTSDQVNEAESQAR
AIVDQARNGADFGKLAIAHSADQQALNGGQMGWGRIQELPGIFAQALSTA
KKGDIVGPIRSGVGFHILKVNDLRGESKNISVTEVHARHILLKPSPIMTD
EQARVKLEQIAADIKSGKTTFAAAAKEFSQDPGSANQGGDLGWATPDIFD
PAFRDALTRLNKGQMSAPVHSSFGWHLIELLDTRNVD KTDAAQKDRAYRM
LMNRKFSEEAASWMQEQRASAYVKILSN
> P52663|BLAN_ENTCL Imipenem -hydrolyzing beta -lactamase - Enterobacter cloacae.
MSLNVKQSRIAILFSSCLISISFFSQANTKGIDEIKNLETDFNGRIGVYA
LDTGSGKSFSYRANERFPLCSSFKGFLAAAVLKGSQDNRLNLNQIVNYNT
RSLEFHSPITTKYKDNGMSLGDMAAAALQYS DNGATNIILERYIGGPEGM
TKFMRSIGDEDFRLDRWELDLNTAIPGDERDTSTPAAVAKSLKTLALGNI
LSEHEKETYQTWLKGNTTGAARIRASVPSDWVVGDKTGSCGAYGTANDYA
VVWPKNRAPLIISVYTTKNEKEAKHEDKVIAEASRIAIDNLK
> P13063|PHSS_DESBA Periplasmic [NiFeSe] hydrogenase small subunit - Desulfovibrio baculatu s (Desulfomicrobium baculatus).
MSLSRREFVKLCSAGVAGLGISQIYHPGIVHAMTEGAKKAPVIWVQGQGC
TGCSVSLLNAVHPRIKEILLDVISLEFHPTVMASEGEMALAHMYEIAEKF
NGNFFLLVEGAIPTAKEGRYCIVGETLDAKGHHHEVTMMELIRDLAPKSL
ATVAVGTCSAYGGIPAAEGNVTGSKSVRDFFADEKIEKLLVNVPGCPPHP
DWMVGTLVAAWSHVLNPTEH PLPELDDDGRPLLFFGDNIHENCPYLDKYD
NSEFAETFTKPGCKAELGCKGPSTYADCAKRRWNNGINWCVENAVCIGCV
EPDFPDGKSPFYVAE
> P0C1A4|PELE2_ERWCH Pectate lyase E - Erwinia chrysanthemi.
MNNSRMSSVSTQKTTGRSALGTKSALAAIIATTMMVSVASAASLQTTKAT
EAASTGWATQSGGTTGGAKASSSKIYAVKSISEFKAALNGTDSSPK IIQV
TGAIDISGGKAYTSFDDQKARSQISIPSNTTIIGIGNKGKFTNGSLVVKG
VSNVILRNLYIETPVDVAPHYEEGDGWNAEWDAVVIDSTDHVWVDHVTIS

DGSFTDDKYTTKNGEKYVQHDGSLDIKRGSDYVTVSNSRFELHDKTILIG
HSDNNGSQDAGKLRVTFHNNLFDRVGERTPRVRFGSVHAYNNVYVGDVNH
KAYRYQYSFGIGTSGSLLSESNAFTIDNMKKISGRDKECSVVKAFNG KIF
SDKGSIINGASYNLNGCGFGFSAYSAKIPYKYSAQTITTSLAGSISSNAG
YGKL
> Q07740|NODB_AZOCA Chitooligosaccharide deacetylase - Azorhizobium caulinodans.
MSVLGQAARITQNQSSIYITFDDGPHPSVTPAVCEILREHSALTAFFQIG
RFTKEYPSISRQCQLDGHAIGNHTFDHPNLQDRAGEEVEYQISSAQKCLE
HICGRGFVRHFRA PYGAWSTQILNVVNKIGLRPVSWPVDPRDWEAPRIEN
LINEILDNARPSSIILLHDGCPPDEAAMWDVRGGRAQTLAALRYVVPALQ
ARGFALQPLP
> P31697|FIMC_ECOLI Chaperone protein fimC - Escherichia coli.
MSNKNVNVRKSQEITFCLLAGILMFMAMMVAGRAEAGVALGATRVIYPAG
QKQEQLAVTNNDENSTYLIQSWVENADGVKDGRFIVTPPLFA MKGKKENT
LRILDATNNQLPQDRESLFWMNVKAIPSMDKSKLTENTLQLAIISRIKLY
YRPAKLALPPDQAAEKLRFRRSANSLTLINPTPYYLTVTELNAGTRVLEN
ALVPPMGESTVKLPSDAGSNITYRTINDYGALTPKMTGVME
> P25394|FMF7_ECOLI F107 fimbrial protein - Escherichia coli.
MKRLVFISFVALSMTAGSAMAQQGDVKFFGNVSATTCNLTP QISGTVGDT
IQLGTVAPSGTGSEIPFALKASSNVGGCASLSTKTADITWSGQLTEKGFA
NQGGVANDSYVALKTVNGKTQGQEVKASNSTVSFDASKATTEGFKFTAQL
KGGQTPGDFQGAAAYAVTYK
> ARGJ_NEIGO P38434 GLUTAMATE N -ACETYLTRANSFERASE (EC 2.3.1.3
MAVNLTEKTAEQLPDIDGIALYTAQAGVKKPGHTDLTLIAVAAGSTVGAV
FTTNRFCAAP VHIAKSHLFDEDGVRALVINTGNANAGTGAQGRIDALAVC
AAAARQIGCKPNQVMPFSTGVILEPLPADKIIAALPKMQPAFWNEAARAI
MTTDTVPKAASREGKVGDQHTVRATGIAKGSGMIHPNMATMLGFIATDAK
VSQPVLQLMTQEIADETFNTITVDGDTSTNDSFVIIATGKNSQSEIDNIA
DPRYAQLKELLCSLALELAQAIVRDGEGATKFITVRVENAKTCDEARQAA
YAAARSPLVKT AFFASDPNLGKRLAAIGYADVADLDTDLVEMYLDDILVA
EHGGRAASYTEAQGQAVMSKDEITVRIKLHRGQAAATVYTCDLSHGYVSI
NADYRS
> P45356|HXUB2_HAEIN Heme/hemopexin utilization protein B - Haemophilus influenzae.
MKMRPRYSVIASAVSLGFVLSKSVMALGQPDTGSLNRELEQRQIQSEAKP
SGELFNQTANSPYTAQYKQGLKF PLTQVQILDRNNQEVVTDELAHILKNY
VGKEVSLSDLSNLANEISEFYRHNNYLVAKAILPPQEIEQGTVKILLLKG
NVGEIRLQNHSALSNKFVSRLSNTTVNTSEFILKDELEKFALTINDVPGV
NAGLQLSAGKKVGEANLLIKINDAKRFSSYVSVDNQGNKYTGRYRLAAGT
KVSNLNGWGDELKLDLMSSNQANLKNARIDYSSLIDGYSTRFGVTANYLD
YKLGGNFKSLQSQGHSHTLGAYLL HPTIRTPNFRLSTKVSFNHQNLTDKQ
QAVYVKQKRKINSLTAGIDGSWNLIKDGTTYFSLSTLFGNLANQTSEKKH
NAVENFQPKSHFTVYNYRLSHEQILPKSFAFNIGINGQFADKTLESSQKM
LLGGLSGVRGHQAGAASVDEGHLIQTEFKHYLPVFSQSVLVSSLFYDYGL
GKYYKNSQFLEQGVKNSVKLQSVGAGLSLSDAGSYAINVSVAKPLDNNIN
NADKHQFWLSMIKTF
> P29957|AMY_PSEHA Alpha -amylase - Pseudoalteromonas haloplanktis (Alteromonas haloplanktis).
MKLNKIITTAGLSLGLLLPSIATATPTTFVHLFEWNWQDVAQECEQYLGP
KGYAAVQVSPPNEHITGSQWWTRYQPVSYELQSRGGNRAQFIDMVNRCSA
AGVDIYVDTLINHMAAGSGTGTAGNSFGNKSFPIYSPQDFHESCTINNSD
YGNDRYRVQNCELVGLAD LDTASNYVQNTIAAYINDLQAIGVKGFRFDAS
KHVAASDIQSLMAKVNGSPVVFQEVIDQGGEAVGASEYLSTGLVTEFKYS
TELGNTFRNGSLAWLSNFGEGWGFMPSSSAVVFVDNHDNQRGHGGAGNVI
TFEDGRLYDLANVFMLAYPYGYPKVMSSYDFHGDTDAGGPNVPVHNNGNL
ECFASNWKCEHRWSYIAGGVDFRNNTADNWAVTNWWDNTNNQISFGRGSS
GHMAINKEDSTLTATVQTD MASGQYCNVLKGELSADAKSCSGEVITVNSD
GTINLNIGAWDAMAIHKNAKLNTSSASSTESDWQRTVIFINAQTQSGQDM
FIRGGIDHAYANANLGRNCQTSNFECAMPIRHNNLKNVTTSPWKANDNYL
DWYGIENGQSSEAEGSATDWTTNVWPAGWGAEKTVNTDGFGVTPLNIWGE
HYWMLDVDMDCSKAVNGWFELKAFIKNGQGWETAIAQDNAPYTSTNHMAQ
CGKINKFEFNNSGVVIRSF

> P24093|FMDR_ECOLI Dr hemagglutinin structural subunit - Escherichia coli.
MKKLAIMAAASMVFAVSSAHAGFTPSGTTGTTKLTVTEECQVRVGDLTVA
KTRGQLTDAAPIGPVTVQALGCDARQVALKADTDNFEQGKFFLISDNNRD
KLYVNIRPTDNSAWTTDNGVFYKNDVGSWGGIIGIYVDGQQTNTPPGNYT
LTLTGGYWAK
> P43859|XGPT_HAEIN Xanthine phosphoribosyltransferase - Haemophilus influenzae.
MSEKYVVTWDMFQMHARRLSERLLPASQWKGIIAVSRGGLFPAAVLAREL
GLRHIETVCIASYHDHNNQGELQVLHAAQVPNGGEGFIVVDDLVDTGNTA
RAIRQMYPNAKFVTVFAKPAGAELVDDYVIDIPQNTWIEQPWDLGLTFVP
PLSRK
> AMPR_CITDI P52658 TRANSCRIPTIONAL ACTIVATOR PROTEIN AMPR. -
MRSNLPLNALRAFEASARHLSFTRAALELCVTQAAVSQQVRILEDRLNRV
LFKRLPRGLEMTDEAQALFAVLTDAFGQIDTIFRQFEGGEYREVLTVAAV
GTFAVGWLLPRIEQFRQAHPFVELRLRTNNNVVNLAAEGLDFAIRFGNGL
WPATHNEMLFEAPLTVLCTPETAQRLRRPADLLQENLLRSYRVDEWDNWF
AAAGVTAERINGAVFDSSRLMVETVIHTGGAALVPAVMFARELAAGQLVR
PFDIEIQMGYWLTHLKSKPMTPAMEIFRDWIVKMA
> P06129|BTUB_ECOLI Vitamin B12 transporter btuB - Escherichia coli.
MIKKASLLTACSVTAFSAWAQDTSPDTLVVTANRFEQPRSTVLAPTTVVT
RQDIDRWQSTSVNDVLRRLPGVDITQNGGSGQLSSIFIRGTNASHVLVLI
DGVRLNLAGVSGSADLSQFPIALVQRVEYIRGPRSAVYGSDAIGGVVNII
TTRDEPGTEISAGWGSNSYQNYDVSTQQQLGDKTRVTLLGDYAHTHGYDV
VAYGNTGTQAQTDNDGFLSKTLYGALEHNFTDAWSGFVRGYGYDNRTNYD
AYYSPGSPLLDTRKLYSQSWDAGLRYNGELIKSQLITSYSHSKDYNYDPH
YGRYDSSATLDEMKQYTVQWANNVIVGHGSIGAGVDWQKQTTTPGTGYVE
DGYDQRNTGIYLTGLQQVGDFTFEGAARSDDNSQFGRHGTWQTSAGWEFI
EGYRFIASYGTSYKAPNLGQLYGFYGNPNLDPEKSKQWEGAFEGLTAGVN
WRISGYRNDVSDLIDYDDHTLKYYNEGKARIKGVEATANFDTGPLTHTVS
YDYVDARNAITDTPLLRRAKQQVKYQLDWQLYDFDWGITYQYLGTRYDKD
YSSYPYQTVKMGGVSLWDLAVAYPVTSHLTVRGKIANLFDKDYETVYGYQ
TAGREYTLSGSYTF
> P0AE22|APHA_ECOLI Class B acid phosphatase - Escherichia coli.
MRKITQAISAVCLLFALNSSAVALASSPSPLNPGTNVARLAEQAPIHWVS
VAQIENSLAGRPPMAVGFDIDDTVLFSSPGFWRGKKTFSPESEDYLKNPV
FWEKMNNGWDEFSIPKEVARQLIDMHVRRGDAIFFVTGRSPTKTETVSKT
LADNFHIPATNMNPVIFAGDKPGQNTKSQWLQDKNIRIFYGDSDNDITAA
RDVGARGIRILRASNSTYKPLPQAGAFGEEVIVNSEY
> P69059|MIP_LEGPN Outer membrane protein MIP - Legionella pneumophila.
MKMKLVTAAVMGLAMSTAMAATDATSLATDKDKLSYSIGADLGKNFKNQG
IDVNPEAMAKGMQDAMSGAQLALTEQQMKDVLNKFQKDLMAKRTAEFNKK
ADENKVKGEAFLTENKNKPGVVVLPSGLQYKVINAGNGVKPGKSDTVTVE
YTGRLIDGTVFDSTEKTGKPATFQVSQVIPGWTEALQLMPAGSTWEIYVP
SGLAYGPRSVGGPIGPNETLIFKIHLISVKKSS
> Q59516|DHGY_METEX Glycerate dehydrogenase - Methylobacterium extorquens (Protomonas extorquens).
MTKKVVFLDRESLDATVREFNFPHEYKEYESTWTPEEIVERLQGAEIAMI
NKVPMRADTLKQLPDLKLIAVAATGTDVVDKAAAKAQGITVVNIRNYAFN
TVPEHVVGLMFALRRAIVPYANSVRRGDWNKSKQFCYFDYPIYDIAGSTL
GIIGYGALGKSIAKRAEALGMKVLAFDVFPQDGLVDLETILTQSDVITLH
VPLTPDTKNMIGAEQLKKMKRSAILINTARGGLVDEAALLQALKDGTIGG
AGFDVVAQEPPKDGNILCDADLPNLIVTPHVAWASKEAMQILADQLVDNV
EAFVAGKPQNVVEA
> P49433|G3P1_SYNY3 Glyceraldehyde-3-phosphate dehydrogenase 1 - Synechocystis sp. (strain PCC 6803).
MLKIGINGFGRIGRLVARIAMANPQVTLVGINDLVPASNLAYLFKYDSTH
GSYGGTVVAKEEGIVIDDQFIPCFSQRNPAQLPWGDLGADYVVESTGLFT
TYATAENHLKAGAKRVIISAPSKDPEKIPTFVVGVNHLNYNADTDKIVSN
ASCTTNCLAPIAKILDDNFGIVEGLMTTVHAMTATQPTVDGPSKKDFRGG
RGAAQNIIPSSTGAAKAAALVLPQLKGKLTGMAFRVPTPNVSVVDLTFKT
EKATSYEEICAAMKTAAEGELKGILGYTADDVVSMDFRTDPRSSIFDAGA
GIGLNSNFFKVVSWYDNEWGYSCRVIDLMLTMASKDGLV
> P18956|GGT_ECOLI Gamma-glutamyltranspeptidase - Escherichia coli.

MIKPTFLRRVAIAALLSGSCFSAAAAPPAPPVSYGVEEDVFHPVRAKQGM
VASVDATATQVGVDILKEGGNAVDAAVAVGYALAVTHPQAGNLGGGGFML
IRSKNGNTTAIDFREMAPAKATRDMFLDDQGNPDSKKSLTSHLASGTPGT
VAGFSLALDKYGTMPLNKVVQPAFKLARDGFIVNDALADDLKTYGSEVLP
NHENSKAIFWKEGEPLKKGDTLVQANLAKSLEMIAENGPDEF YKGTIAEQ
IAQEMQKNGGLITKEDLAAYKAVERTPISGDYRGYQVYSMPPPSSGGIHI
VQILNILENFDMKKYGFGSADAMQIMAEAEKYAYADRSEYLGDPDFVKVP
WQALTNKAYAKSIADQIDINKAKPSSEIRPGKLAPYESNQTTHYSVVDKD
GNAVAVTYTLNTTFGTGIVAGESGILLNNQMDDFSAKPGVPNVYGLVGGD
ANAVGPNKRPLSSMSPTIVVKDGKTWLVTGSPGGSRIITTVLQ MVVNSID
YGLNVAEATNAPRFHHQWLPDELRVEKGFSPDTLKLLEAKGQKVALKEAM
GSTQSIMVGPDGELYGASDPRSVDDLTAGY
> P43927|SELB_HAEIN Selenocysteine -specific elongation factor - Haemophilus influenzae.
MIIVTSGHVDHGKTALLKALTGTSTAHLPEEKKRGMTIDLGYAYLPLENK
VLGFIDVPGHEKFLSNMLAGLGGVHYA MLIVAADEGVAVQTKEHLAILRQ
LQFHEIIVVITKADRTNSAQIESLIQTIKQDYSFLRNANYFVTSAETGQG
ISELRHYLANLAELADTQKPFRYAIDRVFSVKGAGTVVTGTAFSGTVKVN
DEIYLSTGQKIRIKAIHAQNTSSEQGIAGQRLALNLNADLDRTPMKRGDW
LLQNEPLPPTDRISVQILAEVPLNESQPVHIYHGASRTTGKLTLLQGKNA
AKNDRTLAEIILDSPLFLAFGDKLILRS GDTKTLIAGARVLEINSPKRHK
RTEVRLNFLANLALAENASQRIALTLQHNATTARQLMWTEQLTSLQLDKA
LAERDAVRYQDWCFNPNYVQEKTQQILTALNIYHEQHNDQLGVSKARLYR
MATLNQPENLIHHFIDEMLDDGRLQQTRGWIHLPEHKIQFNTEEKSRWTD
VLNEFEKANGQAIWVRDMANALAIDESIMRNFMYKAGKLGYLTPIVKDRF
FLTETLYAYARLIKQIAEEKGKVSVNEVR DKLNFGRKLTVQLMEYFDRMG
FLRRKGNDHILRDKNVFDL
> P0A9L7|PPIC_ECO57 Peptidyl -prolyl cis-trans isomerase C - Escherichia coli O157:H7.
MAKTAAALHILVKEEKLALDLLEQIKNGADFGKLAKKHSICPSGKRGGDL
GEFRQGQMVPAFDKVVFSCPVLEPTGPLHTQFGYHIIKVLYRN
> P54263|SYN_THET8 Asparaginyl -tRNA synthetase - Thermus thermophilus (strain HB8 / ATCC 27634 / DSM 579).
MRVFIDEIARHVDQEVELRGWLYQRRSKGKIHFLILRDGTGFLQATVVQG
EVPEAVFREADHLPQETALRVWGRVREDRRAPGGFELAVRDLQVVSRPQG
EYPIGPKEHGIDFLMDHRHLWLRHRRPFAVMRIRDELERAIHEFFGERGF
LRFDAPILTPSAVEGTTELFEVELFDGEK AYLSQSGQLYAEAGALAFAKV
YTFGPTFRAERSKTRRHLLEFWMVEPEVAFMTHEENMALQEELVSFLVAR
VLERRSRELEMLGRDPKALEPAAEGHYPRLTYKEAVALVNRIAQEDPEVP
PLPYGEDFGAPHEAALSRRFDRPVFVERYPARIKAFYMEPDPEDPELVLN
DDLLAPEGYGEIIGGSQRIHDLELLRRKIQEFGLPEEVYDWYLDLRRFGS
VPHSGFGLGLERTVAWICGLAHVREAIPFP RMYTRMRP
> P15922|PEHX_ERWCH Exo -poly-alpha-D-galacturonosidase - Erwinia chrysanthemi.
MKVITFSRRSALASIVATCLMSTPALAATAQAPQKLQIPTLSYDDHSVML
VWDTPEDTSNITDYQIYQNGQLIGLASQNNDKNSPAKPYISAFYKSDAAN
FHHRIVLQNAKVDGLKAGTDYQFTVRTVYADGTTSNDSNTVTTTTTAVPK
VINITQYGAKGDGTT LNTSAIQKAIDACPTGCRIDVPAGVFKTGALWLKS
DMTLNLLQGATLLGSDNAADYPDAYKIYSYVSQVRPASLLNAIDKNSSAV
GTFKNIRIVGKGIIDGNGWKRSADAKDELGNTLPQYVKSDNSKVSKDGIL
AKNQVAAAVATGMDTKTAYSQRRSSLVTLRGVQNAYIADVTIRNPANHGI
MFLESENVVENSVIHQTFNANNGDGVEFGNSQNIMVFNSVFDTGDDSINF
AAGMGQDAQKQEPSQN AWLFNNFFRHGHGAVVLGSHTGAGIVDVLAENNV
ITQNDVGLRAKSAPAIGGGAHGIVFRNSAMKNLAKQAVIVTLSYADNNGT
IDYTPAKVPARFYDFTVKNVTVQDSTGSNPAIEITGDSSKDIWHSQFIFS
NMKLSGVSPTSISDLSDSQFNNLTFSNLRSGSSPWKFGTVKNVTVDGKTV
TP
> P43820|SYFB_HAEIN Phenylalanyl -tRNA synthetase beta chain - Haemophilus influenzae.
MKFSEQWVREWVNPAVSTEQLCEQITMLGLEVDGVEAVAGTFNGVVVGEV
VECAQHPDADKLRVTKVNVGGDRLLDIVCGAPNCRQGLKVACATEGAVLP
GDFKIKKTKLRGQPSEGMLCSFSELGIDVEADGIIELPLDAPIGTDLREY
LALDDNAIEISLTPNRADCLSIAGIAREIGVVNKQLVNQLHFEAAPATIS

DKVQIDLQAPEACPRYLLRVIKNVNVKAPSP MWMQEKLRRCGIRSIDPIV
DITNYILLEFGQPMHAFDAAKVTQPVQVRFAKEGEELVLLDGSTAKLQSN
TLLIADQNGPLAMAGIFGGAASGVNSETKDVILESAFFAPLAIAGRARQY
GLHTDASHRFERGVDFELARKAMERATALLLEICGGEAGEICEASSETHL
PKVNTVQLRRSKLDALLGHHIETGSVTEIFHRLGFDVTYANDIWTVTSAS
WRFDIEIEEDLIEEVARIYGYNSIPNNAPLAH LCMREHKESDLDLARIKT
ALVDADYQEAITYSFVDPKIQSLLHPHQEALVLPNPISVEMSAMRVSLIS
GLLGAVLYNQNRQQSRVRLFETGLRFVPDANAEFGVRQEFVLSAVITGTA
KSEHWAGKAESVDFFDLKGDLESVLSLTEGGHRVRFVAKQFDALHPGQSA
AIELDGQEIGFIGAIHPSISQKLGLNGKTFVFEILWNAIAARNVVQAKEI
SKFPANRRDLALVVADSVPAGELIACKQAGGE KLVQVNLFDVYQGVGVA
EGYKSLAISLTVQDNEKTLEDEEINAVISAVLAEVKQRFNAELRD
> P43816|SYC_HAEIN Cysteinyl -tRNA synthetase - Haemophilus influenzae.
MLKIFNTLTREKEIFKPIHENKVGMYVCGVTVYDLCHIGHGRTFVCFDVI
ARYLRSLGYDLTYVRNITDVDDKIIKRALENKETCDQLVDRMVQEMYKDF
DALNVLRPDFEPRATHHIP EIIEIVEKLIKRGHAYVADNGDVMFDVESFK
EYGKLSRQDLEQLQAGARIEINEIKKNPMDFVLWKMSKENEPSWASPWGA
GRPGWHIECSAMNCKQLGEYFDIHGGGSDLMFPHHENEIAQSCCAHGGQY
VNYWIHSGMIMVDKEKMSKSLGNFFTIRDVLNHYNAEAVRYFLLTAHYRS
QLNYSEENLNLAQGALERLYTALRGTDQSAVAFGGENFVATFREAMDDDF
NTPNALSVLFEMAREINKLK TEDVEKANGLAARLRELGAILGLLQQEPEK
FLQAGSNDDEVAKIEALIKQRNEARTAKDWSAADSARNELTAMGIVLEDG
PNGTTWRKQ
> ACEA_ECOLI P05313 ISOCITRATE LYASE (EC 4.1.3.1) (ISOCITRASE
MKTRTQQIEELQKEWTQPRWEGITRPYSAEDVVKLRGSVNPECTLAQLGA
AKMWRLLHGESKKGYINSLGALTGGQALQQAKAGIEAVYLSGWQVAADAN
LAASMYPDQSLYPANSVPAVVERINNTFRRADQIQWSAGIEPGDPRYVDY
FLPIVADAEAGFGGVLNAFELMKAMIEAGAAAVHFEDQLASVKKCGHMGG
KVLVPTQEAIQKLVAARLAADVTGVPTLLVARTDADAADLITSDCDPYDS
EFITGERTSEGFFRTHAGIEQAISRGLAYAPYADLVWCETSTPDLELARR
FAQAIHAKYPGKLLAYNCSPSFNWQKNLDDKTIASFQQQLSDMGYKFQFI
TLAGIHSMWFNMFDLANAYAQGEGMKHYVEKVQQPEFAAAKDGYTFVSHQ
QEVGTGYFDKVTTIIQGGTSSVTALTGSTEESQF
> Q56143|SOXS_SALTY Regulatory protein soxS - Salmonella typhimurium.
MSHQQIIQTLIEWIDEHIDQPLNIDVVAKKSGYSKWYLQRMFRTVTHQTL
GEYIRQRRLLLAAVELRTTERPIFDIAMDLGYVSQQTFSRVFRREFDRTP
SDYRHRL
> HOXY_ALCEU P22319 NAD -REDUCING HYDROGENASE HOXS DELTA SUBUN
MRAPHKDEIASHELPATPMDPALAANREGKIKVATIGLCGCWGCTLSFLD
MDERLLPLLEKVTLLRSSLTDIKRIPERCAIGFVEGGVSSEENIETLEHF
RENCDILISVGACAVWGGVPAMRNVFELKDCLAEAYVNSATAVPGAKAVV
PFHPDIPRITTKVYPCHEVVKMDYFIPGCPPD GDAIFKVLDDLVNGRPFD
LPSSINRYD
> P36649|CUEO_ECOLI Blue copper oxidase cueO - Escherichia coli.
MQRRDFLKYSVALGVASALPLWSRAVFAAERPTLPIPDLLTTDARNRIQL
TIGAGQSTFGGKTATTWGYNGNLLGPAVKLQRGKAVTVDIYNQLTEETTL
HWHGLEVPGEVDGGPQGIIPPGGKRSVTLNVDQPAATCWFHPHQHGKTGR
QVAMGLAGLVVIEDDEILKLMLPKQWGIDDVPVIVQDKKFSADGQIDYQL
DVMTAAVGWFGDTLLTNGAIYPQHAAPRGWLRLRLLNGCNARSLNFATSD
NRPLYVIASDGGLLPEPVKVSELPVLMGERFEVLVEVNDNKPFDLVTLPV
SQMGMAIAPFDKPHPVMRIQPIAISASGALPDTLSSLPALPSLEGLTVRK
LQLSMDPMLDMMGMQMLMEKYGDQAMAGMDHSQMMGHMGHGNMNHMNHGG
KFDFHHANKINGQAFDMNKPMFAAAKGQYERWVISGVGDMMLHPFHIHGT
QFRILSENGKPPAAHRAGWKDTVKVEGNVSEVLVKFNHDAPKEHAYMAHC
HLLEHEDTGMMLGFTV
> P17989|GUB_FIBSU Beta -glucanase - Fibrobacter succinogenes (Bacteroides succinogenes).
MNIKKTAVKSALAVAAAAAALTTNVSAKDFSGAELYTLEEVQYGKFEARM
KMAAASGTVSSMFLYQNGSEIADGRPWVEVDIEVLGKNPGSFQSNIITGK
AGAQKTSEKHHAVSPAADQAFHTYGLEWTPNYVRWTVDGQEVRKTEGGQV
SNLTGTQGLRFNLWSSESAAWVGQFDESKLPLFQFINWVKVYKYTPGQGE
GGSDFTLDWTDNFDTFDGSRWGKGDWTFDGNRVDLTDKNIYSRDGMLILA
LTRKGQESFNGQVPRDDEPAPQSSSSAPASSSSVPASSSSVPASSSSAFV

PPSSSSATNAIHGMRTTPAVAKEHRNLVNAKGAKVNPNGHKRYRVNFEH
> P18317|UREE_KLEAE Urease accessory protein ureE - Klebsiella aerogenes.
MLYLTQRLEIPAAATASVTLPIDVRVKSRVKVTLNDGRDAGLLLPRGLLL
RGGDVLSNEEGTEFVQVIAADEEVSVVRCDDPFMLAKACYHLGNRHVPLQ
IMPGELRYHHDHVLDDMLRQFGLTVTFGQLPFEPEAGA YASESHGHHHAH
HDHHAHSH
> P09166|AERA_AERTR Aerolysin - Aeromonas trota.
MKALKITGLSLIISATLAAQTNAAEPIYPDQLRLFSLGEDVCGTDYRPIN
REEAQSVRNNIVAMMGQWQISGLANNWVILGPGYNGEIKPGKASTTWCYP
TRPATAEIPVLPAFNIPDGDAVDVQWRMVHDSANFIKPVSYLAHYLGYAW
VGGDHSQFVGDDMDVIQEGDDWVLRGNDGGKC DGYRCNEKSSIRVSNFAY
TLDPGSFSHGDVTQSERTLVHTVVGWATNISDTPQSGYDVTLNYTTMSNW
SKTNTYGLSEKVSTKNKFKWPLVGETEVSIEIAANQSWASQNGGAVTTAL
SQSVRPVVPARSRVPVKIELYKANISYPYEFKADMSYDLTFNGFLRWGGN
AWHTHPEDRPTLSHTFAIGPFKDKASSIRYQWDKRYLPGEMKWWDWNWAI
QQNGLATMQDSLARVLRPVRASITGDFRAESQF AGNIEIGTPVPLGSDSK
VRRTRSVDGANTGLKLDIPLDAQELAELGFENVTLSVTPARN
> Q04710|XYLS1_PSEPU XylDLEGF operon transcriptional activator 1 -
Pseudomonas putida.
MDFRLLNEKSQIFVHAEPYAVSDYVNQYVGTHSIRLPKGGRPAGRLHHRI
FGGLDLCRISYGGSVRVISPGLETCYHLQIILKGHCLWRDHGQEHYFAPG
ELLLLNPDDQADLTYSEDCEKFIVKLPSVVLDRACSENNWHKPREGIRFA
ARHNLQQLDGFINLLGLVCDEAEHTKSMPRVQEHYAGIIASKLLEMLGSN
VSREIFSKGNPSFERVVQFIEENLKRNISLERLAELAMMSPRSLYNLFEK
HAGTTPKNYIRNRKLESIRACLNDPSANVRSITEIALDYGFLHLGRFAEN
YRSAFGELPSDTLRQCKKEVA
> P05825|FEPA_ECOLI Ferrienterobacti n receptor - Escherichia coli.
MNKKIHSLALLVNLGIYGVAQAQEPTDTPVSHDDTIVVTAAEQNLQAPGV
STITADEIRKNPVARDVSKIIRTMPGVNLTGNSTSGQRGNNRQIDIRGMG
PENTLILIDGKPVSSRNSVRQGWRGERDTRGDTSWVPPEMIERIEVLRGP
AAARYGNGAAGGVVNIITKKGSGEWHGSWDAYFNAPEHKEEGATKRTNFS
LTGPLGDEFSFRLYGNLDKTQ ADAWDINQGHQSARAGTYATTLPAGREGV
INKDINGVVRWDFAPLQSLELEAGYSRQGNLYAGDTQNTNSDSYTRSKYG
DETNRLYRQNYALTWNGGWDNGVTTSNWVQYEHTRNSRIPEGLAGGTEGK
FNEKATQDFVDIDLDDVMLHSEVNLPIDFLVNQTLTLGTEWNQQRMKDLS
SNTQALTGTNTGGAIDGVSTTDRSPYSKAEIFSLFAENNMELTDSTIVTP
GLRFDHHSIVGNNWSPALNISQ GLGDDFTLKMGIARAYKAPSLYQTNPNY
ILYSKGQGCYASAGGCYLQGNDDLKAETSINKEIGLEFKRDGWLAGVTWF
RNDYRNKIEAGYVAVGQNAVGTDLYQWDNVPKAVVEGLEGSLNVPVSETV
MWTNNITYMLKSENKTTGDRLSIIPEYTLNSTLSWQAREDLSMQTTFTWY
GKQQPKKYNYKGQPAVGPETKEISPYSIVGLSATWDVTKNVSLTGGVDNL
FDKRLWRAGNAQTTGDLAGANYI AGAGAYTYNEPGRTWYMSVNTHF
> CYAA_HAEIN P40134 ADENYLATE CYCLASE (EC 4.6.1.1) (ATP PYROP
MECNLAQAKQWVSALDQRRFERALQGSGDAFQHVLAIVPLLLHLNHPQLP
GYVIHAPSGIASFLASDYQKKWLTNEYGIHYADHKPSTLKSAVNFHEVFP
PILGVYVMGSFGSISQTSSSDLDTWICVRDGLSLDEYTLLTQKAKRISEW
AMQFNVEINFYLMDQQR FRNEHYADPLTIENSGSAQYMLLLDEFYRSAVR
LAGKPLLWLHLWVENEKDYEKEVARLITEGEIDPNDWVDFGGLGQFSANE
YFGASLWHLYKGIDSPYKSVLKILLLEAYSKEYPNTCLIARTFKRDLLAG
NTNPDHHFDPYIAILAKVTQYLTALSEFKRLDFVHRCFYVKATEDFARYQ
ANNWRIRYMEILAQEWGWSAETVKHLNKRPFWKIKAVKENHDNIMKFLML
SYRNLVEFARKHHIHSSV VPQDINILSRKLYTAFEELPGKVSLLNTQISH
NLSEAHLTFVEVRGNKHFKDGWYLINQPIHHIMFSKERVIEYGESLNKLV
SWAYFNHLLTEKTELSIFSKNVTLSTLQRFVTNLRQSFPSTIAKQPKNSD
LLNQCEIRSLFIAINLTTDPTSKVEEVLTGISSRDLFSFGSLEQSLVGSI
DFTYRNVWNEIRTLHFEGQNAILLALKVLSNKIYRGVNRPDSIQVYCYSE
RYRQDLRQLVMGLVNRCVS IQVGDIQQPCQTSRLRVAGKNWQLFFEDRGI
SLQEIGNESVCNEAESAVDFDEVLQTPIEDGETNQESRRYPPEMDAFASE
GFLQFFFEDNSDHSFNVYILDESNHLEIYRHCDGEKDEKVREINQLYQNA
KQEGDKNPYNIVQHNFNYPQFYQLQNGKNGISIVPFKFRQMNK
> P27033|GUNC_PSEFL Endoglucanase C - Pseudomonas fluorescens.
MGHVTSPSKRYPASF KRAGSILGVSIALAAFSNVAAAGCEYVVTNSWGSG
FTAAIRITNSTSSVINGWNVSWQYNSNRVTNLWNPNLSGSNPYSASNLSW

NGTIQPGQTVEFGFQGVTNSGTVESPTVNGAACTGGTSSSVSSSSVVSSS
SSSRSSVSSSSVVSSSSSVVSSSSSSVVSGGGQCNWYGTLYPLCVSTTSG
WGYENNRSCISPSTCSAQPAPYGIVGGSSSPSSISSSSVRSSSSSSVVPP
SSSSSSSVPSSSCSSV SSSSVVSSSSSSVSVPGTGVFRVNTKGNLTKDGQ
LLPARCGNWFGLEGRHEPSNDADNPSGAPMELYAGNMWWVNNSQGSGRTI
QQTMTELKQQGITMLRLPIAPQTLDANDPQGRSPNLKNHQSIRQSNARQA
LEDFIKLADQNDIQIFIDIHSCSNYVGWRAGRLDARPPYVDANRVGYDFT
REEYSCSATNNPSSVTRIHAYDKQKWLANLREIAGLSAKLGVSNLIGIDV
FNEPYDYTWAEWKGMVE EAYQAINEVNPNMLIIVEGISANANTQDGTPDT
SVPVPHGSTDLNPNWGENLYEAGANPPNIPKDRLLFSPHTYGPSVFVQRQ
FMDPAQTECAGLEGDEAAQARCRIVINPTVLEQGWEEHFGYLRELGYGIL
IGEFGGNMDWPGAKSSQADRNAWSHITTNVDQQWQQAAASYFKRKGINAC
YWSMNPESADTMGWYLTPWDPVTANDMWGQWTGFDPRKTQLLHNMWGL
> P0A8L1|SYS_ECOLI S eryl-tRNA synthetase - Escherichia coli.
MLDPNLLRNEPDAVAEKLARRGFKLDVDKLGALEERRKVLQVKTENLQAE
RNSRSKSIGQAKARGEDIEPLRLEVNKLGEELDAAKAELDALQAEIRDIA
LTIPNLPADEVPVGKDENDNVEVSRWGTPREFDFEVRDHVTLGEMHSGLD
FAAAVKLTGSRFVVMKGQIARMHRALSQFMLDLHTEQHGYSENYVPYLVN
QDTLYGTGQLP KFAGDLFHTRPLEEEADTSNYALIPTAEVPLTNLVRGEI
IDEDDLPIKMTAHTPCFRSEAGSYGRDTRGLIRMHQFDKVEMVQIVRPED
SMAALEEMTGHAEKVLQLLGLPYRKIILCTGDMGFGACKTYDLEVWIPAQ
NTYREISSCSNVWDFQARRMQARCRSKSDKKTRLVHTLNGSGLAVGRTLV
AVMENYQQADGRIEVPEVLRPYMNGLEYIG
> P21338|RNI_ECOLI Ribonuclease I - Escherichia coli.
MKAFWRNAALLAVSLLPFSSANALALQAKQYGDFDRYVLALSWQTGFCQS
QHDRNRNERDECRLQTETTNKADFLTVHGLWPGLPKSVAARGVDERRWMR
FGCATRPIPNLPEARASRMCSSPETGLSLETAAKLSEVMPGAGGRSCLER
YEYAKHGACFGFDPDAYFGTMVRLNQEIKESEAGKFLADNYGKTVSRRDF
DAAFAKSWGKENVKAVKLTCQGNPAYLTEI QISIKADAINAPLSANSFLP
QPHPGNCGKTFVIDKAGY
> P43831|SYQ_HAEIN Glutaminyl -tRNA synthetase - Haemophilus influenzae.
MMSHTETSLGAENTRTHNFITQIIDEDLASGKHKSVHTRFPPEPNGYLHI
GHAKSICLNFGLAKEYQGLCNLRFDDTNPVKEDVEYVDSIKADVEWLGFK
WEGEPRYASDYFDALYGYAVELIKKGLAYVDELSPDEMREYR GTLTEPGK
NSPYRDRTIEENLALFEKMKNGEFAEGKASLRAKIDMASPFMVMREPVIY
RIKFSSHHQTGDKWCIYPMYDFTHCISDAIERITHSICTLEFQDNRRLYD
WVLENISIERPLPHQYEFSRLNLEGTLTSKRKLLKLVNDEIVDGWNDPRM
PTISGLRRRGYTPASLREFCRRIGVTKQDNVVEYSALEACIREDLNENAP
RAMAVIDPVRVVIENFESEAVLTAPNHPNRPELGERQLPFTKE LYIDRAD
FREEANKQYKRLVLGKEVRLRNAYVIKAERVEKDANGEITTIFCTYDPET
LGKNPADGRKVKGVIHWVSAVNNHPAEFRLYDRLFTVPNPGAEDDIESVL
NPNSLVIKQGFVEQSLANAEAEKGYQFEREGYFCADSKDSRPEHLVFNLT
VSLKEGF
> P00964|GLNA_ANASP Glutamine synthetase - Anabaena sp. (strain PCC 7120).
MTTPQEVLKRIQ DEKIELIDLKFIDTVGTWQHLTLYQNQIDESSFSDGVP
FDGSSIRGWKAINESDMTMVLDPNTAWIDPFMEVPTLSIVCSIKEPRTGE
WYNRCPRVIAQKAIDYLVSTGIGDTAFFGPEAEFFIFDSARFAQNANEGY
YFLDSVEGAWNSGKEGTADKPNLAYKPRFKEGYFPVSPTDSFQDIRTEML
LTMAKLGVPIEKHHHEVATGGQCELGFRFGKLIEAADWLMIYKYVIKNVA
KKYGKTVTFMPKP IFGDNGSGMHCHQSIWKDGKPLFAGDQYAGLSEMGLY
YIGGLLKHAPALLAITNPSTNSYKRLVPGYEAPVNLAYSQGNRSASIRIP
LSGTNPKAKRLEFRCPDATSNPYLAFAAMLCAGIDGIKNKIHPGEPLDKN
IYELSPEELAKVPSTPGSLELALEALENDHAFLTDTGVFTEDFIQNWIDY
KLANEVKQMQLRPHPYEFSIYYDV
> P37321|BLE1_PSEAE Extended -spectrum beta-lactamase PER-1 - Pseudomonas aeruginosa.
MNVIIKAVVTASTLLMVSFSSFETSAQSPLLKEQIESIVIGKKATVGVAV
WGPDDLEPLLINPFEKFPMQSVFKLHLAMLVLHQVDQGKLDLNQTVIVNR
AKVLQNTWAPIMKAYQGDEFSVPVQQLLQYSVSHSDNVACDLLFELVGGP
AALHDYIQSMGIKETAVVANEAQMHADDQVQYQNWTSMKGAAEILKKFEQ
KTQLSETSQALLWKWMVETTTGPERLKGLLPAGTVVAHKTGTSGIKAGKT
AATNDLGIILLPDGRPLLVAVFVKDSAESSRTNEAIIAQVAQTAYQFELK
KLSALSPN

> P07294|TRAM2_ECOLI Protein traM - Escherichia coli.
MAKVQAYVSDEIVYKINKIVERRRAEGAKSTDVSFSSISTMLLELGLRVY
EAQMERKESAFNQAEFNKVLLECAVKTQSTVAKILGIESLSPHVSGN PKF
EYANMVEDIRDKVSSEMERFFPENDEE
> P24702|SODC_ACTPL Superoxide dismutase [Cu -Zn] - Actinobacillus pleuropneumoniae (Haemophilus pleuropneumoniae).
MKLTNLALAFTLFGASAVAFAHADHDHKKADNSSVEKLVVQVQQLDPVKG
NKDVGTVEITESAYGLVFTPHLHGLAQGLHGFHIHQNPSCEPKEKDGKLV
AGLGAGGHWDPKETKQHGYPWSDNAHLGDLPALFVEHDGSATNPVLAPRL
KKLDEVKGHSLMIHEGGDNHSDHPAPLGGGGPRMACGVIK
> P48982|BGAL_XANMN Beta -galactosidase - Xanthomonas manihotis.
MLRTTLAPLVLALALALPAAAATPESWPTFGTQGTQFVRDGKPYQLLSGA
IHFQRIPRAYWKDRLQKARALGLNTVETYVFWNLVEPQQGQFDFSGNNDV
AAFVKEAAAQGLNVILRPGPYACAEWEAGGYPAWLFGKGNIRVRSRDPRF
LAASQAYLDALAKQVQPLLNHNGGPIIAVQVENEYGSYADDHAYMADNRA
MYVKAGFDKALLFTSDGADMLANGTLPDTLAVVNFAPGEAKSAFDKLIKF
RPDQPRMVGEYWAGWFDHWGKPHAATDARQQAEEFEWILRQGHSANLYMF
IGGTSFGFMNGANFQNNPSDHYAPQTTSYDYDAILDEAGHPTPKFALMRD
AIARVTGVQPPALPAPITTTTLPATPLRESASLWDNLPTPIAIDTPQPME
QFGQDYGYILYRTTITGPRKGPLYLGDVRDVARVYVDQRPVGSVERRLQQ
VSLEVEIPAGQHTLDVLVENSGRINYGTRMADGRAGLVDPVLLDSQQLTG
WQAFPLPMRTPDSIRGWTGKAVQGPAFHRGTLRIGTPTDTYLDMRAFGKG
FAWANGVNLGRHWNIGPQTALYLRPSSARVTTRWWSSTWTMLHPSVRG
> CAIB_ECOLI P31572 L-CARNITINE DEHYDRATASE (EC 4.2.1.89) (L -
MDHLPMPKFGPLAGLRVVFSGIEIAGPFAGQMFAEWGAEVIWIENVAWAD
TIRVQPNYPQLSRRNLHALSLNIFKDEGREAFLKLMETTDIFIEASKGPA
FARRGITDEVLWQHNPKLVIAHLSGFGQYGTEEYTNLPAYNTIAQAFSGY
LIQNGDVDQPMPAFPYTADYFSGLTATTAALAALHKVRETGKGESIDIAM
YEVMLRMGQYFMMDYFNGGEMCPRMSKGKDPYYAGCGLYKCADGYIVMEL
VGITQIEECFKDIGLAHLLGTPEIPEGTQLIHRIECPYGPLVEEKLDAWL
ATHTIAEVKERFAELNIACAKVLTVPELESNPQYVARESITQWQTMDGRT
CKGPNIMPKFKNNPGQIWRGMPSHGMDTAAILKNIGYSENDIQELVSKGL
AKVED
> MURF_ECOLI P11880 UDP -N-ACETYLMURAMOYLALANY L-D-GLUTAMYL-2,6
MISVTLSQLTDILNGELQGADITLDAVTTDTRKLTPGCLFVALKGERFDA
HDFADQAKAGGAGALLVSRPLDIDLPQLIVKDTRLAFGELAAWVRQQVPA
RVVALTGSSGKTSVKEMTAAILSQCGNTLYTAGNLNNDIGVPMTLLRLTP
EYDYAVIELGANHQGEIAWTVSLTRPEAALVNNLAAAHLEGFGSLAGVAK
AKGEIFSGLPENGIAIMNADNNDWLNWQSVIGSRK VWRFSPNAANSDFTA
TNIHVTSHGTEFTLQTPTGSVDVLLPLPGRHNIANALAAAALSMSVGATL
DAIKAGLANLKAVPGRLFPIQLAENQLLLDDSYNANVGSMTAAVQVLAEM
PGYRVLVVGDMAELGAESEACHVQVGEAAKAAGIDRVLSVGKQSHAISTA
SGVGEHFADKTALITRLKLLIAEQQVITILVKGSRSAAMEEVVRALQENG
TC
> P0A8X2|YCEI_ECOLI Protein yceI - Escherichia coli.
MKKSLLGLTFASLMFSAGSAVAADYKIDKEGQHAFVNFRIQHLGYSWLYG
TFKDFDGTFTFDEKNPAADKVNVTINTTSVDTNHAERDKHLRSADFLNTA
KYPQATFTSTSVKKDGDELDITGDLTLNGVTKPVTLEAKLIGQGDDPWGG
KRAGFEAEGKIKLKDFNIKTDLGPASQEVDLIISVEGVQQK
> P36936|PEPE_SALTY Peptidase E - Salmonella typhimurium.
MELLLLSNSTLPGKAWLEHALPLIANQLNGRRSAVFIPFAGVTQTWDEYT
DKTAEVLAPLGVNVTGIHRVADPLAAIEKAEIIIVGGGNTFQLLKESRER
GLLAPMADRVKRGALYIGWSAGANLACPTIRTTNDMPIVDPNGFDALDLF
PLQINPHFTNALPEGHKGETREQRIRELLVVAPELTVIGLPEGNWIQVSN
GQAVLGGPNTTWVFKAGEEAVALEAGHRF
> P45482|FTSZ_ANASP Cell division protein ftsZ - Anabaena sp. (strain PCC 7120).
MTLDNNQELTYRNSQSLGQPGFSLAVNSSNPFNHSGLNFGQNNDSKKISV
ENNRIGEIVPGRVANIKVIGVGGGGGNAVNRMIESDVSGVEFWSINTDAQ
ALTLAGAPSRLQIGQKLTRGLGAGGNPAIGQKAAEESRDEIATALEGADL
VFITAGMGGGTGTGAAPIVAEVAKEMG ALTVGVVTRPFVFEGRRRTSQAE
QGIEGLKSRVDTLIIIPNNKLLEVIPEQTPVQEAFRYADDVLRQGVQGIS
DIITIPGLVNVDFADVRAVMADAGSALMGIGVSSGKSRAREAAIAAISSP

LLECSIEGARGVVFNITGGSDLTLHEVNAAAETIYEVVDPNANIIFGAVI
DDRLQGEVRITVIATGFTGEIQAAPQQNAANARVVSAPPKRTPTQTPLTN
SPAPTPEPKEKSGLDIPDFLQRRRPPKN
> P27830|RFFG_ECOLI dTDP -glucose 4,6-dehydratase - Escherichia coli.
MRKILITGGAGFIGSALVRYIINETSDAVVVVDKLTYAGNLMSLAPVAQS
ERFAFEKVDICDRAELARVFTEHQPDCVMHLAAESHVDRSIDGPAAFIET
NIVGTYTLLEAARAYWNALTEDKKSAFRFHHISTDEVYGDLHSTDDFFTE
TTPYAPSSPYSASKASSDHLVRAWLRTYGLPTL ITNCSNNYGPYHFPEKL
IPLMILNALAGKSLPVYGNGQQIRDWLYVEDHARALYCVATTGKVGETYN
IGGHNERKNLDVVETICELLEELAPNKPHGVAHYRDLITFVADRPGHDLR
YAIDASKIARELGWLPQETFESGMRKTVQWYLANESWWKQVQDGSYQGER
LGLKG
> P23598|PRTF_ERWCH Proteases secretion protein prtF - Erwinia chrysanthemi.
MRRKAVLLTVVLSLSGGSAQAMGLLDAWELALRNDAQLRAAGFERDAGQE
EVAIGRAGLLPSLQYTYGANYSHSKVTQRDRTLNNTTKRDYDNYVSTLTL
RQPLLDYAAWARYQQGVTRKLMADQRFRDRSQDLMVRLYQSWSEALLAQE
KLMLLDAQRRAYQEQLALNRRLLAAGEGTQTDLRETEARYTVTEAQRIEQ
EDTLDAAMTDLENMMGSPLQIQDLSPLALDTLPDNVTENRSLSQWRELTV
RHNAKLAVQRENVDYSRYEIERNRAGHLPTLDLVASTRNSLSESEYNYNQ
KYDTQTVGLQVRVPLYSGGAVSASMRQAAAEYQQSQAELDNQTRQTFAEL
RRQFNLCANGAAKIRAWQMSVAAAEEAIRATRQSVAGGERINLDVLMAEQ
EWYNARRELTEVKYRWLQAWLNLRYTAGTLNEQDMMQLAAWFQSAPVINK
TGINAATGNKTN
> ASRC_SALTY P26476 ANAEROBIC SULFITE REDU CTASE SUBUNIT C (EC
MSIDIDIIKARAKNEYRLSKVRGEAMISVRIPGGILPAHLLTVARDIAET
WGNGQIHLTTRQKLAMPGIRYEDIDNVNAALEPFLREIEIELCDVQVEDT
KAGYLAIGGRNIVACQGNRICQKANTDTTGLSRRLEKLVYPSPYHLKTVI
VGCPNDCAKASMADLGIIGVAKMRFTADRCIGCGACVKACSHHAVGCLAL
KNGKAVKEESACIGCGECVLACPTLAWQRKPD QLWQVRLGGRTSKKSGAL
GKLFLNWVTEDVIKQVIVNLYEFEKEMLGGKPIYLHMGHLIDKGGYLRFK
ERVLRGVQLNPEAMVAERIYWAEDESVARMHLKPAGH
> NHAR_ECOLI P10087 TRANSCRIPTIONAL ACTIVATOR PROTEIN NHAR. -
MSMSHINYNHLYYFWHVYKEGSVVGAAEALYLTPQTITGQIRALEDALQA
KLFKRKGRGLEPSELGELVYRYADKMFTLSQEMLD IVNYRKESNLLFDVG
VADALSKRLVSSVLNAAVVEGEPIHLRCFESTHEMLLEQLSQHKLEMIIS
DCPIDSTQQEGLFSVRIGECGVSFWCTNPPPEKPFPACLEERRLLIPGRR
SMLGRKLLNWFNSQGLNVEILGEFDDAALMKAFGAMHNAIFVAPTLYAYD
FYADKTVVEIGRVENVMEEYHAIFAERMIQHPAVQRICNTDYSALFSPAV
R
> P28904|TREC_ECOLI Trehalose -6-phosphate hydrolase - Escherichia coli.
MTHLPHWWQNGVIYQIYPKSFQDTTGSGTGDLRGVIQHLDYLHKLGVDAI
WLTPFYVSPQVDNGYDVANYTAIDPTYGTLDDFDELVTQAKSRGIRIILD
MVFNHTSTQHAWFREALNKESPYRQFYIWRDGEPETPPNNWRSKFGGSAW
RWHAESEQYYLHLFAPEQADLNWENPAVRAELKKVCEFWADRGVDGLRLD
VVNLISKDPRFPED LDGDGRRFYTDGPRAHEFLHEMNRDVFTPRGLMTVG
EMSSTSLEHCQRYAALTGSELSMTFNFHHLKVDYPGGEKWTLAKPDFVAL
KTLFRHWQQGMHNVAWNALFWCNHDQPRIVSRFGDEGEYRVPAAKMLAMV
LHGMQGTPYIYQGEEIGMTNPHFTRITDYRDVESLNMFAELRNDGRDADE
LLAILASKSRDNSRTPMQWSNGDNAGFTAGEPWIGLGDNYQQINVEAALA
DDSSVFYTYQKLIAL RKQEAILTWGNYQDLLPNSPVLWCYRREWKGQTLL
VIANLSREIQPWQAGQMRGNWQLVMHNYEEASPQPCAMNLRPFEAVWWLQ
K
> P56126|SYK_HELPY Lysyl -tRNA synthetase - Helicobacter pylori
(Campylobacter pylori).
MFSNQYIQQRIHKANSLREEGKNPYQNGLKRSLTNAAFLEKYAYVKGLEE
PKDKEKCESIVGRVKLLRLMGKACFIKVE DESTILQVYVSQNELNDEFKS
LKKHLEVGDIVLVKGFPFATKTGELSIHALEFHILSKTIVPLPEKFHGLS
DIELRYRQRYLDLIVNPSVKDVFKKRSLIVSSVRKFFEMEGFLEVETPMM
HPIPGGANARPFITYHNALEVERYLRIAPELYLKRLIVGGFEAVFEINRN
FRNEGMDHSHNPEFTMIEFYWAYHTYEDLIELSKRLFDYLLKTLNLDSKI
IYNDMEVDFNQTSVISYLDALETIGGISKD ILEKEDRLLAYLLEQGIKVE
PNLTYGKLLAEAFDHFVEHQLINPTFVTQYPIEISPLARRNDSNPNIADR
FELFIAGKEIANGFSELNDPLDQLERFKNQVAEKEKGDEEAQYMDEDYVW

ALAHGMPPTAGQGIGIDRLVMLLTGAKSIKDVILFPAMRPVKNDFNVESE
E
> P15412|RUBR_DESVM Rubredoxin - Desulfovibrio vulgaris (strain Miyazaki).
MKKYVCTVCGYEYDPAEGDPDNGVKPGTAFEDVPADWVCPICGAPKSEFE
PA
> P0ADA1|TESA_ECOLI Acyl -CoA thioesterase I - Escherichia coli.
MMNFNNVFRWHLPFLFLVLLTFRAAAADTLLILGDSLSAGYRMSASAAWP
ALLNDKWQSKTSVVNASISGDTSQQGLARLPALLKQHQPRWVLVELGGND
GLRGFQPQQTEQTLRQILQDVKAANAEPLLMQIRLPANYGR RYNEAFSAI
YPKLAKEFDVPLLPFFMEEVYLKPQWMQDDGIHPNRDAQPFIADWMAKQL
QPLVNHDS
> SYN_HAEIN P43829 ASPARAGINYL -TRNA SYNTHETASE (EC 6.1.1.22)
MNYIQLQRFNMSKVASIVDVLQGKVAIGETVTVRGWVRTRRDSKAGLSFL
AVYDGSCFDPIQAIINNDIENYESEILRLTTGCSVIVTGKVVESPAEGQA
VELQAEKVEVTGFVEDPDTYPM AAKRHSIEYLREVAHLRPRTNIIGAVAR
VRHCLSQAIHRFFHEQGFYWVATPLITASDTEGAGEMFRVSTLDLENLPR
SENGKVDFSQDFFGKESFLTVSGQLNGETYACALSKIYTFGPTFRAENSN
TTRHLAEFWMVEPEVAFATLADNAKLAEDMLKYVFRAVLAERKDDLQFFE
KHVDKDVITRLENFVNSDFAQIDYTDAIDVLLKSGKKFEFPVSWGIDLSS
EHERFLAEEYFKSPVVVKNYPKD IKAFYMRLNDDEKTVAAMDVLAPGIGE
IIGGSQREERLEVLDKRMEEMGLNPDDYWWYRDLRKYGSVPHSGFGLGFE
RLIVYVTGVQNIRDVIPFPRAPRNANF
> P09790|IGA_NEIGO IgA -specific serine endopeptidase - Neisseria
gonorrhoeae.
MKAKRFKINAISLSIFLAYALTPYSEAALVRDDVDYQIFRDFAENKGKFF
VGATDLSVKNKRGQNIGNAL SNVPMIDFSVADVNKRIATVVDPQYAVSVK
HAKAEVHTFYYGQYNGHNDVADKENEYRVVEQNNYEPHKAWGASNLGRLE
DYNMARFNKFVTEVAPIAPTDAGGGLDTYKDKNRFSSFVRIGAGRQLVYE
KGVYHQEGNEKGYDLRDLSQAYRYAIAGTPYKDINIDQTMNTEGLIGFGN
HNKQYSAEELKQALSQDALTNYGVLGDSGSPLFAFDKQKNQWVFLGTYDY
WAGYGKKSWQEWNIYKKEFAD KIKQHDNAGTVKGNGEHHWKTTGTNSHIG
STAVRLANNEGDANNGQNVTFEDNGTLVLNQNINQGAGGLFFKGDYTVKG
ANNDITWLGAGIDVADGKKVVWQVKNPNGDRLAKIGKGTLEINGTGVNQG
QLKVGDGTVILNQKADADKKVQAFSQVGIVSGRGTLVLNSSNQINPDNLY
FGFRGGRLDANGNDLTFEHIRNVDEGARIVNHNTDHASTITLTGKSLITN
PNSLSVHSIQNDYDEDDYSYYY RPRRPIPQGKDLYYKNYRYYALKSGGRL
NAPMPENGVAENNDWIFMGYTQEEARKNAMNHKNNRRIGDFGGFFDEENG
KGHNGALNLNFNGKSAQKRFLLTGGANLNGKISVTQGNVLLSGRPTPHAR
DFVNKSSARKDAHFSKNNEVVFEDDWINRTFKAAEIAVNQSASFSSGRNV
SDITANITATDNAKVNLGYKNGDEVCVRSDYTGYVTCNTGNLSDKALNSF
DATRINGNVNLNQNAALVLGKAA LWGKIQGQGNSRVSLNQHSKWHLTGDS
QVHNLSLADSHIHLNNASDAQSANKYHTIKINHLSGNGHFHYLTDLAKNL
GDKVLVKESASGHYQLHVQNKTGEPNQEGLDLFDASSVQDRSRLFVSLAN
HYVDLGALRYTIKTENGITRLYNPYAGNGRPVKPAPSPAANTASQAQKAT
QTDGAQIAKPQNIVVAPPSPQANQAEEALRQQAKAEQVKRQQAAEAEKVA
RQKDEEAKRKAAEIARQQEEARKA AELAAKQKAEAERKARELARQKAEEA
SHQANAKPKRRRRRAILPRPPAPVFSLDDYDAKDNSESSIGNLARVIPRM
GRELINDYEEIPLEELEDEAEEERRQATQFHSKSRNRRAISSEPSSDEDA
SESVSTSDKHPQDNTELHEKVETAGLQPRAAQPRTQAAAQADAVSTNTNS
ALSDAMASTQSILLDTGAYLTRHIAQKSRADAEKNSVWMSNTGYGRDYAS
AQYRRFSSKRTQTQIGIDRSLSENM QIGGVLTYSDSQHTFDQAGGKNTFV
QANLYGKYYLNDAWYVAGDIGAGSLRSRLQTQQKANFNRTSIQTGLTLGN
TLKINQFEIVPSAGIRYSRLSSADYKLGDDSVKVSSMAVKTLTAGLDFAY
RFKVGNLTVKPLLSAAYFANYGKGGVNVGGKSFAYKADNQQQYSAGVALL
YRNVTLNVNGSITKGKQLEKQKSGQIKIQIRF
> P21413|FM98_ECOLI Fimbrial protein 987P - Escherichia coli.
MRMKKSALTLAVLSSLFSGYSLAAPAENNTSQANLDFTGKVTASLCQVDT
SNLSQTIDLGELSTSALKATGKGPAKSFAVNLINCDTTLNSIKYTIAGNN
NTGSDTKYLVPASNDTSASGVGVYIQDNNAQAVEIGTEKTVPVVSNGGLA
LSDQSIPLQAYIGTTTGNPDTNGGVTAGTVTASAVMTIRSAGTP
> P04127|PAPA_ECOLI Pap fimbrial major p ilin protein - Escherichia coli.
MIKSVIAGAVAMAVVSFGVNNAAPTIPQGQGKVTFNGTVVDAPCSISQKS
ADQSIDFGQLSKSFLEAGGVSKPMDLDIELVNCDITAFKGGNGAKKGTVK

LAFTGPIVNGHSDELDTNGGTGTAIVVQGAGKNVVFDGSEGDANTLKDGE
NVLHYTAVVKKSSAVGAAVTEGAFSAVANFNLTYQ
> METC_HAEIN P44527 CYSTATHIONINE BETA-LYASE (EC 4.4.1.8) (CB
MQTKYDLSTMFIHSGRQKRFSQGSVNPVLQRASSLLFDSIEDKKHATQRR
AKGELFYGRRGTLTHFALQDLMCEMEGGAGCYLYPCGTAAVTNSILSFVK
TGDHVLMSGAAYEPTQYFCNIVLKKMQIDITYYDPLIGEDIATLIQPNTK
VLFLEAPSSITMEIPDIPTIVKAARKVNPNIVIMIDNTWSAGVLFKALEH
DIDISIQAGTKYLVGHSDIMIGTA VANARTWDQLREHSYLMGQMVDADSA
YTTARGIRTLGVRLKQHQESSIKVAKWLSEQPEVKTVYHPALPSCPGHEF
FLRDFSGSSGLFSFELTQRLTSEQVSKFMDHFQLFAMAYSWGGFESLILC
NQPEEIAHIRPNIKRNLTGSLIRVHIGFENVDELIADLKAGFERIA
> AMPD_ECOLI P13016 AMPD PROTEIN. - ESCHERICHIA COLI.
MLLEQGWLVGARRVPSPHYDCRPDDE TPTLLVVHNISLPPGEFGGPWIDA
LFTGTIDPQAHPFFAEIAHLRVSAHCLIRRDGEIVQYVPFDKRAWHAGVS
QYQGRERCNDFSIGIELEGTDTLAYTDAQYQQLAAVTRALIDCYPDIAKN
MTGHCDIAPDRKTDPGPAFDWARFRVLVSKETT
> P42673|PCP_PSEFL Pyrrolidone -carboxylate peptidase - Pseudomonas fluorescens.
MRIVLLTGFEPFDQD PVNPSWEAVRQLDGVQLGSDVKIVARRLPCAFATA
GECLTRLIDELHPAMVIATGLGPGRSDISVERVAININDARIPDNLGEQP
IDTAVVADGPAAFFTTLPIKAMVKAVREAGIAASVSQTAGTFVCNQVFYL
LQHALAGSGVRSGFIHVPFLPEQVAGSQRPSMALDAMVAGLQAAVLTAWH
TPVDVKEAGGQVS
> P39187|YTFJ_ECOLI Protein ytfJ - Escherichia coli.
MTLRKILALTCLLLPMMASAHQFETGQRVPPIGITDRGELVLDKDQFSYK
TWNSAQLVGKVRVLQHIAGRTSAKEKNATLIEAIKSAKLPHDRYQTTTIV
NTDDAIPGSGMFVRSSLESNKKLYPWSQFIVDSNGVALGAWQLDEESSAV
VVLDKDGRVQWAKDGALTPEEVQQVMDLLQKLLK
> P0A940|YAET_ECOLI Outer membrane protein assembly factor yaeT - Escherichia coli.
MAMKKLLIASLLFSSATVYGAEGFVVKDIHFEGLQRVAVGAALLSMPVRT
GDTVNDEDISNTIRALFATGNFEDVRVLRDGDTLLVQVKERPTIASITFS
GNKSVKDDMLKQNLEASGVRVGESLDRTTIADIEKGLEDFYYSVGKYSAS
VKAVVTPLPRNRVDLKLVFQEGVSAEIQQINIVGNHAFTTDELISHFQLR
DEVPWWNVVGDRKYQKQKLAGDLETLRSYYLDRGYA RFNIDSTQVSLTPD
KKGIYVTVNITEGDQYKLSGVEVSGNLAGHSAEIEQLTKIEPGELYNGTK
VTKMEDDIKKLLGRYGYAYPRVQSMPEINDADKTVKLRVNVDAGNRFYVR
KIRFEGNDTSKDAVLRREMRQMEGAWLGSDLVDQGKERLNRLGFFETVDT
DTQRVPGSPDQVDVVYKVKERNTGSFNFGIGYGTESGVSFQAGVQQDNWL
GTGYAVGINGTKNDYQTYAELSVTNPYFTVDGVSLGG RLFYNDFQADDAD
LSDYTNKSYGTDVTLGFPINEYNSLRAGLGYVHNSLSNMQPQVAMWRYLY
SMGEHPSTSDQDNSFKTDDFTFNYGWTYNKLDRGYFPTDGSRVNLTGKVT
IPGSDNEYYKVTLDTATYVPIDDDHKWVVLGRTRWGYGDGLGGKEMPFYE
NFYAGGSSTVRGFQSNTIGPKAVYFPHQASNYDPDYDYECATQDGAKDLC
KSDDAVGGNAMAVASLEFITPTPFISDKYANSVRTSFF WDMGTVWDTNWD
SSQYSGYPDYSDPSNIRMSAGIALQWMSPLGPLVFSYAQPFKKYDGDKAE
QFQFNIGKTW
> P01559|HST1_ECOLI Heat -stable enterotoxin ST -IA/ST-P - Escherichia coli.
MKKLMLAIFISVLSFPSFSQSTESLDSSKEKITLETKKCDVVKNNSEKKS
ENMNNTFYCCELCCNPACAGCY
> P31884|MBHS_WOLSU Quinone -reactive Ni/Fe-hydrogenase small chain - Wolinella succinogenes.
MLEEKGIERRDFMKWAGAMTAMLSLPATFTPLTAKAAELADRLPVIWLHM
AECTGCSESLLRTDGPGIDSLIFDYISLEYHETVMAAAGWQAEHNLEHAI
EKYKGRYVLMVEGGIPAGSSEFYLTVGPHGTTGAEHARHASANAAAIFAI
GSCSSFGGVQAARPNPTNAQPLSKVTNKPVINVPGCPPSEKN IVGNVLHF
ILFGTLPSVDAFNRPMWAYGLRIHDLCERRGRFDAGEFVQEFGDEGAKKG
YCLYKVGCKGPYTFNNCSKLRFNQHTSWPVQAGHGCIGCSEPDFWDTMGP
FEEPVANRLYATAFDGLGADKTADKIGITLLAATAVGVAAHAVLSMMVKD
KENN
> P43825|SYK_HAEIN Lysyl -tRNA synthetase - Haemophilus influenzae.
MSEQEVKELDLNGEMLVRREKL AALRAKGNAFPNKFRRDALAQDLHNQYD
AEDGEILKEKGIEVQVAGRIMTRRAMGKATFITIQDMSGKIQLYVARDNL

PEGVYKDDVGTWDLGDIVGIKGTLFKTKTDELTVKTTEVQLLTKALRPLP
DKFHGLTDQEVRYRQRYLDLISNEESRRTFIIRSKVVAGIREYFISKGFM
EVETPMLQVIPGGASARPFVTHHNALDVDMYLRIAPELYLKRLVVGGFER
VFELNRNFRNEGVSVRHNPEFTM LEYYQAYADYHDLMDNTEELLRKLAID
ILGTTIVKYGDLEFDFGKPFERITLHDATIKYGADKGIVKEDLYDFDRAK
ATAERLGIEVQKSWGLGSIVNAIFEEVAEHHLIQPTFLNGSPAEISPLAR
RNDENPEVTDRFELFIGGREIGNGFSELNDAEDQNERFDAQVAAKEAGDD
EAMFKDEDFVVALEHGLPPTAGEGLGIDRLAMLYANAPSIRDVILFPAMR
QK
> P05827|ILVY_ECOLI H TH-type transcriptional regulator ilvY - Escherichia coli.
MDLRDLKTFLHLAESRHFGRSARAMHVSPSTLSRQIQRLEEDLGQPLFVR
DNRTVTLTEAGEELRVFAQQTLLQYQQLRHTIDQQGPSLSGELHIFCSVT
AAYSHLPPILDRFRAEHPSVEIKLTTGDAADAMEKVVTGEADLAIAGKPE
TLPGAVAFSMLENLAVVLIAPALPCPVRNQVSVEKPDWSTVPFI MADQGP
VRRRIELWFRRNKISNPMIYATVGGHEAMVSMVALGCGVALLPEVVLENS
PEPVRNRVMILERSDEKTPFELGVCAQKKRLHEPLIEAFWKILPNHK
> P37648|YHJJ_ECOLI Protein yhjJ - Escherichia coli.
MQGTKIRLLAGGLLMMATAGYVQADALQPDPAWQQGTLSNGLQWQVLTTP
QRPSDRVEIRLLVNTGSLAESTQQSGYSHAIPRIALTQSGGLDAAQ ARSL
WQQGIDPKRPMPPVIVSYDTTLFNLSLPNNRNDLLKEALSYLANATGKLT
ITPETINHALQSQDMVATWPADTKEGWWRYRLKGSTLLGHDPADPLKQPV
EAEKIKDFYQKWYTPDAMTLLVVGNVDARSVVDQINKTFGELKGKRETPA
PVPTLSPLRAEAVSIMTDAVRQDRLSIMWDTPWQPIRESAALLRYWRADL
AREALFWHVQQALSASNSKDIGLGFDCRVLYLRAQCAINIESPNDKL NSN
LNLVARELAKVRDKGLPEEEFNALVAQKKLELQKLFAAYARADTDILMGQ
RMRSLQNQVVDIAPEQYQKLRQDFLNSLTVEMLNQDLRQQLSNDMALILL
QPKGEPEFNMKALQAVWDQIMAPSTAATTSVATDDVHPEVTDIPPAQ
> P0A283|PTGA_SALTY Glucose -specific phosphotransferase enzyme IIA component - Salmonella typhimurium .
MGLFDKLKSLVSDDKKDTGTIEIVAPLSGEIVNIEDVPDVVFAEKIVGDG
IAIKPTGNKMVAPVDGTIGKIFETNHAFSIESDSGIELFVHFGIDTVELK
GEGFKRIAEEGQRVKVGDPVIEFDLPLLEEKAKSTLTPVVISNMDEIKEL
IKLSGSVTVGETPVIRIKK
> P11312|FMF3_ECOLI F17 fimbrial protein - Escherichia coli.
MQKIQFILGILAAASSSATL AYDGKITFNGKVVDQTCSVTTESKNLTVKL
PTVSANSLASSGKVVGLTPFTILLEGCNTPAVTGAQNVNAYFEPNANTDY
TTGNLTNTASSGASNVQIQLLNADGVKAIKLGQAAAAQSVDTVAINDANV
TLRYNAQYYATGVATAGDVTSTVNYTIAYQ
> P44443|RNE_HAEIN Ribonuclease E - Haemophilus influenzae.
MKRMLINATQKEELRVALVDGQRLFDLDIESP GHEQKKANIYKGKITRVE
PSLEAAFVDYGAERHGFLPLKEIAREYFPDDYVFQGRPNIRDILVEGQEV
IVQVNKEERGNKGAALTTFVSLAGSYLVLMPNNPRAGGISRRIEGDERTE
LKEALSSLDVPDGVGLIVRTAGVGKSPEELQWDLKVLLHHWEAIKQASQS
RPAPFLIHQESDVIVRAIRDYLRRDIGEILIDSPKIFEKAKEHIKLVRPD
FINRVKLYQGEVPLFSHYQIESQIESAFQREVR LPSGGSIVIDVTEALTA
IDINSARSTRGGDIEETALNTNLEAADEIARQLRLRDLGGLVVIDFIDMT
PIRHQREVENRIRDAVRPDRARIQISRISRFGLLEMSRQRLSPSLGESSH
HICPRCQGTGKVRDNESLSLSILRLLEEEALKENTKQVHTIVPVQIASYL
LNEKRKAISNIEKRHNVDIIVAPNEAMETPHFSVFRLRDGEEVNELSYNL
AKIHCAQDENTEESLLSRNVETTAVIEQPAVESA VVALSISEAAPTPVER
KSNEPSLLAKIIAKIKGLFATKSEENKPKNNRTSRNPNRNQRRSQDRRSS
RRPRSENNETERTEEQVRNVRERNQRRPRRNLVEESIAESAVNSTPVFEA
KEERTEPVTQRRQRRDLRKRVRVEDNETVVENNFSTTEKMPEVDVITVQN
NDEKPVHQNQRSERQERQRRTPRHLRAANNQRRRRDQEPKSPMPLFAAVV
SPELASGKAWIDYSTVNLPKENHFLSVDELLEQEK TKKGFITPAMGIVVE
EKSPDVKPALDFITQPANESVQKKVQESLDRLSSYKPQEVVESIDPAINV
DEPETLEKVSKFVRTYEFNGRLGTISSVPHTKAEMTLAKANDEMPEDFPI
RAWQDSRYYFYGKGAAGHHCAISHVYSEPTRTKSE
> P35636|SCR_ZYMMO Sucrose -6-phosphate hydrolase - Zymomonas mobilis.
MESPSYKNLIKAEDAQKKAGKRLLSSEWYPGF HVTPLTGWMNDPNGLIFF
KGEYHLFYQYYPFAPVWGPMHWGHAKSRDLVHWETLPVALAPGDSFDRDG
CFSGCAVDNNGVLTLIYTGHIVLSNDSLDAIREVQCMATSIDGIHFQKEG

IVLEKAPMPQVAHFRDPRVWKENNHWFMVVGYRTDDEKHQGIGHVALYRS
ENLKDWIFVKTLLGDNSQLPLGKRAFMWECPDFFSLGNRSVLMFSPQGLK
ASGYKNRNLFQNGYILGKWQAPQFTPETSFQEL DYGHDFYAAQRFEAKDG
RQILIAWFDMWENQKPSQRDGWAGCMTLPRKLDLIDNKIVMTPVREMEIL
RQSEKIESVVTLSDAEHPFTMDSPLQEIELIFDLEKSSAYQAGLALRCNG
KGQETLLYIDRSQNRIILDRNRSGQNVKGIRSCPLPNTSKVRLHIFLDRS
SIEIFVGDDQTQGLYSISSRIFPDKDSLKGRLFAIEGYAVFDSFKRWTLQ
DANLAAFSSDAC
> P43928|RF3_HAEIN Pe ptide chain release factor 3 - Haemophilus influenzae.
MSYPLEEVNKRRTFAIISHPDAGKTTITEKVLLYGNAIQTAGSVKGKGSA
AHAKSDWMEMEKQRGISITTSVMQFPYNDCLVNLLDTPGHEDFSEDTYRT
LTAVDSCLMVIDSAKGVEERTIKLMEVTRLRDTPIITFMNKLDRDIRDPI
ELLDEVENVLKIRCAPITWPIGCGKLFKGVYHLAKDETYLYQSGQGST IQ
AVRVVKGLNNPELDVAVGDDLAQQLREELELVQGASNEFEQDAFIKGELT
PVFFGTALGNFGVDHFLDGLTQWAPKPQSRQADTRTVESAEEKFSGFVFK
IQANMDPKHRDRVAFMRVVSGKYEKGMKLKHVRIGKDVVISDALTFMAGD
RAHAEEAYAGDIIGLHNHGTIQIGDTFTQGETLKFTGIPNFAPELFRRIR
LKDPLKQKQLLKGLVQLSEEGAVQVFRPLLNNDLIVGAVGVLQFDVVVS R
LKTEYNVEAIYENVNVATARWVECADEKKFEEFKRKNEQNLALDGGDNLT
YIAPTMVNLNLAQERYPDVVFYKTREH
> P13720|PAPG_ECOLI Fimbrial adhesin papG - Escherichia coli.
MKKWFPAFLFLSLSGGNDALAGWHNVMFYAFNDYLTTNAGNVKVIDQPQL
YIPWNTGSATATYYSCSGPEFASGVYFQEYLAWMVVPKHVYTNEGFNIFL
DVQSKYGWSME NENDKDFYFFVNGYEWDTWTNNGARICFYPGNMKQLNNK
FNDLVFRVLLPVDLPKGHYNFPVRYIRGIQHHYYDLWQDHYKMPYDQIKQ
LPATNTLMLSFDNVGGCQPSTQVLNIDHGSIVIDRANGNIASQTLSIYCD
VPVSVKISLLRNTPPIYNNNKFSVGLGNGWDSIISLDGVEQSEEILRWYT
AGSKTVKIESRLYGEEGKRKPGELSGSMTMVLSFP
> P13734|AGAR_ALTAT Beta -agarase - Alteromonas atlantica (Pseudomonas atlantica).
MLKVIPWLLVTSSLVAIPTYIHATTEVVVNLNVKHSVEGKSEFERKNHIK
LHSTLNDNDWQGEEDKLKYMMEELDVYFGRDNGGTVWNFNQAIEDPANIG
YADPQNIIARGQAQRETNWGQNKSALHQYDGRGDLMIGGQPRAHYLGNTS
PCCGGSAWQAKGGDAVGDFLGQYVNEFFRSAGDPVTKGHLAPVYFEVL NE
PLYQVTDAPHELGLEQPIPPIDIFTFHNDVADAFRQHNTHIKIGGFTVAF
PIFEQREFARWEERMKLFIDTSGSHMDVYSTHFYDLEDDNRFKGSRLEAT
LDMIDQYSLLALGETKPHVISEYGGRNRPMENAPWSALRDWWFLKTASPM
LMQFLSRPDSVLTSIPFVPIKALWGTAADGTPYNWRLLRQQKEAPNETGE
NWVFTEMVKFYQLWSDVKGTRVDTFSTNSDFLIDSYVQNDKAYVLISNL T
EQAEKIVVHKYGAPASSQPTTRIKHLYLKGAAPRLMKQVMRQISKKSRLL
LKRLW
> P46025|OPP23_HAEIN Outer membrane protein P2 - Haemophilus influenzae.
MKKTLAALIVGAFAASAANAAVVYNNEGTNVELGGRLSIITEQSNSTVDD
QEQQHGALRNAGSRFHIKATHNFGDGFYAQGYLETRLVSDYPESSSDHFG
GITTKYAYVTLGNKAFGEVKLG RAKTIADGITSAEDKEYGVLNNKKYIPT
NGNTVGYTYKGIDGLDGLVLGANYLLAQSRVPGGPSPFPRKQGEVYPQQI
SNGVQVGAKYDANNIIAGIAFGRTNYKTAGADFDPYGDFGLGRKEQVEGV
LSTLGYRFSDLGLLVSLDSGYAKTKYYTTTDSSSGSQTITNPAYDEKRSF
VSPGFQYELMEDTNVYGNFKYERTSVNQGKNTREQAVLFGVDHKLHKQVL
TYIEGAYARTKTNDKGKTEKTGK EKSVGVGLRVYF
> P24930|RUS2_THIFE Rusticyanin - Thiobacillus ferrooxidans (Acidithiobacillus ferrooxidans).
MYTQNTMKKNWYVTVGAAAALAATVGMGTAMAGTLDTTWKEATLPQVKAM
LEKDTGKVSGDTVTYSGKTVHVVAAAVLPGFPFPSFEVHDKKNPTLEIPA
GATVDVTFINTNKGFGHSFDITKKGPPYAVMPVIDPIVAGTGFSPVP KDG
KFGYTDFTWHPTAGTYYYVCQIPGHAATGMFGKIVVK
> P24305|OMP32_COMAC Outer membrane porin protein 32 - Comamonas acidovorans (Pseudomonas acidovorans) (Delftia acidovorans).
MKKSLIALAVLAASGAAMAQSSVTLFGIVDTNVAYVNKDAAGDSRYGLGT
SGASTSRLGLRGTEDLGGGLKAGFWLEGEIFGDDGNA SGFNFKRRSTVSL
SGNFGEVRLGRDLVPTSQKLTSYDLFSATGIGPFMGFRNWAAGQGADDNG
IRANNLISYYTPNFGGFNAGFGYAFDEKQTIGTADSVGRYIGGYVAYDNG
PLSASLGLAQQKTAVGGLATDRDEITLGASYNFGVAKLSGLLQQTKFKRD

IGGDIKTNSYMLGASAPVGGVGEVKLQYALYDQKAIDSKAHQITLGYVHN
LSKRTALYGNLAFLKNKDASTLGLQAKGVYAGGVQAGE SQTGVQVGIRHA
F
> P02930|TOLC_ECOLI Outer membrane protein tolC - Escherichia coli.
MKKLLPILIGLSLSGFSSLSQAENLMQVYQQARLSNPELRKSAADRDAAF
EKINEARSPLLPQLGLGADYTYSNGYRDANGINSNATSASLQLTQSIFDM
SKWRALTLQEKAAGIQDVTYQTDQQTLILNTATAYFNVLNAIDVLSYTQA
QKEAIYRQLDQTTQRFNVGL VAITDVQNARAQYDTVLANEVTARNNLDNA
VEQLRQITGNYYPELAALNVENFKTDKPQPVNALLKEAEKRNLSLLQARL
SQDLAREQIRQAQDGHLPTLDLTASTGISDTSYSGSKTRGAAGTQYDDSN
MGQNKVGLSFSLPIYQGGMVNSQVKQAQYNFVGASEQLESAHRSVVQTVR
SSFNNINASISSINAYKQAVVSAQSSLDAMEAGYSVGTRTIVDVLDATTT
LYNAKQELANARYNYLINQLN IKSALGTLNEQDLLALNNALSKPVSTNPE
NVAPQTPEQNAIADGYAPDSPAPVVQQTSARTTTSNGHNPFRN
> P73456|FTSZ_SYNY3 Cell division protein ftsZ - Synechocystis sp. (strain PCC 6803).
MTLNNDLPLNNIGFTGSGLNDGTEGLDDLFSSSIVDNEPLEALVETPTFA
SPSPNLKRDQIVPSNIAKIKVIGVGGGGCNAVNRMIASGVTGIDF WAINT
DSQALTNTNAPDCIQIGQKLTRGLGAGGNPAIGQKAAEESRDEIARSLEG
TDLVFITAGMGGGTGTGAAPIVAEVAKEMGCLTVGIVTRPFTFEGRRRAK
QAEEGINALQSRVDTLIVIPNNQLLSVIPAETPLQEAFRVADDILRQGVQ
GISDIIIIPGLVNVDFADVRAVMADAGSALMGIGVGSGKSRAKEAATAAI
SSPLLESSIQGAKGVVFNVTGGTDLTLHEVNVAAEIIYEVVDADAN IIFG
AVIDDRLQGEMRITVIATGFNGEKEKPQAKTSSKPVLSGPPAGVETVPST
TTPEDPLGEIPMAPELDIPDFLQKRRFPRR
> P52682|BLAN_SERMA Carbapenem -hydrolyzing beta -lactamase Sme -1 - Serratia marcescens.
MSNKVNFKTASFLFSVCLALSAFNAHANKSDAAAKQIKKLEEDFDGRIGV
FAIDTGSGNTFGYRSDERFPLCSSFKGFLAA AVLERVQQKKLDINQKVKY
ESRDLEYHSPITTKYKGSGMTLGDMASAALQYSDNGATNIIMERFLGGPE
GMTKFMRSIGDNEFRLDRWELELNTAIPGDKRDTSTPKAVANSLNKLALG
NVLNAKVKAIYQNWLKGNTTGDARIRASVPADWVVGDKTGSCGAIGTAND
YAVIWPKNRAPLIVSIYTTRKSKDDKHSDKTIAEASRIAIQAID
> P39186|NAPB_RALEH Diheme cytochrome c napB - Ralstonia eutropha (strain ATCC 17699 / H16 / DSM 428 / Stanier 337) (Cupriavidus necator (strain ATCC 17699 / H16 / DSM 428 / Stanier 337)).
MKPSRSWASLLAVCAVLLAALAMQAIFFPAPARAQGLVDAMRGPTAIANE
PRAPLLYPTENKDIRRTRNYTMQPPTIPHKIDGYQLDKDFNRCMFCHART
RTEETQAIPVSITHYMDRDNNVLADVSPRRYFCTQCHVPQADTKPLIGNN
FVDVDTILKRRPGAKGAAK
> P45600|CYSB_KLEPN HTH -type transcriptional regulator cysB - Klebsiella pneumoniae.
MKLQQLRYIVEVVNHNLNVSSTAEGLYTSQPGISKQVRMLEDELGIQIFA
RSGKHLTQVTPAGQEIIRIAREVLSKVDAIKSVAGEHTWPDKGSLYVATT
HTQARYALPGVIKGFIERYPRVSLHMHQGSPTQIAEAVSKGNADFAIATE
ALHLYDDLVMLPCYHWNRSIVVTPEHPLATKGSVSIEELAQYPLVTYTFG
FTGRSELDTAFNRAGLTPRIVFTATDADVIKTYVRLGLGVGVIASMAVDP
VSDPDLVKLDANGIFSHSTTKIGFRRSTFLRSYMYDFIQRFAPHLTRDVV
DTAVALRSNEDIEAMFKDIKLPEK
> P56000|SYV_HELPY Valyl -tRNA synthetase - Helicobacter pylori (Campylobacter pylori).
MIMKQEPTTYQPEEIEKKIYEICSHRGYFEIDGNEAIQEKNKRFCLMMPP
PNVTGVLHIGHALTLSLQDILARYKRMDGYKTLYQPGLDHAGIATQNVVE
KQLLSQGIKKEDLGREEFIKKVWEWKEKSGGAILEQMKRLGVSAAFSRTR
FTMDKGLQRAVKLAFLKWYEKGLIIQDNYMVNWCTKDGALSDIE VEYEER
KGALYYIRYYLENQKDYLVVATTRPETLFGDSALMVNPNDERYKHLVGQK
AILPLIHRTIPIIADEHVEMEFGTGCVKVTPGHDFNDYEVGKRHHLETIK
IFDEKGILNAHCGEFENLERLEARDKVVERLKENALLEKIEEHTHQVGHC
YRCHNVVEPYVSKQWFVKPEIAQSSIEKIQQGLARFYPSNWINNYNAWMR
ELRPWCISRQLFWGHQIPVFTCENNHQFVSLDTPLSCPTCKSETL EQDKD
VLDTWFSSGLWAFSTLGWGQEKSGLFNESDLKDFYPNTTLITGFDILFFW
VARMLFCSESLLGELPFKDIYLHALVRDEKGEKMSKSKGNVIDPLEMIEK
YGADSLRFTLANLCATGRDIKLSTTHLENNKNFANKLFNAASYLKLKQES

FKDKERLNEYQTPLGRYAKSRLNSATKEARNALDNYRFNDATTLLYRFLW
GEFCDWFIEFSKVENEAIDELGSVLKEALKLLHPFMPFISESLYHK LSNT
ELENTESIMVMPYPKDLAQDEKLEHEFEVIKDCIVSLRRLKIMLETPPIV
LKEASVGLREAIENTERLQTYAQKLARLEKVSVISSKPLKSVSDVGEFCQ
TYANLENLDLSPLVARLKKQLEKLEKEKLKLNLHNENFVKNAPKSVLEKA
KESLKTLLEKESKIKQELDLLEQP
> P15488|FMS3_ECOLI CS3 fimbrial subunit A - Escherichia coli.
MLKIKYLLIGLSLSAMSSYSLAAAGPTLTKELALNVLSPAALDATWAPQD
NLTLSNTGVSNTLVGVLTLSNTSIDTVSIASTNVSDTSKNGTVTFAHETN
NSASFATTISTDNANITLDKNAGNTIVKTTNGSQLPTNLPLKFITTEGNE
HLVSGNYRANITITSTIK
> Q06530|DHSU_CHRVI Sulfide dehydrogenase [flavocytochrome c] flavoprotein chain - Chromatium vinosum (Allochromatium vinosum).
MTLNRRDFIKTSGAAVAAVGILGFPHLAFGAGRKVVVVGGGTGGATAAKY
IKLADPSIEVTLIEPNTDYYTCYLSNEVIGGDRKLESIKHGYDGLRAHGI
QVVHDSATGIDPDKKLVKTAGGAEFGYDRCVVAPGIELIYDKIEGYSEEA
AAKLPHAWKAGEQTAILRKQLEDMADGGTVVIAPPAAPFRCPPGPYERAS
QVAYYLKAHKPKSKVIIL DSSQTFSKQSQFSKGWERLYGFGTENAMIEWH
PGPDSAVVKVDGGEMMVETAFGDEFKADVINLIPPQRAGKIAQIAGLTND
AGWCPVDIKTFESSIHKGIHVIGDACIANPMPKSGYSANSQGKVAAAAVV
ALLKGEEPGTPSYLNTCYSILAPAYGISVAAIYRPNADGSAIESVPDSGG
VTPVDAPDWVLEREVQYAYSWYNNIVHDTFG
> P26509|PGLR2_PECCC Endo -polygalacturonase - Pectobacterium carotovorum subsp. carotovorum (Erwinia carotovora subsp. carotovora).
MEYQSGKRVLSLSLGLIGLFSASAWASDSRTVSEPKTPSSCTTLKADSST
ATSTIQKALNNCDQGKAVRLSAGSTSVFLSGPLSLPSGVSLLIDKGVTLR
AVNNAKSFENAPSSCGVVDKNGKGCDAFITAVSTTNSGIYGPGTIDGQGG
VKLQDKKVSWWELAADAKVKKLKQNTPRLIQINKSKNFTLYNVSLINSPN
FHVVFSDGDGFTAWKTTIKTPSTARNTDGIDPMSSKNITIAYSNIATGDD
NVAIKAYKGRAETRNISILHNDFGTGHGMSIGSETMGVYNVTVDDLKMNG
TTNGLRIKSDKSAAGVVNGVRYSNVVMKNVAKPIVIDTVYEKKEGSNVPD
WSDITFKDVTSETKGVVVLNGENAKKPIEVTMKNVKLTSDSTWQIKNVNV
KK
> P12608|IRPA_SYNP7 Iron-regulated protein A - Synechococcus sp. (strain PCC 7942) (Anacystis nidulans R2).
MIVTGSQVRQGLNTWFVLPLRRTAIGLGCAGVATLFSACGQTQALITNQT
IQGFVDQVVVPSYVSVAAGATQLEQALQTYQQAPTAANLEAARQAWRVAR
DRWEQTECFAFGPADSEGFDGAMDTWPIDRQGLKTAAAQPVEQREDSRKG
FHAIEELLFAATEPTLSDRQHLVILATDLTKQAQGLVTRWQQASDQPAYR
SVLLSAGSTDSAYPTLNAAGTEIVQGLVDSLSEVASEKIGGPLETQEPDR
FESFVSRNTLSDLRNNWTGAWNVYRGQRSDGVAAGSLQQRLQQQHPVIAQ
QLDQQFATARQALWAIPEPIETNLASPRGKVAVLTAQTAIAAVSDTLERQ
VLPLVQ
> P29492|TCPN_VIBCH TCP pilus virulence regulat ory protein - Vibrio cholerae.
MIGKKSFQTNVYRMSKFDTYIFNNLYINDYKMFWIDSGIAKLIDKNCLVS
YEINSSSIILLKKNSIQRFSLTSLSDENINVSVITISDSFIRSLKSYILG
DLMIRNLYSENKDLLLWNCEHNDIAVLSEVVNGFREINYSDEFLKVFFSG
FFSKVEKKYNSIFITDDLDAMEKISCLVKSDITRNWRWADICGELRTNRM
ILKKELESRGVKFRELINSIR ISYSISLMKTGEFKIKQIAYQSGFASVSY
FSTVFKSTMNVAPSEYLFMLTGVAEK
> P23536|PT1_RALEH Phosphoenolpyruvate -protein phosphotransferase - Ralstonia eutropha (strain ATCC 17699 / H16 / DSM 428 / Stanier 337) (Cupriavidus necator (strain ATCC 17699 / H16 / DSM 428 / Stan ier 337)).
MPFALHGIPVSRGVAIGRAHLLAPAALDVSHYLVDEDQLDAEVERLRAAR
AAVRAELAALKRDLPRDAPEELGAFLDVHAMILDDEALAREPEALIRGRR
YNAEWALTTRLEELMRQFDEIEDEYLRERKTDIRQVVERILKALAGAPVL
VPAPVPALAADGEAATGVIVVAHDIAPADMLQFRHTVFHGFVTDMGGRTS
HTAIVARSLDIPAAVGVQSASELIRQDDWIIIDGDAGLVIV DPTAIILEE
YRHRQSERALEKKRLQRLRHTPAVTLDGLEIDLLANIEMAEDAGAALAAG
AVGVGLFRSEFLFMNRRDELPGEDEQFQAYRGAVDAMHGLPVTIRTIDIG
ADKPLDARGDEFETALNPALGLRAIRWSLSEPGMFLTQLRALLRASAFGP
VRLLVPMLAHASEIDQTLALIAKAKRQLDERGEAYDPGMKVGAMIEIPAA

VLLLPLFLRKMDFLSIGTNDLIQYTLAIDRADNAVAHLFDPL HPAVLQLV
ARTIREANRAGVPVAVCGEMAGDPSMTRLLLGMGLREFSMHPAQLLRVKQ
EILHAHCERLEPLVDQVLQAFDPEEQAAALRQLARP
> P96197|LEU3_AZOVI 3-isopropylmalate dehydrogenase - Azotobacter vinelandii.
MSKQILVLPGDGIGPEIMAEAVKVLQLANEKYQLGFVLGYDELGGAAVDK
YGVPLADETLERARRRCHPARRRRRSEMGR HRPGHPPGARPAENPFATGP
VRQPAPGVLYPQLAEASTLRPEVVAGLDILIVRELTGGIYFGAPRESRLL
ANGERMAYDTLPYSESEIRRIAKVGFDMARVRGKKLCSVDKANVLASSQL
WRAVVEEVAKDYPDVVLSHMYVDNAAMQLVRAPKQFDVIVTDNMFGDILS
DEASMLTGSIGMLPSASLDADNKGMYEPCHGSRRPDIAGKGIANPLATIL
SVSMMLRYSFGQVEAANAIEQAVSKVLDQGL RTGDIWSEGCRKVGTAEMG
DAVVAALATL
> P43832|SYR_HAEIN Arginyl-tRNA synthetase - Haemophilus influenzae.
MNIQSILSDKIKQAMILAGADQSCDALIRQSGKPQFGDYQANGIMAAAKK
LGLNPREFAQKVLDNLQLSDIAEKLEIAGPGFINIFLNPTWLTTEISAAL
SHKNLGIQATNKQTVVIDYSSPNVAKEMHVGHLRSTIIGDAVARTLEFLG
HNVIRANHVGDWGTQFGMLIAYLEKMQNEHASEMELQDLEAFYREAKKHY
DEDEVFAEKARNYVVKLQSGDEYCRTMWKRLVDITMQQNQHNYARLNVTL
TEKDVMGESLYNPMLPSIVKDLKKQGLAVENDGALVVYLDEFKNKDGDPM
GVIVQKKDGGFLYTTTDIAAAKYRYETLKANRALVFSDTRQSQHMQQAWL
ITRKAGYVPDSFSLEHKNFGMMLGKDGKPFKTRTGGTVKLADLLDEAIER
ATVLINEKNTNLSNDEKEAVIEAVGIGAVKYADLSKNRTTDYVFDWDNML
SFEGNTAPYMQYAYTRIRSIFNKTDINSTALLAAPLTIKDDKERTLAIKL
LQFEEAVQTVGKEGTPHVLCAYLYELAGIFSSFYEHCPILNAEDESIKLS
RLKLALLTEKTLKQGLTLLGIKTVEKM
> CATA_PROMI P42321 CATALASE (EC 1.11.1.6). - PROTEUS MIRABIL
MEKKKLTTAAGAPVVDN NNVITAGPRGPMLLQDVWFLEKLAHFDREVIPE
RRMHAKGSGAFGTFTVTHDITKYTRAKIFSEVGKKTEMFARFSTVAGERG
AADAERDIRGFALKFYTEEGNWDMVGNNTPVFYLRDPLKFPDLNHIVKRD
PRTNMRNMAYKWDFFSHLPESLHQLTIDMSDRGLPLSYRFVHGFGSHTYS
FINKDNERFWVKFHFRCQQGIKNLMDDEAEALVGKDRESSQRDLFEAIER
GDYPRWKLQIQIMPEKEA STVPYNPFDLTKVWPHADYPLMDVGYFELNRN
PDNYFSDVEQAAFSPANIVPGISFSPDKMLQGRLFSYGDAHRYRLGVNHH
QIPVNAPKCPFHNYHRDGAMRVDGNSGNGITYEPNSGGVFQEQPDFKEPP
LSIEGAADHWNHREDEDYFSQPRALYELLSDDEHQRMFARIAGELSQASK
ETQQRQIDLFTKVHPEYGAGVEKAIKVLEGKDAK
> P06996|OMPC_ECOLI Outer membrane protein C - Escherichia coli.
MKVKVLSLLVPALLVAGAANAAEVYNKDGNKLDLYGKVDGLHYFSDNKDV
DGDQTYMRLGFKGETQVTDQLTGYGQWEYQIQGNSAENENNSWTRVAFAG
LKFQDVGSFDYGRNYGVVYDVTSWTDVLPEFGGDTYGSDNFMQQRGNGFA
TYRNTDFFGLVDGLNFAVQYQGKNGNPSGEGFTSGVTNNGRDALRQNGDG
VGGSITYDYEGFGIGGAISSSK RTDAQNTAAYIGNGDRAETYTGGLKYDA
NNIYLAAQYTQTYNATRVGSLGWANKAQNFEAVAQYQFDFGLRPSLAYLQ
SKGKNLGRGYDDEDILKYVDVGATYYFNKNMSTYVDYKINLLDDNQFTRD
AGINTDNIVALGLVYQF
> P06972|FHUB_ECOLI Ferrichrome transport system permease protein fhuB - Escherichia coli.
MSKRIALFPALLLALL VIVATALTWMNFSQALPRSQWAQAAWSPDIDVIE
QMIFHYSLLPRLAISLLVGAGLGLVGVLFQQVLRNPLAEPTTLGVATGAQ
LGITVTTLWAIPGAMASQFAAQAGACVVGLIVFGVAWGKRLSPVTLILAG
LVVSLYCGAINQLLVIFHHDQLQSMFLWSTGTLTQTDWGGVERLWPQLLG
GVMLTLLLLRPLTLMGLDDGVARNLGLALSLARLAALSLAIVISALLVNA
VGIIGFIGLFAPLLAKM LGARRLLPRLMLASLIGALILWLSDQIILWLTR
VWMEVSTGSVTALIGAPLLLWLLPRLRSISAPDMKVNDRVAAERQHVLAF
ALAGGVLLLMAVVVALSFGRDAHGWTWASGALLEDLMPWRWPRIMAALFA
GVMLAVAGCIIQRLTGNPMASPEVLGISSGAAFGVVLMLFLVPGNAFGWL
LPAGSLGAAVTLLIIMIAAGRGGFSPHRMLLAGMALSTAFTMLLMMLQAS
GDPRMAQVLTWISGSTYN ATDAQVWRTGIVMVILLAITPLCRRWLTILPL
GGDTARAVGMALTPTRIALLLLAACLTATATMTIGPLSFVGLMAPHIARM
MGFRRTMPHIVISALVGGLLLVFADWCGRMVLFPFQIPAGLLSTFIGAPY
FIYLLRKQSR
> P09489|PRTS_SERMA Extracellular serine protease - Serratia marcescens.

MILNKRLKLAYCVFLGCYGLSIHSSLAAYQDPGRLGA PDSWKTAEFNRQW
GLEAISAEFAYARGYTGKGITIGVIDNAILSHSEFSGKLTRLDNGSYNFS
YDKQDNMSFGDHGTHVAGIAAAKRDGAGMHGVAFDADIIGTKLNDYGNRN
GREELIQSAARVINNSWGIAPDIRRDAKGDIIWLPNGRPDYVAFVKSEVI
AEMMRSKSSVEWGSEQPVPTGGHSAMSTLLRAARHGKLIVFSAGNYNNYN
IPEAQKSLPYAFPDVLNNYLIVTNLSDENQLSVSSTSC GQTASYCVSAPG
SDIYSTVGRLESNTGGAVNREAYNKGELSLNPGYGNKSGTSMAAPHVTGV
AAVLMQRFPYMSADQISAVIKTTATDLGVAGIDNLFGWGRVNLRDAINGP
KMFITKEDIPQEYYVPGSYSEKQFVVNIPGLGNIVEPGTPVERRCTSSEC
SFDSWSNDISGHGGLTKTGAGTLALLGNNTYRGDTWVKQGVLAIDGSVAS
NVYIENSGTLSGEGTVGAFRAARSGSVAPGNGIGTLHVL HDAIFDRGSQY
NVEVADNGRSDKIAARRAFLNGGSVNVSLERSQNLLSQNEAQSLLGNKYT
ILTTTDGVTGRFENANPSYPFVKVALDYRGNDVGLGITRTDASFDSLAST
ENEKAVARAVETLNATEPVTETAKRSVAIPAAEEANLLQSDGGEAQAVNE
EASIVAGHPIYESFLGFTSARELQQATRQLSGQIHADMASAQINESRYLR
DTATERLRQAEGRRTATDIKADDNGAWAKLLGSWGHASGN DNATGYQTST
YGVLLGLDSELFGDGRLGMMTGYTRTSLDGGYQSDAHSDNYHLGLYGDKR
FGALALRAGGTYTWHRIDTSRSVNYGAQSDREKAKYNARTGQLFIESGYD
WTSDAVNLEPFANLAYTHYRNEEINEQGGAAALRGDKQSQSATASTLGLR
ADTEWQTDSVAIALRGELGWQHQYGKLERKTQLMFKRTDAAFDVNSVPVS
RDGAILKAGVDVSINKNAVLSLGYGGQLSSNHQDNSVNAGL TWRF
> P44529|IPYR_HAEIN Inorganic pyrophosphatase - Haemophilus influenzae.
MADFNQILTPGDVDAGIINVVNEIPEGSCHKIEWNRKVAAFQLDRVEPAI
FAKPTNYGFIPQTLDEDGDELDVLLITRQPLATGVFLEAKVIGVMKFVDD
GEVDDKIVCVPADDRDTGNAYNSLADLPANLIKQIEFHFNNYKALKKPGS
TKVTHWGDVEEAKEVIRESIKRWNER
> P20379|SODC_CAUCR Superoxide dismutase [Cu -Zn] - Caulobacter crescentus (Caulobacter vibrioides).
MIRLSAAAALGLAAALAASPALAQTSATAVVKAGDGKDAGAVTVTEAPHG
VLLKLELKGLTPGWHAAHFHEKGDCGTPDFKSAGAHVHTAATTVHGLLNP
DANDSGDLPNIFAAADGAATAEIYSPLVSLKGAGGRPALLDADGSSIVVH
ANPDDHKTQPIGGAGARVACGVIK
> P18668|EFTU_SYNP6 Elongation factor Tu - Synechococcus sp. (strain ATCC 27144 / PCC 6301 / SAUG 1402/1) (Anacystis nidulans).
MARAKFERTKPHANIGTIGHVDHGKTTLTAAITTVLAKAGMAKARAYADI
DAAPEEKARGITINTAHVEYETGNRHYAHVDCPGHADYVKNMITGAAQMD
GAILVVSAADGPMPQTREHILLAKQVGVPNIVVFLNKEDMVDDAELLELV
ELEVRELLSSYDFPGDDIPIVAGSALQALEAIQGGASGQKGDNPWVDKIL
KLMEEVDAYIPTPEREVDRPFLMAVEDVFTITGRGTVATGRIERGSVKVG
ETIEIVGLRDTRSTTVTGVEMFQKTLDEGLAGDNVGLLLRGIQKTDIERG
MVLAKPGSITPHTKFESEVYVLKKEEGGRHTPFFPGYRPQFYVRTTDVTG
AISDFTADDGSAAEMVIPGDRIKMTVELINPIAIEQGMRFAIREGGRTIG
AGVVSKILQ
> P0AG78|SUBI_ECOLI Sulfate -binding protein - Escherichia coli.
MNKWGVGLTFLLAATSVMAKDIQLLNVSYDPTRELYEQYNKAFSAHWKQQ
TGDNVVIRQSHGGSGKQATSVINGIEADVVTLALAYDVDAIAERGRIDKE
WIKRLPDNSAPYTSTIVFLVRKGNPKQIHDWND LIKPGVSVITPNPKSSG
GARWNYLAAWGYALHHNNNDQAKAQDFVRALYKNVEVLDSGARGSTNTFV
ERGIGDVLIAWENEALLAANELGKDKFEIVTPSESILAEPTVSVVDKVVE
KKGTKEVAEAYLKYLYSPEGQEIAAKNYYRPRDAEVAKKYENAFPKLKLF
TIDEEFGGWTKAQKEHFANGGTFDQISKR
> P13507|AMT4_PSEST Glucan 1,4 -alpha-maltotetraohydrolase - Pseudomonas stutzeri (Pseudomonas perfectomarina).
MSHILRAAVLAAMLLPLPSMADQAGKSPNAVRYHGGDEIILQGFHWNVVR
EAPNDWYNILRQQAATIAADGFSAIWMPVPWRDFSSWSDGSKSGGGEGYF
WHDFNKNGRYGSDAQLRQAASALGGAGVKVLYDVVPNHMNRGYPDKEINL
PAGQGFWRNDCADPGNYPNDCDDGDRFIGGDADLNTGHPQVYGMFRD EFT
NLRSQYGAGGFRFDFVRGYAPERVNSWMTDSADNSFCVGELWKGPSEYPN
WDWRNTASWQQIIKDWSDRAKCPVFDFALKERMQNGSIADWKHGLNGNPD
PRWREVAVTFVDNHDTGYSPGQNGGQHHWALQDGLIRQAYAYILTSPGTP
VVYWSHMYDWGYGDFIRQLIQVRRAAGVRADSAISFHSGYSGLVATVSGS
QQTLVVALNSDLGNPGQVASGSFSEAVNASNGQVRVWRSGTGSGGGEP GA
LVSVSFRCDNGATQMGDSVYAVGNVSQLGNWSPAAALRLTDTSGYPTWKG

SIALPAGQNEEWKCLIRNEANATQVRQWQGGANNSLTPSEGATTVGRL
> P39049|ALXM_PHOS4 Alginate lyase - Photobacterium sp. (strain ATCC 433367).
MIKSNLVISSLAIVSSMSYAGVEFSNPSGQLGEPANYTQFANILSASELQ
ISDPNGKKGNKEYFALDNDFTGIV NDNFYVDKQSQALVFKMANDHLRNEL
RVQKNFRTDLPDHFYTLYANVEILHPLQSMANSTSKQNEITFLQVHNKGL
DDQGTHNVPHPLLRVVWKENNQGVKGHFWAITKNNAVICKGSFGKKNKDK
EMCRADVAYSKIDLGPAPTDKGTDFTITVGNKTLAIDVNGQRKVEKNIDY
WRHLLSYFKAGVYNQFTQGESEAHFNQLRYQVNTP
> P96142|SYV_THETH Valyl -tRNA synthetase - Thermus thermophilus.
MDLPKAYDPKSVEPKWAEKWAKNPFVANPKSGKPPFVIFMPPPNVTGSLH
MGHALDNSLQDALIRYKRMRGFEAVWLPGTDHAGIATQVVVERLLLKEGK
TRHDLGREKFLERVWQWKEESGGTILKQLKRLGASADWSREAFTMDEKRS
RAVRYAFSRYYHEGLAYRAPRLVNWCPRCETTLSDLEVETEPTPGKLYTL
RYEVEGGGFIEIATVRPETVFADQAIA VHPEDERYRHLLGKRARIPLTEV
WIPILADPAVEKDFGTGALKVTPAHDPLDYEIGERHGLKPVSVINLEGRM
EGERVPEALRGLDRFEARRKAVELFREAGHLVKEEDYTIALATCSRCGTP
IEYAIFPQWWLRMRPLAEEVLKGLRRGDIAFVPERWKKVNMDWLENVKDW
NISRQLWWGHQIPAWYCEDCQAVNVPRPERYLEDPTSCEACGSPRLKRDE
DVFDTWFSSALWPLSTLGWPEETEDLKA FYPGDVLVTGYDILFLWVSRME
VSGYHFMGERPFKTVLLHGLVLDEKGQKMSKSKGNVIDPLEMVERYGADA
LRFALIYLATGGQDIRLDLRWLEMARNFANKLYNAARFVLLSREGFQAKE
DTPTLADRFMRSRLSRGVEEITALYEALDLAQAAREVYELVWSEFCDWYL
EAAKPALKAGNAHTLRTLEEVLAVLLKLLHPMMPFLTSELYQALTGKEEL
ALEAWPEPGGRDEEAERAFEALKQAVTAV RALKAEAGLPPAQEVRVYLEG
ETAPVEENLEVFRFLSRADLLPERPAKALVKAMPRVTARMPLEGLLDVEE
WRRRQEKRLKELLALAERSQRKLASPGFREKAPKEVVEAEEARLKENLEQ
AERIREALSQIG
> P50199|GNO_GLUOX Gluconate 5 -dehydrogenase - Gluconobacter oxydans (Gluconobacter suboxydans).
MSHPDLFSLSGARALVTGASRG IGLTLAKGLARYGAEVVLNGRNAESLDS
AQSGFEAEGLKASTAVFDVTDQDAVIDGVAAIERDMGPIDILINNAGIQR
RAPLEEFSRKDWDDLMSTNVNAVFFVGQAVARHMIPRGRGKIVNICSVQS
ELARPGIAPYTATKGAVKNLTKGMATDWGRHGLQINGLAPGYFATEMTER
LVADEEFTDWLCKRTPAGRWGQVEELVGAAVFLSSRASSFVNGQVLMVDG
GITVSL
> P17835|FM3_BORPE Serotype 3 fimbrial subunit - Bordetella pertussis.
MSKFSYPALRAALILAASPVLPALANDGTIVITGSISDQTCVIEEPSTLN
HIKVVQLPKISKNALRNDGDTAGATPFDIKLKECPQALGALKLYFEPGIT
TNYDTGDLIAYKQTYNASGNGNLSTVSSATKAKGVEFRLANLNGQHIRMG
TDKTTQAAQTFTGKVTNGSKSYTLRYLASYVKKPKEDVDAAQITSYVG FS
VVYP
> O84686|EFTS_CHLTR Elongation factor Ts - Chlamydia trachomatis.
MSDFSMETLKNLRQQTGVGLTKCKEALEHAKGNLEDAVVYLRKLGLASAG
KKEHRETKEGVIAARVDERGAALVEVNVETDFVANNNVFRAFVTSLLSDL
LDHELSDVDALALVMSSQEPSLSVEELKAVTMQTVGENIRISRAFYTPVN
SGQSVGIYSHGNGKAVAIAFLSGSENQEA LAKDIAMHIVASQPQFLSKES
VPQEVLEREREVFSSQVAGKPQEVVEKITQGKFRAFFQEACLLEQAFIKD
PEVTIQGLIDRAAKASGEPLKVEHFVFWKMGA
> P16481|PTHP_KLEPN Phosphocarrier protein HPr - Klebsiella pneumoniae.
MFQQEVTITAPNGLHTRPAAQFVKEAKGFTSEITVTSNGKSASAKSLFKL
QTLGLTQGTVVTLSAEGEDEQKAVEHL VKLMAELE
> P31101|HCP_DESVH Hydroxylamine reductase - Desulfovibrio vulgaris (strain Hildenborough / ATCC 29579 / NCIMB 8303).
MFCFQCQETAKNTGCTVKGMCGKPEETANLQDLLIFVLRGIAIYGEKLKE
LGQPDRSNDDFVLQGLFATITNANWDDARFEAMISEGLARRDKLRNAFLA
VYKAKNGKDFSEPLPEAATWTGDSTAF AEKAKSVGILATENEDVRSLREL
LIIGLKGVAAYAEHAAVLGFRKTEIDEFMLEALASTTKDLSVDEMVALVM
KAGGMAVTTMALLDEANTTTYGNPEITQVNIGVGKNPGILISGHDLKDMA
ELLKQTEGTGVDVYTHGEMLPANYYPAFKKYPHFVGNYGGSWWQQNPEFE
SFNGPILLTTNCLVPLKKENTYLDRLYTTGVVGYEGAKHIADRPAGGAKD
FSALIAQAKKCPPPVEIETGSIVGGFAH HQVLALADKVVEAVKSGAIKRF
VVMAGCDGRQKSRSYYTEVAENLPKDTVILTAGCAKYRYNKLNLGDIGGI

PRVLDAGQCNDSYSLAVIALKLKEVFGLDDINDLPVSYDIAWYEQKAVAV
LLALLFLGVKGIRLGPTLPAFLSPNVAKVLVENFNIKPIGTVQDDIAAMM
AGK
> FES_ECOLI P13039 ENTEROCHELIN ESTERASE. - ESCHERICHIA COLI.
MFEVTFWWRDPQGS EEYSTIKRVWVYITGVTDHHQNSQPQSMQRIAGTNV
WQWTTQLNANWRGSYCFIPTERDDIFSVPSPDRLELREGWRKLLPQAIAD
PLNLQSWKGGRGHAVSALEMPQAPLQPGWDCPQAPEIPAKEIIWKSERLK
KSRRVWIFTTGDATAEERPLAVLLDGEFWAQSMPVWPVLTSLTHRQQLPP
AVYVLIDAIDTTHRAHELPCNADFWLAVQQELLPLVKAIAPFSDRADRTV
VAGQSFGGLSALYAG LHWPERFGCVLSQSGSYWWPHRGGQQEGVLLEKLK
AGEVSAEGLRIVLEAGIREPMIMRANQALYAQLHPIKESIFWRQVDGGHD
ALCWRGGLMQGLIDLWQPLFHDRS
> P77849|KDSA_CHLTR 2 -dehydro-3-deoxyphosphooctonate aldolase - Chlamydia trachomatis.
MFPENKMLLIAGPCVIEDNSVFETARRLKEIVAPYASSVHWIFKSSYDKA
NRSSVHNYRGPGLKLGLQTLAKIKEELDVEILTDVHSPDEAREAAKVCDI
IQVPAFLCRQTDLLVTAGETQAIVNIKKGQFLSPWEMQGPIDKVLSTGNN
KIILTERGCSFGYNNLVSDMRSIEVLRRFGFPVVFDGTHSVQLPGALHSQ
SGGQTEFIPVLTRSAIAAGVQGLFIETHPNPSSALSDAASMLSLKDLERL
LPAWVQLFTYIQEMDAVSV
> P04042|CHEB_SALTY Chemotaxis respons e regulator protein -glutamate methylesterase - Salmonella typhimurium.
MSKIRVLSVDDSALMRQIMTEIINSHSDMEMVATAPDPLVARDLIKKFNP
DVLTLDVEMPRMDGLDFLEKLMRLRPMPVVMVSSLTGKGSEVTLRALELG
AIDFVTKPQLGIREGMLAYSEMIAEKVRTAARARIAAHKPMAAPTTLKAG
PLLSSEKLIAIGASTGGTEAIRHVLQPLPLSS PAVIITQHMPPGFTRSFA
ERLNKLCQISVKEAEDGERVLPGHAYIAPGDKHMELARSGANYQIKIHDG
PPVNRHRPSVDVLFHSVAKHAGRNAVGVILTGMGNDGAAGMLAMYQAGAW
TIAQNEASCVVFGMPREAINMGGVSEVVDLSQVSQQMLAKISAGQAIRI
> P15319|PAPD_ECOLI Chaperone protein papD - Escherichia coli.
MIRKKILMAAIPLFVISGADAA VSLDRTRAVFDGSEKSMTLDISNDNKQL
PYLAQAWIENENQEKIITGPVIATPPVQRLEPGAKSMVRLSTTPDISKLP
QDRESLFYFNLREIPPRSEKANVLQIALQTKIKLFYRPAAIKTRPNEVWQ
DQLILNKVSGGYRIENPTPYYVTVIGLGGSEKQAEEGEFETVMLSPRSEQ
TVKSANYNTPYLSYINDYGGRPVLSFICNGSRCSVKKEK
> P37726|PORF_PSEFL Outer membrane porin F - Pseudomonas fluorescens.
MKLKNTLGFAIGSIIAATSFGALAQGQGAVEGELFYKKQYNDSVKHIEDG
FNPGARIGYFLTDDLSLNLSYDKTNHTRSNDGTGSQKIGGDTSSLTAQYH
FGQAGVDSLRPYVEGGFGHQSRGNVKADGHSGRDQSTLAIAGAGVKYYFT
NNVYARAGVEADYALDNGKWDYSALVGLGVNFGGNAGAAAPAPTPAPAPE
PTPEPEAPVAQVVRVE LDVKFDFDKSVVKPNSYGDVKNLADFMAQYPATN
VEVAGHTDSIGPDAYNQKLSQRRADRVKQVLVKDGVAPSRITAVGYGESR
PVADNATEAGRAVNRRVEASVEAQAQ
> P43821|SYGA_HAEIN Glycyl -tRNA synthetase alpha chain - Haemophilus influenzae.
MSTKFNVKTFQGMILALQEYWANQGCTIVQPFDMEVGAGTSHPMTALRAL
GPEPMAFAYVQ PSRRPTDGRYGENPNRLQHYYQFQVVIKPSPDNIQELYL
GSLEMLGFDPTKNDIRFVEDNWENPTLGAWGLGWEVWLNGMEVTQFTYFQ
QVGGLECKPVTGEVTYGLERLAMYIQGVDSVYDLVWSDGPLGKTTYGDVF
HQNEVEQSTYNFEHANTDFLFYCFDQYEKEAQELLALEKPLPLPAYERIL
KAAHSFNLLDARKAISVTERQRYILRIRALTKGVAEAYYASREALGFPGC
KK
> P0A6P1|EFTS_ECOLI Elongation factor Ts - Escherichia coli.
MAEITASLVKELRERTGAGMMDCKKALTEANGDIELAIENMRKSGAIKAA
KKAGNVAADGVIKTKIDGNYGIILEVNCQTDFVAKDAGFQAFADKVLDAA
VAGKITDVEVLKAQFEEERVALVAKIGENINIRRVAALEGDVLGSYQHGA
RIGVLVAAKGADEELVKHIAMHVAASKPEFIKPEDVSAEVVEKEYQVQLD
IAMQSGKPKEIAEKMVEGRMKKFTGEVSLTGQPFVMEPSKTVGQLLKEHN
AEVTGFIRFEVGEGIEKVETDFAAEVAAMSKQS
> P46739|NFAA_ECOLI Nonfimbrial adhesin 1 - Escherichia coli.
MKAKKYENQIYNENGRRCQRHGRRLAIADANGLNTVNAGDGKNLGTATAT
ITTLQSCSVDLNLVTPNATVNRAGMLANREITKFSVGSKDCPSDTYAVWF
KEIDGEGQGVAQGTTVTNKFYLKMTSADGTASVGDINIGTKSGKGLSGQL

VGGKFDGKITVAYDSATAPADVYTYDLMAAVYVQ
> FDHD_WOLSU P28181 FDHD PROTEIN. - WOLINELLA SUCCINOGENES.
MRHTDRFVKKVVIERIGDQRVLAEEEDVVIKEERISLYLNGTKLMSMMSL
PSDQDAHAVGFLMSEGVIEKIEDLKSVQISSDGSSVYVEALINHENITNL
FKEKTLTSGCCVGVT GNLEGNVLRKFIATPMQISLERIWEGMEEFEMSSH
LFHETGCVHKASLLLEDGSKITAEDIGRHNAIDKVMGKARLGRIDTEKAV
LVVSGRLSMEMVVKAVMHNIPMIVSRAAATFLGIKTAQELGVTLVGFARG
EKMNIYTHSGRVDLRACKRKRGVTLHAPNQSSSLLR
> P61316|LOLA_ECOLI Outer-membrane lipoprotein carrier protein - Escherichia coli.
MKKIAITCALLSSLVASSVWADAASDLKSRLDKVSSFHASFTQKVTDGSG
AAVQEGQGDLWVKRPNLFNWHMTQPDESILVSDGKTLWFYNPFVEQATAT
WLKDATGNTPFMLIARNQSSDWQQYNIKQNGDDFVLTPKASNGNLKQFTI
NVGRDGTIHQFSAVEQDDQRSSYQLKSQQNGAVDAAKFTFTPPQGVTVDD
QRK
> P25910|BLAB_BACFR Beta-lactamase type II - Bacteroides fragilis.
MKTVFILISMLFPVAVMAQKSVKISDDISITQLSDKVYTYVSLAEIEGWG
MVPSNGMIVINNHQAALLDTPINDAQTEMLVNWVTDSLHAKVTTFIPNHW
HGDCIGGLGYLQRKGVQSYANQMTIDLAKEKGLPVPEHGFTDSLTVSLDG
MPLQCYYLGGGHATDNIVVWLPTENILFGGCMLKDNQATSIGNISDADVT
AWPKTLDKVKAKFPSARYVVPGHGDYGGTEL IEHTKQIVNQYIESTSKP
> P70865|SYA_BARBA Alanyl-tRNA synthetase - Bartonella bacilliformis.
MNSVNNIRSTFLDYFHRNGHEVLSSSPLVPRNDPTLMFTNAGMVDFKNVF
TGLEKHSYNRATTAQKCVRAGGKHNDLDNVGYTARHHTFFEMLGNFSFSN
YFKEEAIFYAWNLLTKEFCLSKDKLLVTVYHDDDVAAGLWRKISGLSEEK
IIRIATNDNFWMMG DTGPCGPCSEIFYDHGDKIWGGPPGSADEDGDRFIE
IWNLVFMQYEQLSKEKRIELPQPSIDTGMGLERIAAVLQGVHDNYDIDLF
RTLIHASQEIIGVKATGDFFASHRVIADHLRSSAFLIADGIMPSNEGRGY
VLRRIMRRAMRHAHLLGSKDLLMWRLVPVLISEMGQAYPELVRAESLISE
ILKLEETRFRKTLERGLGLLNEASTHLEEGDYFNGEVAFKLYDTYGFPLD
LTQDALRRRGISVDV DAFDKAMKRQKAEARANWSGSGDCVTETVWFSIRD
QVGATEFLGYETEKAEGIITALIRDGEVVDHIDLGQKAMIVVNQTPFYGE
SGGQVGDSGIISGANFIFEVHDTQKKGDNVFIHIGEIKTGQAKKHDCVEL
IVDSARRRKIRANHSATHLLHESLRQTLGSHVVQKGSFVSPDRLRFDFSH
PKSISSEELKKIEDLANDIVLQNSKVTTRLMAIDDAIAEGAMALFGEKYG
DEVRVISMGNNLEQTG SKKWWSIELCGGTHVQRTGDIGLIHIISETSVAA
GVRRIEALTATAARLYLHGQDRRVYEIAGLLKTSPADVQERVQTLLDERR
KLEKELNDSRKKIALNGGSVNSQGDIQTINGISFMGGVVSNILPKDLKAL
VDSGKKKIGSGVVAFISVSEDGKGSAVVGVTDDLTDTLNAVDLVRIISVT
LGGQGGGGRRDMAQAGGSEGGKADEALVALKDSLKG
> P50203|PHAB_ACISR Acetoacetyl-CoA reductase - Acinetobacter sp. (strain RA3849).
MSEQKVALVTGALGGIGSEICRQLVTAGYKIIATVVPREEDREKQWLQSE
GFQDSDVRFVLTDLNNHEAATAAIQEAIAAEGRVDVLVNNAGITRDATFK
KMSYEQWSQVIDTNLKTLFTVTQPVFNKMLEQKSGRIVNISSVNGLKGQF
GQANYSASKAGIIGFTKALAQEGARSNICVNVVAPGYTATPMVTAMREDV
IKSIEAQIPLQRLAAPAEIAAAVMYLVSEHGAYVTGETLSINGGLYMH
> P0A1F0|DDLA_SALTY D-alanine--D-alanine ligase A - Salmonella typhimurium.
MAKLRVGIVFGGKSAEHEVSLQSAKNIVDAIDKTRFDVVLLGIDKAGQWH
VNDAENYLQNADDPAHIALRPSAISLAQVPGKHQHQLINAQNGQPLPTVD
VIFPIVHGTLGEDGSLQGMLRVANLPFVG SDVLSSAACMDKDVAKRLLRD
AGLNIAPFITLTRTNRHAFSFAEVESRLGLPLFVKPANQGSSVGVSKVAN
EAQYQQAVALAFEFDHKVVVEQGIKGREIECAVLGNDNPQASTCGEIVLN
SEFYAYDTKYIDDNGAQVVVPAQIPSEVNDKIRAIAIQAYQTLGCAGMAR
VDVFLTADNEVVINEINTLPGFTNISMYPKLWQASGLGYTDLISRLIELA
LERHTANNALKTTM
> P18190|MBHS_AZOCH Uptake hydrogenase small subunit - Azotobacter chroococcum mcd 1.
MSQLETXYDVMRRQGITRRSFLKYCSLTGRPCLGPTFAPQIAHAMETRPP
TPVVWLHGLECTCCSESFIRSGDPLVKDVVLSMISLDYDDTLMPPRHQGT
VEETMRKYKGEYILAVEGNPPLNEDGMFCIVGGKPFLDQLKHAAKDAKAV
IAWGSCASWGCVQAAKPNPTQAVPIHKVITDK PMIKVPGCPPIAEVMTGV
ITYMLTFGKLPELDRQGRPKMFYGQRIHDKSYRRPHFDAGQFVEHWDDEG

ARKGYCLYKVGCKGPTSYNACSTVRWNEGTSFPIQAGHGCIGCSEDGFWD
KGSFYERLTTIPQFGIEKNADQIGPRGRRGSGAAIAAHAAVTAIKRLQNK
GDQA
> P21982|FLAA_SPIAU Flagellar filament outer layer protein - Spirochaeta aurantia.
MKRFFAILGAALFVGNSGAFAEQATLIDFSKLVGEGNTGLHAPTTIDYSR
QAGSAYSAEDKAAMKISLAIPSWEIELASSSQTVENQTLSLVTAAPVKQD
AARYGGETVMGVRIHFPSFGINSFAVIKPPFTIPAYATLGDATAQNAVAG
GQFDGFGVLKNVGVIKSIQINILGRNYLNRLSLLLEDQNGDEREIVMGYL
NFDGWKSLQWNNPNYQTEVRNRDLQIVPLYPRSAPLIKLKGIKIHRDG SQ
EGGDIVSYIKDIKVIYDQAVVDRNSDVDDEAIWGILRQREEQYRNFELAK
LGNLQVLRSLEKKKMAKEADFDQAAPAAAAARAPATN
> P0AA19|OMPR_SALTY Transcriptional regulatory protein ompR - Salmonella typhimurium.
MQENYKILVVDDDMRLRALLERYLTEQGFQVRSVANAEQMDRLLTRESFH
LMVLDLMLPGEDGLSICRRLRSQSNPM PIIMVTAKGEEVDRIVGLEIGAD
DYIPKPFNPRELLARIRAVLRRQANELPGAPSQEEAVIAFGKFKLNLGTR
EMFREDEPMPLTSGEFAVLKALVSHPREPLSRDKLMNLARGREYSAMERS
IDVQISRLRRMVEEDPAHPRYIQTVWGLGYVFVPDGSKA
> P27002|SYFB_THETH Phenylalanyl -tRNA synthetase beta chain - Thermus thermophilus.
MRVPFSWLKAYVPELESPEVLEERLAGLGFETDRIERVFPIPRGVVFARV
LEAHPIPGTRLKRLVLDAGRTVEVVSGAENARKGIGVALALPGTELPGLG
QKVGERVIQGVRSFGMALSPRELGVGEYGGGLLEFPEDALPPGTPLSEAW
PEEVVLDLEVTPNRPDALGLLGLARDLHALGYALVEPEAALKAEALPLPF
ALKVEDPEGAPHFTLGYAFGLRVAPSPLWMQRALFAAGMRPINNVVDVTN
YVMLERAQPMHAFDLRFVGEGIAVRRAREGERLKTLDGVERTLHPEDLVI
AGWRGEESFPLGLAGVMGGAESEVREDTEAIALEVACFDPVSIRKTARRH
GLRTEASHRFERGVDPLGQVPAQRRALSLLQALAGARVAEALLEAGSPKP
PEAIPFRPEYANRLLGTSYPEAEQIAILKRLGCRVEGEGPTYRVTPPSHR
LDLRLEEDLVEEVARIQGYETIPLALPAFFPAPDNRGVEAPYRKEQRLRE
VLSGLGFQEVYTYSFMDPEDARRFRLDPPRLLLLNPLAPEKAALRTHLFP
GLVRVLKENLDLDRPERALLFEVGRVFREREETHLAGLLFGEGVGLPWAK
ERLSGYFLLKGYLEALFARLGLAFRVEAQAFPFLHPGVSGRVLVEGEEVG
FLGALHPEIAQELELPPVHLFELRLPLPDKPLAFQDPSRHPAAFRDLAVV
VPAPTPYGEVEALVREAAGPYLESLALFDLYQGPPLPEGHKSLAFHLRFR
HPKRTLRDEEVEEAVSRVAEALRARGFGLRGLDTP
> P40120|OPGD_ECOLI Glucans biosynthesis protein D - Escherichia coli.
MDRRRFIKGSMAMAAVCGTSGIASLFSQAAFAADSDIADGQTQRFDFSIL
QSMAHDLAQTAWRGAPRPLPDTLATMTPQAYNSIQYDAEKSLWHNVENRQ
LDAQFFHMGMGFRRRVRMFSVDPATHLAREIHFRPELFKYNDAGVDTKQL
EGQSDLGFAGFRVFKAPELARRDVVSFLGASYFRAVDDTYQYGLSARGLA
IDTYTDSKEEFPDFTAFWFDTVKPGATTFTVYALLDSASITGAYKFTIHC
EKSQVIMDVENHLYARKDIKQLGIAPMTSMFSCGTNERRMCDTIHPQIHD
SDRLSMWRGNGEWICRPLNNPQKLQFNAYTDNNPKGFGLLQLDRDFSHYQ
DIMGWYNKRPSLWVEPRNKWGKGTIGLMEIPTTGETLDNIVCFWQPEKAV
KAGDEFAFQYRLYWSAQPPVHCPLARVMATRTGMGGFSEGWAPGEHYPEK
WARRFAVDFVGGDLKAAAPKGIEPVITLSSGEAKQIEILYIEPIDGYRIQ
FDWYPTSDSTDPVDMRMYLRCQGDAISETWLYQYFPPAPDKRQYVDDRVM
S
> ISPA_HAEIN P45204 GERANYLTRANSTRANSFERASE (EC 2.5.1.10) (FA
MGHFSEELQQVQTRINRFLEAQFEGIESHNAPLLEAMKYALLLGG KRVRP
FLVYATGQMLGAEKQTLDYAAAAIEAIHAYSLIHDDLPAMDDDNLRRGHP
TCHIQFDEATAILAGDALQSFAFEILTKTPNISTEQKLALIQILAQGAGV
QGMCLGQSLDLISEHKQISLSELELIHRNKTGALLIAALKLGFICSPHFT
DKRLEQSLTQYAEAIGLAFQVQDDILDIEGDSAEIGKQVGADLDLDKSTY
PKLLGLSGAKQKAQDLYQSALSELEKIPFDTTVRALAEFIITRKS
> P41255|SYK_THETH Lysyl -tRNA synthetase - Thermus thermophilus.
MNDQTRQRLLNLEALVEAGFAPYPYRFPKTHSAEAILKAKRGAPPESEWP
EEEVAVAGRLVALRRMGKVTFAHLLDETGRIQLYFQRDLTPKYELLKKLD
VGDILGVRGHPFTTKTGEVTVKVLDWTPLVKSLHPLPDKWHGLRDKEVRY
RQRYLDLIVNPEVREVFRRRSEIVRYIRRFFEAKGFLE VETPILQPTTGG
AEARPFKTYHNALDHEFYLRISLELYLKRLLVGGYEKVFEIGRNFRNEGI

DHNHNPEFTMLEAYWAYADYQDMAGLVEELLSGLVLHLFGSHEVPYQGRV
LNFKPPFRRISFVEALKEKAGLPFDPLDLERLRLWADAHHPELSQVPNYK
LLDKLFGIYVEPELQDPTFVFDFPLAISPLAKRHREKPGLVERWDLYAGG
MELAPCYSELNDPLDQRERFLEQARRRKEGDEEAPEPDE DFLLALEYGMP
PAAGLGLGIDRLAMLLTDQPSLRDVLLFPLLKPKKEAVEEGV
> P40601|LIP1_PHOLU Lipase 1 - Photorhabdus luminescens (Xenorhabdus luminescens).
MKRSFIFAPGMLALSISAISNAHAYNNLYVFGDSLSDGGNNGRYTVDGIN
GTESKLYNDFIAQQLGIELVNSKKGGTNYAAGGATAVADLNNKHNTQDQV
MGYLASHSNRADHNGM YVHWIGGNDVDAALRNPADAQKIITESAMAASSQ
VHALLNAGAGLVIVPTVPDVGMTPKIMEFVLSKGGATSKDLAKIHAVVNG
YPTIDKDTRLQVIHGVFKQIGSDVSGGDAKKAEETTKQLIDGYNELSSNA
SKLVDNYNQLEDMALSQENGNIVRVDVNALLHEVIANPLRYGFLNTIGYA
CAQGVNAGSCRSKDTGFDASKPFLFADDFHPTPEAHHIVSQYTVSVLNAP
YRVMLLTNANNVPVKGA LASLDGRLQQLRNVDNEQGKLGVFGGYSGNHSH
TLTLGSDYQIMDNILLGGMISRYQDNSSPADNFHYDGRGYVFTAYGLWRY
YDKGWISGDLHYLDMKYEDITRGIVLNDWLRKENASTSGHQWGGRITAGW
DIPLTSAVTTSPIIQYAWDKSYVKGYRESGNNSTAMHFGEQRYDSQVGTL
GWRLDTNFGYFNPYAEVRFNHQFGDKRYQIRSAINSTQTSFVSESQKQDT
HWREYTIGMNAVITKDWG AFASISRNDGDVQNHTYSFSLGVNASF
> P0AEM9|FLIY_ECOLI Cystine -binding periplasmic protein - Escherichia coli.
MKLAHLGRQALMGVMAVALVAGMSVKSFADEGLLNKVKERGTLLVGLEGT
YPPFSFQGDDGKLTGFEVEFAQQLAKHLGVEASLKPTKWDGMLASLDSKR
IDVVINQVTISDERKKKYDFSTPYTISGIQALVKKGNEGTIKTADDLKGK
KVGVGLGTNYEEWLRQNVQGVDVRTYDDDPTKYQDLRVGRIDAILVDRLA
ALDLVKKTNDTLAVTGEAFSRQESGVALRKGNEDLLKAVNDAIAEMQKDG
TLQALSEKWFGADVTK
> P05695|PORP_PSEAE Porin P - Pseudomonas aeruginosa.
MIRRHSCKGVGSSVAWSLLGLAISAQSLAGTVTTDGADIVIKTKGGLEVA
TTDKEFSFKLGGRLQADYGRFDGYYTNNGNT ADAAYFRRAYLEFGGTAYR
DWKYQINYDLSRNVGNDSAGYFDEASVTYTGFNPVNLKFGRFYTDFGLEK
ATSSKWVTALERNLTYDIADWVNDNVGTGIQASSVVGGMAFLSGSVFSEN
NNDTDGDSVKRYNLRGVFAPLHEPGNVVHLGLQYAYRDLEDSAVDTRIRP
RMGMRGVSTNGGNDAGSNGNRGLFGGSSAVEGLWKDDSVWGLEGAWALGA
FSAQAEYLRRTVKAERDREDLKASGYYAQLAY TLTGEPRLYKLDGAKFDT
IKPENKEIGAWELFYRYDSIKVEDDNIVVDSATREVGDAKGKTHTLGVNW
YANEAVKVSANYVKAKTDKISNANGDDSGDGLVMRLQYVF
> P69791|PTQA_ECOLI N,N' -diacetylchitobiose -specific phosphotransferase enzyme IIA component - Escherichia coli.
MMDLDNIPDTQTEAEELEEVVMGLIINSGQAR SLAYAALKQAKQGDFAAA
KAMMDQSRMALNEAHLVQTKLIEGDAGEGKMKVSLVLVHAQDHLMTSMLA
RELITELIELHEKLKA
> P13394|SCRB_VIBAL Sucrose -6-phosphate hydrolase - Vibrio alginolyticus.
MSLNNRWTVEQRYRRLEQIPQCDIEEMTLSRQQDKGFPSFHIAPKFGLLN
DPNGLCYFNGEHHIFYQWTPVGPVHGMKYWYHLSTKDFIHFTDH GVGLHP
DQDYDSHGVYSGGALVENNQVLLFFTGNKRDQNWNRIPTQCFATMDSDGS
IEKHGVVIENEHYTEHFRDPKVWKKGDDYLMVVGAQTKTEHGSMALYQSK
DLKTWQHKGPIKTKFSDLGYMWECPDFFEINGQSVMLFSPQGVSSSNPYD
FKNIYSVAYIVGDQLNLESMTLENHQDILQPDYGFDFYAPQTYLDESGRR
ILIAWIGLPEIDTPSVTHQWAGMLSLPRELTLKDGFLVQTPLPEL KSLRK
EEVVFAQSHTLESTSCLIQLDLVGDGFELELSNLKGDNIVFSATEHEFML
DRRYMSHLYAEEFGGIRKAPRLDAKQTIDIYIDNSVIEIFINGGKHTMTS
RFFIDDLNKVTLKGLEQARLFPLKGITGLFESAK
> P0A1D4|CH60_SALTI 60 kDa chaperonin - Salmonella typhi.
MAAKDVKFGNDARVKMLRGVNVLADAVKVTLGPKGRNVVLDKSFGAPTIT
KDGVSVAREIELEDKFENMGAQMVKEVASKANDAAGDGTTTATVLAQSII
TEGLKAVAAGMNPMDLKRGIDKAVAAAVEELKALSVPCSDSKAIAQVGTI
SANSDETVGKLIAEAMDKVGKEGVITVEDGTGLQDELDVVEGMQFDRGYL
SPYFINKPETGAVELESPFILLADKKISNIREMLPVLEAVAKAGKPLLII
AEDVEGEALATLVVNTMRGIVKVAAVKAPGFGDRRKAMLQDIATLTGGTV
ISEEIGMELEKATLEDLGQAKRVVINKDTTTIIDGVGEEAAIQGRVAQIR
QQIEEATSDYDREKLQERVAKLAGGVAVIKVGAATEVEMKEKKARVEDAL
HATRAAVEEGVVAGGGVALIRVASKIADLKGQNEDQNVGIKVALRAMEAP

LRQIVLNCGEEPSVVANTVKGGDGNYGYNAATEEYGNMIDMGILDPTKVT
RSALQYAASVAGLMITTECMVTDLPKSDAPDLGAAGGMGGMGGMGGMM
> P69828|PTKA_ECOLI Galactitol -specific phosphotransferase enzyme IIA component - Escherichia coli.
MTNLFVRSGISFVDRSEVLTHIGNEMLAKGVVHDTWPQALIAREAEFPTG
IMLEQHAIAIPHCEAIHAKSSAIYLLRPTNKVHFQQADDDNDVAVSLVIA
LIVENPQQQLKLLRCLFGKLQQPDIVETLITLPETQLKEYFTKYVLDSDE

> P60720|LIPB_ECOLI Lipoyltransferase - Escherichia coli.
MYQDKILVRQLGLQPYEPISQAMHEFTDTRDDSTLDEIWLVEHYPVFTQG
QAGKAEHILMPGDIPVIQSDRGGQVTYHGPGQQVMYVLLNLKRRKLGVRE
LVTLLEQTVVNTLAELGIEAHPRADAPGVYVGEKKICSLGLRIRRGCSFH
GLALNVNMDLSPFLRINPCGYAGMEMAKISQWKPEATTNNIAPRLLENIL
ALLNNPDFEYITA
> P45483|FTSZ_BORBU Cell division protein ftsZ - Borrelia burgdorferi (Lyme disease spirochete).
MKDYNMIDSHTRRFDSTTNPTILKVIGAGGGGSNAVNRMIEYGVRDVEFI
VANTDLQALQTSIAPIKIALGAKVTAGLGAGGKPEIGQAAAEEDIDVIRN
HLSGADMVFITAGMGGGTGTGAAPVIAQVAKELGILTVGVVTKPFKF EGP
KKLRLAEQGINNLRKSVDTLIIIPNQKLLTVVDKRTTIKDAFKRADDVLR
MGVQGIAGLIIEHGEVNIDFADVKSIMQGQGDALMGIGYGKGENRAVDAA
TSAISNPLLEEVRIEGSKGLLVNVTGGDDFSLLELEEIMGIITVSVDDEA
TVIYGHAINSNLEDEIYVTVVATGFASKKQKEISSTPENNTLSSKEFDTL
MSGNQNAPSGSYEQQDSSFAAKSKNVNYFDDDIDVPTFLRNLNKKSSD D
> Q01578|GNL_ZYMMO Gluconolactonase - Zymomonas mobilis.
MTTGRMSRRECLSAAVMVPIAAMTATATITGSAQAAKNNMNGSTIGKITK
FSPRLDAILDVSTPIEVIASDIQWSEGPVWVKNGNFLLFSDPPANIMRKW
TPDAGVSIFLKPSGHAEPIPAGQFREPGSNGMKVGPDGKIWVADSGTRAI
MKVDPVTRQRSVVVDNYKGKRFNSPNDLFFSKSGAVYFTDPPYG LTNLDE
SDIKEMNYNGVFRLSPDGRLDLIEAGLSRPNGLALSPDETKLYVSNSDRA
SPNIWVYSLDSNGLPTSRTLLRNFRKEYFDQGLAGLPDGMNIDKQGNLFA
SAPGGIYIFAPDGECLGLISGNPGQPLSNCCFGEKGQTLFISASHNVVRV
RTKTFG
> P25718|AMY1_ECOLI Alpha -amylase - Escherichia coli.
MKLAACFLTLLPGFAVAASWTSPGFPAFSEQGTGT FVSHAQLPKGTRPLT
LNFDQQCWQPADAIKLNQMLSLQPCSNTPPQWRLFRDGEYTLQIDTRSGT
PTLMISIQNAAEPVASLVRECPKWDGLPLTVDVSATFPEGAAVRDYYSQQ
IAIVKNGQIMLQPAATSNGLLLLERAETDTSAPFDWHNATVYFVLTDRFE
NGDPSNDQSYGRHKDGMAEIGTFHGGDLRGLTNKLDYLQQLGVNALWISA
PFEQIHGWVGGGTKGDFPHYAYHGYYTQDWTNLDAN MGNEADLRTLVDSA
HQRGIRILFDVVMNHTGYATLADMQEYQFGALYLSGDEVKKSLGERWSDW
KPAAGQTWHSFNDYINFSDKTGWDKWWGKNWIRTDIGDYDNPGFDDLTMS
LAFLPDIKTESTTASGLPVFYKNKMDTHAKAIDGYTPRDYLTHWLSQWVR
DYGIDGFRVDTAKHVELPAWQQLKTEASAALREWKKANPDKALDDKPFWM
TGEAWGHGVMQSDYYRHGFDAMINFDYQEQAAKAVDC LAQMDTTWQQMAE
KLQGFNVLSYLSSHDTRLFREGGDKAAELLLLAPGAVQIFYGDESSRPFG
PTGSDPLQGTRSDMNWQDVSGKSAASVAHWQKISQFRARHPAIGAGKQTT
LLLKQGYGFVREHGDDKVLVVWAGQQ
> P07941|PAC_KLUCI Penicillin G acylase - Kluyvera citrophila (Kluyvera cryocrescens).
MKNRNRMIVNGIVTSLICCSSLSALA ASPPTEVKIVRDEYGMPHIYADDT
YRLFYGYGYVVAQDRLFQMEMARRSTQGTVSEVLGKAFVSFDKDIRQNYW
PDSIRAQIASLSAEDKSILQGYADGMNAWIDKVNASPDKLLPQQFSTFGF
KPKHWEPFDVAMIFVGTMANRFSDSTSEIDNLALLTAVKDKYGNDEGMAV
FNQLKWLVNPSAPTTIAARESSYPLKFDLQNTQTAALLVPRYDQPAPMLD
RPAKGTDGALLAVTAIKNRETIAAQFA NGANGLAGYPTTSNMWVIGKNKA
QDAKAIMVNGPQFGWYAPAYTYGIGLHGAGYDVTGNTPFAYPGLVFGHNG
TISWGSTAGFGDDVDIFAEKLSAEKPGYYQHNGEWVKMLSRKETIAVKDG
QPETFTVWRTLDGNVIKTDTRTQTAYAKARAWAGKEVASLLAWTHQMKAK
NWPEWTQQAAKQALTINWYYADVNGNIGYVHTGAYPDRQPGHDPRLPVPD
GKWDWKGLLSFDLNPKVYNPQSGYIANW NNSPQKDYPASDLFAFLWGGAD
RVTEIDTILDKQPRFTADQAWDVIRQTSLRDLLRLFLPALKDATANLAEN
DPRRQLVDKLASWDGENLVNDDGKTYQQPGSAILNAWLTSMLKRTVVAAV

PAPFGKWYSASGYETTQDGPTGSLNISVGAKILYEALQGDKSPIPQAVDL
FGGKPEQEVILAALDDAWQTLSKRYGNDVTGWKTPAMALTFRANNFFGVP
QAAAKEARHQAEYQNRGTENDMIVFSPTS GNRPVLAWDVVAPGQSGFIAP
DGKADKHYDDQLKMYESFGRKSLWLTPQDVDEHKESQEVLQVQR
> P26648|SUFI_ECOLI Protein sufI - Escherichia coli.
MSLSRRQFIQASGIALCAGAVPLKASAAGQQQPLPVPPLLESRRGQPLFM
TVQRAHWSFTPGTRASVWGINGRYLGPTIRVWKGDDVKLIYSNRLTENVS
MTVAGLQVPGPLMGGPARMMSPNADWAPVLPIRQ NAATLWYHANTPNRTA
QQVYNGLAGMWLVEDEVSKSLPIPNHYGVDDFPVIIQDKRLDNFGTPEYN
EPGSGGFVGDTLLVNGVQSPYVEVSRGWVRLRLLNASNSRRYQLQMNDGR
PLHVISGDQGFLPAPVSVKQLSLAPGERREILVDMSNGDEVSITCGEAAS
IVDRIRGFFEPSSILVSTLVLTLRPTGLLPLVTDSLPMRLLPTEIMAGSP
IRSRDISLGDDPGINGQLWDVNRIDVTAQQGTWER WTVRADEPQAFHIEG
VMFQIRNVNGAMPFPEDRGWKDTVWVDGQVELLVYFGQPSWAHFPFYFNS
QTLEMADRGSIGQLLVNPVP
> P0ADV1|YHBN_ECOLI Protein yhbN - Escherichia coli.
MKFKTNKLSLNLVLASSLLAASIPAFAVTGDTDQPIHIESDQQSLDMQGN
VVTFTGNVIVTQGTIKINADKVVVTRPGGEQGKEVIDGYGKPATFYQMQD
NGKPVEGHASQMH YELAKDFVVLTGNAYLQQVDSNIKGDKITYLVKEQKM
QAFSDKGKRVTTVLVPSQLQDKNNKGQTPAQKKGN
> P33770|HEMF_RHOS4 Coproporphyrinogen III oxidase, anaerobic 1 - Rhodobacter sphaeroides (strain ATCC 17023 / 2.4.1 / NCIB 8253 / DSM 158).
MTNIALLQSLGLFDARVPRYTSYPAAPVFSGAVGADFQAQA IEALDPAVP
ISVYVHVPFCERLCWFCACRTQGTQTLAPVEAYVGTLLQELELVKQHLPA
GVKAGRLHWGGGTPTILSPELIHKLAQAIKAVIPFAEDYEFSVEIDPMMV
DEPKIRALSEEGMNRASIGIQDFTDIVQNAIGREQPFENTKACVETLRRY
GVHSLNTDLVYGLPHQNRESLAATIDKVLSLRPDRVAIFGYAHVPWMAKR
QKLIDETVLPPDIERHELANLAARLFTEGGFERIGIDHFALP DDSMAVAA
RSRKLRRNFQGYTDDTCPTLLGIGASSISKFEQGYLQNTAATAAYIKSIE
EGRLPGYRGHRMTEEDYLHGRAIEMIMCDFFLDLPALRARFGEPAETMVP
RIAEAAEKFTPFVTVDADGSMSIAKEGRALARMIARLFDAYETPEARYSQ
AS
> P14489|BLP2_PSEAE Beta -lactamase PSE -2 - Pseudomonas aeruginosa.
MKTFAAYVIIACLSSTALAGSITE NTSWNKEFSAEAVNGVFVLCKSSSKS
CATNDLARASKEYLPASTFKIPNAIIGLETGVIKNEHQVFKWDGKPRAMK
QWERDLTLRGAIQVSAVPVFQQIAREVGEVRMQKYLKKFSYGNQNISGGI
DKFWLEGQLRISAVNQVEFLESLYLNKLSASKENQLIVKEALVTEAAPEY
LVHSKTGFSGVGTESNPGVAWWVGWVEKETEVYFFAFNMDIDNESKLPLR
KSIPTKIMESEGIIGG
> P0A1N4|FLHE_SALTY Flagellar protein flhE - Salmonella typhimurium.
MRKWLALLLFPLTVQAAGEGAWQDSGMGVTLNYRGVSASSSPLSARQPVS
GVMTLVAWRYELNGPTPAGLRVRLCSQSRCVELDGQSGTTHGFAHVPAVE
PLRFVWEVPGGGRLIPALKVRSNQVIVNYR
> P06202|OPPA_SALTY Periplasmic oligopeptide -binding protein - Salmonella typhimurium.
MSNITKKSLIAAGILTALIAASAATAADVPAGVQLADKQTLVRNNGSEVQ
SLDPHKIEGVPESNVSRDLFEGLLISDVEGHPSPGVAEKWENKDFKVWTF
HLRENAKWSDGTPVTAHDFVYSWQRLADPNTASPYASYLQYGHIANIDDI
IAGKKPATDLGVKALDDHTFEVTLSEPVPYFYKLLVHPSVSPVPKSAVEK
FGDKWTQPANIVTNGAYKLKNWVVNER IVLERNPQYWDNAKTVINQVTYL
PISSEVTDVNRYRSGEIDMTYNNMPIELFQKLKKEIPNEVRVDPYLCTYY
YEINNQKAPFNDVRVRTALKLALDRDIIVNKVKNQGDLPAYSYTPPYTDG
AKLVEPEWFKWSQQKRNEEAKKLLAEAGFTADKPLTFDLLYNTSDLHKKL
AIAVASIWKKNLGVNVNLENQEWKTFLDTRHQGTFDVARAGWCADYNEPT
SFLNTMLSDSSNNTAHYKSPAFDKLIAD TLKVADDTQRSELYAKAEQQLD
KDSAIVPVYYYVNARLVKPWVGGYTGKDPLDNIYVKNLYIIKH
> MOXR_PARDE P29901 MOXR PROTEIN. - PARACOCCUS DENITRIFICANS.
MDSNISDWHARFRDAEAALNGVVLGQARTIRLLLISALCRGHVLLAGDVG
TGKTTLLRAMARALGGPYGRVEGTVDLLPTDLIYSTHIAEDGRPRIEPGP
VLEQGEDMAVFFFNEINRARPQVHA LLLRLMAERSLSAFRREYRFPHLQV
FADRNQIERDETFELPAAARDRFLMEIAVDAPAAPEDRVALAFEPRFHDT
TALIAEVGQGILPYRGLNGLAAGVQSGTHASPALRRYVFDLCEALRNPAS
AGLALEGADAARLIRGGVSPRGMQHLVRAARACAWLEGREAVLPQDVRAV

LAPVMAHRIFLSPAYEPRREALVPALIDAAFATIPVPAA
> Q09064|UREE_HELPY Urease accessory protein ureE - Helicobacter pylori (Campylobacter pylori).
MIIERLVGNLRDLNPLDFSVDHVDLEWFETRKKIARFKTRQGKDIAIRLK
DAPKLGLSQGDILFKEEKEIIAVNILDSEVIHIQAKSVAEVAKICYEIGN
RHAALYYGESQFEFKTPFEKPTLALLEKLGVQNRVLSSKLDSKERLTVSM
PHSEPNFKVSLASDFKVVVK
> P29930|COBO_PSEDE Cob(I)yrinic acid a,c-diamide adenosyltransferase - Pseudomonas denitrificans.
MSDETTVGGEAPAEKDDARHAMKMAKKKAAREKIMATKTDEKGLIIVNTG
KGKGKSTAGFGMIFRHIAHGMPCAVVQFIKGAMATGERELIEKHFGDVCQ
FYTLGEGFTWETQDRARDVAMAEKAWEKAKELIRDERNSMVLLDEINIAL
RYDYIDVAEVVRFLKEEKPHMTHVVLT GRNAKEDLIEVADLVTEMELIKH
PFRSGIKAQQGVEF
> P10332|MOMPA_CHLPS Major outer membrane porin - Chlamydia psittaci (Chlamydophila psittaci).
MKKLLKSALLFAATGSALSLQALPVGNPAEPSLLIDGTMWEGASGDPCDP
CATWCDAISIRAGYYGDYVFDRVLKVDVNKTFSGMAATPTQATGNASNTN
QPEANGRPNIAYGRHMQDAE WFSNAAFLALNIWDRFDIFCTLGASNGYFK
SSSAAFNLVGLIGFSATSSTSTELPMQLPNVGITQGVVEFYTDTSFSWSV
GARGALWECGCATLGAEFQYAQSNPKIEVLNVTSSPAQFVIHKPRGYKGA
SSNFPLPITAGTTEATDTKSATIKYHEWQVGLALSYRLNMLVPYIGVNWS
RATFDADTIRIAQPKLKSEILNITTWNPSLLGSTTTLPNNGGKDVLSDVL
QIASIQINKMKSRKACGVAVG ATLIDADKWSITGEARLINERAAHMNAQF
RF
> P0AEB2|DACA_ECOLI Penicillin-binding protein 5 - Escherichia coli.
MNTIFSARIMKRLALTTALCTAFISAAHADDLNIKTMIPGVPQIDAESYI
LIDYNSGKVLAEQNADVRRDPASLTKMMTSYVIGQAMKAGKFKETDLVTI
GNDAWATGNPVFKGSSLMFLKPGMQVPVSQLIRGINLQSGNDACVAMADF
AAGSQDAFVGLMNSYVNALGLKNTHFQTVHGLDADGQYSSARDMALIGQA
LIRDVPNEYSIYKEKEFTFNGIRQLNRNGLLWDNSLNVDGIKTGHTDKAG
YNLVASATEGQMRLISAVMGGRTFKGREAESKKLLTWGFRFFETVNPLKV
GKEFASEPVWFGDSDRASLGVDKDVYLTIPRGRMKDLKASYVLNSSELHA
PLQKNQVVGTINFQLDGKTIEQRPLVVLQEIPEGNFFGKIIDYIKLMFHH
WFG
> P29725|BMP_TREPA Basic membrane protein - Treponema pallidum.
MGRYIVPALLCVAGMGFAHAQSALQPIAEVNLFRREPVTLGQIKARISAI
EKEMGKKLSTAERRQFMDSLIDEKLFAQAAEKAGIQVTDAEVNQYFNGML
SQQIGRAVTEAEFANYVKEKQNISLDQFMKQQNGMTMAEYKKFLKTQVST
QRYVAQKKADEFRNLKGPEDSQIRSYYELNKQAFFRP DTVKLFLISVPKG
SGPAAAKAKAQEFVKKLKSSGVKATADIKSKANGAQAGYSAGEIYLGKTA
VTATQLGLTMEALLEIFGMGVGAVSDVNETANDYQCFIVLKKEEAKILTL
SDLVEPDKTVSLYEFIKNLLTSQIQQKALEDAIREVSAELRKSGTYKVFL
ADAELGKVLDW
> P0ABE7|C562_ECOLI Soluble cytochrome b562 - Escherichia coli.
MRKSLLAILAVSSLVFSSASFAADLEDNMETLNDNLKVIEKADNAAQVKD
ALTKMRAAALDAQKATPPKLEDKSPDSPEMKDFRHGFDILVGQIDDALKL
ANEGKVKEAQAAAEQLKTTRNAYHQKYR
> P19247|VVHA_VIBVU Cytolysin - Vibrio vulnificus.
MKKMTLFTLSLLATAVQVGAQEYVPIVEKPIYITSSKIKCVLHTSGDFNA
TRDWCNAGASIDVRVNVAQMRSVQSATSDGFTPDAK IVRFTVDADKPGTG
IHLVNELQQDHSWFQSWANRRTYIGPFASSYDLWVKPVSGYTPKKARDLP
QNENKNYQHRDTYGYSIGINGKVGAEVNKDGPKVGGEVSGSFTYNYSKTL
VFDTKDYRINNRSSLSDFDISFEREFGECDELRRQELGCYFTAAHWGSGW
VFDKTKFNPISYSNFKPNYDVLYEAPVSETGVTDFEMGVKLNYRARFGTV
LPSALFSVYGSAGSSTNSSTVKQRIRIDWNHPLFEAE AHVTLQSLSNNDL
CLDVYGENGDKTVAGGSVNGWSCHGSWNQVWGLDKEERYRSRVASDRCLT
VNADKTLTVEQCGANLAQKWYWEGDKLISRYVDGNNTRYLLNIVGGRNVQ
VTPENEANQARWKPTLQQVKL
> Q47898|ASPG_ELIMR N(4)-(Beta-N-acetylglucosaminyl)-L-asparaginase - Elizabethkingia miricola (Chryseobacterium miric ola).
MRIIYKQQTMNNNRRDFIKKLGIATAAIAINPLEAKNLLDTSEPKTTNKP
IVLSTWNFGLHANVEAWKVLSKGGKALDAVEKGVRLVEDDPTERSVGYGG

RPDRDGRVTLDACIMDENYNIGSVACMEHIKNPISVARAVMEKTPHVMLV
GDGALEFALSQGFKKENLLTAESEKEWKEWLKTSQYKPIVNIENHDTIGM
IALDAQGNLSGACTTSGMAYKMHGRVGDSPIIGAGLFVDNEIGAAT ATGH
GEEVIRTVGTHLVVELMNQGRTPQQACKEAVERIVKIVNRRGKNLKDIQV
GFIALNKKGEYGAYCIQDGFNFAVHDQKGNRLETPGFALK
> P41258|SYK_CAMJE Lysyl -tRNA synthetase - Campylobacter jejuni.
MFDNILEQQRIEKAKELKNLGINPYPHFLEKEMSLKTFKDKFSYILEQVE
KRDESVNAVVAGRLKLLRIAGKSIFANIEDEDTNLQIYFSKDS VGEELYT
ILKKNLEVGDIVLVKGFPFVTKTGEFSLHASEVKLATKAIVPLPEKYHGL
TDIEQRYRKRYVDMIMNVEVRKDFLVRSKVVSLIRHFFENKGFLEVETPM
MHPIAGGANAKPFVTFHNSLGVERFLRIAPELYLKRLIVGGFEAVFEINR
CFRNEGMDLTHNPEFTTIEFYWAYHNYKDLMDLTEELFALLLDKLNLGKT
IEFDGKMINFSKPFERITYKDALCKYGGLDRDLIEDKEKILTKL KADGFE
ANEKLELGHLQAELFDNYVEEKLINPTFVIDFPISISPLSRRSDEDSQIA
ERFELFICGRELANGFNELNDPLDQYERFLKQIEAKNAGDEEACEMDEDF
VNALGYGMPPTAGQGIGIDRLVMLLTNKKSIRDVILFPAMRPLKSELKEK
E
> P43886|CYSE_HAEIN Serine acetyltransferase - Haemophilus influenzae.
MTLDVWQHIRQEAKELAENEPML ASFFHSTILKHQNLGGALSYLLANKLA
NPIMPAISLREIIEEAYQSNPSIIDCAACDIQAVRHRDPAVELWSTPLLY
LKGFHAIQSYRITHYLWNQNRKSLALYLQNQISVAFDVDIHPAAKIGHGI
MFDHATGIVVGETSVIENDVSILQGVTLGGTGKESGDRHPKVREGVMIGA
GAKILGNIEVGKYAKIGANSVVLNPVPEYATAAGVPARIVSQDKAAKPAF
DMNQYFIGIDDGMNLNI
> P00811|AMPC_ECOLI Beta -lactamase - Escherichia coli.
MFKTTLCALLITASCSTFAAPQQINDIVHRTITPLIEQQKIPGMAVAVIY
QGKPYYFTWGYADIAKKQPVTQQTLFELGSVSKTFTGVLGGDAIARGEIK
LSDPTTKYWPELTAKQWNGITLLHLATYTAGGLPLQVPDEVKSSSDLLRF
YQNWQPAWAPGTQRLYANSSIGLFGALAVKPSGLSFEQAMQTRVFQPLKL
NHTWINVPPAEEKNYAWGYREGKAVHVSPGALDAEAYGVKSTIEDMARWV
QSNLKPLDINEKTLQQGIQLAQSRYWQTGDMYQGLGWEMLDWPVNPDSII
NGSDNKIALAARPVKAITPPTPAVRASWVHKTGATGGFGSYVAFIPEKEL
GIVMLANKNYPNPARVDAAWQILNALQ
> P22751|PELX_ERWCH Pectate disaccharide -lyase - Erwinia chrysanthemi.
MKYAASGLLSVALNSLLLLGSNQRFATQDVAPVWRGIAFGQSTDVNFATN
VLPEKVGVNDVTINGKKLTVNDKADLSAPITIESRGGKIANTHDGLTFFY
TQLPANVNFTLQSDVTVEQFGPESDAKPNAQEGAGLLVRDILGVPRQEPL
KEGYEEFPAASNMVMNAIMTQDKKSKTEVKMQLISRNGVTQPWGNTNAEI
TRTSYQEKINLEQTPTFRLKLERTNDGFITAYAPKGSDQWVSKTVKGADL
VTHQDKDHYYVGFFASRNAKITISNASLTTSPANTKPSAPFKAETTAPLL
QVASSSLSTSDTYPVQARVNYNGTVEVFQNGKSLGKPQRVRAGDDFSLTT
RLTQQKSDFKLVYIPSEGEDKTAKETSFSVEKITLADARNLYVSPEGKAG
NDGSKNAPLDIKTAINALPGGGTLWLMDGDYSATVIPVSATQRKGMKTLM
PVGKKAVFHGLQLNASYWKVKGIEITEKSFRIEGSHNQIERLLAHHCDNT
GIQVSSSDNVGRPLWASHNLILNSESHSNQHPSKKDADGFAVKMRVGEGN
VIRGAFSHDNVDDGFDLFNKIEDGPNGAVMIENSISLNNTSNGFKLGGEG
QPVAHQVKNSIAIGNHMDGFSDNFNPGALQVSNNIALDNVRFNFIFRPSP
YYGYEKQGIFKNNVSLRTQPGKYDDAVVGRLDASNYFIRIIERSTVRVRK
SRRRITNPSRCQRSSAGMKKAACNWVIFCRRSNRHKTQRHRNRYPSTPA
> DHSS_SYNP1 P14776 SOLUBLE HYDROGENASE, SMALL SUBUNIT (EC 1.
MQDKAMLMIPGPTPVPESVLLSLGKHPIGHRSGEFSQIMAAMTAGIKWLH
QTQNEVLILAASGTGAMEAGIINFLSAGDRVVVGCNGKFGDRWGEVCDAY
GLTTERISAPWGQPLNPDDFKAVLDGHRQKPSKAVIVTHSETSTGVINDL
EAINRHVKAHGQALIIVDAVTSLGAVSVPIDEWGLDVVGSGSQKGYMIPP
GLAFVSVSPKAWEAYKTATLPKFYLDLGKYRKDAAKHTTPFTPPVNLFFA
LKTALEMMQAEGLEAIFQRHQRLMQATRAAMKALNLPLYAADSCASPAIT
AVAPQGVEAENIRSLMKKRFDIALAGGQDHLKGQIFRIGHLGFVGDRDIL
AAVSALEAVLAELGYTNFTPGAGVAAASRVLSTA
> P06960|OTC2_ECOLI Ornithine carbamoyltransferase chain F - Escherichia coli.
MSDLYKKHFLKLLDFTPAQFTSLLTLAAQLKADKKNGKEVQKLTGKNIAL
IFEKDSTRTRCSFEVAAFDQGARVTYLGPSGSQIGHKESIKDTARVLGRM
YDGIQYRGHGQEVVETLAQYAGVPVWNGLTNEFHPTQLLADLMTMQEHLP

GKAFNEMTLVYAGDARNNMGNSMLEAAALTGLDLRLLAPKACWPEESLVA
ECSALAEKHGGKITLTEDVAAGVKGADFIYTDVWVSMGEAKE KWAERIAL
LRGYQVNAQMMALTDNPNVKFLHCLPAFHDDQTTLGKQMAKEFDLHGGME
VTDEVFESAASIVFDQAENRMHTIKAVMMATLGE
> P0A8N3|SYK1_ECOLI Lysyl -tRNA synthetase - Escherichia coli.
MSEQHAQGADAVVDLNNELKTRREKLANLREQGIAFPNDFRRDHTSDQLH
AEFDGKENEELEALNIEVAVAGRMMTRRIMGKASFVTLQDVGGRIQLY VA
RDDLPEGVYNEQFKKWDLGDILGAKGKLFKTKTGELSIHCTELRLLTKAL
RPLPDKFHGLQDQEARYRQRYLDLISNDESRNTFKVRSQILSGIRQFMVN
RGFMEVETPMMQVIPGGAAARPFITHHNALDLDMYLRIAPELYLKRLVVG
GFERVFEINRNFRNEGISVRHNPEFTMMELYMAYADYKDLIELTESLFRT
LAQDILGKTEVTYGDVTLDFGKPFEKLTMREAIKKYRPETDMADLDNFD S
AKAIAESIGIHVEKSWGLGRIVTEIFEEVAEAHLIQPTFITEYPAEVSPL
ARRNDVNPEITDRFEFFIGGREIGNGFSELNDAEDQAQRFLDQVAAKDAG
DDEAMFYDEDYVTALEHGLPPTAGLGIGIDRMVMLFTNSHTIRDVILFPA
MRPVK
> P21163|PNGF_ELIMR Peptide -N(4)-(N-acetyl-beta-D-glucosaminyl)asparagine amidase F - Elizabethkingia miricola (Chryseobacterium miricola).
MRKLLIFSISAYLMAGIVSCKGVDSATPVTEDRLALNAVNAPADNTVNIK
TFDKVKNAFGDGLSQSAEGTFTFPADVTTVKTIKMFIKNECPNKTCDEWD
RYANVYVKNKTTGEWYEIGRFITPYWVGTEKLPRGLEIDVTDFKSLLSGN
TELKIYTETWLAKGREYSVDFDIVYGTPDYKYSAVVPVIQYNKSSIDGVP
YGKAHTLGLKKNIQLPTNTEKAYLRTTISGWGHAKPYDAGSRGCAEWCFR
THTIAINNANTFQHQLGALGCSANPINNQSPGNWAPDRAGWCPGMAVPTR
IDVLNNSLTGSTFSYEYKFQSWTNNGTNGDAFYAISSFVIAKSNTPISAP
VVTN
> P0AFK0|PMBA_ECOLI Protein pmbA - Escherichia coli.
MALAMKVISQVEAQRKILEEAVSTALELASGKSDGAEVAVSKTTGISVST
RYGEVENVEFNSDGALGITVYHQNRKGSASSTDLSPQAIARTVQAALDIA
RYTSPDPCAGVADKELLAFDAPDLDLFHPAEVSPDEAIELAARAEQAALQ
ADKRITNTEGGSFNSHYGVKVFGNSHGMLQGYCSTRHSLSSCVIAEENGD
MERDYAYTIGRAMSDLQTPEWVGADCARRTLSRLSPRKLSTMKAPVIFAN
EVATGLFGHLVGAIAGGSVYRKSTFLLDSLGKQILPDWLTIEEHPHLLKG
LASTPFDSEGVRTERRDIIKDGILTQWLLTSYSARKLGLKSTGHAGGIHN
WRIAGQGLSFEQMLKEMGTGLVVTELMGQGVSAITGDYSRGAAGFWVENG
EIQYPVSEITIAGNLKDMWRNIVTVGNDIETRSNIQCGSVLLPEMKIAGQ

> P29822|LACE_AGRRD Lactose -binding protein - Agrobacterium radiobacter.
MDYSRLLKRSVSAALTAAALLCSTAAFAGEV TIWCWDPNFNVAIMKEAAE
RYTAKHPDTTFNIVDFAKADVEQKLQTGLASGMTDTLPDIVLIEDYGAQK
YLQSFPGSFAALTDKIDFSGFAKYKVDLMTLEGQVYGVPFDSGVTGLYYR
TDYLEQAGFKPEDMQNLTWDRFIEIGKEVKAKTGHEMMALDANDGGLIRI
MMQSGGQWYFNEDGSLNITGNAALKAALETQARIVNERVAKPTSGSNDGI
RALTSGDVASVLRGVWITGTVKSQPDQAGKWA LTAIPKLNIEGATAASNL
GGSSWYVLEASAEKDEAIDFLNEIYAKDLDFYQKILTERGAVGSLLAART
GEAYQKPDDFFGGQTVWQNFADWLVQVPAVNYGIFTNELDTAVTANFPAL
VKGTPVDEVLKAIEDQAAGQIQ
> P56194|SYH_THET8 Histidyl -tRNA synthetase - Thermus thermophilus (strain HB8 / ATCC 27634 / DSM 579).
MTARAVRGTKDLFGKELRMHQRIVATARKVLEAAGALELVTPIFEETQVF
EKGVGAATDIVRKEMFTFQDRGGRSLTLRPEGTAAMVRAYLEHGMKVWPQ
PVRLWMAGPMFRAERPQKGRYRQFHQVNYEALGSENPILDAEAVVLLYEC
LKELGLRRLKVKLSSVGDPEDRARYNAYLREVLSPHREALSEDSKERLEL
NPMRILDSKSERDQALLKELGVRPMLDFLGEEARAHLKEVERHLERLSVP
YELEPALVRGLDYYVRTAFEVHHEEIGAQSALGGGGRYDGLSELLGGPRV
PGVGFAFGVERVALALEAEGFGLPEEKGPDLYLIPLTEEAVAEAFYLAEA
LRPRLRAEYALAPRKPAKGLEEALKRGAAFAGFLGEDELRAGEVTLKRLA
TGEQVRLSREEVPGYLLQALG
> P44584|PNP_HAEIN Polyribonucleotide nucleotidyltransferase - Haemophilus influenzae.
MNPIVKQFKYGQHTVTLETGAIARQATAAVMASMDDTTVFVTVVAKKDVK
EGQDFFPLTVNYQERTYAAGKIPGGFFKREGRPSEGETLIARLIDRPIRP
LFPEGFFNEIQVVATVVSVNPQISPDLVAMIGASAALTLSGVPFNGPIGA

ARVGFIDNQFVLNPTMAEQKQSRLDLVVAGTDKAVLMVESEADILTEEQM
LAAVVFGHQQQQVVVEAIKEFAKEAGKPRWDWVAPQPNTDLINKVKAIAE
ARLGDAYRITEKQLRYEQIDAIKADVIAQITAEDEEXSEGKIVDIFTALE
SQIVRGRIIAGEPRIDGRTVDTVRALDICTGVLPRTHGSAIFTRGETQAL
AVATLGTERDAQIIDELTGERQDHFLFHYNFPPYSVGETGMIGSPKRREI
GHGRLAKRGVAAVMPSLAEFPYVVRVVSEITESNGSSSMASVCGASLALM
DAGVPIKAAVAGIAMGLVKEEEKFVVLSDILGDEDHLGDMDFKVAGTREG
VTALQMDIKIEGITPEIMQIALNQAKSARMHILGVMEQAIPAPRADISDY
APRIYTMKIDPKKIKDVIGKGGATIRSLTEETGTSIDIDDDGTVKIAAVD
SNAAKNVMGRIEEIVAEVEAGVIYKGKVTRLADFGAFVAIVGNKEGLVHI
SQIAEERVEKVSDYLQVGQEVNVKVVEIDRQGRIRLTMKDLAPKQETEIN
QEDSVEEQE
> P74132|HEMN_SYNY3 Oxygen -independent copropor phyrinogen III oxidase -
Synechocystis sp. (strain PCC 6803).
MTTTFPTVEFSAELLNKYNQGIPRYTSYPPATELNKEFDPSDFQTAINLG
NYKKTPLSLYCHIPFCAKACYFCGCNTIITQHKPAVDPYLKAVAKQIALV
APLVDQQRPVQQLHWGGGTPNYLTLEQAEFLFNTITDAFPLAENAEISIE
INPCYVDKDYIFALRQLGFNRISFGIQDFNSQVQQAVNRIQ PEAMLFQVM
DWIRQANFDSVNVDLIYGLPHQNLATFRETLRKTAQLNPDRIAVFNFAYV
PWLKPVQKKMPESALPPAEEKLKIMQATIADLTEQGYVFIGMDHFAKPDD
ELAIAQRRGELHRNFQGYTTQPESDLLGFGITSISMLQDVYAQNHKTLKA
FYNALDREVMPIEKGFKLSQDDLIRRTVIKELMCQFKLSAQELESKYNLG
FDCDFNDYFAKELSALDVLEADGLLRRLGDGLEVTPRGRILI RNIAAVFD
TYLQNKSKQQMFSRAI
> P07102|PPA_ECOLI Periplasmic appA protein [Includes: Phosphoanhydride
phosphohydrolase - Escherichia coli.
MKAILIPFLSLLIPLTPQSAFAQSEPELKLESVVIVSRHGVRAPTKATQL
MQDVTPDAWPTWPVKLGWLTPRGGELIAYLGHYQRQRLVADGLLAKKGCP
QSGQVAIIADVDERTRKTG EAFAAGLAPDCAITVHTQADTSSPDPLFNPL
KTGVCQLDNANVTDAILSRAGGSIADFTGHRQTAFRELERVLNFPQSNLC
LKREKQDESCSLTQALPSELKVSADNVSLTGAVSLASMLTEIFLLQQAQG
MPEPGWGRITDSHQWNTLLSLHNAQFYLLQRTPEVARSRATPLLDLIKTA
LTPHPPQKQAYGVTLPTSVLFIAGHDTNLANLGGALELNWTLPGQPDNTP
PGGELVFERWRRLSDNSQWI QVSLVFQTLQQMRDKTPLSLNTPPGEVKLT
LAGCEERNAQGMCSLAGFTQIVNEARIPACSL
> P69795|PTQB_ECOLI N,N' -diacetylchitobiose -specific phosphotransferase
enzyme IIB component - Escherichia coli.
MEKKHIYLFCSAGMSTSLLVSKMRAQAEKYEVPVIIEAFPETLAGEKGQN
ADVVLLGPQIAYMLPEIQRLLPNKPVEV IDSLLYGKVDGLGVLKAAVAAI
KKAAAN
> Q3J164|CYC2_RHOS4 Cytochrome c2 - Rhodobacter sphaeroides (strain ATCC
17023 / 2.4.1 / NCIB 8253 / DSM 158).
MKFQVKALAAIAAFAALPALAQEGDPEAGAKAFNQCQTCHVIVDDSGTTI
AGRNAKTGPNLYGVVGRTAGTQADFKGYGEGMKEAGAKGLAWDEEHFVQY
VQDPTKFLKEYTG DAKAKGKMTFKLKKEADAHNIWAYLQQVAVRP
> NADA_SALTY P24519 QUINOLINATE SYNTHETASE A. - SALMONELLA TY
MSVMFDPQAAIYPFPPKPTPLNDDEKQFYREKIKRLLKERNAVMVAHYYT
DPEIQQLAEETGGCISDSLEMARFGTKHAASTLLVAGVRFMGETAKILSP
EKTILMPTLAAECSLDLGCPIDEFSAFCDAHPDRTVVVYANTSAAVKARA
DWVVTSSIAVELIEHLDSLGEKIIWAPDRHLGNYVQKQTGADVLCWQGAC
IVHDEFKTQALTRLKKIYPDAALLVHPESPQSIVEMADAVGSTSQLIKAA
KTLPHRQLIVATDRGIFYKMQQAVPEKELLEAPTAGEGATCRSCAHCPWM
AMNGLKAIAEGLEQGGARMKYRLMRRYARAHYCRSTGCWILRLHFGRKDG
NLWGREWIFLVRTTY
> NODA_BRAEL P50326 NODULATION PROTEIN A (EC 2.3.1.-). - BRAD
MNIAVSPSAEEPSARTQVQWSLRWESELQLADHAELADFFRKSYGPTGAF
NAQPFEGIRSWAGARPEMRVIGYDAHGVAAHIGLLRRFIKIGGVDLLVAE
LGLYAVRPDLEGLGISHSMRVMYPALQELGVPFGFGTVRPALEKHLTRLV
GRRGLATLMPGIRVRSTQADVYPNLSPIRIEDVLVVVFPVGRSMGEWPAG
TIIDRNGPEL
> P68588|PSAE_YERPE Pro tein psaE - Yersinia pestis.
MSHCVVLNKLESVLIIGDSRYALSKNEVLLLECLYLRAGDVISHDELLTT
CWPDRVVSPTSLPVAIKHIRDVFRKITRSEVIKTYKNEGYSYQKDSVLII

IDDGSTEKESHSAAYTRKEKPDIPIKLVGLQILSHLNSTFFIAIMMVIII
IFFMVGGNDIVSFIDSDTNSVIITNVTTKMNGPTAGLPKVKNSMIFKDDF
GLVIICDQSECKQQ
> CH10_ECOLI P05380 10 KD CHAPERONIN (PROTEIN CPN10) (PROTEIN
MNIRPLHDRVIVKRKEVETKSAGGIVLTGSAAAKSTRGEVLAVGNGRILE
NGEVKPLDVKVGDIVIFNDGYGVKSEKIDNEEVLIMSESDILAIVEA
> P33136|OPGG_ECOLI Glucans biosynthesis protein G - Escherichia coli.
MMKMRWLSAAVMLTLYTSSSWAFSIDDVAKQA QSLAGKGYETPKSNLPSV
FRDMKYADYQQIQFNHDKAYWNNLKTPFKLEFYHQGMYFDTPVKINEVTA
TAVKRIKYSPDYFTFGDVQHDKDTVKDLGFAGFKVLYPINSKDKNDEIVS
MLGASYFRVIGAGQVYGLSARGLAIDTALPSGEEFPRFKEFWIERPKPTD
KRLTIYALLDSPRATGAYKFVVMPGRDTVVDVQSKIYLRDKVGKLGVAPL
TSMFLFGPNQPSPANNYRPELHDSNGLSIHAGN GEWIWRPLNNPKHLAVS
SFSMENPQGFGLLQRGRDFSRFEDLDDRYDLRPSAWVTPKGEWGKGSVEL
VEIPTNDETNDNIVAYWTPDQLPEPGKEMNFKYTITFSRDEDKLHAPDNA
WVQQTRRSTGDVKQSNLIRQPDGTIAFVVDFTGAEMKKLPEDTPVTAQTS
IGDNGEIVESTVRYNPVTKGWRLVMRVKVKDAKKTTEMRAALVNADQTLS
ETWSYQLPANE
> P0A9P0|DLDH_ECOLI Di hydrolipoyl dehydrogenase - Escherichia coli.
MSTEIKTQVVVLGAGPAGYSAAFRCADLGLETVIVERYNTLGGVCLNVGC
IPSKALLHVAKVIEEAKALAEHGIVFGEPKTDIDKIRTWKEKVINQLTGG
LAGMAKGRKVKVVNGLGKFTGANTLEVEGENGKTVINFDNAIIAAGSRPI
QLPFIPHEDPRIWDSTDALELKEVPERLLVMGGGIIGLEMGTVYHALGSQ
IDVVEMFDQVIPAADKDIVKVFTKRISKKFNLMLETKVTAVEAKEDGIYV
TMEGKKAPAEPQRYDAVLVAIGRVPNGKNLDAGKAGVEVDDRGFIRVDKQ
LRTNVPHIFAIGDIVGQPMLAHKGVHEGHVAAEVIAGKKHYFDPKVIPSI
AYTEPEVAWVGLTEKEAKEKGISYETATFPWAASGRAIASDCADGMTKLI
FDKESHRVIGGAIVGTNGGELLGEIGLAIEMGCDAEDIALTIHAHPTLHE
SVGLAAEVFEGSITDLPNPKAKKK
> P31075|PSRA_WOLSU Polysulfide reductase chain A - Wolinella succinogenes.
METTMTRRDFLKSAGAAGAAGLVWSQTIPGTLGALEKQEIKGSAKFVPSI
CEMCTSSCTIEARVEGDKGVFIRGNPKDKSRGGKVCARGGSGFNQLYDPQ
RLVKPIMRVGERGEGKWKEVSWDEAYTFIAKKLDEIKQKHGAHTVAFTAR
SGWNKTWFHHLAQAYGSPNIFGHESTCPLAYNMAGRDVFGGSMNRDFAKA
KYIINMGHNVFEGIVISYVRQYMEAIENGAKVVTLEPRLSVMAQKASEWH
AIKPGHDLPFVLGFMHTLIFENLYDKKFVQKYCTGFEELKASIEPCTPEK
MALECDIPADTIKRLAREFAKAAPKAIFDFGHRVTFTPQELELRRAMMMV
NALVGNIERDGGMYFGKNASFYNQFLGEEDPKAKGLKKPKTPAYPKVEVP
RIDRIGEKDGEFFLANKGEGIVSLVPKATLNELPGVPCKIHGWFIVRNNP
VMTQTNADTVIKALKSMDLVVCVDIQVSDTAWFADVVLPDTTYLERDEEF
TAGGGKNPSFGIGRQKVVEPLGDAKPGWKIAKELSEKMGLGEYFPWKDIE
DYRLQQVDGDLDLLAKLKKDGSASFGVPLMLQEKKSVAEFVKKFPGAASK
VNEEGLIDFPKKIQLFSPKLEEVSGKGGLGYEPFKYKEEDELYFVQGKTP
VRSNSHTGNVPWLNNLMEYDAIWIHPKTASKLGIKNGDAIELYNKFSSQK
SKALITEGVREDTLFGYFGFGHVSKDLKRAYGKGVNSNALMPSFTSPNSG
MDLHVFGVKVKKA
> P0AFK9|POTD_ECOLI Spermidine/putrescine -binding periplasmic protein - Escherichia coli.
MKKWSRHLLAAGALALGMSAAHADDNNTLYFYNWTEYVPPGLLEQFTKET
GIKVIYSTYESNETMYAKLKTYKDGAYDLVVPSTYYVDKMRKEGMIQKID
KSKLTNFSNLDPDMLNKPFDPNNDYSIPYIWGATAIGVNGDAVDPKSVTS
WADLWKPEYKGSLLLTDDAREVFQMALRKLGYSGNTTDPKEIEAAYNELK
KLMPNVAAFNSDNPANPYMEGEVNLGMIWNGSAFVARQAGTPIDVVWPKE
GGIFWMDSLAIPANAKNKEGALKLINFLLRPDVAKQVAETIGYPTPNLAA
RKLLSPEVANDKTLYPDAETIKNGEWQNDVGAASSIYEEYYQKLKAGR
> P44323|IF2_HAEIN Translation initiation factor IF -2 - Haemophilus influenzae.
MTEDVKADAPKKLSIQRRTKTTVSSTTTGGKSKSEVQVEVRKKRTVKTDIA
QQEEAKLKAQQEAEAKKIAEQKAAEEKARLEAEKAKAETAKPVKSAVDSK
AKSVESEKEKRKAGEAELRRKAEELARQKAEEQARR AVEEAKRYAEADDS
DNESSSEDYSDYNLSSRYALEAEDEEDRRNENRGRGKNKVAKAKKGGRDD
ENSKNSKNERESNRKNQKDAKFGKGKNGKKGAALQQAFTKPAQVVKSDVV
IGETITVAELANKMAVKATEIIKMMMKMGEMVTINQVIDQETAQLVAEEL

GHKVILRNENELEEAVLGDRDVNAEKVTRAPVVTIMGHVDHGKTSLLDYI
RKAKVAAGEAGGITQHIGAYHVEMDDGKMITFLDTPG HAAFTSMRARGAK
ATDIVVLVVAADDGVMPQTIEAIQHAKAAGAPLVVAVNKIDKPEANLDRV
EQELLQHDVISEKFGGDVQFVPVSAKKGTGVDDLLDAILLQSEVLELTAV
KDGMASGVVIESYLDKGRGPVATILVQSGTLRKGDIVLCGFEYGRVRAMR
DENGKEVDEAGPSIPVELLGLSGVPAAGDEATVVRDEKKAREVALYRQGK
FREVKLARQQKAKLENMFSNMSEGDVAELNVIVKADVQ GSVEAIVQALNE
LSTNEVKVKVVGSGVGGITETDATLATASNAIIVGFNVRADATARRVIEA
ENIDLRYYSIIYELLNEIKAAMSGMLEPEFKQEIIGLAEVRDVFRHPKFG
AIAGCMVTEGVVKRNNPIRVLRDNVVIFEGELESLRRFKDDVSEVRNGME
CGIGVKNYNDVKVGDQIEVFEVVEVKRSI
> FTSZ_HELPY P56097 CELL DIVISION PROTEIN FTSZ. - HELICOBACTE
MVHQSEMENYNIGQASIEEVSDPAYKGAKIVVIGVGGGGSNMIKHLVEYG
VHQDVTPIATNTDGQHLKNNPAPVKILLGKESTGGLGAGGIPDIGRKAAE
ESANEIKEAIKDAKLVIISTGLGGGTGTGATPTIVKIAKEVGALTIAIVT
KPFKYEGNQKRKRAEEGLKELEQSSDSILVIPNDKILLTMKKNASTTECY
REVDDVLVRAVSGISTIITKPGNINVDFADLKSALGFKGFALMGIGEATG
EESAKLAVQNAIQSPLLDDASIEGAKSIIVFFEHHPDYPMMAYSQACDFI
QDQAHQDVDVKFGQHTSDNIPIDHVRVTIIATGAERNSGGASLESIATPS
QPVVKQTRKVGNGEYLKIPTEEELSIPTTMRIQQD
> P13627|CY1_PARDE Cytochrome c1 - Paracoccus denitrificans.
MTLRNASLTAVAALTVALAGGAVAQDASTAPGTTAPAGSSYHTNEAAPAA
ADTAPAAEAADEPAAEEAEAGEAEVTEEPAATETPAEEPAADEPAATEEP
DAEAEPAAEEAQATTEEAPAEEPAAEEPAAEEPAEEPAADAPAEEAAAEE
APAEPEAAAEEPAAEEPEATEEEAPAEEAAAEEAPAEEVVEDEAAADHGD
AAAQEAGDSHAAAHIEDISFSFEGPFGKFDQHQLQRGLQVYTEVCSACHG
LRYVPLRTLADEGGPQLPEDQVRAYAANFDITDPETEEDRPRVPTDHFPT
VSGEGMGPDLSLMAKARAGFHGPYGTGLSQLFNGIGGPEYIHAVLTGYDG
EEKEEAGAVLYHNAAFAGNWIQMAAPLSDDQVTYEDGTPATVDQMATDVA
AFLMWTAEPKMMDRKQVGFVSVIFLIVLAALLYLTNKKLWQPIKHPRKPE

> P33781|FMS5_ECOLI CS5 fimbrial subunit - Escherichia coli.
MKKNLLITSVLAMATVSGSVLAAVTNGQLTFNWQGVVPSAPVTQSSW AFV
NGLDIPFTPGTEQLNITLDSNKDITARSVKPYDFFIVPVSGNVTPGAPVT
RDTSANINSVNAFLSSVPVSNGFVGNKQLTLSTAVEAAKGEVAITLNGQA
LKVGSASPTVVTVASNKKESHISIDMNAKAAAADVAEGAAINFVAPVTFA
VDI
> P44336|SERC_HAEIN Phosphoserine aminotransferase - Haemophilus influenzae.
MSQVFNFSAGPAMIFPEV LQKAQNELINWLNQGVSVMEVSHRGKYFMELV
TQAEKDLREVYNIPDNYRTLFLQGGARGQFATIPMNLIGKKGKALYLNSG
HWSATAAKEARNFAEIDEITIVENGEQTRITDLDFSHIADQYDYVHYCPN
ETISGVEIFDVPNVGNAVLVADMSSNILSRQIDISKFGVIYAGAQKNLGP
AGITLVIIRDDLIGNARKETPSIWNYATQRDADSMINTPPTFAWYLCSLV
FKHLKEIGGLEIIEKRNAL KAQTLYDYIDSSKLYRNVVAKENRSTMNVTF
ITGNPELDAKFVAESTAAGLQALKGHKVLGGMRASIYNAMSQNGVEALIS
FMKKFETENLPQ
> KAD_HELPY P56104 ADENYLATE KINASE (EC 2.7.4.3) (ATP -AMP TRA
MKQLFLIIGAPGSGKTTDAELIAKNNSETIAHFSTGDLLRAESAKKTERG
LLIEKFTSQGELVPLEIVVETILSAIKSSGKGIILIDGYPRSVEQMQ ALD
KELNAQNEVILKSVIEVEVSENTAKERVLGRSRGADDNEKVFHNRMRVFL
DPLGEIQNFYKNKKVYKAIDGERSIEEIVGEMQEYILSFGN
> P24040|NIRS_PSEST Nitrite reductase - Pseudomonas stutzeri (Pseudomonas perfectomarina).
MSNVGKPILAGLIAGLSLLGLAVAQAAAPEMTAEEKEASKQIYFERCAGC
HGVLRKGATGKNLEPHW SKTEADGKKTEGGTLNLGTKRLENIIAYGTEGG
MVNYDDILTKEEINMMARYIQHTPDIPPEFSLQDMKDSWNLIVPVEKRVT
KQMNKINLQNVFAVTLRDAGKLALIDGDTHKIWKVLESGYAVHISRMSAS
GRYVYTTGRDGLTTIIDLWPEEPMTVATVRFGSDMRSVDVSKFEGYEDKY
LIGGTYWPPQYSIVDGLTLEPIKVVSTRGQTVDGEYHPEPRVASIVASHI
KPEWVVNVKETGQIILVD YTDLKNLKTTTIESAKFLHDGGWDYSKRYFMV
AANASNKVAAVDTKTGKLAALIDTAKIPHPGRGANFVHPQFGPVWSTGHL
GDDVVSLISTPSEESKYAKYKEHNWKVVQELKMPGAGNLFVKTHPKSKHF
WADAPMNPEREVAESVYVFDMNDLSKAPIQLNVAKDSGLPESKAIRRAVQ

PEYNKAGDEVWISLWGGKTDQSAIVIYDDKTLKLKRVITDPAVVTPTGKF
NVFNTMNDVY
> P55972|IF2_HELPY Translation initiation factor IF -2 - Helicobacter pylori (Campylobacter pylori).
MSGMVDLKEFLAELGKTQKELKNVIEQAKDIGLELKTNSKMTPEQAGKLY
KYIVDGIKEQIQANQPAKNPEQDNKDDLNTAVASKSLNKKVSKTPKKEET
KSQPKPKKTKEKKKEAPTPIAKKKGGIEIVNTFENQTPPTENTPKVVSHS
QIEKAKQKLQEIQKSREALNKLTQSNANNASNANNAKKEISEVKKQEQEI
KRHENIKRRTGFRVIKRNDEVENESENSVTESKKPTQSAAAIFEDIKKEW
QEKDKQEAKKAKKPSKPKATPTAKNNKSHKIDFSDARDFKGNDIYDDETD
EILLFDLHEQDNFNKEEEEKEIRQNINDRVRVQRKNPWMNESGIKRQSKK
KRAFRNDNSQKVIQSTTAIPEEVRVYEFAQKANLNLADVIKTLFNLGLMV
TKNDFLDKDSIEILAEEFHLEISVQNTLEEFEVEEVLEGVKKERPPVVTI
MGHVDHGKTSLLDKIRDKRVAHTEAGGITQHIGAYMVEKNDKWVSFIDTP
GHEAFSQMRNRGAQVTDIAVIVIAADDGVKQQTIEALEHAKAANVPVIFA
MNKMDKPNVNPDKLKAECAELGYNPVDWGGEHEFIPVSAKTGDGIDNLLE
TILIQAGIMELKAIEEGSARAVVLEGSVEKGRGAVATVIVQSGTLSVGDS
FFAETAFGKVRTMTDDQGKSIQNLKPSMVALITGLSEVPPAGSVLIGVEN
DSIARLQAQKRATYLRQKALSKSTKVSFDELSEMVANKELKNIPVVIKAD
TQGSLEAIKNSLLELNNEEVAIQVIHSGVGGITENDLSLVSSSEHAVILG
FNIRPTGNVKNKAKEYNVSIKTYTVIYALIEEMRSLLLGLMSPIIEEEHT
GQAEVRETFNIPKVGTIAGCVVSDGVIARGIKARLIRDGVVIHTGEILSL
KRFKDDVKEVSKGYECGIMLDNYNEIKVGDVFETYKEIHKKRTL
> P33129|HTRE_ECOLI Outer membrane usher protein htrE - Escherichia coli.
MTIEYTKNYHHLTRIATFCALLYCNTAFSAELVEYDHTFLMGQNASNIDL
SRYSEGNPAIPGVYDVSVYVNDQPIINQSITFVAIEGKKNAQACITLKNL
LQFHINSPDINNEKAVLLARDETLGNCLNLTEIIPQASVRYDVNDQRLD I
DVPQAWVMKNYQNYVDPSLWENGINAAMLSYNLNGYHSETPGRKNESIYA
AFNGGMNLGAWRLRASGNYNWMTDSGSNYDFKNRYVQRDIASLRSQLILG
ESYTTGETFDSVSIRGIRLYSDSRMLPPTLASFAPIIHGVANTNAKVTIT
QGGYKIYETTVPPGAFVIDDLSPSGYGSDLIVTIEESDGSKRTFSQPFSS
VVQMLRPGVGRWDISGGQVLKDDIQDEPNLFQASYYYGLNNYLTGYTGIQ
ITDNNYTAGLLGLGLNTSVGAFSFDVTHSNVRIPDDKTYQGQSYRVSWNK
LFEETSTSLNIAAYRYSTQNYLGLNDALTLIDEVKHPEQDLEPKSMRNYS
RMKNQVTVSINQPLKFEKKDYGSFYLSGSWSDYWASGQNRSNYSIGYSNS
TSWGSYSVSAQRSWNEDGDTDDSVYLSFTIPIEKLLGTEQRTSGFQSIDT
QISSDFKGNNQLNVSSSGYSDNARVSYSVNTGYTMNKASKDLSYVGGYAS
YESPWGTLAGSISANSDNSRQVSLSTDGGFVLHSGGLTFSNDSFSDSDTL
AVVQAPGAQGARINYGNSTIDRWGYGVTSALSPYHENRIALDINDLENDV
ELKSTSAVAVPRQGSVVFADFETVQGQSAIMNITRSDGKNIPFAADIYDE
QGNVIGNVGQGGQAFVRGIEQQGNISIKWLEQSKPVSCLAHYQQSPEAEK
IAQSIILNGIRCQIQ
> P69797|PTNAB_ECOLI PTS system mann ose-specific EIIAB component - Escherichia coli.
MTIAIVIGTHGWAAEQLLKTAEMLLGEQENVGWIDFVPGENAETLIEKYN
AQLAKLDTTKGVLFLVDTWGGSPFNAASRIVVDKEHYEVIAGVNIPMLVE
TLMARDDDPSFDELVALAVETGREGVKALKAKPVEKAAPAPAAAAPKAAP
TPAKPMGPNDYMVIGLARIDDRLIHGQVATRWTKETNVSRIIVVSDEVAA
DTVRKTLLTQVAPPGVTAHVVDVAKMIRVYNNPKYAGERVMLLFTNPTDV
ERLVEGGVKITSVNVGGMAFRQGKTQVNNAVSVDEKDIEAFKKLNARGIE
LEVRKVSTDPKLKMMDLISKIDK
> P0AFH8|OSMY_ECOLI Osmotically -inducible protein Y - Escherichia coli.
MTMTRLKISKTLLAVMLTSAVATGSAYAENNAQTTNESAGQKVDSSMNKV
GNFMDDSAITAKVKAALVDHDNIKSTDISVKTDQKVVTLSGFVESQAQAE
EAVKVAKGVEGVTSVSDKLHVRDAKEGSVKGYAGDTATTSEIKAKLLADD
IVPSRHVKVETTDGVVQLSGTVDSQAQSDRAESIAKAVDGVKSVKNDLKT
K
> Q47474|PMEB_DICD3 Pectinesterase B - Dickeya dadantii (strain 3937) (Erwinia chrysanthemi (strain 3937)).
MSLTHYSGLAAAVSMSLILTACGGQTPNSARFQPVFPGTVSRPVLSAQEA
GRFTPQHYFAHGGEYAKPVADGWTPTPIDTSRVTAAYVVGPRAGVAGATH
TSIQQAVNAALRQHPGQTRVYIKLLPGTYTGTVYVPEGAPPLTLFGAGDR
PEQVVVSLALDSMMSPADYRARVNPHGQYQPADPAWYMYNACATKAGATI

NTTCSAVMWSQSNDFQLKNLTVVNALLDTVDSGTHQAVALRTDGESGATG
KCPPAQPSDTFFVNTSDRQNSYVTDHYSRAYIKDSYIEGDVDYVFGRATA
VFDRVRFHTVSSRGSKEAYVFAPDSIPSVKYGFLVINSQLTGDNGYRGAQ
KAKLGRAWDQGAKQTGYLPGKTANGQLVIRDSTIDSSYDLANPWGAAATT
DRPFKGNISPQRDLDDIHFNRLWEYNTQVLLHE
> P56060|KDSA_HELPY 2-dehydro-3-deoxyphosphooctonate aldolase - Helicobacter pylori (Campylobacter pylori).
MKTSKTKTPKSVLIAGPCVIESLENLRSIATKLQPLANNERLDFYFKASF
DKANRTSLESYRGPGLEKGLEMLQTIKEEFGYKILTDVHESYQASVAAKV
ADILQIPAFLCRQTDLIVEVSQTNAIVNIKKGQFMNPKDMQYSVLKALKT
RDKSIQSPTYETALKNGVWLCERGSSFGYGNLVVDMRSLKIMREFAPVIF
DATHSVQMPGGANGKSS GDSSFAPILARAAAAVGIDGLFAETHVDPKNAL
SDGANMLKPDELEQLVTDMLKIQNLF
> Q55653|SYH_SYNY3 Histidyl-tRNA synthetase - Synechocystis sp. (strain PCC 6803).
MGAIQAIRGTRDILPPETNYWQWVEAIAKSILDRALYQEIRTPIFEQTSL
FERGIGEATDVVGKEMYSFTDRGDRPITLRPEGTAGVVRAYIEQNLQAAG
GVQRLWYTGPMFRYERPQAGRQRQFHQLGVEVLGSADPRADVEVIALGTD
ILKALGLSNLSLALNSVGNGGDRQRYREALIAYLTPFKAELDPDSQDRLE
RNPLRILDSKAKRTQEIVQDAPSILDHLGVDSQRHFDQVQQLLTNLGIAY
QLTPTLVRGLDYYTHTAFEIQSSDLGAQATVCGGGRYDGLVAELGGPVTP
AVGWAMGLERLIILLQQMATPPAPSPDLYLISKGEKAEPQALILAQKLRN
QGLAVALDLSA SAFGKQFKRADKSGAIACLVLGDGEIATGTVQLKWLADK
AQETLQLQDLMGNITELKQRLAGHREKYPHLTSNFSVTCHDPMDNLV
> P65106|IF1_CHLTR Translation initiation factor IF-1 - Chlamydia trachomatis.
MAKKEDTIVLEGRVEELLPGMHFRVMLENGVPITAHLCGKMRMSNIRLLV
GDRVTVEMSTYDLTKARVVYRHR
> P48464|TYSY_SHIFL Thymidylate synthase - Shigella flexneri.
MKQYLELMQKVLDEGTQKNDRTGTGTLSIFGHQMRFNLQDGFPLVTTKRC
HLRSIIHELLWFLQGDTNIAYLHENNVTIWDEWADENGDLGPVYGKQWRA
WPTPDGRHIDQITTVLNQLKNDPDSRRIIVSAWNVGELDKMALAPCHAFF
QFYVADGKLSCQLYQRSCDVFLGLPFNIASYALLVHMMAQQCDLEVGDFV
WTGGDTHLYSNHMDQTHLQLSREPRPLPKLIIKRKPESIFDYRFEDFEIE
GYDPHPGVKAPVAI
> HLYX_ACTPL P23619 REGULATORY PROTEIN HLYX. - ACTINOBACILLUS
MKIVSDAKHTGRTRCTIHCQNCSISQLCLPFTLSEHELTQLDNIIERKKP
VQKSQIIFQSGDELRSIYAIRSGTIKSYTISESGEEQITAFHLPGDLVGF
DAIMNMKHVGFAQALETSMICEIPFDILDD LAGKMPKIRHQIMRLMSNEI
KSDQEMILLLSKMSAEEKLAAFLHNLSQRYAAPGFSAREFRLTMTRGDIG
NYLGLTIETISRLLGRFQKSGMITVQGKYITINRMDELTV
> P00805|ASPG2_ECOLI L-asparaginase 2 - Escherichia coli.
MEFFKKTALAALVMGFSGAALALPNITILATGGTIAGGGDSATKSNYTVG
KVGVENLVNAVPQLKDIANVKGEQVVNIGSQDMN DNVWLTLAKKINTDCD
KTDGFVITHGTDTMEETAYFLDLTVKCDKPVVMVGAMRPSTSMSADGPFN
LYNAVVTAADKASANRGVLVVMNDTVLDGRDVTKTNTTDVATFKSVNYGP
LGYIHNGKIDYQRTPARKHTSDTPFDVSKLNELPKVGIVYNYANASDLPA
KALVDAGYDGIVSAGVGNGNLYKSVFDTLATAAKTGTAVVRSSRVPTGAT
TQDAEVDDAKYGFVASGTLNPQKARVLLQLALTQT KDPQQIQQIFNQY
> P07464|THGA_ECOLI Galactoside O-acetyltransferase - Escherichia coli.
MNMPMTERIRAGKLFTDMCEGLPEKRLRGKTLMYEFNHSHPSEVEKRESL
IKEMFATVGENAWVEPPVYFSYGSNIHIGRNFYANFNLTIVDDYTVTIGD
NVLIAPNVTLSVTGHPVHHELRKNGEMYSFPITIGNNVWIGSHVVINPGV
TIGDNSVIGAGSIVTKD IPPNVVAAGVPCRVIREINDRDKHYYFKDYKVE
SSV
> Q05811|OM3A_RHILV Outer membrane protein IIIA - Rhizobium leguminosarum bv. viciae.
MNIRMVLLASAAAFAASTPVLAADAIVAAEPEPVEYVRVCDAYGTGYFYI
PGTETCLKIEGYIRFQVNVGDNPGGDNDSDWDAVTAVRFSSRKSDTEYGP
LTGVIVMQFNADNASDQDAILDSAYLDVAG FRAGLFYSWWDDGLSGETDD
IGSVVTLHNSIRYQYESGTFYAGLSVDELEDGVYQGTFTPGVIPGTTDFT
ADDGPNNVGVAFGIGGTAGAFSYQVTGGWDVDNEDGAIRAMGTVEIGPGT
FGLAGVYSSGPNSYYSSAEWAVAAEYAIKATDKLKITPGRWHGHVPEDFD

GLGDAWKVGLTVDYQIVENFYAKASVQYLDPQDGEDSTSGYFACSVRSNH
LVDAPGLRIGSTTISF
> P56146|SYFA_HELPY Phenylalanyl-tRNA synthetase alpha chain - Helicobacter pylori (Campylobacter pylori).
MHTLIERLEKVTNSKELEEARLNALGKKGVFADKFNQLKHLNGEEKNAFA
KEIHHYKQAFEKAFEWKKKAIIELELEERLKKEKIDVSLFNAIKTSSSHP
LNYTKNKIIEFFTPLGYKLEIGSLVEDDFHNFSALNLPPYHPARDMQDTF
YFKDHKLLRTHTSPVQIHTMQEQTPPIKMICLGETFRRDYDLTHTPMFHQ
IEGLVVDQKGNIRFTHLKGVIEDFLHYFFGGVKLRWRSSFFPFTEPSAEV
DISCVFCKQEGCRVCSHTGWLEVLGCGMVNNAVFEAIGYENVSGFAFGMG
IERLAMLTCQINDLRSFFETDLRVLESF
> P32670|PTFX2_ECOLI Multiphosphoryl transfer protein 2 - Escherichia coli.
MALIVEFICELPNGVHARPASHVETLCNTFSSQIEWHNLRTDRKGNAKSA
LALIGTDTLAGDNCQLLISGADEQEAHQRLSQWLRDEFPHCDAPLAEVKS
DELEPLPVSLTNLNPQIIRARTVCSGSAGGILTPISSLDLNALGNLPAAK
GVDAEQSALENGLTLVLKNIEFRLLDSDGATSAILEAHRSLAGDTSLREH
LLAGVSAGLSCAEAIVASANHFCEEFSRSSSSYLQERALDVRDVCFQLLQ
QIYGEQRFPAPGKLTQPAICMADELTPSQFLELDKNHLKGLLLKSGGTTS
HTVILARSFNIPTLVGVDIDALTPWQQQTIYIDGNAGAIVVEPGEAVARY
YQQEARVQDALREQQRVWLTQQARTADGIRIEIAANIAHSVEAQAAFGNG
AEGVGLFRTEMLYMDRTSAPGESELYNIFCQALESANGRSIIVRTMDIGG
DKPVDYLNIPAEANPFLGYRAVRIYEEYASLFTTQLRSILRASAHGSLKI
MIPMISSMEEILWVKEKLAEAKQQLRNEHIPFDEKIQLGIMLEVPSVMFI
IDQCCEEIDFFSIGSNDLTQYLLAVDRDNAKVTRHYNSLNPAFLRALDYA
VQAVHRQGKWIGLCGELGAKGSVLPLLVGLGLDELSMSAPSIPAAKARMA
QLDSRECRKLLNQAMACRTSLEVEHLLAQFRMTQQDAPLVTAECITLESD
WRSKEEVLKGMTDNLLLAGRCRYPRKLEADLWAREAVFSTGLGFSFAIPH
SKSEHIEQSTISVARLQAPVRWGDDEAQFIIMLTLNKHAAGDQHMRIFSR
LARRIMHEEFRNALVNAASADAIASLLQHELEL
> P04744|PAPB_ECOLI Major pilu subunit operon regulatory protein papB - Escherichia coli.
MAHHEVISRSGNAFLLNIRESVLLPGSMSEMHFFLLIGISSIHSDRVILA
MKDYLVGGHSRKEVCEKYQMNNGYFSTTLGRLIRLNALAAR LAPYYTDES
SAFD
> P25447|FAEF_ECOLI K88 minor fimbrial subunit faeF - Escherichia coli.
MKKTMMAAALVLSALSIQSALAAEYSEKTQYLGVVNGQVVGNSVVKVTRT
PTDPVLYRSGSNSPLPAELIIRHAESRPASGGLANITVKEALPDNGEARI
TLKTSLMVDGKRVALSARQQGEDVVITVPEAQQQIELRTDAPAELEVPVS
YRGNLQIALQVED
> P14756|ELAS_PSEAE Pseudolysin - Pseudomonas aeruginosa.
MKKVSTLDLLFVAIMGVSPAAFAADLIDVSKLPSKAAQGAPGPVTLQAAV
GAGGADELKAIRSTTLPNGKQVTRYEQFHNGVRVVGEAITEVKGPGKSVA
AQRSGHFVANIAADLPGSTTAAVSAEQVLAQAKSLKAQGRKTENDKVELV
IRLGENNIAQLVYNVSYLIPGEGLSRPHFVIDAKTGEVLDQWEGLAH AEA
GGPGGNQKIGKYTYGSDYGPLIVNDRCEMDDGNVITVDMNSSTDDSKTTP
FRFACPTNTYKQVNGAYSPLNDAHFFGGVVFKLYRDWFGTSPLTHKLYMK
VHYGRSVENAYWDGTAMLFGDGATMFYPLVSLDVAAHEVSHGFTEQNSGL
IYRGQSGGMNEAFSDMAGEAAEFYMRGKNDFLIGYDIKKGSGALRYMDQP
SRDGRSIDNASQYYNGIDVHHSSGVYNRAFYLLANSPGWDTRKAFEVF VD
ANRYYWTATSNYNSGACGVIRSAQNRNYSAADVTRAFSTVGVTCPSAL
> P26877|LIP_PSEU0 Lactonizing lipase - Pseudomonas sp. (strain 109).
MKKKSLLPLGLAIGLASLAASPLIQASTYTQTKYPIVLAHGMLGFDNILG
VDYWFGIPSALRRDGAQVYVTEVSQLDTSEVRGEQLLQQVEEIVALSGQP
KVNLIGHSHGGPTIRYVAAVRPDLMPSATSVG APHKGSDTADFLRQIPPG
SAGEAVLSGLVNSLGALISFLSSGSAGTQNSLGSLESLNSEGAARFNAKY
PQGIPTSACGEGAYKVNGVSYYSWSGSSPLTNFLDPSDAFLGASSLTFKN
GTANDGLVGTCSSHLGMVIRDNYRMNHLDEVNQVFGLTSLFETSPVSVYR
QHANRLKNASL
> P08306|COX2_PARDE Cytochrome c oxidase subunit 2 - Paracoccus denitrificans.
MMAIATKRRGVAAVMSLGVATMTAVPALAQDVLGDLPVIGKPVNGGMNFQ
PASSPLAHDQQWLDHFVLYIITAVTIFVCLLLLICIVRFNRRANPVPARF
THNTPIEVIWTLVPVLILVAIGAFSLPILFRSQEMPNDPDLVIKAIGHQW

YWSYEYPNDGVAFDALMLEKEALADAGYSEDEYLLATDNPVVVPVGKKVL
VQVTATDVIHAWTIPAFAVKQDAVPGRIAQLWFSVDQEGVYFGQC SELCG
INHAYMPIVVKAVSQEKYEAWLAGAKEEFAADASDYLPASPVKLASAE
> P19845|NOSY_PSEST Membrane protein nosY - Pseudomonas stutzeri (Pseudomonas perfectomarina).
MNQVWNIARKELSDGLRNRWLLAISLLFAVLAVGIAWLGAAASGQLGFTS
IPATIASLASLATFLMPLIALLLAYDAIVGEDEGGTLMLLLTYPLGRGQI
LLGKFVGHGLILALAVLIGFGCAALAIALLVEGVELGMLFWAFGRFMISS
TLLGWVFLAFAYVLSGKVNEKSSAAGLALGVWFLFVLVFDLVLLALLVLS
EGKFNPELLPWLLLLNPTDIYRLINLSGFEGSGSAMGVLSLGADLPVPAA
VLWLCLLAWIGVSLLLAYAIFRRRLT
> P09888|OMPC_NEIGO Outer membrane protein P.IIC - Neisseria gonorrhoeae.
MQPAKNLLFSSLLFSSLLFSSAARAASEDGGRGPYVQADLAYAAERITHD
YPKPTGTGKNKISTVSDYFRNIRTHSVHPRVSVGYDFGSWRIAADYARYR
KWNNNKYSVSIKELLRNDNSASGVRGHLNIQTQKTEHQENGTFHAVSSLG
LSTIYDFDTGSRFKPYIGMRVAYGHVRHQVRSVEQETEIITTYPSNGGGK
VSLSSKMPPKSAHHQSNSIRRVGLGVIAGVGFDITPNLTLDTGYRYHNWG
RLENTRFKTHEASLGMRYRF
> P0A9K1|PHOH_ECOLI Protein phoH - Escherichia coli.
MVTSCTGHVLDNQRATTRGVFSSGSHLVTLHFQPHPFFSCVTDAVNGARS
RFSAFYPKANYGLQGSQPSDVRAHNRAANGACDEYKQLKVLSMGRQKAVI
KARREAKRVLRRDSRSHKQREEESVTSLVQMGGVEAIGMARDSRDTSPIL
ARNEAQLHYLKAIESKQLIFATGEAGCGKTWISAAK AAEALIHKDVDRII
VTRPVLQADEDLGFLPGDIAEKFAPYFRPVYDVLVRRLGASFMQYCLRPE
IGKVEIAPFAYMRGRTFENAVVILDEAQNVTAAQMKMFLTRLGENVTVIV
NGDITQCDLPRGVCSGLSDALERFEEDEMVGIVRFGKEDCVRSALCQRTL
HAYS
> P31550|THIB_ECOLI Thiamine -binding periplasmic protein - Escherichia coli.
MLKKCLPLLLLCTAPVFAKPVLTVYTYDSFAADWGPGPVVKKAFEADCNC
ELKLVALEDGVSLLNRLRMEGKNSKADVVLGLDNNLLDAASKTGLFAKSG
VAADAVNVPGGWNNDTFVPFDYGYFAFVYDKNKLKNPPQSLKELVESDQN
WRVIYQDPRTSTPGLGLLLWMQKVYGDDAPQAWQKLAKKTVTVTKGWSEA
YGLFLKGESDLVLSYTTSPAYHILEEKKDNYAAANFSEGHYLQVEVAART
AASKQPELAQKFLQFMVSPAFQNAIPTGNWMYPVANVTLPAGFEKLTKPA
TTLEFTPAEVAAQRQAWISEWQRAVSR
> P13907|PSBO_ANASP Photosystem II manganese -stabilizing polypeptide - Anabaena sp. (strain PCC 7120).
MRYRALIVAFLAVCLGLLTACSDAPASSTRDILTYEQIRGTGLANKCPQL
TETSRGSIPLDSSKSYVLKELCLEPTNFFV KEEPANKRQTAEFVAGKLLT
RYTSTIDQVSGDLKFNDDSSLTFVEKDGLDFQAITVQLPGGERVPFLFTI
KNLVAQTQPGLSSLNTSTDFEGTFKVPSYRGAAFLDPKGRGVVSGYDNAV
ALPAQADDEDLTRTNVKRAEILNGKISLQIAKVDSSSGEIAGTFESEQPS
DTDLGADEPKEVKIRGIFYARVE
> MALQ_ECOLI P15977 4 -ALPHA-GLUCANOTRANSFERASE (EC 2.4.1.2 5)
MESKRLDNAALAAGISPNYINAHGKPQSISAETKRRLLDAMHQRTATKVA
VTPVPNVMVYTSGKKMPMVVEGSGEYSWLLTTEEGTQYKGHVTGGKAFNL
PTKLPEGYHTLTLTQDDQRAHCRVIVAPKRCYEPQALLNKQKLWGACVQL
YTLRSEKNWGIGDFGDLKAMLVDVAKRGGSFIGLNPIHALYPANPESASP
YSPSSRRWLNVIYIDVNAVEDFHLSEEAQAWWQLPTTQQTLQQARDAD WV
DYSTVTALKMTALRMAWKGFAQRDDEQMAAFRQFVAEQGDSLFWQAAFDA
LHAQQVKEDEMRWGWPAWPEMYQNVDSPEVRQFCEEHRDDVDFYLWLQWL
AYSQFAACWEISQGYEMPIGLYRDLAVGVAEGGAETWCDRELYCLKASVG
APPDILGPLGQNWGLPPMDPHIITARAYEPFIELLRANMQNCGALRIDHV
MSMLRLWWIPYGETADQGAYVHYPVDDLLSILALESKRHRCMVIGEDLG T
VPVEIVGKLRSSGVYSYKVLYFENDHEKTFRAPKAYPEQSMAVAATHDLP
TLRGYWECGDLTLGKTLGLYPDEVVLRGLYQDRELAKQGLLDALHKYGCL
PKRAGHKASLMSMTPTLNRGLQRYIADSNSALLGLQPEDWLDMAEPVNIP
GTSYQYKNWRRKLSATLESMFADDGVNKLLKDLDRRRRAAAKKK
> ASRB_SALTY P26475 ANAEROBIC SULFITE REDUCTASE SUBUNIT B (EC
MSHCSCHDKPQHSLLAAAYRILSITRHTPLEWNFRVAVDFPAHWGQFVEV
SLPRVGEAPISVSDYGDGWIDLLIRNVGKVTSALFTLKEGDNVWLRGCYG
NGYPVDTLRHKPLLVVAGGTGVAPVKGLMRYFVENPQEIGQLDMILGYKN
RDCVLYKEEMATWRGKHNLVLTLDEGEADDRYQIGRVTDRLADMTLSDID

TMQAIVVGPPIMITFTVKMLLQKGLKPEQIWVDYERRMACSVGKCG HCRM
GEVYVCTDGPIFNYAVAQRFAD
> P36912|EBA2_FLAME Endo-beta-N-acetylglucosaminidase F2 - Flavobacterium meningosepticum (Chryseobacterium meningosepticum) (Elizabethkingia meningoseptica).
MKTANFSFALCLSVVIMLFIKCTRSEQDLSVTKDAIAQKSGVTVSAVNLS
NLIAYKNSDHQISAGYYR TWRDSATASGNLPSMRWLPDSLDMVMVFPDYT
PPENAYWNTLKTNYVPYLHKRGTKVIITLGDLNSATTTGGQDSIGYSSWA
KGIYDKWVGEYNLDGIDIDIESSPSGATLTKFVAATKALSKYFGPKSGTG
KTFVYDTNQNPTNFFIQTAPRYNYVFLQAYGRSTTNLTTVSGLYAPYISM
KQFLPGFSFYEENGYPGNYWNDVRYPQNGTGRAYDYARWQPATGKKGGVF
SYAIERDAPLTSSNDNTLR APNFRVTKDLIKIMNP
> P22365|AMCY_PARVE Amicyanin - Paracoccus versutus (Thiobacillus versutus).
MISAKTLRPAIAAIALFAIGATGAWAQDKITVTSEKPVAAADVPADAVVV
GIEKMKYLTPEVTIKAGETVYWVNGEVMPHNVAFKKGIVGEDAFRGEMMT
KDQAYAITFNEAGSYDYFCTPHPFMRGKVIVE
> P24404|LEU3_AGRT5 3-isopropylmalate dehydrogenase - Agrobacterium tumefaciens (strain C58 / ATCC 33970).
MTVRSLFLLPGDGIGPEAMTEVRKLIEYMNSAHNAGFTVSEGLVGGSAYD
AHGVAISDADMEKALAADAILFGAVGGPKWDGVPYEHRPEAGLLRLRKDL
ELFANLRPAICYPALAAASSLKPELVEGLDILIVRELTGGVYFGEPKQII
DLGNGQKRGIDTQIYDTFEIERI ASVAFELARSRDNRVCSMEKRNVMKSG
VLWNQVVTETHAAKYKDVQLEHMLADAGGMQLVRKPKQFDVIVTDNLFGD
MLSDVAAMLTGSLGMLPSASLGAPDAKTGKRKAMYEPVHGSAPDIAGKSI
ANPIAMIASFAMCLRYSFNMVDEATKLEAAIANVLDKGIRTADIMADGCR
QVGTSDMGDAVLAEFKALSA
> P07862|DDLB_ECOLI D-alanine--D-alanine ligase B - Escherichia coli.
MTDKIAVLLGGTSAEREVSLNSGAAVLAGLREGGIDAYPVDPKEVDVTQL
KSMGFQKVFIALHGRGGEDGTLQGMLELMGLPYTGSGVMASALSMDKLRS
KLLWQGAGLPVAPWVALTRAEFEKGLSDKQLAEISALGLPVIVKPSREGS
SVGMSKVVAENALQDALRLAFQHDEEVLIEKWLSGPEFTVAILGEEILPS
IRIQPSGTFYDYEAKYLSDETQYFCPAGLEASQEAN LQALVLKAWTTLGC
KGWGRIDVMLDSDGQFYLLEANTSPGMTSHSLVPMAARQAGMSFSQLVVR
ILELAD
> P24037|C552_PSEST Cytochrome c-552 - Pseudomonas stutzeri (Pseudomonas perfectomarina).
MKKTLMASAVGAVIAFGTHGAMAAAPADWSSVAATDVTLFYPGVSPVEWI
TKGTEHGGARALKKGETCAGCHSEEASDMGEKMASGKKLEPS PIAGKAPF
INAKVQAANDGENLYLRFTWKQPAASGAAPMDADNPVKIAYMLEGGSKVE
LAEAGGCWGSCHGDARTMPGAADTKTKYVKDGSLANGVYYDLNQWRSGEN
KAFDGYVATERVMEGGQALVDAQGKLDGDTWTVVFTRKFAGGEGDVTLAP
GNLYNFGFAIHDDSATGRYHHVSLGYSLGIDAQGDITAAKQ
> P18103|FANC_ECOLI K99 fimbrial protein - Escherichia coli.
MKKTLLAIILGGMAFATTNASANTGTINFNGKITSATCTIDPEVNGNRTS
TIDLGQAAISGHGTVVDFKLKPAPGSNDCLAKTNARIDWSGSMNSLGFNN
TASGNTAAKGYHMTLRATNVGNGSGGANINTSFTTAEYTHTSAIQSFNYS
AQLKKDDRAPSNGGYKAGVFTTSASFLVTYM
> P74764|SYFB_SYNP7 Phenylalanyl-tRNA synthetase beta chain - Synechococcus sp. (strain PCC 7942) (Anacystis nidulans R2).
MRISLNWLRELVQVDLEPEVLAEKLTLAGFEVEEIEDRRTWAAGVVVGRV
LEREQHPNADRLSVCQVEIGQAEPVTIVCGASNVRADIWVAVATLGSYLP
CIDLKLKPTKLRGVRSEGMICSLSELGLTKESEGIHIFPEDAGLQAGQPV
GPLLGLDDVVLDLTSTANRADALSLIGIAREVRALTAATLTL PEVELQTY
PELPCLAISLQSEACSHYSGTIIEGVTIAPSPEWLQKRLQLAGIRTINNV
VDITNYILLEYGQPLHAFDRQKLQAIAGSSDLAIGVRSAQAGETLKTLDD
QERTLAEAALVITAGDCPVALAGVMGGADSEVSQETTQLLLEAAWFEPIA
VRRSARSQGLRTEASARYERGVNVTELPIATQRAIDLLLQIAGGTVISQT
VATTTQTEPEHSITLRLQRINELLGPVQAEDEELKDLGADDIE RLLTAIG
CHLTLVDDAVWQVRVPPYRYRDLEREIDLIEEVARLYGYDNFGETLPPLG
SDEGALSIDESLRRQIRAVCRGVGLTELQHYSLVKPGSDRQVHLANPLLA
EYSALRLDLLSGLIDAFQYNWEQGNGPLWGFEIGRIFWREEDGFFEADRM
GGILGGDPSRGRWQRGGKEQAIDWYAAKGVLEEIFERFGLTIEFQPDRQD
DRFHPGRTASLWLQGDRLGRFGQLHPSLCEGRGLPAEVYAFELD LDVWLD

HLDQPERQVPRFQPYSSFPASDRDLAFFVDQSVTVAELERIIRRQGGALL
SEVELFDQYCGEHVPENQRSLAFRLTYRASDRTLTEAEVEPVHDQVRQSL
VERFRVTLRS
> NDK_SYNY3 P74494 NUCLEOSIDE DIPHOSPHATE KINASE (EC 2.7.4.6)
MERTFIMIKPDGVQRQLIGEIVGRFEKKGFKLVAMKVMTVSQELAEKHYE
ALNDKPFFSGLVNFICSSPVVAM VWEGNSIVSTSRQMIGATDPHAAAPGT
IRGDYGVSVGRNIIHGSDAIETAKREISLWFKDEEVNEWDATLNPWLYE
> P29739|OMPH_PHOPR Porin -like protein H - Photobacterium profundum (Photobacterium sp. (strain SS9)).
MKKTLVALAILTAAGSANAGINLYDADGVKTDLSGAAEVQYRQTFKEDSD
AELRMDDGDLAVNTTVAISDSLNA VAAVAFEFEDGKVTNDELWVGVAGDF
GTLTAGRQYMLADDAGVGKDYELGGDGIDFVQANGDQVVKYVFDNGQFYG
GVGALITETNPDNNADEASVYEGRLGARFGDFDVRAYLYSGEDVNTDNFD
VFGDDKVNVDIDGYQIEAEYIVNAFAFAASFGQVDYELASDSSQKIEADT
AALAGSYTMNKTTFAVGYTYWSPEAKGTVKKMEEANVFYANVTQQLHSNV
KVYGEIGSSDTDNSEFGYVAGMEVT F
> P42512|FPTA_PSEAE Fe(3+) -pyochelin receptor - Pseudomonas aeruginosa.
MKTETKVIKGRQGIARNRHTPLCLGLLLALSPLAAAVADARKDGETELPD
MVISGESTSATQPPGVTTLGKVPLKPRELPQSASVIDHERLEQQNLFSLD
EAMQQATGVTVQPFQLLTTAYYVRGFKVDSFELDGVPALLGNTASSPQDM
AIYERVEILRGSNGLLHGTGNPAATVNLV RKRPQREFAASTTLSAGRWDR
YRAEVDVGGPLSASGNVRGRAVAAYEDRDYFYDVADQGTRLLYGVTEFDL
SPDTLLTVGAQYQHIDSITNMAGVPMAKDGSNLGLSRDTYLDVDWDRFKW
DTYRAFGSLEQQLGGGWKGKVSAEYQEADSRLRYAGSFGAIDPQTGDGGQ
LMGAAYKFKSIQRSLDANLNGPVRLFGLTHELLGGVTYAQGETRQDTARF
LNLPNTPVNVYRWDPHGVPRPQIGQYTSPG TTTTTQKGLYALGRIKLAEP
LTLVVGGRESWWDQDTPATRFKPGRQFTPYGGLIWDFARDWSWYVSYAEV
YQPQADRQTWNSEPLSPVEGKTYETGIKGELADGRLNLSLAAFRIDLENN
PQEDPDHPGPPNNPFYISGGKVRSQGFELEGTGYLTPYWSLSAGYTYTST
EYLKDSQNDSGTRYSTFTPRHLLRLWSNYDLPWQDRRWSVGGGLQAQSDY
SVDYRGVSMRQGGYALVNMRLGYKIDEHWTA AVNVNNLFDRTYYQSLSNP
NWNNRYGEPRSFNVSLRGAF
> P43478|CGKA_ALTCA Kappa -carrageenase - Alteromonas carrageenovora (Pseudoalteromonas carrageenovora).
MKPISIVAFPIPAISMLLLSAVSQAASMQPPIAKPGETWILQAKRSDEFN
VKDATKWNFQTENYGVWSWKNENATVSNGKLKLTTKRESHQRTFWDGCNQ
QQVANYPLYYTSGVAKSRATGNYGYYEARIKGASTFPGVSPAFWMYSTID
RSLTKEGDVQYSEIDVVELTQKSAVRESDHDLHNIVVKNGKPTWMRPGSF
PQTNHNGYHLPFDPRNDFHTYGVNVTKDKITWYVDGEIVGEKDNLYWHRQ
MNLTLSQGLRAPHTQWKCNQFYPSANKSAEGFPTSMEVDYVRTWVKVGNN
NSAPGEGQSCPNTFVAVNSVQLSAAKQTLRKGQSTTLESTVLPNCATNKK
VIYSSSNKNVATVNSAGVVKAKNKGTATITVKTKNKGKIDKLTIAVN
> Q2RVM4|CYC2_RHORT Cytochrome c2 - Rhodospirillum rubrum (strain ATCC 11170 / NCIB 8255).
MKKGFLAAGVFAAVAFASGAALAEGDAAAGEKVSKKCLACHTFDQGGANK
VGPNLFGVFENTAAHKDDYAYSESYTEMKAKGLTWTEANLAAYVKDPKAF
VLEKSGDPKAKSKMTFKLTKDDEIE NVIAYLKTLK
> FRZC_MYXXA P43500 FRZCD PROTEIN (FRIZZY AGGREGATION PROTEIN
MSLDTPNEKPAGKARARKAPASKAGATNAASTSSSTKAITDTLLTVLSGN
LQARVPKELVGESGVELAHLLNQVLDQFAASEHRKHVAAQEIDQALDALI
GLVREGDLSRWNTTTEDPQLGPLLEGFGKVIETLRTFVREINEAALRLSS
SANQVLAASTQHETSSTEQAAAIHETTATM EELKHASAQIAENAGSVARV
AEETLGAARAGRGAIGEFIQAMQQIRSDGVAVADSIAKLSKRVERIGTVV
EVIDEIADRSDLLALNAALEGSRAGEAGKGFSIVAAEMRRLAENVLDSTK
EIKNLITEIREATAAAAGAAEASKSATESGEKLGAVAAQAVEGILAGVQE
TSDAARVINLATQQQRTATEQVVASMAEIEDVTRQTTQASKQATGAAAEL
TQLAGRLAELIKRFKAD
> P26997|XYLA_THET8 Xylose isomerase - Thermus thermophilus (strain HB8 / ATCC 27634 / DSM 579).
MYEPKPEHRFTFGLWTVGNVGRDPFGDAVRERLDPVYVVHKLAELGAYGV
NLHDEDLIPRGTPPQERDQIVRRFKKALDETGLKVPMVTANLFSDPAFKD
GAFTSPDPWVRAYALRKSLETMDLGAELGAEIYVVWPGREGAEVEATGKA
RKVWDWVREALNFMAAYAE DQGYGYRFALEPKPNEPRGDIYFATVGSMLA

FIHTLDRPERFGLNPEFAHETMAGLNFVHAVAQALDAGKLFHIDLNDQRM
SRFDQDLRFGSENLKAAFFLVDLLESSGYQGPRHFDAHALRTEDEEGVWA
FARGCMRTYLILKERAEAFREDPEVKELLAAYYQEDPAALALLGPYSREK
AEALKRAELPLEAKRRRGYALERLDQLAVEYLLGVRG
> P0AFL3|PPIA_ECOLI Peptidyl -prolyl cis-trans isomerase A - Escherichia coli.
MFKSTLAAMAAVFALSALSPAAMAAKGDPHVLLTTSAGNIELELDKQKAP
VSVQNFVDYVNSGFYNNTTFHRVIPGFMIQGGGFTEQMQQKKPNPPIKNE
ADNGLRNTRGTIAMARTADKDSATSQFFINVADNAFLDHGQRDFGYAVFG
KVVKGMDVADKISQVPTHDVGPYQNVPSKPVVILSAKVLP
> P22320|HOXH_RALEH NAD-reducing hydrogenase hoxS subunit beta - Ralstonia eutropha (strain ATCC 17699 / H16 / DSM 428 / Stanier 337) (Cupriavidus necator (strain ATCC 17699 / H16 / DSM 428 / Stanier 337)).
MSRKLVIDPVTRIEGHGKVVVHLDDDNKVVDAKLHVVEFRGFEKFVQGHP
FWEAPMFLQRICGICFVSHHLCGAKALDDMVGVGLKSGIHVTPTAEKMRR
LGHYAQMLQSHTTAYFYLIVPEMLFGMDAPPAQRNVLGLIEANPDLVKRV
VMLRKWGQEVIKAVFGKKMHGINSVPGGVNNNLSIAERDRFLNGEEGLLS
VDQVIDYAQDGLRLFYDFHQKHRAQVDSFADVPALSMCLVGDDDNVDYYH
GRLRIIDDDKHIVREFDYHDYLDHFSEAVEEWSYMKFPYLKELGREQGSV
RVGPLGRMNVTKSLPTPLAQEALERFHAYTKGRTNNMTLHTNWARAIEIL
HAAEVVKELLHDPDLQKDQLVLTPPPNAWTGEGVGVVEAPRGTLLHHYRA
DERGNITFANLVVATTQNNQVMNRTVRSVAEDYLGGHGEITEGMMNAIEV
GIRAYDPCLSCATHALGQMPLVVSVFDAAGRLIDERAR
> P06608|ASPG_ERWCH L-asparaginase - Erwinia chrysanthemi.
MERWFKSLFVLVLFFVFTASAADKLPNIVILATGGTIAGSAATGTQTTGY
KAGALGVDTLINAVPEVKKLANVKGEQFSNMASENMTGDVVLKLSQRVNE
LLARDDVDGVVITHGTDTVEESAYFLHLTVKSDKPVVFVAAMRPATAISA
DGPMNLLEAVRVAGDKQSRGRGVMVVLNDRIGSARYITKTNASTLDTFKA
NEEGYLGVIIGNRIYYQNRIDKLHTTRSVFDVRGLTSLPKVDILYGYQDD
PEYLYDAAIQHGVKGIVYAGMGAGSVSVRGIAGMRKAMEKGVVVIRSTRT
GNGIVPPDEELPGLVSDSLNPAHARILLMLALTRTSDPKVIQEYFHTY
> P50500|IRO_THIFE Iron oxidase - Thiobacillus ferrooxidans (Acidithiobacillus ferrooxidans).
MSEKDKMITRRDALRNIAVVVGSVATTTMMGVGVADAGSMPKAAVQYQDT
PKGKDHCSVCAQFIAPHSCKVVAGNISPNGWCV AFVPKSA
> AAT_ECOLI P00509 ASPARTATE AMINOTRANSFERASE (EC 2.6.1.1) (T
MFENITAAPADPILGLADLFRADERPGKINLGIGVYKDETGKTPVLTSVK
KAEQYLLENETTKNYLGIDGIPEFGRCTQELLFGKGSALINDKRARTAQT
PGGTGALRVAADFLAKNTSVKRVWVSNPSWPNHKSVFNSAGLEVREYAYY
DAENHTLDFDALINSLNEAQAGDVVLFHGCCHNPTGIDPTLEQWQTLAQL
SVEKGWLPLFDFAYQGFARGLEEDAEGLRAFAAMHKELIVASSYSKNFGL
YNERVGACTLVAADSETVDRAFSQMKAAIRANYSNPPAHGASVVATILSN
DALRAIWEQELTDMRQRIQRMRQLFVNTLQEKGANRDFSFIIKQNGMFSF
SGLTKEQVLRLREEFGVYAVASGRVNVAGMTPDNMAPLCEAIVAVL
> P45678|PEB1_CAMJE Major cell -binding factor - Campylobacter jejuni.
MVFRKSLLKLAVFALGACVAFSNANAAEGKLESIKSKGQLIVGVKNDVPH
YALLDQATGEIKGFEVDVAKLLAKSILGDDKKIKLVAVNAKTRGPLLDNG
SVDAVIATFTITPERKRIYNFSEPYYQDAIGLLVLKEKKYKSLADMKGAN
IGVAQAATTKKAIGEAAKKIGIDVKFSEFPDYPSIKAALDAKRVDAFSVD
KSILLGYVDDKSEILPDSFEPQSYGIVTKKDDPAFAKYVDDFVKEHKNEI
DALAKKWGL
> Q07792|ESTE_VIBMI Arylesterase - Vibrio mimicus.
MIRLLSLVLFFCLSAASQASEKLLVLGDSLSAGYQMPIEKSWPSLLPDAL
LEHGQDVTVINGSISGDTTGNGLARLPQLLDQHTPDLVLIELGANDGLRG
FPPKVITSNLSKMISLIKDSGANVVMMQIRVPPNYGKRYSDMFYDIYPKL
AEHQQVQLMPFFLEHVITKPEWMMDDGLHPKPEAQPWIAEFVAQELVKHL

> P0A855|TOLB_ECOLI Protein tolB - Escherichia coli.
MKQALRVAFGFLILWASVLHAEVRIVIDSGVDSGRPIGVVPFQWAGPGAA
PEDIGGIVAADLRNSGKFNPLDRARLPQQPGSAQEVQPAAWSALGIDAVV
VGQVTPNPDGSYNVAYQLVDTGGAPGTVLAQNSYKVNKQWLRYAGHTASD
EVFEKLTGIKGAFRTRIAYVVQTNGGQFPYELRVSDYDGYNQFVVHRSPQ
PLMSPAWSPDGSKLAYVTFESGRSALVIQTLANGAVRQVASFPRHNGAPA

FSPDGSKLAFALSKTGSLNLYVMDLASGQIRQVTDGRSNNTEPTWFPDSQ
NLAFTSDQAGRPQVYKVNINGGAPQRITWEGSQNQDADVSSDGKFMVMVS
SNGGQQHIAKQDLATGGVQVLSSTFLDETPSLAPNGTMVIYSSSQGMGSV
LNLVSTDGRFKARLPATDGQVKFPAWSPYL
> P18012|IPAC_SHIFL Invasin ipaC - Shigella flexneri.
MEIQNTKPTQTLYTDISTKQTQSSSETQKSQNYQQIAAHIPLNVGKNPVL
TTTLNDDQLLKLSEQVQHDSEIIARLTDKKMKDLSEMSHTLTPENTLDIS
SLSSNAVSLIISVAVLLSALRTAETKLGSQLSLIAFDATKSAAENIVRQG
LAALSSSITGAVTQVGITGIGAKKTHSG ISDQKGALRKNLATAQSLEKEL
AGSKLGLNKQIDTNITSPQTNSSTKFLGKNKLAPDNISLSTEHKTSLSSP
DISLQDKIDTQRRTYELNTLSAQQKQNIGRATMETSAVAGNISTSGGRYA
SALEEEEQLISQASSKQAEEASQVSKEASQATNQLIQKLLNIIDSINQSK
NSAASQIAGNIRA
> ALKH_ERWCH P38448 4-HYDROXY-2-OXOGLUTARATE ALDOLASE (EC 4.1
MKNWKTSAEQILTAGPVVPVIVINKLEHAVPMAKALVAGGVRVLELTLRT
ECAVEAIRLIAQEVPDAIVGAGTVTNPQQLAEVTAAGAQFAISPGLTEPL
LKAATEGTIPLIPGISTVSELMLGMDYGLREFKFFPAEANGGVKALQAIA
GPFGKIRFCPTGGISLKNYRDYLALKSVLCVGGSWLVPADALESGDYDRI
TALAREAVAGATA
> ACKA_HAEIN P44406 ACETATE KINASE (EC 2.7 .2.1) (ACETOKINASE)
MSKLVLILNCGSSSLKFAILDPATGEEKLSGLAEAFFLPEARIKWKLNGE
KGNADLGAGAAHTEALNFIASNILNDELKNSIAAIGHRIVHGGEKYTQSV
IVTDEVVKGIEDAAQFAPLHNPAHLIGIREAFKAFPHLKDKNVVVFDTAF
HQTMPEEAFLYALPYSLYKEHGVRRYGAHGTSHYFVSREVAKYVGKPADQ
VNAIICHLGNGGSVSVVRNGQCIDTSMGLTPL EGLVMGTRCGDIDPAIVF
YLYKTLGMSMDQIEETLVKKSGLLGLTEVTSDCRYAEDNYDDESKPETRR
ALNVYSYRLAKYIGAYMAVLGDDHLDAIAFTGGIGENSAHVRELALNHLK
LFGIKIDNERNLATRFGKDGVITTDDSAFKAIVLPTNEELVIAQDTAKLC
F
> P0ABK9|NRFA_ECOLI Cytochrome c -552 - Escherichia coli.
MTRIKINARRIFSLLIPFFFFTSVH AEQTAAPAKPVTVEAKNETFAPQHP
DQYLSWKATSEQSERVDALAEDPRLVILWAGYPFSRDYNKPRGHAFAVTD
VRETLRTGAPKNAEDGPLPMACWSCKSPDVARLIQKDGEDGYFHGKWARG
GPEIVNNLGCADCHNTASPEFAKGKPELTLSRPYAARAMEAIGKPFEKAG
RFDQQSMVCGQCHVEYYFDGKNKAVKFPWDDGMKVENMEQYYDKIAFSDW
TNSLSKTPMLKAQHPEYETWTAGIHG KNNVTCIDCHMPKVQNAEGKLYTD
HKIGNPFDNFAQTCANCHTQDKAALQKVVAERKQSINDLKIKVEDQLVHA
HFEAKAALDAGATEAEMKPIQDDIRHAQWRWDLAIASHGIHMHAPEEGLR
MLGTAMDKAADARTKLARLLATKGITHEIQIPDISTKEKAQQAIGLNMEQ
IKAEKQDFIKTVIPQWEEQARKNGLLSQ
> P13811|ELBH_ECOLI Heat -labile enterotoxin B cha in - Escherichia coli.
MNKVKFYVLFTALLSSLCAHGAPQSITELCSEYHNTQIYTINDKILSYTE
SMAGKREMVIITFKSGATFQVEVPGSQHIDSQKKAIERMKDTLRITYLTE
TKIDKLCVWNNKTPNSIAAISMEN
> P80505|G3P2_SYNY3 Glyceraldehyde -3-phosphate dehydrogenase 2 -
Synechocystis sp. (strain PCC 6803).
MTRVAINGFGRIGRNFLRCWLGRTDSQLEVVGINDTSDPRTNAHLLRYDS
MLGKLDADISADENSITVNGKTIKCVSDRNPLNLPWAEWNVDLVIEATGV
FVTHEGATKHVQAGAKKVLITAPGKGPNIGTYVVGVNAHEYKHEEYEVIS
NASCTTNCLAPFGKVINDNFGIIKGTMTTTHSYTGDQRILDASHRDLRRA
RAAAVNIVPTSTGAAKAVALVIPELQGKLNGIALRVPTPNVSVVDLVVQV
EKNTIAEQVNGVLKEAANTSLKGVLEYTDLELVSSDFRGTDCSSTVDGSL
TMVMGGDMVKVIAWYDNEWGYSQRVVDLAEIVAKNWK
> P22318|HOXU_RALEH NAD -reducing hydrogenase hoxS subunit gamma - Ralstonia
eutropha (strain ATCC 17699 / H16 / DSM 428 / Stanier 337) (Cupriavidus
necator (strain ATCC 176 99 / H16 / DSM 428 / Stanier 337)).
MSIQITIDGKTLTTEEGRTLVDVAAENGVYIPTLCYLKDKPCLGTCRVCS
VKVNGNVAAACTVRVSKGLNVEVNDPELVDMRKALVEFLFAEGNHNCPSC
EKSGRCQLQAVGYEVDMMVSRFPYRFPVRVVDHASEKIWLERDRCIFCQR
CVEFIRDKASGRKIFSISHRGPESRIEIDAELANAMPPEQVKEAVAICPV
GTILEKRVGYDDPIGR RKYEIQSVRARALEGEDK
> NODB_BRASP P04675 CHITOOLIGOSACCHARIDE DEACETYLASE (EC 3.5.
MTEFIPLFAVRRNYGDVSGTRSVYLTFDDGPNPFCTPDVLDVLTQHRVPA

TFFVIGTYVANQPELIRRMVAEGHEVANHTMTHPDLSRCEPAEVHDEVLT
ASRAIRSACPQALPRHMRAPYGIWTQDVLATSAKAGLAAVHWSVDPRDWA
RPGVDRIVSSVLAAIRPGAIVL LHDGYPPGEERSCTDATSRDQTVRALSY
LIPALQRRGFEIHPLPQLH
> P08331|CN16_ECOLI 2',3' -cyclic-nucleotide 2'-phosphodiesterase -
Escherichia coli.
MIKFSATLLATLIAASVNAATVDLRIMETTDLHSNMMDFDYYKDTATEKF
GLVRTASLINDARNEVKNSVLVDNGDLIQGSPLADYMSAKGLKAGDIHPV
YKALNTLDYTVGTLGNHEFN YGLDYLKNALAGAKFPYVNANVIDARTKQP
MFTPYLIKDTEVVDKDGKKQTLKIGYIGVVPPQIMGWDKANLSGKVTVND
ITETVRKYVPEMREKGADVVVVLAHSGLSADPYKVMAENSVYYLSEIPGV
NAIMFGHAHAVFPGKDFADIEGADIAKGTLNGVPAVMPGMWGDHLGVVDL
QLSNDSGKWQVTQAKAEARPIYDIANKKSLAAEDSKLVETLKADHDATRQ
FVSKPIGKSADNMYSYLALVQ DDPTVQVVNNAQKAYVEHYIQGDPDLAKL
PVLSAAAPFKVGGRKNDPASYVEVEKGQLTFRNAADLYLYPNTLIVVKAS
GKEVKEWLECSAGQFNQIDPNSTKPQSLINWDGFRTYNFDVIDGVNYQID
VTQPARYDGECQMINANAERIKNLTFNGKPIDPNAMFLVATNNYRAYGGK
FAGTGDSHIAFASPDENRSVLAAWIADESKRAGEIHPAADNNWRLAPIAG
DKKLDIRFETSPSDKAAAFIKE KGQYPMNKVATDDIGFAIYQVDLSK
> P18187|PHNS_DESFR Periplasmic [NiFe] hydrogenase small subunit -
Desulfovibrio fructosovorans.
MNFSVGLGRMNAEKRLVQNGVSRRDFMKFCATVAAAMGMGPAFAPKVAEA
LTAKHRPSVVWLHNAECTGCTEAAIRTIKPYIDALILDTISLDYQETIMA
AAGETSEAALHQALEGKDGYYLVVEGGLPTI DGGQWGMVAGHPMIETTKK
AAAKAKGIICIRHLPHGGVQKAKPNPSQAKGVSEALGVKTINIPGCPPNP
INFVGAVVHVLTKGIPDLDENGRPKLFYGELVHDNCPRLPHFEASEFAPS
FDSEEAKKGFCLYELGCKGPVTYNNCPKVLFNQVNWPVQAGHPCLGCSEP
DFWDTMTPFYEQG
> P50320|PGKP_RALEH Phosphoglycerate kinase, plasmid - Ralstonia eutropha
(strain ATCC 17699 / H16 / DSM 428 / Stanier 337) (Cupriavidus necator
(strain ATCC 17699 / H16 / DSM 428 / Stanier 337)).
MMSLSHASVPHTNPTAPHTLAALLAAGGLAGKRVFIRADLNVPQDAAGDI
TDDTRIRASVPAIAACLQAGAAVMVTSHLGRPQEGAPDPRHSLAPVGRRL
SELLGRQVPLLSGWTEGGFQVPPGQV VLLENCRMNTGEKKNSDELAQKMA
ALCDVYVNDAFGTAHRAEATTHGIARYAPVACAGPLLAAEIDALGKALGQ
PARPLVAIVAGSKVSTKLTILKSLADKVDNLVVGGGIANTFMLAAGLKIG
KSLAEPDLLADARAIIDIMAARGASVPIPVDVVCAKEFSATAAAAVKDVR
DVADDDMILDIGPKTAAMLADQLKAAGTIVWNGPVGVFEFDQFGNGTRVL
AQAIAESKAFSIAGGGDTLAAIAKYGI ADRVGYISTGGGAFLEFLEGKKL
PALDVLEQRAAS
> P32676|PTFB3_ECOLI Fructose -like phosphotransferase enzyme IIB component
3 - Escherichia coli.
MAYLVAVTACVSGVAHTYMAAERLEKLCLLEKWGVSIETQGALGTENRLA
DEDIRRADVALLITDIELAGAERFEHCRYVQCSIYAFLREPQRVMSAVRK
VLSAPQQTHLILE
> P06621|CBPG_PSES6 Carboxypeptidase G2 - Pseudomonas sp. (strain RS -16).
MRPSIHRTAIAAVLATAFVAGTALAQKRDNVLFQAATDEQPAVIKTLEKL
VNIETGTGDAEGIAAAGNFLEAELKNLGFTVTRSKSAGLVVGDNIVGKIK
GRGGKNLLLMSHMDTVYLKGILAKAPFRVEGDKAYGPGIADDKGGNAVIL
HTLKLLKEYGVRDYGTITVLFNTDEEKGSFGSRDL IQEEAKLADYVLSFE
PTSAGDEKLSLGTSGIAYVQVNITGKASHAGAAPELGVNALVEASDLVLR
TMNIDDKAKNLRFNWTIAKAGNVSNIIPASATLNADVRYARNEDFDAAMK
TLEERAQQKKLPEADVKVIVTRGRPAFNAGEGGKKLVDKAVAYYKEAGGT
LGVEERTGGGTDAAYAALSGKPVIESLGLPGFGYHSDKAEYVDISAIPRR
LYMAARLIMDLGAGK
> P37329|MODA_ECOLI Molybdate-binding periplasmic protein - Escherichia
coli.
MARKWLNLFAGAALSFAVAGNALADEGKITVFAAASLTNAMQDIATQFKK
EKGVDVVSSFASSSTLARQIEAGAPADLFISADQKWMDYAVDKKAIDTAT
RQTLLGNSLVVVAPKASVQKDFTIDSKTNWTSLLNGGRLAVGDPEHVPAG
IYAKEALQKLGAWDTLSPKLAPAEDVRGALALVERNEAPLGIVYG SDAVA
SKGVKVVATFPEDSHKKVEYPVAVVEGHNNATVKAFYDYLKGPQAAEIFK
RYGFTIK

> P0A1V8|BLO2_SALTY Beta -lactamase OXA -2 - Salmonella typhimurium.
MAIRIFAILFSIFSLATFAHAQEGTLERSDWRKFFSEFQAKGTIVVADER
QADRAMLVFDPVRSKKRYSPASTFKIPHTLFALDAGAVRDEFQIFRWDGV
NRGFAGHNQDQDLRSAMRNSTV WVYELFAKEIGDDKARRYLKKIDYGNAD
PSTSNGDYWIEGSLAISAQEQIAFLRKLYRNELPFRVEHQRLVKDLMIVE
AGRNWILRAKTGWEGRMGWWVGWVEWPTGSVFFALNIDTPNRMDDLFKRE
AIVRAILRSIEALPPNPAVNSDAAR
> HEMN_PSEAE P77915 OXYGEN -INDEPENDENT COPROPORPHYRINOGEN III
MLDTIRWDADLIRRYDLSGPRYTSYPTAVQFHEGIGP FDQLHALRDSRKA
GHPLSLYVHIPFCANICYYCACNKVITKDRGRSAPYLARLVREIEIVSRH
LSRAQVVEQLHFGGGTPTFLSPGQLRELMSQLRTHLNLLDDDSGDYGIEI
DPREADWSTMGLLRELGFNRVSLGVQDFDMEVQKAVNRMQTPEETRTIVE
AARTLQYRSINLDLIYGLPKQTPDSFARTVDEVIALQPDRLSVFNYAHLP
ERFMPQRRINADDLPSPGQKLEMLQRTTEQLAAAGYRY IGMDHFALPDDE
LASAQEDGTLQRNFQGYTTHGHCDLVGLGVSAISQIGDLYSQNSSDINDY
QTSLDNGQLAIRRGLHCNSDDRVRRAVIQQLICHFELAFEDIETEFGIDF
RSYFAELWPDLERFAADGLIRPDAKGIDITSSGRLLVRSICMLFDRYLPS
LNRQRFSRVI
> ADHI_RHOSH P72324 ALCOHOL DEHYDROGENASE CLASS III (EC 1.1.1
MRTRAAVAVEAGKPLEI MEVNLEGPKAGEVMVEIKATGICHTDEFTLSGA
DPEGMFPAILGHEGAGVVVEVGPGVTSVKPGDHVIPLYTPECRQCPSCLS
QKTNLCTAIRGTQGQGLMPDGTSRFSMLDGTPILHYMGCSTFSNYTVLPE
IAVAKVRPDAPFDKICYIGCGVTTGIGAVINTAKVEIGAKAVVFGLGGIG
LNVIQGLKLAGADMIIGVDLNNAKKEWGERFGMTHFVNPSEIDGDVVAHL
VNMTKTPFDQIGGADYTF DCTGNVKVMRQALEACHRGWGQSIVIGVAPAG
AEIQTRPFQLVTGRVWKGSAFGGARGRTDVPKIVDWYMEGKIQIDPMITH
ILSLEEINKGFDLMHAGESIRSVVVF
> P00446|SODC_PHOLE Superoxide dismutase [Cu -Zn] - Photobacterium leiognathi.
MNKAKTLLFTALAFGLSHQALAQDLTVKMTDLQTGKPVGTIELSQNKYGV
VFTPELADLTPGMHGF HIHQNGSCASSEKDGKVVLGGAAGGHYDPEHTNK
HGFPWTDDNHKGDLPALFVSANGLATNPVLAPRLTLKELKGHAIMIHAGG
DNHSDMPKALGGGGARVACGVIQ
> P07103|GUNZ_DICD3 Endoglucanase Z - Dickeya dadantii (strain 3937) (Erwinia chrysanthemi (strain 3937)).
MPLSYLDKNPVIDSKKHALRKKLFLSCAYFGLSLACLSS NAWASVEPLSV
NGNKIYAGEKAKSFAGNSLFWSNNGWGGEKFYTADTVASLKKDWKSSIVR
AAMGVQESGGYLQDPAGNKAKVERVVDAAIANDMYAIIGWHSHSAENNRS
EAIRFFQEMARKYGNKPNVIYEIYNEPLQVSWSNTIKPYAEAVISAIRAI
DPDNLIIVGTPSWSQNVDEASRDPINAKNIAYTLHFYAGTHGESLRNKAR
QALNNGIALFVTEWGTVNADGNGGVNQTETDAWVTFMRDN NISNANWALN
DKNEGASTYYPDSKNLTESGKKVKSIIQSWPYKAGSAASATTDPSTDTTT
DTTVDEPTTTDTPATADCANANVYPNWVSKDWAGGQPTHNEAGQSIVYKG
NLYTANWYTASVPGSDSSWTQVGSCN
> P0A9H7|CFA_ECOLI Cyclopropane -fatty-acyl-phospholipid synthase - Escherichia coli.
MSSSCIEEVSVPDDNWYRIANELLSRAGIAI NGSAPADIRVKNPDFFKRV
LQEGSLGLGESYMDGWWECDRLDMFFSKVLRAGLENQLPHHFKDTLRIAG
ARLFNLQSKKRAWIVGKEHYDLGNDLFSRMLDPFMQYSCAYWKDADNLES
AQQAKLKMICEKLQLKPGMRVLDIGCGWGGLAHYMASNYDVSVVGVTISA
EQQKMAQERCEGLDVTILLQDYRDLNDQFDRIVSVGMFEHVGPKNYDTYF
AVVDRNLKPEGIFLLHTIGSKKTDLNVDPWIN KYIFPNGCLPSVRQIAQS
SEPHFVMEDWHNFGADYDTTLMAWYERFLAAWPEIADNYSERFKRMFTYY
LNACAGAFRARDIQLWQVVFSRGVENGLRVAR
> P23031|XYNC_PSEFL Alpha -L-arabinofuranosidase C - Pseudomonas fluorescens.
MINHNKTPNILAKVFKRTCGLVSTGAALAILSQAASAACTYTIDSEWSTG
FTANITLKNDTGAAINNWNVNWQYS SNRMTSGWNANFSGTNPYNATNMSW
NGSIAPGQSISFGLQGEKNGSTAERPTVTGAACNSATTSSVASSSSTPTT
SSSSASSVASALLLQEAQAGFCRVDGTIDNNHTGFTGSGFANTNNAQGAA
VVWAIDATSSGRRTLTIRYANGGTANRNGSLVINGGSNGNYTVSLPTTGA
WTTWQTATIDVDLVQGNNIVQLSATTAEGLPNIDSLSVVGGTVRAGNCGS
VSSSSSVQSSSSSSSTPSQTCELKAP LRWTSTGPLISPKNPGWISIKDPS
IVKYNDTYHVYATYYDTAYRSMYTSFTDWNTAQQAPHISMNGSRVGNTVA

PQVFYFRPHNKWYLITQWAGAYATTDDIRNPNWSAKQKLLQGEPNGALDF
WVICNDTHCYLYFSRDDGVLYVSKTTLANFPNFSGYSIVMEDHRGNGNSY
LFEAANVYKLDGQNRYLLMVEAYISGRAFSAPGQRPAWMAHGPLADTEAN
PFAGMMFCFTMASSLKVYTCY
> P16465|HLYB_PROMI Hemolysin activator protein - Proteus mirabilis.
MKKKVVLLTLLSCFSTSGLSANETGNLGSISESRRALQDSQREINQLIEQ
NRYQQLQEKAVNISPTPTLITESEHCLPIKGVYIQGITLLTEKDLNSLSP
LPDQCIKSADINRLVKELTQRYLQHGYITARIQFLRPNQHGELGLYAIEG
FVERIEGGDRGVNTTLLFPRIKGQPLKLATLDQGLDQAN RLQSNKVTVDI
LPGTELGGSVIKLSNQRKSPWHLNIASDNYGQKNSGRWLIRTNASLDSPL
GLSDFVSLNANITTDNPNTRFNRAYTLLYSIPYGGFTFSSFGSYSEYQFH
QKLQTRTVNLYGDTTQVGIRGDYAFSRSQKQIDTLNIQVTHKRIRNYFSQ
IRLDLSSPKLTTIELGINHLQIIPNGVLSTNLSVEKAVGWFGAEETPYIA
NGNGNDYRFTKVKLFTNWYQRFSLWHSTFLFNSTFLGQYS HDTLPGVEWL
SLTDKNAIRGFDQSTLSGDNGGYLRNTLSYPYRLNHFSITPRIGVDIGQV
KQHGNYKGWQGGYGLSSGLNIQYQQAQLDLEVAKGELLYHQTNSNKTKDP
TQLLVKFSYLF
> ADI_ECOLI P28629 BIODEGRADATIVE ARGININE DECARBOXYLASE (EC
MKVLIVESEFLHQDTWVGNAVERLADALSQQNVTVIKSTSFDDGFAILSS
NEAIDCLMFSYQMEHPDE HQNVRQLIGKLHERQQNVPVFLLGDREKALAA
MDRDLLELVDEFAWILEDTADFIAGRAVAAMTRYRQQLLPPLFSALMKYS
DIHEYSWAAPGHQGGVGFTKTPAGRFYHDYYGENLFRTDMGIERTSLGSL
LDHTGAFGESEKYAARVFGADRSWSVVVGTSGSNRTIMQACMTDNDVVVV
DRNCHKSIEQGLMLTGAKPVYMVPSRNRYGIIGPIYPQEMQPETLQKKIS
ESPLTKDKAGQKPSYCVVT NCTYDGVCYNAKEAQDLLEKTSDRLHFDEAW
YGYARFNPIYADHYAMRGEPGDHNGPTVFATHSTHKLLNALSQASYIHVR
EGRGAINFSRFNQAYMMHATTSPLYAICASNDVAVSMMDGNSGLSLTQEV
IDEAVDFRQAMARLYKEFTADGSWFFKPWNKEVVTDPQTGKTYDFADAPT
KLLTTVQDCWVMHPGESWHGFKDIPDNWSMLDPIKVSILAPGMGEDGELE
ETGVPAALVTAWLGRHGIVP TRTTDFQIMFLFSMGVTRGKWGTLVNTLCS
FKRHYDANTPLAQVMPELVEQYPDTYANMGIHDLGDTMFAWLKENNPGAR
LNEAYSGLPVAEVTPREAYNAIVDNNVELVSIENLPGRIAANSVIPYPPG
IPMLLSGENFGDKNSPQVSYLRSLQSWDHHFPGFEHETEGTEIIDGIYHV
MCVKA
> CILG_HAEIN P44461 CITRATE LYASE ACYL CARRIER PROTEIN (CITRA
MKITKVAVAGTLESSDVQVRVQPFDSLDIEINSSVAKQFGEQIEATVREV
LAKLGITAAQVIVEDKGALDCVLQARVKAAAMRATDEAINWEAVL
> LPCA_ECOLI P51001 PHOSPHOHEPTOSE ISOMERASE (EC 5. -.-.-). -
MYQDLIRNELNEAAETLANFLKDDANIHAIQRAAVLLADSFKAGGKVLSC
GNGGSHCDAMHFAEELTGRYRENRPGYPAIAISDVSHISCVGNDFGFNDI
FSRYVEAVGREGDVLLGISTSGNSANVIKAIAAAREKGMKVITLTGKDGG
KMAGTADIEIRVPHFGYADRIQEIHIKVIHILIQLIEKEMVK
> P02911|ARGT_SALTY Lysine -arginine-ornithine-binding periplasmic protein -
Salmonella typhimurium.
MKKTVLALSLLIGLGATAASYAALPQTVRIGTDTTYAPFSSKDAKGEFIG
FDIDLGNEMC KRMQVKCTWVASDFDALIPSLKAKKIDAIISSLSITDKRQ
QEIAFSDKLYAADSRLIAAKGSPIQPTLESLKGKHVGVLQGSTQEAYAND
NWRTKGVDVVAYANQDLIYSDLTAGRLDAALQDEVAASEGFLKQPAGKEY
AFAGPSVKDKKYFGDGTGVGLRKDDTELKAAFDKALTELRQDGTYDKMAK
KYFDFNVYGD
> P22608|PILB_PSEAE Type 4 fimbrial assembly protei n pilB - Pseudomonas
aeruginosa.
MNDSIQLSGLSRQLVQANLLDEKTAVQAQAQAQRNKLSLVTHLVQSKLVS
GLALAELSAEQFGIAYCDLNSLDKESFPRDAISEKLVRQHRVIPLWRRGN
KLFVGISDPANHQAINDVQFSTGLTTEAILVEDDKLGLAIDKLFESATDG
LAGLDDVDLEGLDIGSADKSTQEDASAEADDAPVVRFVNKMLLDAIKGGS
SDLHFEPYEKIYRVRFRTD GMLHEVAKPPIQLASRISARLKVMAGLDISE
RRKPQDGRIKMRVSKTKSIDFRVNTLPTLWGEKIVMRILDSSSAQMGIDA
LGYEEDQKELYLAALKQPQGMILVTGPTGSGKTVSLYTGLNILNTTDINI
STAEDPVEINLEGINQVNVNPRQGMDFSQALRAFLRQDPDVIMVGEIRDL
ETAEIAIKAAQTGHMVMSTLHTNSAAETLTRLLNMGVPAFNLATSVNLII
AQRLARKLCSHCKKEHEVPR ETLLHEGFPEDKIGTFKLYSPVGCDHCKNG
YKGRVGIYEVVKNTPALQRIIMEEGNSIEIAEQARKEGFNDLRTSGLLKA

MQGITSLEEVNRVTKD
> P08038|DRNE_VIBCH Extracellular deoxyribonuclease - Vibrio cholerae.
MMIFRFVTTLAASLPLLTFAAPISFSHAKNEAVKIYRDHPVSFYCGCEIR
WQGKKGIPDLESCGYQVRKNENRASRIEWEHVVPA WQFGHQLQCWQQGGR
KNCTRTSPEFNQMEADLHNLTPAIGEVNGDRSNFSFSQWNGVDGVTYGQC
EMQVNFKERTAMPPERARGAIARTYLYMSEQYGLRLSKAQSQLMQAWNNQ
YPVSEWECVRDQRIEKVQGNSNRFVREQCPN
> P13431|SFAH_ECOL5 S-fimbrial adhesin protein sfaH - Escherichia coli O6:K15:H31 (strain 536 / UPEC).
MAYSQPSFALLCRNNQTGQEFNSGDTSFRVNVSPVVQYDKSISVLDLSQL
VSCQNEDSTGQNYDYLKILKGSGFSPALDTKTYGRLDFTSRPTGYARQLP
LQFDLQVTEAFYQYGVWKPFPAKLYLYPEPGVFGKVINNGDLLATLYVNK
FSTKGQEAGERNFTWRFYATNDVHIQTGTCRVSSNNVKVDLPSYPGGPVT
VPLTVRCDQTQSVSYTLSGPVTGSGNTVFANTAASGAGGVGVQLSDKAGP
VPAGQPRSLGQVGSSPVSLGLKASYALTGQASLTPGAVQSVINVTFSYN
> P14774|CYCL_METEX Cytochrome c-L - Methylobacterium extorquens (Protomonas extorquens).
MMNRVKIGTALLGLTLAGIALPALAQPQSGPQTGVVFRNTVTGEALDVSQ
GKEGGRDTPAVKKFLETGENLYIDDKSCLRNGESLFATSCSGCHGHLAEG
KLGPGLNDNYWTYPSNTT DVGLFATIFGGANGMMGPHNENLTPDEMLQTI
AWIRHLYTGPKQDAVWLNDEQKKAYTPYKQGEVIPKDAKGQCKPLDE
> P28784|CPG1_PORGI Gingipain R1 - Porphyromonas gingivalis (Bacteroides gingivalis).
MKNLNKFVSIALCSSLLGGMAFAQQTELGRNPNVRLLESTQQSVTKVQFR
MDNLKFTEVQTPKGMAQVPTYTEGVNLSEKGMPTLPIL SRSLAVSDTREM
KVEVVSSKFIEKKNVLIAPSKGMIMRNEDPKKIPYVYGKSYSQNKFFPGE
IATLDDPFILRDVRGQVVNFAPLQYNPVTKTLRIYTEITVAVSETSEQGK
NILNKKGTFAGFEDTYKRMFMNYEPGRYTPVEEKQNGRMIVIVAKKYEGD
IKDFVDWKNQRGLRTEVKVAEDIASPVTANAIQQFVKQEYEKEGNDLTYV
LLVGDHKDIPAKITPGIKSDQVYGQIVGNDHYNEVFIGR FSCESKEDLKT
QIDRTIHYERNITTEDKWLGQALCIASAEGGPSADNGESDIQHENVIANL
LTQYGYTKIIKCYDPGVTPKNIIDAFNGGISLVNYTGHGSETAWGTSHFG
TTHVKQLTNSNQLPFIFDVACVNGDFLFSMPCFAEALMRAQKDGKPTGTV
AIIASTINQSWASPMRGQDEMNEILCEKHPNNIKRTFGGVTMNGMFAMVE
KYKKDGEKMLDTWTVFGDPSLLVRTLVPTKMQVTAPAQIN LTDASVNVSC
DYNGAIATISANGKMFGSAVVENGTATINLTGLTNESTLTLTVVGYNKET
VIKTINTNGEPNPYQPVSNLTATTQGQKVTLKWDAPSTKTNATTNTARSV
DGIRELVLLSVSDAPELLRSGQAEIVLEAHDVWNDGSGYQILLDADHDQY
GQVIPSDTHTLWPNCSVPANLFAPFEYTVPENADPSCSPTNMIMDGTASV
NIPAGTYDFAIAAPQANAKIWIAGQGPTKEDDYVFEAGKKY HFLMKKMGS
GDGTELTISEGGGSDYTYTVYRDGTKIKEGLTETTYRDAGMSAQSHEYCV
EVKYAAGVSPKVCVDYIPDGVADVTAQKPYTLTVVGKTITVTCQGEAMIY
DMNGRRLAAGRNTVVYTAQGGYYAVMVVVDGKSYVEKLAVK
> P37387|XYLF_ECOLI D-xylose-binding periplasmic protein - Escherichia coli.
MKIKNILLTLCTSLLLTNVAAHAKE VKIGMAIDDLRLERWQKDRDIFVKK
AESLGAKVFVQSANGNEETQMSQIENMINRGVDVLVIIPYNGQVLSNVVK
EAKQEGIKVLAYDRMINDADIDFYISFDNEKVGELQAKALVDIVPQGNYF
LMGGSPVDNNAKLFRAGQMKVLKPYVDSGKIKVVGDQWVDGWLPENALKI
MENALTANNNKIDAVVASNDATAGGAIQALSAQGLSGKVAISGQDADLAG
IKRIAAGTQTMTVYKPITLLANTAAE IAVELGNGQEPKADTTLNNGLKDV
PSRLLTPIDVNKNNIKDTVIKDGFHKESEL
> P36431|SYP_CHLTR Prolyl-tRNA synthetase - Chlamydia trachomatis.
MRMSLLFYRTSKNANKEASVLSYELLQKAGYLFKTSKGIYSYTPLFQRVI
LKMTEIIREELNAIGGQEVCLPLLQPAELWEKTGRWKAFLSEKLLYVLKD
RENKAMCLAPTHEEVVSEFVAQWLTGREQLP IHLYQIGTKFRDEIRPRFG
LMRAKEFLMEDSYTFSDSPEQMEEQYAKLRLAYQRIFDRLNLKYVIVAAD
GGKIGKGKSEEFHVLCSLGEDTICVSGSYGANVEAAQAIPPSYVYDSNLL
PVEEVATPNIRTIEDLEVFFNTPKHKILKTLVVKTCQKDSEKFFAICIRG
DRQINLTKVASFLQVDDCELASEEEILKHLHVEKGFIGPLYCPIPCYADE
TTRPMTNFICANNQKDVHCKHVNWGRDIPLPA FGDFLLAEAGDLCPQNGG
APYEIFQGVEVAHIFNLGTRYTESFSVGFQDKNGDKQLCWMGTYGIGVGR
TLAACIEQLADNKGLVWPLAVAPFSITILYNGGDTEGEATALQLYQSLNT
EGFEPLLDDRNERLGFKLKDSDLLGIPYKLIIGKSFQSTGLLEIESRSGE

KCNVSPENLLDWCSKNLPCHTRKIPPLREQN
> P49250|AMO_KLEAE Copper amine oxidase - Klebsiella aerogenes.
MANGLKFSPRKTALALAVAVVCAWQSPVFAHGSEAHMVPLDKTLQEFGAD
VQWDDYAQMFTLIKDGAYVKVKPGAKTAIVNGKSLDLPVPVVMKEGKAWV
SDTFINDVFQSGLDQTFQVEKRPHPLNSLSAAEISKAVTIVKAAPEFQPN
TRFTEISLHEPDKAAVWAFALQGTPVDAPRTADVVMLDGKHVIEAVVDLQ
NKKILSWTPIKGAHGMVLLDDFVSVQNIINTSSEFAEVLK KHGITDPGKV
VTTPLTVGFFDGKDGLQQDARLLKVVSYLDTGDGNYWAHPIENLVAVVDL
EAKKIIKIEEGPVIPVPMEPRPYDGRDRNAPAVKPLEITEPEGKNYTITG
DTIHWQNWDFHLRLNSRVGPILSTVTYNDNGTKRQVMYEGSLGGMIVPYG
DPDVGWYFKAYLDSGDYGMGTLTSPIVRGKDAPSNAVLLDETIADYTGKP
TTIPGAVAIFERYAGPEYKHLEMGKPNVSTERRELVVRWIS TVGNYDYIF
DWVFHDNGTIGIDAGATGIEAVKGVLAKTMHDPSAKEDTRYGTLIDHNIV
GTTHQHIYNFRLDLDVDGENNTLVAMDPEVKPNTAGGPRTSTMQVNQYTI
DSEQKAAQKFDPGTIRLLSNTSKENRMGNPVSYQIIPYAGGTHPAATGAK
FAPDEWIYHRLSFMDKQLWVTRYHPTERYPEGKYPNRSAHDTGLGQYAKD
DESLTNHDDVVWITTGTTHVARAEEWPIMPTEWALALLKPWN FFDETPTL
GEKKK
> ASG1_ECOLI P18840 L-ASPARAGINASE I (EC 3.5.1.1) (L-ASPARAGI
MQKKSIYVAYTGGTIGMQRSEQGYIPVSGHLQRQLALMPEFHRPEMPDFT
IHEYTPLMDSSDMTPEDWQHIAEDIKAHYDDYDGFVILHGTDTMAYTASA
LSFMLENLGKPVIVTGSQIPLAELRSDGQINLLNALYVAANYPINEVTLF
FNNRLYRGNRTTKAHADGFDAFASPN LPPLLEAGIHIRRLNTPPAPHGEG
ELIVHPITPQPIGVVTIYPGISADVVRNFLRQPVKALILRSYGVGNAPQN
KAFLQELQEASDRGIVVVNLTQCMSGKVNMGGYATGNALAHAGVIGGADM
TVEATLTKLHYLLSQELDTETIRKAMSQNLRGELTPDD
> Q59967|SRPH_SYNP7 Serine acetyltransferase, plasmid - Synechococcus sp. (strain PCC 7942) (Anacystis nidulans R2).
MSLSPRSDRTEIRRSWGLDSIVSALSQASTDPLPHHLLSDQFYPLPSRES
LGLILHGLRSVLFPRHFGDPELSVETTHYFIGNTLDKTLNLLNEQIRREL
WLQHVTQGTPEATPAVLSQHASELTQAFAQALPEIKRLLDSDVNAAYLGD
PAAQSISEILFCYPGITAITFHRLAHRLYQLGLPLLARITAEVSHSETGI
DIHPGAAIGGSFFIDHGTGVVIGET CVIGDRVRIYQAVTLGAKSFPRDET
GALIKGQARHPVIEDDVVIYAGATLLGRITVGRGSTIGGNVWLTRSVPAG
SFISQAQIRSDNFESGGGI
> P54354|LEU3_BACFR 3-isopropylmalate dehydrogenase - Bacteroides fragilis.
MDFKIAVLAGDGIGPEISVQGVEVMSAVCEKFGHKVNYEYAICGADAIDK
VGDPFPEETYRVCKNADAVLFSAVGDPKFDND PTAKVRPEQGLLAMRKKL
GLFANIRPVQTFKCLVHKSPLRAELVEGADFLCIRELTGGMYFGEKYQDN
DKAYDTNMYTRPEIERILKVGFEYAMKRRKHLTVVDKANVLASSRLWRQI
AQEMAPQYPEVTTDYMFVDNAAMKMIQEPKFFDVMVTENTFGDILTDEGS
VISGSMGLLPSASTGESTPVFEPIHGSWPQAKGLNIANPLAQILSVAMLF
EYFDCKAEGALIRKAVDASLDANVRTPEIQVEG GEKFGTKEVGAWIVDYI
RKA
> P13041|VRPR_SALTY Virulence genes transcriptional activator - Salmonella typhimurium.
MDFLINKKLKIFITLMETGSFSIATSVLYITRTPLSRVISDLERELKQRL
FIRKNGTLIPTEFAQTIYRKVKSHYIFLHALEQEIGPTGKTKQLEIIFDE
IYPGSLKNLIISALTISGQKTNIMGRAVNSQIIEELCQTNNCIV ISARNY
FHRESLVCRTSVEGGVMLFIPKKFFLCGKPDINRLAGTPVLFHEGAKNFN
LDTIYHFFEQTLGITNPAFSFDNVDLFSSLYRLQQGLAMLLIPVRVCRAL
GLSTDHALHIKGVALCTSLYYPTKKRETPDYRKAIKLIQQELKQSTF
> DAPD_ECOLI P03948 2,3,4,5-TETRAHYDROPYRIDINE-2-CARBOXYLATE
MQQLQNIIETAFERRAEITPANADTVTREAVNQVIAL LDSGALRVAEKID
GQWVTHQWLKKAVLLSFRINDNQVIEGAESRYFDKVPMKFADYDEARFQK
EGFRVVPPAAVRQGAFIARNTVLMPSYVNIGAYVDEGTMVDTWATVGSCA
QIGKNVHLSGGVGIGGVLEPLQANPTIIEDNCFIGARSEVVEGVIVEEGS
VISMGVYIGQSTRIYDRETGEIHYGRVPAGSVVVSGNLPSKDGKYSLYCA
VIVKKVDAKTRGKVGINELLRTID
> P73162|ACKA_SYNY3 Acetate kinase - Synechocystis sp. (strain PCC 6803).
MKFLILNAGSSSQKSCLYELTGDRLPETIPEPLWEAFIDWTVLANQGRLT
VETAGQKQVIILETGDRQQGIARMLDTLVTGDDAVLKSLAEIDLVGHRVV
HGGTDHAEATLITPEVQQAIADLIPLAPAHNPAHLEGIEAISALLVLGEV

PQIAVFDTAFHRTIPTPAAEYPIPQAWTNLGIRRYGFHGTSH KYCAQKTA
EILGKPLADLKLITCHIGNGASLTAIKNGVSIDTTMGFTPLEGLMMGARS
GSIDPAILLFLQETQGLTPAEINTTLNKKSGLLGVSGLSADLRTILQAKA
EGNEQAQLAYVMYIHRFRSCLGQMIASLEGLDTLVFTAGVGENAATVRAD
VCQAFEFLGLKLDPELNNRSPRDTVISHSDSLVTVLIVHTEEDWAIAQDC
WHWWHSQGQRKQS
> P43922|PT1_HAEIN Phosphoeno lpyruvate-protein phosphotransferase - Haemophilus influenzae.
MISGILASPGIAFGKALVLKEEKIVLDTQKITDDQIDAEVARFYEGRNAA
VEQLNSIRERALISLGEEKAAIFEGHLMILEDEELEEEILDYLRSNKVNA
GVAASKILDQQVTMLSEIDDEYLKERAGDIRDIANRLVKNILGMYIVDLG
DIQEESILVAYDLTPSETAQLNLEKVLGVVTDIGGRTSHT SIMARSLELP
AIVGTNKVTKLVNTGDYLILDAINNQVYINPTASQIDELKALEAKISEEK
AELAKLKDLPAITLDGHKVDVVANIGTIRDCDGAERNGAEGIGLYRTEFL
FMDREQLPTEEEQFIAYKQVVEAMNGRLTVIRTMDIGGDKELSYLDLPKE
MNPFLGWRAIRIALDRREILNAQLRAVLRASAFGKLAVMFPMIISVEEIR
ELKAVIETLKAELREEGRLFDNNIQVGVMVETPSAAVNAKF LAKEVDFFS
IGTNDLTQYTLAVDRGNEFISHLYNPMHPSVLGLIKQVIDASHAEGKWTG
MCGELAGDERATLLLLGMGLDEFSMSAISVPRIKKLIRNVNFQDAKVLAD
TALQKPTAAEIDQLIEEFLLENSLN
> P18958|INH_ERWCH Proteinase inhibitor - Erwinia chrysanthemi.
MKQLIIATLLSALSGGCMASSLRLPSAAELSGQWVLSGAEQHCDIRLNTD
VLDGTTWKLAGDTACLQKLLPEAPVGWRPTPDGLTLTQADGSAVAFFSRN
RDRYEHKLVDGSVRTLKKKA
> P12903|FM12_KLEPN Fimbrial subunit type 1 - Klebsiella pneumoniae.
MKIKTLAMIVVSALALSSTAALADTTTVNGGTVHFKGEVVNAACAVDAGS
IDQTVQLGQVRSAKLATAGSTSSAVGFNIQLDDCDTTVATKASVAFAGTA
IDSSNTTVLALQNSAA GSATNVGVQILDNTGTPLALNGATFSAATTLNDD
PNIIPFQARYYATGAATAGIANADATFKVQYE
> P19369|FMFL_AERHY Flexible pilin - Aeromonas hydrophila.
MPNFFRNGCIALVGSVAAMGAAHAEGGIAEAAGKALDSAQSDVTITAPKV
MMVVATVVGVGILINMMRKA
> P23847|DPPA_ECOLI Periplasmic dipeptide transport prote in - Escherichia coli.
MRISLKKSGMLKLGLSLVAMTVAASVQAKTLVYCSEGSPEGFNPQLFTSG
TTYDASSVPLYNRLVEFKIGTTEVIPGLAEKWEVSEDGKTYTFHLRKGVK
WHDNKEFKPTRELNADDVVFSFDRQKNAQNPYHKVSGGSYEYFEGMGLPE
LISEVKKVDDNTVQFVLTRPEAPFLADLAMDFASILSKEYADAMMKAGTP
EKLDLNPIGTGPFQLQQYQKDSRIRYKAF DGYWGTKPQIDTLVFSITPDA
SVRYAKLQKNECQVMPYPNPADIARMKQDKSINLMEMPGLNVGYLSYNVQ
KKPLDDVKVRQALTYAVNKDAIIKAVYQGAGVSAKNLIPPTMWGYNDDVQ
DYTYDPEKAKALLKEAGLEKGFSIDLWAMPVQRPYNPNARRMAEMIQADW
AKVGVQAKIVTYEWGEYLKRAKDGEHQTVMMGWTGDNGDPDNFFATLFSC
AASEQGSNYSKWCYKPFEDLIQPARATDDH NKRVELYKQAQVVMHDQAPA
LIIAHSTVFEPVRKEVKGYVVDPLGKHHFENVSIE
> P44708|GLMS_HAEIN Glucosamine --fructose-6-phosphate aminotransferase [isomerizing] - Haemophilus influenzae.
MCGIVGAVAQRDVAEILINGLHRLEYRGYDSAGVAVINKQNELQRIRCLG
KVKALDEAVSEKPLIGGTGIAHTRWATHGEPSETNAH PHSSGTFAVVHNG
IIENHEELRELLKSRGYVFLSQTDTEVIAHLVEWEMRTTDSLLDAVKKAV
KQLTGAYGMVVMDSRHPEHLVAARSGSPLVIGLGIGENFLASDQLALLSV
TRRFIFLEEGDIAEITRRTVDIYDTHGNKAKREIHESNLENDAAEKGKFR
HFMQKEIYEQPTALINTMEGRINHENVIVDSIGNGAKGILEKVEHIQIVA
CGTSYNAGMVARYWFESLAGVSCDVEIASEFRYRKFVT RPNSLLITLSQS
GETADTLAALRLAKEKGYMAALTICNVAGSSLVRESDLAFMTRAGVEVGV
ASTKAFTTQLAALLMLVTALGKVKGHISVEKEREIIKAMQSLPAEIEKAL
AFDTEIEALAEDFAEKHHALFLGRGAFYPIAVEASLKLKEISYIHAEAYA
AGELKHGPLALIDADMPVIVVAPNNELLEKVKSNIEEVRARGGQLYVFAD
KEAGFTPSEGMKIITMPKVNDIVAPIFYTIPMQLLSYYV ALIKGTDVDQP
RNLAKSVTVE
> P06970|FAED_ECOLI Outer membrane usher protein faeD - Escherichia coli.
MKKYVTTKSVQPVAFRLTTLSLVMSAVLGSASVIAGEKLDMSFIQGGGGV
NPEVWAALNGSYAPGRYLVDLSLNGKEAGKQILDVTPQDSNELCLTEAWL

TKAGVYVSADYFREGYDATRQCYVLTKAPSVKVDFDVSTQSLALSIPQKG
LVKMPENVDWDYGTSAFRVNYNANANTGRNNTSAFGSADLKANIGHWVVS
SSATASGGDSGDNSTTINMFTATRAIRALSADLAVGKTSTGDSLLGSTGT
YGVSLSRNNSMKPGNLGYTPVFSGIANGPSRVTLTQNGRLLHSEMVPAGP
FSITDVPLYTSGDVTMKITGEDGRDEVQNFPLSVMAGQLSPGQHEFSVAA
GLPDDDSDLKGGVFAASYGYGLDGLTLRAGGVFNQDWQGASAGVVAGLGY
LGAVSADGAYATAKYRDGSHSGNKVQLSWSKQLETTNTGLRVSWSRQSEE
YEGMSSFDPTELWSQSNHGRRTKDEWNAGISQPVGGLFSLSVSGWQRSYY
PASMTGSYRYSDDNGKETGITGSLSTQIKGVSLNLGWSGSRNSRGENNWS
ASASVSVPFTLFDRRYSSSASVSTSKGGGTGFSTGVSGSLNDRFSYGLGG
GRDGDGGTSSYLNASYSGDRAYLNGVLNHSQSGGTSGSVSVSGSVLAVPA
AKDIMFSRTTGDTVAVVNVKDTPGVKVTSGDGQTDSDGNLVVPLNSYDWN
TVTIDTGTLPLSTELTNTSQKVVPTDKAVVWMPFDALKVKRYLLQVKQRD
GEFVPGGTWARDSKNTPLGFVANNGVLMINTVDAPGDITLGQCRIPAARL
QDTEKLQEITCE
> P07111|PAPH_ECOLI PAP fimbrial minor pilin protein - Escherichia coli.
MRLRFSVPLFFFGCVFVHGVFAGPF PPPGMSLPEYWGEEHVWWDGRAAFH
GEVVRPACTLAMEDAWQIIDMGETPVRDLQNGFSGPERKFSLRLRNCEFN
SQGGNLFSDSRIRVTFDGVRGETPDKFNLSGQAKGINLQIADVRGNIARA
GKVMPAIPLTGNEEALDYTLRIVRNGKKLEAGNYFAVLGFRVDYE
> P04377|AZUP_ALCFA Pseudoazurin - Alcaligenes faecalis.
MRNIAIKFAAAGILAMLAAPALAEN IEVHMLNKGAEGAMVFEPAYIKANP
GDTVTFIPVDKGHNVESIKDMIPEGAEKFKSKINENYVLTVTQPGAYLVK
CTPHYAMGMIALIAVGDSPANLDQIVSAKKPKIVQERLEKVIASAK
> P43529|E2BB_ECOLI Heat -labile enterotoxin IIB, B chain - Escherichia coli.
MSFKKIIKAFVIMAALVSVQAHAGASQFFKDNCNRTTASLVEGVELTKYI
SDINNNTDGMYVVSSTGGVWRISRAKDYPDNVMTAEMRKIAMAAVLSGMR
VNMCASPASSPNVIWAIELEAE
> P52992|DLDH_RALEH Dihydrolipoyl dehydrogenase - Ralstonia eutropha
(strain ATCC 17699 / H16 / DSM 428 / Stanier 337) (Cupriavidus necator
(strain ATCC 17699 / H16 / DSM 428 / Stanier 3 37)).
MSKQFDVLVIGAGPGGYIAAIRAGQLGLNVACCEGNPYDDPKGEARLGGT
CLNVGCIPSKALLASSEEFENVQHHLGDHGITVGDVKVDVAKMLKRKDDI
VGKMTKGIEFLFRKNKVTLLKGYGKFVGKSAEGFQVDVAGEVVTAKQVII
ATGSKARHLPGIKVDNDLVSDNEGALKFPAVPKKLGVIGAGVIGLELGSV
WRRLGSDVTVLEALPAFLGAADEGVAKEAQKQLTKQGLKFSLGVNV NEVT
TGKNGVTVKYTDKDGKAQTLEVDRLIVSVGRVPNTDNLGLDAVGLAADQR
GFIEVDDHCATKVPGLWAIGDVVRGPMLAHKAEDEGVAVAERIAGQKPHI
DYNCVPWVIYTFPEIAWVGKTEAQLKAEGREYKAGQFPFMANGRALGMGH
ADGFVKMLADAKTDEILGVHIVAANASDLIAEAVVAMEFKAASEDIGRVC
HPHPSMSEVMREAALAVDKRQLNM
> Q01269|PHEC_PSEAE Cy clohexadienyl dehydratase [Includes: Prephenate
dehydratase - Pseudomonas aeruginosa.
MPKSFRHLVQALACLALLASASLQAQESRLDRILESGVLRVATTGDYKPF
SYRTEEGGYAGFDVDMAQRLAESLGAKLVVVPTSWPNLMRDFADDRFDIA
MSGISINLERQRQAYFSIPYLRDGKTPITLCSEEARFQTLEQIDQPGVTA
IVNPGGTNEKFARANLK KARILVHPDNVTIFQQIVDGKADLMMTDAIEAR
LQSRLHPELCAVHPQQPFDFAEKAYLLPRDEAFKRYVDQWLHIAEQSGLL
RQRMEHWLEYRWPTAHGK
> Q51434|CHEZ_PSEAE Chemotaxis protein cheZ - Pseudomonas aeruginosa.
MQLIQELSQARDRGLYQEVGKLTRELHNAIVDFQIDPHSPHAQEMSQIAD
ATDRLSYVVEMTEKAANRTMDLVEQSAPLVN QLGDDSRELHQEWQRFMRR
EIDADGFRELAKRIEQFLVRSGENAGQLSSQLNDILLAQDYQDLTGQVIK
RVTKLVTEVESNLVKLVWMAGQVDRYAGIEHDHVSMRHQAALERSAKGEG
PQVAAEKREDVVSGQDDVDDLLSSLGF
> P36774|LON2_MYXXA ATP -dependent protease La 2 - Myxococcus xanthus.
MSDEKKKGSAASAMPTAMAPPGLINKEDIPQVLPIL PLRNSVFFPGGVLP
LAVGRQKTIALIKDAVRDDQVIGVVTQRRAEEEDPGAADLYTMGTVARIV
KLLKMGEDNYSLVVQGLARFRVVELVQEAPYLKARVDAVEDKTSSENVEV
EALGINLKKLAREVIELMPELPAAATELVESITHPGHLADLIAANVDVPI
EEKQAVLETVDLKARMKLVLELLNRKREILKLSNKIDSAVKGEMSKTQRE
YYLRQQLKAIKEELGEMGEEEEELDELQERLKKAGLP PDVEKVANKELNR
LKTIPAASSEYTVARTYLDWIADLPWAKISEDNLDIENARQQLDKDHFGI

KKVKKRILEYLAVRKLKNDMRGPILCLVGPPGVGKTSLGQSVAKATGRKF
VRLSLGGVRDEAEIRGHRRTYVGALPGRFIQSMKKAGTKNPVMMLDEIDK
LGADFRGDPSAALLEVLDPEQNNTFSDHYLDVPFDLSKVMFVATANQLDP
IPGPLRDRMEIIELTGYTFEEKQSIARIHLVPKQLKEH GLSPDHIDITDE
ALLTLTTAYTREAGVRNLERRIADICRAVAVEVAGGKTEKQTINADRVKE
ILGPEMFYSEVAERTEVPGVATGLAWTAAGGDLLFIEATKMAGKGGMTLT
GQLGDVMKESATAALSYLRSKAEQLGISPNFLEKTDLHLHFPAGSIPKDG
PSAGVTILTALTSLLTGIRVRHDTAMTGEATLRGLVLPVGGIKEKVLAAH
RAGIKRVILPERCRKDLIDVPDQARNELEFIFVTHMDDV LKAALETPPVG
VAGTPGGEPGKEAPLPKPAESAPEVRA
> P17201|DHAQ_ACEPO Membrane -bound aldehyde dehydrogenase
[pyrroloquinoline -quinone] - Acetobacter polyoxogenes.
MGRLNRFRLGKDGRREQASLSRRGFLVTSLGAGVMFGFARPSSANQIFPL
DRSLPGDGAFEPTIWCSIAPDGEITVNIIRAEMGQHIGTALARIIADEME
ADWSKVRINYVDTDPKWGLMVTGGSWSVWMTWDVFRQAGAATRTAMVEEG
ARLLGTTPDKCTVASSIVSAGGKQISFGDIVAKGHPSHAFTPEEMAKLPL
KPASERRLIGNAELKALDIPAKTNGTAIYGIDAKVEGMLYGRPKMPPTRY
GSKVRSVDDTEAKKIKGYVRYLLIDDPSQVVQGWVVVLAESYSAAIRATD
ALKVEWTPGETIHTSERDIQDRGRELINNKAGGVYIFNDDGVDQAFGSAH
TVMDQEYTCASVLHYQLEPTNALAFEKDGVYEIHAGNQWQSLILPTLAKS
LQVPESKVILRSYLLGGGFGRRLNGDYMIPAALASKALGGKPVKLILTRS
DDMQFDSFRSPSVQRVRMAFDASDRITAMDYQAAAGWPTGVMAEAFMEKG
VDGKPYDQFAIAGGDHWYEVGAFRVRALRNDLAEKTFRPGWLRSVSPGWT
SWGVECFLDEVAHRQKKDPAQFRLELLTGQGRNKGQAPDSVGGALRQAAV
VRRLMEKVNWGKTSLPKDTAMGLATTAGQERGMPTWDRCVAQVHVDRSTG
VVTCQKLTILVDAGTVVDPDGAKAQTEGAALWGLSMVLFENTEIVNGMPV
DRNLNTYTPLRIADTPEMDIEFLPSTEKPMGLGEPGTTVVGPAIGNAIFN
AVGVRLRHMPVRPADVLRGLQNG
> P54893|P5CR_THET2 Pyrroline -5-carboxylate reductase - Thermus
thermophilus (stra in HB27 / ATCC BAA -163 / DSM 7039).
MRLAFVGLGKMGRSILKGALERGFLRPEEVGVLGRTPERSRELAEPFGVR
PLTRADLGMAERVLIAVQPRDFPALAPEIAHHRLGYISIMAGISTSVLAR
RLDNRRVVRAMPNLAVVIGESSTALTALKEAREAEDLAFARALFATVGDV
YEIPEHLFDAFTGMSASAPAYLAVVAEALADAGVKMGMPRALALRLAADA
LAATGELLKGRHPAQV KDEVASPGGTTIHGLHALEARAVRAAFYEAVEAA
TRRGHELGESE
> P43014|SYT_HAEIN Threonyl -tRNA synthetase - Haemophilus influenzae.
MPIITLPDGSQRQFDRPVSVLEVAQDIGAGLAKATIAGRVNGERRDACYV
IEQDATLEIITAKDEDGLEIIRHSCAHLLGHAIKQLFPDVKMAIGPTIEN
GFYYDVDLDRSLTQEDIDAIEKRMLELAKTNYDVVKK RVTWQEARDTFEK
RGEPYKMAILDENIERTATPALYHHLEYIDMCRGPHVPNMRFCQHFKLQK
VAGAYWRGDSKNKMLQRIYGTAWADKKQLAEYLTRLEEAAKRDHRKIGKA
LDLYHMQEEAPGMVFWHNDGWTIFRELETFVRTKLKQYDYQEVKGPFMMD
RVLWEKTGHWQNYADLMFTTQSENREYAIKPMNCPGHVQIFNQGLKSYRD
LPIRMAEFGSCHRNEPSGSLHGLMRVRGFTQDDAHIFC TEDQIESEVTSC
IKMVYDIYSTFGFTNIAVKLSTRPENRIGSDEMWDRAEAGLAAALAHNGL
EYEIQEGEGAFYGPKIEFALRDCLGREWQCGTVQLDFALPGRLDATYVAE
DNSRKTPVMIHRAILGSIERFIGIITEEYAGFFPAWLAPTQAVVMNITDS
QADYVQKVAKQLSDVGLRVKTDLRNEKVGFKIREHTLRRVPYMLVCGDKE
IAEGKVAVRTRKGADLGTFTVEEFAEILKNQVRSRELKL LNEE
> Q01605|PHOE_CITFR Outer membrane pore protein E - Citrobacter freundii.
MKKSTLALVVMGITASASVQAAEVYNKNGNKLDLYGKVKAMHYMTDYDSK
DGDQSYIRLGFKGETQINDELTGYGRWEAEFAGNKAESDSNQQKTRLAFA
GSKLKNLGSFDYGRNLGALYDVEAWTDMFPEFGGDSSAQTDNFMTKRASG
LATYRNTDFFGVVDGLDLTLQYQG KNQDRDVKKQNGDGFGTSVTYDFGGS
DFAVSGAYTNSDRTNQQNLQTRGTGDKAEAWATGLKYDANDIYIATFYSE
TRNMTPISGGFANKTQNFEAVVQYQFDFGLRPSLGYVLSKGKDIEGVGNE
DLVNYIDVGATYYFNKNMSAFVDYKINQLDSDNKLGINNDDIVAVGMVYQ
F
> P0A2C5|RBSB_SALTY D -ribose-binding periplasmic protein - Salmonella
typhimurium.
MNMKKLATLVSAVALSATVSANAMAKDTIALVISTLNNPFFVSLKDGAQK
EADKLGYNLVVLDSQNNPAKELANVQDLTVRGTKILLINPTDSDAVGNAV

KMANQAKIPVITLDRQATKGDVVSHIASDNVLGGKIAGDYIAKKAGEGAK
VIELQGIAGTSAARERGEGFQQAVAAHKFNVLASQPADFDRTKGLNVMQN
LLTAHPDVQAVFAQNDEMALGALRALQTAGKADVMVVGFDGTP DGEKAVK
DGKLAATIAQLPDQIGAKGVEVADKVLKGEKVQAKYPVDLKLVIKQ
> P14542|IUTA_ECOLI Ferric aerobactin receptor - Escherichia coli.
MMISKKYTLWALNPLLLTMMAPAVAQQTDDETFVVSANRSNRTVAEMAQT
TWVIENAELEQQIQGGKELKDALAQLIPGLDVSSRSRTNYGMNVRGRPLV
VLVDGVRLNSSRTDSRQLDSIDPFNMHHIEVI FGATSLYGGGSTGGLINI
VTKKGQPETMMEFEAGTKSGFSSSKDHDERIAGAVSGGNEHISGRLSVAY
QKFGGWFDGNGDATLLDNTQTGLQYSDRLDIMGTGTLNIDESRQLQLITQ
YYKSQGDDDYGLNLGKGFSAIRGTSTPFVSNGLNSDRIPGTDGHLISLQY
SDSAFLGQELVGQVYYRDESLRFYPFPTVNANKQVTAFSSSQQDTDQYGM
KLTLNSKPMDGWQITWGLDADHERFTSNQMFFD LAQASASGGLNNKKIYT
TGRYPSYDITNLAAFLQSGYDINNLFTLNGGVRYQYTENKIDDFIGYAQQ
RQIGAGKATSADAFWRLSRLRHFLFNAGLLMHITEPQQAWLNFSQGLELP
DPGKYYGRGIYGAAVNGHLPLTKSVNVSDSKLEGVKVDSYELGWRFTGNN
LRTQIAAYYSISDKSVVANKDLTISVVDDKRRIYGVEGAVDYLIPDTDWS
TGVNFNVLKTESKVNGTWQKYDVKTASPSKATAY IGWAPDPWSLRVQSTT
SFDVSDAQGYKVDGYTTVDLLGSYQLPVGTLSFSIENLFDRDYTTVWGQR
APLYYSPGYGPASLYDYKGRGRTFGLNYSVLF
> P24735|AMPC_PSEAE Beta-lactamase - Pseudomonas aeruginosa.
MRDTRFPCLCGIAASTLLFATTPAIAGEAPADRLKALVDAAVQPVMKAND
IPGLAVAISLKGEPHYFSYGLASKEDGRRVTPETLFEIGSVSK TFTATLA
GYALTQDKMRLDDRASQHWPALQGSRFDGISLLDLATYTAGGLPLQFPDS
VQKDQAQIRDYYRQWQPTYAPGSQRLYSNPSIGLFGYLAARSLGQPFERL
MEQQVFPALGLEQTHLDVPEAALAQYAQGYGKDDRPLRVGPGPLDAEGYG
VKTSAADLLRFVDANLHPERLDRPWAQALDATHRGYYKVGDMTQGLGWEA
YDWPISLKRLQAGNSTPMALQPHRIARLPAPQALEGQRLLNKTG STNGFG
AYVAFVPGRDLGLVILANRNYPNAERVKIAYAILSGLEQQGKVPLKR
> SYW_SYNY3 P73655 TRYPTOPHANYL-TRNA SYNTHETASE (EC 6.1.1.2)
MKNCENDHRFTTVSSGKAWGQLHRFPSLIKFNFAHRSTTAMDKPRILSGV
QPTGNLHLGNYLGAIRSWVEQQQHYDNFFCVVDLHAITVPHNPQTLAQDT
LTIAALYLACGIDLQYSTIFVQSHVAAHSELAWLLNC VTPLNWLERMIQF
KEKAVKQGENVSVGLLDYPVLMAADILLYDADKVPVGEDQKQHLELTRDI
VIRINDKFGREDAPVLKLPEPLIRKEGARVMSLADGTKKMSKSDESELSR
INLLDPPEMIKKKVKKCKTDPQRGLWFDDPERPECHNLLTLYTLLSNQTK
EAVAQECAEMGWGQFKPLLTETAIAALEPIQAKYAEILADRGELDRIIQA
GNAKASQTAQQTLARVRDALGFLAPPY
> P43835|SYW_HAEIN Tryptophanyl-tRNA synthetase - Haemophilus influenzae.
MAKPIVFSGVQPSGELTIGNYLGALRNWVKMQEDYECIFCVVDLHAITVR
QDPVALRKATLDVLALYLACGIDPNKSTIFVQSHVPEHTQLSWVLNCYTY
FGEMSRMTQFKDKSARYAENINVGLFDYPVLMAADILLYQAKSVPVGDDQ
KQHLEITRDIANRFNALYGNIFTIPEIFIGKAGARIMSL QDPEKKMSKSD
DNRNNVVTLLEDPKSVAKKIKRAVTDSDEPPVVRYDVQNKAGVSNLLDIL
SAVTDKPIADLEKEFEGKMYGHLKTAVADEVSTLLASLQERFHQYRNDET
LLDNILRQGAEKARAKAQETLAKVYEAVGFVAAK
> P39325|YTFQ_ECOLI ABC transporter periplasmic-binding protein ytfQ -
Escherichia coli.
MWKRLLIVSAVSAAMSSM ALAAPLTVGFSQVGSESGWRAAETNVAKSEAE
KRGITLKIADGQQKQENQIKAVRSFVAQGVDAIFIAPVVATGWEPVLKEA
KDAEIPVFLLDRSIDVKDKSLYMTTVTADNILEGKLIGDWLVKEVNGKPC
NVVELQGTVGASVAIDRKKGFAEAIKNAPNIKIIRSQSGDFTRSKGKEVM
ESFIKAENNGKNICMVYAHNDDMVIGAIQAIKEAGLKPGKDILTGSIDGV
PDIYKAMMDGEANASVELT PNMAGPAFDALEKYKKDGTMPEKLTLTKSTL
YLPDTAKEELEKKKNMGY
> METK_HAEIN P43762 S-ADENOSYLMETHIONINE SYNTHETASE (EC 2.5.1
MSSYLFTSESVSEGHPDKIADQISDAVLDEILKQDPKARVACETYVKTGM
ALVGGEITTSAWVDIENLTRKVICDIGYEHSEMGFDGHSCAVLNAIGKQS
ADINQGVDRENPLDQGAGDQGIMFGYATNETDVLMPAAITY AHRLMEKQS
EVRKSGKLAWLRPDAKSQVTLKYEDNKIVGVDAVVLSTQHSEEVSQKDLH
EGVMEEIIKPVLPSEWLSKETKFFINPTGRFVIGGPMGDCGLTGRKIIVD
TYGGAARHGGGAFSGKEPSKVDRSAAYAARYVAKNIVAAGLADRCEIQLS
YAIGVAEPTSIMVETFGTGKVANELLVSLVREFFDLRPYGLIKMLDLIQP

IYRETAAYGHFGREQFPWEKVDRAEDLRIAAGLK
> P09155|RND_ECOLI Ribonuclease D - Escherichia coli.
MNYQMITTDDALASLCEAVRAFPAIALDTEFVRTRTYYPQLGLIQLFDGE
HLALIDPLGITDWSPLKAILRDPSITKFLHAGSEDLEVFLNVFGELPQPL
IDTQILAAFCGRPMSWGFASMVEEYSGVTLDKSESRTDWLARPLTERQCE
YAAADVWYLLPITAKLMVETEASGWLPAALDECRLMQMRRQEVVAPEDAW
RDITNAWQLRTRQLACLQLLADWRLRKARERDLAVNFVVREEHLWSVARY
MPGSLGELDSLGLSGSEIRFHGKTLLALVEKAQTLPEDALPQPMLNLMDM
PGYRKAFKAIKSLITDVSETHKISAELLASRRQINQLLNWHWKLKPQNNL
PELISGWRGELMAEALHNLLQEYPQ
> P51752|MIP_COXBU Peptidyl-prolyl cis-trans isomerase Mip - Coxiella burnetii.
MKRLILPFLSVGLLLGTTAHAATPLKTEQDKLSYSMGVMTGKAFRKHDIK
IDPQTFSMGLSDAYLGKETQMTEAEMRQTLQQFEKQSLQKMQHKMKQTAQ
QNAEKSRAFLTANKNKPGVKTLANGLQYKVLQAGQGQSPTLNDEVTVNYE
GRLINGTVFDSSYKRGQPATFPLKSVIKGWQEALTRMKPGAIWEIYVPPQ
LAYGEQGAPGVIGPNEALIFKVNLISVKKK
> P22542|HSTI_ECOLI Heat-stable enterotoxin II - Escherichia coli.
MKKNIAFLLASMFVFSIATNAYASTQSNKKDLCEHYRQIAKESCKKGFLG
VRDGTAGACFGAQIMVAAKGC
> P18775|DMSA_ECOLI Anaerobic dimethyl sulfoxide reductase chain A - Escherichia coli.
MKTKIPDAVLAAEVSRRGLVKTTAIGGLAMASSALTLPFSRIAHAVDSAI
PTKSDEKVIWSACTVNCGSRCPLRMHVVDGEIKYVETDNTGDDNYDGLHQ
VRACLRGRSMRRRVYNPDRLKYPMKRVGARGEGKFERISWEEAYDIIATN
MQRLIKEYGNESIYLNYGTGTLGGTMTRSWPPGNTLVARLMNCCGGYLNH
YGDYSSAQIAEGLNYTYGGWADGNSPSDIENSKLVVLFGNNPGETRMSGG
GVTYYLEQARQKSNARMIIIDPRYTDTGAGREDEWIPIRPGTDAALVNGL
AYVMITENLVDQAFLDKYCVGYDEKTLPASAPKNGHYKAYILGEGPDGVA
KTPEWASQITGVPADKIIKLAREIGSTKPAFISQGWGPQRHANGEIATRA
ISMLAILTGNVGINGGNSGAREGSYSLPFVRMPTLENPIQTSISMFMWTD
AIERGPEMTALRDGVRGKDKLDVPIKMIWNYAGNCLINQHSEINRTHEIL
QDDKKCELIVVIDCHMTSSAKYADILLPDCTASEQMDFALDASCGNMSYV
IFNDQVIKPRFECKTIYEMTSELAKRLGVEQQFTEGRTQEEWMRHLYAQS
REAIPELPTFEEFRKQGIFKKRDPQGHHVAYKAFREDPQANPLTTPSGKI
EIYSQALADIAATWELPEGDVIDPLPIYTPGFESYQDPLNKQYPLQLTGF
HYKSRVHSTYGNVDVLKAACRQEMWINPLDAQKRGIHNGDKVRIFNDRGE
VHIEAKVTPRMMPGVVALGEGAWYDPDAKRVDKGGCINVLTTQRPSPLAK
GNPSHTNLVQVEKV
> P0A8F8|UVRB_ECOLI UvrABC system protein B - Escherichia coli.
MSKPFKLNSAFKPSGDQPEAIRRLEEGLEDGLAHQTLLGVTGSGKTFTIA
NVIADLQRPTMVLAPNKTLAAQLYGEMKEFFPENAVEYFVSYYDYYQPEA
YVPSSDTFIEKDASVNEHIEQMRLSATKAMLERRDVVVVASVSAIYGLGD
PDLYLKMMLHLTVGMIIDQRAILRRLAE LQYARNDQAFQRGTFRVRGEVI
DIFPAESDDIALRVELFDEEVERLSLFDPLTGQIVSTIPRFTIYPKTHYV
TPRERIVQAMEEIKEELAARRKVLLENNKLLEEQRLTQRTQFDLEMMNEL
GYCSGIENYSRFLSGRGPGEPPPTLFDYLPADGLLVVDESHVTIPQIGGM
YRGDRARKETLVEYGFRLPSALDNRPLKFEEFEALAPQTIYVSATPGNYE
LEKSGGDVVDQVVRPTGLLDPIIEVRPVA TQVDDLLSEIRQRAAINERVL
VTTLTKRMAEDLTEYLEEHGERVRYLHSDIDTVERMEIIRDLRLGEFDVL
VGINLLREGLDMPEVSLVAILDADKEGFLRSERSLIQTIGRAARNVNGKA
ILYGDKITPSMAKAIGETERRREKQQKYNEEHGITPQGLNKKVVDILALG
QNIAKTKAKGRGKSRPIVEPDNVPMDMSPKALQQKIHELEGLMMQHAQNL
EFEEAAQIRDQLHQLRELFIAAS
> P28307|CSGA_ECOLI Major curlin subunit - Escherichia coli.
MKLLKVAAIAAIVFSGSALAGVVPQYGGGGNHGGGGNNSGPNSELNIYQY
GGGNSALALQTDARNSDLTITQHGGGNGADVGQGSDDSSIDLTQRGFGNS
ATLDQWNGKNSEMTVKQFGGGNGAAVDQTASNSSVNVTQVGFGNNATAHQ
Y
> P11278|PELP_YERPS Periplasmic pectate lyase - Yersinia pseudotuberculosis.
MKKRALLLSMSVLAMLYIPAGQAAEIDRLTVVKQYVDNVLNKASDTYHGD
KPSPLLADGVDPRTGQQMEWIFPDGRRAVLSNFSAQQNLMRVMSGLSELS
GDPQYQKRAEDIVRYHFQNYQDNSGLLYWGGHRFVDLKTLQPEGPSEKEK

VHELKNAYPYYDLMFSVDSDATTRFIRGFWNAHVYDWRILETSRHGEYGK
PMGALWESTFEQQPPFFATKG LSFLNAGNDLIYSASLLYKYQQDQGALVW
AKRLADQYVLPRDAKTGLGVYQFTQALKREEPTDDADTHSKFGDRAQRQF
GPEFGPTALEGNMMLKGRTSTLYSENALMQLQLGKDLGGQGDDLLKWTVD
GLKAFAKYGYNEQDNTFRPMIANGQDLSNYTLPRDGYYGKKGSVLKPYKA
GNEFLISYARAYAVDNDPLLWKVARGIASDQGLGDIGSAPGKEMKVKLDT
TNSDPYALFALLDLYNASQVAE YRSLAEKVADNIIKTRYIDGFFMASPDR
QYADVDAIEPYALLALEASLRNKPQAVAPFLNGAGFTEGAYLMADGSARI
STRDNELFLLNVGETLQPNGRK
> GAL_PSEFL P11886 D -GALACTOSE 1 -DEHYDROGENASE (EC 1.1.1.48).
MQPIRLGLVGYGKIAQDQHVPAINANPAFTLVSVATQGKPCPGVENFQSL
GELLENGPPVDAIAFCTPPQGRFALVQQALAAGKHVLVEK PPCATLGKAA
LWIKREQASAPCSPCIAYAPAIAAARDWLATRTLQSVQIDWKEDVRKWHP
GQAWIWQPGLGVFDPGINALSIVTHLLPLPLFVESAELRVPSNCQSPIAA
SIKMSDPRLLDVRAEFDFDHGHDELWSIQIRCAEGTLRLDNGGALLSIDG
VRQTVAEEGEYAAVYRHFQQLIGDKTSDVDVQPLRLVADSFFVGSRVSVE
AFYD
> P38683|TORT_ECOLI Periplasmic prot ein torT - Escherichia coli.
MRVLLFLLLSLFMLPAFSADNLLRWHDAQHFTVQASTPLKAKRAWKLCAL
YPSLKDSYWLSLNYGMQEAARRYGVDLKVLEAGGYSQLATQQAQIDQCKQ
WGAEAILLGSSTTSFPDLQKQVASLPVIELVNAIDAPQVKSRVGVPWFQM
GYQPGRYLVQWAHGKPLNVLLMPGPDNAGGSKEMVEGFRAAIAGSPVRIV
DIALGDNDIEIQRNLLQEMLERH PEIDVVAGTAIAAEAAMGEGRNLKTPL
TVVSFYLSHQVYRGLKRGRVIMAASDQMVWQGELAVEQAIRQLQGQSVSD
NVSPPILVLTPKNADREHIRRSLSPGGFRPVYFYQHTSAAKK
> FTN_HELPY P52093 NONHEME IRON -CONTAINING FERRITIN. - HELICO
MLSKDIIKLLNEQVNKEMNSSNLYMSMSSWCYTHSLDGAGLFLFDHAAEE
YEHAKKLIVFLNENNVPVQLT SISAPEHKFEGLTQIFQKAYEHEQHISES
INNIVDHAIKGKDHATFNFLQWYVSEQHEEEVLFKDILDKIELIGNENHG
LYLADQYVKGIAKSRKS
> Q06987|TBP12_NEIMB Transferrin -binding protein 1 - Neisseria meningitidis serogroup B.
MQQQHLFRLNILCLSLMTALPVYAENVQAEQAQEKQLDTIQVKAKKQKTR
RDNEVTGLGKLVKSSDT LSKEQVLNIRDLTRYDPGIAVVEQGRGASSGYS
IRGMDKNRVSLTVDGVSQIQSYTAQAALGGTRTAGSSGAINEIEYENVKA
VEISKGSNSSEYGNGALAGSVAFQTKTAADIIGEGKQWGIQSKTAYSGKD
HALTQSLALAGRSGGAEALLIYTKRRGREIHAHKDAGKGVQSFNRLVLDE
DKKEGGSQYRYFIVEEECHNGYAACKNKLKEDASVKDERKTVSTQDYTGS
NRLLANPLEYGSQSWLFR PGWHLDNRHYVGAVLERTQQTFDTRDMTVPAY
FTSEDYVPGSLKGLGKYSGDNKAERLFVQGEGSTLQGIGYGTGVFYDERH
TKNRYGVEYVYHNADKDTWADYARLSYDRQGIDLDNRLQQTHCSHDGSDK
NCRPDGNKPYSFYKSDRMIYEESRNLFQAVFKKAFDTAKIRHNLSINLGY
DRFKSQLSHSDYYLQNAVQAYDLITPKKPPFPNGSKDNPYRVSIGKTTVN
TSPICRFGNNTYTDCTPRN IGGNGYYAAVQDNVRLGRWADVGAGIRYDYR
STHSEDKSVSTGTHRNLSWNAGVVLKPFTWMDLTYRASTGFRLPSFAEMY
GWRAGESLKTLDLKPEKSFNREAGIVFKGDFGNLEASYFNNAYRDLIAFG
YETRTQNGQTSASGDPGYRNAQNARIAGINILGKIDWHGVWGGLPDGLYS
TLAYNRIKVKDADIRADRTFVTSYLFDAVQPSRYVLGLGYDHPDGIWGIN
TMFTYSKAKSVDELLGSQAL LNGNANAKKAASRRTRPWYVTDVSGYYNIK
KHLTLRAGVYNLLNYRYVTWENVRQTAGGAVNQHKNVGVYNRYAAPGRNY
TFSLEMKF
> Q43990|SYK_ACIAD Lysyl -tRNA synthetase - Acinetobacter sp. (strain ADP1).
MAQPNVQSTSEPILSENDLIAQRHAKLKQIQDKAKETGKSVWPNTFKREH
YAADLQEQFKDIDKAQIEGSDKTYVKVAGRVMLNRGSF MVIQDMTGRIQL
YVDRKGLPADTLETIKSLDLGDIIAAEGYIGRSGKGDLYVHLEGFELLTK
SLRPLPDKFHGLTDTEAKYRKRYLDLIVNEETRKTFEIRAQVVAGIRAFL
TNQRFMEVETPMMHVIPGGASAQPFVTHHNALDMELYLRIAPELYLKRLV
VGGFERVFEINRNFRNEGVSTRHNPEFTMIEFYQAYADYKDLMQLTENML
EKLALDILGTTDVPYGDEVYSFKGPFKKISMFDAILEHN PDFTPENVNDR
EFLAKFTQDVLKEQVKPGFGLGKLQTIVFEETVETKLRQPTFITEYPAET
SPLARRNDDNPHITDRFEFFIGGRELANGFSELNDPIDQAERFQAQVAEK
DAGDDEAMHYDADFIEALEYGLPPTAGQGIGIDRLVMIFANAPSIRDVLL
FPHMRRKDV

> P52854|EFTU_TREHY Elongation factor Tu - Treponema hyodysenteriae (Serpulina hyodysenteriae).
MAKGTYEGNKTHVNVGTIGHVDHGKTTLTSAITAVSSAMFPATVQKVAYD
SVAKASESQGRRDPTKILTIATSHVEYESDNRHYAHVDCPGHADYIKNMI
TGAAQMDGAILVVSAEDGVMPQTKEHVLLSRQVGVNYIVVFLNKCDKLDD
PEMAEIVEAEVIDVLDHYGFDGSKTPIIRGSAIKAIQAIEAGKDPRTDPD
CKCILDLLNALDTYIPDPVREVDKDFLMSIEDVYSIP GRGTVVTGRIERG
KIEKGNEVEIVGIRPTQKTTCTGVEMFKKEVVGIAGYNVGCLLRGIERKA
VERGQVLAKPGTITPHKKFEAEVYILKKEEGGRHSGFVSGYRPQMYFRTT
DVTGVINLQGDAQMIMPGDNANLTIELITPIAMEEKQRFAIREGGKTVGN
GVVTKNIRII
> P12625|PHB_ALCFA Poly(3-hydroxybutyrate) depolymerase - Alcaligenes faecalis.
MVRRLWRRIAGWLAACVAILCAFPLHAATAGPGAWSSQQTWAADSVNGGN
LTGYFYWPASQPTTPNGKRALVLVLHGCVQTASGDVIDNANGAGFNWKSV
ADQYGAVILAPNATGNVYSNHCWDYANASPSRTAGHVGVLLDLVNRFVTN
SQYAIDPNQVYVAGLSSGGGMTMVLGCIAPDIFAGIGINAGPPPGTTTAQ
IGYVPSGFTATTAANKCNAWAGSNAGKFSTQIAGAVWGTSDYTVAQAYGP
MDAAAMRLVYGGNFTQGSQVSISGGGTNTPYTDSNGKVRTHEISVSGMAH
AWPAGTGGDNTNYVDATHINYPVFVMDYWVKNNLRAGSGTGQAGSAPTGL
AVTATTSTSVSLSWNAVANASSYGVYRNGSKVGSATATAYTDSGLIAGTT
YSYTVTAVDPTAGESQPSAAVSATTKSAFTCTATTASNYAHVQAGRAHDS
GGIAYANGSNQSMGLDNLFYTSTLAQTAAGYYIVGNCP
> P17215|LIVJ_SALTY Leu/Ile/Val/Thr-binding protein - Salmonella typhimurium.
MKGKTLLAGCIALSLSHMAFADDIKVAVVGAMSGPVAQYGDQEFTGAEQA
IADINAKGGIKGDKLVAVKYDDACDPKQAVAVANKVVNDGIKYVIGHLCS
SSTQPASDIYEDEGILMITPAATAPELTARGYKLVLRTTGLDSDQGPTAA
KYILEKVKPQRIAIIHDKQQYGEGLARAVQDGLKKGG VNVVFFDGITAGE
KDFSTLVARLKKENIDFVYYGGYHPEMGQILRQSRAAGLKTQFMGPEGVA
NVSLSNIAGESAEGLLVTKPKNYDQVPANKPIVDAIKAKKQDPSGAFVWT
TYAALQSLQAGLNHSDDPAEIAKYLKGATVDTVMGPLSWDEKGDLKGFEF
GVFDWHANGTATDAK
> P12061|FM_SALEN Fimbrial protein - Salmonella enteritidis.
MRKSASAVAVLA LIACGSAHAAGFVGNKAVVQAAVTIAAQNTTSANWSQD
PGFTGPAVAAGQKVGTLSITATGPHNSVSIAGKGASVSGGVATVPFVDGQ
GQPVFRGRIQGANINDQANTGIDGLAGWRVASSQETLNVPVTTFGKSTLP
AGTFTATFYVQQYQN
> P05458|PTRA_ECOLI Protease 3 - Escherichia coli.
MPRSTWFKALLLLVALWAPLSQAETGWQPIQETIRKSDKDNRQYQAIR LD
NGMVVLLVSDPQAVKSLSALVVPVGSLEDPEAYQGLAHYLEHMSLMGSKK
YPQADSLAEYLKMHGGSHNASTAPYRTAFYLEVENDALPGAVDRLADAIA
EPLLDKKYAERERNAVNAELTMARTRDGMRMAQVSAETINPAHPGSKFSG
GNLETLSDKPGNPVQQALKDFHEKYYSANLMKAVIYSNKPLPELAKMAAD
TFGRVPNKESKKPEITVPVVTDAQKGIIIHYVPALPRKVLRVEFRIDNN S
AKFRSKTDELITYLIGNRSPGTLSDWLQKQGLVEGISANSDPIVNGNSGV
LAISASLTDKGLANRDQVVAAIFSYLNLLREKGIDKQYFDELANVLDIDF
RYPSITRDMDYVEWLADTMIRVPVEHTLDAVNIADRYDAKAVKERLAMMT
PQNARIWYISPKEPHNKTAYFVDAPYQVDKISAQTFADWQKKAADIALSL
PELNPYIPDDFSLIKSEKKYDHPELIVDESNLRVVYAPSRYFASEPKADV
SLILRNPKAMDSARNQVMFALNDYLAGLALDQLSNQASVGGISFSTNANN
GLMVNANGYTQRLPQLFQALLEGYFSYTATEDQLEQAKSWYNQMMDSAEK
GKAFEQAIMPAQMLSQVPYFSRDERRKILPSITLKEVLAYRDALKSGARP
EFMVIGNMTEAQATTLARDVQKQLGADGSEWCRNKDVVVDKKQSVIFEKA
GNSTDSALAAVFVPTGYDEYTSSAYSSLLGQIVQPWFYNQLRTEEQLGYA
VFAFPMSVGRQWGMGFLLQSNDKQPSFLWERYKAFFPTAEAKLRAMKPDE
FAQIQQAVITQMLQAPQTLGEEASKLSKDFDRGNMRFDSRDKIVAQIKLL
TPQKLADFFHQAVVEPQGMAILSQISGSQNGKAEYVHPEGWKVWENVSAL
QQTMPLMSEKNE
> P13626|CYF_NOSSP Apocytochrome f - Nostoc sp. (strain PCC 7906).
MRNASVTARLTRSVRAIVKTLLI AIATVTFYFSCDLALPQSAAAYPFWAQ
QTYPETPREPTGRIVCANCHLAAKPTEVEVPQSVLPDTVFKAVVKIPYDT
SAQQVGADGSKVGLNVGAVLMLPEGFKIAPEDRISEELQEEIGDTYFQPY

SEDKENIVIVGPLPGEQYQEIVFPVLSPNPATDKNIHFGKYSVHVGGNRG
RGQVYPTGEKSNNNLYNASATGTIAKIAKEEDEDGNVKYQVNIQPESGDV
VVDTVPAGPELIVSEGQAVKAGDA LTNNPNVGGFGQRDAEIVLQDAGRVK
GLIAFVALVMLAQVMLVLKKKQVERVQAAEMNF
> Q00499|CY550_PARVE Cytochrome c -550 - Paracoccus versutus (Thiobacillus versutus).
MKISIYATLAALSLALPAVAQEGDAAKGEKEFNKCKACHMVQAPDGTDIV
KGGKTGPNLYGVVGRKIASVEGFKYGDGILEVAEKNPDMVWSEADLIEYV
TDPKPWLVEKTGDSAAKTKMTFKLGKNQADVVAFLAQHSPDAGAEAAPAE
GAAN
> P18895|ALGE_PSEAE Alginate production protein algE - Pseudomonas aeruginosa.
MNSSRSVNPRPSFAPRALSLAIALLLGAPAFAANSGEAPKNFGLDVKITG
ESENDRDLGTAPGGTLNDIGIDLRPWAFGQWGDWSAYFMGQAVAATDTIE
TDTLQSDTDDGNNSRNDGREPDKSYLA AREFWVDYAGLTAYPGEHLRFGR
QRLREDSGQWQDTNIEALNWSFETTLLNAHAGVAQRFSEYRTDLDELAPE
DKDRTHVFGDISTQWAPHHRIGVRIHHADDSGHLRRPGEEVDNLDKTYTG
QLTWLGIEATGDAYNYRSSMPLNYWASATWLTGDRDNLTTTTVDDRRIAT
GKQSGDVNAFGVDLGLRWNIDEQWKAGVGYARGSGGGKDGEEQFQQTGLE
SNRSNFTGTRSRVHRFGEAFRGELSNLQ AATLFGSWQLREDYDASLVYHK
FWRVDDDSDIGTSGINAALQPGEKDIGQELDLVVTKYFKQGLLPASMSQY
VDEPSALIRFRGGLFKPGDAYGPGTDSTMHRAFVDFIWRF
> G6PI_SYNY3 P52983 GLUCOSE -6-PHOSPHATE ISOMERASE (GPI) (EC 5
MNNQQLWQRYQDWLYYHGGLDFYLDVSRMGFSDALVEDLQPKFAKAFQDM
VALEKGAIANPDEQRMVGHYWLRNPALA PNDGIRAEITEPLRQIKAFVAD
VHQGNIKPPTAPKFTDLLAIGIGGSALGPQFVAQALAPNFPPLAIHFIDN
SDPDGIDRVLNCLKAQDKLKSTLVVTTSKSGGTPEPRNGLAETKAVFEAQ
GLHFADYAVAVTMPGSKLSQQAQTEQWLQAFPMQDWVGGRTSELSAVGLL
PAALQGIDIQAMLDGAKTMDEATRVRELRQNPAALLALAWYYAGDGQGKK
DMVILPYKDRLLLFSRYLQQLVMESLGKE RDLDGNVVHQGIAVYGNKGST
DQHAYVQQLRDGVPNFFATFIEVLHDRQGPSLELEPGVTSGDYLSGFLQG
TRQALFENQRDSITVTIPEVDATSVGALIALYERAVSFYGSLVNVNAYHQ
PGVEAGKKAAASILELQKAILSTLQNESGPIALEALATKVQAPEQVETVY
KIVRHLAANDRGVTLQGDRQFPQRLQIQWRS
> P10908|UGPQ_ECOLI Glycerophosphoryl diester pho sphodiesterase - Escherichia coli.
MSNWPYPRIVAHRGGGKLAPENTLASIDVGAKYGHKMIEFDAKLSKDGEI
FLLHDDNLERTSNGWGVAGELNWQDLLRVDAGSWYSKMFKGEPLPLLSQV
AERCREHGMMANIEIKPTTGTGPLTGKMVALAARELWAGMTPPLLSSFEI
DALEAAQQAAPELPRGLLLDEWRDDWRELTARLGCVSIHLNHKLLNKARV
MQLKDAGLRILVYTVNK PQRAAELLRWGVDCICTDAIDVIGPNFTAQ
> P0AGC3|SLT_ECOLI Soluble lytic murein transglycosylase - Escherichia coli.
MEKAKQVTWRLLAAGVCLLTVSSVARADSLDEQRSRYAQIKQAWDNRQMD
VVEQMMPGLKDYPLYPYLEYRQITDDLMNQPAVTVTNFVRANPTLPPART
LQSRFVNELARREDWRGLLAFSPEKPGTTEAQCNYYYAKWNTGQSE EAWQ
GAKELWLTGKSQPNACDKLFSVWRASGKQDPLAYLERIRLAMKAGNTGLV
TVLAGQMPADYQTIASAIISLANNPNTVLTFARTTGATDFTRQMAAVAFA
SVARQDAENARLMIPSLAQAQQLNEDQIQELRDIVAWRLMGNDVTDEQAK
WRDDAIMRSQSTSLIERRVRMALGTGDRRGLNTWLARLPMEAKEKDEWRY
WQADLLLERGREAEAKEILHQLMQQRGFYPMVAAQRIGEEYELKIDK APQ
NVDSALTQGPEMARVRELMYWNLDNTARSEWANLVKSKSKTEQAQLARYA
FNNQWWDLSVQATIAGKLWDHLEERFPLAYNDLFKRYTSGKEIPQSYAMA
IARQESAWNPKVKSPVGASGLMQIMPGTATHTVKMFSIPGYSSPGQLLDP
ETNINIGTSYLQYVYQQFGNNRIFSSAAYNAGPGRVRTWLGNSAGRIDAV
AFVESIPFSETRGYVKNVLAYDAYYRYFMGDKPTLMSATEWGRRY
> IADA_ECOLI P39377 ISOASPARTYL DIPEPTIDASE (EC 3.4.19. -). -
MIDYTAAGFTLLQGAHLYAPEDRGICDVLVANGKIIAVASNIPSDIVPNC
TVVDLSGQILCPGFIDQHVHLIGGGGEAGPTTRTPEVALSRLTEAGVTSV
VGLLGTDSISRHPESLLAKTRALNEEGISAWMLTGAYHVPSRTITGSVEK
DVAIIDRVIGVKCAISDHRSAAPDVYHLANMAAESRVGGLLGG KPGVTVF
HMGDSKKALQPIYDLLENCDVPISKLLPTHVNRNVPLFEQALEFARKGGT
IDITSSIDEPVAPAEGIARAVQAGIPLARVTLSSDGNGSQPFFDDEGNLT
HIGVAGFETLLETVQVLVKDYDFSISDALRPLTSSVAGFLNLTGKGEILP

GNDADLLVMTPELRIEQVYARGKLMVKDGKACVKGTFETA
> P0AET2|HDEB_ECOLI Protein hdeB - Escherichia coli.
MNISSLRKAFIFMGAVAALSLVNAQSALAANESAKDMTCQEFIDLNPKAM
TPVAWWMLHEETVYKGGDTVTLNETDLTQIPKVIEYCKKNPQKNLYTFKN
QASNDLPN
> P21948|PPB_ESCFE Alkaline phosphatase - Escherichia fergusonii.
MKQSAIALALLSCLITPVSQAQTSQNINILENRAAQGDITMPGGARRLSG
DQTEALRASLNDKPAKNIILLIGDGMGDS EITAARNYAEGAGGYFKGIDA
LPLTGQYTHYALDKKTGKPDYVTDSAASATAWTTGVKTYNGALGVDIHEN
PHTTILEMAKAAGLATGNVSTAELQDATPAALVSHVTSRKCYGPSVTSEK
CPGNALEKGGKGSITEQLLNARADVTLGGGAKTFAETATAGEWQGKTLRE
QALARGYQIVSDAASLAAVTQAGQDKPLLGLFAEGNMPVRWHGPKASYHG
NLDKPAVTCTPNPQRNETVPTLAQMTDKAI ELLSKNERGFFLQVEGASID
KQDHAANPCGQIGETVDLDEAVQRALEFAKKDGNTLVIVTADHAHSSQIV
APDTKAPGLTQALNTKDGAVMAISYGNSEEDSQEHTGSQLRIAAYGPNAA
NVVGLTDQTDLFYTMKAALGLQ
> NAHR_PSEPU P10183 TRANSCRIPTIONAL ACTIVATOR PROTEIN NAHR. -
MELRDLDLNLLVVFNQLLVDRRVSITAENLGLTQPAVSNALKRLRTSL QD
PLFVRTHQGMEPTPYAAHLAEPVTSAMHALRNALQHHESFDPLTSERTFT
LAMTDIGEIYFMPRLMDVLAHQAPNCVISTVRDSSMSLMQALQNGTVDLA
VGLLPNLQTGFFQRRLLQNHYVCLCRKDHPVTREPLTLERFCSYGHVRVI
AAGTGHGEVDTYMTRVGIRRDIRLEVPHFAAVGHILQRTDLLATVPIRLA
DCCVEPFGLSALPHPVVLPEIAINMFWHAKYHKDLANIWLRQLMFDLFT D

> P37188|PTKB_ECOLI Galactitol -specific phosphotransferase enzyme IIB component - Escherichia coli.
MKRKIIVACGGAVATSTMAAEEIKELCQNHNIPVELIQCRVNEIETYMDG
VHLICTTAKVDRSFGDIPLVHGMPFISGIGIEALQNKILTILQG
> P00956|SYI_ECOLI Isoleucyl -tRNA synthetase - Escherichi a coli.
MSDYKSTLNLPETGFPMRGDLAKREPGMLARWTDDDLYGIIRAAKKGKKT
FILHDGPPYANGSIHIGHSVNKILKDIIVKSKGLSGYDSPYVPGWDCHGL
PIELKVEQEYGKPGEKFTAAEFRAKCREYAATQVDGQRKDFIRLGVLGDW
SHPYLTMDFKTEANIIRALGKIIGNGHLHKGAKPVHWCVDCRSALAEAEV
EYYDKTSPSIDVAFQAVDQDALKAKFAVSNVNGPISLVIWTTTP WTLPAN
RAISIAPDFDYALVQIDGQAVILAKDLVESVMQRIGVTDYTILGTVKGAE
LELLRFTHPFMGFDVPAILGDHVTLDAGTGAVHTAPGHGPDDYVIGQKYG
LETANPVGPDGTYLPGTYPTLDGVNVFKANDIVVALLQEKGALLHVEKMQ
HSYPCCWRHKTPIIFRATPQWFVSMDQKGLRAQSLKEIKGVQWIPDWGQA
RIESMVANRPDWCISRQRTWGVPMSLFVHKDTEELHPRTLELMEE VAKRV
EVDGIQAWWDLDAKEILGDEADQYVKVPDTLDVWFDSGSTHSSVVDVRPE
FAGHAADMYLEGSDQHRGWFMSSLMISTAMKGKAPYRQVLTHGFTVDGQG
RKMSKSIGNTVSPQDVMNKLGADILRLWVASTDYTGEMAVSDEILKRAAD
SYRRIRNTARFLLANLNGFDPAKDMVKPEEMVVLDRWAVGCAKAAQEDIL
KAYEAYDFHEVVQRLMRFCSVEMGSFYLDIIKDRQYTAKADSVARR SCQT
ALYHIAEALVRWMAPILSFTADEVWGYLPGEREKYVFTGEWYEGLFGLAD
SEAMNDAFWDELLKVRGEVNKVIEQARADKKVGGSLEAAVTLYAEPELSA
KLTALGDELRFVLLTSGATVADYNDAPADAQQSEVLKGLKVALSKAEGEK
CPRCWHYTQDVGKVAEHAEICGRCVSNVAGDGEKRKFA
> NIRD_ECOLI P23675 NITRITE REDUCTASE (NAD(P)H) SMALL SUBUN IT
MSQWKDICKIDDILPETGVCALLGDEQVAIFRPYHSDQVFAISNIDPFFE
SSVLSRGLIAEHQGELWVASPLKKQRFRLSDGLCMEDEQFSVKHYEARVK
DGVVQLRG
> P73071|PROB_SYNY3 Glutamate 5 -kinase - Synechocystis sp. (strain PCC 6803).
MTMAMQPQTLVIKIGTSSLARPETGQLALSTIAALVETVCKLIGQGHRVV
LVSSGAIGVGCSR LGLTERPKKMALKQAIAAVGQGRLMRTYDDLFSSLRQ
PIAQILLTRRELIERTAYVNAYNTFQALFELGVIAIVNENDTVAIDELKF
GDNDTLSALVASLVEADWLFLLTDVDRLYSSDPRLDPDAYPIPLVKAAEL
AQLQVRTDSTGSAWGTGGMATKITAARIATGSGVRTVITHGQKPEQILAI
LQGANLGTQFEAQPRSDNARKRWIAYGLVPTGKIFIDAGAVQALKARGKS
LLAIGVVALEGEFS ATDAVIICDPQGQELGRGLVNYNCNELEKIKGLHSE
AIAAVLGYVGPDTVIHRDNLVLQEN
> P04693|TYRB_ECOLI Aromatic -amino-acid aminotransferase - Escherichia coli.

MFQKVDAYAGDPILTLMERFKEDPRSDKVNLSIGLYYNEDGIIPQLQAVA
EAEARLNAQPHGASLYLPMEGLNCYRHAIAPLLFGADHPVLKQQRVATIQ
TLGGSGALKVGADF LKRYFPESGVWVSDPTWENHVAIFAGAGFEVSTYPW
YDEATNGVRFNDLLATLKTLPARSIVLLHPCCHNPTGADLTNDQWDAVIE
ILKARELIPFLDIAYQGFGAGMEEDAYAIRAIASAGLPALVSNSFSKIFS
LYGERVGGLSVMCEDAEAAGRVLGQLKATVRRNYSSPPNFGAQVVAAVLN
DEALKASWLAEVEEMRTRILAMRQELVKVLSTEMPERNFDYLLNQRGMFS
YTGLSAAQVDRLREE FGVYLIASGRMCVAGLNTANVQRVAKAFAAVM
> P21517|MALZ_ECOLI Maltodextrin glucosidase - Escherichia coli.
MMLNAWHLPVPPFVKQSKDQLLITLWLTGEDPPQRIMLRTEHDNEEMSVP
MHKQRSQPQPGVTAWRAAIDLSSGQPRRRYSFKLLWHDRQRWFTPQGFSR
MPPARLEQFAVDVPDIGPQWAADQIFYQIFPDRFARSLPREAEQDHVYYH
HAAGQEIILRDWDEPVTAQAGGSTFYGGDLDGISEKLPYLKKLGVTALYL
NPVFKAPSVHKYDTEDYRHVDPQFGGDGALLRLRHNTQQLGMRLVLDGVF
NHSGDSHAWFDRHNRGTGGACHNPESPWRDWYSFSDDGTALDWLGYASLP
KLDYQSESLVNEIYRGEDSIVRHWLKAPWNMDGWRLDVVHMLGEAGGARN
NMQHVAGITEAAKETQPEAYIVGEHFGDARQWLQADVEDAAMNYRGFTFP
LWGFLANTDISYDPQQIDAQTCMAWMDNYRAGLSHQQQLRMFNQLDSHDT
ARFKTLLGRDIARLPLAVVWLFTWPGVPCIYYGDEVGLDGKNDPFCRKPF
PWQVEKQDTALFALYQRMIALRKKSQALRHGGCQVLYAEDNVVVFVRVLN
QQRVLVAINRGEACEVVLPASPFLNAVQWQCKEGHGQLTDGILALPAISA
TVWMN
> P20041|PGLR2_RALSO Polygalacturonase - Ralstonia solanacearum
(Pseudomonas solanacearum).
MNHRYTLLALAAAALSAGAHATGTSVTAPWGEVAEPSLPADSAVCKTLSA
SITPIKGSVDSVDGNPANSQPDASRIQSAIDNCPAGQAVKLVKGSAGESG
FLSGSLKLKSGVTLWIDTGVTLFASRNPADYDNGLGTCGTATTSNDKSCN
ALIVARDTAGSGIVGAGAIDGRGGSLVTSGPNANRLTWWDIAYLNKTKGL
NQQNPRLIQTYN GSAFTLYGVTVQNSPNFHIVTTGTSGVTAWGIKIVTPS
LAYAVAGYKCPSGSTPDKVTPATCFTPETVKNTDGFDPGQSTNVVLAYSY
INTGDDHVAVKASSGPTRNLLFAHNHFYYGHGLSIGSETNTGVSNMLVTD
LTMDGNDSSAGNGLRIKSDASRGGKVTNIVYDGICMRNVKEPLVFDPFYS
SVKGSLYPNFTNIVVKNFHDLGSAKSIKRTMTFLGYKANKQKNPLTITLD
NVVFDGTLPAFEG SHYGGPASPNGVHFTFGGTGPVSFADAIVTSSTTDVT
VTGTPGTAAAVDCSKAFVPLKSVAPTSPI
> P04170|RUBR1_DESDE Rubredoxin 1 - Desulfovibrio desulfuricans.
MQKYVCNVCGYEYDPAEHDNVPFDQLPDDWCCPVCGVSKDQFSPA
> P45369|THIL_CHRVI Acetyl-CoA acetyltransferase - Chromatium vinosum
(Allochromatium vinosum).
MSENIVIVDAGRSAIGTFGGSLSSLSATEIGTAVLKGLLARTGLAPEQID
EVILGQVLTAGVGQNPARQTTLHAGLPHSVPAMTINKVCGSGLKAVHLAM
QAIACGDADIVIAGGQESMSQSSHVLPRSRDGQRMGDWSMKDTMIVDGLW
DAFNNYHMGTTAENIAQKYGFTREQQDAFAAASQQKTEAAQKAGRFQDEI
IPIEIPQRKGDPKVFDADEFPRHGTTAESLGKL RPAFSKDGSVTAGNASG
INDGAAMVVVMKESKAKELGLKPMARLVAFASAGVDPAIMGTGPIPASTK
CLEKAGWTPADLDLIEANEAFAAQAMSVNQDMGWDLSKVNVNGGAIAIGH
PIGASGARVLVTLLYEMQKRDAKKGLATLCIGGGQGVALAVERM
> P22391|BLO1_KLEOX Beta-lactamase OXY-1 - Klebsiella oxytoca.
MLKSSWRKTALMAAAAVPLLLASGSLWA SADAIQQKLADLEKRSGGRLGV
ALINTADDSQTLYRGDERFAMCSTGKVMAAAAVLKQSESNPEVVNKRLEI
KKSDLVVWSPITEKHLQSGMTLAELSAAALQYSDNTAMNKMISYLGGPEK
VTAFAQSIGDVTFRLDRTEPALNSAIPGDKRDTTTPLAMAESLRKLTLGN
ALGEQQRAQLVTWLKGNTTGGQSIRAGLPASWAVGDKTGAGDYGTTNDIA
VIWPENHAPLVLVTYFTQPQQDAKSRKEV LAAAAKIVTEGL
> LIPA_ECOLI P25845 LIPOIC ACID SYNTHETASE (LIP-SYN). - ESCHE
MSKPIVMERGVKYRDADKMALIPVKNVATEREALLRKPEWMKIKLPADST
RIQGIKAAMRKNGLHSVCEEASCPNLAECFNHGTATFMILGAICTRRCPF
CDVAHGRPVAPDANEPVKLAQTIADMALRYVVITSVDRDDLRDGGAQHFA
DCITAIREKSPQIKIETLVPDFRGRMDR ALDILTATPPDVFNHNLENVPR
IYRQVRPGADYNWSLKLLERFKEAHPEIPTKSGLMVGLGETNEEIIEVMR
DLRRHGVTMLTLGQYLQPSRHHLPVQRYVSPDEFDEMKAEALAMGFTHAA
CGPFVRSSYHADLQAKGMEVK
> P09545|HLYA_VIBCH Hemolysin - Vibrio cholerae.

MPKLNRCAIAIFTILSAISSPTLLANINEPSGEAADIISQVADSHAIKYY
NAADWQAEDNALPSLAELRDLVINQQKRVLVDFSQISDAEGQAEMQAQFR
KAYGVGFANQFIVITEHKGELLFTPFDQAEEVDPQLLEAPRTARLLARSG
FASPAPANSETNTLPHVAFYISVNRAISDEECTFNNSWLWKNEKGSRPFC
KDANISLIYRVNLERSLQYGIVGSATPDAKIVRISLDDDSTGAGIHLNDQ
LGYRQFGASYTTLDAYFREWSTDAIAQDYRFVFNASNNKAQILKTFPVDN
INEKFERKEV SGFELGVTGGVEVSGDGPKAKLEARASYTQSRWLTYNTQD
YRIERNAKNAQAVSFTWNRQQYATAESLLNRSTDALWVNTYPVDVNRISP
LSYASFVPKMDVIYKASATETGSTDFIIDSSVNIRPIYNGAYKHYYVVGA
HQSYHGFEDTPRRRITKSASFTVDWDHPVFTGGRPVNLQLASFNNRCIQV
DAQGRLAANTCDSQQSAQSFIYDQLGRYVSASNTKLCLDGEALDALQPCN
QNLTQRWEWRK GTDELTNVYSGESLGHDKQTGELGLYASSNDAVSLRTIT
AYTDVFNAQESSPILGYTQGKMNQQRVGQDHRLYVRAGAAIDALGSASDL
LVGGNGGSLSSVDLSGVKSITATSGDFQYGGQQLVALTFTYQDGRQQTVG
SKAYVTNAHEDRFDLPAAAKITQLKIWSDDWLVKGVQFDLN
> P17315|CIRA_ECOLI Colicin I receptor - Escherichia coli.
MFRLNPFVRVGLC LSAISCAWPVLAVDDDGETMVVTASSVEQNLKDAPAS
ISVITQEDLQRKPVQNLKDVLKEVPGVQLTNEGDNRKGVSIRGLDSSYTL
ILVDGKRVNSRNAVFRHNDFDLNWIPVDSIERIEVVRGPMSSLYGSDALG
GVVNIITKKIGQKWSGTVTVDTTIQEHRDRGDTYNGQFFTSGPLIDGVLG
MKAYGSLAKREKDDPQNSTTTDTGETPRIEGFSSRDGNVEFAWTPNQNHD
FTAGYGFDRQDRDS DSLDKNRLERQNYSVSHNGRWDYGTSELKYYGEKVE
NKNPGNSSPITSESNTVDGKYTLPLTAINQFLTVGGEWRHDKLSDAVNLT
GGTSSKTSASQYALFVEDEWRIFEPLALTTGVRMDDHETYGEHWSPRAYL
VYNATDTVTVKGGWATAFKAPSLLQLSPDWTSNSCRGACKIVGSPDLKPE
TSESWELGLYYMGEEGWLEGVESSVTVFRNDVKDRISISRTSDVNAAPGY
QNFVGFETGANGRRI PVFSYYNVNKARIQGVETELKIPFNDEWKLSINYT
YNDGRDVSNGENKPLSDLPFHTANGTLDWKPLALEDWSFYVSGHYTGQKR
ADSATAKTPGGYTIWNTGAAWQVTKDVKLRAGVLNLGDKDLSRDDYSYNE
DGRRYFMAVDYRF
> FABA_HAEIN P45159 3 -HYDROXYDECANOYL -[ACYL-CARRIER-PROTEIN]
MQNACTLNKKSSYSYDDLLASGRGELFGKEGPQLPAPTMLMM DRIIEMNE
ETGAFGKGYIEAELDIKPELPFFGCHFIGDPVMPGCLGLDAMWQLVGFYL
GWIGGKGKGRALGVGEVKFTGQILPTAKKVVYRIHMKRVINRKLVMGMAD
GEVEVDGRVIYTATDLKVGLFQDTSTF
> OTCA_PSESH Q02047 ORNITHINE CARBAMOYLTRANSFERASE, PHASEOLOT
MNARHFLSMMDYTPDELLGLIRRGVELKDLRIRGELFEPLKNRVLGMIFE
KSSTRTRLSFEAGMIQLGGQAIFLSHRDTQLGRGEPIADSAKVMSRMLDA
VMIRTYAHSNLTEFAANSRVPVINGLSDDLHPCQLLADMQTFLEHRGSIK
GKTVAWIGDGNNMCNSYIEAAIQFDFQLRVACPAGYEPNPEFLALAGERV
TIVRDPKAAVAGAHLVSTDVWTSMGQEEETARRMALFAPFQVTRASLDLA
EKDVLFMHCLPAHRGEEISVDLLDDSRSVAWDQAENRLHAQKALLEFLVA
PSHQRA
> DCP_SALTY P27236 PEPTIDYL -DIPEPTIDASE DCP (EC 3.4.15.5) (DI
MSTNPLLDQSMLPYQAPRFDRIKDCHYRPAFDEGVRQKRVEIEAIVNHPA
APDFTNTLLALEQSGALLSRVTSVFFAMTAAHTNDELQRLDEAFSAELAA
LSNDIYLNSALFARVDAVWQQRHSLGLDDESLRLVDVIHQRFVLAGAQLA
EEDKARLKVLNTESATLMSQFNQRLLAASKAGGLAVDDA HCLAGLSPEEM
TVAAEAAREKGLEERWFIPLLNTTQQPALATLRDRQTRENLFAASWTRAE
KGDAHDTRAIVQRLVEIRRCQAKLLGFPNYAAWKMADQMAKTPQAALSFM
RGIVPPARQRVLNEQAEIQNVIDGEQGGYTVQAWDWMFYAEQVRREKYAL
DEAQLKPYFALNTVLQEGVFWTANQLFGITFVERFDIPVYHPDVRVWEIF
DSDGVGMALFYGDFFARDSKSGGAWMGNFVEQSTLNETRP VIYNVCNYQK
PVDGQPALLLWDDVITLFHEFGHTLHGLFAVQRYATLSGTNTPRDFVEFP
SQINEHWASHPRVFERYARHVDSGEKMPADLQERMRKASLFNKGYDMTEL
LGAALLDMRWHMLEESVAEQSVAEFEQQALAAEHLDLPAVPPRYRSSYFA
HIFGGGYAAGYYAYLWTQMLADDGYQWFVEQGGLTRENGQRFRDAILARG
NSTDLETLYSAWRGHEPHIDPMLQYRGLDR
> P13650|DHGB_ACICA Quinoprotein glucose dehydrogenase -B - Acinetobacter
calcoaceticus.
MNKHLLAKIALLSAVQLVTLSAFADVPLTPSQFAKAKSENFDKKVILSNL
NKPHALLWGPDNQIWLTERATGKILRVNPESGSVKTVFQVPEIVNDADGQ
NGLLGFAFHPDFKNNPYIYISGTFKNPKSTDKELPNQTIIRRYTYNKSTD

TLEKPVDLLAGLPSSKDHQSGRLVI GPDQKIYYTIGDQGRNQLAYLFLPN
QAQHTPTQQELNGKDYHTYMGKVLRLNLDGSIPKDNPSFNGVVSHIYTLG
HRNPQGLAFTPNGKLLQSEQGPNSDDEINLIVKGGNYGWPNVAGYKDDSG
YAYANYSAAANKSIKDLAQNGVKVAAGVPVTKESEWTGKNFVPPLKTLYT
VQDTYNYNDPTCGEMTYICWPTVAPSSAYVYKGGKKAITGWENTLLVPSL
KRGVIFRIKLDPTYSTTYDDAVPMFK SNNRYRDVIASPDGNVLYVLTDTA
GNVQKDDGSVTNTLENPGSLIKFTYKAK
> P30125|LEU3_ECOLI 3-isopropylmalate dehydrogenase - Escherichia coli.
MSKNYHIAVLPGDGIGPEVMTQALKVLDAVRNRFAMRITTSHYDVGGAAI
DNHGQPLPPATVEGCEQADAVLFGSVGGPKWEHLPPDQQPERGALLPLRK
HFKLFSNLRPAKLYQGLEAFCPLRADIA ANGFDILCVRELTGGIYFGQPK
GREGSGQYEKAFDTEVYHRFEIERIARIAFESARKRRHKVTSIDKANVLQ
SSILWREIVNEIATEYPDVELAHMYIDNATMQLIKDPSQFDVLLCSNLFG
DILSDECAMITGSMGMLPSASLNEQGFGLYEPAGGSAPDIAGKNIANPIA
QILSLALLLRYSLDADDAACAIERAINRALEEGIRTGDLARGAAAVSTDE
MGDIIARYVAEGV
> P16869|FHUE_ECOLI FhuE receptor - Escherichia coli.
MLSTQFNRDNQYQAITKPSLLAGCIALALLPSAAFAAPATEETVIVEGSA
TAPDDGENDYSVTSTSAGTKMQMTQRDIPQSVTIVSQQRMEDQQLQTLGE
VMENTLGISKSQADSDRALYYSRGFQIDNYMVDGIPTYFESRWNLGDALS
DMALFERVEVVRGATGLMTGTGNPSAAINMVRKHATSREFKGDVSAEYGS
WNKERYVADLQSP LTEDGKIRARIVGGYQNNDSWLDRYNSEKTFFSGIVD
ADLGDLTTLSAGYEYQRIDVNSPTWGGLPRWNTDGSSNSYDRARSTAPDW
AYNDKEINKVFMTLKQQFADTWQATLNATHSEVEFDSKMMYVDAYVNKAD
GMLVGPYSNYGPGFDYVGGTGWNSGKRKVDALDLFADGSYELFGRQHNLM
FGGSYSKQNNRYFSSWANIFPDEIGSFYNFNGNFPQTDWSPQSLAQDDTT
HMKSLYAATRVTLA DPLHLILGARYTNWRVDTLTYSMEKNHTTPYAGLVF
DINDNWSTYASYTSIFQPQNDRDSSGKYLAPITGNNYELGLKSDWMNSRL
TTTLAIFRIEQDNVAQSTGTPIPGSNGETAYKAVDGTVSKGVEFELNGAI
TDNWQLTFGATRYIAEDNEGNAVNPNLPRTTVKMFTSYRLPVMPELTVGG
GVNWQNRVYTDTVTPYGTFRAEQGSYALVDLFTRYQVTKNFSLQGNVNNL
FDKTYDTNVEGSIVY GTPRNFSITGTYQF
> P26311|PEPT_SALTY Peptidase T - Salmonella typhimurium.
MDKLLERFLHYVSLDTQSKSGVRQVPSTEGQWKLLRLLKQQLEEMGLVNI
TLSEKGTLMATLPANVEGDIPAIGFISHVDTSPDFSGKNVNPQIVENYRG
GDIALGIGDEVLSPVMFPVLHQLLGQTLITTDGKTLLGADDKAGVAEIMT
ALAVLKGNPIPHGDIKVAFTPDEEVGKGAK HFDVEAFGAQWAYTVDGGGV
GELEFENFNAASVNIKIVGNNVHPGTAKGVMVNALSLAARIHAEVPADEA
PETTEGYEGFYHLASMKGTVDRAEMHYIIRDFDRKQFEARKRKMMEIAKK
VGKGLHPDCYIELVIEDSYYNMREKVVEHPHILDIAQQAMRDCHITPEMK
PIRGGTDGAQLSFMGLPCPNLFTGGYNYHGKHEFVTLEGMEKAVQVIVRI
AELTAKRGQ
> P45996|OMP53_HAEIN Outer membrane protein P5 - Haemophilus influenzae.
MKKTAIALVVAGLAAASVAQAAPQENTFYAGVKAGQGSFHDGINNNGAIK
KGLSSSNYGYRRNTFTYGVFGGYQILNQDNFGLAAELGYDDFGRAKLREA
GKPKAKHTNHGAYLSLKGSYEVLDGLDVYGKAGVALVRSDYKFYEDANGT
RDHKKGRHTARASGLFAVGAEYAVLPELAVRLEYQWLTRVGKYRPQDKPN
TAINYNPWIGCINAGISYRFGQGEAPVVAAPEMVSKTFSLNSDVTFAFGK
ANLKPQAQATLDSVYGEISQVKSRKVAVAGYTNRIGSDAFNVKLSQERAD
SVANYFVAKGVAADAISATGYGEANPVTGATCDQVKGRKALIACLAPDRR
VEIAVNGTK
> P21175|BRAC_PSEAE Leucine-, isoleucine-, valine-, threonine-, and alanine-binding protein - Pseudomonas aeruginosa.
MKKGTQRLSRLFAAMAIAGFASYSMAADTIKIALAGPVTGPVAQYGDMQR
AGALMAIEQINKAGGVNGAQLEGVIYDDACDPKQAVAVANKVVNDGVKFV
VGHVCSSSTQPATDIYEDEGVLMITPSATAPEITSRGYKLIFRTIGLDNM
QGPVAGKFIAERYKDKTIAVLHDKQQYGEGIATEVKKTVEDAGIKVAVFE
GLNAGDKDFNALISKLKKAGVQFVYFG GYHPEMGLLLRQAKQAGLDARFM
GPEGVGNSEITAIAGDASEGMLATLPRAFEQDPKNKALIDAFKAKNQDPS
GIFVLPAYSAVTVIAKGIEKAGEADPEKVAEALRANTFETPTGNLGFDEK
GDLKNFDFTVYEWHKDATRTEVK
> P13482|TREA_ECOLI Periplasmic trehalase - Escherichia coli.
MKSPAPSRPQKMALIPACIFLCFAALSVQAEETPVTPQPPDILL GPLFND

VQNAKLFPDQKTFADAVPNSDPLMILADYRMQQNQSGFDLRHFVNVNFTL
PKEGEKYVPPEGQSLREHIDGLWPVLTRSTENTEKWDSLLPLPEPYVVPG
GRFREVYYWDSYFTMLGLAESGHWDKVADMVANFAHEIDTYGHIPNGNRS
YYLSRSQPPFFALMVELLAQHEGDAALKQYLPQMQKEYAYWMDGVENLQA
GQQEKRVVKLQDGTLLNRYWDDRDTPRPESWVEDIATAKSNPNRP ATEIY
RDLRSAAASGWDFSSRWMDNPQQLNTLRTTSIVPVDLNSLMFKMEKILAR
ASKAAGDNAMANQYETLANARQKGIEKYLWNDQQGWYADYDLKSHKVRNQ
LTAAALFPLYVNAAAKDRANKMATATKTHLLQPGGLNTTSVKSGQQWDAP
NGWAPLQWVATEGLQNYGQKEVAMDISWHFLTNVQHTYDREKKLVEKYDV
STTGTGGGGGEYPLQDGFGWTNGVTLKMLDLICPKEQPCDNVPATR PTVK
SATTQPSTKEAQPTP
> P15214|GST_PROMI Glutathione S -transferase GST-6.0 - Proteus mirabilis.
MKLYYTPGSCSLSPHIVLRETGLDFSIERIDLRTKKTESGKDFLAINPKG
QVPVLQLDNGDILTEGVAIVQYLADLKPDRNLIAPPKALERYHQIEWLNF
LASEVHKGYSPLFSSDTPESYLPVVKNKLKSKFVYINDVLSKQKCVCGDH
FTVADAYLFTLSQWAPHVALDLTDLSHLQDYLARIAQRPNVHSALVTEGL
IKE
> P40130|CYAA_ERWCH Adenylate cyclase - Erwinia chrysanthemi.
MYFYIETLKQRLDAINQLRVDRALEAMKPAFQQVYSLLPVLLHHHHPLMP
GYLEGKVPHGICLFSPDEKQQHYLDSVELRWGELSAPDRKGELPITGVYS
MGSTSSIGQSCSSDLDIWVCHQSWLDNEERQLQQKCSLLEKWAAG QGVDV
SFFLMDENRFRHNESGSLGGEDCGSTQHILLLDEFYRTAVRMAGKRILWN
MVPVEEEAHYDEFVLSLYARGALAPNEWLDLGGLSALSAEEYFGASLWQL
YKSIDSPYKAVLKTLLLEAYSWEYPNTRLLSSEIKARLHKGEIVSFGLDP
YCMMLERVTQYLDAINDQTRLDLVRRCFYLKVCEKLSRERACTAWRRQIL
TQMVQAWGWSDERLVMLDNRANWKIGQVREAHNELLDAMMQSYRNL IRFA
RRNNLSVSASPQDIGVLTRKLYAAFEALPGKVTLVNPQISPDLSETNLTF
IYVPAGRANRSGWYLYNQAPSMDAIISHQPLEYNRYLNKLVAWAYFNGLL
TSSTRLHIKGHELCDIARLQELVSDVSSHFPLRVAAPTPKALYSPCEIRH
LAIIVNLEHDPTAAFRNQVVHFDFRQLDVFSFGQQQQCLVGSIDLLYRNS
WNEVRTLHFSGEQAMLEALKTILGKMHQDAALPESLEVFCYSQHLRG LIR
TRVQQLVSECIELRLSSTRQEPGRFKAVKVAGETWGLFFERLSVSAQKLE
NAVEFYGAISNNKLQGLPVQVETNHIHLPPVVDGVASEGIIQFFFEDQHD
NQGFNIYILDESNRVEVYHHCEGSKEELVRDVSRFYSSSHDRFTYGSSFI
NFNLPQFYQIVQLDGRTQVIPFRSSALSHLCVTPSSEDKKNLVLSQRLQM
L
> GLYA_CAMJE P24531 SERINE HYDROXYMETHYLTRANSF ERASE (EC 2.1.2
MSLEMFDKEIFDLTNKELERQCEGLEMIASENFTLPEVMEVMGSILTNKY
AEGYPGKRYYGGCEFVDEIETLAIERCKKLFNCKFANVQPNSGSQANQGV
YAALINPGDKILGMDLSHGGHLTHGAKVSSSGKMYESCFYGVELDGRIDY
EKVREIAKKEKPKLIVCGASAYARVIDFAKFREIADEVGAYLFADIAHIA
GLVVAGEHPSPFPHAHVVSSTTHKTLRGPRGGIIMT NDEELAKKINSAIF
PGIQGGPLMHVIAAKAVGFKFNLSDEWKVYAKQVRTNAQVLANVLMDRKF
KLVSDGTDNHLVLMSFLDREFSGKDADLALGNAGITANKNTVPGEIRSPF
ITSGLRLGTPALTARGFKEKEMEIVSNYIADILDDINNEKLQENIKQELK
KLASNFIIYERAMF
> P24092|HMWC_DESVH High-molecular-weight cytochrome c - Desulfovibrio
vulgaris (strain Hildenborough / ATCC 29579 / NCIMB 8303).
MRNGRTLLRWAGVLAATAIIGVGGFWSQGTTKALPEGPGEKRADLIEIGA
MERFGKLDLPKVAFRHDQHTTAVTGMGKDCAACHKSKDGKMSLKFMRLDD
NSAAELKEIYHANCIGCHTDLAKAGKKTGPQDGECRSCHNPKPSAASSWK
EIGFDKSLHYRHVASKAIKPVGDPQKNCGACHHVYDEASKKLVWGK NKED
SCRACHGEKPVDKRPALDTAAHTACISCHMDVAKTKAETGPVNCAGCHAP
EAQAKFKVVREVPRLDRGQPDAALILPVPGKDAPREMKGTMKPVAFDHKA
HEAKANDCRTCHHVRIDTCTACHTVNGTADSKFVQLEKAMHQPDSMRSCV
GCHNTRVQQPTCAGCHGFIKPTKSDAQCGVCHVAAPGFDAKQVEAGALLN
LKAEQRSQVAASMLSARPQPKGTFDLNDIPEKVVIGSIAKEYQPSEF PHR
KIVKTLIAGIGEDKLAATFHIEKGTLCQGCHHNSPASLTPPKCASCHGKP
FDADRGDRPGLKAAYHQQCMGCHDRMKIEKPANTACVDCHKERAK
> CILA_HAEIN P44459 CITRATE LYASE ALPHA CHAIN (EC 4.1.3.6) (C
MTTREQRIEKYHADRSVYQAVPKSESLSRTAKDRKLCTSLEEAIKRSGLK
DGMTVSFHHAFRGGDFVVNMVMNKIAEMGFKNLTLASSSLID SHFPIVEH
IKNGVVTKIYSSGLRGELAEQISRGLLNEPVNIHSHGGRVHLVKSGELKI

DVAFLGVPCCDTFGNANGFTGKSKCGSLGYARVDAEYADKVVLLTEEFVE
YPHHPISIAQDQVDLIVQVEAVGDPKKIGGGATRMTTNPRELLIARKCAE
VIFASGYFKDGFSLQTGSGGAALAVTRFLEEKMRRENITADFALGGITAS
MVALHEAGLIKKLLDVQSFDSVAAESLARNPNHIEVSANQYAN YSSKGAS
VERLDMVILSALEIDTKFNVNVLTGSDGVIRGASGGHCDTAASAQVAIIV
APLVRGRIPTVVENVITCVTPGENVDILVTDHGVAVNPKRPDLIEALSKT
DIPLFTIEQLCERAYSITGKPKEIEFTNKPVAVVRYRDGSVIDTVYQVKD

> P34895|GLYA_HYPME Serine hydroxymethyltransferase - Hyphomicrobium methylovorum.
MSSAPAAGTAS TSRFFKSHVSETDPDIFSAIQKEFGRQQHEIELIASENI
VSQAVLDAAGSVLTNKYAEGYPGKRYYGGCQYVDIVEDIAIDRAKKLFNC
EFANVQPNSGSQANQGVFNALAQPGDTILGLSLAAGGHLTHGAPVNQSGK
WFKAVHYMVKPDSHLIDMDEVRKLAQEHKPRIIIAGGSAYPRKIDFAAFR
AIADEVGAIFLVDMAHFAGLVAAGLIPSPFPHAHVVTTTTHKTLRGPRGG
MILTNDADIAKK INSAIFPGIQGGPLMHVIAGKAVAFGEALRPDFKVYIK
QVMDNARALGEVLVQNGFALVSGGTDTHLVLVDLRPKKLTGTKAEKALGR
ANITCNKNGIPFDPEKPMVTSGIRLGSPAGTTRGFGVAEFQEIGRLISEV
LDGVAKNGEDGNGAVEAAVKAKAIALCDRFPIYA
> P04805|SYE_ECOLI Glutamyl -tRNA synthetase - Escherichia coli.
MKIKTRFAPSPTGYLH VGGARTALYSWLFARNHGGEFVLRIEDTDLERST
PEAIEAIMDGMNWLSLEWDEGPYYQTKRFDRYNAVIDQMLEEGTAYKCYC
SKERLEALREEQMAKGEKPRYDGRCRHSHEHHADDEPCVVRFANPQEGSV
VFDDQIRGPIEFSNQELDDLIIRRTDGSPTYNFCVVVDDWDMEITHVIRG
EDHINNTPRQINILKALKAPVPVYAHVSMINGDDGKKLSKRHGAVSVMQY
RDDGYLPEALLNYLVRL GWSHGDQEIFTREEMIKYFTLNAVSKSASAFNT
DKLLWLNHHYINALPPEYVATHLQWHIEQENIDTRNGPQLADLVKLLGER
CKTLKEMAQSCRYFYEDFAEFDADAAKKHLRPVARQPLEVVRDKLAAITD
WTAENVHHAIQATADELEVGMGKVGMPLRVAVTGAGQSPALDVTVHAIGK
TRSIERINKALDFIAERENQQ
> P48515|IF2_THET8 Translation initiation facto r IF-2 - Thermus thermophilus (strain HB8 / ATCC 27634 / DSM 579).
MAKVRIYQLAKELGMETQELLELLDQMGVAYKSHASTLEEKDAEAVRELV
KEQRGLQEKLAEEERRKSLPRRPPVVVIMGHVDHGKTTLLDYLRKSRIAE
KEAGGITQHVGAFEVKTPQGTVVFIDTPGHEAFTTIRQRGAKVADIAVIV
IAADDGIMPQTEEAIAHAKAAGAKLIFAINKIDLPQ ADPEKVKRQLMERG
FVPEEYGGDAIVIPISAKTGQGVQDLLEMILLLAELEDYRADPNAEPRGV
ILESKLDKQAGIIANMLVQEGTFRVGDYVVAGEAYGRIRAMMDADGNQRK
EAGPGSAVQVLGFQELPHAGDVVEWVPDLEAAKEIAEERKEERKAREEEE
KARRPRTMAELLRAMQEEGRKELNLILRADTQGSLEAIQHILARESTEDV
KINILLAQVGAPTESDVLLAQTANAAILAFGVNPPGS VKKKAEEKGVLLK
TFRIIYDLVDEVRNMVKGQREPQYKEEVLGQAEVRAIFRLPTGKQVAGCM
VTQGRIPRNAEVRVLRDGQVIWQGRIASLKRFKEDVREVAQGYECGIGLD
GFDDFREGDVIEAFQMVEVPA
> ACEK_SALTY P51067 ISOCITRATE DEHYDROGENASE KINASE/PHOSPHATA
MPRGLELLIAQTILQGFDAQYGRFLEVTSGAQQRFEQADWHAVQQAMKSR
IHLYDHHVGLVVEQLRCITDGKSTDANFLLRVKEHYTRLLPDYPRFEIAE
SFFNSVYCRLFDHRSLTPERLFIFSSQPERRFRTIPRPLAKDFFPDHGWE
PLLMRILSDLPLRLPWQNKRRDIRYIIAHLTEALGEDALPRCHVQVANEL
FYRNKAAWLVGKLTTPDGTLPFLLPIHRTDEGELFVDTCLTTTAEASIVF
GFARSYFMVYAPLPAALVEWLREILPGKTTAELYMAIGCQKHAKTESYRE
YLCYLAESDEKFIEAPGIRGMVMLVFTLPGFDRVFKIIKDKFAPQKEMSA
AHVRACYQLVKEHDRVGRMADTQEFENFVLDKRQIDPALMALLRQEAPEK
ITDLGEQIVIRHLYIERRMVPLNIWLEQVEGQQLRDAIEEYGNAIRQLAA
ANIFPGDMLFKNFGVTRHGRVVFYDYDEICYMTEVNFRDIPPARYPEDEL
ASEPWYSVSPGDVFPEEFRHWLCADPRIGPLFEEMHADLFRADYWRALQT
RIKEGHVEDVYAYRRRQRFSVRYGAISSTVNSS
> P0C0X7|C554_RHOSH Cytochrome c -554 - Rhodobacter sphaeroides (Rhodopseudomonas sphaeroides).
QDARQIERMIEGRHGLMTLMAYELGKLGGMAKEETPYDAEVAGDAASNLS
ALASVLSPELFPKGSAVGEAEDSEALPAIWEKPDDFAQKISDMEEAAAKM
QAAAGTDLASLQGAMRDLGAGCGSCHETYRQK D
> P44442|RND_HAEIN Ribonuclease D - Haemophilus influenzae.

MSKWDSNIPFLFFSNKKITMIKECQNPPHFRVVTDNTALLEVCNLAQQKS
AVALDTEFMRVSTYFPKLGLIQLYDGEHVSLIDPLAITDFSPFVALLANP
KVLKILHSCSEDLLVFLQEFDQLPRPMIDTQIMARFLGLGTSAGLAKLAQ
QYLNVEIDKGATRTNWIKRPLSDIQLQYAAGDVWYLLPLYH ILEKELAKT
PWEQAVRDDCELVLAKTHKLQERDSEKAYLDIPNAWKLNPLELSRLRVLA
QWRQNVAIERDLALSYIVKSEHLWKVAKNNPRNTSEMLEMGLTENEVRVR
GKEILQLLSQARRISSNDYPKSIERISEDPRYKKTIRLLQEKVNSLTPEG
LTPEIVASKRTLEELIKWVWKYDCSQDKRPELLIGWRKPIGEKLVDALK
> NIRF_PSEST Q52521 NIRF PROTEIN. - PSEUDOMONAS STUTZERI (PSE
MIRPFLLLAAVGLLTACAQQPLRGTGDLGVVVERATGSLQIIESSNQSQI
ARIEGLGDLSHASVVFSRDQRYAYVFGRDGGLTKVDLLRHRIDRRVIQGG
NSIGGAISQDGTLIAVGNYEPGGVKVFDAKTLELVADIPATPLADGSRNA
RVVGVIDVPGRRFIYSLFDTDETWLLDFSQGNEPQITRFEGIGRQPYDAL
LTPEGRYYIAGLFGEDGMAKIDLWHPERGVERI LDGYGRGEQKLPVYKMP
HLEGWTVAGNQTFVPAVGQHRVLVMDSQNWQQTAAIDVAGQPIFVMARPD
ARQIWVNFAHPDNHRVQVIDSETHEIIADLEPGPAVLHMEFTARGDQLWL
SVRDGEEIQVWDPYTLKLLKRLPAHSPSGIFFSSRAHETGL
> P07110|PAPC_ECOLI Outer membrane usher protein papC - Escherichia coli.
MKDRIPFAVNNITCVILLSL FCNAASAVEFNTDVLDAADKKNIDFTRFSE
AGYVLPGQYLLDVIVNGQSISPASLQISFVEPALSGDKAEKKLPQACLTS
DMVRLMGLTAESLDKVVYWHDGQCADFHGLPGVDIRPDTGAGVLRINMPQ
AWLEYSDATWLPPSRWDDGIPGLMLDYNLNGTVSRNYQGGDSHQFSYNGT
VGGNLGPWRLRADYQGSQEQSRYNGEKTTNRNFTWSRFYLFRAIPRWRAN
LTLGENNINSDIFRSWSYTGA SLESDDRMLPPRLRGYAPQITGIAETNAR
VVVSQQGRVLYDSMVPAGPFSIQDLDSSVRGRLDVEVIEQNGRKKTFQVD
TASVPYLTRPGQVRYKLVSGRSRGYGHETEGPVFATGEASWGLSNQWSLY
GGAVLAGDYNALAAGAGWDLGVPGTLSADITQSVARIEGERTFQGKSWRL
SYSKRFDNADADITFAGYRFSERNYMTMEQYLNARYRNDYSSREKEMYTV
TLNKNVADWNTSFNLQYSRQTY WDIRKTDYYTVSVNRYFNVFGLQGVAVG
LSASRSKYLGRDNDSAYLRISVPLGTGTASYSGSMSNDRYVNMAGYTDTF
NDGLDSYSLNAGLNSGGGLTSQRQINAYYSHRSPLANLSANIASLQKGYT
SFGVSASGGATITGKGAALHAGGMSGGTRLLVDTDGVGGVPVDGGQVVTN
RWGTGVVTDISSYYRNTTSVDLKRLPDDVEATRSVVESALTEGAIGYRKF
SVLKGKRLFAILRLADGSQPPFG ASVTSEKGRELGMVADEGLAWLSGVTP
GETLSVNWDGKIQCQVNVPETAISDQQLLLPCTPQK
> P45573|DSVC_DESVH Sulfite reductase, dissimilatory -type subunit gamma - Desulfovibrio vulgaris (strain Hildenborough / ATCC 29579 / NCIMB 8303).
MAEVTYKGKSFEVDEDGFLLRFDDWCPEWVEYVKESEGISDISP DHQKII
DFLQDYYKKNGIAPMVRILSKNTGFKLKEVYELFPSGPGKGACKMAGLPK
PTGCV
> ASRA_SALTY P26474 ANAEROBIC SULFITE REDUCTASE SUBUNIT A (AN
MAIKITPDEFSLLIQRLNKKWRVFAPSAEFRGGRFSDTDNIIYQRISGWR
DLIWHEKSHMSPNTIIAPITETLFYFDKDTIQIAETDTSPIIIFARACDI
NAMSRLDYMYLSNGNNSDYSYQLLREHI RFVLIECEESFENCFCVSMGTN
KTDCYSAAMRFSDEGALVSIRDPFIEAAIQGLGQEADYTPSFVSENRETV
VTPDSVCHDPQKIRDILTHHPLWDAYDSRCISCGRCTTGCPTCTCYSVFD
VAYDENPQRGERRRQWASCMVPGFSDMAGGHGFREKPGERLRYRALHKVN
DYKARNGIEHMCVGCGRCDDRCPQYIKFSLIINKMTAAVRQALAEEA
> P56145|SYFB_HELPY Phenylalanyl -tRNA synthetase beta chain - Helicobacter pylori (Campylobacter pylori).
MKLSINDLNVFVNTPKDIAKLCEDLSRLGLEVESCIPCIAPKNVVVGKIL
EKAPHKNAEKLSVCQVDVGKEVLQIVCGAKNVAPNQFVPVALNGALIGST
TIAKTELRGVESHGMICSSIELGFPKINDGILELDESVGELVLGKELNEY
APFNTHVLEISLTPNRGDCLSVLGIAREI SAFYHTPLKPIKALNFTPKSG
LITLSAGENIESHLAYYLICNHSLKTPLNIKLSLAHNNALSENDLNNFIE
FSTHFSGVIMNAYSLNTTPMDLSVKNDENNLESVYINHQKRSTIAIKHQV
QKDLSECLLLEASYTDPISLSLKLHALKDKTLQKDNALIYRSARGSNPNL
SDGLNFLSAHLKATILESKQTEHSLKDRTLTFQLEDITEILGLAVEKEKI
QGILKNLGFKVSVKEPNSKPQILEVIAPNF RHDIKTIQDIAEEILRFVGI
DNLVSKPLHCVSSKNSNPNYDTHRFFENLKHKALACGFKEVIHYVFYSKE
KQQKLGFEVLEDPLELQNPITTELNTLRTSLVCGLLDASLRNKNLGFKSI
ALYEKGSVYNSKREEIQKLGFLISGLQKKESYPDTKGKAWDFYSFAECVS
KVIGDFSLEKLTTQTPINHPYQSAKIIQNHEIIGVIAKIHPKVIQELDLF

ESYYAEIDAFKLKRPAMLLKPFSIYPSSVRD LTLIIDENTAFSGIKKALK
DAQIPNLSEILPLDIFKESNNSIALSVRCVIHSLEKTLNDEEVNSAVQKA
LEILEKEFNARLKG
> P34945|SYS_THET2 Seryl -tRNA synthetase - Thermus thermophilus (strain HB27 / ATCC BAA -163 / DSM 7039).
MVDLKRLRQEPEVFHRAIREKGVALDLEALLALDREVQELKKRLQEVQTE
RNQVAKRVPKAPPE EKEALIARGKALGEEAKRLEEALREKEARLEALLLQ
VPLPPWPGAPVGGEEANREIKRVGGPPEFSFPPLDHVALMEKNGWWEPRI
SQVSGSRSYALKGDLALYELALLRFAMDFMARRGFLPMTLPSYAREKAFL
GTGHFPAYRDQVWAIAETDLYLTGTAEVVLNALHSGEILPYEALPLRYAG
YAPAFRSEAGSFGKDVRGLMRVHQFHKVEQYVLTEASLEASDRAFQELLE
NAEEILRLLELPYRL VEVATGDMGPGKWRQVDIEVYLPSEGRYRETHSCS
ALLDWQARRANLRYRDPEGRVRYAYTLNNTALATPRILAMLLENHQLQDG
RVRVPQALIPYMGKEVLEPCG
> P04981|TOX5_BORPE Pertussis toxin subunit 5 - Bordetella pertussis.
MQRQAGLPLKANPMHTIASILLSVLGIYSPADVAGLPTHLYKNFTVQELA
LKLKGKNQEFCLTAFMSGRSLVRACL SDAGHEHDTWFDTMLGFAISAYAL
KSRIALTVEDSPYPGTPGDLLELQICPLNGYCE
> P94282|SYFA_BORBU Phenylalanyl -tRNA synthetase alpha chain - Borrelia burgdorferi (Lyme disease spirochete).
MKVIFMKADLNLIKTLHPLEIKVIVNNEEEDDISASIIIEKLGFNEGQAN
KTIEWLNSKGIIEEIYRKLNVFYKATERGLGALRD GFVEEKIINLVSQKA
VLASNLALELDIDVKEVRKAFGNLLKEGILSLDLDKQIIINCLDGTETNY
QKVRVLLERAKNSDLLRESLTTEELLLISNFAKKKGADSVFFKIIEKLDL
KFRLSSFGLEVKNFLMKSKLTGDELTKLTPEILKNKTYENKKFRAYNIHI
PSAKTFIGRANSYLDYISKIKDKLVGLGFQEFDGPLVETEFFNNDALFMP
QFHPSRDIKDVYYISDPSMQESLPEPYFSNVKLAHE TGYATGSRGWRYSF
SEDLSKRLVLRTHGTVLSAKQLINAKNPSRYFGVIRCFRYDQVDATHGVD
FYQTEGIVIEDGVSIKTLLGLLEIFAKELAGATEIKYVPAYFPFTEPSIE
IHVKHPVLGWFELGGSGIFRPEVTKPLGIDLPVIAWGIGIDRMALMHLGL
NDLRDLFTYDIGDVILRRGKIDAKVRNL
> P04738|FMKA_ECOLI K88 fimbrial protein A - Escherichia coli.
MKKAFLLACVFFLTGGGVSHAAVQKTIFSADVVASVCHVVVDADSTGNSG
RLTFGTYRKSTGASVPPRDFTVRLYESGATVQGCSAFLAGQVATLDFGNP
GQLDAAGVVTRGAGDGIRVDVRAVDAQADYRGRLTQDNHSVKYPVDFAAK
GQFRFRAQPVFPADVKAGEYSGALTFVVTYQ
> P14898|AMY2_DICTH Alpha -amylase 2 - Dictyoglomus thermophilum.
MIYDDKIFGDLCHKEFLVEREVKKLEEIYLEEVLPEDPKPEDEIEFTFNC
PLKFHITSGKIVKDNREIYTFNIQERKTQWNDSIFNFSEIIKIKIPPLKE
NGLYQIHLYEMNEKIYEQYLSIDSFEAPLWSEESIIYHIFIDRFAKDEKE
VEYSENLKEKLGGNLKGILSRLDYIENLGINTIWISPIFKSTSYHGYDIE
DYFEIDPIWGTKEDLKKLVREAFNRGIRIILDFVPNHMSYKNPIFQKALK
DKNSNLRSWFIFKGEDYETFFGVKSMPKINLKNKEAIDYIINAAKYWIRE
FGISGYRMDHATGPDINFWSIFYYNLKSEFPETFYFGEIVETPKETKKYV
GKFDGTLDFYLFKIIRDFFIGKRWSTKEFVKMIDLEEKFYGNKFKRISFL
ENHDSNRFLWVAKDKKLLRLASIFQFSINAIPIIYNGQEMGCSQYRDILE
GNRTLHEHARLPIPWSDDKQDKELIDFYRQLVKIRKSHPALYKGTFIPIF
SDMISFIKETQEESILVLINIEDKEEIFNLNGTYRDLFSGNIYTNSLKLG
PMSAHLLLRIDH
> P02943|LAMB_ECOLI Maltoporin - Escherichia coli.
MMITLRKLPLAVAVAAGVMSAQAMAVDFHGYARSGIGWTGSGGEQQCFQT
TGAQSKYRLGNECETYAELKLGQEVWKEGDKSFYFDTNVAYSVAQQNDWE
ATDPAFREANVQGKNLIEWLPGSTIWAGKRFYQRHDVHMIDFY YWDISGP
GAGLENIDVGFGKLSLAATRSSEAGGSSSFASNNIYDYTNETANDVFDVR
LAQMEINPGGTLELGVDYGRANLRDNYRLVDGASKDGWLFTAEHTQSVLK
GFNKFVVQYATDSMTSQGKGLSQGSGVAFDNEKFAYNINNNGHMLRILDH
GAISMGDNWDMMYVGMYQDINWDNDNGTKWWTVGIRPMYKWTPIMSTVME
IGYDNVESQRTGDKNNQYKITLAQQWQAGDSIWSRPAIRVFATY AKWDEK
WGYDYTGNADNNANFGKAVPADFNGGSFGRGDSDEWTFGAQMEIWW
> P05706|PTHA_ECOLI Glucitol/sorbitol -specific phosphotransferase enzyme IIA component - Escherichia coli.
MTVIYQTTITRIGASAIDALSDQMLITFREGAPADLEEYCFIHCHGELKG
ALHPGLQFSLGQHRYPVTAVGSVAEDNLRELGHVTLRFDGLNEA EFPGTV

HVAGPVPDDIAPGSVLKFESVKE
> P45354|HXUA2_HAEIN Heme/hemopexin -binding protein - Haemophilus influenzae.
MYKLNVISLIILTTCSGAAYASTPDFPQHHKTVFGTVTIEKTTADKMTIK
QGSDKAQIDWKSFDIGQKKEVKFEQPNEHAVAYNRVIGGNASQIQGKLTA
NGKVYLANPNGVIITQGAEINVAGLLATTKDLERISENSNSYQFT RRTKD
RQVLKEGLVLKDGQVVKEGQVINEGNITAQDFVVLNGDEVINKGNINVEK
NSTINGKVYLSSGYNFTFTLPDSGISVALEDNTVQGIVKNEGSIKAGEIT
LSAKGRKQALDSLVMNNGVLEATKVSNKNGKVVLSADNVELNNESNIKGE
IVTFGADVTSNKELKDNIKITSKTGSKVTSPKINFTGKSVNINGNFGRED
STTHYKDEFKKLNTEVNIDVPDNENIRIADIEDNTGTGTTGTGTSS FIQT
GALSSLLANNGKVNLKGNNVNISGRIHIDSFRGSDSLLKLTNKGHIDINN
ADIHSKGRLFFITSLQNEEDFKSNITITDSKINLGNGAMGLGRSVDEKDY
DNRWQKTEGSQRKKFDVKMSNVEFNQVDDVILAGGFEKVNLDKIVATGQT
NFYIDGGVSRNGRKYEYGVLDLDKRTQLSELNQGRRRWGYYYDLELDMNR
AYLYRFDLFATKNTGRSTIKDTEINISNSNINLKNGFVHLLAEKIKL DNS
KIDITFDKDNSQDTLAQTNRLGMNGKVSMINSHIKIVGDEKEGISPTGTY
ATMFLIGELIGEKSSIFVKSHQGYTFKTDGNTKIAGKYSKEDLKITAINT
GGRAAEEVLINGALGSADNDANIANMAFTIGDSANTKTTIENADITALAP
NGGTAYLSSKDVEIEVKPNSNFTFFELPREKNLNQTKINGASTKLSERGF
ARLYDKINGVRASNLSAEQLNVTDASEKIINTKLVSSLDVEKLVSVAV CD
AGNGCEEQQFGDKGNNTKVSVGELEAEQ
> P0AGJ9|SYY_ECOLI Tyrosyl -tRNA synthetase - Escherichia coli.
MASSNLIKQLQERGLVAQVTDEEALAERLAQGPIALYCGFDPTADSLHLG
HLVPLLCLKRFQQAGHKPVALVGGATGLIGDPSFKAAERKLNTEETVQEW
VDKIRKQVAPFLDFDCGENSAIAANNYDWFGNMNVLTFLRDIGKHFSVNQ
MINKEAVKQRLNREDQGISFTEFSYNLLQGYDFACLNKQYGVVLQIGGSD
QWGNITSGIDLTRRLHQNQVFGLTVPLITKADGTKFGKTEGGAVWLDPKK
TSPYKFYQFWINTADADVYRFLKFFTFMSIEEINALEEEDKNSGKAPRAQ
YVLAEQVTRLVHGEEGLQAAKRITECLFSGSLSALSEADFEQLAQDGVPM
VEMEKGADLMQALVDSELQPSRGQARKTIASNAITINGEKQSDPEYFFKE
EDRLFGRFTLLRRGKKNYCLICWK
> P0C0T5|MEPA_ECOLI Penicillin -insensitive murein endopeptidase - Escherichia coli.
MNKTAIALLALLASSASLAATPWQKITQPVPGSAQSIGSFSNGCIVGADT
LPIQSEHYQVMRTDQRRYFGHPDLVMFIQRLSSQVSNLGMGTVLIGDMGM
PAGGRFNGGHASHQTGLDVDIFLQLPKTRWTSAQLLRPQALDLVSRDGKH
VVSTLWKPEIFSLIKLAAQDKDVTRIFVNPAIKQQLCLDAGTDRDWLRKV
RPWFQHRAHMHVRLRCPADSLECEDQPLPPSGDGCGAELQSWFEPPKPGT
TKPEKKTPPPLPPSCQALLDEHVI
> EFG2_SYNY3 P74228 ELONGATION FACTOR G 2 (EF -G 2). - SYNECHO
MARTVPLERIRNIGIAAHIDAGKTTTTERILFYSGVVHKIGEVHEGTAVT
DWMAQERERGITITAAAIS TDWLGHHINIIDTPGHVDFTIEVERSMRVLD
GVIAVFCSVGGVQPQSETVWRQAERYQVPRIAFVNKMDRTGANFFRVCQQ
IGDRLRANAVPVQIPIGSEAEFEGIVDLVRMKAYLYKNDLGTDIQEVPIP
DSVKDKTEEYRLRLVESVAEADDALMEKYLEGEELTADELVAGLRRGTIA
GTMVPVLCGSAFKNKGVQLLLDAVVDYLPSPLEVPAIEGHLPDGEVATRP
AEDKAPLSALAFKVMADPFG RLTFVRVYSGVLEKGSYVLNSTKEKKERIS
RLIIILKADDRIEVDQLNAGDLGAVLGLKDTLTGDTLCDDQEPIILESLFV
PQPVISVAVEPKTKQDMDKLSKALQSLSEEDPTFRVSVDPETNQTVIAGM
GELHLEILVDRMLREFKVEANVGAPQVAYRETIRKAVQAEGKFIRQSGGK
GQYGHVVIEVEPTEPGTGFEFVSKIVGGVIPKEYIAPSEQGMKEACASGV
LAGYPVIDLKATLVDGSFHDV DSSEMAFKIAGSMAIREAVGQADPVLLEP
VMKVEIEVPDDFMGNVIGDLNARRGHIEGQETEQGIAKVAASVPLAEMFG
YATDIRSKTQGRGIFSMEFSHYAEVPRNVAEAIVAKSRGYA
> Q04064|OMP_BORPE Outer membrane porin protein BP0840 - Bordetella pertussis.
MKKTLLAAALLAGFAGAAQAETSVTLYGIIDTGIGYNDVDFKVKGANADD
SDFKYNHSRFGMINGVQNGSRWGLRGTEDLGDGLQAVFQLESGFNSGNGN
SAQDGRLFGRQATIGLQSESWGRLDFGRQTNIASKYFGSIDPFGAGFGQA
NIGMGMSAMNTVRYDNMVMYQTPSYSGFQFGIGYSFSANDKDADAVNRVG
FATADNVRAITTGLRYVNGPLNVALSYDQLNASNNQAQGEVDATPRSYGL
GGSYDFEVVKLALAYARTTDGWFGGQGYPVAVTLPSGDKFGGFGVNTFAD

GFKANSYMVGLSAPIGGASNVFGSWQMVDPKLTGGDEKMNVFSLGYTYDL
SKRTNLYAYGSYAKNFAFLEDAKSTAVGVGIRHRF
> P0ABW7|FMS1_ECOLI CS1 fimbrial subunit A - Escherichia coli.
MKLKKTIGAMALATLFATMGASAVEKTISVTASVDPTVDLLQSDGSALPN
SVALTYSPAVNNFEAHTINTVVHTNDSDKGVVVKLSADPVLSNVLNPTLQ
IPVSVNFAGKPLSTTGITIDSNDLNFASSGVNKVSSTQKLSIHADATRVT
GGALTAGQYQGLVSIILTKST
> Q05092|XYLS2_PSEPU XylDLEGF operon transcriptional activator 2 -
Pseudomonas putida.
MSMTLVAEHYTKIIATKLLETLSSNVSRKGFVEGNPCFERVVQFIEENVK
RSISLEQLAELALMSPRSLYTMFEKHTGTTPMNYIRNRKLECVRACLSNP
TTNIRSITEVALDYGFLHLGRFAEKYRSTFGELPSDTLSLHKMKCIDSRE
SSLSSLF
> Q06529|CYSD_CHRVI Cytochrome subunit of sulfide dehydrogenase -
Chromatium vinosum (Allochromatium vinosum).
MTQSTPRLMLAASVLALGLASNAGAEPTAEMLTNNCAGCHGTHGNSVGPA
SPSIAQMDPMVFVEVMEGFKSGEIASTIMGRIAKGYS TADFEKMAGYFKQ
QTYQPAKQSFDTALADTGAKLHDKYCEKCHVEGGKPLADEEDYHILAGQW
TPYLQYAMSDFREERRPMEKKMASKLRELLKAEGDAGLDALFAFYASQQ
> P72181|NIRS_PARPN Nitrite reductase - Paracoccus pantotrophus
(Thiosphaera pantotropha).
MRQRTPFARPGLLASAALALVLGPLAASAQEQVAPPKDPAAALEDHKTRT
DNRYEPSLDNLAQQDVAAPGAPEGVSALSDAQYNEANKIYFERCAGCHGV
LRKGATGKALTPDLTRDLGFDYLQSFITYGSPAGMPNWGTSGELSAEQVD
LMANYLLLDPAAPPEFGMKEMRESWKVHVAPEDRPTQQENDWDLENLFSV
TLRDAGQIALIDGATYEIKSVLDTGYAVHISRLSASGRYLFVIGRDGKVN
MIDLWMKEPTTVAEIKIGSEARSIETSKMEGWEDKYAIAGAYWPPQYVIM
DGETLEPKKIQSTRGMTYDEQEYHPEPRVAAILASHYRPEFIVNVKETGK
ILLVDYTDLDNLKTTEISAERFLHDGGLDGSHRYFITAANARNKLVVIDT
KEGKLVAIEDTGGQTPHPGRGANFVHPTFGPVWATSHMGDDSVALIGTDP
EGHPDNAWKILDSFPALGGGSLFIKTHPNSQYLYVDATLNPEAEISGSVA
VFDIKAMTGDGSDPEFKTLPIAEWAGITEGQPRVVQGEFNKDGTEVWFSV
WNGKDQESALVVVDDKTLELKHVIKDERLVTPTGKFNVYNTMTDTY
> P0A9K9|SLYD_ECOLI FKBP-type peptidyl-prolyl cis-trans isomerase slyD -
Escherichia coli.
MKVAKDLVVSLAYQVRTEDGVLVDESPVSAPLDYLHGHGSLISGLETALE
GHEVGDKFDVAVGANDAYGQYDENLVQRVPKDVFMGVDELQVGMRFLAET
DQGPVPVEITAVEDDHV VVDGNHMLAGQNLKFNVEVVAIREATEEELAHG
HVHGAHDHHHDHDHDGCCGGHGHDHGHEHGGEGCCGGKGNGGCGCH
> LPSA_PASHA Q05770 LPSA PROTEIN. - PASTEURELLA HAEMOLYTICA.
MNNYVISLTSAQERRKHIEAEFGKQNIPFQFFDAITPDLIKEKAKAFNID
ISNTNLTKGEIACALSHIALWHLAKQQNLDYICIFEDDIYLGNNAFELLK
TNYIPENTHIVK LETLPFDRINRFNKTEKYILNRRLFKLNSRHVGTAGYI
LTNKGAEFLINILKTLNIPIDDLIFDEYLKIKEYKVLQMSPALCVQDFIL
NSKTNFKSSLQDDRAIRCNNEDKIKNQAKLKKPNYFLTKIQKELYRPFKQ
LKQKVFTTYITFK
> P44430|DEOC_HAEIN Deoxyribose-phosphate aldolase - Haemophilus influenzae.
MTSNQLAQYIDHTALTAEKNEQDI STLCNEAIEHGFYSVCINSAYIPLAK
EKLAGSNVKICTVVGFPLGANLTSVKAFETQESIKAGANEIDMVINVGWI
KSQKWDEVKQDIQAVFNACNGTPLKVILETCLLTKDEIVKACEICKEIGV
AFVKTSTGFNKGGATVEDVALMKNTVGNIGVKASGGVRDTETALAMIKAG
ATRIGASAGIAIISGTQDTQSTY
> P12267|FM3_KLEPN Fimbrial subunit type 3 - Klebsiella pneumoniae.
MKKVLLSAAMATAFFGMAAANAADTNVGGGQVNFFGKVTDVSCTVSVNGQ
GSDANVYLSPVTLTEVKAAAADTYLKPKSFTIDVSDCQAADGTKQDDVSK
LGVNWTGGNLLAGATAKQQGYLANTEAAGAQNIQLVLSTDNATALTNKII
PGDSTQPKAAGDASAVQDGARFTYYVGYATSTPTTVTTGVVNSYATYEIT
YQ
> P0C1C0|PEL1_ERWCA Pectate lyase 1 - Erwinia carotovora.
MKYLLPSAAAGLLLLAAQPTMAANTGGYATTDGGDVSGAVKKTARSLQEI
VDIIEAAKKDSSGKAVKGGAYPLVITYNGNEDALIKAAEANICGQWSKDP
RGVEIKEFTKGITILGTNGSSANFGIWMVNSSNVVVRNMRFGYMPGGAKD
GDAIRIDNSPNVWIDHNEIFAKNFECAGTPDNDTTFESAVDIKKGATNVT

VSYNYIHGVKKVGLSGSSNTDTGRDLT YHHNIYSDVNSRLPLQRGGKVHA
YNNLYDGIKSSGFNVRQKGIALIESNWFENALNPVTARNDDSNFGTWELR
NNNITSPSDFAKYKITWGKPSTPHINADDWKSTGKFPAVPYSYSPVSAQC
VKDKLASYAGVGKNLAVLTAANCK
> P75948|THIK_ECOLI Thiamine kinase - Escherichia coli.
MPFRSNNPITRDELLSRFFPQYHPVTTFNSGLSGGSFLIEHQGQRFVVR Q
PHDPDAPQSAFLRQYRALSQLPACIAPKPHLYLRDWMVVDYLPGAVKTYL
PDTNELAGLLYYLHQQPRFGWRITLLPLLELYWQQSDPARRTVGWLRMLK
RLRKAREPRPLRLSPLHMDVHAGNLVHSASGLKLIDWEYAGDGDIALELA
AVWVENTEQHRQLVNDYATRAKIYPAQLWRQVRRWFPWLLMLKAGWFEYR
WRQTGDQQFIRLADDTWRQLLIKQ
> P10641|OPP12_HAEIN Oute r membrane protein P1 - Haemophilus influenzae.
MKKFNQSLLATAMLLAAGGANAAAFQLAEVSTSGLGRAYAGEAAIADNAS
VVATNPALMSLFKTAQFSTGGVYIDSRINMNGDVTSYAQIITNQIGMKAI
KDGSASQRNVVPGAFVPNLYFVAPVNDKFALGAGMNVNFGLKSEYDDSYD
AGVFGGKTDLSAINLNLSGAYRVTEGLSLGLGVNAVYAKAQVERNAGLIA
DSVKDNQITSALSTQQEPFRDLKKYLPSKDKSVVSLQDRAAWGFGWNAGV
MYQFNEANRIGLAYHSKVDIDFADRTATSLEANVIKEGKKGNLTFTLPDY
LELSGFHQLTDKLAVHYSYKYTHWSRLTKLHASFEDGKKAFDKELQYSNN
SRVALGASYNLYEKLTLRAGIAYDQAASRHHRSAAIPDTDRTWYSLGATY
KFTPNLSVDLGYAYLKGKKVHFKEVKTIGDKRTLTLNTTANYTSQAHANL
YGLNLNYSF
> P55995|LON_HELPY ATP -dependent protease La - Helicobacter pylori
(Campylobacter pylori).
MTEDFPKILPLLVEEDTFLYPFMIAPIFLQNNASIKAVAYAKNNKSLVFI
ACQKDKLNDNEAPYYDVGVIGSVMREANMPNGRVKLLFNGIAKGRILEPA
KENEQGFLEAQISPIEYLEYDKENIQAIVEVLKEKVITLANVSSLFPPDL
IKALEDNDDPNRIADLIAAALHLKKDQAYSLFANNNTEQRLLDLIDIVIE
ETKTQKLQKEIKSKVHQKMEQTNKEYFLKEQLKQIQKELGTDKQRDEDLN
QYYQKLESIKPFLKEEAFKEIKKQIDRLSRTHADSSDSATLQNYIETMLD
VPFGQYGKKALDIKHVREQLDKDHYSLKRPKERIVEYFATMQLLEMRRKK
KPEKKDKTKGTILCFYGPPGVGKTSLANSIAKAIERPLVRIALGGLEDVN
ELRGHRRTYIGSMPGRIVQGLIEAKKMNPVMVLDEIDKVDRSVRGDPASA
LLEILDPEQNTAFRDHYANFSIDLSQVIFIATANNIDRIPAPLRDRMEFI
SVSSYTPNEKEEIAKNYLIPQELEKHALKPSEVEISHECLKLIIEKYTRE
AGVRDLRRQIATIMRKVALKYLEDNPHQKGRTKKGKNEKSEDQKSEDQKS
ENQKSENKDFCVSITPNNLKEYLERMVFEIDPIDEENKIGIVNGLAWTPV
GGDVLKIEVLKIRGKGELKLTGSLGDVMKESAIIAFSVVKVLLDNETLKV
PKIPSETDAEGKKKKKVLKVYNAYDLHLHVPEGATPKDGPSAGIAMASVM
ASILCDRATRSEVAMTGELTLSGEVLPIGGLKEKLIAAFKAGIKTALIPV
KNYERDLDEIPAEVRENLNIVAVKNIAEVLEKTLL
> P04979|TOX3_BORPE Pertussis toxin subunit 3 - Bordetella pertussis.
MLINNKKLLHHILPILVLALLGMRTAQAVAPGIVIPPKALFTQQGGAYGR
CPNGTRALTVAELRGNAELQTYLRQITPGWSIYGLYDGTYLGQAYGGIIK
DAPPGAGFIYRETFCITTIYKTGQPAADHYYSKVTATRLLASTNSRLCAV
FVRDGQSVIGACASPYEGRYRDMYDALRRLLYMIYMSGLAVRVHVSKEEQ
YYDYEDATFQTYALTGISLCNPAASIC
> P27369|CYSR_SYNP7 Regulatory protein cysR - Synechococcus sp. (strain PCC
7942) (Anacystis nidulans R2).
MVREPASTLLPPTSPATPAPHRLLIGRRGMVPTGANVIWKIQSGLVRSST
WGEEGDMISLGLWGPGDLIGRPLSCLDPYELECLTAVEVVAVSDPALESH
ESLVRSLRYTERLLSITRLRRAEAKLASLLGWIGERFGQPGATGWEIDLR
RIPLTHQVIAELSGSTRVTTTRLLGE FRQAGRIHRRDRALIVRYPETLYP
PARLSA
> BTUR_SALTY P31570 COB(I)ALAMIN ADENOSYLTRANSFERASE (EC 2.5.
MSDERYQQRQQKVKDRVDARVAQAQEERGIIIVFTGNGKGKTTAAFGTAA
RAVGHGKNVGVVQFIKGTWPNGERNLLEPHGVEFQVMATGFTWETQNREA
DTAACMAVWQHGKRMLADPLLDMVVLDELTYMVAYDYLPLEEVISALNAR
PGHQTVIITGRGCHRDILDLADTVSELRPVKHAFDAGVKAQMGIDY
> P05818|BMAE_ECOLI M -agglutinin - Escherichia coli.
MNLKKIAIASSVFAGITMALTCHAVTVTATHTVESDAEFTIDWVDAGPTT
TDAKDGEVWGHLDMTQTRGTPTFGKLRNPQGETSPGPLKAPFSFTGPDGH
TARAYLDSYGAPIHNYAGDNLANGVKVGSGSGNTPFVVGTASRLTARIFG

DQTLVPGVYRTTFELTTWTD
> P15279|DHM1_METOR Methanol dehydrogenase subunit 1 - Methylobacterium organophilum XX.
MSRFVTSVSALAMLALAPAALSSVAYANDKLVELSKSDDNWVMPGKNYDS
NNYSELKQVNKSNVKQLRPAWTFSTGLLNGHEGAPLVVDGKMYVHTSFPN
NTFALDLDDPGHILWQDKPKQNPAARAVACCDLVNRGLAYWPGDGKTPAL
ILKTQLDRHVVALNAETGETVWKVENSDIKVGSTLTIAPYVVKDKVIIGS
SGAELGVRGYLTAYDVKTGGQVWRAYATGPDKDLLLADDFNVKNAHYGQK
GLGTATWEGDAWKIGGGTNWGWYAYDPGTNLIYFGTGNPAPWNETMRPGD
NKWTMTIFGRDADTGEAKFGYQKTPHDEWDYAGVNVMMPSEQKDKDGKTR
KLLTHPDRNGIVYTLDRTDGALVSANKLDDTVNVFKTVDLKTGQPVRDPE
YGTRMDHLAKDVCPSAMGYHNQGHDSYDPKRELFFMGINHICMDWEPFML
PYRAGQFFVGATLNMYPGPKGDRQNYEGLGQIKAYNAITGSYKWEKMERF
AVWGGTLATAGDLVFYGTLDGYLKARDSDTGDLLWKFKIPSGAIGYPMTY
THKGTQYVAIYYGVGGWPGVGLVFDLADPTAGLGAVGAFKKLANYTQQGG
GVIVFSLDGKGPYDDPNVGEWKSASK
> P09373|PFLB_ECOLI Formate acetyltransferase 1 - Escherichia coli.
MSELNEKLATAWEGFTKGDWQNEVNVRDFIQKNYTPYEGDESFLAGATEA
TTTLWDKVMEGVKLENRTHAPVDFDTAVASTITSHDAGYINKQLEKIVGL
QTEAPLKRALIPFGGIKMIEGSCKAYNRELDPMIKKIFTEYRKTHNQGVF
DVYTPDILRCRKSGVLTGLPDAYGRGRIIGDYRRVALYGIDYLMKDKLAQ
FTSLQADLENGVNLEQTIRLREEIAEQHRALGQMKEMAAKYGYDISGPAT
NAQEAIQWTYFGYLAAVKSQNGAAMSFGRTSTFLDVYIERDLKAGKITEQ
EAQEMVDHLVMKLRMVRFLRTPEYDELFSGDPIWATESIGGMGLDGRTLV
TKNSFRFLNTLYTMGPSPEPNMTILWSEKLPLNFKKFAAKVSIDTSSLQY
ENDDLMRPDFNNDDYAIACCVSPMIVGKQMQFFGARANLAKTMLYAINGG
VDEKLKMQVGPKSEPIKGDVLNYDEVMERMDHFMDWLAKQYITALNIIHY
MHDKYSYEASLMALHDRDVIRTMACGIAGLSVAADSLSAIKYAKVKPIRD
EDGLAIDFEIEGEYPQFGNNDPRVDDLAVDLVERFMKKIQKLHTYRDAIP
TQSVLTITSNVVYGKKTGNTPDGRRAGAPFGPGANPMHGRDQKGAVASLT
SVAKLPFAYAKDGISYTFSIVPNALGKDDEVRKTNLAGLMDGYFHHEASI
EGGQHLNVNVMNREMLLDAMENPEKYPQLTIRVSGYAVRFNSLTKEQQQD
VITRTFTQSM
> P13810|E2AA_ECOLI Heat-labile enterotoxin IIA, A chain - Escherichia coli.
MIKHVLLFFVFISFSVSANDFFRADSRTPDEIRRAGGLLPRGQQEAYERG
TPININLYEHARGTVTGNTRYNDGYVSTTVTLRQAHLIGQNILGSYNEYY
IYVVAPAPNLFDVNGVLGRYSPYPSENEFAALGGIPLSQIIGWYRVSFGA
IEGGMQRNRDYRGDLFRGLTVAPNEDGYQLAGFPSNFPAWREMPWSTFAP
EQCVPNNKEFKGGVCISATNVLSKYDLMNFKKLLKRRLALTFFMSEDDFI
GVHGERDEL
> P0A9L3|FKBB_ECOLI FKBP-type 22 kDa peptidyl-prolyl cis-trans isomerase - Escherichia coli.
MTTPTFDTIEAQASYGIGLQVGQQLSESGLEGLLPEALVAGIADALEGKH
PAVPVDVVHRALREIHERADAVRRQRFQAMAAEGVKYLEENAKKEGVNST
ESGLQFRVINQGEGAIPARTDRVRVHYTGKLIDGTVFDSSVARGEPAEFP
VNGVIPGWIEALTLMPVGSKWELTIPQELAYGERGAGASIPPFSTLVFEV
ELLEIL
> P19250|HLY2_VIBPA Thermostable direct hemolysin 2 - Vibrio parahaemolyticus.
MKYRYFAKKSFLFISMLAAFKTFAFELPSVPFPAPGSDEILFVVRDTTFN
TNAPVNVEVSDFWTNRNVKRKPYKDVYGQSVFTTSGTKWLTSYMTVNIND
KDYTMAAVSGYKHGHSAVFVKSDQVQLQHSYDSVANFVGEDEDSIPSKMY
LDETPEYFVNVEAYESGSGNILVMCISNKESFFECKHQQ
> P0A432|PSBO_SYNEN Photosystem II manganese-stabilizing polypeptide - Synechococcus elongatus naegeli.
MKYRILMATLLAVCLGIFSLSAPAFAAKQTLTYDDIVGTGLANKCPTLDD
TARGAYPIDSSQTYRIARLCLQPTTFLVKEEPKNKRQEAEFVPTKLVTRE
TTSLDQIQGELKVNSDGSLTFVEEDGIDFQPVTVQMAGGERIPLLFTVKN
LVASTQPNVTSITTSTDFKGEFNVPSYRTANFLDPKGRGLASGYDSAIAL
PQAKEEELARANVKRFSLTKGQISLNVAKVDGRTGEIAGTFESEQLSDDD
MGAHEPHEVKIQGVFYASIEPA
> P45175|RNG_HAEIN Ribonuclease G - Haemophilus influenzae.
MDAVELLMNVTPNETRIALVETGMLREVHIERQAKRGIVGNIYKGRVTRV

LPGMQSAFVDIGLEKAAFLHAADIVSHTECVDENEQKQFKVKSISELVRE
GQDIVVQVVKEPLGTKGARLTTDITLPSR HLVFMPENSHVGVSQRIESEE
ERARLKALVEPFCDELGGFIIRTATEGASEEELRQDAEFLKRLWRKVLER
KSKYPTKSKIYGEPALPQRILRDFIGTNLEKIRIDSKLCFGEVKEFTDEF
MPELSDKLVLYSGNQPIFDVYGVENAIQTALDKRVNLKSGGYLIIEQTEA
MTTIDINTGAFVGHRNLEETIFNTNIEATKAIAHELQLRNLGGIIIIDFI
DMQTDEHRNRVLQSLCDALSKDRMKTNVNG FTQLGLVEMTRKRTRESLEH
VLCDECPTCHGRGRVKTVETVCYEIMREIIRVYHLFSSEQFVVYASPAVS
EYLINEESHGLLPEVEMFIGKRVKVKTEQFYNQEQFDVVVM
> P27458|PRLB_ACHLY Beta -lytic metalloendopeptidase - Achromobacter lyticus.
MKKISKAGLGLALVCALATIGGNAARRATAQRRGSGVFYDEMFDFDIDAH
LAKHAPHLHKHSEE ISHWAGYSGISRSVDRADGAAERAVTPSARRIVRSA
SWRAPTASARRPARSRWRCASRCTSAIPTRQGAGDAGPRQSAAGAVRAFR
RQRAGGRAARRRRVPAGLRPPVQRTAPGQGGFGPLRQGRPGRAAVSPNGL
LQFPFPRGASWHVGGAHTNTGSGNYPMSSLDMSRGGGWGSNQNGNWVSAS
AAGSFKRHSSCFAEIVHTGGWSTTYYHLMNIQYNTGANVSMNTAIANPAN
TQAQALCNGGQSTGP HEHWSLKQNGSFYHLNGTYLSGYRITATGSSYDTN
CSRFYLTKNGQNYCYGYYVNPGPN
> Q47945|CYCA_GLUOX Alcohol dehydrogenase cytochrome c subunit -
Gluconobacter oxydans (Gluconobacter suboxydans).
MLNALTRDRLVSEMKQGWKLAAAIGLMAVSFGAAHAQDADEALIKRGEYV
ARLSDCIACHTALHGQPYAGGLEIKSPIG TIYSTNITPDPEHGIGNYTLE
DFTKALRKGIRKDGATVYPAMPYPEFARLSDDDIRAMYAFFMHGVKPVAL
QNKAPDISWPLSMRWPLGMWRAMFVPSMTPGVDKSISDPEVARGEYLVNG
PGHCGECHTPRGFGMQVKAYGTAGGNAYLAGGAPIDNWIAPSLRSNSDTG
LGRWSEDDIVTFLKSGRIDHSAVFGGMADVVAYSTQHWSDDDLRATAKYL
KSMPAVPEGKNLGQDDGQTTALLNKGGQGN AGAEVYLHNCAICHMNDGTG
VNRMFPPLAGNPVVITDDPTSLANVVAFGGILPPTNSAPSAVAMPGFKNH
LSDQEMADVVNFMRKGWGNNAPGTVSASDIQKLRTTGAPVSTAGWNVSSK
GWMAYMPQPYGEDWTFSPQTHTGVDDAQ
> P0AEG6|DSBC_ECOLI Thiol:disulfide interchange protein dsbC - Escherichia
coli.
MKKGFMLFTLLAAFSGFAQADDA AIQQTLAKMGIKSSDIQPAPVAGMKTV
LTNSGVLYITDDGKHIIQGPMYDVSGTAPVNVTNKMLLKQLNALEKEMIV
YKAPQEKHVITVFTDITCGYCHKLHEQMADYNALGITVRYLAFPRQGLDS
DAEKEMKAIWCAKDKNKAFDDVMAGKSVAPASCDVDIADHYALGVQLGVS
GTPAVVLSNGTLVPGYQPPKEMKEFLDEHQKMTSGK
> P50286|ASPG_WOLSU L -asparaginase - Wolinella succinogenes.
MAKPQVTILATGGTIAGSGESSVKSSYSAGAVTVDKLLAAVPAINDLATI
KGEQISSIGSQEMTGKVWLKLAKRVNELLAQKETEAVIITHGTDTMEETA
FFLNLTVKSQKPVVLVGAMRSGSSMSADGPMNLYNAVNVAINKASTNKGV
VIVMNDEIHAAREATKLNTTAVNAFASPNTGKIGTVYYGKVEYFTQSVRP
HTLASEFDISKIEELPRVDILYAHPDDTD VLVNAALQAGAKGIIHAGMGN
GNPFPLTQNALEKAAKSGVVVARSSRVGSGSTTQEAEVDDKKLGFVATES
LNPQKARVLLMLALTKTSDREAIQKIFSTY
> P39176|ERFK_ECOLI Protein erfK/srfK - Escherichia coli.
MRRVNILCSFALLFASHTSLAVTYPLPPEGSRLVGQSFTVTVPDHNTQPL
ETFAAQYGQGLSNMLEANPGADVFLPKSGSQLTIPQQLILPDT VRKGIVV
NVAEMRLYYYPPDSNTVEVFPIGIGQAGRETPRNWVTTVERKQEAPTWTP
TPNTRREYAKRGESLPAFVPAGPDNPMGLYAIYIGRLYAIHGTNANFGIG
LRVSQGCIRLRNDDIKYLFDNVPVGTRVQIIDQPVKYTTEPDGSNWLEVH
EPLSRNRAEYESDRKVPLPVTPSLRAFINGQEVDVNRANAALQRRSGMPV
QISSGSRQMF
> P24251|CRL_ECOLI Sigma factor -binding protein crl - Escherichia coli.
MTLPSGHPKSRLIKKFTALGPYIREGKCKDNRFFFDCLAVCVNVKPAPEV
REFWGWWMELEAQESRFTYSYQFGLFDKAGDWKSVPVKDTEVVERLEHTL
REFHEKLRELLTTLNLKLEPADDFRDEPVKLTA
> P13429|SFAG_ECOL5 S -fimbrial adhesin protein sfaG - Escherichia coli
O6:K15:H31 (strain 536 / UPEC).
MVKDIIKTVTFSCMLAGSMFVTCHVCAAGSVVNITGNVQDNTCDVDINSR
NFDVSLGSYDSRQFTAAGDTTPASVFHVGLTSCGSAVRAVKLTFTGTPDN
QEAGLIQINSINGARGVGIQLLDKDKHELKINVPTTIALMPGTQTIAFYA
RLKATYLPVKAGNVDAVVNFVLDYQ

> P14283|PERT_BORPE Pertactin - Bordetella pertussis.
MNMSLSRIVKAAPLRRTTLAMALGALGAAPAAHADWNNQSIVKTGERQHG
IHIQGSDPGGVRTASGTTIKVSGRQAQGILLENPAAELQFRNGSVTSSGQ
LSDDGIRRFLGTVTVKAGKLVADHATLANVGDTWDDDGIALYVAGEQAQA
SIADSTLQGAGGVQIERGANVTVQRSAIVDGGLHIGALQSLQPEDLPPSR
VVLRDTNVTAVPASGAPAAVSVLGASELTLDGGHITGGRAAGVAAMQGAV
VHLQRATIRRGDAPAGGAVPGGAVPGGAVPGGFGPGGFGPVLDGWYGVDV
SGSSVELAQSIVEAPELGAAIRVGRGARVTVSGGSLSAPHGNVIETGGAR
RFAPQAAPLSITLQAGAHAQGKALLYRVLPEPVKLTLTGGADAQGDIVAT
ELPSIPGTSIGPLDVALASQARWTGATRAVDSLSIDNATWVMTDNSNVGA
LRLASDGSVDFQQPAEAGRFKVLTVNTLAGSGLFRMNVFADLGLSDKLVV
MQDASGQHRLWVRNSGSEPASANTLLLVQTPLGSAATFTLANKDGKVDIG
TYRYRLAANGNGQWSLVGAKAPPAPKPAPQPGPQPPQPPQPQPEAPAPQP
PAGRELSAAANAAVNTGGVGLASTLWYAESNALSKRLGELRLNPDAGGAW
GRGFAQRQQLDNRAGRRFDQKVAGFELGADHAVAVAGGRWHLGGLAGYTR
GDRGFTGDGGGHTDSVHVGGYATYIADSGFYLDATLRASRLENDFKVAGS
DGYAVKGKYRTHGVGASLEAGRRFTHADGWFLEPQAELAVFRAGGGAYRA
ANGLRVRDEGGSSVLGRLGLEVGKRIELAGGRQVQPYIKASVLQEFDGAG
TVHTNGIAHRTELRGTRAELGLGMAAALGRGHSLYASYEYSKGPKLAMPW
TFHAGYRYSW
> CILB_HAEIN P44460 CITRATE LYASE BETA CHAIN (EC 4.1.3.6) (AC
MKLRRSMLFVPGSNAAMLSNSFIYKPDSIMFDLE DAVALKEKDSARLLVA
HALQHPLYKEIETVVRVNPLDSEFGLLDLNSVVRAGVDVVRMPKTESAQD
VLDMDHAITEIEKACGREAGSTKMLAAIESPLGITQANQIAFASKRLIGI
ALGAEDYVRNLKTERSPEGIELLFARCSILQAARAAGIQAFDTVYSNANN
EEGFLKEAALIKQLGFDGKSLINPRQIELLHNLFAPTQKDVEQAKRIIEA
AVEAERQGAGVVSLNGKMIDAPIIDRAKLVLERAK SGIREE
> Q00971|NPRV_VIBPR Neutral protease - Vibrio proteolyticus (Aeromonas proteolytica).
MNKTQRHINWLLAVSAATALPVTAAEMINVNDGSLLNQALKAQSQSVAPV
ETGFKQMKRVVLPNGKVKVRYQQTHHGLPVFNTSVVATESKSGSSEVFGV
MAQGIADDVSTLTPSVEMKQAISIAKSRFQQQEKMVAEPATENEKAELMV
RLDDNNQAQL VYLVDFFVAEDHPARPFFFIDAQTGEVLQTWDGLNHAQAD
GTGPGGNTKTGRYEYGSDFPPFVIDKVGTKCSMNNSAVRTVDLNGSTSGN
TTYSYTCNDSTNYNDYKAINGAYSPLNDAHYFGKVVFDMYKDWMNTTPLT
FQLTMRVHYGNNYENAFWNGSSMTFGDGYSTFYPLVDINVSAHEVSHGFT
EQNSGLVYENMSGGMNEAFSDIAGEAAEFYMKGSVDWVVGADIFKSSGGL
RYFDQPSRDGR SIDHASDYYNGLNVHYSSGVFNRAFYLLANKAGWDVRKG
FEVFTLANQLYWTANSTFDEGGCGVVKAASDMGYSVADVEDAFNTVGVNA
SCGATPPPSGDVLEIGKPLANLSGNRNDMTYYTFTPSSSSSVVIKITGGT
GDADLYVKAGSKPTTTSYDCRPYKYGNEEQCSISAQAGTTYHVMLRGYSN
YAGVTLRAD
> P02970|FMKB_ECOLI K88 fimbrial protein AB - Escherichia coli.
MKKTLIALAIAASAASGMAHAWMTGDFNGSVDIGGSITADDYRQKWEWKV
GTGLNGFGNVLNDLTNGGTKLTITVTGNKPILLGRTKEAFATPVSGGVDG
IPQIAFTDYEGASVKLRNTDGETNKGLAYFVLPMKNAEGTKVGSVKVNAS
YAGVFGKGGVTSADGELFSLFADGLRAIFYGGLTTTVSGAALTSGSAAAA
RTELFGSLSRNDILGQIQRVNANITSLVDVAGSYREDMEYT DGTVVSAAY
ALGIANGQTIEATFNQAVTTSTQWSAPLNVAITYY
> P33590|NIKA_ECOLI Nickel-binding periplasmic protein - Escherichia coli.
MLSTLRRTLFALLACASFIVHAAAPDEITTAWPVNVGPLNPHLYTPNQMF
AQSMVYEPLVKYQADGSVIPWLAKSWTHSEDGKTWTFTLRDDVKFSNGEP
FDAEAAAENFRAVLDNRQRHAWLELANQIVDVK ALSKTELQITLKSAYYP
FLQELALPRPFRFIAPSQFKNHETMNGIKAPIGTGPWILQESKLNQYDVF
VRNENYWGEKPAIKKITFNVIPDPTTRAVAFETGDIDLLYGNEGLLPLDT
FARFSQNPAYHTQLSQPIETVMLALNTAKAPTNELAVREALNYAVNKKSL
IDNALYGTQQVADTLFAPSVPYANLGLKPSQYDPQKAKALLEKAGWTLPA
GKDIREKNGQPLRIELSFIGTDALSKSMAEIIQA DMRQIGADVSLIGEEE
SSIYARQRDGRFGMIFHRTWGAPYDPHAFLSSMRVPSHADFQAQQGLADK
PLIDKEIGEVLATHDETQRQALYRDILTRLHDEAVYLPISYISMMVVSKP
ELGNIPYAPIATEIPFEQIKPVKP
> P00154|CYCP_CHRVI Cytochrome c' - Chromatium vinosum (Allochromatium vinosum).

MKHVLASTAAGLMALGLASSAIAAGLSPEEQ IETRQAGYEFMGWNMGKIK
ANLEGEYNAAQVEAAANVIAAIANSGMGALYGPGTDKNVGDVKTRVKPEF
FQNMEDVGKIAREFVGAANTLAEVAATGEAEAVKTAFGDVGAACKSCHEK
YRAK
> P28581|PHOC_MORMO Major phosphate -irrepressible acid phosphatase - Morganella morganii (Proteus morganii).
MKKNIIAGCLFSLFSLSALA AIPAGNDATTKPDLYYLKNEQAIDSLKLLP
PPPEVGSIQFLNDQAMYEKGRMLRNTERGKQAQADADLAAGGVATAFSGA
FGYPITEKDSPELYKLLTNMIEDAGDLATRSAKEHYMRIRPFAFYGTETC
NTKDQKKLSTNGSYPSGHTSIGWATALVLAEVNPANQDAILERGYQLGQS
RVICGYHWQSDVDAARIVGSAAVATLHSDPAFQAQLAKAKQEFAQKSQK
> Q99289|HLT_VIBPA The rmolabile hemolysin - Vibrio parahaemolyticus.
MMKKTITLLTALLPLASAVAEEPTLSPEMVSASEVISTQENQTYTYVRCW
YRTSYSKDDPATDWEWAKNEDGSYFTIDGYWWSSVSFKNMFYTNTSQNVI
RQRCEATLDLANENADITFFAADNRFSYNHTIWSNDAAMQPDQINKVVAL
GDSLSDTGNIFNASQWRFPNPNSWFLGHFSNGFVWTEYIAKAKNLPLYNW
AVGGAAGENQYIALTGVGEQVSSYLTYAKLAKNYKPANTLFTLEFGLNDF
MNYNRGVPEVKADYAEALIRLTDAGAKNFMLMTLPDATKAPQFKYSTQEE
IDKIRAKVLEMNEFIKAQAMYYKAQGYNITLFDTHALFETLTSAPEEHGF
VNASDPCLDINRSSSVDYMYTHALRSECAASGAEKFVFWDVTHPTTATHR
YVAEKMLESSNNLAEYRF
> P0A7A5|PIMT_ECOLI Protein -L-isoaspartate O-methyltransferase - Escherichia coli.
MVSRRVQALLDQLRAQGIQDEQVLNALAAVPREKFVDEAFEQKAWDNIAL
PIGQGQTISQPYMVARMTELLELTPQSRVLEIGTGSGYQTAILAHLVQHV
CSVERIKGLQWQARRRLKNLDLHNVSTRHGDGWQGWQARAPFDAIIVTAA
PPEIPTALMTQLDEGGILVLPVGEEHQYLKRVRRRGGEFIIDTVEAVRFV
PLVKGELA
> Q55187|SYFA_SYNY3 Phenylalanyl -tRNA synthetase alpha chain - Synechocystis sp. (strain PCC 6803).
MTISLEADLKSLQQSAQAAISGCDDLDGLDKLRVQYLGKKGELSLILKGM
GKLSAEERPKFGAIANEVKEALQHDLESRKANLQNAAIEAQLAAETLDVT
MAGSYRPQGRRHPLNSTVDRVLDIFVGLGYTVATGPQVETDYYNFEALNI
PADHPARDMQDTFFLKDGRLLRTHTSPVQIRYMEKHDPPIRIVAPGRVYR
RDTVDATHSAVFHQVELLAIDKGLAFTHLKGTIQAFIKQMFGEALPIRFR
ASYFPFTEPSAEVDVQWQGKWLEVMGCGMVDPNVMEAVGYDPEVYTGFAA
GFGVERFAMVLHQIDDIRRLYNSDLRFLRQF
> P16454|AIL_YEREN Attachment invasion locus protein - Yersinia enterocolitica.
MKKTLLASSLIACLSIASVNVYAASESSISIGYAQSHVKENGYTLDNDPK
GFNLKYRYELDDNWGVIGSFAYTHQGYDFFYGSNKFGHGDVDYYSVTMGP
SFRINEYVSLYGLLGAAHGKVKASVFDESISASKTSMAYGAGVQFNPLPN
FVIDASYEYSKLDSIKVGTWMLGAGYRF
> P44398|XYLA_HAEIN Xylose isomerase - Haemophilus influenzae.
MTTYFDKIEKISFEGEKSTNPFAFKHYDANQVILGKTMAEHLRLAVCYWH
TFCWNGNDMFGLGSLERSWQKNSNLLAGAEQKADIAFEFLNKLGVPYYCF
HDVDIAPEGNSVREYVQNFHHIVDILERKQVETGVKLLWGTANCFTNPRY
MSGAATNPNPEVFAWAATQVFNAMNATQRLGGENYVLWGGREGYETLLNT
DLKREREQIGRFMQMVVEHKHKIGFKGTLLIEPKPQEPTKHQYDYDVATV
YGFLKQFGLEKEIKVNIEANHATLAGHTFQHEIATACALDIFGSIDANRG
DPQLGWDTDQFPNSVEENTLVMYEILKHGGFTTGGFNFDAKIRRQSIDPY
DLFYAHIGAIDVLALSLKRAAKMLQEETLQKIVNERYAGWNSELGQHILQ
GKTSLETLAQLVQQKDLAPKPVSGQQEYLENLVNQVIYS
> Q05918|IPHP_NOSCO Tyrosine -protein phosphatase - Nostoc commune.
MKTHHANLALALMLGLSSSATAVAADAPQAVATKAAAPNVKPVAADAHGV
IPDGAPGMCARSPACRATAIPADAFVRTADLGRLTDADRDALAALGVKLD
IDLRTADEEAQSPDLLARDDRFDYQRISLMGTEKMDLQKMMTSFPDSLGE
AYVQWLGHSQPQFKQVFQRIAAQQDGAVLFHCTAGKDRTGIIAGLLLDLA
GVPKAEIVHNYAISAHYLEGQPKDSDERADHGAGQAEPGDRPQDGGHGRY
RAGQHGAVLAALHSQYGGAEGYLKSIGVSEQEIQQLKVRLGQAG
> P42195|PECS_DICD3 HTH -type transcriptional regulator pecS - Dickeya dadantii (strain 3937) (Erwinia chrysanthemi (strain 3937)).
MARYLEVSDIVQQWRNERPDLDVEPMLVIGTLSRVSLLIDRALDKVFSKY

KLSAREFDILATLRRRGAPYAYSPSQIVNA LMINNSTLTSRLDRLEQAGW
LRRMPIEGDRRSVNIQLTDEGFALINRVVEEHVENERDILSPFSEEEKTH
LRALLGRVEKHLVNNR
> P24474|NIRS_PSEAE Nitrite reductase - Pseudomonas aeruginosa.
MPFGKPLVGTLLASLTLLGLATAHAKDDMKAAEQYQGAASAVDPAHVVRT
NGAPDMSESEFNEAKQIYFQRCAGCHGVLRKGATGKPLTPDITQQRGQQY
LEALITYGTPLGMPNWGSSGELSKEQITLMAKYIQHTPPQPPEWGMPEMR
ESWKVLVKPEDRPKKQLNDLDLPNLFSVTLRDAGQIALVDGDSKKIVKVI
DTGYAVHISRMSASGRYLLVIGRDARIDMIDLWAKEPTKVAEIKIGIEAR
SVESSKFKGYEDRYTIAGAYWPPQFAIMDGETLEPKQIVSTRGMTVDTQT
YHPEPRVAAIIASHEHPEFIVNVKETGKVLLVNYKDIDNLTVTSIGAAPF
LHDGGWDSSHRYFMTAANNSNKVAVIDSKDRRLSALVDVGKTPHPGRGAN
FVHPKYGPVWSTSHLGDGSISLIGTDPKNHPQYAWKKVAELQGQGGGSLF
IKTHPKSSHLYVDTTFNPDARISQSVAVFDLKNLDAKYQVLPIAEWADLG
EGAKRVVQPEYNKRGDEVWFSVWNGKNDSSALVVVDDKTLKLKAVVKDPR
LITPTGKFNVYNTQHDVY
> P0A433|OPD_FLAS2 Parathion hydrol ase - Flavobacterium sp. (strain ATCC 27551).
MQTRRVVLKSAAAAGTLLGGLAGCASVAGSIGTGDRINTVRGPITISEAG
FTLTHEHICGSSAGFLRAWPEFFGSRKALAEKAVRGLRRARAAGVRTIVD
VSTFDIGRDVSLLAEVSRAADVHIVAATGLWFDPPLSMRLRSVEELTQFF
LREIQYGIEDTGIRAGIIKVATTGKATPFQELVLKAAARASLATGVPVTT
HTAASQRDGEQQAAIFESEGLSPSRVCIGHSDDTDDLSYLTALAARGYLI
GLDHIPHSAIGLEDNASASALLGIRSWQTRALLIKALIDQGYMKQILVSN
DWLFGFSSYVTNIMDVMDRVNPDGMAFIPLRVIPFLREKGVPQETLAGIT
VTNPARFLSPTLRAS
> P0A282|PTH_SALTI Peptidyl -tRNA hydrolase - Salmonella typhi.
MAIKLIVGLANPGAEYAATRHNAGAWYVDL LAERLRAPLREEPKFFGYTS
RITLEGEDVRLLVPTTFMNLSGKAVGAMASFYRIQPDEILVAHDELDLPP
GVAKFKLGGGHGGHNGLKDIISKLGNNPNFHRLRVGIGHPGDKNKVVGFV
LGKPPVSEQKLIDEAIDEAARCTELWFKEGLAKATSRLHTFKAQ
> P14005|PELP_PECCC Periplasmic pectate lyase - Pectobacterium carotovorum subsp. carotovorum (Erwinia carotovora subsp. carotovora).
MKRFALSLLAGLVALQASAATPDRLTIVNQYVDNVLTKAGDHYHGQSPTP
LLADGVDPRTGKQMEWIFPDGRHAVLSNFSAQQNLMRVLVGLSNLSGNPS
YKQRAEAIVKYHFQHYQDESGLLIWGGHRFVDLKTLQPEGPSEKEMVHEL
KNAYPYYDLMFSVDKDATARFIRGFWNAHVYDWKIMETSRHGKYGQKIGA
LWQSPFEQQPPFFATKGLSFLNAGNDLIYSASLLYKYNKEDGALVWAKRL
AQQYVLPRDKATGLGVYQFTQALKRDETTDDADTHSKYGDRAQRQFGPEF
GPTALEGNMMLKGRTSTIYSENALMQLQLGKDLGAEGKELLTWTTDGLKA
FAKYAYNESDNTFRPMLANGKDLSNYVLPRDGYYGKKGTVIKPYPADNSF
LLSYARAYTVLPDAELWRVARGIARAQGLGELGSAPGKDVKVDLATKNND
PYALFALLDLYQASKVKDYLSLAEKVGDNIISTRYQNGFFMAEPNRQYAD
VDTIEPYALLALEAAVRNQPQSVAPFLNGAGFTEGGYRMEDGSTRVSTRD
NEIFLLNVGETLKPNNKK
> P0A3R5|TOX4_BORPE Pertussis toxin subunit 4 - Bordetella pertussis.
MLRRFPTRTTAPGQGGARRSRVRALAWLLASGAMTHLSPALADVPYVLVK
TNMVVTSVAMKPYEVTPTRML VCGIAAKLGAAASSPDAHVPFCFGKDLKR
PGSSPMEVMLRAVFMQQRPLRMFLGPKQLTFEGKPALELIRMVECSGKQD
CP
> P25253|OMPX_ENTCL Outer membrane protein X - Enterobacter cloacae.
MKKIACLSALAAVLAVSAGTAVAATSTVTGGYAQSDMQGVMNKTNGFNLK
YRYEQDNNPLGVIGSFTYTEKDRTENGSYNKGQYYGITAGPAYRLNDWAS
IYGVVGVGYGKFQQTENQGLNRTASNSDYGFSYGAGMQFNPIENVALDFS
YEQSRIRNVDVGTWIAGVGYRF
> P23827|ECOT_ECOLI Ecotin - Escherichia coli.
MKTILPAVLFAAFATTSAWAAESVQPLEKIAPYPQAEKGMKRQVIQLTPQ
EDESTLKVELLIGQTLEVDCNLHRLGGKLENKTLEGWGYDYYVFDKVSSP
VSTMMACPDGKKEKKFVTAYLGDAGMLRYNSKLP IVVYTPDNVDVKYRVW
KAEEKIDNAVVR
> Q47537|TAUA_ECOLI Taurine -binding periplasmic protein - Escherichia coli.
MAISSRNTLLAALAFIAFQAQAVNVTVAYQTSAEPAKVAQADNTFAKESG
ATVDWRKFDSGASIVRALASGDVQIGNLGSSPLAVAASQQVPIEVFLLAS

KLGNSEALVVKKTISKPEDLIGKRIAVPFISTTHYSLLAALKHWGIKP GQ
VEIVNLQPPAIIAAWQRGDIDGAYVWAPAVNALEKDGKVLTDSEQVGQWG
APTLDVWVVRKDFAEKHPEVVKAFAKSAIDAQQPYIANPDVWLKQPENIS
KLARLSGVPEGDVPGLVKGNTYLTPQQQTAELTGPVNKAIIDTAQFLKEQ
GKVPAVANDYSQYVTSRFVQ
> P13857|RIML_ECOLI Ribosomal -protein-serine acetyltransferase -
Escherichia coli.
MTETIKVSESLELHAVAENHVKPLYQLICKNKTWLQQSLNWPQFVQSEED
TRKTVQGNVMLHQRGYAKMFMIFKEDELIGVISFNRIEPLNKTAEIGYWL
DESHQGQGIISQALQALIHHYAQSGELRRFVIKCRVDNPQSNQVALRNGF
ILEGCLKQAEFLNDAYDDVNLYARIIDSQ
> P09394|GLPQ_ECOLI Glycerophosphoryl diester phosphodiesterase -
Escherichia coli.
MKLTLKNLSMAIMMSTIVMGSSAMAADSNEKIVIAHRGASGYLPEHTLPA
KAMAYAQGADYLEQDLVMTKDDNLVVLHDHYLDRVTDVADRFPDRARKDG
RYYAIDFTLDEIKSLKFTEGFDIENGKKVQTYPGRFPMGKSDFRVHTFEE
EIEFVQGLNHSTGKNIGIYPEIKAPWFHHQEGKDIAAKTLEVLKKYGYTG
KDDKVYLQCFDADELKRIKNELEPKMGMELNLVQLIA YTDWNETQQKQPD
GSWVNYNYDWMFKPGAMKQVAEYADGIGPDYHMLIEETSQPGNIKLTGMV
QDAQQNKLVVHPYTVRSDKLPEYTPDVNQLYDALYNKAGVNGLFTDFPDK
AVKFLNKE
> P16630|GUNS_PECCC Endoglucanase S - Pectobacterium carotovorum subsp.
carotovorum (Erwinia carotovora subsp. carotovora).
MQTVNTQPHRIFRVLLPAVFSSLLLSSLTVSAASSSNDADKLYFGNNKYY
LFNNVWGKDEIKGWQQTIFYNSPISMGWNWHWPSSTHSVKAYPSLVSGWH
WTAGYTENSGLPIQLSSNKSITSNVTYSIKATGTYNAAYDIWFHTTDKAN
WDSSPTDELMIWLNDTNAGPAGDYIETVFLGDSSWNVFKGWINADNGGGW
NVFSFVHTSGTNSASLNIRHFTDYLVQTKQWMSDEKYISSVEFGTEIFGG
DGQIDITEWRVDVK
> Q02286|PHEA_ENTAG P-protein [Includes: Chorismate mutase - Enterobacter
agglomerans (Erwinia herbicola) (Pantoea agglomerans).
MNPDNPLLALRDKISAVDKKLLTLLAERRLLAVEVAQAKLATHRPIRDVE
RERALLENLIVLGKAHNLDAHYITRLFQLVIEDSVLTQQALLQKNLNHPH
AHAARIAFLGPKGSYSHL AARNYASRHFDSMVECGCLKFHDIIKQVENGV
ADYAVMPIENTSSGSINDVYDLLQQTSLSIVGELTLPIDHCVLVNGPTDL
QQIETVYSHPQPFQQCSQFINRFPHWKIEYTESTAAAMEKVAALNSPKVA
ALGSEAGGELYQLQVLERNLANQQQNHTRFIVLARKAIEVSDQVPAKTTL
IMATGQQAGALVDALLVLRQHNLIMSKLESRPINGNPWEEMFYIDVQGNL
QSERMQQALQELQTMTRSL KVLGCYPSENVVPAEPGR
> CYSE_SYNP7 Q56002 SERINE ACETYLTRANSFERASE (EC 2.3.1.30) (S
MFKTLAADFRIIFERDPAARNGLEVLLCYPGFQALVCHRVAHWLYQQRLP
VIPRLLSHLSRLLTGVEIHPGARLGQGIFIDHGMGVVIGETAIVGDYCLI
YQGVTLGGTGKQSGKRHPTLANNVVVGAGAKVLGNIQIGENVRIGAGSVV
LRDVPSDCTVVGIPGRVIYRSG VRVDPLDHSQMPDSEARVIRMLLDRIEA
LEDQLEGLNLPPAEEAIDVTLAPVGDRCQLRDRTIEEFLDGAGI
> P56069|METB_HELPY Cystathionine gamma -synthase - Helicobacter pylori
(Campylobacter pylori).
MRMQTKLIHGGISEDATTGAVSVPIYQTSTYRQDAIGRHKGYEYSRSGNP
TRFALEELIADLEGGVKGFAFASGLAGIHAVFSLLQ SGDHVLLGDDVYGG
TFRLFNQVLVKNGLSCTIIDTSDISQIKKAIKPNTKALYLETPSNPLLKI
TDLAQCASVAKDHGLLTIVDNTFATPYYQNPLLLGADIVAHSGTKYLGGH
SDVVAGLVTTNNEALAQEIAFFQNAIGGVLGPQDSWLLQRGIKTLGLRME
AHQKNALCVAEFLEKHPKVERVYYPGLPTHPNYELAKKQMRGFSGMLSFT
LKNDSEAVAFVESLKLFILGESLGGVESLVGIPAFMT HACIPKTQREAAG
IRDGLVRLSVGIEHEQDLLEDLEQAFAKIG

Positive Training Data

> gi|51591594|emb|CAF25398.1| yopB; putative Yop targeting protein
[Yersinia pseudotuberculosis IP 32953]
MSALITHDRSTPVTGSLLPYVETPAPAPLQTQQVAGELKDKNGGVSSQGV
QLPAPLAVVASQVTEGQQQEVTKLLESV TRGAAGSQLISNYVSVLTKFTL
ASPDTFEIELGKLVSNLEEVRKDIKIADIQRLHEQNMKKIEENQEKIKET
EENAKQVKKSGIASKIFGWLSAIASVIVGAIMVASGVGAVAGAMMVASGV
IGMANMAVKQAAEDGLISQEAMKILGPILTAIEVALTVVSTVMTFGGSAL
KCLANIGAKLGANTASLAAKGAEFSAKVAQISTGISNTVGSAVTKLGGSF
AGLTMSHAIRTGSQATQVAVGVGSGITQT INNKKQADLQHNNADLALNKA
DMAALQSIIDRLKEELSHLSESHQQVMELIFQMINAKGDMLHNLAGRPHT
V
> Q8XYE3_RALSO
MRIGKSSGWLNESVSLEYEHVSPPTRPRDTRRRPRAAGDGGLAHLHRRLA
VGYAEDTPRTEARSPAPRRPLPVAPASAPPAPSLVPEPPMPVSLPAVSSP
RFSAGSSAAITDPFPSLPPTPVLYAMARELEALSDATWQPAVPLPAEPPT
DARRGNTVFDEAS ASSPVIASACPQAFASPPRAPRSARARRARTGGDAWP
APTFLSRPSSSRIGRDVFGKLVALGYSREQIRKLKQESLSEIAKYHTTLT
GQGFTHADICRISRRRQSLRVVARNYPELAAALPELTRAHIVDIARQRSG
DLALQALLPVATALTAAPLRLSASQIATVAQYGERPAIQALYRLRRKLTR
APLHLTPQQVVAIASNTGGKRALEAVCVQLPVLRAAPYRLSTEQVVAIAS
NKGGKQALEAVKAH LLDLLGAPYVLDTEQVVAIASHNGGKQALEAVKADL
LDLRGAPYALSTEQVVAIASHNGGKQALEAVKADLLELRGAPYALSTEQV
VAIASHNGGKQALEAVKAHLLDLRGVPYALSTEQVVAIASHNGGKQALEA
VKAQLLDLRGAPYALSTAQVVAIASNGGGKQALEGIGEQLLKLRTAPYGL
STEQVVAIASHDGGKQALEAVGAQLVALRAAPYALSTEQVVAIASNKGGK
QALEAVKAQLLELRG APYALSTAQVVAIASHDGGNQALEAVGTQLVALRA
APYALSTEQVVAIASHDGGKQALEAVGAQLVALRAAPYALNTEQVVAIAS
SHGGKQALEAVRALFPDLRAAPYALSTAQLVAIASNPGGKQALEAVRALF
RELRAAPYALSTEQVVAIASNHGGKQALEAVRALFRGLRAAPYGLSTAQV
VAIASSNGGKQALEAVWALLPVLRATPYDLNTAQIVAIASHDGGKPALEA
VWAKLPVLRGAPYALS TAQVVAIACISGQQALEAIEAHMPTLRQASHSLS
PERVAAIACIGGRSAVEAVRQGLPVKAIRRIRREKAPVAGPPPASLGPTP
QELVAVLHFFRAHQQPRQAFVDALAAFQATRPALLRLLSSVGVTEIEALG
GTIPDATERWQRLLGRLGFRPATGAAAPSPDSLQGFAQSLERTLGSPGMA
GQSACSPHRKRPAETAIAPRSIRRSPNNAGQPSEPWPDQLAWLQRRKRTA
RSHIRADSAASVPANLH LGTRAQFTPDRLRAEPGPIMQAHTSPASVSFGS
HVAFEPGLPDPGTPTSADLASFEAEPFGVGPLDFHLDWLLQILET
> gi|12025046|gb|AAG45729.1|AF229440|2 y4yA [Sinorhizobium fredii]
MTLHCQKIGHGLPPILRSATADLLTIHGPLLFDWAARHGSPLNLVWPDAL
QENLAALKGVLTDCRVEHAIYYGAKVNKSPGLMQAALSAGAGLDVSSLYE
LRDARLLGAEGVRLVATGPAKTGAFHQELIDCNALISVDPPGRLEDLIHG
LPADVGPQPILLRLRPRNQSKSRFGMPADAVVHCLARLAGESRLRFDGLN
FHLSGYLWETRVAALSEAADLIAEARRMGFSPRMIDIGGGLPIQYVDRAR
YEAHLAAQAPEDYRNGKIPDSFYPYGSNLSAADWLHRLLEAEMSQGRSVA
GYLAREGLTLAMEPGRALADQAAITVFLISRVKALGPDSHVIFVEGSSFS
ACETWFAS EFLVDPILVPATRPAAQSPPIRAYLAGHSCLDEDVLSNRWLS
FPIAPHAGDLLVYANTGGYQMDLLENEFHRHAMPARLCVVKDAEGHPILV
PDTIGEV
> Q8Y0Z8_RALSO
MGCFNVTGASGKASDYVAERHANQPSTEQATQRPAARSAGQGVLQGLQRQ
NSTSRPSDDESAIQHAVAQARAGERFRPGPLPEQKELTGPKIGRSD
> gi|12512890|gb|AAG54470.1|AE005192|12 m ethionine aminopeptidase
[Escherichia coli O157:H7 EDL933]
MAISIKTPEDIEKMRVAGRLAAEVLEMIEPYVKPGVSTGELDRICNDYIV
NEQHAVSACLGYHGYPKSVCISINEVVCHGIPDDAKLLKDGDIVNIDVTV
IKDGFHGDTSKMFIVGKPTIMGERLCRITQESLYLALRMVKPGINLREIG
AAIQKFVEAEGFSVVREYCGHGIGRGFHEEPQVLHYDSRETNVV LKPGMT
FTIEPMVNAGKKEIRTMKDGWTVKTKDRSLSAQYEHTIVVTDNGCEILTL
RKDDTIPAIISHDE

> gi|28868585|ref|NP|791204.1| avirulence protein AvrE(Pto) [Pseudomonas syringae pv. tomato str. DC3000]
MQSPSIHRNTGSIIQPTVTPDARAATDLQERAEQPRQRSSHSLSSVGKRA
LKSVGKLFQKSKAPQQKAATPPTAKN VKTPPPASNVATPRNKARESGFSN
SSPQNTHSAPKSILRNHPNQASSSGAQTHEIHPEAAPRKNLRVRFDLPQD
RLERSPSYLDSDNPMTDEEAVANATRQFRSPDSHLQGSDGTRISMLATDP
DQPSSSGSKIGDSDGPIPPREPMLWRSNGGRFELKDEKLVRNSEPQGSIQ
LDAKGKPDFSTFNTPGLAPLLDSILATPKQTYLAHQSKDGVHGHQLLQAN
GHLLHLAQDDSSLAVIRSSNEALLIEG KKPPAVKMEREDGNIHIDTASGR
KTQELPGKAHIAHITNVLLSHDGERMRVHEDRLYQFDPISTRWKIPEGLE
DTAFNSLSTGGNGSVYAKSDDAVVDLSSPFMPHVEVEDLQSFSVAPDNRA
ALLSGKTTQAILLTDMSPVIGGLTPKKTKGLELDGGKAQAAAVGLSGDKL
FIADTQGRLYSADRSAFEGDDPKLKLMPEQANFQLEGVPLGGHNRVTGFI
NGDDGGVHALIKNRQGETHSHALDEQSS KLQSGWNLTNALVLNNNRGLTM
PPPPTAADRLNLDRAGLVGLSEGRIQRWDATPECWKDAGIKDIDRLQRGA
DSNAYVLKGGKLHALKIAAEHPNMAFDRNTALAQTARSTKVEMGKEIEGL
DDRVIKAFAMVSNKRFVALDDQNKLTAHSKDHKPVTLDIPGLEGDIKSLS
LDEKHNLHALTSTGGLYCLPKEAWQSTKLGDQLRARWTPVALPGGQPVKA
LFTNDDNVLSAQIEDAEGKGLMQLKAGQW QRFEQRPVEENGLNDVHSRIT
GSNKTWRIPKTGLTLRMDVNTFGRSGVEKSKKASTSEFIRANIYKNTAET
PRWMKNVGDHIQHRYQGRLGLKEVYETESMLFKQLELIHESGGRPPARGQ
DLKARITALEAKLGPQGATLVKELETLRDELENHSYTALMSIGQSYGKAK
NLKQQDGILNQHGELAKPSVRMQFGKKLADLGTKLNFKSSGHDLVKELQD
ALTQVAPSAENPTKKLLGTLKHQGLKLSHQ KADIPLGQRRDASEDHGLSK
ARLALDLVTLKSLGALLDQVEQLPPQSDIEPLQKKLATLRDVTYGENPVK
VVTDMGFTDNKALESGYESVKTFLKSFKKADHAVSVNMRAATGSKDQAEL
AGKFKSMLKQLEHGDDEVGLQRSYGVNLTTPFIILADKATGLWPTAGATG
NRNYILNAERCEGGVTLYLISEGAGNVSGGFGAGKDYWPGFFDANNPARS
VDVGNNRTLTPNFRLGVDVTATVAASQRAGV VFNVPDEDIDAFVDDLFEG
QLNPLQVLKKAVDHESYEARRFNFDLTAGGTADIRAGINLTEDRDPNADP
NSDSFSAVVRGGFAANITVNLMTYTDYSLTQKNDKTELKEGGKNRPRFLN
NVTAGGQLRAQIGGSHTAPTGTPASAPGPTPASQTAANNLGGALNFSVEN
RTVKRIKFRYNVAKPITTEGLSKLSKGLGEAFLDNTTKAKLAELADPLNA
RYTGKKPDEVIQAQLDGLEELFADIPPPKDND KQYKALRDLKRAAVEHRA
SANKHSVMDNARFETSKTNLSGLSSESILTKIMSSVRDASAPGNATRVAE
FMRQDPKLRAMLKEMEGSIGTLARVRLEPKDSLVDKIDEGSLNGTMTQSD
LSSMLEDRNEMRIKRLVVFHTATQAENFTSPTPLVSYNSGANVSVTKTLG
RINFVYGADQDKPIGYTFDGELSRPSASLKEAAGDLKKEGFELKS
> Q7AGE0_ECO57
MRPTSLNLVLHQSSRSSSMSDTD IESLVKASSVQWIKNNPQLRFQGTDHN
IYQQIEAALDKIGSTETGRVLLNAIESISRLKSETVVIHLNSSRLGVMAH
RDIDAENHRGTGSDFHCNLNAVEYPCGEGISVVDFHATIVFHELLHVFHN
LNGERLKVESSRAESQKYSPLLLEEARTVGLGAFSEEVLSENKFHEEIGM
PRRTSYPHDSALIHDDNTVSLGFQQVRLHPLL
> CP0144 mxiE MxiE, similarities to transc riptional activators of the AraC family, function unknown 123331:123963 forward MW:24552
MEGFFFVRNQNIKFSDNVNYHYRFNINSCAKFLAFWDYFSGALVEHSHAE
KCIHFYHENDLRDSCNTESMLDKLMLRFIFSSDQNVSNALAMIRMTESYH
LVLYLLRTIEKEKEVRIKSLTEHYGVSEAYFRSLCRKALGAKVKEQLNTW
RLVNGLLDVFLHNQ TITSAAMNNGYASTSHFSNEIKTRLGFSARELSNIT
FLVKKINEKI
> Q3CBI8_9CLOT
MIYIISDDLTGANDTGIQYTKQGFRTLVTVKTDVEFLETTSKSYDVISIN
VDTRSKLPDDAYHTVYDLVKKFENTGVDYIYKKIDSIVRGNPGVELDAVM
DASNAKIALVATSFPEVGRKLMNGKLELIDWEGNKSVIDVIELFTDDMKR
KVRGINLSTVKEGISSIVEVVERGTSEGVEVFVIDAESDE DLAVIKGAAT
SLKVPPVLCGSAGLAKQLSLSGREIFNKKSNEVTQIEDKATLILIGSRNN
ITSQQLKVLEEEMRIPVLTLMTGEVLNGGREAIIENTRGEVSSLIDNGCR
LLAIVVDTLFQGFTVQLKDSEAALMDSNYIAHAIGELAKLIYETNSIDTI
VSSGGDTSQKLLDALNAKGIHLESEILSGIPVGRVIDGIADGMTIVTKSG
GFGDQDSLIKVIEYLEDRKNKLAYA
> Q7DB74_ECO57
MSLSGAVFKTFLTSEHASWNRFNRRLHIPNEDIVDEIQLKARMQQRHHRV

YPEIGDSTIVSFRGKDYAVHFIKDGPKDDYVYKVQRITPENGCFSTLFSV
FSGGVTKALERKLNERHITPLSSTWFPRTPLEGILAERGLSSLLRRVQST
ERLDNRAIATRASSYSVL
> PSCF_PSEAE
MAQIFNPNPGNTLDTVANALKEQANAANKDVNDAIKALQGTDNADNPALL
AELQHKINKWSVIYNINSTV TRALRDLMQGILQKI
> Q8XR37_RALSO
MPRFSNCFIPTAHKPSSATPVSDTAAPTSGPEPTPAAAPAQPRPPRSARG
TLKDRLKQMAHLKHVPHKPAISVDHWDEHATLKLCEQWRTGQARQPSVPD
LARLAALNFERAHANSIPADQKARRLLPEGTSESVYYQAMIETFLKAVGI
EDNDVAATLESFKKVGTGGLHRQPVSIASAVGGAVSTAQYAVAANLPAKT
ALSGVQLLLTLLTTQLAFDSA DLRFRNAGTEEVMPLGRADTTPSAKTGPN
VLRTTGRLAWDLRKIRTNVSQMEKAQAALDGAQNAYDKAQATIAAAREAL
KAAQNALAAAQATPAAPKVTLFQHMQAESAITQAKTDIEQATRKIGQGQE
AAVQAEHAIQAAGETLKIAFARFCMRNELKSDYKTASESAKIEYHGNKRF
LGISVATGAANLTATILGILTPVVVSATVTTGVTAAAAALAAVLYVGYQL
SSGPSKDGEAKAKRAIVALAKS LDLLAGNAAKQQKARAAAYRTYIAERRI
WNRPEVRKNAKPKLVATLDEIARKDTTEHDLDPLKNWLGYAKHREAMEAA
GNDHDAARAIEEAFSQAHGAQFSAKTVADAWKTPERMRFDSMGRLLLGKL
GNAIGAVHRFNAETERAAPGESAREAFARRQIHAGRLADVKACLRDWISF
EQAQSQMKSALQEKDPDQARTLLRGAAQALAAVRDTDAQNLFSRDGRKQV
EATELAKRMTIGERERYTMTNAG PAALAGLVNIGGAAASLGLNIEKVVAE
SHGISMPAQYGDQNDARTLAQGSAPVTAPYVAAERARFQKTRMGKTVKTL
ARKQDDHVALKLELPAGKPMAPNTDYAAPDIDQALDKLIGQLEDLRDIPD
EIQLSIGGKALPSGKLSGTTGYLNWRYDNAPLQTKAKFQMRMMEMVADNL
AISVASPIAQGIAQVPLSMTRAAAHRGNAMSADVRDRLTRLAGQPADPQP
SPEHPPAGTQPVPQAEPPASPRRS APVAQAQPEQRQPAPGRVPGEFDPSA
ARHALSDVPMLGGEAPADAPAAPAALADAVFQPRPAGDPHAEAVVQQRRM
LVGSQGADATHQWLEARGIAAAPNSGATSMDCLIISLLQHATNRYDAASE
PALAREAARYRTALALQHPEILSGDRMLYADEPATGALIKMINETQQVSL
QLQMVLPTDDGPVQLPSPKTGHDPVGVVLFGNHFQALHQTHRDQDRTADA
LGANAGARDAQDSGRRIAPQSEGDA VGQPVPSTAGASPQRNAAGTPPRGP
QGGTPDIGPGDDAVSDDDFYSATASLSSVASGSESERDIEDAMRRAGRHD
SADTGKQARPEHPKPQADAAPEPATASPPAKRSNRMIRWLSKTLKGKPKP
DVQQTAAPADQQPPSMRR
> Q8XRK9_RALSO
MGLPRIPSIFRPSSTASPDARRTDPEHTPPHAPPRRPKNRGLGALLDLPR
LVRGRRDGASTSMDGPAIGAPPASPPAGFKLPRQNALNAEGIC ALERQFP
PPSSGPSSGPLLRPSPSGQASASNPLPVRTLAEQLLRREQILASQPPAIS
VASSPPYIPVDALPPPLSKAFLEAFDPIRSLGLTDQSVFYRALASRYLLA
DGHQMKLAGNPRSGAYVRHHHRLLPSSGLASRETFESLPATARERMLNDP
MQQYEWVRMRARDLPEPSLNVMFGVHAEAGARSYAKTSDHVVVSMTFGDL
RKAGGQVFLDTRASAGSDHTKALIVTLPKGRTVPVKIIPNGRQE ASTSTR

> gi|28868239|ref|NP|790858.1| avirulence protein AvrPpiB1(Pto)
[Pseudomonas syringae pv. tomato str. DC3000]
MHANPLSSFNRAQHGNLTNVEASQVKSAGTSSTTNIDSKNIEEHVADRLS
DLGRPDGGWFFEKSLGTLKNLNLEQLAGIHDVLKLTDGVKNIVSFGAREG
GFELAMQFRHDLYRSQHPDENSPHDAATHYLDAISL QSNKFTKLEKLQHV
DVFKMQNPFWDVGYKNGIAHAKKMAFFITPEWLGSDFCKQEFQWLSETKN
KDIKSAFVIFKDVDLKSKNMTSIFNFADFHKSRVMMASTPPESGLNNVKI
ENSVDLNFKRLLTDRESWELNNFLGD
> HopAB1 Pph race 6 -1448A|Genbank: AAZ37972
MPGINGAGPSNFFWQWRTDGEPVTEREHDSSRSASSANSPELPPPASPAE
SGRQRLLRSSALSRQTR EWLEATPARVQGATPPAEARQSPEAQQAERIVQ
ELVRGGADLNNVRTMLRNVMDNNAVAFSRVERDILLQHFPNMPMTGISSD
SVLANELRQRLRQTVRQQRIQSSTPARLADSSSGSSQRSLIGRSTMLMTP
GRSSSSSAAASRTSVDRHPQGLDLESARLASAARHNHSANQTNEALRRLT
QEGVDMERLRTSLGRYIMSLEPLPPDLRRALESVGINPFIPEELSLVDHP
VLNFSAALNRMLASRQTT TNSPELPPLASSAESGRRRLLRSPPLLSGQRE
WIEQSMRQEAEPQSSRLNRAVRLAVMPPQNENEDNVAYAIRLRRLNPGAD
VSRVVASFITDPAARQQVVNDIRAALDIAPQFSQLRTISKADAESEELGF
RDAADHPDNATSCLFGEELSLSNPDQQVIGLAVNPTDKPQPYSQEVNKAL

TFMDMKKLAQYLADKPEHPLNRQRLDAKNIAKYAFKIVP
> gi|12329139|emb|CAC05870.1| IpaH1.4, member of the IpaH family, secreted by the Mxi-Spa secretion machinery, function unknown [Shigella flexneri]
MIKSTNIQAIGSGIMHQINNVYSLTPLSLPMELTPSCNEFYLKTWSEWEK
NGTPGEQRNIAFNRLKICLQNQEAELNLSELDLKTLPDLPPQITTLEIRK
NLLTHLPDLPPMLKVIHAQFNQLESLPALPETLEEL NAGDNKIKELPFLP
ENLTHLRVHNNRLHILPLLPPELKLLVVSGNRLDSIPPFPDKLEGLALAN
NFIEQLPELPFSMNRAVLMNNNLTTLPESVLRLAQNAFVNVAGNPLSGHT
MRTLQQITTGPDYSGPRIFFSMGNSATISAPEHSLADAVTAWFPENKQSD
VSQIWHAFEHEEHANTFSAFLDRLSDTVSARNTSGFREQVAAWLEKLSAS
AELRQQSFAVAADATESCEDRVALTWNNLRKTLLVHQ ASEGLFDNDTGAL
LSLGREMFRLEILEDIARDKVRTLHFVDEIEVYLAFQTMLAEKLQLSTAV
KEMRFYGVSGVTANDLRTAEAMVRSREENEFTDWFSLWGPWHAVLKRTEA
DRWAQAEEQKYEMLENEYSQRVADRLKASGLSGDADAEREAGAQVMRETE
QQIYRQLTDEVLALRLSENGSNHIA
> Q1QSN5_CHRSD
MTMPIIGCIADDFTGATDVASMLVSAGMRTLQTIGVPSTALDDDVEAV VV
ALKSRTLPADEAVAQSLEALAWLQAQGCEQFYFKYCSTFDSTPAGNIGPV
TDALMTALDTDFTVACPALPANQRTVYNGYLFAGGVPLNESGMQDHPLTP
MTDANLVRVLGAQTHQRVDLIDYATLSEGVEAVEARMQALRSAGVGIAVC
DAIDTQHLHTLARACRHLPLVTAGSGLALGLPANLTDRLPAAGQADALPA
IAGREAILSGSCSRATLAQLEHAREHYPSHYLDALALADDFEGVIAAAL A
LAEEHLDRGPVLLYASASPEDVRRAQEVLGVAEAGALVERAMAEIAQSLV
EHHGVRRLLVAGGETSGAVVNALGVQGLRIGPSIDPGVPWTTTFGTPAPL
ALTLKSGNFGARDFMTKAWRMLP
> Q8XTC0_RALSO
MRASGPTSSPFHPEHEPMWRSNIGDQRVRHAPDAPDMQQQPASGQTSEPR
PEGSRAVRASNEALAPLAANRWSVLGSASNAGFRRSTMPLPKRPEPADGG
VDASSQVGDPR SNAAAIATYADEGCPADEVLKLTTSLLRIGGDFARSMAL
FAAVKDAGICPNVVTYSAAISACEKAGRMDEALVLLNELKAIGSDDPMMR
PNVVTYSAAISACEKAGRADHAVALLGELKALAAHDPTMRPNVVTYSATI
SACEKAGWVDEALALLDELRALGQSDPALRPNVITYSAAISACMGAGQAD
RARWLVAQAIRDEVVLPDAGYDARANTLDFHANLVFREPAPSSRPVGVAA
PVALALMSYHGH RGNLNGETHYIVGQHGGDAIKSAVLETLNRVRPRAYVV
SPTNAGMLIATAPDPYAVWSAEDDLPPSPRAASSALPDALADRLRAMWPQ
AGASEALAAGQSDAMSIDDAEAMSSDEEDDGLIRFHADPAARAAQLRDLK
ARVREAATAEDVLALVEAAGRLRAEAAERYPGASAEQLKDAFLPGSENVD
SNFRRLNDMRLDCDDAAQAWQAASDHYRSARDAALKLNLPGDFVTPLETA
EAECEAQATRLEI THGLLDGIWRFKGWRQSFDAGHPKSLDVVLKPLQRAL
ALGNAEALATLPFCDVPIQAEFHDQLNIIHGDAAQLVSAGGGVLPLALLN
RLRPEAQAARRAMSAAASAEIVKAIALLGPALKGEMPAAAPEPMLIDEPG
VSERAVSVALTELIAHLTPGIESARRVAALAQSHETGALSVGDQVRAFGL
WHRTVEVFGRARQAFEAVAASGWPSEALSAPDVQALHAEIRQGYDAAVGK
MQATAGRVFAAMWE QVRGSLGQVRDAQTVAQLMARMHVPDELGIHVADGC
QHMRSCVEDAPVALRAGGIDAARMVAPFTALQAFLRITRQPLAQPEQLLP
TAAHLLQVVTLAKEAGAGAASALKPALHAFSTFCADLRMQLVHTAHRIER
REVSTLCTSVSPDIWAAATRVEHRLRALVDPKAVTGQGITSAGTAEEPSD
AAMAAAADMAALRLEQRREGLLALPPGVPATLTRKQARSALLLRGIAAAG
RLTAELIRCHERAAP AMGEANLAQRAKALGKIAAEVQAAGAKAGEAFVDT
VTLGDAPESASPNSEAALLSAAVSRCEFLAEYMRFCVQTLADLAAGRRNA
QSRIRQPAGDASAYVREADAIQGIHDDIVDRLYEACERVVGLIEAKPAIG
DTREAIDAETNFVHELVDSLEWRAIVETDMTQRAWMRATLDVAAVSIDHA
SRDVLQGMSTMLAKIEDDLRESKGRLVRYAEKTGDQAYRASLKLEDVTLG
VGVARAQTRLIDRLNA LAGASRAQPSKSKGKGTKGKGKAK
> gi|28868581|ref|NP|791200.1| type III helper protein HrpW(Pto) [Pseudomonas syringae pv. tomato str. DC3000]
MSIGITPRPQQTTTPLDFSALSGKSPQPNTFGEQNTQQAIDPSALLFGSD
TQKDVNFGTPDSTVQNPQDASKPNDSQSNIAKLISALIMSLLQMLTNSNK
KQDTNQEQPDSQAPFQNN GGLGTPSADSGGGGTPDATGGGGGDTPSATGG
GGGDTPTATGGGGSGGGGTPTATGGGSGGTPTATGGGEGGVTPQITPQLA
NPNRTSGTGSVSDTAGSTEQAGKINVVKDTIKVGAGEVFDGHGATFTADK
SMGNGDQGENQKPMFELAEGATLKNVNLGENEVDGIHVKAKNAQEVTIDN
VHAQNVGEDLITVKGEGGAAVTNLNIKNSSAKGADDKVVQLNANTHLKID

NFKADDFGTMVRTNGGKQF DDMSIELNGIEANHGKFALVKSDSDDLKLAT
GNIAMTDVKHAYDKTQASTQHTEL
> gi|16766171|ref|NP|461786.1| secreted effector protein [Salmonella typhimurium LT2]
MIFSVQELSCGGKSMLSPTTRNMGASLSPQPDVSGELNTEALTCIVERLE
SEIIDGSWIHISYEETDLEMMPFLVAQANKKYPELNLKFVMSVHELVSSI
KETRMEGVESA RFLVNMGSSGIHISVVDFRVMDGKTSVILFEPAACSAFG
PALLALRTKAALEREQLPDCYFAMVELDIQRSSSECGIFSLALAKKLQLE
FMNLVKIHEDNICERLCGEEPFLPSDKADRYLPVSFYKHTQGAQRLNEYV
EANPAAGSSIVNKKNETLYERFDNNAVMLNDKKLSISAHKKRIAEYKSLL
KP
> gi|27376921|ref|NP|768450.1| hypothetical protein bll1810 [Bradyrhizobium japonicum USDA 110]
MDFNAVAPANTSPEPDTARTATDATEFERQLSGSEAPASAQGVAHPVLQG
EAYSPYLDAGHPYSPYLETGHLYPPYPDLAHPLGPDSGWQDNLYAAPAAV
AAPEPDNGQQHLSPQAIAQAIEEHPGFDQDVIWQTLDVGPSEAEPRHGEP
QAGTSHAGPSRTAPFSG
> SOPE2_SALPA
MANITLSTQHYRIHRSDVEPVKEKTTDKDVFAKSI TAVRNSFISLSTSLS
DRFSLHLQTDIPTTHFHRGSASEGRAVLTSKTVKDFMLQKLNSLDIKGNA
SKDPAYARQTCEAMLSAVYSNNKDHCCKLLISKGVSITPFLKEIGEAAQN
AGLPGEIKNGVFTPGGAGANPFVVPLIAAASIKYPHMFINHNQQVSFKAY
AEKIVMKEVTPLFNKGTMPTPQQFQLTIENIANKYLQNAS
> SOPB_SALHO
PAKEARRLAAADFKSAQVKQLNNQPWQTIKNTL THNGHQYTSTQVPAAEM
KIGAQDIFPKAYQGKGVCSWDTQNIHHATNLWMSTISVHEDGEDKTLFSG
IRHGVLSPYHVEDPLLRQTGAESRAKEVLTAALFSKPELLTRALKGEAVS
LKLVSVCLLTASNVLGQEGTMVKEQMRAWQSLTQPGKMIHLKIRNDDGEL
QTVKIKPEVAAFNVGVNELALKFGFGLKASDSYNIEALQQLLGNDLRPEA
RPGGWVGEWLARYPDNDESVNTLARQIKDIWQNK LHHKDGGEPYKLAQRL
AMLANEIDVVPAWNCKSGKDRTGMMDSETKREAISFHQTHTLSSPGSLPD
RSGQQIFQKVLLNSGNLEIQKQNTSGAGNKVIKNLSPEVLNLSYHKRIGD
ENTWQSVKGISTLIIS
> gi|34497747|ref|NP|901962.1| hypothetical protein CV2292 [Chromobacterium violaceum ATCC 12472]
MPHTMSSSINLNTRQSAFSLSN ERAARMMSGDKQTATHMGLWDRFKDLFR
SEKKQDALNALFDLVNSQQGPARSFHVFNQLAQMAAPDNRTLFTVALTSD
LAGCKASYCISGHPVRTEQVSPEEREVMLARLGVPPGGEHVLTQASSLDD
CRLEITPHQLNALRDADFGAGGVHKRFHAATGVLQARDNIGREDFVRESR
LQEYAESRPDLQHYISTQRQIDAPPGVSDKFAYAQVPLYDSARVASRELD
AALPKLSQDQARSMAAQLVDMAR AFYCNQLSHRDLHMQNLLVHERLEDGA
VFLKAIDFGRAKFGGAEFEADRFNDIDYLFDRQGCSLAETVGRNYLAGKE
SAVAKKHYPLHKLLERFNNRGADVTETLSGIGQRLKADLRMAGDDPARID
QAFASAAVETQTALSWGARPR
> gi|34498036|ref|NP|902251.1| hypothetical protein CV2581 [Chromobacterium violaceum ATCC 12472]
MNEMTQLKSMIDGLKKRRMGFKKQLDDFKALQGEIAALEARAESDPVARG
KLQKLEAMMKSGGEQLHQQIVSKVAMAEKTFSQLGKQLKQLAPEDDAKAS
AMAEPVKAAPVLAKKHRRSFV
> gi|51591579|emb|CAF25383.1| yop targeting protein yopK, yopQ [Yersinia pseudotuberculosis IP 32953]
MFIKDTYNMRALCTALEQSAPDTIINTSKEENNSYY CATAHLLRTDVCSL
VNRVGIEPLKSGSILSTLEELWQAVGIVYRLYEWQHVSDIDTNFKKLPNN
SDFGLVFSVLDCDIEYVFIGKKDSEGNIEFYDPKNSLLIENDDIKKYLYD
ENFHRFCIMLIISKSELEELSRESCDQECIMG
> gi|28868590|ref|NP|791209.1| type III helper protein HrpZ(Pto) [Pseudomonas syringae pv. tomato str. DC3 000]
MQALNSISSLQTSASLFPVSLNSDVSANTSTSSKELKAVIDQLVQALTQS
GQLDETSPLGKMLAKAMAADGKSANSIDDITASLDKLIHEKLGDNFGASA
GIGAGGGGGGIGGAGSGSGVGGGLSSDAGAGQSDLMSQVLNGLGKAVLDD
LLTPSGEGGTTFSSDDMPTLEKVAQFMDDNKAQFPTRDGGSWMNELKEDN
GLDAQETAQFRSALDVIGQQLGQQQGDASGVTSGGGLGSPVSDSSLG NPA
IDANTGPAANGNASVDVGQLIGQLIDRGLQSVSSGGGLGTPVDNSTQPTG

GTPAANPTGNVSNQDLGQLLSGLLQRGLEATLQDAGNTGADLQSSAAQVA
AQLINALLQGTNNQTNQAVA
> Q8XSZ4_RALSO
MPKIPKNLFHAIGSVTQHINETRAPLRSVQTARERSAPLPKAPKTASEGQ
RSGFSRFGSRVMKGILGAGAMAYTYDTIANHFFLSTTALHDGKYGFTSDE
RLDKARPEAEEN YQIYQSRTPVEQQAHNKSVNPFRLCGENHFVTMTDYRV
ATRVYLSRLVDSKAAHALMSNNVQCLKGTHVKKEAVEQFNPTKLPKNFDL
TQSAAYDRKNKYSLIGVRNEETGSFGYTSRSATHPFVAEGWDHFFEATRG
ENGVTPKQCVETLEALLERGDTLSAEAQFAAGQLLLIYRQAYADDENWGN
AESVVLADLYRHGLASKAEADKIELTRPPYQEDLDMGWARRNTSLLGPML
QHTDIWWQEHILR KDPETIKSINNLHVADLQYTPLSHFKLNEQGNGFEDC
SGLGDSFTSLNATACLNHARLMSGRERLSRDEVIVLIACLNAVYDNAGGI
RHTLQEIARGCFVGAGYTVAEADDFYRRLCKNAAEMFYGGRNLKPLVRPE
LPEVISAASPAA
> avrB1 Pgy race 4|Genbank: M21965
MGCVSSKSTTVLSPQTSFNEASRTSFRALPGPSQRQLEVYDQCLIGAARW
PDDSSKSNTPENRAYCQ SMYNSIRSAGDEISRGGITSFEELWGRATEWRL
SKLQRGEPLYSAFASERTSDTDAVTPLVKPYKSVLARVVDHEDAHDEIMQ
DNLFGDLNVKVYRQTAYLHGNVIPLNTFRVATDTEYLRDRVAHLRTELGA
KALKQHLQRYNPDRIDHTNASYLPIIKDHLNDLYRQAISSDLSQAELISL
IARTHWWAASAMPDQRGSAAKAEFAARAIASAHGIELPPFRNGNVSDIEA
MLSGEEEFVEKYRSLLDS DCF
> Q3BTM6|Q3BTM6_XANC5 Xanthomonas outer protein J - Xanthomonas campestris pv. vesicatoria (strain 85-10).
MGLCVSKPSVAGSPEHYAAHVAEQATPSEEGSGTPAQATSSYSATDPALQ
GLARRGKKVDLSRSGLPERVALKTKLLANSLSDAKDAGVRSSIVKYGERT
LKMLAANKQPDEALLKLDIKNLPALASVYNRRYPGLNIQHFDS PIDFLQS
LSEQTTSVVSQRAVLRLERDGEHHVAADVRRRPNGEASVIVLEPARLLTF
VTGHTQLRRQALSQLGENAKFAFIQVGAQKSAADCLMFDLHFALHAHQHS
SLFDQWHDNMVNHGTIDPYGQGAASPDALLEDAGVELMLGEQMLPAIFYK
HTHSSGVVEEVDRSQPGSAYTDVSTSGRQQQHESLEQRVQAFRVDRGYSA
SIETSRATKIRSALETAISDRQS
> gi|28869866|ref|NP|792485.1| type III helper protein HopPtoP [Pseudomonas syringae pv. tomato str. DC3000]
MTMGVSPIRNSNSLPIDFSSLSAKSGGHNGLGSGDNSTIDPSTLLFGNQG
QTQVNFAPPNSTDSSTSGVNAASGNTASGLVEQIMSLLKQLMQMLMQNNN
ASGNPQTDSSTPGVGSGNSVGSGGTGSSLAGSDGGDETSGVGNGGLGDAG
STPTTSAADGVPSD TSLTGSGGLHLPQQLEQYRGDIMDAAKATGVPPSVI
AGQIWAESRGQLNAATTNVNGKADAGLMQVNADTFKSLQQQNPGLLGNDV
NDSHTNIMAGALYLRDQNKEFGDMGAALRAYNSGPDKVNKADLSDTGGVG
GSSYPADVLNFAKIIESGQGNLPA
> Q8XUA3_RALSO
MPIETRYSNIYAQSLVNTREALEDFKQDAHQALDIINSRPAGNQLLSELS
ELCRARRHKITIHELNSNEEPCSEPV LSRHQIEEYSPENFRENREKACDL
AEKRSGWFGKKPGEGASVIVSWSMSHSSMTFSANGSPTGTCPSNTDKVSQ
LAHELVHAKHMVAGTWKGRWGDDRDPTTSAGKEELRAVGLGKYAETGEPS
ENAIRAEHGLPLRRKYY
> gi|24210995|ref|NP|710166.1| AopP protein [Aeromonas salmonicida subsp. salmonicida]
MNIPPIHIKTDLTNQDEKTTIQEA TKEELQLLIATMERELASGEFFTSHE
NYASIDLGKMPLLIEAANNKHVGLNLNFVSNPIDLPSEIGRAISNGKEQF
RYVVNMGESGIHFAAIDCKMVDGKLSLLLMEPANLNSMGPAMLAMRVSSC
LKREAEIIPKPHFCIAVMDIQRSNSECGIFSVGLAKKMFSERAPLDALHE
EILSERLPDGMKCDVLEGEALDRLLPPTFYKHAQSQRRLDQYIRAHPDGN
DTSVNKKGELLLDRAKRLMVPVDEK LISSSIHQKRIMEYSAISDDGKSV
> Q4KBD3_PSEF5
MTISNPRPLLGCIADDFTGATDLANMLVRGGMRTVQSIGIPSAEVAAGLD
ADAVVIALKSRTTAASEAVAESLAALQWLRDQGCEQIFFKYCSTFDSTAA
GNIGQVSEALLEALGSDFTLACPAFPENGRTIFRGHLFVQDQLLSESGMQ
HHPLTPMTDANLVRVLQSQTRLPVGLLRYDSIAQGVEAVRSRIAELRGQG
VALAIADALSDA DLYTLGAACADLPLLTGGSGLALGLPENFRRAGKLRDL
DAASLPKVAGGEVVLAGSASLATNAQVDAWLEAERPAWRIDPLALAAGEA
VVEQALAFAREQQGTVLIYATSTPEEVKAVQRQLGAERAGALVENALGEI

ARGLRDSGVRRFVVAGGETSGAVVKALDVRLLQIGAQIDPGVPATVSSGG
EPLALALKSGNFGGRDFFSKALGQLAGGQA
> Q8X606_ECO57
MITHIPRSSFSANINNTA QTNEHQTLSELFYKELEDKFSGKELATPLLKS
FSENCRHNGRHIFSNKDFVIKFSISVLQADKKEITIINKNENTTLTQTIA
PIFEEYLMEILPQRSDALDKKELNLNSDRKEKEFPRVKLNGQCYFPGRPQ
NRIVCRHIAAQYINDIYQNVDYKPHQDDYSSAEKFLTHFNKKCKNQTLAL
ISSRPEGRCVAACGDFGLVMKAYFDKMESNDLSVMAAILLVDNHALTVRL
RIKNTTEGCIHYVVSVYDP NVTNDKIRIMSESKEDIKHYSLMDFMNVDYS
LLKWSNDHVINQSVAIIPALPKEQLLMLKGTVDEITPPLSPATMNLLMAI
GQNHQLKQLMIQLQKMPELHRTEMLTAYNSINLPGLYLAINYGNADIVET
IFNSLSEPGYEGLLSKKNLMHILEAKDKNGFSGLFLAISRKDKNVVTSIL
NALPKLAATHHLDNEQVYKFLSAKNSTSSHVLYHVMANGDADMLKIVLDA
LSLLIRTCHLTKEQVLDLLK AKDFYGCPGLYLAMQNGHSDIVKVILEALP
SLAQEINISASDIVDLLTAKSLARDTGLFMAMQRGHMNVINTIFNALPTL
FNTFKFDKKNMKPLLLANNSNEYPGLFSAIQHKQQNVVEMVYLALSDHAR
LFGFTAEDIMDFWQHKAPQKYSAFELACELGHRVIAELIFNTLNKMAESF
GFTDNPRYIAEKNYMEALLKKASPHTVR
> gi|17547494|ref|NP|520896.1| PROBABLE HAR PIN-RELATED PROTEIN [Ralstonia solanacearum GMI1000]
MSIQIDRPNNHFQTPSTWNHDAGSSIDTSQLQRAVQLLDQVLQQLEARKL
FGNMLNQPGADNAGQNHAGGHGGGHHGGSNGFGENGRFGSPHANSPAQPD
LELPANKPNNGKHNTSASTPDTQTAPSSTSPTTGTSPTPTSTSATEGKVA
YGVKPPEPTGVVDVSKPIVVKAGETFDGGGKYYRPTKEMGDGSQNEHQKP
LFILEPGATLKNVQYSGGDGIHLLGSAKLDRVVNRQVGEDAITIDGAKNR
AHDAKIAGIDPASIPGGTPKVEITNSAFYGAKDKLAQINGDVDLQVKGMY
VNGAGKVFRTNGGDTQIKATVNVQDSNFQNVSEAVFRTDSKFSTASFSDD
VKSDAPFDGLAPDKSQVTGTNKVSYKAYSG
> gi|51591596|emb|CAF25400.1| lcrV; putative V antigen, antihost protein/regulator [Yersinia pseudotuberculosis IP 32953]
MIRAYEQNPQHFIEDLEKVRVEQLTGHGSSVLEELVQLVKDKNIDISIKY
DPRKDSEVFANRVITDDIELLKKILAYFLPEDAILKGGHYDNQLQNGIKR
VKEFLESSPNTQWELRAFMAVIHFSLTADRIDDDILKVIVDSMNHHGDAR
SKLREELAELTAELKIYSVIQAEINKHLSSGGTINIHDKSINLMDKNLYG
YTDEEIFKASAEYKILEKMPQTTIQEGETEKKIVSIKNFLESEKKRTGAL
GNLKDSYSYNKDNNELSHFATTCSDKSRPLNDLVSQKTTQLSDITSRFNS
AIEALNRFIQKYDSVMQRLLDDTSGK
> hrpA3 Pma R1|Genbank: BAD20871
MSASSMGTKLVNGIGNGMQGLSDINSAKDRQTSLYKNTGSNDSTDSVYDN
LRKGDKENGKLQDISTEEGAKRREESMMAGFEAADEKLANQIVA KKIENA
VVQF
> A1JQ86_YERE8
MVDIKAGSDRINNILTFTQGANFNQIRDDSTKNLFDVSLGDAVQELASME
QEKHHRVKGAPRLVMPKMTLAQAKSEAAANQPSAEEAAGASISARHGANS
EATSQTSGFNAAASLMGSLATVRQIVSDSSLSSLRGRLQMINIESSVQRE
RAENLLSAFENSTNELQQSSNEVTKCQLAWQQSVTTVKGLTGQQLELQSS
LNTNQQKLTTTQQQLAKTTAELKVI SPPQDLAQLKQHELLTDKVSGLSIR
LSALQVEQQQLSTNLTKLKGTLTKAQLNSTQLENAYKQVLNQATEIAILA
DQDRIAINEFIESSVTTPKIDGERWVNQLAILALLTAELRMVMGKDAIKD
MEKKQEVLETISAASRRDSEKKAKEAEAAQRKADEASKTASCTSKIFSYI
MLAVSVVATIATLGTAAPLTLAIAAVGIALTVTDIVLEETGQGSLMQMLA
AEISTGISNMLMTFGMSAEKAKEIGN IVGMVLAAVAFLAISLFSMSSFFK
NAGQMISNLAKNGSKLVGNLMKSAVKALPTDLLNSMGKVGTKATNLSKSL
AQIADKTDDVVHVADKAASVGKTASVTARKIEMGMNGTNMVLGVTNAAVS
GGLNLQVGTNTRDMKEMMAGLMHNNAMMQFIEDLLKSLIASVAKNYDQLN
EMFNSMLTALDQSGTAKANIIKSSFA
> gi|34498071|ref|NP|902286.1| invasion protein [Ch romobacterium violaceum ATCC 12472]
MPITGASHAAMRPLSAPPDGTAGPASPLGRQLRDVRADSVEPARRHSLAD
LASYQADYARRSVATLFAASPHADKLGELYQASPNRYAKLEIAEFAKVYS
QLKRQPDLDPAAGKTLDDLAQQYAARILKDGLGEKSAFGPWTQRTDKHYQ
LRSGLERKLAEIASRHCQGDAQKLGNDFMRAEVTTFILSCVETHLGRQLD
EATSRQITGLVDSAAM QAFEDLRQRRGDLIEQRGFSVGTLARDLDTVAVL

PQLLRSLLEALPPGPGQRAPEEPARDGPARPTPSPDPGPAGPGEAQRPQE
IHYHIDNSIHWNDNSQDNRRWNRRGDTYLGGARQGDRHLRSSGFPASGLA
ASLRTAASQDLPKPQQAVLSGPASPAAGRHPLLNAVDQVGQSLSGLVDAA
IGTAGTRPLSPGGAAESSAVRLAGLGSDGLSALPPRDDPGASLHGAARAV
ADSLSAVLTAADGAKIT KIATPARLDAAASGEDAQQPAAGEAIPADAGQP
GAGGFDGIRFRDGNLYMLPTQAYLRGLASPRRAENELLRAVRGALEPAAA
QPMAQRREFEALRNRILPSDRFDQDKVLDRFASGGEDRLADDAARLRQAL
AGHPGLERHRQALRSFARTLIREANLLPSAKPANPLVAGLLDALGIQADE
EARRAPLPSKPCGVVLTTDGLHVDSSRAGGRRGAS
> Q8X4W3_ECO57
MPMNTTGMSFSSFGISCH RENSFRNSFRGKNDEVVKCSIGERTISFSVRK
FSGNILETVRRQSTKDIDEWIKDERIVYPSRVINQEIDNYCFQKNAKIST
EERQRVFFLVSQENQLTLDVKAAQSSINHVIMGSASFGKKMDALCDGMSR
DVKNRTSDTIANLLADKFYQKHIDSDIDIVKLRNDIPDYLMRAIQG
> Q8XB16_ECO57
MDNLIGTPPNHAVPHNYIDMEQMEYLCHLNRFSKLSNDFLINGPREDLFT
YKYVLDGS FSNLQHLLPKGQLQKIQQRLSSLMHKNMFHCFHVFLLKLCSI
ESIPLPNADYAFFDDEMPFTLTDEQIENISFLNAYHKEKKGNNDIVTFDF
MSADHHYNFSTTIALTNDSFHISSINNHNSQVIFDENIHLHPYELPESSQ
WCYQLIKNMISLHCRYNNNFKIN
> avrRps4 Pph race 6 -1448A|Genbank: AAZ38042
MNRISTSSVNSSFSYSAPAEEAQNRVSSAPTNSSPPGTTTAV AQASEGQK
KPGAVLSMQAQRLRQLCESSSYQYRQNALLAKSFDAQRLDINTQAGPSNS
PHLNALNKLQQRDFKPAAGGLEIPFTPKSLLGGGKRVYQIGSSSRDVQVC
PRGAGAALRQEIADKQLMVNHLTDELQDAIDEANQAEIANTSQQLRQARS
DLAELQRRFAVLGNEDRRINQ
> gi|15618110|ref|NP|224395.1| similarity to CT119 IncA [Chlamydophila pneumoniae CWL029]
MSSPVNNTPSAPNIPIPAPTTPGIPTTKPRSSFIEKVIIVAKYILFAIAA
TSGALGTILGLSGALTPGIGIALLVIFFVSMVLLGLILKDSISGGEERRL
REEVSRFTSENQRLTVITTTLETEVKDLKAAKDQLTLEIEAFRNENGNLK
TTAEDLEEQVSKLSEQLEALERINQLIQANAGDAQEISSELKKLISGWDS
KVVEQINTSIQALKVLLGQEWVQEAQTHVKAMQE QIQALQAEILGMHNQS
TALQKSVENLLVQDQALTRVVGELLESENKLSQACSALRQEIEKLAQHET
SLQQRIDAMLAQEQNLAEQVTALEKMKQEAQKAESEFIACVRDRTFGRRE
TPPPTTPVVEGDESQEEDEGGTPPVSQPSSPVDRATGDGQ
> gi|51591597|emb|CAF25401.1| lcrG; putative Yop regulator [Yersinia pseudotuberculosis IP 32953]
MKSSHFDEYDKTLKQAELAIADSDHRAKLLQEMCADIGLTPEAVMKIFAG
RSAEEIKPAERELLDEIKRQRERQPQHPYDGKRPRKPTMMRGQII
> Q8XVD4_RALSO
MSRTLATPRTPQGNDSLFASYEAIASLSTEMVEAAEAGDWAGVSALEREC
ALYMERLGRTPSRPVLSEAELRRKRDLMMRILANDARVRALVCPRQDQLL
RPMDAARRSIGARQAYAAVSYY
> Q3BRE0|Q3BRE0_X ANC5 Xanthomonas outer protein F2 - Xanthomonas campestris pv. vesicatoria (strain 85 -10).
MKLQRQNSAPPSIPSGSSANPGAAHASEPFVPEPQTPRSTVLKGLAEPSP
RLRARAGKTTTLPSGELSPAKTRQWRPEAGESSRAASERAERQHRAGRHP
GNQQAASSSATQGAYAQRCVAACERLKAQLRAGLKARPFAEPSDEQLALQ
AEYVQWADARLQ ERIEAFGPDAAYEVAGDMKAVGRKASLPIAYESLRSFF
VGALRTPLGISAATAYQSNRPPASEPLSTATLAGVISGMGSHACDTLLIP
SMDRRARVANLPRFQAIDPKILVPDPPPVLLEITPEGSKRFVRPGENGTP
TRAELTAQAYDRRAGIAQRQSVLDDTALDTMLLRPTISGGFNAARRTSGD
PSTRTAVAEVGLAGLAVGGAGALHKALLDTGKAMGRTGQMTVPDLLGGHQ
RLNLFALALPDKT RPPATWSDAVHFPSYLLQTGQEAMALARQAFSTANAA
TTALRDVLGRHMVGNVLANMASLGATRLIASPLRGGYGGGSLPGEADNSP
AWVLQQGAQMFFNDVGWNALKAKNGTNTTQATRLDHERAIAAAEQDRTIS
RTLQTLAEPLDTALGLLPPPAQGEVPRAMEEGTGVPVPLSADVDRLRNAL
QQLRDQVAAQAVSIATIDDALGRLADLPVAGDGEGRAVIAELSAQLHALG
AALRQKQALQQWQQ GRP
> Q8XT97_RALSO
MPISACKPHRSATTIISGDIQISSSQEAPSKKSAKEPRLETNPLLSKLRP
RSSSVTAGAQSETSSSLRIKAAPFLTVPKSLDAYAIDSSVPYQFQPSKIA

IIGGGLTGVISALKLRSLGHEVSLFESNEDICLEASKIPAHCYGAPGYCH
LPKDEQIEIFRSGLAFAKLFPQAISLRPTIFALRKNDRVRVTNDQNGRFR
TIDDLKNSVNLAENEYKKAVESDPKSEVFGPVG KYQSGYTHEQIQALRNQ
PLTGAPGNNDEWVANWAHEMSEEALSELQYPVFLVKEPEINMLRFRELAR
SALGELGVDTHIGETVNDLTSQAGKPGIMVNQAHFDYAVNCSGAESGTHD
DKREAISERTVIVKGAGVASLPSVPFR
> gi|28868073|ref|NP|790692.1| type III helper protein HopPmaG(Pto) [Pseudomonas syringae pv. tomato str. DC 3000]
MRSRVITTSLVVIMLSCASAAPACFSADMTPSVSNESTSEADFQQWLATF
RSNATTKGIDTATLDLAFQNITLDPTVHQLDMAQPEFTTAIWDYLSERLT
PKNIQQGQELLQKEPVLNEVERHYGVDAKIIAAIWCIESGYGKDIGSRDV
IRSLATLAYKGRRMDYGATQLMAALHIVQNKDIARAQLIGSWAGAMGQTQ
FIPTTYLDYAVDFNHDNRRDVWSSRADALASTASYLQRSAWNSRVS WGQE
VQLPENFDYAQADMSIKKPVAEWQRLGVMGTKQAIPGELAQEQASVLLPA
GYRGPAFMVLSNFRSILRYNNSTAYALTIGLLADSYAGGTGVSHPWPTDN
PPLGSIAQVTDLQKLLTAKGYSLGAADGVIGAMTRAAIRAYQKDQHLPPD
GYASTVLLESLRR
> gi|15618928|ref|NP|225214.1| hypothetical protein CPn1020 [Chlamydophila pneumoniae CWL029]
MSSWLSQASEVLLNQDPYIPDAPRSQESSVPKISYSITVAPQEAQKSLPK
FFTQKFQSQCKSEPPITHHKTFIIATPRERILRFGSSFESQLHNTSQAQT
SSPWNLFSQKNSTEASKALMQELTMPKSPEKTSEKALDKNLSSKQEGSCK
NFDTLHLQQHLKLFGTVDSLYSQSLDSEQQELLQSRREERSETYANQQSS
EKKIETKVQIKDLCKDLFSQDQDSNQKQKKSPFQQDTSRKNR IAKAAQAV
PVIPPPSIGVFTLSYLLTKQGILSDFSSYGCHKDSVESTQRELDALHEKR
IETIKVSIEKEKRERLWGSLSDIIGWLAPFVSIGIGIVAILSGGGIFAFA
GFFAGLISLVIKCLEKLKFWDWLEKHLPIKNEELRRKIITIIQWVVYLTP
VILSICTLKVENLGFSPIIEGAIKGIQPAIESTMAALRCAILFSQAEIYK
LKGKLTKIQLDIELKSFDRDDHYERSQELLDNMESSFEALSRI LNYMREL
DQVYLHSLRG
> hopAH2 Pph race 6-1448A|Genbank: AAZ34780
MNIRSDRSYDPLMAAREKPVMAGRTTRAVGAGATEANPDTQEKLDAYTAS
DSRSAEAPEAFPVYTRTMLSRNARSLESEASPSDMALLASQQTLATQGDT
KALRDEVAAVKDNATPSAPETKVTDKDKVASAEPVKIAEKVSRGGANANP
DDIATPAAATKMIQDLKTIGGGTLRFQMTHDQIKDARQMD KLRALLSEGD
KQGVKVQFTFRDNANQGGGNVLTGEKLKQASADISNVVKKFGSRTSFVLD
TFNQGGKSASQDWADMQTTLIKAARNSGYKGTIVVEDSNWGGGLTAGPQS
GLVKFADQLKAANGEGNPALIGSFHVYARESEASSRLGKQIKALREAGYK
FQIGEVGNAKFLVGNTFQQKDEATKALQDNMTALKAAGADILPGKDQFQD
GKLRRRAGFSKSDQFL
> Q7PC42_PSEU2
MTQTPPSLDFNLSTPSPVPMTPSDTWAGASAALKRLDELRTLLARELDAL
PQAGEALLSALDGADVSERELQIFGLLQQIDDYWTDPGETGESRRDRLLP
ALQRSLHDEARVRIHERDFDSGYLACLPDSPDQEGPALAYSTVRVQLHDD
EQIEMAGVLVISQDQGRTLLMLPGLGISGFATQAMLVATLVQWLNTPTLR
DALLSNAQRQHQERLTEILQDADLYLEPFTAADVQLQPVVTTAPFIHAFD
RLLNKQRNDI RYACEQPGTADRLKRQSLIQQAIDMPGLFGPAAMLELREL
TNRRRQYQRNLPEWMKIASAADLQTYALHLQRYDAAHTAMLSVLGSAASP
ERFAEMQLRTRLANDLGHDLDPRDLTIDTRRTLPATSETYRVKLPLTELA
LYGLHPDDETAGSDFLDQTLITLDGQPLDAAYSDLNPAYLARVIDELDLR
AVFGDFQRQAYQQEHNQQMLRALARVRLTTLGWAAKMQGHIRPDDFAIVA
ALTSAATGTHD PALRVQQIRLNDRNVMARLLVCRKQDAQGRTQRLIMFAS
EAPGQQYFKAFDTDTQLLHEMVGWTASPAMTAWLLDQVEVAARPELAAQL
TALREKPQPAKDFLQFIDHPDCETALRRFTDEQTRVLLSEQARHTPDWYL
RASRAQRRELLALERAINGALDNYQAQAHTGVQSFKDYVHQRASQQLGKL
LGVPAGTVDPDQIVITSERETQTYTDMLLKGYNDSIDPLSTSAATDATFS
GPPGIDLSALSP AAVAGSVRGQWLADEYIALIRNTLLNSENDGYAYRRQY
SVMITQLQMKAAALRSLLKGHVEAAQYAWLTQSLDNAHLSDPASRERYPL
YPLQIHVDKPLIASGLTDVDQLVIPSPLLTHIETVQGCLVILPTQIRHAA
LLYTPQAPDGIEFRLFSDFVSSLNSEGMIDYYKDRCRIKARRTLSFFLRD
MQKGNANKPPVIPRAFIADVADTCFNRPLERRLRDVEETTTGRNDMLTKL
IRVSVEIVATALT LPFPPASFAVGSLISLHDSGQALAALSAGDRERATNY
MLSALLNGLGAGSDLLVGLKGLGGALHQLENSQHSTPVLRSFQRQPSLPR

YEDLYPVELQEQVFLLGKPNVHGHAAIFQASHVASAPPLATGQFAAREIG
GAWQPLLPLPPTAQRAPSGLRIDLAVDISLENVPRIAEGHAKGVHAINGK
YYIQLSDQAFEVQYDAYWQYWQIIDPANPFAFFGKQPVRLNDQGQWLLVE
RQRLRGGGLDTPGT YRPLPEQASASSSSLNTLSDYEMPSGMRAHLDIVIN
KEVFDPTGAGLDVYFETYFTEVRQTFTARREQLYQDAQAFFARFTPPPRP
TLPAYGLPGSVDTLIKHIFSHSNGLVFSEAPKSVASKRLLLLNMPLLAEQ
RVEVLYIEHLLTDKHLRKLARYRQLGKKSRSGSHELKYYLQETNRGALNN
ASSEFDYYHLIKAAHRYGIEVRPLSSSISYPFLGHTVLSAADDPAAAMKM
SNFFGHRLINHDIEP ASARRWVALLDQKLATTHDQVPGIAEMQGAVSVQV
QDIPAGRPTRIRQGAGEAHENTPSHCDFSIAFADPTLPARPLPPATALDN
TLIRELGDPTAIAEGERWAGEYGFVLDENHTWLRVEADDWSVDRSMTAIQ
QSLTDATYEMPPDTRTTLHRLANFEKKGLDMEYFFEDIELDTVRNTFDLR
RKNLQKDAASISAAQLPPRPTLPAIEPQTSTAGLLETLYRHTEGLVIGES
HSSVASKKLIIDNLPL LSQQNVKTLYMEHLLSDLHQADLDRFLETGQMSK
TLLHDLKTLDRGHHTDPNGVYTFEQLVIRARQQGLEIRAIDCASSYHLKG
IAEDEPLIRQQMMNYFASRTIRRHQDVMGSHKWIALVGNSHSNTYQGVVP
GIAELEGGIGLRVVDVAPGHSRGITLDQGELVSGGIADDKVYIKADYRVE
MEVPRPQPARLPSIEKRLSRPGMFLVQEGEGNLQTIVHRARDTWIHRTPV
LRNAEGKLYIERLRWPR IHLKPFDDIDALVAALEAMNLTRAT
> gi|2695858|emb|CAA74156.1| DspA protein [Erwinia amylovora]
MELKSLGTEHKAAVHTAAHNPVGHGVALQQGSSSSSPQNAAASLAAEGKN
RGKMPRIHQPSTAADGISAAHQQKKSFSLRGCLGTKKFSRSAPQGQPGTT
HSKGATLRDLLARDDGETQHEAAAPDAARLTRSGGVKRRNMDDMAGRPMV
KGGSGEDKVPTQQKR HQLNNFGQMRQTMLSKMAHPASANAGDRLQHSPPH
IPGSHHEIKEEPVGSTSKATTAHADRVEIAQEDDDSEFQQLHQQRLARER
ENPPQPPKLGVATPISARFQPKLTAVAESVLEGTDTTQSPLKPQSMLKGS
GAGVTPLAVTLDKGKLQLAPDNPPALNTLLKQTLGKDTQHYLAHHASSDG
SQHLLLDNKGHLFDIKSTATSYSVLHNSHPGEIKGKLAQAGTGSVSVDGK
SGKISLGSGTQSHNKT MLSQPGEAHRSLLTGIWQHPAGAARPQGESIRLH
DDKIHILHPELGVWQSADKDTHSQLSRQADGKLYALKDNRTLQNLSDNKS
SEKLVDKIKSYSVDQRGQVAILTDTPGRHKMSIMPSLDASPESHISLSLH
FADAHQGLLHGKSELEAQSVAISHGRLVVADSEGKLFSAAIPKQGDGNEL
KMKAMPQHALDEHFGHDHQISGFFHDDHGQLNALVKNNFRQQHACPLGND
HQFHPGWNLTDALVIDN QLGLHHTNPEPHEILDMGHLGSLALQEGKLHYF
DQLTKGWTGAESDCKQLKKGLDGAAYLLKDGEVKRLNINQSTSSIKHGTE
NVFSLPHVRNKPEPGDALQGLNKDDKAQAMAVIGVNKYLALTEKGDIRSF
QIKPGTQQLERPAQTLSREGISGELKDIHVDHKQNLYALTHEGEVFHQPR
EAWQNGAESSSWHKLALPQSESKLKSLDMSHEHKPIATFEDGSQHQLKAG
GWHAYAAPERGPLAVGTS GSQTVFNRLMQGVKGKVIPGSGLTVKLSAQTG
GMTGAEGRKVSSKFSERIRAYAFNPTMSTPRPIKNAAYATQHGWQGREGL
KPLYEMQGALIKQLDAHNVRHNAPQPDLQSKLETLDLGEHGAELLNDMKR
FRDELEQSATRSVTVLGQHQGVLKSNGEINSEFKPSPGKALVQSFNVNRS
GQDLSKSLQQAVHATPPSAESKLQSMLGHFVSAGVDMSHQKGEIPLGRQR
DPNDKTALTKSRLILDTVT IGELHELADKAKLVSDHKPDADQIKQLRQQF
DTLREKRYESNPVKHYTDMGFTHNKALEANYDAVKAFINAFKKEHHGVNL
TTRTVLESQGSAELAKKLKNTLLSLDSGESMSFSRSYGGGVSTVFVPTLS
KKVPVPVIPGAGITLDRAYNLSFSRTSGGLNVSFGRDGGVSGNIMVATGH
DVMPYMTGKKTSAGNASDWLSAKHKISPDLRIGAAVSGTLQGTLQNSLKF
KLTEDELPGFIHGLTHGTLT PAELLQKGIEHQMKQGSKLTFSVDTSANLD
LRAGINLNEDGSKPNGVTARVSAGLSASANLAAGSRERSTTSGQFGSTTS
ASNNRPTFLNGVGAGANLTAALGVAHSSTHEGKPVGIFPAFTSTNVSAAL
ALDNRTSQSISLELKRAEPVTSNDISELTSTLGKHFKDSATTKMLAALKE
LDDAKPAEQLHILQQHFSAKDVVGDERYEAVRNLKKLVIRQQAADSHSME
LGSASHSTTYNNLSRINNDGI VELLHKHFDAALPASSAKRLGEMMNNDPA
LKDIIKQLQSTPFSSASVSMELKDGLREQTEKAILDGKVGREEVGVLFQD
RNNLRVKSVSVSQSVSKSEGFNTPALLLGTSNSAAMSMERNIGTINFKYG
QDQNTPRRFTLEGGIAQANPQVASALTDLKKEGLEMKS
> Q121N8_POLSJ
MGKILLGCIADDFTGASDLANNLVRAGMRVVQTIGVPAGPLGAEVDAVVV
ALKSRTIAPAEAVAQSLQA LAWLQAQGAQQIYFKYCSTFDSTFSGETRGN
IGPVTEALMDALKTDFTIATPAFPDNQRTVFKGYLFAGDVLLNESGMQNH
PLTPMTDANLVRVLQAQTRRKVGLIDYRVVARGEAAIRARIDQLKAEGIG
VAVVDALSNDDLLRLGPALKGMPLVTAGSGVAIGLPQNFGITPSSKASEL

PPAGGLQAVVSGSCSLATNRQVLAFIKSGRPALAIEPLRIAAGVDVAAEA
LTWAAPLIQQGPVLVYSTAD SNAVKSVQGRLGVEEAGAMVERTIAAIARG
LVERGVRQLVVAGGETSGACVQALNISQLQIGPQIDPGVPWCHALSDAAA
GQGVHLTLKSGNFGSDDFFTKAFTMLQ
> Q8XZC6_RALSO
MKIGGTQSGPVVPSSDSTVEGSPAPLTHGPTSTAPTTPEPRQVSGEHGGH
LRRRVGAGAAMVSSMTRRAKLPEAVDTVRNASLTRALQTTVKGVVAPAVT
LTGSVLSTVGKAVRQASQAPASSLPLQRA LGGDLTQPDRQMEAHRKAVNY
GKHSAQHLDRIVAFLHGVLRLSAGTSTAFGVGRNAGLAAGDALGRAERGV
PQASGVITGSEAPARTMSSQSAFLLAAAGVQQWALGAVTGAAGNLAGQVV
AGPIINMLPRQFQAIDARAVVPQEIVALMNALKPGAGNELRDKAKAMQAD
IANIASDSSVWLGQIAFDSITAARMAAQGGTPLGVAGQISLGLAVSGTAG
AVIGAVIAVRQSTATIDVPKADALRAALEL APADRPAVLETHAVPLFFAK
HATMPATAAAGDIETGHIATAAQPHQQEQMSRMHAAVQQVKQMVGGLGGL
VTAPAGAIAKAFTAGPLLEPQPTVEGAAPSGQVSRATATVSNVLASGVRR
TKLMAESTAVTSLMSTGSAMLASATEGPARRAVLALGNAIGIHAAIRPWF
NALANDIPAGDNAMRAERQQHVNAQVPRPEQPEV
> Q8X3G2_ECO57
MTAWTVTSEKRHLHRIVYRVKYFNFTTLHDYN HFCDFIEFKHKNIIMNTS
QYTGSSW
> NleF_Crod
MFGMLPASSSSANLYSWMYISGKENPSTPESVSELNHNHFLSPELQEKLD
VMFAIYSCARNNDERENIYPELRDFVSSLMDKRNNVFEVINEDTDEVTGA
LRAGMTIEDRDSYIRDLFFLHSLKVKIEESRQDKEDWKCKVYDLLCPHHS
SELYGDLRAIKCLVEGCSDDFSPFDTIKVPDLTYNKGSLQIG
> Q8XZN8_RALSO
MMFKRIDSSVFSPYVRPEPSAPPLPEQGAPSGRAANSASHPYIRPEPSAP
LLPEGGFPHVPDQAGEQRAYSPSRSSAGVSSSPLGGLAAMRLDSGSAGTA
PMLRSRLPPAPSAPSGVPQVRNIEEMPAGVLQHVASFLDPRSRRALSQVS
TTMNDAARSSQTHMQAWNKAMLGQLHRYPNLQSLRLRGDITLDELKALPK
TLRHIDLGECDPGCGAKSHAAIEYLATLPLESLNVKGAAIGDRGAALLAG
NRSLKTLNVADGGISEVGARKLADHASLESLDMTGNQIDARGAQHLATSE
SIQTLRLCCCGVTDPGIQALAGNRQLKSLDVSGNHINEDALRALAANPSL
TTLDVSCNRQTPVGEPQSVEQGVSMALALAEGLVGRETPLASLKADGNAF
VDFAVEMLAFPTIRTASLSLKSNFIGPEGAQKLAENPALKSLDLTRNKIG
DAGAEALSHSRSLKTLSVLNCDVKDPGAQALARNPTLTTLDLGNLISEKQ
NPAARQQEQDEFDTTANEITENGTRALAQSPSLISLSVQGNLCEDAGVLP
LARSPRLTSLNVAYTNMTLESARELASNPVLTSLNVRWNYEHLGTAGVLE
LAKSQSLTFLDARDTCMGEAGALALEANTRIKGTPDDPNFIRDSYRG
> HopAB2 Pto DC3000|Genbank: AY074795
MAGINRAGPSGAYFVGHTDPEPVSGQAHGSGSGASSSNSPQVQPRPSNTP
PSNAPAPPPTGRERLSRSTALSRQT REWLEQGMPTAEDASVRRRPQVTAD
AATPRAEARRTPEATADASAPRRGAVAHANSIVQQLVSEGADISHTRNML
RNAMNGDAVAFSRVEQNIFRQHFPNMPMHGISRDSELAIELRGALRRAVH
QQAASAPVRSPTPTPASPAASSSGSSQRSLFGRFARLMAPNQGRSSNTAA
SQTPVDRSPPRVNQRPIRVDRAAMRNRGNDEADAALRGLVQQGVNLEHLR
TALERHVMQRLPIPLDIGSALQNVGI NPSIDLGESLVQHPLLNLNVALNR
MLGLRPSAERAPRPAVPVAPATASRRPDGTRATRLRVMPEREDYENNVAY
GVRLLNLNPGVGVRQAVAAFVTDRAERPAVVANIRAALDPIASQFSQLRT
ISKADAESEELGFKDAADHHTDDVTHCLFGGELSLSNPDQQVIGLAGNPT
DTSQPYSQEGNKDLAFMDMKKLAQFLAGKPEHPMTRETLNAENIAKYAFR
IVP
> Q8XQI9_RALSO
MPPPIRNARTTPPSFDPSAAGDDLRATPPRVAAATPTHRTAPSQLAGLPS
RPRLQPPQPDPTPTPLAPRGNTHTGSATGRLIALALANPAANRTPGRLAG
GLHLPSIDNGNPVLASLNVASGELAEIGAAALLATRLRLPSIALDGPRAR
GPAATLLAGLEHRTRALSLAQEPGGLGPGHPPLAVLPDIVAAALANSNSL
RDLLDRHPASVLANRLRGVGPAQPAADPSLGHAQRAAQILEGWAQQCQLP
PKTVDAWRPVLNQEESRPFVSLLARLNGCASFRPAHRAESLKELGGILQL
AAHNDAYRTRCFDICGGADANCHDNVDVIFGNLRLAARDPSYHGNAQLHE
VLSYHNRCMPWTLIDDFVSHRFGRGDQLERALALRVRLSDILPITTPAML
HHDIARIDNAHEREARAYISAHLGTQEHLLRNLSRSPAWRQFLEQQRPVE
FAANTLLWESALQDVMAKPAGDGAVADAPRAASTTAPGSRTEALAQAHAM

PGIGTGQAFQ HRQENATAMLAETMTRKLVMESAPPPDETGAHAALLTDPD
WLAYLQKEHPEDRAFSPADMHAAERHDLLMRLTRQEIAEARGMVQPHAGA
PTDHG
> gi|34496429|ref|NP|900644.1| probable tyrosine phosphatase
[Chromobacterium violaceum ATCC 12472]
MSTIQTGIQLGGRQLDLSRLDSLSGVNADKARIGIRKDGTLVVYTGRSYL
LHPDQTRRADQFLLKHDLLIPGQKPREFRLAQLFDRPMDALTQRNTQANE
TIAKPTQQDVDTVTRGGKPKLRWADQAAARPSGEPSRGERASLKQRRGAE
HLKLAQPRAEARPEKHDAIKTELASRLGSSDQPSGLLLQLKAQVGSSAEG
ARFLNDVGQARFRDIPTAAATQVRAPDGAPLPANRVQVGGVNVAIASQYP
KAAQLESYFGMLAANRTPVLVVLASDADMAKQGRDGKADLPDYFSQSGRY
GRVEVESK SKGSTTLEGGLEVRAYHLNVRGADHKSVSIPVLHVPNWADFG
AQGATALKALAQHVDAVADKKTAFYRDNNSSALNDPDKLLPVIHCRAGVG
RTGQLIAARELLKPGASSLESIVADMRGSRNHLMVQTSGQLSTLVDLAQQ
QGRAILQPETAAEPIYANQAQAEPIYANDAPPPPPRRPR
> Q8Y125_RALSO
MKVSSANAGVPASSADNTSARPSQTNADTTPLGRRRRAPDDAPGSPPARR
QRQDS PEDSAQTMFRRAGMTSLPPSPATSEHVPLLDNRPTLERMGVDHPL
PGRTWYETGHTTASLADRTSTASAAQVASSSRSAGPATAARPQPTRTSAG
QQATVGRLRTQVTGFLSGALGKLQALSAQNMDPELAQFRVLDVDRAIMPL
LIVAENARNPGLNLVPLHMDMAEDEEVRTQPPMAGSRHIAEFVASARPGR
YRAVIDDGSHTRAADIRKDASGTSVIVVDPLRKEKDESAYVDYADNVNME
FGEHAKCAFIPVDIQKSFFDCRILSLSLALKMHDKDDAFAAFHETLRNGG
DPSHHVSRAQQTEELGATLVLDGAPLVDARMMKHGQAASSVSRYLGNHPE
QSTVPVNKRNETLGERTTRHLVKRKVRNRADSEGRVTSGETKEITFSNSV
EQKRIALLNRAASYVNSAPPPVVMRMAKLLQDSLLDTN
> gi|12329054|emb|CAC05785.1| OspC2, probably secreted by the Mxi -Spa
secretion machinery, function unknown [Shigella flexneri]
MKIPEAVNHINVQNNIDLVDGKINPNKDTKALQKNISCVTNSSSSGISEK
HLDHCADTVKSFLRKSIAAQSYSKMFSQGTSFKSLNLSIEAPSGARSSFR
SLEHLDKVSRHYLSEIIQKTHPLSSDERHLLSIIINSDFNFRHQSNANLS
NNTLNIKSFDKIKSENIQTYKNTFSEDIEEIANHDFVFFGVEISN HQETL
PLNKTHHTVDFGANAYIIDHDSPYGYMTLTDHFDNAIPPVFYHEHQSFFL
DNFKEVVDEVSRYVHGNQGKTDVPIFNTKDMRLGIGLHLIDFIRKSKDQR
FREFCYNKNIDPVSLDRIINFVFQPEYHIPRMLSTDNFKKIRLRDISLED
AIKASNYEEINNKVTDKKMAHQALAYSLGNAKSDMALYLLSKFNFTKQDV
AEMEKMNNNMYCELYDVEYLLSEDSANYKVLEYFINNGLVDVNKRF QKAN
SGDTMLDNAMKSKDSKTIDFLLKNGAVSGKRFGR
> gi|21231556|ref|NP|637473.1| avirulence protein [Xanthomonas campestris
pv. campestris str. ATCC 33913]
MWSQPVWNPYASFDTGTASSHSQAVQGTATNYLRGPANYSKLSSEEQSLV
GAARWPDDAPGLNISNKSNTQDNKKYCKSLYKASRIAGGSIASGQINSFN
DLWQKATQWRLSRISSGDASKGDFAAERMPNTRFVTSLRRPYHSVIERAR
NHPDAETELYEGEYFKEIEVKVYRQCGAISGETIPMTTVSAAVDDNAISA
RLRNLPKDKRQQARQSMASSHPNMITHTSAEYIKTIRDHLESLYLQAIDP
SLEKHEAFELIARLHWWAASAAPDKRGSAAKAEFAARSIASAHGIEMPPF
RHGIVPDIEAMLRSESQFVADYPNFFERPPI
> Q8Y3D9_RALSO
MPLTKINPSASAD TTLPASPVPEVEASQDHPPAARAKTAELSALAKARLR
NAAGGTAFSLRAKLRRVAHQVGKDAVHYRNVQHRLNAQMKQLRPETGFQD
QLHFSDASLAEVESILRHIAVSPALLSASRSKSAVTALFTKTFLMPLLDG
GQLRLHEFRSGGRAQEDEEDPHRHRWNLKTEHLKGAYVQQVHEETHADAP
GARLYQKHQLEATPKDKTERRFTHLGEVHVRQSATKLYSEEQRPHDFPLA
ETHSVADLSRHAGM TLTLARTGKAIYADSIAYNAGTEILTTAPLGRAPDE
QAFIERLHRSIAILQLVQLQRELEAYLRGKAPASLTAGEQRHLEDAKAPN
YFETSLLPALATLDLATAAGQASDEFSPETVAYLDSALDRIRSTSLRQII
ADNDAHLQAGHFCAELSNFEQLKSLIRTTE
> A0B2L3_BURCH
MTASASRPLLGCIADDFTGATDLANMLVKSGMRTVQTIGVPADGAAADTI
VDADAIVVALKSRTIPAADA VAQSLAAYAWLRAQGCRQFFFKYCSTFDST
DAGNIGPVADALLDAAGGDFTIACPAFPENGRTIYRGHLFVGDVLLNESG
MENHPLTPMKDANLMRVLQRQTKSPVGLIRYDTIALGAAAVRARIDQLRA
EGARFAIADALSDRDLYVLGEACAGLPLVTGGSGIALGLPANFRRAEQLP

ERDNAASLPRIDGLSAVLAGSASKATNAQVAAWRATRPSFRIDPLAASRG
EPVVDQALAFARSHLPQPVLI YATATPDEVKAVQQALGVDAAGHLVESTL
AAIARGLRELGVRKFVVAGGETSGAVVQALDVKSLQIGAQIDPGVPATAT
IDAQPLGLALKSGNFGTTDFFDKALRALDGAAR
> SIPC_SALTI
MLISNVGINPAAYLNNHSVENSSQTASQSVSAKDILNSIGISSSKVSDLG
LSPTLSAPAPGVLTQTPGTITSFLKASIQNTDMNQDLNALANNVTTKANE
VVQTQLREQQAEVGKFFDISGMSSSA VALLAAANTLMLTLNQADSKLSGK
LSLVSFDAAKTTASSMMREGMNALSGSISQSALQLGITGVGAKLEYKGLQ
NERGALKHNAAKIDKLTTESHSIKNVLNGQNSVKLGAEGVDSLKSLNMKK
TGTDATKNLNDATLKSNAGTSATESLGIKNSNKQISPEHQAILSKRLESV
ESDIRLEQNTMDMTRIDARKMQMTGDLIMKNSVTVGGIAGASRQYAATQE
RSEQQISQVNNRVASTASDEARESSRK STSLIQEMLKTMESINQSKASAL
AAIAGNIRA
> hopA2' Pmp 5795|Genbank: AAP23125.
MNPIQTRFSNVEALRHSEVDVQELKAHGQIEVGGKCYDIRAAANNDLTVQ
RSDKQMTMSKFFKKAGLSGSSGSQSDQIAQVLNEKRGPAGPRLIRQGQTH
LGRMPLNIEEGQGSSATTSVQKSGLPNGRLVNSSILQWAEKAKANGNTSS
GALYQIYAKELPRVXXXXXLLPRAEHRACLAH MYKQNAKDGISIWPQFLD
GVRGVQLKHDTKEFMMNNPKAADEFYKIERSGAQFPDEAVKARLTINVKP
QFQKAMVDAAVRLTAERHDIITAKVAGPAKIGTLTDAAVFYVGGDFSSAQ
TLAKELQALLPEDAFINHTPAGMQSMGKGLCYAERTPQDRTSHGMSRASI
IESALADTSMLSLEKKLRNAFKSAGYNPDNPAFRLE
> gi|20139269|sp|Q9RBS2|POPC|RALSO PopC protein
MPRDTPQTVPGHSPFWPLFFFTHNNKREARHDLARLSLTLMPILPRLFHR
TSRTSSADTQRDARTPPNASPLHGEPGRTPRSRGELGRNLRLRSNAQTSG
TPGTPARPQIRASASRTAPSTPQHPQGTEGTRTVPNSPLHNDARVFRERA
DHTGLSAWRTEMLTRFIEHSRKHGLANDFEQVRVYDRLSRAVDHLKSVLR
MSGDSVQLKSLPVPELPDVTFEIAHLKNLETVDCDLHALPATLENLFLLE
TLSLKGAKNFKALPDAVWRLPALQELKLSETGLKSLPPVGGGSALQRLTI
EDSPLEQLPAGFADLDQLASLSLSNTKLEKLSSGIGQLPALKSLSLQDNP
KLERLPKSLGQVEELTLIGGRIHALPSASGMSSLQKLTVDNSSLAKLPAD
FGALGNLAHVSLSNTKLRDLPASIGNLFTLKTLSLQDNPKLGSLPASFGQ
LSGLQELTLNGNRIHELPSMGGASSLQTLTVDDTALAGLPADFGALRNLA
HLSLSNTQLRELPANTGNLHALKTLSLQGNQQLATLPSSLGYLSGLEELT
LKNSSVSELPPMGPGSALKTLTVENSPLTSIPADIGIQCERLTQLSLSNT
QLRALPSSIGKLSNLKGLTLKNNARLELLSESGVRKLESVRKIDLSGCVR
LTGLPSSIGKLPKLRTLDLSGCTGLSMASLPRSLVLPRDGLNVIFPEHLK
TDVGNARIQQNPRARLLEGHLERQNEAMNHAMFGDDESVGSMTSVPDNEA
GVVSMAFHAKHAYKRLERLRQEAGSSMAAPMRNNDAESMRRALAYAFRSM
SDLPTFKKLDNAARSLSYEVQEQLADLVAAPVGQKLVKAIAEGAYGAGRD
ARAIEQMLPELATRIANHPEIQQLREQAKRYPSSLPPEKLAAKLTPLIQP
LWDGTRAVAPMPGQLRQALQDLLVTAEGRQLASEIDRAAHSHRAGDEGKA
LRGLLPVLAAKIGSDPRVVRVREFVSRHTFGSPQQRAESLAQTLTPVVQE
LWAQTQAEVAKQGQTPAASPSRQR
> A3JVV6_9RHOB
MAHKMATVLGCIADDFTGATDLAGLLARSGVAVSLRIGVPDIPPTDTAAF
EVIALKSRTAPVAEAVAETKAALAWLRAAGAQRFFWKYCSTFDSTSEGNI
GPVAEGLMDELGAKQTVYCPAFPENGRSVFMGNLFVGQQPLAESPMKDHP
LTPMNDSNLMRLLDPQTSQDVGLVNRLVVEQGVAAVKSELAALANKNCAH
VVVDAVANSDLNTIA TACQDMPLMTGGSALAMPLPQLYAEQGLFDLKAAL
SKHVAPGSPTLILSGSCSEMTNKQVDAYLKTGAPSFQLDPLALATDGVEP
VLEWLSKQDLECAPLIYATATPESVKATQDRLGVKEAGEIVENALAACAI
AARNAGARRFIVAGGESSGAVTKGLNVDALDIGPEIAPGVPWTFCISSGT
RIALTLKSGNFGAETFFTDATAVLDAI
> gi|2496787|sp|P55730|Y4ZC|RHISN Putat ive cysteine protease yopT -like y4zC
MHSPISGSFTSSTQVHDPIHPANSDGFRETLANVELRTKSPSAECPDKMG
CCASKPQASDPNNPSTSSPARPSTSLFRYRTAELAQANADGICVGLTAEW
LRNLNSHPSIRMEALVPGSQRHASATVRQKEYENLKVHLRRQGAGPSEAD
FAAQNTMLQKAGLAPSGKEKVYKVGEPNFPRMLTKITADGSNHLLSLYFA
EGGAHTVATSAMDGN TTLFDPNFGEFTVQSDQIDDLFRSLANRYSNPNRQ
HLTTVTTQKMT
> Q3JLF5_BURP1

MSTDQALRPLLGCIADDFTGATDLANMLVKSGMRTVQTIGVPAAGAAVPA
DAIVVALKSRTIPAADAVAQSLAALDWLRAQGCRQFFFKYCSTFDSTDAG
NIGPVADALLDALGGERAFTIACPAFPENGRTVYRGHLFVGDALLGESGM
ENHPLTPMKDANLVRVLQRQTPSKVGLIRHDAIALGTCAV RETIDTLRRE
GVRIAIADALTDRDLYVLGEACADLPLITGGSGVALGLPSNFRLGGLLPE
RGDAAALPAIGGASAVLAGSASKATHAQVAAWRAERPALRIDPFAAARGE
PVVDQALAFARAHLPQPVLIYASAAPDEVKQVQQALGIEAAGHLVEATLA
AIARGLREMGVRKFVVAGGETSGAVVQALGVKALRIGAQIDPGVPATATT
EGSPCGTTEGSPCGATEGSPRGTPDAQPLGLALKSGNFGSV DFFEKALRA
LEGAA
> gi|21233646|ref|NP|639563.1| avirulence protein [Xanthomonas campestris pv. campestris str. ATCC 33913]
MPNASPALPRRLPARRLLAAALIATGLLGAASTASAACTAGPWTARANEA
GMPPVRYETTHFAFRWNGDGVSSADVRAAGEHLEMVWDTFINRLQFPEPA
CNSTTKTKASIHLDPGFGLSGGVTGSGNMGMW MAPGGLRDHWGLAHELTH
ALQGQTQSLRDSDYTGWIWESHANWMTHQLPEFHASDVHCSSMLVNYPHL
YLGSTRDRYCNWQFLEYLKNRYGYAIVNDLWAKAPKPGDPAQRSADPFSV
LRTNMGWTQAQLNDVFGDWALRNVNWDYTNPDGSDQGALYRARYGSNLAF
DPQRTQDWDNRDRALRMTVLDPVAGQANRYRVPFEWAPQRWGYNLVQLIP
AAGATSIGVSFEGQVQTAPAVTALPGLVTDPAS IPTPDSDWRWGVVSIDS
SGRARYSALQRGAKASVTVALRSGDRAVYLMVMGAPTNMHKIKWDQSYYA
IYRYPWSVTLSNAAAAGSQPGAPTPTPVGRRHANGGGWVANTANVASTAY
VGPFARVLAGNVLGNARIDGHATVMGGTVQGNAVLGGLTVWHPGAVIGNT
AQAQTVFMGPGAFGAVAVAGTTQVRGDLELREGTTPSQGVFYGYADTETM
RNPEFGADLRQAVPEITARPAGW
> Q4ZPS9_PSEU2
MGLCISKHSGSSYSYSDSDRWEVPVNPSNVRSASSHQTVSASDRASDKVD
ERPATFSHFQLARCGEDYTLSMVSLAAYQAERRHRGNLIKDRSQSALPWV
QVYHSETGLDYSFQIDRTTTVKVAGFNYNVPNDGETRHLYSAGTSQVNMP
VITDNMSACIAVACAAENVDAGTGERRPGAKVRVFHLLPFRREDLMPEQV
LASVRDYLRDIKEQGLTMRVALHGGNREGDFSVSTAEALKSLFADEG IPI
EFDETCANRTSETLLGAVILNDNSTQFIKHLVAL
> gi|21233603|ref|NP|639520.1| leucin rich protein [Xanthomonas campestris pv. campestris str. ATCC 33913]
MSAGGRVLQRSTQLRSYESVLLEWRDQCAANADQWREAWISANESVTGAE
LNLEVALRISAAHLARAASHNVASLDLSYLPLPRFPEQTFRFGHLQDMSI
AGAGLLELPESISDLANLRTLTLSQNPIGALPASISRLGELQELTIFSCP
NLTELPEHLAIRNTAGQREGLVKLRTLTLSRTGIRSLPSSLEYLKDLTHL
KINSSPLTELNTSIHRLPLLEEVDLRGCTRLRHYPSISGQQWSLRKLSLQ
DCSSLQTLPSNIDALRNLQELDLRGCNNLRALPPSISGLPDHCTIRAPAH
LQASLDQLRPRRPPLARPWDVAGPSTPQVAGASSSAARPSDDDPAITQAR
QKIEDTVYALFAAIDEDRNPFVQNAPYFIPEKREEGTPIVLGEVDGIQQM
LKESSREVFKNKLIGLANSLQQRRIPHPWKASDRLDTAEHKAFVNSHLGT
FNEHSPINGVSLYKAAQMWKTRELLVHNDPTNRDFLPPIPTYSLPEQATD
ASES
> gi|17549106|ref|NP|522446.1| hypothetical protein RS01656 [Ralstonia solanacearum GMI1000]
MRTAKCKASIRPNGSAFAAAGSRTAGMRACWKRSPTRRDMRCIAMPKHGL
RFPSILFRHAAQPARDATAQDNAPPSTAPSRQRQGLFRGLLPIGKRRGAD
NPPGAGTQTGSPPRVTAASPRLSRQASRAGETGSGRIDADALAGNPDQSD
AGWGIRLLRNARLNTTREAGGDATTAIQPHAGPTPEIEAVPVPIPPTPAP
APVDAGTKPARGIAVLPLNRQTPSPPSVSPTRAKASINRLPSGPKMDGIS
EPSTSLHRLNETILSTITEALGLGPLKATALAREAQIKQAGRRLLGSVIE
KPELPSAGTVKNLPFYEGLVPGLRGQLGLATSRADCVISEHTQLSKLGAP
RHLLRVQFRASIDRGEYRLASQRGSPNYEAVAEVAQRLAEAIDPQYRTDQ
RQYGIEFVGARGRKGSSAEVFIEHFARHARVKPRQIRSTPSTINDANIRN
ADVFRALSPEQMNKLARAAHVSHLSFKEETSTTRDGTVRHDLYLFFGGSE
NLRRDYGQAAASAVGRVGHVFGAAKKAAWIVAQAVEQRQRDGHEVHFESI
GGVSMGGASAQVFAAALQGSVKLVAPAAPLVLLDPQLLNDAQARHATKGG
AHDYDFGGSRGVAITLDYPAAPRKSLMGRMKGLGYQSPGLVRIKLALQDD
DSVKQLDNGEWVNRPPKPYGPPLTGYHGDLALYTKALLRFTASPLPKPAD
AVP
> A2U5B4_BACCO

MQVKLAVIADDLTGANDTGVQFAKQGFRTVVLFAGTQLQPLHLNEDVVV M
NSDSRGLEQEEAYEAVFKIAMHSRKLGVSKIFKKIDSTMRGNIGLEIDAV
MDAFQFETAFIVPAFPKSDRITIGGYHFVNGVLIENTELAQDPVCPVNES
YLPTLLQSQSKRKVELLSISDVRKGKKHLSELMNKLASEKIPKLIVVDAT
TDSELKMIVEATRNLENDFLWAGSAGIAYHLFDTGQAEEELGKAEIKDRP
PVLAVAGSVNSIIDHQIRNLKEKHHVEEIVLSPEEFFYEDRKKREIENVV
TIGQDLLKKGDLVVTTNREREAICRVKELQRRFGLSNFDVGQTIAKSIGM
IAARLIGSVPVCGVILTGGDIAGATCKVLKGDGIRVIGEVEAGIPYGKLF
GGPYDGMPIVTKAGAFGTAQALSKSLQTITQVYALDDKNKLTQ
> gi|21231996|ref|NP|637913.1| leucin rich protein [Xanthomonas campestris pv. campestris str. ATCC 33913]
MDKNLNLWDMSTFIQQYGALTADHPTHTPEDSPQTVPSPRSSSAHSPEIQ
ELRSLQETRPARLGARSQSSSSKHGLQQCSSSPSDESFRLHAELAAWCER
VETKPSLLAKLGCCAAPPVVGDHREQRREAMERIMRCLDAGQAGTQLTLR
DLNLSQLPPGLHRLAHLRDLDVADNVNLTRLPEDLSLCKHLERINADGCS
IAALPSKIGALKNLSEISLAFNELRTLPDSIGQCSSLTTIVVPGCKINKL
PASLANLTQLKKLDVAANIELSELSPHMNLDDVAVHSTQTRLGLMHRIFK
APTFDPETRQRLSYQASALRDRWAALSHHLSPQARARVDQMREGASTTLS
SQDHKASTAWKTATEKVSSWAEEGAPITLDRIFKLNQLLLPEGDDDNDPI
GGQLRKVGIQAAPSNTWTECRYPPPETLKDEMAKFSGWLEHSEQQAHARD
ALGHIEFAAQLHQRLVSLHPFDDANGRTARLAMDWALQRHGLPPAPPVGE
ASRLPASFLGGKRVSPEKVVLETLEGIATVMNQVHQ
> Q8Y2S8_RALSO
MDFPRRHSLSPLRFLTPSRSPQTERTPGASPAPGNTPRRASGPLSGLTKG
FNALFKADPEKAALRQAFSMAEAGKAEPLAGLLQSHPHLVMAVNANGTTL
LASAAKRGHLEVVRLILARPESAILINQINKRGETPLQRAVEAGRTAVVG
ALLQHAEIDPNRVDKHGQTPLHIAAGNRHADITRALVEHPRTEVNRPDRD
GNTALHLAVRKRGLDAAGELLRHPHIDPNQPNAKHHTPLTMAIAKLHVDC
VHALASHPGVQVNRPGTDGHPPVWQAVAQFVEHLESGSFARHRVRTGKEL
DCLFELARSPHIDLNVLGPGGHTPLTRLACAKPHRYVQAGRDTTFTEIQH
KQRVVDAVRAFLQGSSEGRNGFNPNARNIDGQVAVQIALRNGHDALASRL
LQDPRTDPGAAVTRLIDKPGRLSRLLNPGGPEPKTHGAGQAFLVEQLERS
IRFRNPDGTANPWISLALCEYAARFEITAEAQRRASAESRSCRPDAASYA
AQALEFSMAFQQAPIIKVAAQALIHHAPRHQAHFNLGGIDVSRSEIQAWA
AGDVPEAVLNAHIEQYVRDGRINVHADALLTRGQQLLNAMKRLTPEDQRR
SVEQSAADLRAVIGQRSDQLKQALKAEASEAALEALAAFEAFETVEAMEA
MAAEQAEDAEEAREEEEVEDAQEDEAAQARAHTEAEHQKARLKGRIDDLR
NAGKGIKHLLKMGPTKEDPDYAFHANVALADTWSYVQSRKDPELKDNLTA
ALLLRLADVGKDVPCNTGCIQRVAFASEGIDTSLHQGEPGRGAMYEEIVS
IAKAVNERYKARYGEVEAFDPAAPGTSSAPSVTARDRQAILRYTKGAEID
DDVVTDVKRDMVRADVLADLVGRRGWTRATVEELLAPILDNVEYLDEFSV
KASGSPDVHAGP
> gi|33596828|ref|NP|884471.1| putative secre ted protein [Bordetella parapertussis 12822]
MVPGNGRDIGQFAAWNLPRAQGYSACVFQLEGALMTIDLGVSLTSQAGGL
QGIDLKSMDIQTLMVYVQGRRAELLTAQMQTQAEVVQKANERMAQLNEVL
SALSRAKAEFPPNPKPGDTIPGWDNQKVSRIEVPLNDALRAAGLTGMFEA
RDGQVTAPGGRGTQVVNGTGVMAGSMTYKELESAYTTVKGMLDTASNTQQ
MDMIRLQAASNKRNEAFEVMTNTEKRRSDLNSSITNNMC
> Q8XQL0_RALSO
MSRIFRSSSTQPTQAPSTSGGTSSTQATPPQVRQRSASFQLKALARKTGR
ALGLVSAPRAEPQIKPGDKGPLGVLMHRERPSKLTRAFTGVPQPGDSQPS
SRASSRASSHHTPERTSSSSSSHSAVSASGESLPHLPVHEKGLVQFAAVA
KWTVQGEGVSDASQHNGVDERISMKKINIDNETHKKPGKLAHMDETRAGA
QGDLSVAVSQMVNLLKERMRLNGNPDEVAEAEEIREALAQHQFPESLVYE
AKHFGLRTEENRAAVVARFYEWAADDAELAEDAAQHEEHPATHEAR
> Q8XPQ6_RALSO
MPPSIRGLGTSALLQHSPGDAAAGETSPSSNTVRARTASAAFDRLQRRPS
LERTASQTPDTADIAHAAAAAIPYVRSLLPLKSDNKDYGEDDARIPPSKL
ARYHEDARDNRTILLNKIQCEIYALEATWRQSRQAPDFGALARII TSKGA
SAAGNCYEQAVLAYDFLRMRLTDANLPIDIVSLERPGDHNFVAIGQSTDG
NGQYPKQFSDWHPDAFIVDPWTRIACRAADYPRQWSEKMEKWAARGLRVD
DASPLDSSLLDALRDNNKISRRKAFPV

> hopAU1 Pph race 6 -1448A|Genbank: AY803995
MKPVQSVSATQHYNPLTNLGQEASCSHEISGITQTEGMQATAATTQTAEQ
LTTARVRDITAAAKVIPDGTTVMSR KPEDLSQLQTAYLAAKNAPTHPDLY
LKLCETFRVHDERHASEGHENICAKTMTARIDDDAIRVMAYASRGMHGGD
VNNFLKFSFNSGLPNPISSALPSMLTFGASSFIGAVHGPWSGFGSALAFA
AAKPFMAGGIQPAIVSLCDNIRGRNAPTVLAKGEVNDEIWLDAAAQTLEG
LNEDTRRALTSFDDAVQAAITSVGIRDPKFLETDEGLELFFDHLSDTHFN
CLREALEGVNNALQNTAPQTRESLRA LEEASRPNMLEQRETTLKAEFSNL
RQKEGNDWQRIPRSLRASGGTLTSGLSGLKRDEIPTRMNPALDRKSHFEG
LNALASPAVGTSYAAAISLLQSPMAGLDERNKQKFNVKAAIVFYDAFTPE
GKQRYLAGEEMDPDRDVDQAKLRGVFTSVSEYMVKSTQENLKQRQKVLGV
EISEAGHALNALPENTSSITPPEIRAKVERYKALSADMSAVTQAIREGNP
NFLTDLPDGEVKKAFDDCVNSGGMHVL ENMTKVKYAKQGELKAQWTQKVS
TDVSMFGLAGQFTPSIVNKLAAAICGGAGHIPNAVVGFLGAYSLAASFIG
AQTQFAAINAKNNFREGNPKPTSLETFMRGNLAPVMQASANRHSQHALQG
AEQAISTARQALNSSLGQNLRRRPSARTQQE#
> Q8XRI5_RALSO
MPNLPRVWRVAPAPQSTPPSPEPMGGGGTAALVGRAPRQRGPLSGLKARV
LPKSSETEFSTMFGGRKYAGKPVNALKAYPP PALSEASALQPATAREQAR
AARSQAATAALVQRTQQGLQRMRGTDNSHAAASAEPGARQPVTVPESARQ
YRQLLAQQFAATRDTGAVARLSARLEALAPPADAVGEHLRYARPTLVAEA
LAHTCGHDAAAALRVLASLENEARLDTAAFGTPDAPARASVSSATFHDAI
SEQEGPDEPDGPPPQSPSARSSASSEAFFDAMSVLEEPGEPEDAGAAWRT
AQLLSRYHAGFEALCDLMRLPQTPAHRHAAHA FLQAADALQRGAAGQAAS
PQALLQRCPPGSGTPDDSLAIKTLHAAAAQLRGEALSPEQTGALFAWRQG
FRAEGPGSDLAKVKARTAKFVSRTIRRVENHGWRSTLWQVVGKKKSPLSA
ARLGMQAAHRVSLDKEYTAYAASLRTALGMLGARYREALALSPAHADLPW
KQAEQMWQGAILQHWASLPADAPLSAYVLTHDTLRTIGEQLRHTVLRVRE
ELTISLAHSAEAGDTPDPGMLDKLRTLVELSRQ ANLNLGASNLPVTPPPQ
FIDAYPSGAVLRKWSEQVDAGAASGPLRKALDTAERIEQQDDLKLQEAAI
DAARGVIESLITTIEGGGKLRLASGTSLGVNTGTISTGLQNARQALAVPV
AIQADFHASHKRQAVVEIARGSHGRDLFVGTDKTVLAAAGFGASVGYDFK
VLQNRIRAMVGASVVPIDVEKGERVGVMYRAVRQVTQAAGPHADWDAVKY
DDAATREAMIALSNWLFQQATEHRERPPGADAVW NALAQHCSGAGADAIS
VGWVDQRKHRLRHKLRVGGGVNVRVTAPAAPVRVGVGVRLTGEITSRDTT
AMRETTGIMRAEQYSQGHGGHMTVRAGVNYAVGKRFYTSPAQDAAAAGPT
ASVPSGGRRSITQGFNGGTPLTYTKQFAEHSHTAKLKTVHLDGRLADRVC
YFDKEYKSAKEYLALIRSAQDTWIDMLSRKPGGTVEKARKEFEDYCATVE
QHTGPHIRYLLRERLRPAAAREVEGHLDLANLHRA TATGTPSGQADAPAM
PEAGAMATPPARKTSHMPRPLRNLADKVTGRSQQAEARRMDIDAIRARLA
SGQPEPGRMIRALQREAEHRQATARPPAEADAQACEAKADAVLFAEASWL
PEKLAIVENVSKRESRGVSLTVQMQSTTEASGQRELNSLKFG
> gi|12620504|gb|AAG60780.1|AF322012|85 ID185 [Bradyrhizobium japonicum]
MSELFKGNNDWRHQFPGRDEIT STIAFRVTHPLVENAGDILLEHQLQIDG
NKPLVLSHSSNPEAKARAAALGFLEVSDSMMVLDPTKHDDKWKNEGGNWQ
RAGKPPLYLAATEDNSGNAKYTESVAKPANDPYDEEYDFM
> gi|47013795|gb|AAT08443.1| outer protein J [Vibrio parahaemolyticus]
MKVNLEQNHYYEQRGGSDLREQLSSYIDLMDDAIRQGKQLPKDTAAANDI
ALMDDFIAIANQK KEGLNAHFFRSPLDMVNYVKSLIPSEDTTARFVVNMG
SGGIHCIAVDCAIKNGKCSLIGIEPVTMNSLGASMLAIRLQSVCKRELPE
TSLVIMETDMQRSQGECLMFSLFLVKKMHKECDEFQYLHDKNINRELPLT
QGLIVSVKDADSLLPPSLMKHTQSPNRLQKYLEMRPEAMNCVVNKKGETL
KTRQQRHITTIELGEKTVSYSNSIEQKRIKEAKGLLNNL
> Q8XAJ5_ECO57
MNIQPTIQSG ITSQNNQHHQTEQIPSTQIPQSELPLGCQAGFVVNIPDDI
QQHAPECGETTALLSLIKDKGLLSGLDEYIAPHLEEGSIGKKTLDMFGLF
NVTQMALEIPSSVSGISGKYGVQLNIVKPDIHPTSGNYFLQIFPLHDEIG
FNFKDLPGPLKNALSNSNISTTAVSTIASTGTSATTSTVTTEPKDPIPWF
GLTAQVVRNHGVELPIVKTENGWKLVGETPLTPDGPKANYTEEWVIRPGE
ADFKYGASPLQ ATLGLEFGAHFKWDLDNPNTKYAVLTNAAANALGALGGF
AVSRFASTDPMLSPHIGAMVGQAAGHAIQYNTPGLKPDTILWWAGATLGA
ADLNKAEFEVARFTDYPRIWWHAREGAIFPNKADIEHATGADIRAMEEGI
PVGQRHPNPEDVVIDIESNGLPHHNPSNHVDIFDIIQETRV

> Q8XT99_RALSO
MLGGNITERSIRAPADPVASPAGPDTERAGSPQAHRPPATRPAALEGLAR
PPVCASAGTSMAAVALRQAELPLRALPAAMLQTPEGVVLTKALKYGALLQ
TLVSDASVSTDKRREHAKRGVEQAKAGIEALSDLSRKGGLPSVAAVLGDL
LHHLAQAAMVGIKAYHRMLNEEWDARTERFGIRSEHATAMSAYEGLRSAL
TEKPFQGAMDELASYRAALDHFVEACKPSRPNDSMPARLERNLPAVLSGR
VRCRSARQSLDALHLALCGFSLRRIAVVMGLPDLSQRVSALAGAMKYRRD
EVETAIRRLDAVAMWYPGAEGAPPERLTVRQLAECKALVRAYRDGIDRVG
TQLCMDAAARIEAADTSPLWKTLLEVVEVIARYKGWLDELCDSVRGGDPE
PAAAPGTSGQAPVSAPEIASSMPDPADALAVSDTVDPADATDATDATEAT
EATEATEATEATVRTEDRGSPAPVRPLAAAPRPPDTRTAVQKQADGLLKR
CPVDSETAARFGGDAVAIADVFGKDTRDIERLMGDRKTSAAVVATSMRQE
VDGWFERRERLEQARSGLCAEDPRTGHLTDRLRALDIIDRYVTTWEADAA
KCALFPKAKHLERLLEMGEIEHVDAPVPLRSGKDKRNRDRVFEIRIQPRR
LSSPDGIEDGDRALPLFVHLHLSRPVDAGKLHTLSYGDFNAVHLKLAAQK
GQGRNWEKMMHAMGYRDAKVERAMVGDALLRRLFALAGRDDASASAVAGA
PGAH
> gi|12025045|gb|AAG45728.1|AF229440|1 y4yB [Sinorhizob ium fredii]
MEYCLLSPAQLIPTEEVNLDSVDALQAQILQAGSWTAPITAEKDALFVMD
GHHRLTVAHRLRLTTVPVVLLDYDSVRVESWRPGENITPAEIFAMARSGR
KFPYKTTRHIFAHGLPICDVPLELLSSPAPMDMAPAFSAGAL
> YPCD1.26c yopM secreted effector protein 16336:17565 reverse MW:46203
MFINPRNVSNTFLQEPLRHSSNLTEMP VEAENVKSKTEYYNAWSEWERNA
PPGNGEQREMAVSRLRDCLDRQAHELELNNLGLSSLPELPPHLESLVASC
NSLTELPELPQSLKSLLVDNNNLKALSDLPPLLEYLGVSNNQLEKLPELQ
NSSFLKIIDVDNNSLKKLPDLPPSLEFIAAGNNQLEELPELQNLPFLTAI
YADNNSLKKLPDLPLSLESIVAGNNILEELPELQNLPFLTTIYADNNLLK
TLPDLPPSLEALNVRDNYLTDLPELPQS LTFLDVSENIFSGLSELPPNLY
YLNASSNEIRSLCDLPPSLEELNVSNNKLIELPALPPRLERLIASFNHLA
EVPELPQNLKQLHVEYNPLREFPDIPESVEDLRMNSERVVDPYEFAHETT
DKLEDDVFE
> hopJ1 Pph race 6 -1448A|Genbank: AAZ37567
MMLARWFTACRTASGKLNTRKKQTPNNRRRSPRNPNMTDLNSLRTSLRSG
EHVFADTLAFIAEGYDYQPQPFRNGAV DNAAGQNEGSCKTLGLALLEGLS
DEEALLAFGEHYRSVQATPEGSDHGNIRALIEYGLAGVKFEGEPLKRKA
> Q8XQA2_RALSO
MKVNPPASPGASVSSPSAVQEAAGTQPATEPSASRAHAAGSPLASLAELS
QTRRAGVKRPAGDDGAGSSSEPPRKMPRRSVKFAMSFRDGAPRRPALARH
SATDAEQADINRQIETNQRAFLVQKLGAKQPVVRLLQSELGAEIARHLPA
QDQHRLDATLARSP EARYEHARLLPPQMGGAYLNRDEEALALALAETDSH
GAPLSADARLDRAGWLLLAGTRAAALPDVQLIGRMRRAVRQARVTLFQTF
DRAGPAAQAEALSRLIGACAYAGSQADALRWTREALGEQRFYSLPPGERA
DALAELAEHLDNAEAMEWDRLDDFTDLAMRAEHERTLARLHTNGLASTLP
MMLDAARRIGPMPSDAAELLMQSVVNMARRSMPAPATQQVMLDLFSILLP
HLQGNARADGGARLM AMLPELPAIGDQLRLFNGILRRPPGTPPSENRLSH
LDVAGQYALTQAVFSQLANMGEHVMREEAMKLLDNQLAPGRLGRFPGAAL
ESLLTDAARLPVPASSRPQLLSIVERALEALPGGRIARPLRKLLERAIQP
PAWHLDAREAAAWHDISPRLMQAWHHLLPRLPAVQCADALAFAVTRVPGL
LPLALGQLAAVTVDEHRAMLRGIEDPLRTAFADTIAHTLVGSPGLLYDAI
RHALSIAPVERRTTVV ASLAATLAWTATAVSSPLRDNVALLMEAIGRQCL
GAVPREQLESTLAAFFRRGELPSLPSLAAQHLRSLNLADEARVLAGLTNA
VASLDPPTRALVFSFVMERLAAQAAGQAGAFARIAAGLPPDVARTWAGQL
QTELDAELGLRLASGPRATVDPPHLERLAAEAGRALEFRLSSQPGPSEAR
GDAWNRYGTAVAMIEALHAHHPGPGPYA
> Q8XRH2_RALSO
MSSPAPLSADALIDRIRTDIRSTG DAPDVAARHEHFYLVMQALRSDILAL
SNREPDDERVVRCIRVFHEEVAAFKEAHAIARLPYSPAVDRRYPFRDAAG
EPVYVATLAPTGQPAQGSRRYGAEAVWPYLDAEAAPEGLGALYLGRLHCR
TMMAADLRDPRESALVGERGVFAARRIERGECLGIYGGRLMTPATYYTCL
DDAFVLSTTAEGIESAVDGENILAMANTVFAYEGEHAVSQAADGYNMEAA
VFQATTRCGRRFAIRAFFATEAVPA GDELRWNYRYAPALIRQRFGGLPA
> Q5GYE5_XANOR
MQIKTASHSPNAHPHDPVAGDGSSPADRSIEIEAGHGSGTVRPADGTQAR

ACPSPASETTVCLADLLSISSQRPSRPEVIPAIVQAGGDEGVPDVLPQPA
RSGASQPRLANPGEARDVESRLLMRDCSSRIAAFLGNLSVVQELRQFDDS
AFVGKLRNELVTEPGCAEPKTLLAAIGEKVAELWSHLDKKASTPEEAEFL
AEARRYLEPVLT SLETDIHTLQHDASAAKRIYQGALTLLLYPLPLATLFT
QKTGTYAAFNIASYTYTAIQLVSLMRRPTTDAKLFMKHAINRHSLVFFIS
LIYAVPTFYAKASPLQRNAGFTAGAAVAQGTMMFGLRLGQNLMDSMRLRF
NGAFNRRRDLPDGFRDAIEGVVGDLRTGLSNVNRSVGEFQQDRRITPHMD
RQLTFFKQDLSRVVTGLERLLATGTRKETPVAARDDAPGSLEAVRRTLEA
SFANNPDLKGKLA LATVAFAVLGSNIALMRNNGLALPDFIADAVVSSTFL
LSEALSPHVTHAGMNDSVSDTVGGMTIGLPFSVAAVMSSYMDDPRANPSG
FIAGTVGYTAAYLLFGRVAGDVLSKGLMATSGALGWGQQQAIRLGRSAMA
LGFELVAGHSSPAAADVHDVAGVEMAGVPHEPLFFSPSQRASAERTLVGT
LEQIGDEWHDARDEWEGESEATAGRDAPWVDAPAELPMQPAPQSE
> hopF1 Pph 1449A r ace5 & 7|Genbank: AF231452
MGNICNSGGVSRTYSPPTSPVYGSGVSSPSRFVGQYTLTSIHQLSSEERE
NFLDAHDPMRVYDLNSETSVYRTTQREYVRNGYATGNPNSGAIIALHEEL
QESPYAQHIGARPDQADAYRPRTAHVSSLNTPSLNVMAGQGALSALRGYA
GSDHVTTEMRLGDFLDQGGKVYSDTSAMSAGGDSVEALIVTLPKGRKVPV
NILD
> gi|55977841|sp|Q05 608|YPKA|YERPS Protein kinase ypkA precursor (Protein kinase A) (Targeted effector protein kinase)
MKSVKIMGTMPPSISLAKAHERISQHWQNPVGELNIGGKRYRIIDNQVLR
LNPHSGFSLFREGVGKIFSGKMFNFSIARNLTDTLHAAQKTTSQELRSDI
PNALSNLFGAKPQTELPLGWKGEPLSGAPDLEGMRVAETDKFAEGESHIS
IIETKDKQRLVAKIERSIAEGHLFAELEAYKHIYKTAGKHPNLANVHGMA
VVPYGNRKEEALLMDEVDGWRCSDTLRTLADSWKQGKINSEAYWGTIKFI
AHRLLDVTNHLAKAGVVHNDIKPGNVVFDRASGEPVVIDLGLHSRSGEQP
KGFTESFKAPELGVGNLGASEKSDVFLVVSTLLHCIEGFEKNPEIKPNQG
LRFITSEPAHVMDENGYPIHRPGIAGVETAYTRFITDILGVSADSRPDSN
EARLHEFLSDGTIDEESAKQILKDTLTGEMSPLSTDVRRITPKKLRELSD
LLRTHLSSAATKQLDMGGVLSDLDTMLVALDKAEREGGVDKDQLKSFNSL
ILKTYRVIEDYVKGREGDTKNSSTEVSPYHRSNFMLSIVEPSLQRIQKHL
DQTHSFSDIGSLVRAHKHLETLLEVLVTLSQQGQPVSSETYGFLNRLAEA
KITLSQQLNTLQQQQESAKAQLSILINRSGSWADVARQSLQRFDSTRPVV
KFGTEQYTAIHRQMMAAHAAITLQEVSEFTDDMRNFTVDSIPLLIQLGRS
SLMDEHLVEQREKLRELTTIAERLNRLEREWM
> Q7DB68_ECO57
MEAANLSPSGAVMPLATSLSGNNSVDEKTGVIKPENGTNRTVRVIAGLAL
TTTALAALGTGIAAACSETSSTEYLALGITSGVLGTLTAVGGALAMKYA
> ECs4550 ECs4550 EspF 4589257:4590003 reverse MW:25252
MLNGISNAASTLGRQLVGIASRVSSAGGTGFSVAPQAVRLTPVKVHSPFS
PGSSNVNARTIFNVSSQVTSFTPSRPAPPPPTSGQASGASRPLPPIAQAL
KEHLAAYEKSKGPEALGFKPARQAPPPPTSGQASGASRPLPPIAQALKEH
LAAYEKSKGPEALGFKPARQAPPPPTSGQASGASRPLPPIAQALKEHLAA
YEKSKGPEALGFKPARQAPPPPTGPSGLPPLAQALKDHLAAYEQSKKG
> Q8X394_ECO57
MDFSWEGRELEFLIYFNCINILNGLHRVNVSSTPYSLAYNTCCNLHLSAM
KKHHLSLYEILDLPSANLSFQSTFKYCIYLPTRSYFRKLNMNDNIPTARN
HKQSTCITEKTCLYF
> gi|16764579|ref|NP|460194.1| secreted effector protein [Salmonella typhimurium LT2]
MPITIGNGFLKSEILTNSPRNTKEAWWKVLWEKIKDFFFSTGKAKA DRCL
HEMLFAERAPTRERLTEIFFELKELACASQRDRFQVHNPHENDATIILRI
MDQNEENELLRITQNTDTFSCEVMGNLYFLMKDRPDILKSHPQMTAMIKR
RYSEIVDYPLPSTLCLNPAGAPILSVPLDNIEGYLYTELRKGHLDGWKAQ
EKATYLAAKIQSGIEKTTRILHHANISESTQQNAFLETMAMCGLKQLEIP
PPHTHIPIEKMVKEVLLADKTFQAFLVTDPSTSQSMLAEIVEAISDQ VFH
AIFRIDPQAIQKMAEEQLTTLHVRSEQQSGCLCCFL
> hopAW1 Pph race 6 -1448A|Genbank: AY803997
MRVRVSNTLQPAVEHTTQATIGGCSSSTASRIKEIPFKQADELARVGDQR
AACVVLTAAWLDRVHHHSQPAEARIDHMRHRATLEQVAERQQTYRNHEIN
NPRTPYEILFSPTFRDYSLRLSNARILDIMSDEEQAMGSMANTLRDPNSS
HVLVIVRMNGDNHAIATH CTGNKLHVFDPNHGEYSFKADTGTVEESMRDI

IQAYSSRFPVPEIHILPVRS#
> gi|28871468|ref|NP|794087.1| type III effector HopPtoE [Pseudomonas syringae pv. tomato str. DC3000]
MNRVSGSSSATWQAVNDLVEQVSERTTLSTTGYQTAMGRLNKPEKSDADA
LMTMRRAQQYTDSAKRTYISETLMNLADLQQRKIYRTNSGNLRGAIE MTP
TQLTDCVQKCREEGFSNCDIQALEIGLHLRHKLGISDFTIYSNRKLSHNY
VVIHPSNAFPKGAIVDSWTGQGVVELDFKTRLKFKHREENYAVNANMHEW
IERYGQAHVID
> Q8X782_ECO57
MLPTSQLRPTGTFCSYSAETSADIKSEITPIQIEEARASGRLYIKDCDIE
YLPQLPNEITSVTIENCNNLTTLTGLPVNTQNLSVINCEKLQITDMPSTV
KNLHIELTDSPFIHFISEGIE CLTVCHCYISGVPESVRYLEIKGSATDSI
KNVPNGLSSLSINSYNPENQARIDNLISPSLKTLSLTGCSNIILPEKLPE
SVTSVTIHAEQKTTWNIGVEGMPDGLDLDLQNVLLSPDVVKAKNITFQGN
ALDVALHFREGDIVYGLSSPREKLVNSIKLVNDFSKKDIITQNTLTNAVW
DPRTPRKYKQDPLIKRALNEHERGIKFKQHLKNHNNYNVTMADLSVYNRD
KLWAKTSKAGLEFQTLTRNKTV IFCADELVNSLKLIANKSEGYGQSITAS
ELRWIYRNKDNNQIMKNIKFYLHGKEIPAERILDTPEWKDYRPKYSGSTY
KYS
> A0TW66_9BURK
MDRRRRHSPGSAQVRRVRSTSRRALGCRIEGQAPARQQPEGAHARRHEVH
LPAPAAVNALAPHAARLLIVADDLSGAADCAVAGLRHGLTATVVLDAAHA
AAPPHGVDILSIDVDSRRLPKDVAARRTVDAVASLSSAGTRLYKKVDSTL
RGNFAAEVAALTSRAGMAIVCPAYPASGRTTIDGRQWVRGVPVEASEYWR
NEHIPGTADLVALLSAEDLRVAYMAIGTVRGNANALTAHLRGLQSDRMQA
VVCDAESDDDLQRIARASAALDGVFWVGSAGLAPGVIEALGPPHGASGPT
AGGPHSKGPVLTVVGSMSSISHAQLDCLKANAGTHLVALEVSVCALDDPR
SDVTTVVSDALRGGRHVVVSLSQARRGDVDDGLLFSTRLAALLAPAVRHA
GAIIATGGETARALLTAAGASALRVVDEIEDGMPLLECRLHGHALPVVTK
AGGFGQPESIYHAWRRLANTGEPADTAQPNQ
> SOPE_SALEE
MTKITLSLQNFRIQKQETTPLKEKSTEKTSLAKAILAVKNHFNKLSSNLS
ERFISHKNTESSATHSHRGSASADRAVLTNKVVKEFMLQTLNDMDIRGNA
SKDPAYASQTREAILSAVYSKNKDQCCKLLISKGINIAPFLKEIGEAAQN
AGLPGATKNDVF TPSGAGANPFITPLVSSANCKYPHMFINQHQQASFKIY
AEKIIMTEVAPLFNECVMPTPQQFHLILENIANKYIQNTP
> gi|17549774|ref|NP|523114.1| SECRETED PROTEIN POPF1 [Ralstonia solanacearum GMI1000]
MSTNISSAASPTLPLAGPGVNGPAEGKSDMPGSLFFQFDHSTGSTRPDLP
TDLFFSFGDSISRAVQDASNQSPEQAPAADPAPASPDG QSCQPSQPATPP
VGSDVTWNGGTLNDTQLQIIGILNLHKDKGDISWDKLQDKINDPDTPPDL
KWALQALSQDFNLFQAIGSQGDGRFGGKIKGKDLAEFAKSHSQVLTWNSG
TLNDSQLEIMSILARHKDKMPVDWSSIQDKINDPSTPSDLKAALQALAND
PALFFAIGSQGDGNCKGKIKAGDVSKFADNHPQVEEYNRKKAEGYVKNYI
PSDAKPGDKPSAMTQNDALRELYRYSDYLPKKLDMEAFQ RIVDGDSDVKK
APPQVIAAAEYFLQNRNEWASLNKMDDPDKRVGKSDFLQRAASAVHLSKE
DLQTVSTINSNLDVFFKDGQKITRDRLAAMSQDESLSPAVRNAAKQLLQD
PLLYGLINNANSGYKTKNGFFSFGGPTVDSGVIGKKDFEKFMSSMTDANK
TVQARKTHPANSEASKSAVADMGMGMEDQPDIKAVKKSGGALKKAMDKIL
TIYSKVMDIASQVVGALGVIPGLGEIADALSMGMAAGASA AKVLSTLLNG
GSLKKALAEAGINLASAALGAVAGPEARVALKNGLTKMLVEKVANTGIDL
AVDKAKSFVDGYLQDLKGRLQATAANAANTVNTSVNWVSDKTKDFLENPV
QNLTPRVNIPGITPYQPGYPMVAAAA
> Q8X395_ECO57
MLQLSSNIGWKKGAENALKNKIHSHSFVVNPDEFSCDTQFLKCPITLCVP
EKGVFVKNALNSNICTLYDKSAFMNLTREHLPHPLSREKIVKEMIIERNM
CYFDTISQHFIIMDADQQKQHCK
> hopAA1' Pph race 6-1448A|Genbank: CP000058
LFDRHFAALLSRNYSFRTSPPQRFAVQTPHINRSAPQPPGIEMESFRTAS
DASLASSSVRSVSSGAQNNLHAITDYLKDHVFAAHKLPLTDSLEDHAAIH
AHNEQIDALIDARARRLSEQGEPRASIGETFAKAEKFDRLATTASSALRA
TPFAAASVLQYMQPAINKGDWLPAPLKPLTPFI SGALSGAMDQVGTKMMD
RATGDLRYLSTSPDRHPXXXXXDAMATSVKRHSPGLGRQVVDMGIAVQTY

SARNAVRTVLAPALASRPAVQSAVDISVSTAGGLAANAGFGNRMLSVQSR
DHLRGGAFVLGLKDKEPKADLNEETDWLDAYNAIKSASYSGAALNAGKRM
AGLPLDIATDGLKAVRSLVSATSLVQNGVALAGGFAGVGKLQEMATKNIT
HPATSAAVSQLTNLAGSAGVFAGWTTAALATDPA VKKAESFIQDTVKSTA
SSTTGYVADQTVKLAKTVKDKGGEALVNTGASLRNTVNNLRHRPAREADI
EEGGIAASPSETPFQPGRS
> Q4ZX80_PSEU2
MQAPTVNRNTGNVIAPEATARADNPSNIRQQTEQPTQRSSHSLGSMGKRL
LKSVGKVFQKSRAPRQSAARPPSTQPPSSSPPRSSREAGRNTSRDTRLPD
NPPPRNDPPPKSILRTRANTSGTTKHEIEQGSTRADTAPRNNRRVTFNLP
QDQLTRSASEQLAAEPMTDEEAVASAMRERRNPGGRLQGPDGTRQSMQPV
DPNTASSSGTQADDPDGPLARRNTGLESSTGGSSELRSEQLIRDTRPQAT
IRLSADGKPDFSSFKTPGMAALLNDILAKPGQTYLAQQSEQGVQDHQLLQ
ANGHLLHLAQGDSSLAVIRSSEPTLPVAGGKPPPVNMQREENHIHLDTLD
GRRSQELPGKAHIAHLTDVHQTTNGDRLRVHEDRLYQFEPLAARWKPVDE
IEDIAFNRLITGGNGSVYAKSDDVVVDLSSPFMPHADFNDLKSFSVAPDD
TAALLSGSDVQTVLLTDMSPVIGGLTPKKTKTLELDGGQAQAAEVALSND
RLFIADTQGRLYSTDRSAFESNEPTLRLMPERAGYRLDDQPMGGHNSVSG
FISGDDGRVHALIRNRQGEVHSHALDEQGAKLESGWNLTNALVLDNTRGL
TVMPAPVGADRLHLDRAGLVGLNAGRIQRWDATPQCWKDAGIKDVDRLQR
GADSNAYVLKGGKLLRLDVAPKHPNVAFDHNTALAQIARSTKVAMGKEIA
GLENRVVTAFAMVSDKRFVALDDQNRLTAHSKDHKPIDLDTSAIDGDIKD
LALDEKHNLYTLSSTGKLYCMPREAWQATRFGGQLAAKWTPLAAPDGQPV
KALYSNDDNRLSVQVEDAAGQGLMQLKEGQWQAFEQRPVEKNGLNDVYNR
ITRAHKTWRIPGTGLTANLDINAFGRSGMEKSNRPTASEAIRANVYKHTL
EPPRWMKNVGNNIQHRYHGREGLKELYKKELIAFKQLELVHEAAGAPPTP
GNDLKARIARLQLGPEGAELAKELEVFRDELEKHARTALENIGKDYGKLK
NLKQNDGVLNQHSALAKPSKRTQLGKKLAELGSILNFKSSGHDFVKELDD
ALTRIAPSAENPTARTLKLLKDNGVKLSHTKTDIGLGQRRDANEDHGLSK
ARLALDLVTLNDLGALIGKVELLARSSDLPRLQAELTSLRDVTYGENPVK
QVTDMGFTDNAALEGAYDAVKAFSNALKKSDHAVSVNMRAATGSQNQSEL
ADTLKAMLKNLEHGDDEIGVQRSYGLNLTSPWVVLADKSTGPWPNAGATG
NRNYLFSAERCEGGVTLYLTREAAGNVNGGVGGGKDYWPGFFDENHPARS
VDIGNNRKMTPNFRLGADVTATAAASQRAGVIFNVADDEIDGFVDDLFEG
KLNPLQLLNKVKDHETYEARRFNFDITTGFSADMRVGFGLSEADSAPLSA
VARLGVAANVTVNLLSYTDYSLTQKNDKTELRDGGKNRPRFFNSLIFGGQ
ARGQISGTHSNSTATPTSAAGPTPATQSAANNLGVAANVTIDDKTVKRVK
FRFNVATPMTTESLDKLSKSLGAAFKDKATTARLAELQNPLNALYVDMNP
EQAIQARLDGLKALFAERPSQNDTQYKALRDLKRAGVQHEASINNHSVLD
NARFETSKTSLSGLSKESILTRIMGSLRDASAPGNAARVAEYMRQDPTLS
AMVKQLENSQGTLARIRLEPKDSLIDEIDEGSRRGTLTQTELSGLLENRN
NMRIKRLVVFHTPRQTENFTSPTPLISYNSGASLSVNKPLGRINFIYGED
QDKPIGYTFDGELSRPSASLKEAAGELKLSGFEVKS
> Q8XT19_RALSO
MKFNRTHRNLPPAHVGHTTRGHAPAPAGPQRSRAPRARPQDLPGSSRGAA
RTARIPSSAPGEALSRSLNYCEYNLAGERRTRLENERSQQQNELGHKSGI
DFPARCIDMARNNVLAESNGHGWALVFLFPGGDSDTNLTLLGDYLAKLGD
CEHGRVEPALSGRIRACAELCAHYLLHTSWFEGAPAASYAYPASKFSKFP
DSRPCMEALDWIARQALQARNLQPMGDRKASQFVSGLAKNTRSDACRDAV
QRIVHWLLENSAVRARLDAIQVPTLLNALSKWATHDGATSLALQVADLLA
SKPRLREALDRQGIGNCLNALSKWSTEPWAENVVMQLAGRLTAEPDLRLS
LRGQELVNSLNALSKWAHCEPAKQAILQLADRLATEPQLRRQLNASLLSS
GLNALSKLSGERQAKEAALQLAHSIAIEPRLLQKIDAQAVANALNALSKW
PADERAIRAARLLADCLVSNPGLRRALKPQEFTSTLNALSKWQHEDTIKQ
AALLLAERITTEPALRAAMRERDIANAMNALCKWPGADVSKQAVLLLAKR
LEAEPQLRNAMNDQHVSNTLGALSKWPDEEQASRTALLLVKRLVDEPRLR
QSLEAQGVANILNALSKWPQHDLAKQGALLLADRVASEPSLLTAMDTQET
INTLSALSKWPGEPRATLAAQRLTAASTGRWATLDARNLSNALIALSRWP
DESWARQAAMPLAERIATEPLAWNSADPQSVSNALSALSKWPDENQTRLA
TRELAGRILADEKLRMAFDAQALAHSLNALAKWPNEPEAMRALWQLSTLP
GRASHPWRTFNLVDMAQISNAFARLLQDDGEDLERARGILQHLAVHLELH
PECFERSSVGHIGVLFKSFANLRILPSLRPLSRPVLNRVMTLCRETQLRD
EPLETIGNLCLGLLPLARSPELVRHRASALRAFDALQPIVARKIDSYLRQ

GERATTGAALLVTDGQEACGTRGPALTFYQILKAYTLVARQWKRRYIQDA
RQTVHDRQAALQQWVNDTLARTRSVIEADLQEKSWNLIAQIEAGDDVLNA
LDLRIGKDLEHITQRHPPTRFDLAGAHTRMGTAPGRVRPVTAGVGDTRHV
TVDLQGRELKVDDGIHPPYSLYARLTGQPLVEVRLPGKLSTFMLARTFQY
QGEPWRFDLFGGSRLTRGRSNRVVSILAGDRPLPSVLPAIRYADSAPGSA
FMALTHKLAPQREDWSRMQRALLEMVPHDHVVEGTMRIGWFKDVSGPQHP
FKLTGPGGQRIALCPNDGCGFLKWEVAMQIPAVREQIEAWHAVRRGRATD
AQQQRIADQAPGFNTMPPQALMHFPRDEAVLVEAHEALQHRLDKLRAEVQ
GRGAPASKLEAEAGALSPDAVDLLTLYQMATSGGYQGRRIRAVPSADDKL
YLPTIPMNGFARPAGDLLVGKPPYDKENLLPIGPERVGTAVHGDATGRFL
DQCFAIQYSYTGFDDGSGLAADMLHSKGMLIIPPLGYWSAAHGHMDLACS
TEDLKVLSRWKQGRDRDSVPARMLSTGSLRVKDILLPGRLGALPIPELRK
RNMDTDGDDAFVYAGYPKLAAHIRRVMDDRSDRRGTEHSFKPPKTANPAF
DSQGQYQAGRAREILAEQRGGQLVGVASNAATRFLSQPDELREAMATSMM
FGTYDGIERRLRNGLRALLEGREPAPALQELQALAHQAIARAHLPEAHAV
AVLLHTLTTQLGAAEAQPVPQLAADVTQRFSPLAEAWSAAADTPARIHAI
LDHYPVCRLSHEQFPKGQPGYVKGQPELTMRNLFTLAVKVGTDALKSDTG
TELFTTLIQKCEAVERSFPGRVRYVPHTKQTAREFRNERFDPERAVATLE
RIPTLAAGVMQDAVSSLQQAGLLVARPAPAERLRTVSPEAMDRAAIVLNE
RAHTASAQITPLLQTNLRAWIGADLGADAARLAGLEHAVKSAGSLKDKLG
YMIAAKQLPDLQDALSRVNDALRYSIVLPPDTFVAASRRILAGLEKHGHA
MTARTNHFSQPGTAFGALSVTLQAPSGDFLWEIQFHTEQTFELKARHHNL
YKQAQQERHQGASSDAIRALLRPAWQDFRAVPVPAGCEEIDDWQQESVDT
APPSHPVREVQSAQPIAAYLRPLVRELGTQAHRMEARVSPKLQPLVQKHG
GKLREDKPGNWRQFIFKKERSIARKIALRQRANEHLTPEHAAARVRDTLR
YEVILPAEGFGKAVDTILKTLGRHGLKAMRLKNAFMRPDTTYAGLNVNLR
LADASAPGDFEIQFHTAHSLSTKLKMHRDYEKVRELPPADARIDGDEAGL
DFNAERERRLKKMRDAAALVERPQGIETLIPFDLYQDA
> gi|29840502|ref|NP|829608 .1| hypothetical protein CCA00745 [Chlamydophila caviae GPIC]
MKVKQITALICSLVLGFQISGSAKTLVQKNACSDLDFLEHLLDVKYAPKE
WKHKLFHWDLKDATDQARLKLCIEENPSTSYCQGVLAEYISDLKDFHAGI
TFFRTENSHLPYTVKLSNSRRCFIVDVHTYNSEISVGDEILEMDGMPIME
VIESIRTGRGALSDYAAAARTLFSRSAALGHQIPMGVATLK IRRPSGLTR
TVKAKWRHTPEYIQDLSLISPLVKDPIIQMRSSRACPLLSSASENCLFTN
EMVPYFWKELRQQYKRGLSSDYNIGSKRGFLPDFGHVTWKAKSGPYHAYV
FTCTDNHGQSHSIGFLRISTYSWTDMEDRTAMNMESPWDDFSEIISVLQE
KTEALIIDQTNNPGGSVFYLYALISRLTDRPLETPKHRMILTQSEVQSAV
QWLNLLEGVETDEQARNALGEDMEGYPIDMNAAGYLQTFSNT VLKCWANG
DINLSTPMPLLGFAKVHPHPEHRYTRPICVLINQEDFSCGDLFPAIMKDS
GRALIVGTATAGAGGFVFNVEFPNRTGIKSCSLTGSLAVRPDGSYIENLG
VSPHIFLDFTDTDVQTGKYSDYISTVKSLVLDLIEREADNKAS
> gi|58579718|ref|YP|198734.1| Hpa1 [Xanthomonas oryzae pv. oryzae KACC10331]
MNSLNTQFGGSTSNLQVGPSQDTTFGSNQGGNQGISEKQLDQLLCQLISA
LLQSSKNAEEGKGQGGDNGGGQGGNSQQAGQQNGPSPFTQMLMHIVGEIL
QAQNGGGAGGGGFGGGFGGDFSGDLGLGTNLSSDSASMQ
> Q28MT1_JANSC
MTVPRLAWYGDDFTGSAAVMDELAQAGLTAVLFTRMPDDATATRFAGYDA
VGLAGTARTRGPAWMRRELPGVFDWMERTGADHLHYKICSTFDSSAELGS
IGLAAEIGLAQLGGWAPCLTAAPSMGRWQCYGTLFARGPDGAAHRLDRHP
VMRQHPSTPMAEADLARHLAEQTDLPIGLLTLDQLAGGVPERDRDGIVLL
DAQREADMVLAGAILADNGAGLLLGSQGVEMALIAHWRAAGRLGSEQPRT
PLRPARLAVVSGSVSEGTAAQITAAKAAGFATFALDTASLLDGAVPDLTK
ATAALRQGASILCHTALGPQDPRLSATRDRQRSLGLSPEDTAERIGTALG
QVLARLAPTAERLVVAGGDSSGFVTTALGVDALTALAPVDPGAPLMRVHG
GAADGAELVLKGGQMGARDLFVRLRNGDR
> gi|15618718|ref|NP|225004.1| hypothetical protein CPn0809 [Chlamydophila pneumoniae CWL029]
MSISSSSGPDNQKNIMSQVLTSTPQGVPQQDKLSGNETKQIQQTRQGKNT
EMESDATIAGASGKDKTSSTTKTETAPQQGVAAGKESSESQKAGADTGVS
GAAATTASNTATKIAMQTSIEEASKSMESTLESLQSLSAAQMKEVEAVVV
AALSGKSSGSAKLETPELPKPGVTPRSEVIEIGLALAKAIQTLGEATKSA

LSNYASTQAQADQTNKLGLEKQAIKIDKEREEYQEMKAAEQKSKDLEGTM
DTVNTVMIAVSVAITVISIVAAIFTCGAGLAGLAAGAAVGAAAAGGAAGA
AAATTVATQITVQAVVQAVKQAVITAVRQAITAAIKAAVKSGIKAFIKTL
VKAIAKAISKGISKVFAKGTQMIAKNFPKLSKVISSLTSKWVTVGVGVVV
AAPALGKGIMQMQLSEMQQNVAQFQKEVGKLQAAADMISMFTQFWQQASK
IASKQTGESNEMTQKATKLGAQILKAYAAISGAIAGAHKTNNF
> Q8XYB9_RALSO
MPSRMGYSRISSGLNASRGASPAPQPDTPPQTPPPDNRRRTRMDSPLEGL
HHARRSAVPGAGSSGGMPQTARSLPRRMESRVSEPVEREVPERAT SEPRP
EDASTSDPMRSAGRERSCISPPGSRRAGADAGPTPAQLAEYERWLDAAAL
RQLDAQREEQCWQLQALVQDAWNRKVDLQLCAQLTSDNILARRNGCGQAL
VFLDGAVSAEENARRVGDYLWVLSQSSAQAGRHGQRQLERCVDLAASYLF
KTDWFDGVPCAQLSNLGNLLSKYPTRPAAMEAVAWIAGQVLAPVRLPRLN
GKQLALLANAFSKNTDSLRCERAVARIASQGLDHCTEALATQSISL LFNA
MSKWPDNAVCQTAAERLAAWLAEEAGTLRAMTAQEVASTLNALSKWPDSD
ACRQAVERLADRLAKESRLRQALSAQQVANALNAMSKWPKAQACRTAALQ
LVGRVADDERILLTLNAQGVALALNALCKWPDADACGQAVERLAERLARE
RGLLQAMSAQNVANALNALSKWPNNKACRLAAVLLAERVAKESRLRQAMS
AQQVANAVGAVGKWPDEAFCKAAAECLAVRLADEADLRWALSAQQFV STL
SGLSKWPKSNDCGRAAQCLAVRLAQDAVLRQALDALGIASTLSALGKWPE
ASDCRIAAGYLAARLAQDGGLRQALDVQGIANALNGLSKWPNADDCRTVA
KHLAARLAEDAALRRALDAQGVANALNALSKWSDVEGCRVAAEHLAERLA
TDADLRQALDAQGVASTLNALSKWPDTAVCKAAAEHLAARLADEAGIRRA
LSPQGMANALNALSKWPTAANCCLAAELLAGQLLRDPALWQAMNAQEF AS
TLSALSKWPDTDDCRLAAERLAAYLDKQADLRQALDAQGIANALNALSKW
PDTAVCKAAADHLAARLAEEADLRQAMDVLGIVGALGALSRWPEADTCRM
AAERLAARLGRGPELLRAMSAQQFASALNALSKWPQSEACQQAALGLADA
LGGSGRPFGAFDMGSLAKLANGIARFAAGLAGAAVPDDEDEDGDGQANAA
LSMMHACLRELAAHLSVRPAGLDAAETRDVAMVFKALATAGLKDGLKLL A
RQGLQRLQVLHEQTRLRPDSLETLGSLAAGLLPLVRSPDLKPFRIDALRL
LERMQPDVARKVQHYTDAHAASAALAKARSPEGEAFGVRRPGLTFFLLLK
TYAVVAGQWKQRNAADDRARVAQRREALQAWIGNTLEQVRGAIEGDLDEM
SWNLIAQIEAGDHVLDALDLKLHRDMDRITTAHPPTRLDVAAVRRELRGL
SAVRDLMGSEAGAAALQVVDMRGNPIRSGPDALANYSFFTRLTGGKLPLI
EVELPGKLSAFMLARTLQHNGDLLRMDLFGGSHLTPPNTRVFEMLSGAQG
AAKRYGRIPAIRLADTVPHAPLMKDVIRKLNPQREDWYRMQRALLEVVPR
DHVVEGPVRLALQADRPQGTEPAFALRLPTGEPIHLVSHDGCGFIRESLA
RRIPAMAAAMDEWARSRRQAEPQPPAPRMTSLPPQATHHYPRNAAAIEEA
RTHLRRTLQEDATLWQAEVPDGTHRLGTTRLYELLVGAGITGVQGIAVPS
ADSKVYLPGEKSGPFDRAGGPVLLGKPPYDKPNLMPIPAERVGTPGQGDA
TARFLDTAFAFQYSYTAWDESRTRTPSDDAPMLHGKGVTIVVPDALWPKG
NDAQWVWSTEDMKIHSSWTRRRERDRLPARMDTVGSLRVKDIFAPGALIA
VPIDELGKRDADCDGDKVFVYAGAPKMAQAIDGFFSARERRIGRMPSFKP
VKTARAAVDEAGQYHAGRAVEVLSAVRGQELVRRMSTFQFHFWGQPQALR
ERIAEQAILGTFEGTRRELRRGLRRLLYSPGAATPASLQALCERARLGAA
HAQHPVARQAAKALRDQLEAFVQAPDAPPAPAGQPQALPSALAQRFTSLA
EAYAQAGTARERLEALASHYPTALLPHPGTALPEDCADDPLPLPEMQLGY
VPAAPMETLRNLLTLGVKVGTDAPKAATQTDVYLKIAERLDRALRSEPDR
IRLMPYTKNGLVKKLREGIDVPAERLLLRDNPTLAAGLMEMALEELQPLG
LIDTAPAPGAVSAETASRVWQLAHALHAAAEQAEARVTGMVERAIDGIGE
SRGEAHRLKSASSLFDKLLRLMHKGRLSPQAAAATVEDALRYSIVLDAQT
FVQRYADILGRLDTLGLTRTQVRNSFVLNHTAFKGVNVGFTAGDGDGKAV
RLEIQFHTQETFALKARFHDDYKQAQSLYLAGADTEQRHAALDQAREAFA
PVATPPGGEHILDWDSAPPRTERPRAAAAAAPAAGTHPARDGLAGHVERV
TAQARAIQREVTPLLEAIGLAIAKQHSTPKPASSVRKKIERYQALKGLSL
EQAAASVRDAMRWVVLLPADQFGAGFRQARQALEAKGLRIMRIRNGFAVL
GTTYAGLNVIVRSPAGRDFEMQFHTEDSLRARNTSHPLYRAWQDQELRIG
LEQDPAKRLRLQQANALRQQLRRERAAQVALPTGAKSIVDFDALRDAAPP
RATSPHRPQRPERPQQGKRAGHRP
> gi|51591628|emb|CAF25432.1| yscM, lcrQ; putative type III secretion regulatory [Yersinia pseudotuberculosis IP 32953]
MKINTLQSLINQQITQVGHGGQAGRLTETNPLTENSHQISTAEKAFANEV
LEHVKNTALSRHDIACLLPRVSNLELKQGKKAGEVIVTGLRTEQLSLSDAK

LLLEAAMRQDTAADG
> hopO1-3' Pto DC3000|Genbank: AAO58038
MNINPSLGAHGSAYSSPQSDTSKATGKAPAPSFFKQLGGCFSPCLGSHAS
SSQQLSASHAQTLSQNYSSNIQGTSRTRQPRAPSPRLSDTPMKQALSSMI
ERERLRLQGLSGGMFSGIDSADAMIGRALTKKDSNPKAARFSDDEFLAVH
LYTTCLYRPINHHLRYQHXXXXXMSDEEVMKRFNSGRPYRDEAFMSTSTD
SVIANSLTSSVTLHLQSTSAVNVSPFAMNAYEKEAIIPPQTPFEVVGLKK
MHSTWHVDLKEVQDNADGS
> Q4ZX47_PSEU2
MNISGPNRRQGTQAENTESASSSSVTNPPLQRGEGRRLRRQDALPTDIRY
NANQTATSPQNARAAGRYESGASSSGANDTPQAEGSMPSSSALLQFRLAG
GRNHSELENFHTMMLNSPKASRGDAIPEKPEAIPKRLLEKMEPINLAQLA
LRDKDLHEYAVMVCNQVKKGEGPNSNITQGDIKLLPLFAKAENTRNPGLN
LHTFKSHKDCYQAIKEQNRDIQKNKQ SLSMRVVYPPFKKMPDHHIALDIQ
LRYGHRPSIVGFESAPGNIIDAAEREILSALGNVKIKMVGNFLQYSKTDC
TMFALNNALKAFKHHEEYTARLHNGEKQVPIPATFLKHAQSKSLVENHPE
KDTTVTKDQGGLHMETLLHRNRAYRAQRSAGQHVTSIEGFRMQEIKRAGD
FLAANRVRAKP
> gi|28868774|ref|NP|791393.1| type III effector HopPtoJ [Pseudomo nas syringae pv. tomato str. DC3000]
MGLCISKHSGSSYSYSDSDRWQVPACPPNARSVSSHQTASASDIASGDVD
ERPATFSHFQLARCGGEYTLSMVSAAAYQAERRHRGNLIKDRSQSILPWV
QVYHSKKGLDYSFQIDRTTTVKVAGFNCSIPNNRGTRHLYSAGTSQTNMP
VIADNMSACIAVACAAENVDAGTGERRPGAKVRVFHLLPFRREDLVPEEV
LASVRDYLRTTKEQG LTMRVAMHGGNTEGDFSVSTAQALKGLFANEGIPL
EFDETCANRTSETLLGAVILDDNSTHFIKHLVAQ
> Q139Z8_RHOPS
MTLALGCIADDYTGASDLANTLTRQGLRTVQTIGVPADDLALPEVDAVVV
SLKSRSIAADQAVAKARAAEQWLRGRGAAHVLFKICSTFDSTDQGNIGPV
MDALRADAGDAMVLVTPAFPETGRTVYQGHLFVGASPLNESPLKDHPLNP
MLDPNLVRVLARQSHTK ISLADLSVIAQGPAALRKHLDDLATRGVGAVVA
DAVFERDLETIGAVALAHRVSVGASGLGLGLARALVGAGRVELNHAEASD
RESIGGFAACLVGSCSRATLAQVADAERSMPVLRLDPSAIVAGDGEAQRA
AEWAIARIADGPVLIASSGTPDQVAAVQAKFGRVAAGHAIEQVMADIAVR
LVESGVRRLVIAGGETSGAVVDRLAIPGFLVGPEIAAGVPVLRAVGACRS
GMLLALKSGNFGGPRFFT DALGLMS
> gi|28868614|ref|NP|791233.1| type III effector HopPtoB1 [Pseudomonas syringae pv. tomato str. DC3000]
MRPVGGPAPGYYPPTYEAERPTAQAAGNDRARSSQASSSPAASVAPETPM
LGDLKRFPAGRYPDMKVENIRLKIEGQEPGGKDGVKHTRRRKPDAAGSSH
VHGGQSVASTSASAQSKALQDTNFKASDLAELARWCESPHPY ALAPSKAA
GKSSQLSANVVSILLQEGKHALEQRLEAQGLKLADVVVSEGRDHLHINLN
YLEMDSCLGTSKGLWAPDSNDKKLIAKAARYFDDFNAQKLPELAPLTKMK
SKDSLGVMRELLRDAPGLVIGEGHNSTSSKRELINNMKSLKASGVTTLFM
EHLCAESHDKALNNYLSAPKGSPMPARLKNYLDLQSQGHQAPEELHTKYN
FTTLVEAAKHAGLRVVSLDTTSTYMAPEKAEIKRAQAMNYYAA EKIRLSK
PEGKWVAFVGATHATSCDGVPGLAELHGVRSLVIDDLGLKSRATVDINVK
NYGGKLNPDVRLSYKV
> gi|21230674|ref|NP|636591.1| HrpF protein [Xanthomonas campestris pv. campestris str. ATCC 33913]
MSLNTLSTGSTAGLFLPLTDDASSPGLLGSDSAMNDSDLLLAMDNLFLQQ
IYRLIAATYGNTSLNGPGSGIPGLDTPSA DDLQASQPIEKRTSWPTLSAP
FNVKDIKGSRLPPAVDGSSVTWEGGTLTPSELQIVSTLNQHKDKTPLEFA
KLDDKINDPSTPPDLKSALQGLQKDPRLFFAIGSQGDGKCGGKIKAGDLW
DFADHHQQVTALGGKNAEFNPKNIKGATPPPAAEGSSVTWDGGTLTQSQL
EIVSTLNQHRDMMPIEFAKLDEKINDPATPPDLKKALQGLQQDPGLFFAM
ASQGHGKHHHDDQGKCNGKLIADNLYDFAD RHPQVTAQGGKNATYNPEKM
KGRDLPPPVDGSSVTWDGGTLTQNELEIVATLNRHKDKCPVKWTDLDAKS
KDPAIPPDLQKAFADLQQDPALFHAIGAQGSKGSCDGKFTEKDLTRFSVP
EKHAQIAQYAEQQAKGYTQNYVASDSPDKTEPTVMTESDAMRELYRYSDY
LPKDLNQDAFKQLVEGDSTTKKSPPQVIAAAQYFREHPDQWKALAGDKES
MSTADFLQKSTSEMHLTAPELKTLDTINSHQ EAFFGDGKEVTRDKLDTIV
KDDKADPAVRDAAKQLLGDPLLFGLLNNAITGYKKPHSFFGGGHVVDSGK

ISNKDFQQFYEHMTAVNKTLDTPPTHAATSPEQKKAVADMLMGKADQPEI
KRKKHDVGTFSKGLHEFLKWDSKILDGISVALSAMNGIPLIGEVADAAAL
AFESEAQAAQVIDTALQGGNLSLAWKLAGINMAGAVVGAVGGPTARLAAK
GAAKGVAEGAAKGATQGTTKGAAKGGGKGVAE REKPLDIAKGYIIGTSIN
RPTEMLKTPVLAGLHYEEVRLDKEKKKGEIRKNLEAAGGVPLGKQFIPKA
IADNFEADTKENLRHVRGRRK
> Q8XZP6_RALSO
MPTRVPSPTLGRSHPVTGAASTATSTAATATRSGPATAPASGRSREGLLA
ELPTGRTARTPSVSAAGAHAGRPAWNVQGAIEAMAALSPEREKRLFKKSA
SPQYRYAKGLTAEQRGQLESALEQRFRNPAAPAEARDSALAMWLSVQ QAR
LRTHTAGHRNHHNLEQFQLAALSVPIPLLALGYRRQRRRYYSSPLRPEYR
TAFNNFMRVIGDPSLSEAVRQTVAQRLEYHRRSEETIARHERQLLGEHGV
MGLAESGYQIGTNYDHVNLTALEREAVVESRGSGVPPALHIQALQTERSR
VESGALRHQWLTRELRDAQARAGQGEAVAASAGPSSVRQVDAAESSAAAA
RLRRDTANDIRRLRRPLAAEIKQWLKLAHAEPLPDPKAFDNELYANAF AR
LLERRRPLSLISYFARWDPVVVDGAKVIQAIAKDANLRKEVFAAAETALG
TCGDNVADGFANIVTMVDTHQLVDDVRSGKLDQPALEAWGRQRYRLDSLI
TEVNQWMASRRRQAGQHSIMTERRVAREPLETMLHAKVALKTVLDLPKNL
PSSMRHRLASALKPDDLKRLAETVQAKEADPVELARYLLSNDAWRGAMKA
LHPAAFAALRKRFAPEKDALAKEIPPQPTDPEGLEFLDERMAYAERTDA F
TQKCRAAEDTLLLSLAGRYALVPAVVGAGPSQGSR
> Q8XU25_RALSO
MPPVLPSILRCFRPAVSRPEAETAAPSSSQEHNRPGSPERSPRRAPAALQ
GLTPRAGSSRRQAAPEAPAGPARFLTDGERQFGGYLMARDVDQRPVHGEP
LDTLRSANETLLQTRRILTHGRGNVEDDIDATHGLSTHIAQGGRSIQESM
WRAHPKPVVWAAIAMVAGAGNCGEHADLATFLHAAKLKEGEAVDNVHIDD
FDHFWAIVHRAEPDLERDVYIDAWGKGPAIFAVDGMMTYRPGERRTKFGY
DKASGEEAHADMEMLATVLATRMRGGISNTMRRLGPDYRYPPERVWAVTP
IVAQRFTDRVKAEMSKPADLGKLMVPPDCATPSSVEPPVTNERLMQPLRH
EIHATRIARTLGAHSVDTMAHAARRIVAVASDLQGYPIEAHPLQAKKDAE
DIAAAERRRARRAALGKGEPPATES
> gi|57434450|emb|CAI4386 7.1| translocated intimin receptor [Escherichia coli]
MPIGNLGHNPNVRALIPPAPPLPSQTDGAGGARNQLINSNGPMGSRLLFT
PIRNSVADAADSRASDIPGLPTNPLRFAASEVSLHGALEVLHDKGGLDTL
NSAIGSSLFRVETRDDGSHVAIGQKNGLETTVVLSDQEFSSLQSLDPEGK
NKFVFTGGRGGAGHAMVTVASDIAEARQRIIDKLEPKDTKETKEPGDPN S
GEGKIIEIHTSTSTSSLRADPKLWLSLGTIAAGLIGMAATGIAQAVALTP
EPDDPTTTDPDTAASTAEAATKDRLTQEAFQDPDKQKVNIDENGNAIPSG
ELIDDVVAQIAEQAKAAGEQARQEAIESNSQAQKKYDEQHAKREQEMSLS
SGVGYGISGALILGGGIGAGVTAALHRKNQPAEQTITTRTVVDNQPTNNA
SAQGNTDTSGPEESPASRRNSNASLASNGSDTSSTGTVENPYADVGMPRN
DSLARIPEEPIYDEVAADPNYSVIQHFSGNSPVTGRLVGTPGQGIQSTYA
LLASSGGLRLGMGGLTGGGESAVSTANASPTPGPARFV
> Q4ZX84_PSEU2
MSIGINSSTSYQPASTQLDFSALSGKSPQTNTFSDESTAQGVDPSALLFD
TARQKDVSFGQPDNTVQNPTDSSAATDPQSNVVKLLSALVTSLLQMLMNL
NKKQDTGQDSNEWQDPFQNEGGLGTPSAEGSDGGTQEASGGDEGGGTT AA
TGGDGGGGTSPTTEGDGGGTSPTAEGDGGGSYVSTGADGSGAPSTEDGTG
GGGGSDGVTPQVTPQLANPGRNSGNGTVSDTTGSLEQSGEVNVVKDTIKV
GAGQVFDGHGATFTADKSMGTGDQDEHQKPLFELAEGAVLKNVNLGENEA
DGIHVNAKNSEQVTIDNVHAQNVGEDMITVKGEGGAKVTNLNITNSSANG
ADDKVIQLNADTHLKVDGFKATDFGTLVRTNGGKQFDDMSVELNGVDAT H
GKFALVKSDSDDLKLATGDIAMTDVKHAYDKTKASTQHTEL
> A1FFL0_PSEPU
MACPTPNGKPSTRKKPAPTSKRRLKKENLVTDLNTLRASLASGKHAFADT
LAFIAANYSYQPQAFTNGGVENAAGQNEGSCKTLGLALLEGLSDQEALLA
FGEHYRDVAATPEGTDHGNIRALIAHGLAGVKFTAQPLSRNA
> gi|3414586|gb|AAC62314.1| HrpW [Erwinia amylovora]
MSILTLNNNTSSSPGLFQSGGDNGLGGHNANSALGQQPIDRQTIEQMAQL
LAELLKSLLSPQSGNAATGAGGNDQTTGVGNAGGLNGRKGTAGTTPQSDS
QNMLSEMGNNGLDQAITPDGQGGGQIGDNPLLKAMLKLIARMMDGQSDQF
GQPGTGNNSASSGTSSSGGSPFNDLSGGKAPSGNSPSGNYSPVSTFSPPS

TPTSPTSPLDFPSSPTKAAGGSTPVTDHPDPVGSAGIGAGNSVAFTSAGA
NQTVLHDTITVKAGQVFDGKGQTFTAGSELGDGGQSENQKPLFILEDGAS
LKNVTMGDDGADGIHLYGDAKIDNLHVTNVGEDAITVKPNSAGKKSHVEI
TNSSFEHASDKILQLNADTNLSVDNVKAKDFGTFVRTNGGQQGNWDLNLS
HISAEDGKFSFVKSDSEGLNVNTSDISLGDVENHYKVPMSANLKVAE
> Q8XSA6_RALSO
MLIQTQYPGFYIATASDNQREADRYAGKVNEALGKIASGR SGDELLRGIS
SLSATRQRKLTISEIDSDDDPGTEAVLTRPQIAAYEPSDFRANKRIAKQF
ARGEVSSEPAGCSAIVNWNPKTSIKLSRNGSPKRLHKDPKESFAVLAHEL
IHARHVMAGTSKAWSGDRYNETSEAGQEELRAVGLGAYAHAYTGEPTENS
IRAEQGLQARSKYKPRNA
> gi|34498034|ref|NP|902249.1| probable secreted protein EspA [Chromobacterium violaceum ATCC 12472]
MNTAVNQPPSGVNVPTTGTADSSDNNDYLRAARNYSLLGQAITTMEEVML
LFTELSNAKFAQMSKKMEVSRDAQEKANVMEAVLASLTDPNSKGQLPPDV
IEYIRENGILVGNQTIDDFIRENGQFVGCVSEGRFEKMDEYIKRLANLIE
SCDRTGSDLHPFERMKAFSDFMDALGVKVDGKSVSDLINETLYIDENGYE
RVPVAVLEKMQNALEEAKMPVFNI SSLTAVKSSLESFSGRASDFVQQSQL
KMQQLIQNFNTAVTMANSLQSMNAESTKSIAQAIR
> gi|12329095|emb|CAC05826.1| Spa32, secreted by and component of the Mxi - Spa machinery [Shigella flexneri]
MALDNINLNFSSDKQIEKCEKLSSIDNIDSLVLKKKRKVEIPEYSLIASN
YFTIDKHFEHKHDKGEIYSGIKNAFELRNERATY SDIPESMAIKENILIP
DQDIKAREKINIGDMRGIFSYNKSGNADKNFERSHTSSVNPDNLLESDNR
NGQIGLKNHSLSIDKNIADIISLLNGSVAKSFELPVMNKNTADITPSMSL
QEKSIVENDKNVFQKNSEMTYHFKQWGAGHSVSISVESGSFVLKPSDQFV
GNKLDLILKQDAEGNYRFDSSQHNKGNKNNSTGYNEQSEEEC
> gi|28867732|ref|NP|790351.1| type III eff ector HopPtoF [Pseudomonas syringae pv. tomato str. DC3000]
MGNICGTSGSRHVYSPSHTQRITSAPSTSTHVGGDTLTSIHQLSHSQREQ
FLNMHDPMRVMGLDHDTELFRTTDSRYIKNDKLAGNPQSMASILMHEELR
PNRFASHTGAQPHEARAYVPKRIKATDLGVPSLNVMTGSLARDGIRAYDH
MSDNQVSVKMRLGDFLERGGKVYADASSVADDGETSQALIVTL PKGQKVP
VERV
> SPTP_SALPA
MLRYEERKLNNLTLSSFSKSGVSSDTRLYIAKENTDKAYVAPEKFSSKVL
TWLGKMPLFKNTEVVQKHTENIRVQNQKILQTFLQALTEKYGEKAVNNAL
YMSSINMNKPLTQRLVVQITECVKGADGGFINIIKNKDNVGVMNAALVIK
GGDTKVTEQNNDVGAESKQPLLDIALKGLKRTIPQLEQMDGNSLRENFQE
MASGNGPLRSLMTNLQSLNKIPEAKQ LNDYVTTLKNIQIGADRFSQWGTC
GGEVERWIDKASTHELTQAVKKIHVIAKELKNVTAEFEKIKAGASMPQTM
NGPTLGLARFAVSSIPINQQTQVKLSDGMPVPVNTLTFDGKPVALAGSYP
KNTPDALEAHMKMLLEKECSCLAVLTSEDQMQAKQLPAYFRGSYTFGEVH
TNSQKVSSASQGGAIDQYNMQLSCGEKRYTIPVLHVKNWPDHQPLPSTDQ
LEYLADRVKNSNQNGAPGRSSSDKHLP MIHCLGGVGRTGTMAAALVLKDN
PHSNLEQVRADFRNSRNNRMLEDASQFVQLKAMQAQLLMTTAS
> Q8X5T9_ECO57
MRYNGLNNMFFPLCLINDNHSVTSLSHTKKTKSDNYSKHHKNTLIDNKAL
SLFKMDDHEKVIDLIQKMKRIYDSLPSGKITKETDRKIHKYFIDIASYAN
NKCDDRITRRVYLNKDKEVSIKVVYFINNVTVHNNTIEIPQTVNGGYDFS
HLSLKGIVIKDEDLSNSNFA GCRLQNAIFQDCNMYKTNFNFAIMEKILFD
NCILDDSYFAQIKMTDGTLNSCSAMHVQFYNATMNRANIKNTFLDYSNFY
MAYMAEVNLYKVIAPYINLFRADLSFSKLDLINFKHADLSRVNLNKAILQ
NINLIDSKLFFTRLTNTFLEMVICTDSNMANVNFNNANLNNCHFNCSVLT
KAWMFNTRLYRVNFDEASVQGMGISILRGEENIPINSDTLVTLQKFFEED
CTSHTGMSQTENNTHEVAMKI TADIMQHAD
> Q4ZPQ3_PSEU2
MNTINRNYYPVSGIPAQDAPAQTDQLPPQGQGIKPGHNSNLIDFGLTQQA
NGPHSSLNTLGSRVQPTDTNTSSNMPGGNGEQVLNKLVQAIRNILNNLLS
LLEGNLHNGSGPAQPQRDQTPTLAQSPSSASPSSSPPSTPQGNAEKPFVV
QNDHPSEKPVSLQKNTEPTSAAPSQTAPRTTERSSATPDKTPARPDAVNP
AVVHDLALPKTSTDTTAKPDNTVNAAK TATPAAHGQGADMSGIMGFAKEA
NTTGGNGGEVVTVNTVADLKKYMEDDKARTVKLGANLSADSKVTINFGAN

KTLLGTDKGNSLHNIYLASGKTASNDIFQNLNFDHDSRYRENGDMQMFIS
SGQKYWIDHNTYSGTKDQNAKGLDKLLYVGGTADNVSLTNSKFQNNEYGV
ILGQPDDSAQAKAEYKGYPRMTIANNVFSNLDVRAPGLMRHGQFDVLNNS
IDNFHLGFTATGDATILSQANYFAKGVD VSDKASNSGVLDDYGDAHFKDI
GSNVSFTQKSAVTAWTPSYQRDIKTAEAARAYDQANAGAKTVS
> gi|34497729|ref|NP|901944.1| hypothetical protein CV2274 [Chromobacterium violaceum ATCC 12472]
MLGFNGERLDPASGAFHLGNGYRAYSPALMRFHCPDSLSPFGASGVNPYA
YCAGDPANRADPSGHLSWQAWTGIGLAVASLALAAFTAGT SIIASGGIVA
ALESASAITLAEGGLAVVSDMTAIACGAMEASDPQAAAALSWVSLACGAM
GMVHAGYGAVRGIDRKLGAFLQENSRFLGKITRGERDSLGIPLGGGLTKC
FRPASEMEGASAGIALGRLHRSSSTPDMEAFLALPAAKRKRIGSGVSGVV
YEAGRGWVVKIAIDPLPIETMEREAEIFRAVHGPRSALVAENMLWMKRIP
GVPVNTLNLLADEKAEMWQKTLAEVKRLQEMGIHHGDPSWN NLLFDQQTG
TVSFIDFGKSRYLDGFAPAT
> SOPE_SALET
MTKITLSPQNFRIQKQETTLLKEKSTEKNSLAKSILAVKNHFIELRSKLS
ERFISHKNTESSATHFHRGSASEGRAVLTNKVVKDFMLQTLNDIDIRGSA
SKDPAYASQTREAILSAVYSKNKDQCCNLLISKGINIAPFLQEIGEAAKN
AGLPGTTKNDVFTPSGAGANPFITPLISSANSKYPRMFINQHQQASFKIY
AEKIIMTEVAPLFNECAMPTPQQFQLILENIANKYIQYTP
> Q8XYN5_RALSO
MKSSDRIIKSSARSREWPATFEDADDLQPSITNAARQSQRETPRHPLASP
TFLEKFTQQTSALLLYAAIRCLIGVALTFYSFHRNRCQTILHRSIDSAPK
KKKAAQKKSNRRIKTNLRRNKIHIQPIRFDLCEKRIRKKFQIRRAVR
> gi|27376900|ref|NP|768429.1| hypothetical protein blr178 9 [Bradyrhizobium japonicum USDA 110]
MATMESVVRRMVPQDYAEMQERLVGVMGVQFETRLDERLAEHGLTGDADA
EPVLGARYETRLPARSKARSCIRSSGSSAFSYERTSEGGSVDPPRRSQKS
KFNRRLPGRTACMTDCLHEPLPNELC
> hopAN1 Pph race 6 -1448A|Genbank: AAZ35071
MSLNNARPLLGCIADDFTGATDLANMLVRGGMRTVQSIGIPSAEM AAGLD
ADAIVIALKSRTTPSADAVAESLAALEWLRERGCEQIFFKYCSTFDSTAA
GNIGQVSEALLEQLDSDFTLACPAFPENGRTIFRGHLFVQDQLLSESGMQ
NHPLTPMTDANLVRVLQAQTRHKVGLLRYDSIAQGVEGVRNRIAELRAEG
VSMAIADALSDADLYTLGEACADLPLLTGGSGLALGLPGNFRKAGKLRDI
DAAKQVAISGGEVVLAGSASVATNGQVAAWLEDNRPALRINPLDLA AGKP
VVEQALTFARDAGQTVLIYATSTPDEVKAVQKELGVERSGAMVEAALGEI
AKGLLNAGVRRFVVAGGETSGAVVQALGVQLLQIGAQIDPGVPATVSSGA
QPLALALKSGNFGARDFFAKALKQLAGAA
> CP0133 ipgD IpgD, secreted by the Mxi -Spa machinery, modulates entry of bacteria into epithelial cells 116362:117978 forward MW:59895
MHITNLGLHQVSFQSGDSYKGAEETGKHKGVSVISYQRVKNGERNKGIEA
LNRLYLQNQTSLTGKSLLFARDRAEVFCEAIKLAGGDTSKIKAMMERLDT
YKLGEVNKRHINELNKVISEEIRAQLGIKNKKELQTKIKQIFTDYLNNKN
WGPVDKNISHHGKNYGFQLTPASHMKIGNKNIFVKEYNGKGICCASTRES
DHIANMWLSKVVDDEGKEIFSGIRHGVISAYGLKK NSSERAVAARNKAEE
LVSAALYSRPELLSQALSGKTVDLKIVSTSLLTPTSLTGGEESMLKDQVN
ALKGLNSKRGEPTKLLIRNSDGLLKEVSVNLKVVTFNFGVNELALKMGLG
WRNVDKLNDESICSLLGDNFLKNGVIGGWAAEAIEKNPPCKNDVIYLANQ
IKEIVTKKLQKNDNGEPYKLSQRMTLLAYTIGAVPCWNCKSGKDRTGMQD
AEIKREIIRKHETGQFSQLNSKLSSEEKRLFSTILM NSGNMEIQEMNTGV
PGNKVMKKLPLSSLELSYSERIGDPKIWNMVKGYSSFV
> CP0128 ipaB IpaB, secreted by the Mxi -Spa secretion machinery, required for entry into epithelial cells 111248:112990 reverse MW:62172
MHNVSTTTTGFPLAKILASTELGDNTIQAANDAANKLFSLTIADLTANQN
INTTNAHSTSNILI PELKAPKSLNASSQLTLLIGNLIQILGEKSLTALTN
KITAWKSQQQARQQKNLEFSDKINTLLSETEGLTRDYEKQINKLKNADSK
IKDLENKINQIQTRLSELDPESPEKKKLSREEIQLTIKKDAAVKDRTLIE
QKTLSIHSKLTDKSMQLEKEIDSFSAFSNTASAEQLSTQQKSLTGLASVT
QLMATFIQLVGKNNEESLKNDLALFQSLQESRKTEMERKSDEYAAEVRKA
EELNRVMGCVGKILG ALLTIVSVVAAAFSGGASLALAAVGLALMVTDAIV
QAATGNSFMEQALNPIMKAVIEPLIKLLSDAFTKMLEGLGVDSKKAKMIG

SILGAIAGALVLVAAVVLVATVGKQAAAKLAENIGKIIGKTLTDLIPKFL
KNFSSQLDDLITNAVARLNKFLGAAGDEVISKQIISTHLNQAVLLGESVN
SATQAGGSVASAVFQNSASTNLADLTLSKYQVEQLSKYISEAIEKFGQLQ
EVIADLLASMSNSQAN RTDVAKAILQQTTA
> hopM1' Pph race 6 -1448A|Genbank: CP000058
MNTPRIGGSGAIELSRINQQPDAIPAQTAHPNAVTPGMNPPLTPNQAGPH
AAESSATGAARLNVAARHTQLLQAFKAEQATAPVSGAPMISSRAALLIGS
LLQAEKLPFEVMAERLSPERYQLKQFHGSDLQQLLDKFTQPGQVPDKAEV
GQLIKGFAQSVADQLEHFQLMHDATPTKTGPHANEDRATLAVSQ TALGEY
AGRASKAIGEGLSKGIVSLDDHIAALDVSLQSAEEGAKDALHSNRQALVD
AKTTLVGLHADFVKSPEAKRLASVAAHTQLDTVVSDLVTARNSVGGWKGA
GPIVAAAVPQFLSSMTHLGYVRLSTSDKLREEVPETSSDASMLKAAITGM
VTGIAHETVNSVVKPVFQATFQKTGLNERLNMVPLKAIDTNSVIPDPFEL
KSEHGELIRKTPEEIAQDKAFVKGERAVLNQKKVQGSSTHPLGEM IGYSA
FGGSHAVRQMLNDLHQINGQTLSARALASGFGGAVSVSSQTLLQLKSTYV
DPAGRKIPVFTPDRAETELKKDLAKGMDLREASVRTTFYSKAISGYSELG
ADLGTAACDSPAGRRARYAQCGEYPAQ(siteofframeshift -downs
treamsequenceisnotsimilartootherHopMfamilymembers)
YGSGRNGLDFLSVHALRQPVCHRRSQGVEGCGDGGRNADAGSYRNS LEQH
PPSEQGIAATYLPAEHVERYSKGYGKRLSHGTRRVAAANPDGRGHGSSVG
RRCAERRVVSTRCAYASKTA(whatfollowsistheHopM -homologu
ssequenceinthealternateframe,post -frameshift)MALVA
TGSISYLSTLYANQSVTAEAKALKDAGMGGATPMLDRTETALNNIRHPNR
ASLPHTFQPSTLSGIPRAMESAYHMGRGALQLPTQMAVDTVRVLADG ALN
GVSSARAALTPAKPPEARVSVDELRNTAPTPPSSPTVQRPAPSVPLDDEQ
LRALEESLLAPR
> Q8XXH5_RALSO
MSIGRSKSVAGASASHAPASGENGSTQPQTVTPLAGATRRRRNSGLLCEL
QSMCKRTLRAASQTIIFPFYSKTGTTPTRAQPKPDVVPALKPAPRRMRPP
AAPGRKHQRNVDAVVPPAMATSPRAEIRFPLTTADAQRYLRAQWRQAETG
GRFDQAAVDRLTTTLTARLL ATSAATGNGVHEDSGEKACARAIMVCDALA
QATGGDPARAHDALLALLDTTSHADARVRADVLALQTALGMTAIGLDTLL
AIAPHVLPQRGSAAPEIQREALRHALRAADHLLRRKPADAPGPTSLEALA
ALADPVLPAVERGRPGSSAGASVAPPGKAPAWLADPGNALAIKALHAANA
LRADPAAQCPPHLAQAYLAWRNGFDREGPGTDLAKAQQRLFKLFAYAERA
ARTGMAARAAAGFFGTQKSPL SALQNFGTAGVMLGHPDDEFARFTAALAP
VKTQLADRLKAASTSRALKTRCAVRLAAIEQWERRMASKGLRSTFRFSSR
DLETVAARARPLLSNRVALHADGSPADLALDPHRNRDAIRAEVKPLRGMT
PAQLRTWADEAWRLSGQAVPEPIRANIDLVESRLAGDIRPKPGDTNAQLD
AIGALVKQMPDIYDIRVSSGGTVGLGGVPSQSLAALSSHLGVPHVSVLPD
GGYLQGRHAVIDIGSNQHFGHL FIGTESRKSLYGGLGGYAGWSFGQDGMA
NVGVSGGLRRSRDWGGPRGVTIRTRRSDDEQPGRPDAWRTTMLEVLHATR
SAGPNSDAPRNAREMWGSLARRFWNDPACSINWTDSRSTTSATSGSASAT
ARVGTADTKWGPALGATLRHVARAAHRQWDKTGNHAIDVSTHNSGRATAV
AATLVEALPGIPVPNGSGHLAALSFPTQPYVGIGTTLFTTQTNAALRIGR
DSGRIIPKHTFRDTEFGTFKAFK QFVDTHRSEWLTALGGTDDARRRLDEM
VATVKARATAGNLIMGERMHMTDEAARRLDFLFHRKQRFDRIPAPTPVQA
RERASIDADIRQMLASENSWRRQALYVLEALGVQRTVGLSFLLNAQSTQA
VSGVRELAGLSPE
> Q1UBI8_9GAMM
MSIRHSDVSALLNGLNKKAIGFNDVLNFIDDFYRYTPVKFSNGDLVNAVG
QNEGSAKVFGFAKHHGLNQLDTLKLFAEHYDAVVAAPKGTDHPNIR NFIH
WGWQGFLMEHNPLTARPSVDVKNI
> PIPB2_SALCH
MQRSLDSLAGMATSAFGAGTSAAMRQATSPKTILEYIINFFTCGGIRRRN
ETQYQELIETMAETLKSTMPDRGAPLPENIILDDMDGCRVEFNLPGENNE
AGQVIVRVSKGDHSETREIPLVSFEKICRALLFRCEFSLPQDSVILTAQG
GMNLKGAVLTGANLTAENLCDADLSGANLEGAVLFMADCEGANFKGANLS
GTSLGDSN FKNACLEDSIMCGATLDHANLTGANLQHASLLGCSMIECNCS
GANMDHTNLSGATLIRADMSGATLQGATIMAAIMEDAVLTRANLRKASFI
STNLDGADLAEANLNNTCFKDCTLTHLRTEDATMSTSTQTLFNEFYSENI

> gi|15618212|ref|NP|224497.1| Inclusion Membrane Protein C [Chlamydophila pneumoniae CWL029]
MTSPIPFQSSGDASFL AEQPQQLPSTSESQLVTQLLTMMKHTQALSETVL
QQQRDRLPTASIILQVGGAPTGGAGAPFQPGPADDHHHPIPPPVVPAQIE
TEITTIRSELQLMRSTLQQSTKGARTGVLVVTAILMTISLLAIIIIILAV
LGFTGVLPQVALLMQGETNLIWAMVSGSIICFIALIGTLGLILTNKNTPL
PAS
> gi|53722632|ref|YP|111617.1| hypothetical protein BPSS1609 [Bu rkholderia pseudomallei K96243]
MPRIQSSSPPITPSITANQPTNGANARAAVTRQKRPAGHATLSKLASSGK
KVLHARMPRKGKLAQPGANSSGAATSKPAVPKADAPPAHAQVQANAHEGD
LIDLSDPPPTTKPAGMQPSLLDAQPNDLHALHTPVLQPTVLHSAAAPAAH
GAQPLGQTLAAAGMPSLQDIHHTTDQAIAMQHAIGQANQRLAIAEMLNSF
MQKVIDMIKKAASQP
> gi|53722413|ref|YP|111398.1| putative ATP/GTP binding protein [Burkholderia pseudomallei K96243]
MTSMLFLKEIMPVIIRSSGASIHQSDLSSRRSEADASDPAAAGPAREPDR
VAGAESRSFVQPAPLNKTADKVKGGTPAIPLSLQVLGLYQRDKVCAQPVR
PDHASLPESTRAEGLPRLDGKLPKRIETAPFARALVQGNAGSGHNSRDVF
LNVTGGVS GKAAPSNADHLTDVLLSDCRPGESLAGTPDDNAFVIGGHSEI
IDVATRPDGLSRADKARQMAIVMFGVTDPANDQIAFAERKLTALENAEAR
HLAMSLFTGEEGREALSGWIADFDPGGAARLQRMHQLMYAAAVVLKQNGR
AAARLLRNYCGMSTERPDLQLKRQSYSMQRVLVSARGTSLDILGIPPSIT
GREHHAEQKSVYRNALAIAQYLHEKLDAAYKPDSLEDMLAAAQIVSAEAL
GGSGDRTEREYHFSRVVDNLGSIYQEERLSIDALACAANLISIDRDNGAK
ALGAVAMLSSSEQADVMDALEQAGELVRLLPLGIRRDIATVADLDKVLNA
NAEGPGARASVSSVDPMGQASAQKAIGKLRAALAVAPDIGNIPIGAAARA
AIPMQQKIAYFKWRNGLADERKARLVFERLYKLNKYADRAIPRGKNLTGR
LRRIGDDIAGAFGHRKNPVGMLEEGLLLGGFLREDVFNEKIAPAFHSLIE
KYRESGAGAG AGAGAGAGVAKARSEVVSRVFEKLKIDGFSSKIEIKSDDL
KKWVALAVAGGRNDAVKRSLEFESIRRDLKNGFSVDVLERWAAEAGLDDG
NFRRQLNDLRSVSNNGYGEIPELGDRTSVFSAFSEIVARYRAGKSLKFSS
GGVGGLALGDSQSIGPLVSVSPTLGAGGGRVATVEIGGASGRGGLIAVTR
ANSQKLKVGASVFAGPQFIHVVRAGGSANLELVSAEQTREKGAIVRFVPR
AGSLGGGEAWR GWAQDCLSIIGNSADSADLLERLAEEYVNAGEIGIGMTE
KEENVISSSAAVGAGVKASSSGGDFRGGAGVSASIGVKREWRRHKAVREL
DNKMGNHKFDQSSSTSVTWNAGLNASESYQLNPTPDFSTSASASAQAFSI
GGTLFSVKEGTSVAIRLRDGMVDAELAILDRTRNGGRRAVDLIARDYPIW
VKAMGKTDAEGEAALNRFFASELGRGVNRPLRPEVPETYVASYRMTKEAA
SALNHYRAQIDL EQMLGTNKSRVSELRRRYAEVIENSASWRPSSIQHYVE
TSSAKSVGLHNVFGVEAKKSSSTSTVQVLAHQAASEYVDPVALARHPVSV
GSVQAGGDAPSDVGGRSTHGSAVKTGE
> Q415V5_KINRA
MSDYGTAPVIGAIADDFTGATDVAVAFRRAGLRVAVHFGVPTAASAAVEA
GLDAVVVALKSRTIEPTEAVEQSLQAARWLQDQGVRQLYFKYCSTFDSTA
RGNIGPVADALLELTGAATTV VVPASPAHGRTQYLGNLFVGDVPLAESPM
RHHPLTPMTDSNVQRLLQGQTTHPTALLTRSVVARGAAAVRQSLQAAAAA
GTDYVVVDALDEQDLLTIGQACVDLPLLTGAAGLAGGLGAALAQRERAVS
QRAAGTASPPVEGTLSADAPGADATGGDVPGGDVPGGGPAAVLAGSCSAR
TLEQLTALRTAAPGSSAHPAHFLDAVQTPDAAALAEQALNWYEQQDPAGP
APLIYSSQPPQQLRAAQQALGV ERASLILEEATGLIARGLAERGVRRLVV
AGGETSGAVVTALGVSDGLIGSEAAPGVPWIRTARPETGRGATQGADDGD
GRHLWLLLKSGNFGDPQLLVRAADTASPASSGDTGSPASSGDTASPASSG
DTASPASSGDTATETAVPA
> A0WHJ3_9GAMM
MLLGVIADDFTGATDIAGFLVENGMRTIQVNGIPSANMEINADAVVVSLK
SRSCPTEQAIEQSVAALKWLQAQGCKQFYFKYCSTFDST TKGNIGPVTDA
LLDVLGEDFTIVCPALPVNGRTVFNGHLFVLGQLLSESGMRNHPVTPMTN
SNLMDLMDAQSKGKTGLINYQSIEQGADAIIGEFAELKAQGKRYAVVDAF
TAEHLNEIGKAVSSLKLVTGGSGLAAGIAKNWGEHLQDQTQAKQAGHPAK
APTVIFSGSCSVMTNKQVELYKAKASFFALDIEQCMSNENYVNEVFDWVI
SHANESLAPIVYATADAEKLKTIQQQYGAEASSQAVETLF SMLAIKLQDS
GIRNFIVAGGETSGVVTQSLKVDSFHIGPQIAPGVPWVKAINQDLSLALK

SGNFGDENFFTTAQGYF
> Q8XRH4_RALSO
MAGSHCSPRRPMPANNRIGSQRLVAPQYRRTADQENIAPRKPQRSDKQPR
TRQEPFKGLAPMARAPLQARNLNIGASQPQPRPQPKAAPAKPHLIRTPGH
GVAASTAIQNQREIVRRQIEALGRREGASATGRPQQVMKLLNARTPDLPD
AKSIELLRQLQVILRLPNPASRNSHLAMFVANNRLVAGAAVAKPATAAAP
APAPARQASRPAPMRQAARPAPPPARAPQAARPAQAARPAPAPQPQAQPA
QRIFKSFQDLATQGPFARPARPAPQPSRPSRPAQRFDDFKPRNGAAPARS
RPAATAAPLRTASAPPQYGRTASISAEPTQQPILTIGDFIRFAQDDAEQE
SLRRYSMPEIYAKAQKNFDAYLTRLEGRTEPQPRPARTQSAPPRMQTVPA
ARMDTSWHG KVDQETDDRNRYHNERGGHQSAREDYAARQQPAAYSTAPRP
RRTQSLPELTAAQIDSIHRFEDQRADQENEYPNHDEAVRRYKQQNGYR
> Q2RPK8_RHORT
MPLLGCIADDFTGATDLAAMLVGNGMRTVLMLGRPKAATESPKADAVVVA
LKSRTTPAAQAVSDSLDALTWLRAAGARQIVFKYCSTFDSTAQGNIGPVA
DALAEALETDFALVCPAFPANGRTVYQGHLFVGDHLLNESGMRDHPLT PM
RDASVVRLLAAQTPHRVGLIALPVVEAGVPAIDEALRALRATGVRYGVID
ALTDDHLRTIGAAAAGHRLITGGSGVAMGLPENFRRQGLLPFRDDSASLP
KVDGGAAVLVGSCSQATRQQVAQARPHLPTLILDPLATPEAGALMAIARA
WIDAQPAGAPLMIVGSAAPEAVAALQKRLGAGPAGALIETVLAGLAEDLV
ERGIRSLVVAGGETSGAVVSRLGITSLRVGAEIAPGVPWTLADHPAGPL R
LALKSGNFGSATFFLEALGLPL
> Q8XTK9_RALSO
MKFFSFRSSVHTASTAAPAARDAASSASAAPQQPASASAGSGRISHALHG
LKRCLRPSAINVTAVPGFTKTTIDPLPIPKRPPPPNLPKAPKPAGQHKLD
RIDRQVHNLTQKLYPPLPSGLHADPEAKRAIASSRAFVSAVEALFVPSGA
GVYAVPGLQTLIDGLAGMAEAQPDAVDTTRYAGMLQCARALATATGGRAD
EACRALEHLRDR FSLTDGSNGAPPTPAQMHAWSTAKLLAHTASGFDALLA
LRPTLAEVDATLPDDDNMNGRMKREGLRTFLQAADLMAARLPAGVVPPQT
PHAQLQDARARLDAAADRASLAGDGLALNALVCAAQVHAEPHEAAHAVDD
RAQVAAYVAWRSGYREGGKGSALERSLGRMHKFTTWARRAEHRATHPLAA
FDPRRLLGMRKSPLTAAAYGTGGANLGLLNQEAQALHDTVKHGIEAMQAH
STALRALNGRHAL STEQRGLLVLREAVLQHWAQSIGTTWRSSKLKLSDHD
KRAIADRVRKAAPGARVDAEAVLAYREFRKLDKLDLKTLVRWLDEARAFD
PRASESPTLRAAADNIVKAGDIERGRPIKPRGTTLADFREALTGAIGQMP
LGNYVRYFDGATYGVNTNMTVNQHEFSHNSLVPSMGLGPGARELTGRHAF
VEIGSSSYGGEVFIGTDKRSSTGVGAGLYAGFKVGIKNWHLSAGFSAGVA
HAHDRSAPVGVIVR TGLTYGADGKATSAWRDNVADVTRFLFQTAAEGQAA
RPVPPDRMWEQFSARFFRTPDISVNWRDQRRSSHTVTKHGSGAVRVAAGA
VRVGPAFSVGHDQVLASKNDRVDTNGWLRGVERARARASNVHVSGSLAAV
AQGVGHFSNRSGFPESVTLPSVPIVGASANILPSGTSVTLRRVDEHGRLN
PRFIRRLVEFIDPSAFLSHMQARLPQIAHSDASRAKVGAFMSAMRDMGTQ
GNLAYGESWKIRPEV TEVLNAYTDEIQLMLKCAKAAPGAGGQAIQPAQAA
QTYHDLIERIFETVERETAMPPDEQAQAFRQALAGLRDQEVDQILRLANE
TIRVLKDVQSWKLSGYYAYDITTHGYTAGPAMLLQATASTSVAGERVLAE
LAVRDLELMDDPAHAAQDKDPAAGEVL
> Q8XB17_ECO57
MVAKLKPDVFVNTNPFLEAMYKERLSHKGYSDKIALSINDKKYNVNSKDI
ENILDGKGDLFKKRTLWEFVRDLF PGSHIKEVKGLIYEFVTKVDNKAEVF
DKIKSLAKKEQQWRFSTKTDFTTNENNEVIVSRSFNLYTGATSNDNEKKQ
VSSERLTLDNYLDDLHFDNSPLVRLTFDNYSVKLATLIKNKIPIINTTIN
LSSLSKDVLNSLKYCSFKNVIFSGVINSPNLEGPVFENCYFDDCKFNNIQ
LYETNDDTVESGNKKPIKGMFKGCFISKCKIENYRCETSKIYTVTQPDSI
QEKLGSYLFMQSFVQDCIIQGGCCP GSSILLSHFYNCNIIGLDAHGMDFL
ANSFNKSNYDEIRGPDTGTVFYNCNLKHIKINDGLREDGSDRTMFNPKEY
FSDYIERRSRNLKLDDNFSKCVESIKKGTDSSFSKKNTENNNVFFKNSNL
IGANLGYYYSGDRCKDCAIDPNTNYSKDGLTAQKYMYMDLDGAISKEQIP
DFLKKVSAINIDVINFYNSESKTKEKYKNAFLELESFLSTLYVENKSYEE
KYHFDNSRVEFFIFKDMQENAQNIIN NMIESDRIKFVESIINKMIPPPDG
TILTENPKEYVQKQIKESHKESATNVTFDYKELAPIFEGVEEKQIKALSN
QLEHIKSFKTDYDSRLNKYARDFYYFSSAFAINWEDLLKNYQEIRASIKD
YDDLLREIKELIISRNKSLEDKRTLFDNKRDNWNSIEVQDEVNALNAKIV
DCDDKIRSKLTIVRNNRLENQYKDDKNISDAMRNILDWFERYPDIVQNIT

QA
> gi|16766251|ref|NP|461 866.1| secreted effector protein [Salmonella typhimurium LT2]
MPVTLSFGNHQNYTLNESRLAHLLSADKEKAIHMGGWDKVQDHFRAEKKD
HALEVLHSIIHGQGRGEPGEMEVNVEDINKIYAFKRLQHLACPAHQDLFT
IKMDASQTQFLLMVGDTVISQSNIKDILNISDDAVIESMSREERQLFLQI
CEVIGSKMTWHPELLQESISTLRKEVTGNAQIKTAVYEMMR PAEAPDHPL
VEWQDSLTADEKSMLACINAGNFEPTTQFCKIGYQEVQGEVAFSMMHPCI
SYLLHSYSPFSEFKPTNSGFLKKLNQDYNDYHAKKMFIDVILEKLYLTHE
RSLHIGKDGCSRNILLT
> gi|29171488|ref|NP|808672.1| type III effector HopX1 [Pseudomonas syringae pv. tomato str. DC3000]
MKIHNAGLTPPLPGISNGNVGKAAQ SSITQPQSQQGSYGLPPESSETRPD
RARANYPYSSVQTRLPPVASAGKPLPDTPSSLPGYLLLRRLDHRPVDQEG
TKSLIPADKAVAEARRALPFGRGNIDVDAQLSNLESGARTLAARCLRKDA
EAAGHEPMPANEPMNWHVLVAMSGQVFGAGNCGEHARIASFAYGALAQEN
GRSEYENIYLAASTEEDHVWAETDESQSGTSTIVMDPWSNGSAIFAEDSR
FAKNRNAVERTDTFNLSTAAEAGKIT RETAEKALTQVTTRLQKRLADQQE
QVSPIKSGRYRPEKSVLDDAFVRRVSDKLTSPDLRRALQVDIEAVGVAMS
LGTKGVKDATRQARPLVELAVKVASPQGLARRDV
> Q7ADG2_ECO57
MSQNDIIIRTHYKSPHRMHIDSDIPTPSSEPINQFAPQLITLLDTSDLSS
MLSYCVTQEFTANCRKISQNCYSTALFTINFATSPIHAENIFITLHYKKE
IISLLLETTPIKANHLRSILDYIEQEQL TAENRNHCMKLSKKIHREKTIQ
PTVNLNGSAFFSQSPSDAIFCRHLSLQYALDSLRNGKGKVNLIKHYSSVE
SIQQHVPLVRDAEFRALLRHPPAGSRVIASKDFGFALDIFFCRMMANNVS
HMSAILYIDNHTLSVRLRIKQSAYGQLNYVVSVYDPNDTNVAVRGTHRTA
RGFLSLDKFISSGPDAQTWADRYVRNCAIAILPLLPEGVPGAIFTGIATR
MPFAPIHPSAMLLIMATGQTQQLITLFRQ LPILPEKEIIEIITAQNSIGT
PALFLAMMNGHTDNVKIFMQEIQSLVDNHIIHEDNLVKLLQTKSANETPG
LYISMLYGFDEIIDIFLNALTTPIAQELLNKKLVMSILAMKIHDGEPGLY
AAMENNHPLCVTRFLSKINGIAFKYKLSKANIMDLLKGATAQGTPALYIA
MSKGNEDVVLSYISTLGAFAKKHSFSQHQLFTLLAAKNHDNMSAVHIAIH
HNHYKTVETYYAAINVISQSMSFSADELKT YL
> Q8XXI4_RALSO
MKATRRASLFGWSLARVGTLGKRRRSRNALEMSTEIKGVRKMRPRSAAEP
DVSTPFRHDTQAGRVAPACSAHGAQRALVPTGEQLVECERALLHCAEFQQ
LRAQHVSRLTKLAEDSRLRKQVSVHECVDLACANVLAKRNGYGLALVFLA
AGATPEENVRQLTNYFFALQAASLRASQPERRQLGQCADLAAHYLFKTPW
FARAPLRPFVMLGNLLSKYPDHPACLQALSWIAD QVLAPAQMPALGEKDL
ALLANALSKNSGCGRSEQAVARIGRHVVRAGAGDFRAQSVSLMLNACSKW
SGNADCRDAVEVLAARVSEDAALQRDMAAQAVANALNALSKWPERIGCRN
AVLALAVRVATDVALRCRMKPQEVANALNALSKWPDRIECRNAVLSLATH
VGEHGTLRQAMNAQHVANALNALSKWPEQGECCDAALPLIRRVADDAALR
SSMDALVTSNVLNALSKWPDRVECRDTAVTLALRV ADDAGLRHDMGVQAV
ANALNALSKWSEQAGCRDAALSLAARMVGDASLRHAMSAQNVANTLSALS
KWPEQAGCRDAVLSLAARVADDAALRLDMNAQNVANVLNALSKWPERGEC
RDAMLSLAPRVADDAALRYDMSAQAAANALNALSKWPAQTGCRDAALRLA
ARVAEDAALRHSMDAQQVANTLNALSKWPEASSCRHVTLLLMRSLGESGC
PWRQFNMINLAQIANASARLFLSASDEVEIQALART KLRALAAHLGLHRE
RFETASASSIGTIFKAMEALQLPAEMRSLVCAAMNRIERLCSQASLRSES
RESLGMLRAGLLPYIDTNRGLSDSPGMLEAAAPRLTAWAARHRLEIDL
> gi|28868095|ref|NP|790714.1| candidate type III effector Hop protein [Pseudomonas syringae pv. tomato str. DC3000]
MDKNKPNTILIVSSELDVSEVVSR IRSLYPIDAWTFIYLLEDLLGQAGEA
LNRDGHFVIRDFAVHTEVEKVFGQIARKYSVTAVIPNDEFAVYIAAWAND
CWQLPGLTFDVATRFRDKRRMKKIAQKFNIPTAKELSLDDINRGGATFPV
ILKPRSLAGSVGVRIITEVQQLSEAYATEIDDYQDMNEHQYLIETYNSQP
LYQIDAVVLKGQLAFLSAGKYLGKPIDYLAECPLGYFSLAEDDLHTLWRP
FIERVLSAFEGPDGVYHIEAFGDAG DGFELLEIAYRPGGASTVDMIKIAY
GIDLRFIHLAVQLGLVDALHVDRKGEAFGYMTFPKKHLAKEMLYVTRVSL
SSMETLQTLKMKKIAQVGDIASGEFFCHKDCLGAFVFCGDRGSVAHDLKH
VEEHYQVHAEAARG

> Q4ZT52_PSEU2
MTTLTTRQIQLAHAWTSVHTGAGLALDWVADVAEKVEEVAIKADALSRDL
HRARNLSRSLGRVSTTPMGIGFFGLSQAGKSYLISALAADEKGQLLT RLG
TRQLDFIKHVNPVGGGKEATGLVTRFTRTAAPSLDPQFPVELRLFREVEI
AIILANAWFEDFDHQRLNSQVTDAQIDALLQRFDGQSAAAPTPGVSSDDV
VLLWDYLEHHYANAMRPLNARYWPCVIKLAPRLSVRERAQLFEPLWGGIG
KMTETYEQLASALHRLGLAETVFAPISALVTERDGQLVQSNSIINVDILS
RLGGSADSPIEVRPAHEGTLRSAVSVNRAELAALTNELIFRLDNEPAN AI
VNSVDLLDFPGYRSRQKLMSINEASEVDSNGTANNPVARLLLRGKVAYLF
ERYTNEQEMNALVMCTSTFKQSEVVSVGPVLKSWIDKTQGTSPQQRDGRA
SGLIWALTMCDGFIGGALNGETVQFPEGCDNMLKLTMIERFGNEDWMKQW
GSTPFKNTYLVRKPRFKTSFIELAADGEERAYSDSSHTALQALQQAFSNS
ELVKRHVAEPQEAWQAMLTLNDGGMTRFSSAFSPIANIDFKLQRIAEQL D
ELMVQLLPRLEEYYEAGGEDERAKKKVIANMIARPFATTPHGKHVLGELL
GYMALPEQQLRDLYLNGDFGSPASEATAAVQAVGKPEVEYDIFGEAIAAT
ATVETPAAAAVAPQYQSHEHRFARAAFDLWATHLRNLSRRQHLLDLLELP
AEAIALLVKELVVCAERLDLPLQLSNALLKRAQSGVRRENLVQRQVLTAQ
LLLNDFAAWFGHTAQPAGQRPTGLLGAKQPLFAFYQKEMPGRFPHLAAQA
DDQSVIFADDWISGIAIHTQKNVGHRKGKEITPEQNEAMGRVIQAFKAR
> gi|12620564|gb|AAG60840.1|AF322012|145 ID322 [Bradyrhizobium japonicum]
MATGFTSQRWRELFPGRTDVTSIVDFQVAHPLVGNAGDILLEHQLGRDGE
RPLVNWRPANDEARARAEQLGFVHVDPDDMVLDPTQSKQWRYRDGEWQRA
TNSSMYLSKASSDDEPSSEPDSDGDFM
> hopAT1 Pph race 6 -1448A|Genbank: AY803994
MVGINNWSSNLDAYRILQEAQSLTEEPPHFDTAFPIEPESIAPPEHHNHR
YVIGQEEHRVRRLAANRNKGRPSARAFGNARNNSTRN
> HopAB3-2 Pma ES4326|Genbank: AF458391
MVGSINRAGPSGAPYGHTAPEPAPERAHESGSGASFSNSPQLPAHSSDAP
TSQARDRREMLLRARPLSRQTREWVAQGMPPTA DAGVPIRLHERAATAVP
DARAEERHTPEEPADAAAPQHLGARAHANGIVQQLVDAHADLAGMLSMIQ
DGRRG
> gi|15599036|ref|NP|252530.1| exoenzyme S [Pseudomonas aeruginosa PAO1]
MHIQSLQQSPSFAVELHQAASGRLGQIEARQVATPSEAQQLAQRQDAPKG
EGLLARLGAALVRPFVAIMDWLGKLLGSHARTGPQPSQDAQPAVMSSAVV
FKQMVLQQALPMTLKGLDKASELATLTPEGLAREHSRLASGDGALRSLST
ALAGIRAGSQVEESRIQAGRLLERSIGGIALQQWGTTGGAASQLVLDASP
ELRREITDQLHQVMSEVALLRQAVESEVSRVSADKALADGLVKRFGADAE
KYLGRQPGGIHSDAEVMALGLYTGIHYADLNRALRQGQELDAGQKLIDQG
MSAAFEKSGQAEQVVKTFRGTRGGDAFNAVEEGKVGHDDGYLSTSLNPGV
ARSFGQGTISTVFGRSGIDVSGISNYKNEKEILYNKETDMRVLLSASDEQ
GVTRRVLEEAALGEQSGHSQGLLDALDLASKPERSGEVQEQDVRLRMRGL
DLA
> SopD2_Stym
MPVTLSFGNRHNYEINHSRLARLMSPDKEEALYMGVWDRFKDCFRTHKKQ
EVLEVLYTLIHGCERENQAELNVDITGMEKIHAFTQLKEYANPSQQDRFV
MRFDMNQTQVLFEIDGKVIDKCNLHRLLNVSENCIFKVMEED EEELFLKI
CIKYGEKISRYPELLEGFANKLKDAVNEDDDVKDEVYKLMRSGEDRKMEC
VEWNGTLTEEEKNKLRCLQMGSFNITTQFFKIGYWELEGEVLFDMVHPTL
SYLLQAYKPSLSSDLIETNTMLFSDVLNKDYDDYQNNKREIDAILRRIYR
SHNNTLFISEKSSCRNMLI
> Q8XQB2_RALSO
MTRITHASSHDYPRSHDNQAPASSTHPRKRNRDPAHDASSSFDGLPRRTD
SRRHPEHV PAPKRQRVSVADRQETASHASAPHPQHRARTTAEAPRRQRSD
REPPARQAKRARHDRSDAIRTETGRARTAPAPAQARDRHDPSDARRRAPA
AVPQRPTPEARASFRKQFARTRKLDDDESDLLWTIASDALSHRDCGKALR
LLERPAVDFMDSLACFANFVRAVSALLESAGRLGLSERATDQLKRKWEES
SHAFYAVHHDMAIDNERFVSKLASLCNRWSQAQPAQGAGCVAVARILESL
GMRYGPGLLRNRLADTNPLLGRDMSLLINAFSKWPEDEACRVGAQLLAGV
VADEQDALLSPVQYQPQALARLVGGVSKWPDDKTCRQAAAAMAGALWQRR
QELDNPEAFKPQELAMCLNGLSKFTDAACEQAAMAVAGAVLSRKAQLLDP
GTCPSQHLSMILNGLSKWPLREPARLAAVEVAAALWHRRRDMTDTGQFNA
QDVSNCLNGLSKWHGDATCRQAAVALAEALPRHRDAHNDLPDFLPMHLAS
CLNGLSKWPEEASCRQAAAAVARALLWRKADLDDAERFSAQHLSNCLHGL

SKWPDEDSCRQAALAVADALVRRLGTPGGADAFTPQQVSNCINGLSKWPE
EPACKLAAVAMATALWRRRDALDDAAQFTGQGLANCLHGLSKWPTEDPCR
KAALAVVGALMPRWSSVAVAGDPFLPQLLSNCLNGLSKWPEEDSCRKAAL
TVAETLMRHRRALADPRQFTPQELSNCINGLSKWPEAPGCREAALAVAEA
LLLRRDELGDTEAFGAIGLAACLNGLSKWPGEATCRRAALAVAEALLLGW
DTLAADGQLTPQRLANSLNGVSRWLQDAASQEVSIRIIGMLGSGGQPFGA
FTMAQLAQLANGTARFILELGHAGEADDGDAIQSDASMALAQARLRELAA
HLDSRPDRLAQAETRNVALILKALASTGLKDGLRLLAHQGLTRLAALYAE
TGFKPDNLEALASVAAGLLPVARAPELKAYRADALRLLDQMQPAVDRKIR
LYIDAHLAAADPRRPAAAEAPPSLQIRRDGEAFGTRRPGLTFFLLLKAYS
VVADCWKRSNVPDIPLPAHARRDALRTWVAEMLDRTRGAIETDLDEMSWN
LIAQIEAADQVLDAVDLKLHKDLDTILAAHPPTPLNVGDVRRELRALPEV
RDMMHSDAGAAAIEVIDLAGRNLTPHRPDETRDTAASQYAFFTRLTSGRL
PLLEVQLPGKVSAFMLTRVIRRGGDLLRMDLFGGSHLKPTKVHMRSLLGA
SGTKGGIKRYGRIPAMRLADTAPDAALMKDVIRKLNPQREDWFRMQRALL
EVVPRDHVVEGPIRLALLPDRLPGSPPAFALRTPEGAAIQLVPNDGCGFI
RESLACRIPVIRQAMHSAQALEGESQRMSLLPPQATHHFPRDQATIEEAR
AHLRASLRDDPELWVANPDDGTRDIKTHKLYEALAGALIMGTQGIAVPSG
DDRLHLPRDKSKVFDATGGTVLLGKPPYDKANLMPVTADRIGTCDNGDAT
ARFLDTAFAFQYSYTAWDETGNPPAAADDVPMLHGKGVTIVVPDKMWPQD
NDAQWVWSTEDMKVHSSWTDGRRRDRLAPRMDTVGSLRVKEIFPPGALMA
LPINELKKRDADCDGDKVFLYAGLPRMTQAVTRFFEERERRIGKLGSFKP
PKTAHGVLDAEGRYQAGRAAEVLSALHGQELVGRLSTLQFLFFGQPPALQ
QTLAERTLFGVYEGTERELRRGVRALLRGERATTPQVMEDLLLRAGLGVQ
HACHPVALEVARALEAQLLEVMREAQPPVRAVSPDTASRAPQALSGGSTL
LFAPLTQAYESATTPRERLAALVTHYPRARLPHPATILPTTPPKQPDPLA
QPAPLDDAPIGQSGYVPGAPLETLHNLLTLGVKIGTDAPKAVTQTDLFLD
IADHLEGTLRSSPERIRFMPYTRSGVMQELSQGRFNAQATLQRLRDNPTL
AAGLMEMAIEELLAHGAIEGTPPLRTTPAETEAQLRHCARTLHAAARLVE
PEITPVIKAAIEGIGALRGQAHRVKTERALHDKLRVLLHRGHPSPADAAA
MVNDALRYSVVLKPDDFAAGYAEVLGNLDRAGLIKTRVHNAFKPAWDPFK
GINVKFMGRDAAGQSVRLEVQFHTDETFALKMRYHDNYKQDFELQMQGAS
VEQRVACLEEARQACRQVATPAGCEHIGDWNNEPPQVNKRRAQAKAAQLP
GAGAGKRPDAMATQVERLLQAAVRIEREVGPLLQTMQLHVIREHSVVKKA
KSIEKKIQRLCVLNGITPEQAADRVRDAMRWIIQLPHETFGAHAQKALET
LTANGLRITRVNNSFMARNRTYAGLNVKLQTREALNFEIQFHTGDSLYTR
NKTHKIYRQWQDTDVEQRQATDPAQRQALQKANADRLAALKTYAATVPTP
IGAERIPSFDRYRRDGASASDRARAERTAAPES
> gi|15835481|ref|NP|297240.1| hypothetical protein TC0867 [Chlamydia muridarum Nigg]
MSLSSSSSSDSNNLRSVLSQVITSTPQGVPNADKLTDNQVKQVQQTRQNR
DDLNMESDSAIAGAAGKDRASSVSQMEGQEVIEQQGLAAGKETASASTAS
LTQNASTGAASQKRVEDTNKSLELSSLSSLSSVDASQLQEIQNIVASATR
STNETSLKNLDTPGLPKPTTTPRQDVMEISLALAKAITALGESTQAALEN
FQSTQTQASNMNKMSLESQGLKIDKEREEFQKLQEIQKKAGNNSTMDTVN
KVMIGVTVAITVVSVVAALFTCGLGLIGTAAAGATAAAAGATAGATAAAT
TATSVATTVATQVTMQAVMQAVKQAIIQGVKQAIVQAIKQGLKKGIMQAI
KQAIKAAVKTLTKNIGKIFNTGKNAVSKSFPNLSKVMNTLGSKWVTLGVG
ALTAVPQLVSGIGNLQLSDMQKDLAKIQKEVGALTAQSEMMKAFTLFWQQ
ASKIAAKQTESPSETQQQAAKTGAQIAKALSAISGALAAAA
> gi|34498030|ref|NP|902245.1| secretion system effector [Chromobacterium violaceum ATCC 12472]
MDINKVGVSNTVPTVPQQLSGQSVDKGSTGVYATIDNLIAMLQKMNIDIR
DMERDFHAAGQQRAAAVQLKSIDTKLESIELKYKAAWKNASAKVLSGFLS
AGGAAIGGVAGSGGDLMSTGLSGVGKAAEGGINWGTASLTRDAEQKNILG
EFQADHAAERYKALSAAAEKAAEASSRMHDLTRQLMSMQERMMSVVKI
> gi|29839805|ref|NP|828911.1| hypothetical protein CCA00037 [Chlamydophila caviae GPIC]
MELNKTSESLYNCKTDRHSVQQEVGPEPKDNRDVKVFSLEGRQQSKQDRQ
DKVSSKDSRQESRGADDKHVEEKTSAVSSKEEDKEESDGFMAYDNPTAGM
AFVDVAASVSSEAVVESTTVAIGSADLQWVQDVIASTVESMMVADVNGQQ
LIELVLDAEGNVPDIFAGANLTLVQTGTDLSVKFSNFVDNAQMAEAMSLI
VNNPSQLAGLVEALKNRHLNLTELAVGSSIVQLPTIEEVQTPLHMIAATI

HQRDEERDQEGQDQQQQQDQEQNQYKVEEARL
> gi|140232|sp|P19520|YAV5_XANCV Hypothetical 50 kDa avirulence protein in avrBs1 region
MSDMKVNFSSKIIDS TPSEEEVATQQDSYTKSGLVAPSLDSQALKKAPRK
RVIKENIAALHTSSLERVHQKKVLVQNLAQLQRGLAKINGRVELEELIDG
FSVKELLIKRNPKIAEEYGEGNPLMIRSLRFSNPQEVTSKLGAEGKTPAK
REVDTICNKSTLHDIVMTPASLVKKEVRMNLISEVPRAKDKQKYRGLPSV
VYGQSSRRSESDYLTSRNGFGDVHSLKSNNAFNSDYEKICGSLSHAEKLG
LIERNLTPFIRHDPDR ISTDFVHSIEELAEHQMLLQSRKPASALRHNEYC
TKLELWDAKAIAVGESRALAVATLIEFNLEMLSIAQEIDDDGHKSKMVAD
FIERQLSWLGPQTALDSKSTLERVSAVTIQEREFIANEISRSLRQGVSLC
TYDKDEAGSHIREMSLLDFRVEEIIEGISIFISSKLLHVTNAGEA
> A1JUC5_YERE8
MIGPISQINSPGGLSEKETSSLISNEELKNIITQLETDIADGSWFHKNYS
RTDVKVMPALVTQANNKYPEMNLNFVTSPLDLSIEIKNVIENGVGSSRFI
INMGEDGIHFSVIDYKHINGKTSLILFEPANFNSMGPAMLAIRAKTAIER
YQLPDCHFSMVEMDIQRSSSECGIFSLALAKKLYTERDNLLKIHEDNIKG
ILSDGENPLPHDKLDPYLPVTFYKHTQGKKRLNEYLNTNPQGVGTVVNKK
NETIFNRFDNNKSIIDGKELSVSVHKKRIAEYKTLLKV
> A0VTH1_9RHOB
MATVLGCIADDFTGATDLAGLLARSGAAVRLRMGVPDGPPQDTAGIEVIA
LKIRTAPVAEAVAQARAALAWLRAAGAERVFWKYCSTFDSTPQGNIGPVA
EALMADLGVQQTIYCPAFPENGRAVFMGNLFVGRDPLDESPMKDHPLTPM
RDANLMRLLAPQVTRPVGLVDRLCVARGAGALRAELGRLDAAGVAHVVVD
AVADADLGVIAEACHDMPLMTGGSAVAAPLPGLLSGGAAEARAAAPDLAP
GAVVLS GSCSAMTRAQVSAYLRRAPGYKLDPLVLRTEGAGAALAWLEAQA
LQDAPLVYATAEPGEVRAAQQALGVAEAGALVEDALARIAVAARDRGARR
FVVAGGETSGAVTQALGVVQLDVRREIAPGVPWCFAESGGVDIALTLKSG
NFGAESFFADALALVDTL
> Q8X837_ECO57
MLSPIRTTFHNSVNIVQSSPCQTVSFAGKEYELKVIDEKTPILFQWFEPN
PERYKKDEVPIVNTKQHPYLDNVT NAARIESDRMIGIFVDGDFSVNQKTA
FSKLERDFENVMIIYREDVDFSMYDRKLSDIYHDIICEQRLRTEDKRDEY
LLNLLEKELREISKAQDSLISMYAKKRNHAWFDFFRNLALLKAGEIFRCT
YNTKNHGISFGEGCIYLDMDMILTGKLGTIYAPDGISMHVDRRNDSVNIE
NSAIIVNRSNHPALLEGLSFMHSKVDAHPYYDGLGKGVKKYFNFTPLHNY
NHFCDFIEFNHPNIIMNTSQYTCSS W
> gi|16764446|ref|NP|460061.1| secreted effector protein [Salmonella typhimurium LT2]
MPITNASPENILRYLHAAGTGTKEAMKSATSPRGILEWFVNFFTCGGVRR
SNERWFREVIGKLTTSLLYVNKNAFFDGNKIFLEDVNGCTICLSCGAASE
NTDPMVIIEVNKNGKTVTDKVDSERFWNVCRMLKLMSKHNIQQPDSLITE
DGFLNLRGVNLAHKD FQGEDLSKIDASNADFRETTLSNVNLVGANLCCAN
LHAVNLMGSNMTKANLTHADLTCANMSGVNLTAAILFGSDLTDTKLNGAK
LDKIALTLAKALTGADLTGSQHTPTPLPDYNDRTLFPHPIF
> OspE1_Sflex
MLTQTIFPCLPQKQENIILEVSNPVLLSSTVTTDGYTVFNKKAAIYELQI
PAASRTKTLKFTATEMQWLTKINEAGIDEKQSQRYSDF
> Q8XRC8_RALSO
MGLARLPS GSHRWHAVSPESGNDPSQHSHSNQQTPSLTRRARASGHLEPL
ARSPGHRAHASARALAVEAAGTDRSSTQGSGSGMACAGPREDGADPLERV
SHSRQASRIPLKRKREAGDDTDVRPTSLPRRGEIAAFHTAACDADAPTTP
PTTPRAESRREPTAEQLTDYGRWLGHTEFGRLAALREQQCARLWKLVASA
RRGKVDPDFCLHVARDNTLAQRNGYGLALMFLDGSVPPEENARRLDGYLF
ALGAASAPT HAEPHRQVRGLEQCVEMAACYLFKTRWFMDAPLEKLAELGN
LLSKYPRQPASMAAIAWIAGQALKPGPLPRLGAKELTLLANAFSKNGDSG
RCEQATARIGRHLLHDGGEQTFEARQIGLLLNAFSKWPGDTDCRAAAEYL
AARLERSPGLRQAMDAQAVATAINGLSKWPETAVCRNVAEHLAARLVHEA
ALLERMTATAVAASLNGLSKWPKAEICGAAVECVAEQLVKAPGLRQAMSA
QEIANAFNAL SKWPDSAACRAAAECLAVRLADDPRLQASMNAQEVASTLN
ALCKWPRTTACREAALGLAARIRSEADLRDALDAQQLANTLNALGKWPDA
DVCRAAAERLAIRLGRDAALRDGLNGQHIGNVFNALGKWPESAACLAAAA
CLTARLTDEVALLLSMDAQGVAAALNGLSKWPESAACRTAAERLIARLSS

DADLRQGMDAQAVANVLNALSKWSREDHCRALAEQLAMRLVDDAELRQAM
NAQEVANAING LSRWLEQPHCRRSLLLLTARMGEADLPWRNAEMSALAQA
ANAFARLFLSAPDDAELQAPARIKLQALAAHLDLYRERFETCSAREIAIV
LKAMASVQLQHDMRPLARPALERLAALCPTTGLRNENLETMGTLCLGLLP
LARSRVLIAYRRRALCVLGTLQPIVARKIERFVQPRLGTAEKALAADTAM
REESHATRRAALTFYQVLKTYSVVSDMWKTRYIEGSRRSVRDQRDRLAAW
VKQTLERSREAI EADLGEDSWNIIARIEADADVLNALDLRIQHQTALITQ
NHPPSRFDLGAAHRRMRTLKPGAPVPPARGAGDTHYVVVDMAGKELKHNR
DEPSKPYSLYARLTGLPLVEVKLPGELSAFMLARTFRYQGEPWRFDLFGG
SRLSRGGGLRPHDILSNHAAPAAMLPAVRYADTAPGSSLMQLAAKLAPLR
EDWARMQRALLEMVPSDHVVEGTLRLGFFDDVDGPEHPFKLQSPDGQRIQ
LCPNDGCGFLKFE VAMCIPVFRDHVMAWNAMRAGRATPAQQKLLETSNDR
KRIAPQALQHFPRDEAALEEARRAIERKLAQLAPKPASGQAPSRVDPLTL
YHLTVSGGYEGEQIRAVPSADDNVHLPAQRSGAFDLHGGALLIGKAPYDK
ENLLPFPDEQVGTVERGDATASFLSRCFAIQYSFTGFNDDAGEGAEMLHS
KGMLIVPPPQYWSPDHAGMDMACSREDLKTLSRWITGRNRATLPPEMRST
GSLRVKEVVVPGRL GALPITELRKRDMDTDGDDAFVYAGYPKLAAHISRV
MDDRKKRRGQQRSFKPRKTAAPAQDPKDGQYQPGRAAEILAEQRGRRLRG
TASVFAKRFLAQSDDMREAMARDMMFGVYDGIERPLRNGLLEQFEAEQAD
PQALDALRNHAFEDIANTHLPEAEEAAQLLHDEVFRLQSVPAAKAAGMPA
GLADRFPLLAQAYEQAGDTRARVQAILENYPVCRLSCEQFPEGQPGLVRG
SPELTLRNLCTIAIK VGTDALKSDTGTELFSKVIETCLRSERTFRERIGS
VPYGKETAYAMHDGRFDPEQAKAELRHNPTMAAGVMIHSVEALQQWGLLA
PAPTPQARFANTPALDVSQAIRLLADRASRMDAAITPTLRNIAQAAGAQL
AGLRHRLKSSGSLKEKLKQMVAHKHMTLEDAVPQVNDALRYSVVLPSQDF
AAGCRRIQAALDEQGHARVKLVNHFVKRYEPFSAINVTLRDPEGHLWEIQ
FHTPQTFDLKEHYHDL YKRSHHLRLQGVPAARLQELTRPARDAFRAVPMP
PGCEDIIDWEAEQTSVALPAVRAQLVPEPVYAELVNRLHQAAKAMEPEIT
PVLRALLQHVQGHLHGDGPELHRHVFKKPASTHRKIELLRHQHALPPEQA
AARVRDALRYEVVLQHEGFVASVQWVSRQLQSEGLEVMRINNTFATADTT
YAGLNMNLRSGAHHFEIQFHTPDSLRIKQKTHRLYEKLRRIARPEAMPPH
NGQNPPASERESLEHGL RSAAATVRRPEGIEAIGSIDRYEG
> gi|10567542|gb|AAG18480.1| avirulence protein [Xanthomonas campestris pv. vesicatoria]
MTSSINRYSYNPTNFPQFEQLSASQNQAERSHPSAPLKDRSHEEAFPVRL
ASTRIKRSSGAGREFALSRNKPIKGVGYSTQPAVTASTGTSKPGVLCMTD
GMDLCVGVAVGGEKPSQNAGKARVFHVMPENRRAQWEIKS YIESLRSQGY
AAKAAIHGGDSSSRSSVSKIQAIEATLGAMDVPIEFSSTGAGANNGNGPL
GAVVEENGTVRFVTNLVK
> A0GFG8_9BURK
MGWHRASDLRRRALHRSAVHVGRRHSRASGWARRRRDERAPGMGGGAGGH
AVAGVRRAYAGVATRLGVFRQPRVNASGDTEQEMSDEANSRGPAYAFYGD
DFTGATDTLAHLARAGLRTMLFFAPPDATRLSMLGPLDAIGVAGAARTMP
PAAQRQE LERVGAAFAALGVRVMHYKVCSTFDSAPETGSIGVAIRTLREY
CANELVAVVGGQPNLRRYCVFGELFAAAGADDDAQSIYRIDRHPTMSRHP
VTPMNEADLRVHLQRQGVKNVQSIDWRCYAHGDAELQEEVQRRLDAEPDA
VLFDVLDDSHLQAIGRVIARHAAASAPLLAVGASSVAQAYALAHDNRVKD
AAAENRTTPLLPRAHGPVFVLAGSLSPLTEVQIGAAQSYLRVELDPLKMT
GDAASSYLAERVAAIAGPLRDGRNVLAFTARRASSEGGALPQLAHACASL
LQQVLAAVRLRRIGIAGGDTSSFAVRALGAWGLSYLAPLSAGVTVCRLHA
DRAELDGMEIMLKGGQMGDANLFEQLLDGNG
> Q7DB76_ECO57
MFSPMTMAGRSLVQATAQTLKPAVTRAAMQAGTGATGMRFMPVQSNFVIN
HGKLTNQLLQAVAKQTRNGDTQQWFQQEQTTYISRTVNRTLDDYCRSNNS
VISKETKGHIFRA VENALQQPLDMNGAQSSIGHFLQSNKYFNQKVDEQCG
KRVDPITRFNTQTKMIEQVSQEIFERNFSGFKVSEIKAITQNAILEHVQD
TRL
> gi|27377169|ref|NP|768698.1| hypothetical protein blr2058 [Bradyrhizobium japonicum USDA 110]
MYNRVDGEYAHTEQAEESSWPADGSECAQTLTEIARLESLAPGELFDRMG
LCFSKPHTSDAIDDSS NTSGLSTSSLSSSSELSVATSPVRPLFDYRTAEL
PQANVSGICVGLAAEWLLDLPSSASSRMGVLLPGTENHRSAARRQEQSEK
LKTQLKEDKAEGSHNFQAKSTILRDAGLEPSAEETRYRFGTSSCIDKIVN

ELAQDPSVHLVSLKFVQPGAGTHTIATATSNGTTILSDPNYGEFTVPSDR
VGGLFKSLAERYSTLNKRDISAVVTQRIRYGHPNATDLALFPRAEPHR
> A0UL57_9BURK
MSMLIIADDLSGAADCAIGFASAGYRTVVTLDVPTQGLDATVADVIAADT
DTRRLTPAEAAQRTSAAWRALREPGRRLYKKIDSTLRGNWAAEVAALQPL
AGLAIVAPAFPATGRTVLGGRVFVRGEPLETTDTWKLENAGRTADLHAML
AQNGVSTAQLDVDALRESSQTLIARIAAVAASGTEALVVDAETNDDLIAL
ARATAQMDDALFWVGSGGLAREIASVESLFDARAARTGAAASQRQPRHAP
VLALVGSLSAVSERQCAMLRERAGMMELTVPPAVLRGGERHADVPQWQAR
IGDALRTGADLLLRIGSDDAFDPAEGALLSATLAALVKPHFAVLGGLIAT
GGETARAMLSAVGVGSVELIEEIEAGVAVGKPIGASVCQPHLRIVTKAGA
FGTDHALFAAWRYLRETPAASVRAGARSHGSA
> Q8XZC7_RALSO
MDTHDLAPPPNACAADGAQLAGGNLDTLMQRAHELTHAAARALGLDEPAA
AQAADDGGVVLNGTLVTVTPMPLEGLAGEAGDGALVVSATLGCRVGALDA
RALCALFAHAPGVLAVYNAAIGCQPNGELVLHRMVPVHDAVVDTLAQDLV
VTQQLAVLLGDAATQVRTPQ
> gi|21241166|ref|NP|640748.1| HpaF protein [Xanthomonas axonopodis pv. citri str. 306]
MFNINRLLRSRPRTDTADQAPAEDRPSHQPSAAEGLLSPLLARPRRRESA
AAPRNAGQRSPFRSPLRASGQPAALAGASSAPPRSIEHDIDDWLAQSLPM
AERVRTGYNLHDGQRDTSREVLQSVADAIRRAATRHSTELVLDYGLPATT
LPDAIGRLDALQKLTLLHTGLQSLPDSLGQLHQLRHLQIAGALGLKTLPP
SLTRLSNLRTLQLTMIPLDELPVGIGRMQGLRSLTLGGGHYARLPASIVE
LSGLTELRMPHSSHFRELPENIGLMQGLRSLEVASNSELEQLPGSLTQLH
RLEKLTLSSNRRLAHLPEDIGQLRGLTELSLKNCAALRQLPDSVGDLAQL
QLLDLRGTGLQTLPQSLARLPAQCDIKVPDHLAGQLLQIRDPERAAREPQ
RAAQRLAERRRRAPVPAAQQAGASWNRMPEFARVLRTVDADLGERFDKWT
QGLAQTARISGASITPADMPLLDQVVTEAIRSPEFRSSFGQFLSDHTLKT
LNMDGMTQVGGFGPAVRGDVKTAFAEMLKHKLMHTQDHQTALGLLQDALQ
NPDLGLSREMLLRSRNELTGRVEMWPPLKAYISMHDVEGQAAQDAAITWT
MAQFEEAQQGGIGDAEAKQESERAQANANRFIEQRARVLLREWDIR
> Q221V6_RHOFD
MKKILLGCIADDFTGATDLANNLVRAGLRVVQAMGVPAQPLDADADAVVV
ALKSRTLPVDEAIAQSLAALRWLQAQGAQQIYFKYCSTFDSTAQGNIGPV
AEALMDALGTDFTIATPAFPDNQRTVFKGHLFVGDVLLNESGMQNH PLTP
MTDANLVRVLQGQCRRPVGLIDYKVVAQGEAAIRTRIDQLRAQGVGLAVV
DAISNDDLLRLGAALSDMPLVTGGSGVAIGLPANFGIAPSSTASALPRAT
GLQAVVSGSCSLATNRQVLAFIQAGRPALGIDALQIAAGVDVTTQALAWA
APLLSQGPVLIYSTADANAVKAVQAQLGVEEAGAMVERTLAAIALGLVAL
GVRQLVVAGGETSGACVQALNITQMKIGPQIDPGVPWCHAQADAAPG AGL
HLTLKSGNFGADDFFSKAFALLA
> Q4ZX48_PSEU2
MRIHSSGHGISGPVSSAETVEKAVQSSAQAQNEASHSGPSEHPESRSCQA
RPNYPYSSVKTRLPPVASAGQSLSETPSSLPGYLLLRRLDRRPLDQDAIK
GLIPADEAVGEARRALPFGRGNIDVDAQRSNLESGARTLAARRLRKDAET
AGHEPMPENEDMNWHVLVAMSGQVFGAGNCGEHARIASFAYGASAQEKGR
AGDENIHLAAQSGEDHVWAETDDSSAGSSPIVMDPWSNGPAVFAEDSRFA
KDRRAVERTDSFTLSTAAKAGKITRETAEKALTQATSRLQQRLADQQAQV
SPVEGGRYRQENSVLDDAFARRVSDMLNNADPRRALQVEIEASGVAMSLG
AQGVKTVVRQAPKVVRQARGVASAKGMSPRAT
> gi|15723919|gb|AAL06366.1|AF311901|18 SepZ [Citrobacter rodentium]
MEPTNLSPSGVVLPLATAINGNGPVDENTGVMQAEGGASRNVRVFAGVAL
AVSALAATGAGIAAMCIDNSSQTEKLGLGIAAGVLGGLTSVGGALAMKYA

> Q8XBX8_ECO57
MLSSLNVLQSSFRGKTALSNSTLLQKVSFAGKEYPLEPIDEKTPILFQWF
EARPERYEKGEVPILNTKEHPYLSNIINAAKIENERIIGVLVDGNFTYEQ
KKEFLSLENEYQNIKIIYRADVDFSMYDKKLSDIYLENIHKQESYP ASER
DNYLLGLLREELKNIPEGKDSLIESYAEKREHTWFDFFRNLAMLKAGSLF
TETGKTGCHNISPCSGCIYLDADMIITDKLGVLYAPDGIAVHVDCNDEIK
SLENGAIVVNRSNHPALLAGLDIMKSKVDAHPYYDGLGKGIKRHFNYSSL
HDYNAFCDFIEFKHENIIPNTSMYTCSSW

> gi|28871725|ref|NP|794344.1| candidate type III effector HolPtoU2 [Pseudomonas syringae pv. tomato str. DC3000]
MIKTVSDNSIPGTYGIAFTRVDTAAQISRPVPSDIQRNSSIEKAVIEHIA
DHPAAKVVMSALVEALTGVFVKAQGEIKGWAEIVQAASRPHDSNRKGTGV
LSPRFDVMGCVGWNAATIRATCRVGTLREKGTLFTNLMLSDNFKRILERS
MTDPALQRKLEGLVDLDYLKTTDGNLYAMSGWAARASESREQIGKAAFES
ASNLGSAQISARELAFHRHNPVNHPSNQARVGFGISSPANNDLQVLRGHG
SSVWKVKPGSDFARLASASGKPVIAGPSGTASRFMAVARFISPGCLRDLG
LDSEQAFKELVRYACYGYFGQDDHHSMLEVNLGIAPHGLDEQWDDKLYTE
PFSHVIMGRGFSVDNAAQQHIVARATDEPVEHSAADRVG
> STM1183 flgK flagellar hook -associated protein 1265468:1267129 forw ard MW:59109
MSSLINHAMSGLNAAQAALNTVSNNINNYNVAGYTRQTTILAQANSTLGA
GGWIGNGVYVSGVQREYDAFITNQLRGAQNQSSGLTTRYEQMSKIDNLLA
DKSSSLSGSLQSFFTSLQTLVSNAEDPAARQALIGKAEGLVNQFKTTDQY
LRDQDKQVNIAIGSSVAQINNYAKQIANLNDQISRMTGVGAGASPNDLLD
QRDQLVSELNKIVGVEVSVQDGGTYNLTMANGYTLVQGS TARQLAAVPSS
ADPTRTTVAYVDEAAGNIEIPEKLLNTGSLGGLLTFRSQDLDQTRNTLGQ
LALAFADAFNAQHTKGYDADGNKGKDFFSIGSPVVYSNSNNADKTVSLTA
KVVDSTKVQATDYKIVFDGTDWQVTRTADNTTFTATKDADGKLEIDGLKV
TVGTGAQKNDSFLLKPVSNAIVDMNVKVTNEAEIAMASESKLDPDVDTGD
SDNRNGQALLDLQNSNVVGGNKTFNDAYATLVSDVGNKTS TLKTSSTTQA
NVVKQLYKQQQSVSGVNLDEEYGNLQRYQQYYLANAQVLQTANALFDALL
NIR
> gi|28871720|ref|NP|794339.1| candidate type III effector Hop protein [Pseudomonas syringae pv. tomato str. DC3000]
MKKSGAGTQAYALFASATGSSSKGVLSTIARHLTGCFAPNKTALHSATAV
SYELLPGNYSVAASVHGLSVDH RQPALTRLSNVLFNQALALDLERFDEGA
PADEMFRPSLKREGAHPRLADSLGGEQLAVQTMEKGLKRLAEDPAQSFAR
CHSFFYPISSDTTSPQASLHSVASSSG
> Q8XRE0_RALSO
MGNQFSINRNRPANAGEAHASSTEAKPAPDAASPSRQVRVATAPSILEGL
RKLRLPFNFGARVPPLPEETWAEIARRVDTKTLHRMRAVSKTLKAAADTE
IRQLTVNTVEALQAAKRYACLETLKMDGYFG DADLEGLPASLRELDLFST
RYVSAKGLAHLSKLPLVRLKVRANGIHAEAARALAASTTLTALDIRGNGI
GDAGAQALAANTSITSLDASFNDIGVAGARALAANTTLTSLDLGFNAIGD
EGLQALATNTSLTSLTVGSCEIGDAGAAALANNTALTSVDLTCNHIGEEG
AEGLAANTVLTSLDLTRNSISDRGAAALAGNTTLTSLCVNFNDIYGRGAR
WLAENTTLTTLDISYNPIGDWGALGLADNMTL RSLSVVAGRIGVEGARAL
AGNTTLTTLDISVNAIGNEGAQALAANATLTTLSLRSCELRAAAAEAFKA
NTTLASLDIGENRIEAAGAQALADNTALTTLRVTGNQIGMAGAQALAANR
SLTKLNISQNDIRSAGAQALAANTSLTSLDVSSASIGAAGAQALAANTSI
TWLNINDNHIGDAGAQALAAHPALTSLYACDNFIGTAGVQALAANTRLTS
LSIWGNRIGDAGVKALAANTTLTKLTIGKRVDK QLGEQIGDLAWQELMAS
VDRSGMTFHSL
> gi|15618613|ref|NP|224899.1| S/T Protein Kinase [Chlamydophila pneumoniae CWL029]
MDCRGGIPLPEPQVIGGYHVKKILSKKLRSRVVHGLHPETRHSTVIKVFS
PSPSFTSRSVYNFLKEAQSLHQITHPNIVKFHRYGKWQDCLYIAMEYIEG
ISLREYILAQFISLPQAIDIIFDIAQALEHLHSRNILHKD IKPENILITP
QGKIKLIDFGLADWDTEIQRAHPSVIGTPYYMSPEQRQGESHSPASDIYA
LGLLAYELILGHLSLGRVFLSLVPERISKILAKALQPSPNNRYSSTREFI
QDIHHYRMSGDMQEDLRIKDHTVALYEQLQTQRFWLAPETLRFPDFISGV
LYHQGYPLYPHAYDTLLEGDVFNLWLGYSPISNATIALSVVKSLVCQQDL
QRPLLDRVCEINECLIRMKIPIDEMGISILCLEISKENKEL SWIACGKTV
FWIKRQGRVVQDFESFSPGLGKITSLQIRETKVAWEIGDEAVVCTLELEE
SVASLKTLSLAELQDRRQKAIFCPIESIHGGIQSRQHGSNSPSTLISLKR
IR
> gi|15596903|ref|NP|250397.1| type III secretion protein PcrV [Pseudomonas aeruginosa PAO1]
MEVRNLNAARELFLDELLAASAAPASAEQEELLALLRSERIVLAHAGQ PL
SEAQVLKALAWLLAANPSAPPGQGLEVLREVLQARRQPGAQWDLREFLVS
AYFSLHGRLDEDVIGVYKDVLQTQDGKRKALLDELKALTAELKVYSVIQS

QINAALSAKQGIRIDAGGIDLVDPTLYGYAVGDPRWKDSPEYALLSNLDT
FSGKLSIKDFLSGSPKQSGELKGLSDEYPFEKDNNPVGNFATTVSDRSRP
LNDKVNEKTTLLNDTSSRYNSAVEALNRFIQKYDSVLRDILSAI
> gi|34498031|ref|NP|902246.1| hypothetical protein CV2576 [Chromobacterium violaceum ATCC 12472]
MTDFSVARFSQSTGANGVPGEYSVNSKKTGELPKLPELQIGADPLHNVLN
DLKSLDFESCGLEAPIPDGLNYTVQLLAGNEGFGALDLHGTFSIGDLMAK
LQSLYGQQREGFWAMQAELLPLVAAVALLAVQQRSEAAELNARALQENAA
SEVVGGAGQVFSAVGGETARAVASKGAAQISEAISLHAVGHEVQGAQEET
SGELAQKLANSIGQHLANDARQETANRQEYIPLMEGMIAVLAKDGQSWVR
R
> gi|28868578|ref|NP|791197.1| type III effector HopPtoN [Pseudomonas syringae pv. tomato str. DC3000]
MYIQQSGAQSGVAAKTQHDKPSSLSGLAPGSSDAFARFHPEKAGAFVPLE
GHEEVFFDARSSFSSVDAADLPSPEQVQPQLHSLRTLLPDLMVSIASLRD
GATQYIKTRIKAMADNSIGATANIEAKRKIAQEHGCQLVHPFHQSKFLFE
KTIDDRAFAADYGRAGGDGHACLGLSVNWCQSRAKGQSDEAFFHKLEDYQ
GDALLPRVMGFQHIEQQAYSNKLQNAAPMLLDTLPKLGMTLGKGLGRAQH
AHYAVALENLDRDLKAVLQPGKDQMLLFLSDSHAMALHQDSQGCLHFFDP
LFGVVQADSFSNMSHFLADVFKRDVGTHWRGTEQRLQLSEMVPRADFHLR

> gi|28871243|ref|NP|793862.1| type III helper protein HopPmaH(Pto) [Pseudomonas syringae pv. tomato str. DC3000]
MNTINRNIYPVSGISAQDAPVQTDQLQPQGQGIRPGHNSNLIDFGLIQQA
NGPHSSLNTSSSRIQPTDTSTSSNRLGGNGDQLLNKLVEAIRNILNN LLS
LLEGNQHQGSSPAQTQREQTPTSTQSHASPSSSSSSSPSTSSQSSPSVPS
TPQGNAEKPFVVQSDHPAEKPVSLQRTSEPTSVTPPQTPPQAVERNSITP
DKAPAKPEAVKPAVVNDPVLPKTSIPAAAKPDSTVTAAKHATPAARGQGA
DMSGMIGFAKEANTTGGNNGEVVTVNTVADLKKYMEDDKARTVKLGANLS
ADSKVSINFGANKTLLGTDKGNTLHNIYLASGKTASNDIFQNLNFNHD AR
YRENGDMQMFISSGQKYWIDHITATGTKDQNPKGLDKLLYVGGKADNVSL
TNSKFQNNEYGVILGQPDDSAAAKAEYKGYPRMTIANNVFSNLDVRGPGL
FRQGQFDVVNNSIDKFHLGFTATGNATILSQANYFSNGVDVSNKASNSGV
LDDYGDAHFKDIGSNVSFTQKSPVTAWTPSYNRDVKTAEAARAYDLANAG
AQVVK
> Q7AAV1_ECO57
MPIINKSASNYVEYISKNNPPYLSKKRD ASINLNGKVSDCNGEIIWCRHI
ASYWSEFFCSNSGKIDYETFSSPQLLSKAIVIQENKGTNNIKGDVYFVEN
ESWGSVIYNLFLQLEKENKSHTSLEVHSPGHAMALGIKIKNDKENKFVIN
FYDPNQTATHKRVFFCTNNICDIINLTAYDFLSEQCLKCYGLKEDTLSLF
VDKTKSNDNNNVFIKKLPDNILQGVVINFAMGAGLREIIKKVYNDTRFTD
LTKSQMKILCESKNVNNVPGLLLALQNGH DNVIDEYGTLIKKSNLNKEEL
IHILSARTLDGTIPGLYQALQNGHAQAIKSYGNLVLDTINKNIDLEYLLS
AFKYEAHSSNKYTPGLFSAFQNGHADAIKAYCGVLGNSNLKRGEIIRMLE
ARNYDGAPGLLLAYQNGDINTIQSFFDSLIMLDISKDFIEELLTAKHYDF
TGLSLAISHRHDHVVKLYGKLFKKLDTSPYKMSIILALAIDCERNNANII
IDSEYKSNKAVKEYVEILKEFNICPEKVAE YLSEFSGKHFLDVYNYYSN
> gi|19749310|gb|AAG45731.2|AF229441|2 Y4yA [Sinorhizobium fredii]
MTKIGHGLPPILRSATADLLTKYGPLLFDWAARHGSPLNLVWPDALRENL
AALKGVLTERRVQHAIYYGAKGNKSPGLMQAALSASAGLDVSSLYELRDA
RRLGADGARLVATGPAKTNAFHQALINCNALISVDSPEELEDLIHGLPAD
AGQQPILLRLRPRDQS KSRFGMPPDAIVHCLVRLAGEGRLRFDGLHFHLS
GYRRETRVAALSEAADLIAEARRMGFFPGMIDIGGGLPIQYVDRARYDAH
LAAQAPEDYRTGKIPDSFYPYGSTLSAADWLHRLLEAEMNQGRSVAGYLA
REGLTLAMEPGRALADQAAITVFRISRVKAVGPDSHVIFVSFSACETWFA
SEFLIDPILVPATKATVQSPPVRAYLAGHSCLDEDVISNRWLTFPTAPRA
GDLLDTPTPAATRWTSS KTSSIAIPCPLASASSKTPKGGPISFPDTIGEV

> A0TES8_9BURK
MDSRRRHSSGPAQVRRVRSTSRRVGGRGIEGQAPARQQSEGADARRHEIH
LQPPAAMNANAVAQHAARLLIVADDLSGAADCAVAGLHHGLTAMVLLDTE
HAPAPTNGVDILSVDVDSRRASKDEAARRNVAAIESLSHSDTRIYKKVDS
TLRGNVATEVAALASHLGMAIVCPAYPASGRTTRDGRQWVRGVPVEASEY

WRNENIRGKADLVALLCAEHLRVAYAAIGTVRAEPGALAACLREMQSEGA
QAVVCDAESDDDLQRIARASATLDNVFWVGSAGLAPAVIEALEPRRGIAE
PAAAGDDPQGPVLTIVGSMSSISHAQVEYLKANAGASLVALEVAVSALDD
PRSDVTTAVCNALRHGRHVVVSMSQTQRGDVNDGLLFCNRLASLLAPAVR
HAGAIIATGGETARALLIAAGASALRVVDEIEDGMPLLECRLQGKTFPVV
TKAGGFGQPGSIHHAWRRLANEGKTANTAQTNL
> Q7AE34_ECO57
MITDLILHNHPRMKTITLNDNHIAHLNTKTTTKLEYLNLSNNNLLPTNDI
DQLISSKHLWHVLVNGINNDSLAQMQYWTAVRNIIDDTNEVTIDLSYNLA
ITNIDTSDEHLVEVSDNSEGNYIKDNDSMSIRYRSKYYSRDSALIEEETI
FSDAELKAILPMRRMYGVSDYKSNSSSLSSHSGLKDPTGTPVCYYIHNES
KPSLGYGPTSNNWLSQSFTTEL
> Q8XQK6_RALSO
MLKVTPNFLRPGTGTSAPAEPYHNEKAVSYQSEEDGTSMADFEASPKGRR
VAGQYRAGIDKLKDFLRVNGTPERDTAIHALDAFGKRIGAGHEGMYSTQI
SRMYGEGKVAFDGFCSGVGNPAIPMGVRVSAVEELAKGLLECSEGTASNL
DIAAQALHLSQHGLL
> Q1I4Y7_PSEE4
MTDLNTLRSSLASGEHVFADTLAFVAAHYSYQPQAFDNGD VHNAAGQNEG
SCKTLGLALLEGLSDQEALLAFGEHYRSVVATPEGSDHGNIRALIKHGLA
GVKFAEQPLARLA
> Q8XQ26_RALSO
MKLARTPPPSTSAAAGASAEFSAPSPSVGSKRPRSPGALAYLSQAAMPNK
RLRLSASDGSLSAGPEVKAPALPTLIGTERAADLDVRFGSLQPVRTKDGD
GIHDWEKDAEGRPLAHPRFIALQGGDAPPDWTDPAVRKAFDIDALKAGDK
QYIWAASALGRV FIGEEEPVGHDPDSGKQRRRGHPLLVSGGPARICGEFR
FDAETGKLVVINKLGRYSRYEDRREAQLQEVATIIRAAVAPLGLEVETEY
RSGKTPDALVLPSLDPKYREPPAD
> Q7PC45_PSEU2
MFIASPKTNVQECLMINPVKHNFSHLGFSNAQSTSALGPASNKVPNFVSR
GRGKGVPVEQFNTAEECRLAGRQDSVLDSIDGKEFMRLLQKYTASETTEE
EFADLRASIPRYSIELAKSDQPKV LYRGISLDDEAASLLLNTSRGYRSRE
IAHGLIHGLRVVKGVYTATGVASASTVSNVSQGFALVNAKRKKETPVLFV
LKAMPAVPALNHSGAKGVTLSESRLPLSAASKSEHEVILDITNRYEITQA
RRSGEFIVVDMTVLGRSKRGGEFALVETDKWKQLSGAKGSNPGGLFQAPN
GVKWYVKTNPSANRLLNEVLASRLYRAAGIDVPDIELASRKGKPALISKL
IVGNPKDLDTLAKNSQLKCGFAVDA WLANWDVIGLTGDNVIFNHRNKPVR
IDLGGALVFRAQGEHKGNQFGTTPMELVTMLSREDNSSSRAFRKIERNDI
REGIAAIEKIPDARIAALCAEHGPGNHSERIELGKRLISRKKWLVDMKQT
LPYIHRRKNERGDVVTVKKPTSPSAADTWRDRYATAVFVPHSAVRGSMNN
LPFRSFTPPDTMDGWRRFTTRAVNFTEPEFKRSQHLAPASGAIIFEPDGR
VWITEPTNHPFGATHAFPKGKQEAGL NLRTNALKEVYEETGLLVEFHGFI
GDYDRTTSRTRYYLAKRIDGIPSDMGFESQSVKLANITEAKRLLPNAVDI
AILRDAERAYLKGPFK
> Q5EN61|Q5EN61_XANCV Early chlorosis factor protein - Xanthomonas campestris pv. vesicatoria.
MQIKTAGLGPNAHPHGPVSGDGSSPADRSIEIEAGHGSDTLSRACPQPAS
ETTMGLADLLSISSQRP SRPEVMPAITPAGDDEGVPDLRSQPARSGASQP
CPANPGEARDVESGLLMRDCSSRIAAFLGNLSVVQELRQFDDSEFVDKLR
NELVTEPGCREPKTLLAAMGERVNELRSHLNKQASTREEAEFLAEARRYL
EPVMTSFETDIHTLQHDASAAKRIYQGAMTLLLYPLPLATLFTQKTGTYA
AFNIASYTYTAIQLVSLMRRPTTDAKLFMKHAINRHSLVFFISLIYAVPT
FYAKAAPLQRNAGFVAGA AMAQGMMMFGLRLGQDLMDSMRLRFNGAFNRR
RDLPDGFRDAIEGVVGDLRAGLSNVNRSVGEFQQDRRITPHMDRQLTFFK
QDLSRIVTGLERLVATGTRQESPLAASDDPPGPLEAVRRTLEASFANNPD
LKGKLALATVAFAVLGSNIALMRNNGLALPDFIADAVVSSTFLLREALSP
HVTHAGMNDSVSDTVGGMTIGLPFSVATVMSGYMDDPRANPSGFIAGTVG
YTAAYLLFGRVAGDVLSKG LMATSGALGWGQQQAIRLGRSAMALGFELVA
GHRSPAAADVHDLAGIEMADVPLEPLCFSPSQRASAERTLVGTLEQIGDE
WYDARDEWEGESEGTAGRDAPWVDAPAELPVQPVPQSE
> A0IP74_9ENTR
MLLGVIADDFTGATDIASFLVENGLPTVQLNGVPRDDLPVEAQAVVISLK
SRSCPVAQAIEQSLQALAWLQRQGCRQFYFKYCSTFDSTAQGNIGPVTDA

LLDALGESQTVISPALP VNGRTVYQGYLFVSDQLLAESGMRHHPVTPMTD
SNLLRLMEAQAQGRCGLVNAAEMDRGAGAVAEKLRQLALQGMRYAVLDTL
NEQHLLTQGAALKDMKLVTGGSGLAIGLARQWAQPGQGSPQAAGAPQGAK
AVVLSGSCSTMTNQQVARYRQQAAAMAVDVARCLNDAERTAYITELADWV
QQQASAPLAPLLYATAEPEKLQQVQQQYGMAAASQAVESLFAGVVKQLQQ
RGFSRFIVAGGETSGVVT QALGIRGFHIGPCISPGVPWVRAIDQSISLAL
KSGNFGDENFFARAQTEFPL
> SOPB_SALDZ
PAKEAYRLAAATFRDAQVKHLNSQPWQTIKNTLTHNGHQYTNMQLPAADM
KIGTQDIFPSAYQGKGVCSWDTKNIHHANNLWMSTVSAHEDGKDKTLFCG
IRHGVLSPYDVKDPLLRQTGAENEAKEVLTAALFSKPELLTRALEGEAVN
LKLVSVGLLTASNVFGKEGTMVEDQMRAWQSLTQPG KMIHLKIRNKDGEL
QTVKIKPEIAAFNVGVNELALKLGFGLKTSDSYNVEALHQLLGNDLRPEA
KPGGWVGDWLAQYPDNYEVVNILARQIKDIWKNNLHHKDGGEPYKLAQRL
AMLANEIDAVPAWNCKSGKDRTGMMDSEIKREIICLHQTHTLNAPGSLPD
RSGQEIFQKVLLNSGNLEIQKQNTGGAGNKVMKNLSPEVLNLSYQKRVGD
ENIWQSVKGISSLITS
> Q8XRI4_RALSO
MTNPFRVSPCAPPAQSPSPSAAAAPGDPPPAPAGKAPRKRGTLSLPARLL
PKASETEVSTLIGASKHSGRPVNTVADYSPPARTVSAPPDLPTTADRDRA
SRSLARTAGLVQRAQRGLQKLQGTRHARAPKPDASSTRLPVTVPDSAPRY
RQLLAQGFAATQDAGAVGRLADRLRALAPPLAEEAGERTRYARPMLLAKV
LEHTCGQDAAAALRVLDRLTGGMRLGAPTFAAPDGSPDAHESALIRQASA
ASDAFFDALSDFHDAAPAAETGAAWRTAQLLARYHAGFDALCSLMQIPDV
PEQRQAAHAFLQAADALQHGSVKPAGSPQALLDKHPIGSGTPEESLAIKT
LHGAAAVLRGEAPTPEQAGALFAWRQGFREEGPGTALDKTKARIGRFVRR
VIPRVEKHGWRTMLWQMIGKKASPLSSARLGLQAAHRKNLAGEYKDYVDA
LRSAVTTLAARYAQAEAAPLDPPGALPWRSPQEMWSSAVLKHWASALAQA
SPQDCVLTDDILAAIGLQLRDTVTRAFDALAAGIAQATEHDDGLREHLRA
SLATLTRLSQDAGLPPARDSPIHPPPGLIGLAPSGALLRSWSPAAPAGPL
EKALKSAERIEQQDDLQLREKTIEAARELIESLLTTIDAGGKLRLASGSA
LGVNTGTLSTGLQNAGHALAIPLSVQADFHASRKRQAVVEFGRGAHGRDL
FIGTDKTVHAAAGCGGAIGYDIQVLTNRIRASLGMSVVPIDVERGKRVGV
MFRAVRQL DRAQQTVGGYHDWEAVKYDDTATRQALISLSNWLFEQATEHR
QRPLEREAIWNALALHCGTGNTISVGWVDQRKRRLRHKLRLSGGVQARIS
SKGAPIRVGPSARLTAEATSRDATATRETAGIMRVEQSVQGHGGHLTLRA
GLTYSAGKMFYTSAAPDAATGSDATGGRHRITQGFNGGTPLSWSKQFAEL
GHTVEVRTIHLNGRLADRVSYSDKRYKSGTEFLALIRTRQRVWIDMLSRK
PGMTREAAE QEFKVFCDMVERHSGPNVSYLYRERLKPAIAREIEGQLDLA
DMHRALDIDALAGLPADSERASTEEGTSPPQPASPAKPSLPGKHAAPAPA
PDIMRGEAEASGPPSVAGSPPPAAAESVPLLTRPAMRKTSRLPDKARNLL
DKYTAKVQTAQAAGLNDAQLHTLLTGNAMTKGRSIRAVQREVAKRISEGT
WSPNSRARTAVPAGYATLPGSPLEAAAAVAAAAHAPGSPPSASHLARAQS
TGLTRRESEA AALAEAETHAQACEMAADAVLFDEASWLPERLAIVENISR
RENKGLSTTLQLYGTSEASGQRDLHNLKFG
> HopAB3-1 Pma ES4326|Genbank: AF458050
MVGISGRAGPSGSYNYSGHTDNPEPVSGRARDSNSEANSSNSPQVPPPLN
APASPMPAGRPRFLRSMALSSQTREWLEKGMPTEAEAGVPIRLQERAANT
APQARAEERHTQPADAAAPHARAERGRTLQAPASTSPLYTGA VPRANRIV
QQLVAGADLANIRTMFRNMLRGEEMILSRAEQNVFLQHFPDMLPCGIDRN
SELAIALREALRRADSQQAARAPARTPPRSSVRTPERSPAPRTATESSSG
SNQRSLLGRFAGLMTSNQRRPSSASNASTSQRPVDRNPPRINLMPTGANR
VAMRNRGNNEADAALQALAQNGINMEDLRAALEAYIVWLRPIPLDIANAL
EGVGITPRFDNPEEAKVDNPLMNLSSALKRRLDA
> Q3BZN0|Q3BZN0_XANC5 Avirulence protein AvrBs2 - Xanthomonas campestris pv. vesicatoria (strain 85 -10).
MRIGPLQPSIAHTAAPALPTHTSAISPTQVPHMPGNTPPLRERPRRRAGN
MPPLVPLNDSAMTGKPALVALDSEFSEQRLAEVQARQITVQTLQGKLATH
LAQAGTALKPDSIAARFAAGTLEPVYLDTAAFNAMSRGLPARARAAAGPV
LIDAQQGRIIFNLQRAFAPGDTFSDAALAALGKQLNLSGHGLATPNWLQP
AAGTPGRRKLQQAARYHGHEVPARDGGAGFFKANDHRLLEGKQVLLRNHQ
KSLVHNHYFEAPSTRAFGKDVMVHRGLFDNHAGIPENSLASIDHAYEQGY
RNLELDVEVSSDGVPVLMHDFSIGRMAGDPQNRLVSQVPFAELREMPLVI

RNPSDGNYVKTDQTIAGVEQMLEHVLKKPEPMSVALDCKENTGEAVAMLL
MRRPDLRKAAAIKVYAKYYTGGFDQFLSNLYKHYQINPLHSQDAPRRAAL
DRLLAKINVVPVLSQGMLNDERLRGFFRSNEQGAAGLADTAMQWLDSWTK
MRPVIVEAVATDDSDAGKAMEMARTRMRQPDSAYAKAAYSVSYRYEDFSV
PRANHDKDYYVYRNFGELQKLTNEAFGVKRTTAGAFRDDGESLLTDQPEA
ELLAILENRALARGHTGNELDVPPETPIDINRDAEIVKQRTQQFQASSIP
ADPNHISAVREGKQHDHTADMVNDPAATRALDKRAKALGLLTDKYRGAPV
THYLNEQARQTETD
> Q8XUB9_RALSO
MKRIDVHAIPEPAREPSEAPASAPVIAQPTDATLALLEAAVRKLLQELNG
NPAPARHAKKRWRIGQTEVAVLVFVTERKNANGHSEWIVERVRLGERALG
MDVLSNGGELASPGSADIGPVQGDGGTTGTVAVVVEQYASRTTAADAGTT
TRPA
> gi|15605594|ref|NP|2203 80.1| predicted Protease containing IRBP and DHR domains [Chlamydia trachomatis D/UW -3/CX]
MKMNRIWLLLLTFSSAIHSPVQGESLVCKNALQDLSFLEHLLQVKYAPKT
WKEQYLGWDLVQSSVSAQQKLRTQENPSTSFCQQVLADFIGGLNDFHAGV
TFFAIESAYLPYTVQKSSDGRFYFVDIMTFSSEIRVGDELLEVDGAPVQD
VLATLYGSNHKG TAAEESAALRTLFSRMASLGHKVPSGRTTLKIRRPFGT
TREVRVKWRYVPEGVGDLATIAPSIRAPQLQKSMRSFFPKKDDAFHRSSS
LFYSPMVPHFWAELRNHYATSGLKSGYNIGSTDGFLPVIGPVIWESEGLF
RAYISSVTDGDGKSHKVGFLRIPTYSWQDMEDFDPSGPPPWEEFAKIIQV
FSSNTEALIIDQTNNPGGSVLYLYALLSMLTDRPLELPKHRMILTQDEVV
DALDWLTLLENVD TNVESRLALGDNMEGYTVDLQVAEYLKSFGRQVLNCW
SKGDIELSTPIPLFGFEKIHPHPRVQYSKPICVLINEQDFSCADFFPVVL
KDNDRALIVGTRTAGAGGFVFNVQFPNRTGIKTCSLTGSLAVREHGAFIE
NIGVEPHIDLPFTANDIRYKGYSEYLDKVKKLVCQLINNDGTIILAEDGS
F
> gi|58580860|ref|YP|199876.1| avirulence protein [Xanthomonas oryzae pv. oryzae KACC10331]
MGSLAVSTEISHNPLPILHAGPGSPSCLRLTLVVEGCQDHPKLCTAMRPG
KLCRDHRPLVWRRPCKEVCLMDPIRSRTPSPARELLPGPQPDRVQPTADR
GGAPPAGGPLDGLPARRTMSRTRLPSPPAPSPAFSAGSFSDLLRQFDPSL
LDTSLLDSMPAVGTPHTAAAPAEWDEAQSGLRAADDPPPTVPVAVTAARP
PRAKPAPRRRAAQPSDASPAAQV DLRTLGYSQQQQEKIKPKVRSTVAQHH
EALVGHGFTHAHIVALSQHPAALGTVAVTYQDIIRALPEATHEDIVGVGK
QWSGARALEALLTKAGELRGPPLQLDTGQLVKIAKRGGVTAVEAVHASRN
ALTGAPLNLTPDQVVAIASNIGGKQALETVQRLLPVLCQDHGLTPDQVVA
IANNNGGKQALETVQRLLPVLCQDHGLTPDQVVAIASNIGGKQALETVQR
LLPVLCQDHGLTPDQVVAIASHGG GKQALETVQRLLPVLCQDHGLTPDQV
VAIASHGGGKQALETLQRLLPVLCQDHGLTPDQVVAIASHDGGKQALETV
QRLLPVLCQDHGLTPDQVVAIASNGGGKQALETLQRLLPVLCQDHGLTPD
QVVAIASHDGGKQALETVQRLLPMLCQDHGLTPDQVVAIASHGGGKQALE
TVQRLLPVLCQDHGLTPDQVVAIASHDGGKQALETVQRLLPVLCQDHGLT
LDQVVAIASHDGGKKALETVQRLLP VLCQDHGLTPDQVVAIASHDGGKQA
LETVQRLLPVLCQDHGLTPDQVVAIASNGGGKQALESIVAQLSRPDPALA
ALTNDHLVALACLGGRPALDAVKKGLPHAPELIRRVNRRIGERTSHRVAD
YAQVVRVLEFFQCHSHPAQAFDDAMTQFGMSRHGLVQLFRRVGVTEFEAR
CGTLPPASQRWDRILQASGMKRAKPSPTSAQTPDQASLHAFADSLERDLD
APSPMHEGDQTRASSRKRSRSDRAVT GPSTQQSFEVRVPEQRDALHLPLS
WRVKRPRTRIGGGLPDPGTPIAADLAASSTVMWEQDAAPFAGAADDFPAF
NEEELAWLMELLPQSGSVGGTI
> gi|28871829|ref|NP|794448.1| candidate type III effector HolPtoAA [Pseudomonas syringae pv. tomato str. DC3000]
MNRISTSSVNSSFNYTAPTKEAQNRFASAPDNSPLVATTTSIT QTLVQPG
RLDKNHRLMDSKIINFSVAFNLGRTRSIAGGSA
> Q3BSU7|Q3BSU7_XANC5 Xanthomonas outer protein C - Xanthomonas campestris pv. vesicatoria (strain 85 -10).
MKTSSASKSHYTGQFPQGDLQPKTPIPVGRNSEIPKDAALSAQLKGLRPL
DRKPSKMPTSSSDLQAVNKMIAENRSNDVGKGQFLLSTQSLGSAERVDSQ
NYHSRNESGKAPLFFSEFATEKPSLQSVKSLSQGKQDYYAVRHVGKRGRD
LFTDEPIEGENSKLGRLKTSPLLSTQSQGTRAIRAFAATATIDKSRGEYV
ARLHRHVVEALGDEGGVVHLLRPTRDHYAEDSTLNFFTFCEQTELASSLN

SLEAISAKKEDRKPIVFTDHKSLVGLSRTSRTEASPIGRLVQQPYFVTSD
LKRGDKVVITDDHIQAGGSMLAMEAAAKEAGVDVLALATLSAHPFSPQLT
MSSEVSAFLDETLAAWDPQGKVTERLAAIGMPREKLTNSEAMILIAYAID
PASDSAILRFESIQNNLFKRAHLHNVGIDPDAPDTLEQMQKLSSMQEMAG
TDSARHFVDDAKVLEGEHDSLIPILKMKPASPDEIVKELDQVSLASRSSI
QASEVKQVVVLDWDDCLRDEKGLNYKLMHNALAITAREHASTLPELGDAV
AVLHSKLNGAEPVDEEAPLLMKNQKDFSSYLMGRQSIYKRHIIEDFVRKM
LPGISEHKAASINNAVYSNFVREYKMLVKPEVSKKNYSRDVPFPDIELSL
LPGAKEILEKSRNANSRVILISNRGHGDLENEINHLGMMHYFDVVSGAEE
VTLEKSHLPPSQMPEDLQKRLINSLNGSDEEALRATLSEAAIYAHPDSTT
IERIDKKPDSTRLAESLERLSVQPNVPITSYGDQPSDIKQLKSVAEPKSR
VLKGVIVNAQRDDVGREIDVDGVPTRVVSKITEL
> gi|15835056|ref|NP|296 815.1| adherence factor [Chlamydia muridarum Nigg]
MPVSGSIVASKENTSASLQPATTLNNGSSVEFFQQITVYDDARNFTNHTT
EDVIKIGEQLQRKFYNMTEGTQVPFTTSPAYHTGNWKTAFIYNLAQVIAN
LFPSTIQPIRVNSTRSSPFSTNSSVEKRNRFTSKDTKVQPPHTTRSVSPR
KRKSVIVTRSVNIHNHTPDKKSSGESLRNISLEKPVLSRKRRDVEKNKEI
KNEPSVNKYDLTYKNIAEKLGLSSEQQTSVKELLNKLRKAITNYNALIEK
NSSEGQSLLIRQAKLVEEIQEKISPAVSDKKGNEKKTEEKSKVSFIKDVA
DIIKAEFSGHLVPVDKIIHGIWIAGAPPEGIEQYMRVFLDTYPDYSFYFW
VDSNAYGAAKFSGILKRMAFDVAIQELRDSTEESTKVFVKEYDDLKKKYE
QRIAQTHSTKKKNEYLQDLQSLLEKYKTVSDEVRGRFDVLFLKNVVITQD
NFFNYCLLKGVGNISDETRMEYLEKELKFSKEEIEEYRKLKESNKEKIAS
VVKKLNEQLGSARVHIKDIKELNSMKHSQNIYNYEMEMFLRWNYAAATDQ
VRMYMLEEYGGLYTDLDMMPAFSQEVLEVIKKHSVGDRMFEDMTSRRAIS
DAVLKLAVGETTTVSIEDIGKDIDISRLTEEDKTKLGELFKELEFFVKKD
TQNSSKLGKAKSFFQPMNMDIVRDTMPILRRYHYYPELGWFVRGLNGLMV
SHKGSAAVSHVIKGQQDAYQELAAMRQEVLSGEFFRSLEDLTHRQHKDLI
GGHLVSDYLAKSLFFDYRQDSIMPEAVSTLGITGPDLVAQKLVELFKDWG
PLGRDFLTPRGKKLGDMAFLGSYKKIPLDPSDPRKYTFDWMNPLTVGSND
VTPADESTWCGSKKRCVAELLFSDSSTKLSTNKLQGVTRTKIDTSTFTSL
WKEESKKKLPPGLLESFNRFITEKTVDILKLSELDQKIYVTQSAIQDDNI
AKASLFSLQLQLAHLLRFAPYPVTNHVHFFPQIQKELDSDYVKAIKLFLK
TSSTTTVVLWHSSQQNLSLFFKELLAISERRVAIYNLLNPEEAAKARKAL
EKKERDKKGKQQEKDSSEGEDQQGDQKKNFSEYAELLKQYEQHKAKDSLG
LLSEAEQMTFLEITTQIAENPTLFSVISQIEAEISSGYVFRALESQVSKW
FSLPEQQRRQQILNLLKELEKDAIKNSKQDKKDHKNWLETLYDQAEQKWL
QEPKKKLQDLIKNSGDNSRVILKDSDQLLSRNELFQSMIKKGYPFADFAN
ILRFMVADAGISGIFSSKAIFPAPSKQLVDLLKKTLGGDFATLQDALPTV
YDWILEEPNSERTNKLFKQFPENLQKELKNIAPEHLLTPPIDTSVSALGM
RFSTEVGLESDSVMASIGSGFFNPSSYSMARYMEAIFELQQKIVNGELQT
VETVKNILQKKGAESLYHADRAALLVQFSELRYHLSLTEVQKGLADLDNL
GQAPSYLLTGPLPGLGHIMLRDRDFGLPLATSMTDPIGLSTYDFSGIGGR
KDVFSTPSEVPSIHTIIDRVKYDTFSWTKFYERYSGIWGDLAFRLGAESL
RTHPQTFIYDTEGRCMGLSYLFLAAENIAAYGILQDNLSTLSALFLERER
EKLPLSREDNRFLDRGLALIEWLQHRGNSELQAGGIFSTVDWDIPSLTKL
FEKSSVPGVLVTTPSHAVTLHFFDGAFRVTDPNFGHVDFPSLESALYFLE
YMVQISSDVRAQYGIKEGVSVPQQLKVYVPDSIEARNAWNIPTDAGLVTG
YQMTTLDRMIVRGEASFSGIRTTWATLFAMGLTLNGNRIDERTKESDLDK
TQINGDLLTSFLSKNVLDERGVALGRTLVETLPFVAGTRLVSSGAIVETP
NDVASLLQASRERLSHFKDVIQVLLRELATKIHGKGLKDSDKVSVKSVTV
EPSGEAAIVLEKQDTPKKTSSYTISVKIKSLRDAFQNFGKSLNELANTGV
MDVELGLSVLSLIQYARLVDAGKGSSPEALFNLILDVKELTEMTVGTVIQ
ALQKQFITPAGIDGFRTETLLARQIQKVGTRVGGTVGKALGSVARVLELP
VLETIAGVWGLVSSVEDLLHADSYSDRVAAKVQISFDVITLALTLSSALA
PLAMLAVGPIAAIGMGAASIARNVARKEERHYAWVEYKQFLDSAAKLVVK
AYPESHLLDLSGNRVLGNLYLDLSQNPPLLKGDVSRNYDSLIGHVGDWSD
RQVRNRLGYGYRSSPERALAKGHANTRWPREIPHIAKGVYETVYLGYGIQ
YKVYTEIVYLSNQVAWRDAVMDPTSRYYIPPLVEEGKSATIIAGNTPLNV
IMLRLLDEDTPARISQNLAYKDYKIKLVGGKGGLTVQIGGGGTYTLTGDV
SAKNTISFRAIPPPLGVCFNLSNHAMQNVPMMRPNGTKIEALKIQQTGFS
VIAGSAEGYDILTGEKDTHFYISSGGGKIFSGLGKNWYHIPKLKGRLDII
LAKNSAEHLLLMDDYSYNWQSLGTNLTLIPRGEGNNNSGIFVSNFDASPS

FEQWVNKFTVKMADGITLFALNKSSEDQNFSLATTNTIVTLGVHSVDQTI
WSNRLPEEPSYIENIFNWLKKFCWWLAPKVTVLQREGTADFYYRDQKLIY
HPKPFAELDLHPQPGYHTYVNGSIGDTYIFSESPWVNLSTVKLSLADDLG
HSQTVDLSSLVPTFVRGRMTSQTVNGPSIDLEISSPRYTLPLQISWGSHV
LPRNTRFDVMPNHSPTLGEWYDKLNTNTSEWHTLFHNSMLIPKSLVSVLS
LNNTVTVMVRHLERKNEHILGVENRDSINRKVHGQLYAGFITHTIENRHW
YTLQPNLVEFDITVPAKSIKYFVFRGRLLFRSYLEPKTLEVRSGTPIDFH
VWSQYDKIHVHATTLKLEKFQVYNVTTATDALNRYLMYAQNLAYIYGRDF
VLKFFFIRSGTGIGAIQLVFKDLFKDSFGTEKTLGKEAKSTTPLYSIINP
SYKDHLKFFLGEGEYDLTQYIQEISDTSHIIEMLRDPTTHELQEPSHLPK
NPIVLTYTIDPQKDSAGGGKLQFLDKTIKAYQLPFPTMAEHYYYMDPVSG
DLYITCIALTNPVERAFSLRLPKFKLNWLEFQNIFIFGTHSRTSQALGSS
GTGVMFVGPEILYMEIDLSRVIADRSFPDRVSSRSSVVFPTNDQVVLYNP
SLAKKFYSYSESMIWNLRDRAKGESDRAKAYDIYLLDACTSKDKNLNWAI
PPKLLDFALAYYRAWVSHWVKQSLKKGYLIQLPAGSIQVSLITTQSEYFA
RQRKQGFQVFYSIYGLQGKVIPKQFPGDMLCDIKEDVVLTVKAVDESDYD
NRKIYVVLDLATEEERKLRADKNVIVIPGGEQSKY
> hrpA1 Por 1-2 (1-1?)|Genbank: BAD20931
MPAFPSITSSLTQAGTGVANGIGGALQGANTIASAANMQGNVLAGTGMGN
SLSLAAQNKASAEMDANGAQLIAMQAEETKKKQTMDVLN AIEAGKEDSTN
KKISATAQNARGIRY
> Q8XRI9_RALSO
MQSLTGIATSPSDPTAQEPAQAEPAAAPSSLVARLSRALRRALQRVRPTA
AGHANPAPVAARAHTTIMPRHERRPAAEPSRAQHQMRADAARAAQRLQQP
AGGLHAHHARGTVPFADGNRWMAVCDAVAGWQQEMVRRVSRYSGPLLPDA
NRPTEQGLALAATRMLLAMQQKSRHLVLDSIPVMRLPSALCNAEQVEVLT
VHDCDVFEWPASGGLPPNLCSLHFSRNRRLTAIPGRMGQLQQLRELVILD
SPLRALPTAVSQLPQLERLVLQGSDLRIVPVELGALQRLQTLTLASSRLL
TQLPTSLGQLQRLRQLNLRGNPVLPALPETVGQLSVLESLDLRENTGMTT
LPRSLGSLRRLRHLDCSGMTALTALPADLGACTSLRTLRLRDCVALRTLP
ATLGGLKRLTHLDLRGCVGLTDLPEALRSLPATCRIDVPPHLAERLVQRR
RTVAQAPSVPLPNPGEGADWGRLLQNWRTRLGLYDTEYGSDAFRIWVERS
ANAAMPDEVRSRRDARRLGFLVDGLCNSATLRCLVFQRAHERYALGHLDD
EGLAIPELMALLTEECVRNGPSFADRAATLMRHEAWSSLLEGATGNDFIG
EAVEVLADTLPQIYADRHGMPIDGDTVLPVQRQAAAAQVWLPATPTIH
> Q7PC44_PSEU2
MSMNTSVNNNSPGWSPVSSGNLAPSPDYSGKSSSNAVHFLPPESTQRSP N
TVSWGNGAPTTATAGNGQARGGDQSDLIEALTTLIQALLKRLLGDSSASN
SSANNPSENPSPASSGPGRASASPGGAASSTPDSGLSEQSNQAQPFNNTG
LGSHGGANTVPDRIASPEAAQKMVEDVKNAGGGTLRIQMTHDQINDPAQM
AKLKAMVDAGDKQGVKIQFTFRDNANGGGGNVLSGDKLQQAADDVKNVVS
ALGNHPSFVLDTFNEGGKSATQGWADMQSTLIKSARDAGYKGDIVVEDSN
WGGGLTAGGESGLVKYADQLKAANGSNNPGLIGSIHEYASGADASSRLSS
EIKALTSAGFKPQIGEVGNANWTGGGNFEQRDGANQAVKDNMGALKAAGA
DVLPWMDQFENGKIKHDVGFSKGDQFS
> A4XN96_PSEME
MIRPIPNASSILHIPSVARAASPATPLAQVRSGAATTTGTSAQQASKFAA
ALVQNSRELGRRDLFAKINALQSQASKQHELAHLLLATHERGMEESARAL
RKTLKNGQLLRMERGKLLKQIVDSMAGDAAKAHALLQAAARQAKGEKSLN
EQQALNQQLQLLRQQHGRNPRPLFNTGNNTRILARGGEDPQRRDKLRSLY
SVAVTEQLNVVGLIEALLNSTQDDGQKDGREGFEAVLREIRQVVARDMAE
LDGSASPQKLRTMMYAMSTAQYVATLFGGCEHLLGRMRNKNPAMQVETSA
FLKNLLGLIGKGMALNDTLQLTQQIGGRQLKHQLAFLNGLRPMLHQLPIM
LWRDIKSRQNALGNLLSLMAALTSEEQKRLKEGIA
> HOPM1_PSEU2
MIGTRVGGSGSTEIVQANQPQPSAAVAQAHPHAVSPSSNPPLTASQSAAQ
APESSAAGAARLPVAPRHLPTLEKFRAEQPTVQGTSTPTISANAALLIGS
LLQSEKLPFEVMAARLSPERYALQQFHGSDLQQMLGRFAEPGHLPGKAET
EQLIKGFARSLADQLEHFQLMHDATAEAFGPGGLRDRNTLAVSQAALGEY
AGRASKSIEAGLNHSLAVLDERIAALDSQLEGATEDSRPVLLMDRQALET
ARAMLSDLHVDFCKSPEAKRLSAVAAHTQMDALIDKLNVDRSSVGGWKGI
GPIVAAAVPQFMVSMLHLGYIRTATSDAMKDAVPEKSADASMKRALAVGL
TAGVAHEGVTNLLKPMVQAGFQKAGLNERLNMVPLKGIDTDSVIPDPFEL

KNDNGALVRKTPEEAAEDKAFVASERAVLNQKKVQVSSTHPLGEMIPYGA
FGGGQAVRQMLN DFNLLNGQTLSARAVTSGIAGAISATTQTIAQLNSTYV
DPRGRKIPVFTPDRANADLGKDLAKGLDLREPAVRTAFYSKAVSGVQSAA
LNGALPSVAVQPQGASGTLSAGNIMRNMALAATGSVSYLSTLYANQSVTA
EAKALKEAGMGGATPMVARTETALSNIRHPDRASLPHTFQPDTLGGVPRA
VENAYHMARGALQLPTQVVVDTVRVVEDGVASGVSSLRDAHKPAETSSPT
ADDAAAVELTAME EGRRR
> gi|17233490|ref|NP|490528.1| hydrophilic protein [Salmonella typhimurium LT2]
MPINRPNLNLNIPPLNIVAAYDGAEIPSTNKHLKNNFNSLHNQMRKMPVS
HFKEALDVPDYSGMRQSGFFAMSQGFQLNNHGYDVFIHARRESPQSQGKF
AGDKFHISVLRDMVPQAFQALSGLLFSEDSPVDKWKVTDMEKVVQQARVS
LGAQFTLYIKPDQENSQ YSASFLHKTRQFIECLESRLSENGVISGQCPES
DVHPENWKYLSYRNELRSGRDGGEMQRQALREEPFYRLMTE
> Q2NXG4_XANOM
MTIAEIGAEAQPNNRAVLSASEVEKYDPETFADNLALAKERARKGKGCNA
IIEWSPHSNIELNSNGSPLRLGSNPEESFVVLAHELIHAQHILAGTSKAY
NGGDRYDETSEAGKEELRAVGVGKYEYRKTRQPSENSIRQEHGLPIRKKY
KPHGR
> Q8X9Y4_ECO57
MKFPSIFNKIKPQSIQQHPEKNQLNWMLELNKWKEERILTGEIHRPECRN
EAAKRINCAFLSKQNDIDLSGLNLSTQPPGLQNFTSINLDNNQLTHFDAT
NYDRLVKLSLNSNTLESINIHQGRNVSITHISMNNNCLRNIDIDRLSSIT
YFSAAHNKLEFVQLESCEWLQYLNLSHNQLTDIVTGNKEELLLLDLSHNK
LASLHNALFPNLNTLLINNNLLSEIKMFYSNFCKVQTLNAANN QLEKINL
HFLTYLSSIKSLRLDNNKITRIDTENTSDIRSLFPIIKKSESLNFLNISG
ENNCPTIQLMLFNLFSPALKLNTGLAILSPGAFEDHSDGLDVDNELFHYT
INKAYTPYNIHTYKTEEVVNQRNIKIKNMTLDEINNTYCNNDYYNEAIRE
EPIDFLDRSFSSSSWPFYH
> hopAV1 Pph race 6 -1448A|Genbank: AY803996
MSRQRLGKSMIKANSWSSNLNAYRILQEPQS LTEEPPPIQTICPVQPESM
APPEHHNHRHLVTQEENRGRHLVGNPMRRTRSDVGAHGLTPHQPLLTRSK
SDHGLERVATPLAFEVQSVGSEQTASSAEVMTAAGRRHSLLTDYQAAIAR
PLDLDRVLAAVKLRAAAEIYCKKNNLARLSPRPPRTSAGNQRLDALVNRP
VDELIAEVQAAPRKFSRTQLTDVAQRHLLAALNIDIDQYKKGVHKAGLYE
GLHSVAGRLVSVAAAAVSAGVSEVPGVSKTVT KTVKILSHQLPSSVVNPA
LTGGLRSVTDVMESFKRVGGLPVLSPQIDKSPDMGKIVKAAREMRGQLDE
AVGHFARAHKSDPQALPRLVDAFLALHGVADRQYRRRIGLNRTQTYGKGW
GMAVNGVAAIGTVVSTTVPVVGQVAGPAILASTIPLQWGAGYLDERRNKH
RYNLRANTKWGDFLKDNAANIHFKDLTVEHISESKLRQSFLTQPEVQIAA
IREVYEDTIGELMRRRLEMQKDIDVQREAGRPM RALALKQLRELDAEIDV
TKMHADQFESFNMDRWSAIPEDSVIGRCLDDLDNLQKANRRARLRKPGEG
AQIVQRYVQAFQGGVSTGTTLPVVDAVASIDSFHVHDSQGHDHGLQPAPL
AVAAGTGAAGGAVFTGATGEVRMSKADNKKIMTQLRVDPEVYTADAEHWR
FRAGNRDVDLRTSAGYKQHAHTRWDELALVAQALKHGLTSGPVGLKNLAV
AKVELRQARASLQSALDALAASGLPHRTPDSARA PTISSMKDQLYDYSEV
RRHLGV@
> hopZ2 Ppi 895A (race 4A)|Genbank: AJ277495
MGICVSKPSVRHDYNEDYGRNYGADTAQASSNTSSSDSEWEQSPPHSPRQ
PVAADIPDRAKDKISALRESYYRSQMNSNPDLLHYAKNILDNIEYRQSNI
DTLQLDAQNLGSLAAAYNTSFSQLNLHCFDSRIAFLDHLQSHNEPGAWRG
VFRLNPPSLHHVAVDVRNHSNGQKTLIVLEPIT AYKDDVYPPAYLPGYPQ
LREEVNTRLRGNAKMSVIETDAQRSWHDCVIFSLNFALCSYQKDSAFDSL
HEKLAESGYCFPGNEDSRSRLARGIELIDGKQVLPAVFYKHAHSRGTVTA
VENAQPHIANDNVSTNRSSSRETLNERAEAFRVHREGGDPENYSMSIESS
RARKIRKALESQ
> gi|46395936|sp|Q93Q17|AEXT|AERSA ADP -ribosyltransferase toxin aexT (Exoenzyme T) (aexT protein)
MQIQANTVGTQAVAHHSDATTGVGRMGQMEARQVATGQDAILLGSRSEPQ
KGQGLLSRLGAQLARPFVAIKEWISNLLGTDKRAAAPKAQTAVSPEDLQR
LMKQAAFGSSLGGFAKADVLNNITGEQLGKDHASLATGNGPLRSLCTALQ
AVVIGSQQPQLRELATGLLARPIAGIPLQQWGSVGGKVTELLTSAPPELL
KEAMSQLHTAMGEVADLQRAVKAEVA GEPARSATTAAAVAPLQSGESEVN

VEPADKALAEGLQEQFGLEAEQYLGEQPHGTYSDAEVMALGLYTNGEYQH
LNRSLRQEKQLDAGQALIDQGMSTAFEKSTPTEQLIKTFRGTHGGDAFNE
VAEGQVGHDVAYLSTSRDPKVATNFGGSGSISTIFGRSGIDVSDISVEGD
EQEILYNKETDMRVLLSAKDERGVTRRVLEEASLGEQSGHSKGLLDGLDL
ARGAGGADKPQEQDIRLKMRGLDLA
> STM2066 sopA secreted effector protein 2141570:2143918 forward MW:86783
MKISSGAINFSTIPNQVKKLITSIREHTKNGLTSKITSVKNTHTSLNEKF
KTGKDSPIEFALPQKIKDFFQPKDKNTLNKTLITVKNIKDTNNAGKKNIS
AEDVSKMNAAFMRKHIANQTCDYNYRMTGAAPLPGGVSVSANNRPTVSEG
RTPPVSPSLSLQATSSPSSPADWAKKLTDA VLRQKAGETLTAADRDFSNA
DFRNITFSKILPPSFMERDGDIIKGFNFSNSKFTYSDISHLHFDECRFTY
STLSDVVCSNTKFSNSDMNEVFLQYSITTQQQPSFIDTTLKNTLIRHKAN
LSGVILNEPDNSSPPSVSGGGNFIRLGDIWLQMPLLWTENAVDGFLNHEH
NNGKSILMTIDSLPDKYSQEKVQAMEDLVKSLRGGRLTEACIRPVESSLV
SVLAHPPYTQSALISEWLGPVQERFFAHQCQ TYNDVPLPAPDTYYQQRIL
PVLLDSFDRNSAAMTTHSGLFNQVILHCMTGVDCTDGTRQKAAALYEQYL
AHPAVSPHIHNGLFGNYDGSPDWTTRAADNFLLLSSQDSDTAMMLSTDTL
LTMLNPTPDTAWDNFYLLRAGENVSTAQISPVELFRHDFPVFLAAFNQQA
TQRRFGELIDIILSTEEHGELNQQFLAATNQKHSTVKLIDDASVSRLATI
FDPLLPEGKLSPAHYQHILSAYHLTDATPQKQ AETLFCLSTAFARYSSSA
IFGTEHDSPPALRGYAEALMQKAWELSPAIFPSSEQFTEWSDRFHGLHGA
FTCTSVVADSMQRHARKYFPSVLSSILPLAWA
> gi|28867301|ref|NP|789920.1| candidate type III helper HolPtoY
[Pseudomonas syringae pv. tomato str. DC3000]
MNITPLTSAAGKGSSAQGTDKISIPNSTRMINAASIKWLNKV RSAISDHI
RTSIEKGKLFELASLGSNMFGVPALSARPSTLQPVLAFEADPNHDLNLVR
VYMQDSAGKLTPWDPTPNAVTTTSNPSEPDAQSDTASSSLPRRPPAGSVL
SLLGIALDHAQRHSPRADRSAKGRPGREERNGARFNAKQTKPTEAEAYGD
HQTPNPDLHRQKETAQRVAESINSMREQQNGMQRAEGLLRAKEALQAREA
ARKQLLDVLEAIQAGREDSTDKKISATEKNATGINYQ
> Q8X4X1_ECO57
MPLTSDIRSHSFNLGVEVVRARIVANGRGDITVGGETVSIVYDSTNGRFS
SSGGNGGLLSELLLLGFNSGPRALGERMLSMLSDSGEAQSQESIQNKISQ
CKFSVCPERLQCPLEAIQCPITLEQPEKGIFVKNSDGSDVCTLFDAAAFS
RLVGEGLPHPLTREPITASIIVKHEECIYDDTRGNFVIKGN
> gi|41834168|gb|AAS13306.1| VopD [Vibrio harveyi]
MLDKISNTGRADLYGLGELGKTTQAEKTSETRTEAAAIRSSNDSAVTGAK
NYQLDGPKAPATGDQARVVEKLMSALAPTVNLLMQTTEKALNGEQVVKSP
SDTISQSLSLITLLYQVSKLSREQQVLQREIAVEANVASIKSQAEELNNS
AKAMIAMAVVSGVLAGVTAIIGAVGSFKAGKEIKAEVAGNNVLKMQKAGF
DQVEELMGNTNLSKTQQDQVRRAHAFAKDSITDTTNTLSSGGHKFDKIMG
ANQSKNAVLQGLGQMANSAANAEQTKAQARSKEDEVLATRAQADKQRADE
NIGFQEGLLKELRELFRSIADSQNQAWRASIPTV
> A3NLD2_BURP6
MNMHVDMGRALTVRDWPALEALAKTMPADAGARAMTDDDLRAAGVDRRVP
EQKLGAAIDEFASLRLPDRIDGRFVDGRRANLTVFDDARVAVRGHARAQR
NLLERLETELLGGTLDTAGDEGGIQPDPILQGLVDVIGQGKSDIDAYATI
VEGLTKYFQSVADVMSKLQDYISAKDDKNMKIDGGKIKALIQQVIDHLPT
MQLPKGADIARWRKELGDAVSISDSGVVTINPDKLIKMRDSLPPDGTVWD
TARYQAWNTAFSGQKDNIQNDVQTLVEKYSHQNSNFDNLVKVLSGAISTL
TDTAKSYLQI
> Q2LDQ5_PANAY
MWHNINPYGAPPFSNRAPDGEPPAADPPPQGTAGGTPYPEDEVLTEWFSD
CATIGGTDHGVIIDYGRALRRFSGFLSQN GLPALSAPGRLDDPDRLRRDA
HVYIDRFPQPTNKKYLIPSLNRLIDLVTSGADRITIRGPKRPRRTVPDAD
EAQIKAAFPDRDGYSVNLTTSARAYSAWLHANGRPGLSDTDWLLSEQADA
DAVAYSWAHPGYAGRVNIVLAHLRARAGGEVPLIAKNQNTRTIPLADQRL
ARAYRDVAEDRAGACGIVYKKGKDGRDAIDRSITVLRSFSAWRTARGLPA
LTDHLHDPTLLTDAQMFVEEKSQAGRALNT TRRTLYMLNAALELLRASFP
PGTPVTLPEEPADSFALPGSDWSGWDMSLSLPGAEGGPAPADSDSVSGRP
ADSFALPGSDWSGWGMSLSLGGHGPADSDSVFGGLAPLDSRERFSSDGLS
GALASGWPEPGEVNQPGPSGTPFVNMRYATPPFRPDGVGFASGEGLSCLL
DSILQRYHNIRRGPGTPRGLTDWLDGEVRRVREALSAQGVDLLPARGEID

IYGGAGSYLAGAMNVRLQVIQAEFQEEPDGT YVRYTSHPEMGRSDAPLVR
LLHTPGHFQPLWG
> Q8XQK7_RALSO
MNHARQTWEALLDQAVREFAQQQHRGMADYALYEIHLANGYRNHLAEEYG
VAGRVDSFVPPEVGRHLAACKAFVRQRVTAGAMVRRMAEACLGEVHERLK
LYCQRALTADEAWQFYRDYDKSLEAALEMRYGNIPSDVLINPHQGRGTKD
APYSVIETPTLLARAIARNLRQCELLEPFKFLRMAPTPDGHSIKDVGGDD
FYVKMRSQDGEKGYRPLMIADLHKSAWHHGPIAVRKAALMNTTDPGQLKT
LEPEAVWTLIRQHKDPMGWLEDLSHPAVRRYREAEPAHDAFLVAYALATI
RKQSPGAQQRALLQVLRQGERALAEQLCAVVDRADRADAAGNTALHYAAE
HGFDQLLAELLPKSESPDARNVRRETPLMRAARHGKASAVGTLVLAGACV
EAKNARGETALFLAARVGDAASVGVLSRHVTSLDDQAASGKTALMAASEN
GHEGVVTMLLSLRANANRGNNNATTPLIAACRGGHFACAEALVKAGANVN
AHTKDGTTALMAAIDAGHMALVRLLIPKAKLNRFDRHGKTALMCAVARGN
AEGVSALLQANARVDLPKAMLLALKHGHVEVVGVLDNWAKQQAGNPSRR
> gi|21264239|ref|NP|644739.1| avirulence protein [Xanthomonas axonopodis pv. citri str. 306]
MRRSEAERSPPNLL LQSLQEIKMGLCSSKPSVAGSPVAGSPEHYLTHTTE
QTTPSTPSSPEAPMSPSLHGLVALGSSGTRRDRFRQPTLQPHEVQQAAYQ
LGMRLSGRPIEDASDRQRLADATETVHETRLALHRGRGNVDSDLRLSNGR
SATYSSLSYCLGENDENLLAGSALAAGAGNCDHNAAINARRHAVRMEDGG
QMMNVRDYEQTHLYALYQPPSSAEAEESPVVLDSWGDGPAVLLRDSHWAE
TYGTSTNVIERFDKR DAIDALARTNAFRAEIEDPQTDLHANARDLETAFL
ANPAPGDIFSAMPVIAPELAQSTRQRLQEYSPRTRQALAADAARQAYGLD
NAQPISPRTTAAILQDAERLDALGRPPLSW
> Q8X393_ECO57
MAASGGGEITVGGQTVRITYSETDGRFLASGGNNSLLSGLLLTGLNGGPE
ALRDIMLRMVSGSGNTQSHGDIEGKISQCKFSVNTESLQCPSEAVRCPII
LDKPEEGVFVKNSEGSLVCTL FDSVSFSHLVRDGGKHPLTREPITSSMIV
SQEQCIYDQTKGNFVIKDK
> Q8X5C9_ECO57
MDAFIVDPVQGELYSGLSHTELADIIRLADSVENQLNGGNSFLDVFSTYM
GQVISEFMHSNDNRIELLQRRLHSCSFLVNIEEMSYIDEALQCPITLAIP
QRGVFLRNAEGSRVCSLYDEMALSRIINDGMHHPLSREPITLSMLVAREQ
CEFDCSIGHFTVRSDCYSV
> gi|9885643|gb|AA G01466.1|AF282857|15 HrpN [Pantoea stewartii subsp. stewartii]
MSMNTSPLGTSALQVTLGGNNGLMGTDLRTDGLGLLSQPGLGEGKGHNES
IDLLAAALTGMMMMMSMMGGGGLSSLLGSGTGMGNSPFGGSGSAPGNTLS
GTSGGSPGGTTGAGSSLGLDPTQTGDDSLSGAGQTSGMSPMEQLMKIFAD
ITQSLFGDQDGASGGNAGRQPSQDEQNAYKKGVTDALTAF MGGGLSQVAG
NGSEGGLDGGMGLGGGNGLGGKGLQDLSGPADFQQLGNAIGTGVGMKAGI
EALNNIGTHSDSSTRSFINKEDRALAREVGQFMDQYPETFGKPQYQKNAD
SAVKTDTKSWAEALSQPDDDGMTPASMEQFNKAKGIIKSAMAGDNGNINL
QARGAGGSSMGIDATLTGDAINNMALTRLSAA
> gi|21229530|ref|NP|635447.1| avirulence protein [Xanthomo nas campestris pv. campestris str. ATCC 33913]
MRIALLQPAATPTTALATPAHTSAITPMEVPQPPGGNPPLRARPRRQAAV
LPPLVPLDDSAMTGKQALVALDGEFSEQRLAEVQAHQITLQAAQSALAKQ
LPQATPAPRPDSIAARFAAGTLQPVYLDTAAFDEMTASLPEHSRAAAGPV
LVDAQQGRIVFDLGHAFAPGDTFSDAARTALRKALDLRAHGLETPGWLKP
AAPTPAQPRRKLQQAARYHGHEVPARDGGAAFFKPNDHHLVAGKDALLRK
HRKELVHDAYFQAPSTRALGKDVMVHRGLFDNHAGIPENSLAAIDRAYEH
GYRNLELDVEVSADGVPVLMHDFSIGRMTDDPQNRLVSQVPFAQLREMPL
VIRNPVDGNFIKTDQSIAAVEQALEHALQKPEAMSVALDCKEDTGEAVAM
LLMRRPDLRQGAAIKLYAKYYTGGFDQFLSNLYKHYQINPLHSQDAPRRA
ALDRLLAKINVVPVFSQGMLADAQLRDFFPGKDDGPEGLADTAVQWLESW
NRMRPVIVEAVATDQQSAAGKAMELTRTRLRQPDSSYAQAAFSSGYRYED
FSLPRANHDKDYYVWRNFGEMQKLSGEAFGIQRTTAGAFRDAGESLLTDQ
PEEELLALLENRTLARGHTGMELDLPPETPIDSARDAAIVEQRTSEFRAA
SRPADPAHVAAVREGRLLDRSADHSHDAAARQAVDARADALGLLTDQYRG
APVTHYLNEQARQIEPGE
> OspD3_Sflx

MPSVNLIPSRKICLQNMINKDNVSVETIQSLLHSKQLPYFSDKRSFLLNL
NCQVTDHSGRLIVCRHLASYWIAQFNKSSGHVDYHHFAFPDEIKNYVSVS
EEEKAINVPAIIYFVENGSWGDIIFYIFNEMIFHSEKSRALEISTSNHNM
ALGLKIKETKNGGDFVIQLYDPNHTATHLRAEFNKFNLAKIKKLTVDNFL
DEKHQKCYGLISDGMSIFVDRHTPTSM SSIIRWPDNLLHPKVIYHAMRMG
LTELIQKVTRVVQLSDLSDNTLELLLAAKNDDGLSGLLLALQNGHSDTIL
AYGELLETSGLNLDKTVELLTAEGMGGRISGLSQALQNGHAETIKTYGRL
LKKRAINIEYNKLKNLLTAYYYDEVHRQIPGLMFALQNGHADAIRAYGEL
ILSPPLLNSEDIVNLLASRRYDNVPGLLLALNNGQADAILAYGDILNEAK
LNLDKKAELLEAKDSNGLSGLFVALHNG CVETIIAYGKILHTADLTPHQA
SKLLAAEGPNGVSGLIIAFQNRNFEAIKTYMGIIKNENITPEEIAEHLDK
KNGSDFLEIMKNIKS
> gi|16765466|ref|NP|461081.1| putative cytoplasmic protein [Salmonella typhimurium LT2]
MARFNAAFTRIKIMFSRIRGLISCQSNTQTIAPTLSPPSSGHVSFAGIDY
PLLPLNHQTPLVFQWFERNPDRFGQN EIPIINTQKNPYLNNIINAAIIEK
ERIIGIFVDGDFSKGQRKALGKLEQNYRNIKVIYNSDLNYSMYDKKLTTI
YLENITKLEAQSASERDEVLLNGVKKSLEDVLKNNPEETLISSHNKDKGH
LWFDFYRNLFLLKGSDAFLEAGKPGCHHLQPGGGCIYLDADMLLTDKLGT
LYLPDGIAIHVSRKDNHVSLENGIIAVNRSEHPALIKGLEIMHSKPYGDP
YNDWLSKGLRHYFDGSHIQDYDAFCDF IEFKHENIIMNTSSLTASSWR
> hopQ1-2 Pto DC3000|Genbank: AAO58166
MSSPALERSKSAPTLLTSAQREMLAHPVDQYLRDVQKSALKGLWEGIQAG
LLPGLDDSWFFRTFMPNAQIEAAQMDKNKERSFDDIWPKVTELNLYDLLT
LMASVPGAAKLLFKPKATHSEGFGVLEQVGPDDVTHPEKAKLLMSALSKS
ALVQSTAVPD
> gi|12329042|emb|CAC05773.1| o spF, secreted by the Mxi -Spa secretion machinery, function unknown [Shigella flexneri]
MPIKKPCLKLNLDSLNVVKSEIPQMLSANERLKNNFNILYNQIRQYPAYY
FKVASNVPTYSDICQFFSVMYQGFQIVNHSGDVFIHACRENPQSKGDFVG
DKFHISIAREQVPLAFQILSGLLFSEDSPIDKWKITDMNRVSQQSRVGIG
AQFTLYVKSDQECSQY SALLLHKIRQFIMCLESNLLRSKIAPGEYPASDV
RPEDWKYVSYRNELRSDRDGSERQEQMLREEPFYRLMIE
> Q8XYF7_RALSO
MKPVAPVGQSSSAADGVRAPSADAQPGPPAPGPARPARSAAGSSLLQGLA
QLLSLCQPRPARPLPQLPPDIFREIARRSDPLTVQRLRVASKPVKAAIEA
DMRELVIKDRAGLAGVLRAGNYPALEKLTLAGTFTDDDLRGLPASLKALD
LSRCRGSITAAGI AHLSRLPLVRLNVRNKRIGAEGARLLANHPTLTSLNV
SNGRIGPEGAQALAANTRLTTLNVSGNRIGVAGAKALAANQTLRSLDVSD
NRIGDEGARELAACTQLTTLDANRNGIGVDGATALAASRTLTSLAIGGNE
IGDAGVLALAANARLTTLNVESTGVGADGVKALAASKTLTWLRLDGNDIG
NAGATALAASTSLTTLHLEHSRIGAEGAQALAANTKLTTLDLGYNDIGDA
GVRALSANATLVWL SVRRNNLEDASAVSLAAGKTLTTLDISGNGIQDQGA
KALAANPTLTTLDVSSNDIKNAGARALAANARLVSLDLRNNRMEESGTRA
LLANRTLSSLGVSLNCCGQHLIAELMAWANHNGVTMRH
> gi|62868683|gb|AAY17522.1| elicitor of the hypersensitivity reaction [Erwinia amylovora]
MSLNTSGLGASTMQISIGGAGGNNGLLGTSRQNAGLGG NSALGLGGGNQN
DTVNQLAGLLTGMMMMMSMMGGGGLMGGGLGGGLGNGLGGSGGLGEGLSN
ALNDMLGGSLNTLGSKGGNNTTSTTNSPLDQALGINSTSQNDDSTSGADS
TSDSSDPMQQLLKMFSEIMQSLFGDGQDGTQGSSSGGKQPTEGEQNAYKK
GVTDALSGLMGNGLSQLLGNGGLGGGQGGNAGTGLDGSSLGGKGLQNLSG
PVDYQQLGNAVGTGIGMKAGIQALNDIGTHSDSSTRSFV NKGDRAMAKEI
GQFMDQYPEVFGKPQYQKGPGQEVKTDDKSWAKALSKPDDDGMTPASMEQ
FNKAKGMIKSAMAGDTGNGNLQARGAGGSSLGIDAMMAGDAINNMALGKL
GAA
> STM1959 fliC flagellar biosynthesis protein 2047658:2049145 reverse MW:51612
MAQVINTNSLSLLTQNNLNKSQSALGTAIERLSSGLRINSAKDDAAGQAI
ANRFTANI KGLTQASRNANDGISIAQTTEGALNEINNNLQRVRELAVQSA
NSTNSQSDLDSIQAEITQRLNEIDRVSGQTQFNGVKVLAQDNTLTIQVGA
NDGETIDIDLKQINSQTLGLDTLNVQQKYKVSDTAATVTGYADTTIALDN
STFKASATGLGGTDQKIDGDLKFDDTTGKYYAKVTVTGGTGKDGYYEVSV

DKTNGEVTLAGGATSPLTGGLPATATEDVKNVQVANADLTEAKAALTAAG
VTGTASVVKMSYTDNNGKTIDGGLAVKVGDDYYSATQNKDGSISINTTKY
TADDGTSKTALNKLGGADGKTEVVSIGGKTYAASKAEGHNFKAQPDLAEA
AATTTENPLQKIDAALAQVDTLRSDLGAVQNRFNSAITNLGNTVNNLTSA
RSRIEDSDYATEVSNMSRAQILQQAGTSVLAQANQVPQNVLSLLR
> gi|33600601|ref|NP|888161.1| putative outer protein N [Bordetell a bronchiseptica RB50]
MTRIDAAPNPFHAAMQGRHDASANTSSGWLQGQRIAPAPTGISLADAAEE
LSLHMAQAAEEKHHSERKVTAERPMLWLDAAQLAELFSHTHDPDAQAKLE
ALTAELLRGRGAPMQLAAQAFTGVTQQYLALQHALQRGEHEDAAPHALEA
LRDALADLELAHGPEIRAGINTLPTAGAFARSADELAGFQHAYRDIALGQ
LSLARTLDLVLERYGNDDIHGALGALIQA LGHDLAAATPSTDGVRLQVLA
SDLYQVEVAATVLEECNALKQRLGNAGSQECADAQGLMRDLVGISEDKWI
APARFEKLAERHGANALSERIAFLGGVRQILKDLPTQIYADMDVRATVLA
AAQDALDNAIAMENA
> gi|50121144|ref|YP|050311.1| putative virulence effector protein [Erwinia carotovora subsp. atroseptica SCRI1043]
MLATITDYKQRITLIQDSGIQFLDFALKPQFSAEQPNRYVRKSANGPLLH
LLYDEHTDKYLLPSATGMPPEVVKPELSASLDQSLKLLENIWLPLPFFRF
NPPRTFMGGPDNWARMRILALDTPDQDGNTHRICLAFDTKTYPEGHEYES
LAPNANDIKTGGNFALAYHSDELGEFLDETWVDGWLREIFTQQVKAQEKR
DSHDVKVALRGFEYQAHYLNVLDMLGNQLEIPEIRINTSTLQEPAVNVDL
ILDVGNSHTCGILVEDHADESNGLKQTYELQLRDLSEPHYLYNELFESRV
EFSQAKFGKENFSVESGRDDAFIWPSITRVGREASHMALLRQGTEGSSGI
SSPRRYLWDEESYAPGWRFSQTDAHSQTEPLATAMPLTIMLNDEGQPLYN
QPLDERLPVFSPHYSRSSIMTFMLSELLAQALMQMNSAAQRSKMIHSRAP
RQLRNIILTLPSAMPKPEREIFRRRMHEAIALVWKAMDWHPMDEDFTTLA
DKQQSRVPVPEVQIEWDEATCGQMVYLYNEAQVNFGGRAEDFFASMARPD
KELDEGEPAGKTLRIASIDIGGGTTDLAITQYLLDDGVGNNVKIIPRLLF
REGFKVAGDDILLDVIQLYILPALQAALKTAGMASPDALMAKLFGNEGRM
DAQLTLRQQVTLQVFIPIGRAILEAYERFDPLDTSAEIESTFGELLEQAP
TEKVLEYINTEVQRELPVSDTVFDILQVPLILKLNKLHGEFLSNKMNITQ
NLRLMSEVVSLYSCDVLLLTGRPSRFPGIQALFRHLQPLPINRMLSLDGY
HTNDWYPFNKRGRIDNPKSTAAVGAMLCLLALDLRLPGFYFKVGDFQPYS
TVRYLGMMDSSNALTLDNVYYSDIDLDAPDFVLDPKHSFQVRGSLCLGFR
QLDNERWPASSLYMLSIVDQDLARKVVGDSKLRVRLAVTKGDDQDSPERF
EIADAVLEDGTRVPPHHLRLKLNTLSANGSGATHYWIDSGSVFKK
> gi|21233160|ref|NP|639077.1| avirulence protein [Xanthomonas campestris pv. campestris str. ATCC 33913]
MVAAQNHIHPNLDVKVFDASASEPHALRQAIVNTGRGQRWRAVVNVERIH
GKGAPSHGIAVDVSGGRGKVSVLAVDSVWGCADTLSVMTAALKGVKNATL
TILNTGTQKDVINCKTFALANAKAMADNDDLMVDLHKKNFRGKIVGTGDT
VNDVDVTIARGSDVLYVSFFQHTTSKDVFDDLPEHIREPLEESFDQNFRE
IEAGGKRRAYNTSIQQERLKYLRDALLFADAEYWS
> A4SZD3_9BURK
MAVLLGCIADDFTGGTDLAGMLVKSGMKTIQLIGVPTEPAPHDVDAVVIA
LKSRTNPAPEAIEESLRALEWLKSQGCQQFFFKYCSTFDSTPKGNIGPVA
EALMKALDTKFTIACPAFPANKRSIYKGHLFVGDDLLSESGMRNHPLTPM
TDSNLVRVLQQQVQSKVGLTDYSVVKGGAGVIQKRFADLRKDGNQFSIVD
ALSDIDLYSIGAACADLPLVTGGSGIALGLPENFRKKGLLKIIGDASKLG
TVRGLKAVIAGSCSVATQEQVRAMKEKSPAYFVDPLALVDGRDVVTEALE
WVAQKIVKGPVLIYATSSPETVKSIQDKVGIEKAGEMVEHALAQIAKGLI
KLGVGQLIVAGGETSGAVVKALGISGLRIGPEIAPGVPWTKAIFTEVNHD
PLVLALKSGNFGSPQFFNEAWDYIQ
> gi|33600605|ref|NP|888165.1| putative outer protein D [Bordetella bronchiseptica RB50]
MSVSPTSPGSFGAGPVFDSELQAPAPSAQRRGGAAPVPPPVDRRGVEPGD
PTLGMLPAPDLLAGGAVSRTRAALDDLDAARLGEDIYTLMAVLQQASQQM
REAARIARDAEATRQTQALGDAASQMRQAANERMAGAIVAGAMQIAG GFV
QLGAGLAAGLQAMGGAAAQAKGAAFSEQASTSRKVAAGLHDAPELQATVQ
ARATQLEAQAASFGADAARSSAKSQRVSSVAQAGAATAGGIGGLTSAAQE
RRAAEHEARRAELDVEAKVHETASRRADEAMQQMLDIIRGIREKLAGMEQ
SRSETARSVARNI

> Q8XTZ4_RALSO
MRAASPGAPCPSRPWPTHRKAPQLPVWPAGTASHSHASPSGKRPLSHESA
GRHPVSTNAKRAGTMKFDP NYRHRPPLHLVNAGRGHAPAPSKPQGHRAPR
ARALDLPRPARAPAGVAQQDRASPSAVEGALSKALNYPERDLDREAAMQL
DIQREEQQYELWCKSGLGFPERCLDMARDNVLAERNGHGRALVFLMPDSD
AEANIDLLGHYLAKLGDGGPGRVDPALRDGIRACAELCAGYLVHTSWFEG
APVATYAHLANKLSKFPDRAACMEGLAWIARQVLLARHLYRLGDRQATTL
VGGLAKNTRSEACRDAILRL TGGLLGDNAMRGRLNAIQVPMLLNALSKWP
THDEAKSFVLQLADLMAPPSELLHALDGQGVANSLNALSKWPGEARAEKL
ALRLAKRLVADPDLLLSMREQEVTNSLNALSKWAHREEAKQAMLRLAARL
ADEPQLRRAVQPQGVANALNALSKLSEEPQAKQTVLLLADRIATEPRLLQ
ALEAQGVANALNALSKWPGETRAFRAAHLLTERLAADPPLRRALKVQEFK
CTLNALSKWPREDAMKHVALQ LASRIPAEPGLLPALGAQDIANVLNALCK
WPGAEGAKRTVLLLARRLEMEPQLRSALSALELANSLGALAKWPGEAQMG
RTAMQLAERLMAEPKLRQALEAQGVANVLNALSWWSHQDTAKLVAMQLAD
RVAAEPELLKAMDHQQLANTLSALSRWPDEQRAGHAARALVAHLTAEPAG
RWRDLDARHTSNALFALSKWSDENWATQVVLQLAERIATEPALLDDADAQ
GVSDALCALSKWPGEDSARLAT RELAGRLLADTALQAAHDAQRLANTLNA
LAKWQREPEAMRAFWQLSTLPGRASHPWRAFNIVDMAQIGNAFARLVQDE
GEDFERARTVLQRLAIHLELHPECFECSGSGHIGVLFKSFASLRMQRELR
PLGTPALNRVMTLCRETQLRDEPLETVGNLCLGLLPLARSPELARHRVPA
LKVFESLQPVVARKIDSYLRHGVRASAGSALRVMDGQEACGTRGPALTFY
QILKAYALVVRQWKPRYIQGDRQ AVQARQATLKQWVDATLARTRGVIESD
LQEMSWNLIAQIEAGDNVLNALDLRIGRELTAITQRHPPTRFDLAGARTR
MRTEPGKVRPVKAGVGATRHVTVDLQGRELKVDGGAEQPYSFYARLTGQP
LVEVQLPGALSTFMLARTFQYQGEPWRFDLFGGSRLTRGRMRQVKAILAN
NPPLASVLPAMRYADSAPGSAFMTLTHKLAPQREDWSRMQRALLEMVPPD
HVVEGSLRVGWFEDVKVPGTPHPF RLKGPDGTRIALCPDDGCGFMKWEVA
MRIPVVREHVEAWQAVRAKRATPAQQERVAGQEPGANAMPPQALMHYPRD
EAVLAEAHDVMQRRLDRLRAQVQGPGTNVQNAQAPALSPDAVDLLALYQM
TIGGGYEGRRIRAVPSADDKLYLPTIPMDGFARPAGDLLVGKPPYDKENL
LPIAAERVGTAAKGDATAHFLDQCFAIQYSYTGFDDDSGPAADMLHSKGM
LIIPPPGYWSSAHDGMDLACSREDL KVLSRWKQGRDRAGLPDTMLSTGSL
RVKDILMPGRLGALPIPELRKRNMDTDGDDAFVYAGYPKLAAHIRQVMEE
RGGRRKTEYAFKPPKTASPAFDEQGQYQAGRAREILAVQRGGQLVGAASN
AATRFLSQPDALREAMARDMMFGTYDGVDRRLRNGLRARIGGSADAPALG
ELLALAHQGIGRAHLPLAREVALLLHTLTTQLGAAGARPAPPISAELTQR
FGALAEAWTAAPDTHARIHAILDHYP VCRLSHEQFPNGQPGYLEGEPELT
IRNLFTLAVKVGTDALKSDTGTNLFSTLIEQCAVIERTYHDRVRQVPHTK
QTAREFRDGRFDPARAAAALERMPTLAAGVMQDAVGALQQAGLLASPPAP
AERVRTVSPSDIKRTAATLNDQAHAASARITSLLQANLHGWAGPGAGAVR
LAGLKHAVKSAGSLEDKLGYLIAGKQLDLPEAVSQVNDALRYSIVLPSPT
FVPASRRILAGLEEHGHAMTERTNHFA RRGSAFRALSVTLRDPSDGLLWE
VQFHTEQTFELKERYHDLYKQAQRARHQGASFDALRTLLQPAWKDFNAVP
MPAGCDEIDDWQQEPAQAAVPGPQPGPDGQGAQRTIAPYLLPLARRLGAQ
ARRIETLVSPKVQTVLRTCGGKLREDQPGDWRNFLFKKERSIARKIALRQ
RAGQLTPETAAAGVRDSLRYEVVLPAEGFGKTVTAILKMLGKQGLRTMRM
KNAFVQPDTTYAGLNVNLRLAVGNLPGD FEIQFHTAQSLSIKLRGHKDYE
KLRELPSANTHDEHDEAGADFAAERERLLKKMRDAAARVERPQGIETLIP
FDHYRDAQPSGTSRRTG
> Q8XS09_RALSO
MAAPVSTRNAPLPSNPATADTGTPRGPNPSGELAYTPTRSSAGVASSPLG
GLASLRLGPAATSPSSGPRILPPAAHGDPRTAALPRATHLQIHDRRALSG
LRDHPHLESVHLKGDFTLADLKALPTTLRHLDLSECTGSAKSFRAIA YLA
GLPLESLNVAGAEIGDGGARLLAANPSLKSLNAASGGISASGARMLAESP
TLESLDLTQNAIGDAGAQALAGSRSLRHLAVRNGLVTDLGTRALALNPAL
VSLDLGNLVTETGNQVEQDGYDKTANNITAQGAWALAQNRSLKSLSVQGN
DLCGDDGVRALARNRTLTSLNVAFTNMTSASAKALADNPVLTSLSVRWNY
GLDDAGAMELAMSRSLTSLDARDTGMSARGALALAANARISVWHDHAN PV
RAPLGEPPLSGLAGDPGQASRTAPGGAIWSSAAQARHVSAEAPANSAMTA
STREGFALIDPFFNRVARESGLNVQAPQEMPWTRLRPSASEANHGNAGAH
GASAEGLASSRMAASIREGLGLIDQYVDRMGREYGLPVRAPAAQPDSAAP
EATQPTLPADVWQTIAAQADPRVRRTLSTVSKPLRAAALAATKHLTVWDK

AAFSRLRNYPALESLSFHGPLSLADLRALPPSVRHLDLSGCTGSAVSEA G
LAYLARLPLASLDLSDTGIGNRGAQALAASASLTSLNLSGNGIGTAGAEA
LGRNTVLTALDISANPIRNAGAQALAGSRSLTSLELRDTGIEDGGIEALA
ANTVLRSLDISGNDLSDQSAAALAANRTLTSLKANGCGLTNDMAQQLARI
RSLRTLEVGSNSIGDAGVLTIARNASLRSLNLSRNPITPQGLYPLALSRT
LKSLDVSRIGCGDRGALLLSGNRALTSLKLGFNRVSSEGARRLAANRTLV
SLDLRGNTIDVAAARALANAEPLASLNVSDCRLDDAVACALAESRTLTSL
DVSWNRLSHRAARALANNPVLASLYISHNDIGPEGAQALADSASLTFLDA
RANRIGEAGARLLEANTRMRGTPQNPHFLAQDVPE
> gi|15835482|ref|NP|297241.1| hypothetical protein TC0868 [Chlamydia muridarum Nigg]
MTTGVRGDNTPDPSLLAQLTQNANSASASDK NGAAAKTQQQEEASFEDLI
QDSQGTGKSKKASTSQTSKSGKSEKAQKSSGTTTTTSVAQASQTATAQAV
RGARSSGFNNDGSASLPSPSNPNVNGVVLKKNMGTLALMGLIMTLLAQAS
AKSWSSSFQQQNQAIQNQVAMAPEIGNAIRTQANHQAAATELQAKQSLIS
GITNIVGFAVSVGGGILSASKSLGGLKSAAFTNETATAASSATSAAAKTA
VNALDDVANVAATAGTKAASGAASAASSAATK LTQNMTDTASKTLSQTAS
KATGGLFGNALNTPNWSEKISRGLNVVKTQGGRAAQFAGRALSSAMSISQ
MVHGLTAGVDGITGGIIGAQVANEQRAAGMAEARAEELKSLNSVQSQYAS
QAQQLQEQSQQSFNSALQTLQSIADSSLQTTGAMFN
> gi|28871846|ref|NP|794465.1| type III effector HopPtoD2 [Pseudomonas syringae pv. tomato str. DC 3000]
MNPLQPIQHSITNSQMSGGQQLEAEGSQAHNSYSHPDRISLSQLSQSAHL
ALDHLSTQPNTDHQRVASLVRNAVQDGKFQLQSSNDTQVTYKTSVCPPAN
ADTMGAAHLINNELTVQARLNDQLEYDIVSAHLYGPSEAISIDASSPPSA
NDLASSGLSERTHLGMNRVLLRYAVPPRETEDQCVMVIDKMPPPKHGKMS
FFRTTNDLSKLPLGMETGGLSDLKLAGCERISSVEQVKSIRAALGG GPLT
VLDLREESHAIVNGLPITLRGPMDWANAGLSQVDGAARESAMITELKRTK
SLTLVDANYVKGKKSNPQTTELKNLNVRSEREVVTEAGATYRRVAITDHN
RPSPEATDELVDIMRHCLQANESLVVHCNGGRGRTTTAMIMVDMLKNARN
HSAETLITRMAKLSYDYNMTDLGSISALKRPFLEDRLKFLQAFHDYARNN
PSGLSLNWTQWRAKIALE
> gi|12329122|emb|CAC05853.1 | IpaH9.8, secreted by the Mxi -Spa secretion machinery, function unknown [Shigella flexneri]
MLPINNNFSLPQNSFYNTISGTYADYFSAWDKWEKQALPGEERDEAVSRL
KECLINNSDELRLDRLNLSSLPDNLPAQITLLNVSYNQLTNLPELPVTLK
KLYSASNKLSELPVLPPALESLQVQHNELENLPALPDSLLTMNISYNEIV
SLPSLPQALK NLRATRNFLTELPAFSEGNNPVVREYFFDRNQISHIPESI
LNLRNECSIHISDNPLSSHALQALQRLTSSPDYHGPRIYFSMSDGQQNTL
HRPLADAVTAWFPENKQSDVSQIWHAFEHEEHANTFSAFLDRLSDTVSAR
NTSGFREQVAAWLEKLSASAELRQQSFAVAADATESCEDRVALTWNNLRK
TLLVHQASEGLFDNDTGALLSLGREMFRLEILEDIARDKVRTLHFVDEIE
VYLAFQTMLAE KLQLSTAVKEMRFYGVSGVTANDLRTAEAMVRSREENEF
TDWFSLWGPWHAVLKRTEADRWAQAEEQKYEMLENEYPQRVADRLKASGL
SGDADAEREAGAQVMRETEQQIYRQLTDEVLALRLFENGSQLHHS
> Q7AGC6_ECO57
MDCSKCNGYATMLLNMVQGSDPVNLLELHGFLEHFAYYVSFGKFNAGHQR
YNAFKKFVSEISEISANDINMTIKTGQSRHENVISINMNDAIPRDEKGIT
VRIDNINGKKNNSNSSDVFIPYVNTFPDLKNKILRMKIELTEGSGFSKSL
SDSQIEMHILRTVNSLNVGEKLNDDNLADHSIFTNEFSVIIPPSYYDATS
AVNANNIVREKLFESDSKVKDIVDDMSNHDVESEKDIFVIGGMIEKLNSL
ADESFNDSTDNIQTVKDLLTQLTDGMELFALRDIVAFPSTIIAKLIKSPL
NSDHELVMRALDTYLCYFRNKNLNNNAEIINFFHALFLKRPELMVAENYR
FIQFIDLLFENGNVEEKNLAFDLYHNYLSLSEIKQFVTEEIKLNFNEQQG
LLDKDNKCYILLSSDNSGRVMRLSHQALISMLEPEVKKKTIWNNYSIYPS
LQDTHEVVRDDPETICMRAFPLFAKGWEYAQKNKKHQLILNALGFKGYIR
DIFMSAIMRKTDFVLECNNQPTELNSSFSSLMNDSDQWQQHTLKDKHYAN
LLTMLDLNDASESDKSKIFFCLSAVFANISHSNVFNGIPDASKTLKRYAF
ALLAKAHSLDESMISNQTFNTYKTVLLDFNNLSNEEANQLRISSLYRDMV
RYAQYRFSKVLSEWTPDAWL
> Q4ZLM6_PSEU2
MGNICVGGSRMAHQVNSPDRVSNNSGDEDNVTSNQLLSVRRQLAESAGLP
RDQHEFISSQAPESLRSSYNNLYSHTQRTLDFADMQHRFMTGASGINPGM

LPRENVDDMRSAISDWSDMREALQHAMGIHADIPPSPERFVTTINPSGSI
RMATLAPSPHRNW
> gi|53722552|ref|YP|111537.1| putative cell invasion protein [Burkholderia pseudomallei K96243]
MSIGVQSSGINISHAELSRLVDAGKSEQGDKAVRDDGRALARADAALAAV
VGERVAARRDAVAGSGAQRVELARPKPDAQTRATDRRTVSGLEREHKRLA
ASQTPRVTGMHDALVQRHVSLDGAKAAHGEGVKRAAGDAPRAAADAPQRF
AFADDKAFDAMLALGAAMQKNVQSDLAMQGKLTMLAHDAMMSAAAQDRSI
GAAQMTAAIAGGALQATTSLGGAMQQMKSLSTKSMSIEKELKPQAELKQF
HAEQALELRGINKPVLSNDEVSHVKIKRDTGETVRHEIDHGGERMSDEHA
SVLAQEAPARQHRIDMHGMRHEENLVKAGRQQMKGDLLQSGGQIGKNQID
GASAQQQGADRAEQKEDENAQQTAMAAASTRDEAAHRSREAAQKAIDAAK
SQVANDNAVAAQVAGNLRT
> SOPE_SALDU
MTKITLFPHNFRIQKQEATPLKEKSTEKNSLAKSILAVKNHFIKLNSKLS
ERFISHKNTESSATHFHRGSASEGRAVLTNKVVKNFMLQTLHDIDIRGSA
SKDPAYASQTREAILSAVYSKYKDQYCNLLISKGIDIAPFLKEIGEAAQN
AGLPGATKNDVFSPSGAGANPFITPLITSAYSKYPHMFTSQHQKASFNIY
AEKIIMTEVVPLFNECAMPTPQQFQQILENIA NKYIQNTP
> gi|15723942|gb|AAL06389.1| EspG [Citrobacter rodentium]
MILVIKIFVIDETERAFMLNGLNNNSASLVLDATIKINSDYKKPWNEMTC
AEKLLKILTLGLWNPKYSQDERQQFQGLLTVLEPVSPAHNELGRVYAKFS
DGSSLRISVTNSELIEAEIHTPNNEKFLVLLEANEQNRLLQSLPINRHMP
YIQVHHTLPQEELTDLLSMHKLLSFTSKLSATLIPH NNQTDPLSGLTPFS
TVFMDTSRGLGNSKLSLNGVDIPADAQKLLRNTLGLKDTNSSPDLNVIRN
GIPRHYAEQIVKESSSTNEQKAAVVDFLCQPEAPTAICSAFYQSFNVPAL
MLTHVRISQASAYNAQRSLDMPNACINISITQSSEGSIHVTSHTGVLIMA
PEDRPNQLGMLTNRTSYEVPPGVKCEPNEMARMLKAKYASSETYLNNA
> A1B7G2_PARDP
MLLGAIADDLTGATDLSLILSRAG MSVIQVVGVPDEGDDFGNADAVVVAL
KSRTIPAAEAVEQSLASARVLRARGARQLYFKYCSTFDSTAEGNIGPVTD
ALLDLIGETRTVACPAFPQNGRTVYQGHLFVGSQLLSDSPMKDHPLTPMR
DADLRRVLAAQTARPVDLVAHATVAQGAEAVAAALAGAQGIAITDAICNA
DLMTLGKALAGYGLVTGGSALALGLPQNFRAAGLLAEDAGNADFTAPKGP
GIMLAGSCSAATRQQVARAEANGIA TLALDPLAIASGETRIEDALAFIAA
HAGDPRPPLLYSSADPEAVAQAQEKLGRAQAGVVVEAFLSGVARKLAEAG
TRRFIVAGGETSGAVVEALEAMAVRIGPEIDPGVPWVVSTDPVAPMALAL
KSGNFGCEDFFAKAWDMLA
> gi|15605406|ref|NP|220192.1| S/T Protein Kinase [Chlamydia trachomatis D/UW-3/CX]
MLRSGVSFSSSKTNYLLTRELSRK VGLTVYQGVDEHSSRPVVIKTLVSPG
IHDRRFLRAFEEEARIMQLVTHPAFVRLEDRGECEQGRYLVSEYILGSSL
RDSILSSQISLDKAISIVLQVAQAITTLHRHGVLHLDIKPENIVLSQSGE
IKLIDYGLSAWQFNHWGSPAYMSPEQSRQEPPSPASDVYSLALLAYELIM
GQLALGKVYVSLLPSKISKILIQALQPSPAARFSSMQEFAEALQDYLLHD
VHEDYRKKDHVVAQIEQWHNQRAWL SPEKLSAPEEICVHIYSQKEPCYLH
NIYYDMLTSGNVAEFWFCYAPGNCSFALSMMKQFLNQREEKAKDIQTVIK
SMDTLCKTMHIPICEEGISCCCFIFFLEELMCFSCGKTDFSLKKQTGGGQ
RFQAESQGIGEETPLEIHEQSFLWEPGDELIVHTPKARDLVYLYCPSFLK
LQDRGQIDIFCQTDNLQKGIRQKYDRSLYPSTLISLKRVR
> Q4ZX85_PSEU2
MHINRTGSSQPSIELERFYSA SQSLASSSVRELSPAEHANLQAITDYLKD
HVFAAHKLPLSESAVDQDAVHAHNEQLDKIIDARARRLLDEGETPATIAD
TFAKAEKFDRMATTASITLRATPFAAASVLQYMQPAINKGDWLPMPLKPL
TPFVSGALSGIMDQVGTTVMNRATGNLHYLSTPPEKLHDAMAASVKRHSP
GVMRQAVDLGIAIQAYSARNAVRTVLAPALASRPAVQGAVDISVSAAGGL
AANAGFGNRMHSVQARDHLRGG AFVLGIKDQEPKADLSEETDWLVAYKAI
KSASYSGAALNAGKRVAGLPLDVATDGLKAVRSLVSATGLAQNGLALAGG
FAGVGKLQEMATKNITDPATKAAVSQLTNMAASTGVFAAWTTAAVATDPA
VKKAESFLQDTVKSAASSSTSYVADQTVKLAKAGIDASGEAIAATGASLL
DTLRRRNAREPDIEEGGIAAGSPSAVPFQPGRS
> Q4ZXP9_PSEU2
MTDLNSLRTSLSSGEHAFADTLAFI ADGYDYQPQAFRNGDVDNAAGQNEG

SCKTLGLALLEGLTDEEALLAFGEHYRSVQATPEGSDHGNIRALMANGLA
GVKFAGEPLKRKA
> hopD1 Psp race 6 -1448A|Genbank: AAZ37970
MNPLRSIQHNITTPPISGGQPLDAVGPQAQQSHPKRISPSQLSQSAHQAL
ERLSANAEHQRLASLVRNALQDGTFQFQSSNHTQVTYKASICLPADTDTV
RTDHLINNELTVQARLNDQS EYDIVSAHLHGSSKAISFDVPSPPPAHGSA
SSVLSERTHLGMSSVLSQDAVDSSSLETPLVSSPDHSRPPSQPKPVHIGS
VRRDSGSLVSDNPVVQALLSFVQADQAFPPQAASIAGVQLEMRSRRDIEQ
ALEELKGAFTVEKAQLMSGGSSSERVDEDVNADIHIPLLLKAIERGAGAF
GPGALIEIADGGQISAKAFLASCAPTITSNDDVLSEFINQKLKGDDDLQV
RLGAQELLHVATKKEFQLGGL AGSIGVSSILGSAWELGASELLKNAIFGK
NFSPSQYALQLAGIDSVPPFIIEAMDSFCVLVIIKGMKGELWSMKDLLPK
ALKAGAISSAMSFPNNVLQYAGFKSRVADLAANSITTEAAIFGAASGIPP
EVKESEELMRAGLFQSMKDGVMAHPGEGMDTKETIERMTRHALDIAPGES
TAVKSMGLAAIVGMIPLIASSKATGLVSEQILRIFRNAVFNPIEAIALNA
LALGGRVNVPGLFDSDNAKHAR VAQTILARASQHMEAGDREISAEELHQM
LAPRSEFLRHVGSAIVNGMNASFEAIPALVRKLGYGEAPLAERIPYQDLA
VPDTSRQPAP
> gi|12329059|emb|CAC05790.1| OspC1, secreted by the Mxi -Spa secretion machinery, function unknown [Shigella flexneri]
MNISETLNSANTQCNIDSMDNRLHTLFPKVTSVRNAAQQTMPDEKN LKDS
ANIIKDFFRKTIAAQSYSRMFSQGSNFKSLNIAIDAPSDAKASFKAIEHL
DRLSKHYISEIREKLHPLSAEELNLLSLIINSDLIFRHQSNSDLSDKILN
IKSFNKIQSEGICTKRNTYADDIKKIANHDFVFFGVEISNHQKKHPLNTK
HHTVDFGANAYIIDHDSPYGYMTLTDHFDNAIPPVFYHEHQSFLDKFSEV
NKEVSRYVHGSKGIIDVPIFNTKDMKLGLGLYLIDFIRKSEDQSFKE FCY
GKNLAPVDLDRIINFVFQPEYHIPRMVSTENFKKVKIREISLEEAVTASN
YEEINKQVTNKKIALQALFLSITNQKEDVALYILSNFEITRQDVISIKHE
LYDIEYLLSAHNSSCKVLEYFINKGLVDVNTKFKKTNSGDCMLDNAIKYE
NAEMIKLLLKYGATSDNKYI
> gi|27377015|ref|NP|768544.1| hypothetical protein blr1904 [Bradyrhizobium japonicum USDA 110]
MEILMNTGQAQWSAGSSSAASEEGEQESYLSGFSRAVADLLEAMTGYRTP
ASARAQLLDNWAAEEGQGEAENRRQGRARIRGADNTGSHSLDLSSLSLTA
LPAALPPRLLELRARHNELSSLPASFPPGLQHLLISHNRLISLPDALPAT
LSRLEVADNSLTSLPANLPAGLEILNASDNRLTSLPDALPSRLTSLAVSG
NQLTDLPDYLPSGLIELDVSSNQLADLPAPLPST LQSLNLSGNRLTSLPE
DLLEGRVPNLSLSYPHLENLEFGDNPLPDDVLANLATAEAPPQLAQQSLY
EAAAQWLADDPATLAKWQHFADEPGAQDYAQFLERLRETVNYGNDEFRQA
VADDLQQAAANPTLREQFFAQASEANASCEDRVTLHWNGMQTARLNADVE
DGVYDDRLGDLIQRGRVAFRLEALDEIARDRINSLASGNPNVDDIEVYLA
YQHRLREPLELSHVAPDMRFLAVSHVSEDDATEAL DLVREREASQFTDFM
ASRWQPWETVLRRIAPDEHAAMQDRLVEAMGEEFQSRLDQRLAEHGLVGD
VDAERVLGAQVRNQIAGEIKGEVMHRLLAERGLEQ
> Q8X4Q6_ECO57
MPVNATGVSFSSFGISYHKDNSFRGTIRGKNDEVVKCSMGERSIRFNVNK
FSGCILETVSRQSTKDIHGWVSDERTVYPSRVINQEIDNCCLQKNAKISS
EERKMVFSLVSKEFELTLDVKAAQSSINHIIIGNAS FGKKMDALCDGMSR
AVKNSTTDYIANVLADKFYQKHIAPGVDIVKLRNEIPGYMSRVIQG
> gi|57434478|emb|CAI43895.1| EspG protein [Escherichia coli]
MISVVESFIINEIGESLMINGLNNNSASLVLDAAIRINSDFKKQWNDMSC
AEKLLKVLSFGLWNPTYTRSERQTFQELLTVLEPVSPAPNELGRIYANFA
DGSSLRISVTNSELVEAEIRTPDNEKILML LESNEQNRLLQSLPINLHMP
YIQVHRALSKMDLTDHKSMHNLLSFTSKLSATLIPHNTQTDPFSGPTPFS
SMFMDTFRGLGNAKLSLNGVDIPVDAQKLLRDALGLKDTHSSLARNVINN
GISRHHAEQIARESSGSDKQKAEVVEFLCHPEAATAICSAFYQSFNVPAL
MLTHTRISQAREYNVERSLDVPNACINISISQSPDGSIHVASHTGILIMA
PEDRPNELGMLTNRTSYEVPQGVKCEIDEMV RTLQPRYGASETYLKNI
> Q8Y2V5_RALSO
MSSRISGSWGRYWPDLSSQSDERDEKMPAVSKAQAREVYEHSVLFHGTTL
RSRRSIQRNGFRLENKSAGSITAYAGIQSNFDAAVASSSHHYFAEKRRLR
SASPEWSAVIAAIAPHWFVP
> Q12HJ4_POLSJ

MQASHKDSTLKIAYYGDDFTGATDTLATAARAGLRTLLFLGLPTATQLER
AGPLDCLGIAGAARAMTPAEMQLELEPVGRLFAA LGAPVMHYKTCSTFDS
APHIGSIGAALRILRPHAGNALVPIVGGQPNLHRYCVFGNLFAGTGNGGA
IHRLDRHPTMRQHPVTPMHEADLRLHLAQQGLENLSAIAYPDYAQLDEAL
DRQLDALLAQGPDAVLFDIAHPDNLAAVGRLIWQHARRQRLLAAGPSSVV
QALAAHWQSGAGPARTAIAPAQGPVLVLAGSLSPLTARQVKAATSYEHIA
LDAPRLAGDENYRMQMARQIATRLDQGAHVLACTS GADGATVSTSSSTSS
SPTDGRALAQACGDLLAHVLATRPIRRLGIAGGDTSSHAVQALQAWGLSY
RAPLAPGVALCRLHSEQASLDGLEIMLKGGQMGGETLFEELLAGTPLPV
> gi|16766189|ref|NP|461804.1| translocation machinery component
[Salmonella typhimurium LT2]
MLNIQNYSASPHPGIVAERPQTPSASEHVETAVVPSTTEHRGTDI ISLSQ
AATKIHQAQQTLQSTPPISEENNDERTLARQQLTSSLNALAKSGVSLSAE
QNENLRSAFSAPTSALFSASPMAQPRTTISDAEIWDMVSQNISAIGDSYL
GVYENVVAVYTDFYQAFSDILSKMGGWLLPGKDGNTVKLDVTSLKNDLNS
LVNKYNQINSNTVLFPAQSGSGVKVATEAEARQWLSELNLPNSCLKSYGS
GYVVTVDLTPLQKMVQDIDGLGAPGKDSKLEMDNAKYQAWQSGFKA QEEN
MKTTLQTLTQKYSNANSLYDNLVKVLSSTISSSLETAKSFLQG
> Q3BRD8|Q3BRD8_XANC5 Xanthomonas outer protein N - Xanthomonas campestris
pv. vesicatoria (strain 85-10).
MKSSASVDSASRSAGSHLPEIQETELATASTSAAVTHQSAPDPALSPLGR
APVRRSGANTLGALLARPEQDAQALVPQPVTSSNATKATLARLRQQLAS D
NARAIAPDLAADLISRLRPMKSPGTTGAQARAATHADLVSRISEHDVIDW
YKAQGLNENDVATLRRTALLSGMPNPSGSFLTNVMQYIVSPWINYATRQP
WAGAGFGLATMLVAAPVNAGQQSAVVSLCESIREHGAHVIVPDKKQINDK
HWLPELAHALKEKIETFAKACDVFREIQSDPNSSSKERAAAADVLLAAEK
ELHQAQHDFVMTQGAHDRQCQGNRRQAVPRIMRSPVASTLGLLSKTGAMR
ALSPTAQTVGAMLMTAAQHVAAGFDEQAKQEANNKLNLLYADVLTDAGKQ
KLASGAAVSAEDIKTDKLRSLIQSPTQALVKRVTAGVAELEKLLADAVTA
EGTQPGAITADDRDIEAGDASKAMQELRLVRNDLEALREGRLDEIDPNGT
ASKLLIGAEKSVLSQQLFNDIAKKYNYLEFTAQTAQRIGQMFHLIFLGSA
ASSVISKLVSASQGGTRNVPLPQSLAVSAISGGMAAVGALNQHTAITIKN
NRREGSTDIGLGKQLGRGVLGAMYETFSQRRGTKASKAINALLEGNDVEA
LLATARALSVQEASTSSSQPQDLPSTSRAAPPRREHVSAPSSPNRSRLEP
TNNEITRSKSARNLGTELHEFARMAGKHSKHLV
> gi|12329105|emb|CAC05836.1| VirA, secreted by the Mxi-Spa secretion
machinery, function unknown [Shigella flexneri]
MQTSNITNHERNDSSWMSTVKSTTEVSWNKLSFCDILLKIITFGIYSPHE
TLAEKHSEKKLMDSFSPSLSQDKMDGEFAHANIDGISIRLCLNKGICSVF
YLDGDKIQSTQLSSKEYNNLLSSLPPKQFNLGKVHTITAPVSGNFKTHKP
APEVIETAINCCTSIIPNDDYFHVKDTDFNSVWHDIYRDIRASDSNSTKI
YFNNIEIPLKLIADLINELGINEFIDSKKELQMLSYNQVNKIINSNFPQQ
DLCFQTEKLLFTSLFQDPAFISALTSAFWQSLHITSSSVEHIYAQIMSEN
IENRLNFMPEQRVINNCGHIIKINAVVPKNDTAISASGGRAYEVSSSILP
SHITCNGVGINKIETSYLVHAGTLPSSEGLRNAIPPESRQVSFAIISPDV

> gi|2897963|gb|AAC38401.1| SepZ [Escherichia coli]
MEAANLSPSGAVLPLAATINGNNPVDEKTGVMQSEGGTSRSVRILGGVLI
GAGVLAAIGTGIAAMCVDDPSQRLGLGIAAGVLGGVTTVAGGLAMKYA
> gi|57434460|emb|CAI43877.1| SepZ protein [Escherichia coli]
MSKLDNGEKLMDAANLSPSGRVLPLAATINGNNPVDETTGVMQPENGTNR
TIRILGGVLISAGALAAIGAGIASLCVEDPSQKLGLGIAAGVLGGVTTLG
SGLAMKYA
> gi|12329039|emb|CAC05770.1| OspB, protein secreted by the Mxi-Spa
secretion machinery, function unknown [Shigella flexneri]
MNLDGVRPYCRIVNKKNESISDIAFAHIIKRVKNSSCTHPKAALVFLGEK
GFCDSNDVLSIMGQQIPRVFKNKMLYDYVFKNEKSKNDFLKMAESWLPQS
EPIVINNDDDALNAAAYFSVKKAKIKTVNDTDFKEYNKVYILGHGSPGSH
QLGLGSELIDVQTIISRMKDCGILNVKDIRFTSCGSADKVAPKNFNNAPA
ESLSCILNSLPFFKEKESLLEQIKKHLENDESLSDGLKISGYHGYGVHYG
QELFPYSHYRSTSIPADPEHTVKRSSQKKTFIINKELD
> hopAA1 Psy B728A|Genbank: AAY36237
MATPTNSWNPRHCCDHTALSVLFADGPPDVCNCYHSRTDASLPCLFASKS

SGQTRLGALLRNLSNREGVSMHINRTGSSQPSIELERFYS ASQSLASSSV
RELSPAEHANLQAITDYLKDHVFAAHKLPLSESAVDQDAVHAHNEQLDKI
IDARARRLLDEGETPATIADTFAKAEKFDRMATTASITLRATPFAAASVL
QYMQPAINKGDWLPMPLKPLTPFVSGALSGIMDQVGTTVMNRATGNLHYL
STPPEKLHDAMAASVKRHSPGVMRQAVDLGIAIQAYSARNAVRTVLAPAL
ASRPAVQGAVDISVSAAGGLAANAGFGNRMHSVQARDHLRG GAFVLGIKD
QEPKADLSEETDWLVAYKAIKSASYSGAALNAGKRVAGLPLDVATDGLKA
VRSLVSATGLAQNGLALAGGFAGVGKLQEMATKNITDPATKAAVSQLTNM
AASTGVFAAWTTAAVATDPAVKKAESFLQDTVKSAASSSTSYVADQTVKL
AKAGIDASGEAIAATGASLLDTLRRRNAREPDIEEGGIAAGSPSAVPFQP
GRS
> gi|15618717|ref|NP|225003.1| hypothe tical protein CPn0808 [Chlamydophila pneumoniae CWL029]
MTSGVSGSSSQDPTLAAQLAQSSQKAGNAQSGHDTKNVTKQGAQAEVAAG
GFEDLIQDASAQSTGKKEATSSTTKSSKGEKSEKSGKSKSSTSVASASET
ATAQAVQGPKGLRQNNYDSPSLPTPEAQTINGIVLKKGMGTLALLGLVMT
LMANAAGESWKASFQSQNQAIRSQVESAPAIGEAIKRQANHQASATE AQA
KQSLISGIVNIVGFTVSVGAGIFSAAKGATSALKSASFAKETGASAAGGA
ASKALTSASSSVQQTMASTAKAATTAASSAGSAATKAAANLTDDMAAAAS
KMASDGASKASGGLFGEVLNKPNWSEKVSRGMNVVKTQGARVASFAGNAL
SSSMQMSQLMHGLTAAVEGLSAGQTGIEVAHHQRLAGQAEAQAEVLKQMS
SVYGQQAGQAGQLQEQAMQSFNTALQTLQNIADSQTQTTSAIFN
> A0VG66_COMAC
MTLLLGCIADDFTGATDLANNLVRAGMRVVQSIGVPAGPVDEGVDAVVVA
LKSRTIAPADAIAQSLQALQWLQAQGANGRAPQIYFKYCSTFDSTPQGNI
GPVADALMDALGCEFTIATPAFPDNQRTVFKGHLFVGDVLLSDSGMRNHP
LTPMTDANLVRVLQAQTGRKVGLIDHVAVAQGEAAIRARIAALQAEGVGM
AVVDAVSNDDLLRLGPALAGMPLVTAGSGVAIGLPANFGI QPTAQAAALP
TATGLQAVVSGSCSAATNAQVAHFIASGRPALALEPLRIAAGEDEAGKAL
AWARQHIASGPVLIYSTAESSAVKAVQGHLGVEQAGALVEQTIAAIARGL
VEMGVRQLVVAGGETSGACVQALGIEQMRIGPQIDPGVPWCHAASPLAPQ
GLHLTLKSGNFGTTDFFTKAFGALASA
> gi|15618924|ref|NP|225210.1| hypothetical protein CPn1016 [Chl amydophila pneumoniae CWL029]
MKKGKLGAIVFGLLFTSSVAGFSKDLTKDNAYQDLNVIEHLISLKYAPLP
WKELLFGWDLSQQTQQARLQLVLEEKPTTNYCQKVLSNYVRSLNDYHAGI
TFYRTESAYIPYVLKLSEDGHVFVVDVQTSQGDIYLGDEILEVDGMGIRE
AIESLRFGRGSATDYSAAVRSLTSRSAAFGDAVPSGIAMLKLRRPSGLIR
STPVRWRYTPEHIGDFSLVAPL IPEHKPQLPTQSCVLFRSGVNSQSSSSS
LFSSYMVPYFWEELRVQNKQRFDSNHHIGSRNGFLPTFGPILWEQDKGPY
RSYIFKAKDSQGNPHRIGFLRISSYVWTDLEGLEEDHKDSPWELFGEIID
HLEKETDALIIDQTHNPGGSVFYLYSLLSMLTDHPLDTPKHRMIFTQDEV
SSALHWQDLLEDVFTDEQAVAVLGETMEGYCMDMHAVASLQNFSQSVLSS
WVSGDINLSKPMPLLGFAQVRPH PKHQYTKPLFMLIDEDDFSCGDLAPAI
LKDNGRATLIGKPTAGAGGFVFQVTFPNRSGIKGLSLTGSLAVRKDGEFI
ENLGVAPHIDLGFTSRDLQTSRFTDYVEAVKTIVLTSLSENAKKSEEQTS
PQETPEVIRVSYPTTTSAS
> STM1960 fliD flagellar hook -associated protein 2049402:2050805 forward MW:49835
MASISSLGVGSNLPLDQLLTDLTK NEKGRLTPITKQQSANSAKLTAYGTL
KSALEKFQTANTALNKADLFKSTVASSTTEDLKVSTTAGAAAGTYKINVT
QLAAAQSLATKTTFATTKEQLGDTSVTSRTIKIEQPGRKEPLEIKLDKGD
TSMEAIRDAINDADSGIAASIVKVKENEFQLVLTANSGTDNTMKITVEGD
TKLNDLLAYDSTTNTGNMQELVKAENAKLNVNGIDIERQSNTVTDAPQGI
TLTLTKKVTDATVTVTKDDTKAKEA IKSWVDAYNSLVDTFSSLTKYTAVE
PGEEASDKNGALLGDSVVRTIQTGIRAQFANSGSNSAFKTMAEIGITQDG
TSGKLKIDDDKLTKVLKDNTAAARELLVGDGKETGITTKIATEVKSYLAD
DGIIDNAQDNVNATLKSLTKQYLSVSNSIDETVARYKAQFTQLDTMMSKL
NNTSSYLTQQFTAMNKS
> gi|28871726|ref|NP|794345.1| type III effector HopPtoS3 [Pseudomonas syringae pv. tomato str. DC3000]
MNISPVSGAHGSSYPSAQSTASTASKGPSGSFLKQLGGCFSPCLGSSSTG
AILSPAHEQVLSHTYSSNIKGKLRTTPPKGPSPRLSDTPMKQALSSMIVQ

ERKRLKSQPKSLASDIERPDSMIKKALDEKDGHPFGERFSDDEFLAIHLY
TSCLYRPINHHLRYAPNNDVAPVVEALKSGLAKLAQDPDYQVSSQLHRGI
KQKMSDGEVMSRFKPGKTYRDEAFMSTSTHMQVSEEFTSDVTLHLRSSSA
VNIGPFSKNPYEDEALISPLTPFKVTGLRKQDDKWHVDLNEIADNSDE
> gi|21264224|ref|NP|644725.1| avirulence protein [Xanthomonas axonopodis pv. citri str. 306]
MDPIRSRTPSPARELLPGPQPDGVQPTADRGVSPPAGGPLDGLPARRTMS
RTRLPSPPAPSPAFSAGS FSDLLRQFDPSLFNTSLFDSLPPFGAHHTEAA
TGEWDEVQSGLRAADAPPPTMRVAVTAARPPRAKPAPRRRAAQPSDASPA
AQVDLRTLGYSQQQQEKIKPKVRSTVAQHHEALVGHGFTHAHIVALSQHP
AALGTVAVKYQDMIAALPEATHEAIVGVGKQWSGARALEALLTVAGELRG
PPLQLDTGQLLKIAKRGGVTAVEAVHAWRNALTGAPLNLTPEQVVAIASN
IGGKQALETVQALLPVLCQ AHGLTPEQVVAIASHDGGKQALETVQRLLPV
LCQAHGLTPEQVVAIASNIGGKQALETVQRLLPVLCQAHGLTPEQVVAIA
SHDGGKQALETVQRLLPVLCQAHGLTPEQVVAIASNIGGKQALETVQRLL
PVLCQAHGLTPDQVVAIASHDGGKQALETVQRLLPVLCQAHGLTPDQVVA
IASHDGGKQALETVQRLLPVLCQAHGLTPQQVVAIASNGGGKQALETVQR
LLPVLCQAHGLTPEQVVAIA SHDGGKQALETVQRLLPVLCQAHGLTPEQV
VAIASNGGGKQALETVQRLLPVLCQAHGLTPEQVVAIASNGGGKQALETV
QRLLPVLCQAHGLTPEQVVAIASNGGGKQALETVQRLLPVLCQAHGLTLD
QVVAIASNGGGKQALETVQRLLPVLCQAHGLTPEQVVAIASNIGGKQALE
TVQRLLPVLCQAHGLTPEQVVAIASNIGGKQALETVQRLLPVLCQAHGLT
PEQVVAIASNGGGRPALESIV AQLSRPDPALAALTNDHLVALACLGGRPA
LDAVKKGLPHAPALIKRTNRRIPERTSHRVADHAQVVRVLGFFQCHSHPA
QAFDDAMTQFGMSRHGLLQLFRRVGVTELEARSGTLPPASQRWDRILQAS
GMKRAKPSPTSTQTPDQASLHAFADSLERDLDAPSPMHEGDQTRASSRKR
SRSDRAVTGPSAQQSFEVRVPEQRDALHLPLSWRVKRPRTSIGGGLPDPG
TPTAADLAASSTVMREQDEDPF AGAADDFPAFNEEELAWLMELLPQ
> Q3BM44|Q3BM44_XANC5 Xanthomonas outer protein Q - Xanthomonas campestris pv. vesicatoria (strain 85 -10).
MQPTAIRSTVGLPGADMTADLRDPAPVAVPAHSAADAAAPPPGALQTIVG
RPPRPDGPRHRRAQSLPARLTPAQRGMLAELGVADTSVLTPTETAVLREL
RLHRPPLPLDTLLFTDPNKDPD DVVTYTIAKQLQAEGFLRLTDVVVTLGD
ADMRSQRAQLAKGVFDRLALPEVRVARGQDYPMTSTQAREHSKFLAEGAA
LRAAPDAVHTDGVRAMRERLATSPHKLGMVVIAGMTDASALLAEAGDLVR
EKLASITIMGGIDPARDADGLVQPDTRAYNNATDIHAARALYRRAQQLGI
PLRILSKEAAYRAAVPPAFYEGIARNGHPVGEYLRDVQKNALKGLWEGIQ
ANLIPGLDTAWFFRTFVAAQPQD PAAADQQGAMSFDAIWPQVTKLNLYDP
LTLLAALPGAARLLFQPTPMHREGASPVEHVGHAEVVRPEKARLLLSALA
KAALAQQDEGQRGR
> SIPD_SALPA
MLNIQNYSASPHPGIVAERPQTPSASEHAEIAVVPSTTEHRGTDIISLSQ
AATKIQQAQQTLQSTPPISEENNDERTLARQQLTSSLNALAKSGVSLSAE
QNENLRSTFSAPTSALFSASPMAQPRTTISDAEIWDMVSQNISAIGD SYL
GVYENVVAVYTDFYQAFSDILSKMGGWLSPGKDGNTIKLNVDSLKSEISS
LINKYTQINKNTILFPSQTGSGMTTATKAEAEQWIKELNLPDSCLKASGS
GYVVLVDTGPLSKMVSDLNGIGSGSALELDNAKYQAWQSGFKAQEENLKT
TLQTLTQKYSNANSLYDNLVKVLSSTISSSLETAKSFLQG
> gi|16764750|ref|NP|460365.1| translocation machinery com ponent [Salmonella typhimurium LT2]
MNRIHSNSDSAAGVTALTHHHLSNVSCVSSGSLGKRQHRVNSTFGDGNAA
CLLSGKISLQEASNALKQLLDAVPGNHKRPSLPDFLQTNPAVLSMMMTSL
ILNVFGNNAQSLCQQLERATEVQNALRNKQVKEYQEQIQKAIEQEDKARK
AGIFGAIFDWITGIFETVIGALKVVEGFLSGNPAEMASGVAYMAAGCAGM
VKAGAETAMMCGADHD TCQAIIDVTSKIQFGCEAVALALDVFQIGRAFMA
TRGLSGAAAKVLDSGFGEEVVERMVGAGEAEIEELAEKFGEEVSESFSKQ
FEPLEREMAMANEMAEEAAEFSRNVENNMTRSAGKSFTKEGVKAMAKEAA
KEALEKCVQEGGKFLLKKFRNKVLFNMFKKILYALLRDCSFKGLQAIRCA
TEGASQMNTGMVNTEKAKIEKKIEQLITQQRFLDFIMQQTENQKKIEQKR
LEELYKGSGAALRDVLD TIDHYSSVQARIAGYRA
> gi|15605404|ref|NP|220190.1| hypothetical protein CT671 [Chlamydia trachomatis D/UW -3/CX]
MELNKTSESLFSAKIDHNHPRTEAHEPRDQREVRVFSLEGRSSTRQEKAD

RMPGRTSSRQESSKGSEEGAVHESTAGVSSKEEEESKGDGFFTGGNPTSG
MALVETPMAVVSEAMVETSTMTVSQVDLQWVEQLVTSTVESLLV ADIDGK
QLVEIVLDNSNTVPAAFCGANLTLVQTGEEISVSFSNFVDQAQLTEATQL
VQQNPKQLVSLVESLKARQLNLTELVVGNVAVSLPTIEKIETPLHMIAAT
IRHHDQEGDQEGEGRQDQHQGQHQEKKVEEAHI
> HOAE1_PSEU2
MMPSQITRSSHSSLPEVAPASGDAAGVSEQTPQQARTVAFFASGELAVAF
GRTSTAPAQDSVRLLSALQRELDKQQPSWPTVAQLCHSLAEVAKTEQG WH
QLASEDQAPAPALKDLLERCIGRLADMPASHASHDSLSLACEGLRTARLH
QSVARLTARPPALARAIPDLLALTHLDPETLGAEPPVVSSYTLFSHFVHT
AKQRTAELNDSLQRQPEAVVSLLRSHADTLNDLETLPGALQALTENCQDA
PACNELRELAEVVGALLQLLREHGILPRLEAISIEPGEAPAPGHEAAEPR
LTRSQALLKAGGNLVRKFDAYGALAPMDDKGLLALMRTPAPHLSPDQMH A
FLNKHVLHLTQEQRDIVSNTPLPFAPEGDIEARCGMGFDEKLRLALANGS
LVLSEEQLARLGHLPSAATTTSDVVKTLLEKPSSALSEAERDMLGAIVQA
NGQGQLDAWRAHNEQLPAVLSRSGLPSDVKDELLSLNQSMNAELGTLKNG
ASFKSRILASPAMLLALAPLPLAVAFVSKDNSYSSSLVAHFTKNAVFMAG
LMMNELTNARTNVDHGLNRYFVTVLANAIVAQPTFARNEHLLEQVGFGIA
TAVASGAATLGVFNRESIVAAFKLAKSKLSRQDTGNASIPEEDHLAVVRH
FDVSEHFAQQMKVATELYKQDKSITDIMNSSLTYLGTKSSEFQARFESVD
ALRAGLQLAEGERKADPDFYTKLGLVALTASIGAALVMLMKSMVGKADYA
ADGVWCVSEMLKLAMNPEVDMQKAVQVFKEIVGLNLVMTGFLGVNKVWNF
LDKGIKGYASGAAVLTAANLTLPGMVGEVAGAAAGKGLSYLTDKGKAAHQ
AGRAAASWAGNYVGTSRLGSAVGTLQTAIPGRIAGGQVVAGLYDRFRYLT
GSHPTPAQQAAGEP
> gi|29840709|ref|NP|829815.1| hypothetical protein CCA00954 [Chlamydophila caviae GPIC]
MSLSTSGPDNANQKNIMAQVLASTPQAVPTPDKLAGNETKQIQQTRQGKN
AEMQSDAGIAGTQGKEKTSAVSEAQGADNIMAGQGIAAGQETEAAEAAAG
ANQTAGAAAFRANLQASLEETNKTLETTLSSLSSLDSTQLHEVQELVASA
VSRSSNSAIRGLETPDLPKPSISKPRQEVMEISMSIAKAIAALGEATASA
LSDYQSTQAQASTMNRLSLESQGLKIDSEREEFQKMKEIQAKADGNKTLE
TVNTVMIAVSVTITVVSVVAALFTCGLGLIGTAAAGATAAAAAATAGATA
GVTAATSVATTVATQVTVQAVMQAVKTAIVQAVKTAVMEAVKSAIKQGVK
QAIKAAVKAAVKTLMKNMSKIFKTGQKALSKSFPKLSKVINTLGNKWVTA
GMGVVVAVPSLVKGIGDLKLSEMQTELADIQKKTGMLTAQAEMMNMFTMF
WQQASKIAAKQTDSANEMQQQATKLGAQIAKAFQSISSGLAAAV
> YPCD1.67c yopH putative secreted protein -tyrosine phosphatase 47122:48528 reverse MW:50870
MNLSLSDLHRQVSRLV QQESGDCTGKLRGNVAANKETTFQGLTIASGARE
SEKVFAQTVLSHVANVVLTQEDTAKLLQSTVKHNLNNYDLRSVGNGNSVL
VSLRSDQMTLQDAKVLLEAALRQESGARGHVSSHSHSALHAPGTPVREGL
RSHLDPRTPPLPPRERPHTSGHHGAGEARATAPSTVSPYGPEARAELSSR
LTTLRNTLAPATNDPRYLQACGGEKLNRFRDIQCCRQTAVRADLNANYIQ
VGNTRTIACQYPLQSQL ESHFRMLAENRTPVLAVLASSSEIANQRFGMPD
YFRQSGTYGSITVESKMTQQVGLGDGIMADMYTLTIREAGQKTISVPVVH
VGNWPDQTAVSSEVTKALASLVDQTAETKRNMYESKGSSAVGDDSKLRPV
IHCRAGVGRTAQLIGAMCMNDSRNSQLSVEDMVSQMRVQRNGIMVQKDEQ
LDVLIKLAEGQGRPLLNS
> gi|2865295|gb|AAC38388.1| rOrf10 [Escherichia co li]
MNEQFCKDLAHTFGITLEEQTDALAFHDNDGHEWILECAPQSEILFFYCY
LSTNEPDQINKILEINNNREMLGMFFLSLKDDKTFLNIAFPKNKIDITEF
TNLMENGYLLKKEIIKLLSL
> Q7DBA6_ECO57
MINPVTNTQGVSPINTKYAEHVVKNIYPEIKHDYFNESPNIYDKKYISGI
TRGVAELKQEEFVNEKARRFSYMKTMYSVCPEAFEPISRNEASTPEGSWL
TVISGKRPMGQF SVDSLYNPDLHALCELPDICCKIFPKENNDFLYIVVVY
RNDSPLGEQRANRFIELYNIKRDIMQELNYELPELKAVKSEMIIAREMGE
IFSYMPGEIDSYMKYINNKLSKIE
> gi|34498073|ref|NP|902288.1| cell invasion protein [Chromobacterium violaceum ATCC 12472]
MAQPKSLLKPEDAGVLHSWLAAKREDAGFTAGVEHTANKLEQIIDKELE Q
RGAEQDPGFDISGLSTRMAALLSQAIVLMSALRTADNALSTKLSLVSFDA

TKATAASMVREGIANLSSSIVQSVGQVAITGVGAKKSLNGLNAERGALKN
NAPKLGKLGDEGRNVQATLSRQNTVKLAADADGLKQVGLKPQNGAARPEV
AELSAQPNAAASVADGMAGDKISLQSSNSKLARQHEAALGSSTEDLTAKS
QAEQLAMDDTKLKAQAKQTTGKAIMDSSAAAGNIAGGSGRYAATLEQSEQ
QISQASSRVASTASEETRESSRKADSIIQELLRTLEGISQSKSSAMAAIA
GNIRV
> A0G5K3_9BURK
MKILIIADDLSGAADCAIGFASAGHRTVVTLEATCMPAHDGADTIAVDTD
TRRLTPQDAATRTAAAYAEMSARGQRLYKKIDSTLRGNWAAEVAGLQALA
GMAIVAPAFPATGRTVRDGRVFVHGEPLEQTATWKLEHAGQPAGIAAQLE
AVGLTTERLDAQALADEPETLAVCIGAMAA NGVQALIVDTHSERALRALA
RATLRSDAPFFWVGSGGLAREIAALDSRVDPAGRGPGNTTFPGGPILILV
GSLSAVSERQCAMLRERGGVKELIVPPAVLRAGATHRDWHVLQEQIGVSL
KADVDLLLRIGRDDAFDPAEGAQLSAALAALVEPHFTRTGGVIATGGETA
RAMLAAARIGNLQLLAEVEAGVAVARPLDATRTHCPGVVTKAGAFGTDHA
LYAAWLTLRNQTERDVVPH
> Q8XA51_ECO57
MTDGISTSPHCLYKSNIVDDVIINKTRQNELVKVFCEYKTEFLILFDDFF
RSQDLPKPSPVLHHFFQYTHLRDAHFYRCKLIEHTVQFSFFKHKGITLLR
LDVFDDRTSECLSEEIKIYQECHEKFIKFLKANFNQEIYPELYTPEIFYE
ACRNLQSFYDHQETSQNAKYSAIVKKKSYFNKEIRNLIKKNIYPELYNEQ
CNKIPASSTDDNQKITWQNFKTSNAAYSQLCEKLSLLKSSPSRLIEKS AY
CSNENMITDKFDVVFSYCGDNVKEFILLLPYNKSLEMHELNEQNIQYLTA
LNINIHKLLLSNITIEKSNLSYGYYFGCVLSNISCFESDLSNTIFSNGEI
NNLFIKKSNIFGTSFTNTMIKNLRCEDIMPGRWTTQLVNKHLGYRYTGVF
KTLASIDDKPSRFEILIPLVQTLVRDNVKLNNDVYKELKKFMHDYDKTSP
EMRKYLQSINESMFLMKKISHQD
> gi|28868097|ref|NP|790716.1| candidate type III effector HolPtoQ [Pseudomonas syringae pv. tomato str. DC3000]
MHRPITAGHTTSRLILDQSKQISRTPSESSAQSALSQQASMSSPVLERSK
SAPALLTAAQRTMLAQVGACNAHLTSDENMAINELRSHKPLLPKDTWFFT
DPNKDPDDVVTYTLGKQLQAEGFVHITDVVATLGDAEVRSQRAEMAKGVF
NKLELHDVHVSRGRD YAMNSLQSKEHAKFLLEGHALRAGPGEIHRDSLQD
MSRRLARAPHGVGIVVIAGMSDINALITTCPDMVRERVDDITIMGGVEPL
KDADGFVQPDARAYNNATDMDAARSLYRKAQELGIPLRIVTKEAAYKTAV
SPSFYEGIAGSGHPVGHYLRDVQKSALKGLWEGIQAGLLPGLDDSWFFRT
FMPNAQIEAAQLDKNKESSFEDIWPKVTKLNLYDPLTLLASVPGAAKLLF
KPKAIHTEGFGVVEQV GPDDVTHPEKAKLLMSALAKSALVQSTVAPD
> gi|5070694|gb|AAD39255.1|AF156163_1 avirulence protein AvrBsT [Xanthomonas campestris pv. vesicatoria]
MKNFMRSLGFGSSRSSRSSSSNWNEQQADNDEQTPASSPSTSPSQTSSAF
SGLPERPRKKAIALEESLNSSNNIPYEMRMYAEAALSAANDGSSEAITKA
DVENKYYLAHAYNERFP ELHLSCHDSAQSFFSEFMTSEKQAWRSIVRLSP
SSMHHAAIDVRFKDGKRTMLVIEPALAYGMKDGEIKVMAGYETLGKNVQN
CLGENGDMAVIQLGAQKSLFDCVIFSLNMALCAYQKDSVFDNLHDCLRRN
VRCFSSGERKSILHKNIEFIEGDKFLPPIFYKHSHSRGVVGEFISNQPEY
AHKNVSTGRTNPSEDLSERVENFRVRRGDLSYSMSIEASRLRKIRKTIES

> gi|16520028|ref|NP|444148.1| Y4xL [Rhizobium sp. NGR234]
MDINSTSPLNASPQPDSPPPANASAFAHQLSGFQYSPPHAADSLLPQVEA
DSPYLDTRHPYSQYLDSAYPYPSPCEWQHDLYTRTRERSPHPSEQRPHAR
VLQGAPEHDQDQHLEAAGPREGSWQVGPSRSGPSQAGLSPSATPLNPSPP
PHATDLETKHPYSQYLDWANPSLLDWQQDLHTRATASPAPLTAERGRSPQ
PSEQQPHARA LQVPEYDQDLIWQRVDAAGPQAGPWQVGPSHSGPSQARPS
HAWPSSSAGAEPAELSDFVMDSGVRAWDHWFLAPHMASEDQMSMLRATGL
MPTAEVPTTTFLMMGMRHVAEFRGEGVIRIRPSVDFDI
> Q3CIL9_THEET
MYRVIVIADDFTGSNDTGVQLVKHGYYVITLIGTAGVKKYEDVADALVID
TETRSIPALVAYEKLKEISNIVGEYKEAVVYKKIDSTLRGNIGVEIKALR
EGLKPELTIFAPAFPKNGRTTEKGVHYLKGVPVDKTELARDPKNPVTTAD
VKKILEEGLKVPVKHKVLEEISRDLRKDIEKEMREYDVFSFDAKTDDDLI
KIATSVLDLGKKTLWVGSAGLAEALIASLEKKEKKNPVLVIAGSVSQVTR
RQVFKALEDSRIVLVKLDVKRALSHPEEIDRIRKIVLDFMNAAKDVIIA

SAAEEDAVSEALIAARGKGLSPQDTSEVLAQFLGEVACSIVEAKRPGGLV
LTGGDTAIHVVKSLSTAGCRINSELEPGIPELVLIKGSVDGLRVVTKAGA
FGNDESILNAVKFLRGDGR
> hopT1-1 Pto DC3000|Genbank: AF458399
MMKTVSNHSIPSTNLVVDAGTETSAQKSQPVCSEIQRNSKIEKAVIEHIA
DHPAAKMTISALVDTLTDVFVRAHGEVKGWAEIVQAVSRPHDSNRHGSGV
LSPRFDVMGSVGWNAAAIRATSRVGTLREKGTLFTNLMLSNNFKHLLKRV
VNDPALQQKLDGGLDLNYLKACEGDLYVMSGWAARASESREQIGKARYET
ASNLSQTLISARELAFHRHNPVNHPSAQTKVGFDKGLPEESDLQVLRGHG
SSVWSVKPGSDFAKRAEVSGKPIIAGPSGTASRMVAVARFLAPACLKSLG
IESEQNLKELVRYACYAYFGQDSHHSMLEVNLGVASHGMPEQWDDTLYNE
PFSNSIKGRGFGIDNLAHRQVVRQAAQKS
> NleD_Crod
MRPTSLNLTLPSLPLPSSSNSISATDIQSLVKMSGVRWVKNNQQLCFHGT
DLKIYQHLEAALDKIESTDTGRTLLNCIELTSRLKSEKLAIHLDSAELGV
IAHCNADAENSRGTGSDFHCNLNAVEYPCGQGISLVDFHACIVFHELLHV
FHNLNGERLKVESSQPELQTHSPLLLEEARTVGLGAFSEEVLSENKFREE
IGMPRRTFYPHDSSLIHDDNTVTQRFQRKKLHPLL
> gi|58579791|ref|YP|198807 .1| avirulence protein [Xanthomonas oryzae pv. oryzae KACC10331]
MRSFMRIGPPQTSIAHTDALAIPTHTSASSPTQVPHVQGNTPPLRERAPR
RAVSIPPLVPLNDSAMTGKPALVALDSEFSEQRLAEVQARQITVQTLQGK
LAAHLAQAGTALTPDSIAARFAAGALEPVYLDTAAFNAMSRGLPARARAA
SGPVLIDAQQCRIVFNLQRAFASGDTFSDAALTALGKA LDLPGHGLATPD
WLQPAARTPTRRKLQHAPRYHGHEVPARDGGAAFFKANDHRLLEGKQALL
RNHRKALVHDHYFEAPSTRALGKDVMVHRGLFDNHAGIPENSLSSIDNAY
AKGYRNLELDVEVSADGVPVLMHDFSVGRMAGDPQNRLVSQVPFAELREM
PLVIRNPSDGNYVKTDQTIPGVEQMLEHVIKKPEPMSVALDCKENTGEAV
AMLLMRRPDLRNAAAIKVYAKYYTGGFDQFLSNLYKHYQ INPLHSQDAPR
RAALDRLLAKINVVPVLSQAMLNDEHLRGFFRSNDDGAEGLADTAMQWLE
SWTRMRPVIVEAVATDDSDAGKAMAMARERMRQPDSAYAKAAYSVSYRFE
DFSVPRANHDRDYYVYRNFGELQKLTNEEFGVKRTTAGAFRDDGESLLTD
QAEAELLAILENRTLARGHTGNELDVPPETPIDINRDAEIVKQRTREFQA
GSIPADPNHIAAVREGKQRDHRADMVHDPAATRAVDKRAQ ASGLLTEKYR
GAPVTHYLNERANQTEPE
> Q7ABH4_ECO57
MERRAVALERQLNGGVDFLRSVNNYFQSVMAEHRENKTSNKILMEKINSC
VFGTDSNHFSCPESFLTCPITLDTPANGVFMRNSQGAEICSLYDKDTLVQ
LVETGGAHPLSREPITESMIMRKDECHFDSKKESFVASDA
> Q8XWW1_RALSO
MRPTAPRIGPPSATVSGEPAADAVPQHAEPGRTGRPPRARGLPLEQRRTG
PAARNPRGAVTQGDAGASDALHGQALSAEAPRRSNVAGPPADRAESVPSR
VRGGTQPQASKELRARRQLPAEVVRQLSETVHMQVLLANGFAWDSSLADD
LDDVRLDSARQLSAIAHRDVMPPESANLVFAKLVKGRALILNSAQDQGGC
RSPALAGLLFLGEHHNRWKAHNGMPSHTLPALIYGPGQDSDGFAAAISGS
GGFGTSHLHVRGKAVNNASVIAHESVHEAQYNKRLGLFNALMLRAPTTHV
DTAAHARAGFLEAARTLPVLPPTDVSAETAKICYVSDPTEAQAYLEEAQL
RSLLPSDQGMGAYVPRQDARLHELADLLAEVADALNVAGPQEQAARGILA
EFDPARYPEAGEALERILLSAASLHEMAESTAWDAGAADAWKGTLEILRQ
RCDAIRTEPPLGSGLLGLADALERFLVDTAKQRSAQALALIADTLEVFLE
RVAQTHGHAVHALKARPVTPEAFDAAKQDPIGVLGQLLERCGFAHAQGLP
AAISAARSASGFSSAVADLKTRLGKEEPVAADDLREVVVLLASRLLGLAP
SQLPAALVPAVNLYASPVVVLPNVAGLNGVSHQKRKAAFDVATWQLHIPR
NVFDAPERLESRLAGLAADVAYAAVSLREDKQFETNHPLFALWYGPLSKR
FTNALAAEEVAASSRDFLARHPDSAAAALHAIVQAVRVDDGIEARAWHLG
PRAEVKRASHAGTELPDRAWPGEDEARPDADAARHAYQSRTFERWQVYSV
AFRRAIAHL
> Q8XA11_ECO57
MKVSVPGMPATLLNMSNNDIYKMVSGDKMDMKMNIFQRLWETLRHLFWSD
KQTEAYKLLFNFVNKKAGNINASKYFTGAVNENEKEKFIHSLELFNELKT
CAKNPDEMVAKGNMSWVAQTFGDIELSVTFFIENKEICTQTLQLHKGPGN
LGVDLREAYLPGVDMRDCYLGLKTMKGHNKVLYLEPGWNANLDGATLDGA
TLDGATVDGATHLYDEVIIINKITPKKIDTEE VATKQSTAEQITDNAIIE

> gi|2865297|gb|AAC38390.1| Tir [Escherichia coli]
MPIGNLGNNVNGNHLIPPAPPLPSQTDGAARGGTGHLISSTGALGSRSLF
SPLRNSMADSVDSRDIPGLPTNPSRLAAATSETCLLGGFEVLHDKGPLDI
LNTQIGPSAFRVEVQADGTHAAIGEKNGLEVSVTLSPQEWSSLQSIDTEG
KNRFVFTGGRGGSGHPMVTVASDIAEARTKIL AKLDPDNHGGRQPKDVDT
RSVGVGSASGIDDGVVSETHTSTTNSSVRSDPKFWVSVGAIAAGLAGLAA
TGIAQALALTPEPDDPTTTDPDQAANAAESATKDQLTQEAFKNPENQKVN
IDANGNAIPSGELKDDIVEQIAQQAKEAGEVARQQAVESNAQAQQRYEDQ
HARRQEELQLSSGIGYGLSSALIVAGGIGAGVTTALHRRNQPAEQTTTTT
THTVVQQQTGGNTPAQGGTDATRAEDASLNRRD SQGSVASTHWSDSSSEV
VNPYAEVGGARNSLSAHQPEEHIYDEVAADPGYSVIQNFSGSGPVTGRLI
GTPGQGIQSTYALLANSGGLRLGMGGLTSGGESAVSSVNAAPTPGPVRFV

> gi|53722438|ref|YP|111423.1| hypothetical protein BPSS1411 [Burkholderia pseudomallei K96243]
MRANRNNGLTAGALPPNSAAPDDAAKAAPPVAQSDRGAG PLAGLTALKAD
RAQCMSARPSIRSTTKAPAVMSHARADTSRELTPVEARIAQTFVRPEHLR
AIAAAARAGSFAVSFRASGQLSLKRLANGAPAKGHDILEKTIKPGSVQEV
YGADSERRLAQIERADIDGYVGHWGRDKLLGLYMGPAANERDTPMLFAAL
RSTPSGHRYYPVDHDDLEGSLALLKRTQGPSGTAGSFWMSLPYTGDYDTH
DMINMAGHRTPVPSGSRDEHRIMRSLNVAIEAIDPIRKAS SGSHRLIQHG
PQHNYVAHMRTNEQGAVIDEAVARASFPLAMCDRGTWSVIETPEALAAFY
RQHGVVLKEKWREKRANTFTA
> gi|21241060|ref|NP|640642.1| avirulence protein [Xanthomonas axonopodis pv. citri str. 306]
MGLCVSRPATSGSSVAASPEQHPNPASEQVTPHTPSVPETSVPPSLQGLP
TRCVPRARRGRSGKPTLQPHEVQQAAY QLGMRLSGRPIEDVRDRQRLADA
TATVHETRLALHHGRGNIESDFRLSDGRSSTCSYLSYSFLSYWPGKHDID
LMAGCALAVSAGNCDQNAAVNTRRHAVRMEGGGQITNVCDEACAHMYALY
QPPGSAEADDSAVVLDSWSDGPAVLLRDSRWAGTYRRTSTYPIERFDKPG
AIDALARTRALQAEIEDPQTEFHAHLRGVESVVRTPPVPGKIYASTPVIA
PDLVQRTRQRLQELSPRTRKALAADAAR QAYGLDDAQPISPRTTTAILKD
AARLDALGRPPLSWAPTSHTRLKRFVTSARTNARQAFWYRSGWRPPVTVA
R
> gi|34495751|ref|NP|899966.1| hypothetical protein CV0296 [Chromobacterium violaceum ATCC 12472]
MKIGVLNAGLRQFAAEFGERAARAGRGLAGMFRRAANVAEAAPRRSAAAI
EQGWQRFSARFLGARGADKPAPTTLTRGGAA RSPLSPGDVKAMIRSRLDS
LRDGMADPVYRRQALESTYATIYSEAKQAFYRANGGKMPEGYLRELGEAA
RAAGLPGENKHGVFIPSGDGASPFVNYLLIPVQKEFGHRLRNESQQRLFR
DFARQLSMELVAPHAAERGWEPPAAFAARLEAVGRPWLAQA
> gi|16765569|ref|NP|461184.1| leucine -rich repeat protein [Salmonella typhimurium LT2]
MPFHIGSGCLPATISNRRIYRIAWSDTPPEMSSWEKMKEFFCSTHQTEAL
ECIWTICHPPAGTTREDVINRFELLRTLAYAGWEESIHSGQHGENYFCIL
DEDSQEILSVTLDDAGNYTVNCQGYSETHRLTLDTAQGEEGTGHAEGASG
TFRTSFLPATTAPQTPAEYDAVWSAWRRAAPAEESRGRAAVVQKMRACLN
NGNAVLNVGESGLTTLPDCLPAHITTLVIPDNNLTSLPALPPELRTLEVS
GNQLTSLPVLPPGLLELSIFSNPLTHLPALPSGLCKLWIFGNQLTSLPVL
PPGLQELSVSDNQLASLPALPSELCKLWAYNNQLTSLPMLPSGLQELSVS
DNQLASLPTLPSELYKLWAYNNRLTSLPALPSGLKELIVSGNRLTSLPVL
PSELKELMVSGNRLTSLPMLPSGLLSLSVYRNQLTRLPESLIHLSSETTV
NLEGNPLSERTLQALREITSAPGYSGPIIRFDMAGASAPRETRALHLAAA
DWLVPAREGEPAPADRWHMFGQEDNADAFSLFLDRLSETENFIKDAGFKA
QISSWLAQLAEDEALRANTFAMATEATSSCEDRVTFFLHQMKNVQLVHNA
EKGQYDNDLAALVATGREMFRLGKLEQIAREKVRTLALVDEIEVWLAYQN
KLKKSLGLTSVTSEMRFFDVSGVTVTDLQDAELQVKAAEKSEFREWILQW
GPLHRVLERKAPERVNALREKQISDYEETYRMLSDTELRPSGLVGNTDAE
RTIGARAMESAKKTFLDGLRPLVEEMLGSYLNVQWRRN
> SIPB_SALDU
MVNDASSISRSGYTQNPRLAEAAFEGVRKNTDFLKAADKAFKDVVATKAG
DLKAGTKSGESAINTVGLKPPTDAAREKLSSEGQLTLLLGKLMTLLGDVS

LSQLESRLAVWQAMIESQKEMGIQVSKEFQTALGEAQEATDLYEASIKKT
DTAKSVYDAATKKLTQAQNKLQSLDPADPGYAQAEAAVEQAGKEATEAKE
ALDKATDATVKAGTDAKAKAEKADNILTKFQGTANAASQNQVSQGEQDNL
SNVARLTMLMAMFIEIVGKNTEESLQNDLALFNALQEGRQAEMEKKSAEF
QEETRKAEETNRIMGCIGKVLGALLTIVSVVAAVFTGGASLALAAVGLAV
MVADEIVKAATGVSFIQQALNPIMEHVLKPLMELIGKAITKALEGLGVDK
KTAEMAGSIVGAIVAAIAMVAVIVVVAVVGKGAAAKLGNALSKMMGETIK
KLVPNVLKQLAQNGSKLFTQGMQRITSGLGNVGSKMGLQTNALSKELVGN
TLNKVALGMEVTNTAAQSAGGVAEGVFIKNASEALADFMLARFAMDQIQQ
WLKQSVEIFGENQKVTAELQKAMSSAVQQNADASRFILRQSRA
> gi|53722415|ref|YP|111400.1| putative membrane protein [Burkholderia pseudomallei K96243]
MAISPTIATNRQVVGSVGSENVPGPA AEALQSVESQLHQLMARQLQGGVA
MAQRQGSGIRAAQYAPLDDYTRMYDQADRDRWADLAALGQGVSLERPLAA
MQILSGERQPSAAQTAAALQFVNDNPSLKTAMQNTGGLKPDGSVDPGKVA
DLLKEVEANLAKADEDVKAYLNDHPDADRDALDTVRAAALLEAYEPIVGQ
SAGHQSSGKNHSYSTGGKNNGGGLTTKQQVADLQNNGGFSAALKSAAKAW
STSGAFDNLDRAGDDKATEKVDQIFSA NNLHHFITKDAPTSDAAEHAFLE
KASLENITAGTDISKLNQDIFANPQDYSPQQKAAVMVKLMETLVDVQAGG
ADKLRDVTATVSALKQDIMMLANDPETNAYLHQMVPPEMRDLNATFEQAG
GLEGDTGAAGDMSVADNANRVSARNIVDQTKDALDEAEKVAKRLGKLGNE
GAGEATDATQSIADGAETAADGAVQGGVEEVGGAAASAVDGAEGAITGVA
DGVEAADGAVTGVAEGVEGALAGTEGAV VGAEAGAGVAEGVMGAVAGGMA
AAAPVLAVGAAIAGIAGIVLAIVEAVKKAQNRKAFAENVNPTLNQFGIPL
PT
> gi|51591604|emb|CAF25408.1| yopN, lcrE; putative membrane -bound Yop targeting protein [Yersinia pseudotuberculosis IP 32953]
MTTLHNLSYGNTPLHNERPEIASSQIVNQTLGQFRGESVQIVSGTLQSIA
DMAEEVTFVFSERKELSLDKRKLSDSQARVSDVEEQVNQYLSKVPELEQK
QNVSELLSLLSNSPNISLSQLKAYLEGKSEEPSEQFKMLCGLRDALKGRP
ELAHLSHLVEQALVSMAEEQGETIVLGARITPEAYRESQSGVNPLQPLRD
TYRDAVMGYQGIYAIWSDLQKRFPNGDIDSVILFLQKALSADLQSQQSGS
GREKLGIVINDLQKLKEFGSVSDQVKGFWQFFSEGKTNGVRPF
> hopR1 Pph race 6-1448A|Genbank: AAZ37024
MVKVTSSGFTANPLPHHADSVSPANSPPQLPEPLPLVDESPSSHKGGMRN
RAHASLNSQVLGLQAVPLQRGRHVRIRSHTDGVSVINDWLAKRPSVQSET
SLDNNGKLVRYTAMHHEPLAPRNEAFFTSVPGMLMAVLTVHPDIEYGISG
EITADAVAARLAEPPIGLLTGVWQSSHDRAYIERGGVVHTANMEERWTSL
TLPGADPREPLRMIGL QADGDVYLHNGRQLWRATDTSAESVPTERLPEGA
AVRIGAGREVQWLHEGAVHSNGISRPVELLRPEASGLGVEQSPARPVDLL
PLPRGNAALILDDKGRIYQADLKGIGPVEAHRLKLPGDFAQGKGWAVTTM
GLSRDNTVHLMLQDQNGRRMSLQRAQNEVLFRPAYLLDRPLLLLYTEGLH
VPSETAVQSHVQLDGHAQVGHIDGVLHYQPAPDQPWERLRQPGGEPLTGV
TALYSSALGFIDRKPVF ALLGDARQVVELKLEGRTSWLPTDAELPRHPAG
GPLAVIPDTVELRARLIAQFDEPVQALAVHNNRQSVALMESGLLIAADAD
GKVRRLPLLQRPIALAIGLNDQLLVLHHPRNQRPQLKRLSAKDDWEPVPI
ILPGIDHPSNLRATRTGQIQLQLGDNWHTLLPAMTSHDNRPLPARVKPEP
EADELPSENFLAGTNARVNQQQASRISTPHHDASAVTTLMGTAANNPLTM
TSSVQAVVDTTRAQVGAL ARDVLGGVTKNTMRAMAHKLGVVLPPTTQERR
LASFHYEAKQAYTSGKTLFEQLPTLAQVRVASAVGPSDGEKFGLSHQQTQ
RLLNLREEKLEALLRDLRKIGFHEGVIMGDMGDGESAGDLTSTTSTPTFR
LAELWRRQHSQVNKALSSVGLSRSEDILPNLNQSIKALAGGAALHADRMG
EREAELLSVLCEVSEKIMRAGVRLPADDGSADSASSHAPHGLRTSGLMAG
LVDYDALLTSTDTQALEMA ERLQQDARLPGLCKLGLSSWVQLAAFDDVVT
TFREQISLPGSARRTQLLKNLGLPPDAAPDEMAARMSDLFLDLFNRSTFF
STQSRGLEIRGSLGGADWKHLNAFSVGITGEALQVLGVERIGDGKDGDAG
LVAFFVRHAKASVSATSGVGIDFKPGLGTGGRVLDSRPGRSMNSTWGGSA
NLGISGTYQHGQGAAVIIAPSTISDFVRLLFDVNHPDSTQILRTGVNGGS
IGLDLFETNLNGSVGANVSV SPFSLSQKYGPQKPSADAAASGADNRRSTA
SGALSVGATAQAGAHWGQMELHLDHAWAEIIGLEFQGRTDFNLEFNSNLN
LGGALSSALGDRPQKLINASTGNGNLQLAGIRVASSDVQLPTDSVVDDKR
RGPFLSTASYKRTFDTQVAKPITADEWSQMRQRLASAFPDNIGELGALAY
STSPSERIATINQMIDRIQSSKARNVEAGGALDGNALRRQRLDAAREMSN

AGNSVWQASSEIDRASVVEML HQLRQQEQSAVQHHARAIPGARVEFNLFG
RESLETVVFHAIGHIGLGSKLNDMAELRRKTPGLDQVMRCFQSLPNINQV
RYVFEMRPQARFAINDALLAREQQASARALGLPGSSASELNWRGVLDKIK
NTPDLYRLAAIAVHNTDENPVTSRIGLPLLNVSATGATSHQLFEAEIQFR
YGLYDGLQGIEVLEAGSRALQSPLRVLQQSGIQALGQRTQAGDVPYGPPS
PRKEATMRTAGEAAALTTNDVW RQLDGKIQRMNSAHEREANAIGSFQHAY
GTASAHVDRLLLRIPELPAPEIDDRNADGESVRGAFASLKRNHQALDEDV
KAMRQASEMVYSIPGERTRQTETSALAHVLSVEKSRRSLGHAMEILAGKG
VEAGTATGLELNRVSSQVNALVVRRDELLTQLERGAQEGGSNSEEMAMEL
QQTTSLLQRLRADLLGERQAMDATAKRLDQANRVALEGERSFSDAVRDMT
WGELDKMPQ
> ECs4561 ECs4561 Tir 4599930:4601606 reverse MW:58023
MPIGNLGHNPNVNNSIPPAPPLPSQTDGAGGRGQLINSTGPLGSRALFTP
VRNSMADSGDNRASDVPGLPVNPMRLAASEITLNDGFEVLHDHGPLDTLN
RQIGSSVFRVETQEDGKHIAVGQRNGVETSVVLSDQEYARLQSIDPEGKD
KFVFTGGRGGAGHAMVTVASDITEARQRILELLEPKGTGESKGAGESKGV
GELRESNSGA ENTTETQTSTSTSSLRSDPKLWLALGTVATGLIGLAATGI
VQALALTPEPDSPTTTDPDAAASATETATRDQLTKEAFQNPDNQKVNIDE
LGNAIPSGVLKDDVVANIEEQAKAAGEEAKQQAIENNAQAQKKYDEQQAK
RQEELKVSSGAGYGLSGALILGGGIGVAVTAALHRKNQPVEQTTTTTTTT
TTTSARTVENKPANNTPAQGNVDTPGSEDTMESRRSSMASTSSTFFDTSS
IGTVQNPYADV KTSLHDSQVPTSNSNTSVQNMGNTDSVVYSTIQHPPRDT
TDNGARLLGNPSAGIQSTYARLALSGGLRHDMGGLTGGSNSAVNTSNNPP
APGSHRFV
> Q8X5G5_ECO57
MKITNYILPTSRTHGSFSTIKSWDTMNYIKHLIRHTNDPIFEEQFYKITQ
SHIDFDKRAKDEKNDTINIYDNFFYSSNDDLDSKIRSMLNNLYEKSLTFR
RIINYYVKEINLSDYGFLKCKILPAYAYNYEMENDAPPK ILIPIDHNLNF
IDAKYNGETYRGNEEFAINLFLQHILHNDIQEQTSIDLYTSIINKELDSN
RKSYNNEIFNNFSFDKSVKLNSYNYIADDIEQVIDKGSKVQLEVYNLLSE
EKIFEHKIMNNWTRSIKNILTTYLFMSSGAVTARNVQTFSPTINNESRIR
LPRALPVGHPYPEEHKASGFSPFMMGGLSGDILPEILTGNGPSIFFNGKH
NNQHDGAFGKIIDFTQNGNKISAKDKEIIKRYIFDKINVL IKEYFIRTGK
NSHTPFEVFIKERLFNQYDIFKTLARDILAHPLVIYDAGYKNYHESLNAA
IAINSRPLQEIHYGDVLYHYHKNDISLGVDTLYGRESFDIVLDAMNVYRK
SKKMRVISNNEMKKSIKISELVIHNIIKKGLTNCLLKKDVLNARYDLIRD
ILRYSLNIRQGIKHDDVNRIAENIIKKYGITEGMNPKPRNARISKELLLL
AVDRQIEWAKKHFITKDVLENVVSKCDLSSIFNVNKVLQNT ILEFVHEIN
NISSARWMSKSEKNNKQKEAIEKFKKEVSHMNGGQQFIWGFDKVIQEGLS
GLIELSIDINDSTNHRDKSSLSPDGRAVLHFLGTIWNMAMGAVPGYNALS
GVSSILHSAIVKESSNICDYIQGAVRIGMDFVPGTRSDLHSRSLQIKYEA
LKHIEKNINDNIIYHPSNNANFYSVIESIDGNDFIYNEKQSKILEMKQDR
GGNRYSAVDLNSSKYGYYEKVGGGFYRYIESFNPISSETPNK IVYKGESV
DLTKEPNSELYSGRYSINNKQVNVYFFRDADGTFYKSEGLHGGGVIRYID
KPYSQLREGDIGYDEDLLDIYDDSPVLEDTLPALSSEIVPTPEHSIKQIY
SKIKEGHIELSDSDIILCRGTTGIQAENIVEYKTAGGLPDSNPNVKAPDE
YMAQQQVRIGRILPEYTSDLSVADRFSREHYLIVVKVKAKYITRGSVTES
GWVIDKTAPVEPLAIIDRTFGMKENISMVNASK
> A1JU68_YERE8
MFITPRNVSNTFLQEPLRHSSDLTEMPVEAENVKSKTEYYNAWAVWERNA
PPGNGEQREMAVSRLRDCLDRQAHELELNNLGLSSLPELPPHLESLVASC
NSLTELPELPQSLKSLQVDNNNLKALSDLPPSLEFLAAGNNQLEELPELQ
NSSFLKIIDVDNNSLKKLPDLPPSLEFLAAGNNQLEELSELQNLPFLTEI
HADNNSLKTLPDLPPSLKTLNVRENYLTDLPELPQSLTFLDVSDNI FSGL
SELPPNLYYLDASSNGIRSLCDLPPSLVELDVRDNQLIELPALPPHLERL
IASLNHLAEVPELPQNLKQLHVEHNALREFPDIPESVEDLRMDSERVTDT
YEFAHETTDKLEDDVFE
> gi|21243817|ref|NP|643399.1| leucin rich protein [Xanthomonas axonopodis pv. citri str. 306]
MPPTVGASPLRRSPAIRPYRDVLSQWQRHYSADRNR WHSAWRQANSNNPE
VERRTSRALKATTDLLEDATAPERVALELRSVPLPQFPDQAFRLSHLQHM
TIDAAGLMELPDTMQQFAGLETLTLARNPLRALPASIASLSRLRELSIRA
CPELTELPEHLASTDASGEHQGLVNLQSLRLERTGITSLPASIANLQNLK

SLKIRNSPLSALGPAIHQLPKLEELDLRGCTALRSYPPIFGGSAPLKRLI
LKDCSNLLTLPLDIHRLTQLEKLDLRGCVNLSRLPSL IARLPANCIILVP
PHLQAQLDQHRQVALELNKVALERIEDTAQAMLSTVIDEERNPFLEGAPS
YLPEKRPPGTPTTFGEIPVLQKMLEESRDPHFLQRVSDMAGPSPRIEDES
EEGLRLHYTNVSNWKAQQSAHLGIVDHLGQFVYHEASQLDAASLAKAVQM
WKTRELIVNAHPQDRTSFPDFTLHIPEQVSDDADDEQQTSPAPSRDQ
> Q8XXL2_RALSO
MRRTATSRPAAQGGDPAPSPPRFVVS TPMQIRNVPRSDLTPIRSGDNVST
PAPNSVPPRRARSASDLQAMGLCARGTPSSAPLALPAIGIATHHEAVQET
HLSAAAALETELLLPHATDAGLAPRIAFRLMNVATRIAEHCAVKPSPGEP
QAPIPEAWRCLEALASLRLRLHPAQLGLARQMLLGAVEASTHAPLDKALL
QDILKAVTQADGPGLDEYVARRAYDGLCLGQLQLKADDEAEPPTRTFNSI
LPSLAASTYDYNPLGRTLLWEGACRLF DGPSAQRRRAALQTAVFSLPNVP
APGKQPLDNLIEFVLAIDDMKLYETTPASEPRAA
> gi|28871821|ref|NP|794440.1| type III effector HopPtoI [Pseudomonas syringae pv. tomato str. DC3000]
MLIGHSLHHMRPTAVDSSLPTSATSQTISNTKSRLDPHRVRELTFIGVGS
SVAYLLNELNGRFADSGVTTPFLGKVSIVGKDDSWAENVRGKG YINHQTE
IISQWDQQVPKYDPNYAARAEFSASNRRQLTRTVELGAEHLKAQVTGISR
LDDGCFRINLDNGQILQSRQIVLGTGAGPHTSIWNSVTSHTQAEKRLDNI
KLHEQKALRGKVLDLDEFMRASDASPQTFAGKTVVIHGPNAGIDAAERAG
ELGANAVWFTRSTNPVLLDGNQLKFAPELAKSAIHKVDKLDIRPTKLENG
FALRLHYSSLGQDSREPKKVLDADYYVYAMGQDIHKPGSAAAIL GSLLDH
LEPIYDYDQVYSDQPFKTVIGLQSRGSNSDNGLIIVGAAVAQLATNVQHS
YKDHALDRILEEMTRLPEKQTEKLSQMLLEGAPSVQIQTYLKTWQLDSGQ
PPDKQVLQNQVENYLAARDYFQRQTNEQKGNLDGVAAEVKNQTLTEVASV
IVSPQLGTIKASAAALSGLMPAYVANGENNFTTDNRTMLRAGIAARYPNI
GNAEASAFIDEVVTLRHLNSQRFIEKVAGEMMDKGAQPLVSLRPP VLGVP
ASVRTAYEAYLHALNSGAHDGTPLSQRWLPKK
> GogB_Stym
MKIGFQPAILQYAYTSNEATSNLELLNKWRIESPDIEKEERNSIYDKIIE
ANHTGSLSITAHHVTSIPVFPDNLSELNLSSCYTLESIPNLPDGLKSLTI
SGNQTIKISYFPDSLESLSIDMQAYEENYTFPALPYGLKSFTACYGKFLP
PLPPHLSSLSLQNFSEILCAELPYKLDKLDLQNCPFLPLMKMLPEELKEL
SIELIRTVPGTVIDDILPDKLKKLSINFCDNIKLPVKLPVNLKSINLSSR
TPIAWEIPTCNLPAHIDISTDGYVKLNPEFLTRSDITFSNKPAGDVLSFQ
PGDVVYGLCKARDRVNTLVNSLYYFSKKDIIIQNTLTDAVWDRKNRAVFN
KDEKIAERLNDVQRGIFFREFLSQHKKYNITEDKYSDLSNEECWIKTSKA
GLEFQTRLRERSVIFVIDNLVDAISDIANKTGKHGNSITAHELRWVYRNR
HDDLVKQNVKFFLNGEAISHEDVFSLVGWDKYKPKNRNR
> hopZ1 Psy A2|Genbank: AAR02168
MGNVCVGGSRMSHQVYSPDRADTPPRSERNTPDRRQRAAGDAERTQSMRL
QQKINDLKPYVRHARGPIKAYGQAALDRASGKKTSVSFAELDATHLDAMV
YIENQRNPGLNLKHFRDHKELIQALQSDGPSAFRAIFPQTCPETGQTLKH
HVMADVRLHQGGAPTIIITEPAVIVGARYQQL QRHNLTLEDLSESGVPLS
QVAIIETQAQKTSDDCVMYSLNYAIKAHKNAAQFDDIHHGLQHGTLSTES
ESRARTTLGALEASSSYSVMHEGAHAAFGADVLPVDFYKHGASLTQAKQL
MKRPDGRMAGRVNSEGHSEAENLVQRNQAFRVKRRELLDDETPSNTQFSA
SIDGFRLQEIKRVLAEEQR
> Q8X2S5_ECO57
MRLSALYLMKPMGVFYPAAVVGDCLLSYSFMGLITALKLFAIGCSVCFR T
QVKHNRKRVFRTKYLNVSFLLVQEISSARQSLFSVQLH
> P0A0W1|YAV4_XANCV Hypothetical protein avrBs1.1 - Xanthomonas campestris pv. vesicatoria.
MEREMAHDERLHVHCGMGLGRTTIFIVMHDILRNAAMLSFDDIIERQRKF
NPGRSLDNNKDVSDKGRSEFRNERSEFLPLFYEYAKQNPKGQPLLWSEWL
DHNA
> gi|53722548|re f|YP|111533.1| acyl carrier protein [Burkholderia pseudomallei K96243]
MTAGPHLSDAAALAAAKTLLAGMLGVPEAQIAPPQRLLDDLAMDSLELIE
LAMELDERWNIRLDRARLAEVATVADVAALLGAAARADDSGGA
> hopAI1 Pto DC3000|Genbank: AAO54440
MLALKLNTSIAQAPLKKNAEAELRHMNHAEVRAHTPTRFTLNHRAPTYEV

AQSALGENHGGWTAVNKFKVTESEVFIHMERSDSRSKGDFAGDKIHLSVA
PQHVASAFNAIGKILQADDSPVDKWKVTDMSCASSDLQPEKKRVTQGAQF
TLYAKPDRADNTYSPEYMGKMRGMISSIERELHTAGVQQSNNRPASDVAP
GHWAYASYRNEHRSERAGSSSQANELEKEPFFQLVSFPDVAASPVKSGAS
SRSLMPPPWTR
> gi|16764755|ref|NP|460370.1| secreted e ffector protein [Salmonella typhimurium LT2]
MKPVSPNAQVGGQRPVNAPEESPPCPSLPHPETNMESGRIGPQQGKERVL
AGLAKRVIECFPKEIFSWQTVILGGQILCCSAGIALTVLSGGGAPLVALA
GIGLAIAIADVACLIYHHKHHLPMAHDSIGNAVFYIANCFANQRKSMAIA
KAVSLGGRLALTATVMTHSYWSGSLGLQPHLLERLNDITYGLMSFTRFGM
DGMAMTGMQVSSPLYRLLAQVTPEQRAPE
> Q8XB62_ECO57
MSIIKNCLSLINNALNIQKTSYSLTKMEQAGKLLNRKITPENTPPMLLSY
RNADLTQEKNITERVLSIFKIKRDFVAVRIQNNQFTDLKNKKIQGHQNTV
ASVMDWYNPQKNALGITMGTPRKSADIAKEEHRNALNFMIMEKNTFHEKI
LNSNDNLQKSYSKTEDSSWVAASVGSLLDKGAKVYPDTSCSLRLGEPFIF
TLPESVRVDVDIYP LKK
> Q8XRI6_RALSO
MRPLSFPRFFSSTTTSTSRTTAPHTEATPEPAAQPPALPRARKTSNLPGP
LRGLLDSRTAKYEKTMAKLSDDTELHQALLGQRPGMNGRMTRAVHQEAKR
RMDSGQWNPQPAATQAAHAPTSPPLPEAPKTSHLPGPLRNLLDHHTAKYE
KASTRFADDARLQQVLLGQAPGINHRMTRAAYQEAKKRADHGEWNPPPAA
APHPDGTETTAAHADHAQTSAPAPTQMLALDNK PAQDANRPPSRSSSMRS
STVHPEPHTEAPHAAPAGPVIDPDTGALNKANLRKLVNDLTHALPAVPQK
GSQTRKLSQALKDQLDAIATIAADGSRKPPHETGLAVRDAFAKAAQAASQ
LSRNVGGGDLADVYQGIAALLNEFKRTHLSASAHDVMSHYANKEAANLER
KAPGIGQGKGGSNIVTSTTGGLTYSLLPKVGYTGPDISVGAGGGKGRIYF
ADDDGDIDHWPSGQAYLRASLGGELGKWAARLTG RLDLLAGDTWFEHNDL
GEMVKLVANRDANRSWMRSAGPNARKVRQALETLVNTLSTAVGRNYTESA
GKPLYLNDAKIAKGFNTFKMTLLAAAIDEKLEKMGSASRFGNLVSKAYPS
TVEVLTQRLHSGGEQPLPLPPRHDVPDSVGYADGALYYRQATASVDASIG
NQTHGGTNLEAGGIFGLNLRGNVAQYFMEFGDAPHAQLDPGHQKDYRSVF
KQHQLLDDACGNGTPPAQLYLYDKMRRTLGQIDMG TPAFPFAKTEQHLYG
DNACVPAQFRNAIAHASREQLLHVNEQLEQLKQTYLEFVASASAVLSKTD
KFMPAGAGRTLSDSREAAFNLINRGIWQGRYPRKDALAHPEAFIAKSHAA
LSLALGCAGTHMSVIKQQMAQRHAQDPHPQPDPNAGNEGAHPVDDNQAAI
READSTYKTVREMMDKTYLPLKNYDVRTKSPLKEAGLWQRWDTALRAQAS
GGAQAGLFNTIMSRLRTKSTQVPLGDLTGTISVSDS AGQVSLSAEARLLY
ADHQLNPSRTGKFWQLTFTAQGGAPITGALINLAVKAAIEKFMPNMRLED
EKFDPHEIIRQAQGLLFDITDGTSIVVKLRQAPDVEHAAMQLQYVRVLRN
KSSGPNVSLSIPTPVGAFTPSISHNDSGQSLAGEIVGTDKSYLIMQHPAF
SKVVDPFFARIAADPSNANAIAGELRQALDADPALRNRYLASPTMMADVV
KEHFDAMAELAAAAAENRPPRILNEFLRYYATEPFAR AANVSAQVASHAP
GSTANGGDPFKIARSPLDERVSADTVNDLQGKREHLQGLKTIEARADYLC
GAEGRPLLEAFTKIVGNMRAINSAAFFHSNPQNIGFRSMLRDPRALAREN
KRIDAALNGKPESTGVLNRVRAHLQPDRINLKEASVTELRALAEPNPQPG
QATGRRQNRQIAEAAQRELARREQRLGAALPPSGGGAPEA
> Q3BY60|Q3BY60_XANC5 Xanthomonas outer protein X - Xanthomonas campestris pv. vesicatoria (strain 85 -10).
MEIKKQQTAGPSSLAPLHDIHTDNAPVADATSPTQTPLHPSLAALTPRSR
SRASSADSEHGASPVPSHDTEPQADSPERASGPSTAATLAARANAALASG
VQQAYAAVSKGASNLWSLADLRTMLQIHADDPAFLQMVRHTDPEQSAPHT
LAACRQQITQLRDAIEAATGLEARFRQQLLGDIKAVEDALQPLE HGTPAT
GRALKSLANLVNLWPLMVPSPLLGNQAKTFAYSIAAATKGVMTLSASALR
PTADGLPFPLMGGQLGRDANEMHFYSVLLNGLFLTTELPKKFGTPSMRHQ
AEAVENNLGFAAAASTACAAMVLTPFLWNSLNVLGNRLQHKLSHLGAGIA
ENAGFQSQAQRLRARLTPGQVSAQLRTQLNEICAALEHGREAFQQARRDF
TDPSQGRELTRTLNAQCTHLLETLDRCSKRLSAALQVDQDQPATL PRQVT
NHDIAPKLALALLGAGVTGLTVYLIQPDRIGTVDLLADSVVVTAVMMQSA
VNAHATRQDAMERFKAMCSGSLVMALALGVEKLSKTFADKSLIEASSSSP
YYAGAIMSLMSMTMPGPMARGAELAMNWGGGQIMRVFRGPDGTALATRMP
SSPEELIQHVQDTARYVQSLSPEQAQEYAQTVADSALQGIEDAGAQAQTR

PASSVTITEITEANEEEALAAASASPEQRSPARTHPSPDQAAASTV PSS
> Q8X2S4_ECO57
MPFSIKSIFSGHTWHQPEISRPIADKSSTKNCILDSTTCNVDGFTVFNRR
SCSFDMRPPGSADRTPQLRLSISEVAWMSKIIETETNNTNKS
> A3T0G1_9RHOB
MTTALGCIADDFTGATDLAGLLARSGVRVSLRIGVPSEPPADPAAFEVIA
LKSRTAPVGEAVAETRAALKWLQAAGARRFFWKYCSTFDSTAEGNIGPVA
EALMADLGTDQTIYCPAFPENGRSIF MGNLFVGRQPLAESPMKDHPLTPM
RDSNLVRLLEPQVTRPVGLADRLTVARGPQALRAELEALKAQGVAHVVVD
AVANEDLAVIAEACRDMPLMTGGSAVAMPLPALYLADGTLSADAPRAEAP
RLDTGTIVLSGSCSAMTNKQVADYTSRGVPAFQLDPLALAEHGPQEALDW
LAQQDLDKAPLIYATANPDSVRAAQEKLGVAGAGEIVEATLSACAVAARD
RGARRVIVAGGETSGAVTRALGVTQLD IGAEIAPGVPWTYCCSGGHQVAL
TLKSGNFGAETFFSDAQERLGS
> gi|21231082|ref|NP|636999.1| avirulence protein [Xanthomonas campestris pv. campestris str. ATCC 33913]
MGLCVSKPSVAGSPDHYATHALEQTPPSTPSSSEAPMSPSLHGLPSLGAR
RARRAGAGPTLQPHEVQQAAYQLGMRLNDRPIEDARDRQRLADATATVHE
TRLALHLGRGNVDSDLRLSNGRSATYSALSYTLSRNDQNSWAGSALALGA
GNCDENAAINARQHAVRMEDGGQMMTVRDYGVPHIYALYQPPGAIEAEES
AVVLDSWCDGPAVRLGDSRWAETYRTTTTVVERFDKPDAIEALDRTNGFR
AEIEDPQTTRHAYARDLGAVFLANHAKHAPGYIFSSMPVIAPDLAEGTRQ
RLQEYSQGTLEDLAADAARQAYGLDEAQPISPRTTTAILEDAERLDALGR
PPLSW
> gi|51591586|emb|CAF25390.1| yopM; putative targeted effector protein [Yersinia pseudotuberculosis IP 32953]
MYGFVCSEKLDNKNIFRKAFNMFINPRNVSNTFLQEPLRHSSNLTEMPVE
AENVKSKTEYYNAWSEWERNAPPGNGEQREMAVSRLRDCLDRQAHELELN
NLGLSSLPELPPHLESLVASCNSLTELPELPQSLKSLQVE NNNLKALPDL
PPSLKKLHVRENDLTDLPELPQSLESLRVDNNNLKALSDLPPSLEYLTAS
SNKLEELPELQNLPFLAAIYADNNLLETLPDLPPSLKKLHVRENDLTDLP
ELPQSLESLRVDNNNLKALSDLPPSLEYLTASSNKLEELPELQNLPFLAA
IYADNNLLETLPDLPPHLEILVASYNSLTELPELPQSLKSLRVDNNNLKA
LSDLPPSLEYLTASSNKLEELPELQNLPFLAAIYADNNLLE TLPDLPPSL
KKLHVRENDLTDLPELPQSLTFLDVSDNNISGLSELPPNLYYLDASSNEI
RSLCDLPPSLVDLNVKSNQLSELPALPPHLERLIASFNYLAEVPELPQNL
KQLHVEQNALREFPDIPESLEELEMDSERVVDPYEFAHETTDKLEDDVFE

> gi|16520035|ref|NP|444155.1| NolX [Rhizobium sp. NGR234]
MSASNLLPMISSNPAQFAQASLAKAFAPRVAQG QQSVLSFEAMLSTNMLD
RIGPLASREDLPPPDAESTLEDLQKDPLALLPPHMRAAIESMDQTPQSAV
VIDDHYVAPAPIQSSRITWNGGSLTKPELQIVAVLNRHKDLCPLSWESLE
AKANDPSTPPDLKAAIEALLQDPELFYAIGSQGDGRCGGKISAKDLSEFS
KHHPQVAAFQESQAQSYAQNYIPSDSAENAQPSVMTENDALRELYRYSEY
LPKNLSLADFKQIVDGEAKTGKCPPQVIAAAQYF VSHPEEWKQLYGGNID
KVHKEDFLQVASSSMSLTQAELDTLKTINSHQELFFGSGDLTRDKLASMA
DDKSLDPKVREAASQLLSDPLLFGLLNNAITGYKTHHGFFDFGGGHTVDS
GNVSKEDFGRFYTNMTTANRTVQQPKFHVPETEAAQNAVADMKMGLADQP
DIKSPKKNGGALMHVVDSVLRVGSKVLDWAATAVGVLSFIPGIGQVADLV
SMTLACEAQAANLLRTAITGGNMKQALIEAGIGVA AQAVGLVSGPGVKLA
IRNGLARKAIEEAATAGINLPLSMAQHYAEGYLNDLKARLAADHPA
> Q8XA03_ECO57
MKVRNPEQISIPASNTTKDPGLTNSQIIRMANLVKKTENMNIFEELWETL
RNLFQSDKHSQTAARQILKDAFYFQNSDDYSKYFTGAVDGKARDKLTHWL
IKFNELKEYAKDPENMAAKASLSPEGTLCVSFFIGDEAIFTLELQLKKST
RTGGIDLSNAYFNGVVICGIDCLEV DLSNAETNNSRWYD
> O34208_PSEAE
MHIQSLGATASSLNQEPVETPSQAAHKSASLRQEPSGQGLGVALKSTPGI
LSGKLPESVSDVRFSSPQGQGESRTLTDSAGPRQITLRQFENGVTELQLS
RPPLTSLVLSGGGAKGAAYPGAMLALEEKGMLDGIRSMSGSSAGGITAAL
LASGMSPAAFKTLSDKMDLISLLDSSNKKLKLFQHISSEIGASLKKGLGN
KIGGFSELLLNVLPRIDSRAEP LERLLRDETRKAVLGQIATHPEVARQPT
VAAIASRLQSGSGVTFGDLDRLSAYIPQIKTLNITGTAMFEGRPQLVVFN

ASHTPDLEVAQAAHISGSFPGVFQKVSLSDQPYQAGVEWTEFQDGGVMIN
VPVPEMIDKNFDSGPLRRNDNLILEFEGEAGEVAPDRGTRGGALKGWVVG
VPALQAREMLQLEGLEELREQTVVVPLKSERGDFSGMLGGTLNFTMPDEI
KAHLQERLQERVGEHLEKRLQAS ERHTFASLDEALLALDDSMLTSVAQQN
PEITDGAVAFRQKARDAFTELTVAIVSANGLAGRLKLDEAMRSALQRLDA
LADTPERLAWLAAELNHADNVDHQQLLDAMRGQTVQSPVLAAALAEAQRR
KVAVIAENIRKEVIFPSLYRPGQPDSNVALLRRAEEQLRHATSPAEINQA
LNDIVDNYSARGFLRFGKPLSSTTVEMAKAWRNKEFT
> Q8X2I7_ECO57
MGSLFNIYKDIFPTLGMYSGLK ACHEKNNLPFDINTEIETIQKQINYDIN
HLNDGLIKRVLNLFIHLISNPDNLELTLNRYSSTTEQIIGRTKRNGLHEF
DDGDLKIIFNRQDDNESVLTVKDKDKDKDKDKDKDKDISHHCNVKTEQLQ
QFIKIMEQKAQLPIYIDKNNLKESIFSVLHNDPQQVDKDQHLPCEKFLKH
ACKSSNSFEVKLDATHQYQHLNNFMISFDPVENQLTIRDNNNKTETFSFT
NLQWENLLQYYKENHQQPNIAGS RNLTDNIDKIKNTISTSEIIECASPEI
RSSVLNDLYSIANFLPDNNLTPNESWKRFCETCERFYVAQKSITGDKSER
LTRKLSISDAGITMTFKIGDVVINTISTAIPEDATGQRCIEGLNLAEMDL
TDIDLSKMALRNVNFNGSILRNAKFSGTICEGVDFTDCDLRNAEFENASL
ENNDFRKVRHLTYVNFKNANLRNSNFNGKVLTGVTFTGSDLSNAYLEHID
FTTVILYETSKIPGIPGTPQIPGT PKVILTGAILNYSDLSGKDLSEYNLT
GILCMYTNFSNANLTNCKISNANFSNAKFYNTNCTGANCSNILFDYAWFD
NTIFIKTLFKNTCFYNVRAKNVYLEGAYLNNDNIVNQANNSTEKQSIDST
DKQANDSTVQQSIDSTVQQANDSTDKQANDNIDKQVNDSTDKQAKNSTEQ
QDSNSFNQARLKKEVNRRFSIPGLTSYQPTYIVEE
> gi|17548381|ref|NP|521721.1| hypotheti cal protein RS05518 [Ralstonia solanacearum GMI1000]
MRAGIEADIVLKGRIDERNIRAPAPSAAPPTPIDPDRAVSPPVGGHQAVR
PAALADLAKAPAASVSTDTAGSFSRRAALPAAPADPGDVLNKALKRIDVW
KQLVVAEQDPRGRAWSPEEEVQYASPVMKAVWTGLDALSALRMQGRATDS
DANQKEVMLLTVLTKASDVVLDVRDRMLRAEWAQLRAVQADTAEAESGPA
EEEDPGPDFEQLPRWLDDLRQVRDALDEVMKRFKSSESPAAVRAGLQPIM
PAVTVQQSFCRTAMQTAHGIIISDRVGWLLMLAEWKGLPGVVARVGELRQ
ALRHRREEVAAASSALEAEADDSWKRRRPGVQSADRPTLQALLKHHEVLQ
DYGRQLDQVGHDMCLDAATMIEMNDADGLWQCMMDIAHSIAGYQEGLEAL
CRAAGNVRPARVEPPPLRVAEPPPPAEPAQPSGSSQRKHGKPGKQPGAIA
GAVPAPRPVSVDKRTDTQKTADGLLRRYPVDRKTLAQFDGDLVRIAQSLG
RDTRTLQRELADPRRDAAITADYLRAVVQDWLGDVGKRGLLREAVAALSS
QDVRVGQLTGRIKALERIERRLDTMEADLLKRDACPRAPHLKRLLNAKEN
PIRSVGQPARLPAERPNDRLGTLFEMRIALKPLSNGQQVAPWVVHIHTRE
PVTADMLGTMPSGDFAAVHLKTDWEKNLGPRWEAVMRALGYPEAKVHRGP
IGMELLGQLFARVKLPASG
> Q8XT13_RALSO
MGNLQIKASSAPYALLSDVSPDSPGSQPPTPKLTRVASPPQALADLPTKG
TGKRPQVSAQPSSPKATTGPSRTVSAELPLSSPAPLQLTSLLPPSESGET
PEKLGAVPISGNLQIKNGQLTSNNETIRSLIHYLGAAADKFANQANLNGM
QVTIRPNPYATAGHEALKEYCSVETALVHADKPPRRKPSKDPLRQPLRQT
PPPSQLKSEDIEGNVLMP LLTSVIESGPAAFGTNDRIMLSGQEVPVHALL
AALAPRVEVNDPDLQLAFNSKLSANTQANAKLGAATLLQMETHKTFALKE
LGMSLAVSSGLGTAWELVAAEGIKGGLKHMGLTAKEYILAAAGIDSSSAI
GIETSDSAIVLSAVQSMNRATASFLRRVWDALPAAAVAGLKSAVGSYPNN
LLQYMSTGSYAADLALNSVTTEVAILAAASGVPGEVKKNEANLQAAIVEK
LRDGLLAVPPREPHMSPEQ YTEIVEKHVQALTRRALDMSPGDGIAQKSLA
FATMVGLIPLILGNKVTNLVAESALRIVRSTIFNPIESIGMNALVLTSKI
DIPGLMSSDATKHAQTVGRILQEAALGKPLQDGEVHKIFHQWDGLTLGGR
AILNGMSATMDAPPEKVMSMLKKDSSPPLSQRIAYESIDYDRV
> avrD1 Pph race 6-1448A|Genbank: AAZ37987
MQDLSFSTIENHLGPAKDRFFGDGFKHVEYSARHV NLTESEANASISLSY
PANWSKKNDSGELIPHLSSIDALTISINLSQDILLNRFKSIDHCWVRRIS
IRAGKKPEEDLRNINAKITKESQGLDSQGDTNLIFGGNVGTMTVQLEFII
PAAHEVDTIKDSTEKNCYSLHFKNRTQFIDDIIFYSPLNAISKLFVANDN
EPHFLPGGIEANYPNIINPVDSLVSHAQIAQALLYKLDGLTRGESNTLWM
RNLNIIAENPAKRRAATRLLVTELKRANIVSLKGEN WRVAEVAGHMNGIT
FSSSVAHLLPL

> SOPB_SALDU
MQIQSFYHSASLKTQEAFKSLQKTLYNGMQILSGQGKAPAKAPDARPEII
VLREPGATWGNYLQHQKTSNHSLHNLYNLRRDLLTVGATVLGKQDPVLTS
MANQMELAKVKADRPATKQEEAAAKALKKNLIELIAARTQQQDGLPAKEA
HRFAAVAFRDAQDKQLNNQPWQTIKNTLTHNGHHYTNTQLPAAEMKIGAK
DIFPSAYEGKGV CSWDTKNIHHANNLWMSTVSVHEDGKDKTLFCGIRHGV
LSPYHEKDPLLRQVGAENKAKEVLTAALFSKPELLNKALAGEAVSLKLVS
VGLLTASNIFGKEGTMVEDQMRAWQSLTQPGKMIHLKIRNKDGDLQTVKI
KPDVAAFNVGVNELALKLGFGLKASDSYNAEALYQLLGNDLRPEARPGGW
VGEWLAQYPDNYEVVNTLARQIKDIWKNNQHHKDGGEPYKLAQRLAMLAH
EIDAVPAWNCKSG KDRTGMMDSEIKREIISLHQTHMLSAPGSLPDSGGQK
IFQKVLLNSGNPGDSEPNTGGAGNKVMKNLSPEVLNLSYQKRVGDENIWQ
SVKGISSLITS
> gi|16764946|ref|NP|460561.1| secreted effector [Salmonella typhimurium LT2]
MPITIGRGFLKSEMFSQSAISQRSFFTLLWEKIKDFFCDTQRSTADQYIK
ELCDVASPPDAQRLFDLFCKLYELSS PSCRGNFHFQHYKDAECQYTNLCI
KDGEDIPLCIMIRQDHYYYEIMNRTVLCVDTQSAHLKRYSDINIKASTYV
CEPLCCLFPERLQLSLSGGITFSVDLKNIEETLIAMAEKGNLCDWKEQER
KAAISSRINLGIAQAGVTAIDDAIKNKIAAKVIENTNLKNAAFEPNYAQS
SVTQIVYSCLFKNEILMNMLEESSSHGLLCLNELTEYVALQVHNSLFSED
LSSLVETTKNEAHHQS
> gi|28901182|ref|NP|800837.1| putative exoenzyme T [Vibrio parahaemolyticus RIMD 2210633]
MKVCRIHTENIGSDLKINSDRHSFRIHSINSSISEKQLLEKLKSCFPSAR
AINNITTHSQRVSSLSAAEKMAVNLYSGDSYESLNRKLRSGQSMTPAEQL
LDIGLSKAVAKEPLDVLTKTYRGSRLRDAFGSVAEGEFGVDPAYLSTSRD
PEIACEFAEEHKRSAFSVIFGV SGFDMALVNGDTVEAEVLYPKATPMRVL
LRDVNKGKESRVLEELSVSETQGHVPALLDALDLAK
> Q12HD1_POLSJ
MADNRWPDGLLLAYYGDDFTGSTDVMEAFSAAGVPTVLFLRPPTADWLKR
FPEVRCVGLAGQSRGRSPDWMDRELPQAFASLAKLGAPILQYKVCSTFDS
SPAVGSIGRAIDIGVPLMPGNWSPMVVGAPRLKRYQAFGNLFAAVDGVGY
RLDRHPTMSRHPVTPMAEADLR LHLAAQTPRRSELIDMVQLRTGDAAARV
QALAGADVPVVLLDVLDEETLAAAGRLVWTQRGRGLFTASSSGLQYALAA
HWRSQGLLPEQPSLPVAAPVPVIAVVSGSCSPVTAAQIAWAQGQGFQAER
LNLPRLLDARESEVEIERVVAAAVGAIALGVSPLVYSAAGPDDAAVRQFD
ATATQAGLARGEAAQRVGEGLAEVMRRLLERAPLQRIVVAGGDSSGEVAS
ALDITALSVVAGLAPGAPLCKAW SERRERDGLEIVLKGGQMGTASFFGAV
RAGRLLD
> Q1YN89_9RHIZ
MSFWCRNAMERPFPGSCICMQEPVRHQAGSYSMILGCIGDDFTGSSDLAN
TLAKGGMRTVQYSGVPTGDAAPDVEAGVVALKSRTIPVAEAVRQSLEALA
WLRSQGCRQFFFKYCSTFDSTDEGNIGPVGEAIARELGAGAVIVCPAFPA
TGRTVYQGHLFVNDRLLSESGMENHPLTPMRDADLRRVLARQTDAPVSHV
PYGVVAKGEDAIADALRQAGADGLCFVITDAVLDADLHATGHAARDLPLI
TGGSGIALGLPENFRAQGLIGSSTALWTGVVGRAAALAGSVSTATRRQVG
VHRDAGHPTFELGADAVMAGEVTAASLTEWVLAQSGVPLIYSSADPETVA
AAQQRHGKEAVATAIERLFAATAAALVEAGITRLVSAGGETSGAVVTGLD
VQSLEIGPEIDPGVPAVRVEGRELALALKSGNFGADDFFAKAAARLGGEA

> gi|28868583|ref|NP|791202.1| type III effector HopPtoM [Pseudomonas syringae pv. tomato str. DC3000]
MISSRIGGAGGVELSRVNQQHDTVPAQTAHPNAVTAGMNPPLTPDQSGSH
ATESSSAGAARLNVAARHTQLLQAFKAEHGTAPVSGAPMISSRAALLIGS
LLQAEPLPFEVMAEKLSPERYQLKQFQGSDLQQRLEKFAQPGQIPDKAEV
GQLIKGFAQSVADQLEHFQLMHDASPATVGQHAKADKATLAVSQTALGEY
AGRASKAIGEGLSNSIASLDEHISALDLTLQDAEQGNKESLHADRQALVD
AKTTLVGLHADFVKSPEAKRLASVAAHTQLDNVVSDLVTARNTVGGWKGA
GPIVAAAVPQFLSSMTHLGYVRLSTSDKLRDTIPETSSDANMLKASIIGM
VAGIAHETVNSVVKPMFQAALQKTGLNERLNMVPMKAVDTNTVIPDPFEL
KSEHGELVKKTPEEVAQDKAFVKSERALLNQKKVQGSSTHPVGELMAYSA
FGGSQAVRQMLNDVHQINGQTLSARALASGFGGAVSASSQTLLQLKSNYV

DPQGRKIPVFTPDRAESDLKKDLLKGMDLREPSVRTTFYSKALSGIQSSA
LTSALPPVTAQAEGASGTLSAGAILRNMALAATGSVSYLSTLYTNQSVTA
EAKALKAAGMGGATPMLDRTETALNNIRHPNRESLPHTFQKSTLSGIPRV
AENAYHMGRGALQLPTQMAVDTVRVVDEGVLNAVASAREALKQPTKDDDA
LRALEEGLLDPR
> gi|1351732|sp|P14727|YAV1_XANCV Hypothetical 122 kDa avirulence protein in avrBs3 region
MDPIRSRTPSPARELLPGPQPDGVQPTADRGVSPPAGGPLDGLPARRTMS
RTRLPSPPAPSPAFSAGSFSDLLRQFDPSLFNTSLFDSLPPFGAHHTEAA
TGEWDEVQSGLRAADAPPPTMRVAVTAARPPRAKPAPRRRAAQPSDASPA
AQVDLRTLGYSQQQQEKIKPKVRSTVAQHHEALVGHGFTHAHIVALSQHP
AALGTVAVKYQDMIAALPEATHEAIVGVGKQWSGARALEALLTVAGELRG
PPLQLDTGQLLKIAKRGGVTAVEAVHAWRNALTGAPLNLTPEQVVAIASH
DGGKQALETVQRLLPVLCQAHGLTPQQVVAIASNGGGKQALETVQRLLPV
LCQAHGLTPQQVVAIASNSGGKQALETVQRLLPVLCQAHGLTPEQVVAIA
SNGGGKQALETVQRLLPVLCQAHGLTPEQVVAIASNIGGKQALETVQALL
PVLCQAHGLTPEQVVAIASNIGGKQALETVQALLPVLCQAHGLTPEQVVA
IASNIGGKQALETVQALLPVLCQAHGLTPEQVVAIASHDGGKQALETVQR
LLPVLCQAHGLTPEQVVAIASHDGGKQALETVQRLLPVLCQAHGLTPQQV
VAIASNGGGKQALETVQRLLPVLCQAHGLTPEQVVAIASNSGGKQALETV
QALLPVLCQAHGLTPEQVVAIASNSGGKQALETVQRLLPVLCQAHGLTPE
QVVAIASHDGGKQALETVQRLLPVLCQAHGLTPEQVVAIASHDGGKQALE
TVQRLLPVLCQAHGLTPEQVVAIASHDGGKQALETVQRLLPVLCQAHGLT
PQQVVAIASNGGGRPALETVQRLLPVLCQAHGLTPEQVVAIASHDGGKQA
LETVQRLLPVLCQAHGLTPQQVVAIASNGGGRPALESIVAQLSRPDPALA
ALTNDHLVALACLGGRPALDAVKKGLPHAPALIKRTNRRIPERTSHRVAD
HAQVVRVLGFFQCHSHPAQAFDDAMTQFGMSRHGLLQLFRRVGVTELEAR
SGTLPPASQRWDRILQASGMKRAKPSPTSTQTPDQASLHAFADSLERDLD
APSPMHEGDQTRASSRKRSRSDRAVTGPSAQQSFEVRVPEQRDALHLPLS
WRVKRPRTSIGGGLPDPGTPTAADLAASSTVMREQDEDPFAGAADDFPAF
NEEELAWLMELLPQ
> gi|53722414|ref|YP|111399.1| hypothetical protein BPSS1387 [Burkholderia pseudomallei K96243]
MRRVLLDRSDEVMRFVADRTGESRYDDYATIGLERDGRIVAGVVYQGHGG
PNVLMHFALDGSRHLATPAFVCAVFTYPFKVLRCNRVTGLVRTDNHEAQ R
LDEHLGFVREGVMRAGASDGTDFILYGMLKAECRFLSGKYVEALRVELAG
SIDGVLASVHS
> hopD::IS52 (h) Pto DC3000 |Genbank: AE016873
MNPLQSTQHSITTPLISGGRPLDAVGPQAQQSHPKRISPSQLSPGAHQAL
KRPSANAEHQRIASLVRNALQDGTLQFQSSNDKQVTYKAPVCLPADTSTD
TDTDTVRTERLINNELTVQARLNDQSEYDIVSAHLHGSSRSI SFDVPSPP
PAHGSASSALSERTHLGRRCKNSQLSPPLAHXXXXXMSRVLSQDAVDGSG
LESPLLSSRAHSRPPSQPEPVHIGSVRRDSGSLVSDNPVVHALLAFAQAD
QAFPPQAASIDGVQLEMRPTRDIEQALKKFKDAFTVEKAQLMSVTNSSER
ADEDINADIHIPLLLKAIEQGAAAFGPNASIGQNSAKAFLASCAPTITSN
DDVLSAFINQKLKGDDDLKVRLGAQELLHVATKKEFQLGGLAG SIGVSSI
LGSAWELGASELLKNAIFGKNFSPSQYALQLAGIDSVPPLMIESMDTMCV
LAIIKGMKGEEWSMSDLLPKALKAGAISSVVSFPNNVLQYAGFKSRVGDL
AANSVTTEAAIFGAASGIPPEVKESEELMRAGLFQSMKDGVMAHPGEGVD
TKETIERMTRHALDIAPGESTAVKSMGLASIVGMIPLIASNKATGLLSEQ
VLRIFRSAVFNPIEAIALNALALGGRVNVPGLFDSDNAKHARVV QTILAR
ASQQMEAGDRNISAEELHQMLAPRSEFLRHVGSAIVNGMNASFEAIPALA
RKLGYGEAPLAERIPYQDLAVRDSPRQPAP
> gi|28871237|ref|NP|793856.1| ATP -dependent helicase HrpA [Pseudomonas syringae pv. tomato str. DC3000]
MTDESPSIDQLLKNLDHAMISERHRLRRQLHELRKKPDEAKLAQWVARVQ
ASCAQVTARRE SVPAMRYDDNLPIAAKRDEIKEALLKHQVLIIAGETGSG
KTTQLPKICLEIGRGQHGLIGHTQPRRIAARSVASRVAEEIGTPLGALVG
YQVRFEDQSDSNTLIKLMTDGILLAETQNDRFLERYDTIIVDEAHERSLN
IDFLLGYLKTLLPRRPDLKVIITSATIDLQRFSEHFNDAPVIEVSGRTFP
VDLWYRPLTSEQDEEGNSVEEDLTVDQAILATLDELAAFERSERKSPGDV
LVFLPGEREIRD AAEVLRKAQLRHTEILPLYARLSPAEQQRIFQSHPGRR
VVLATNVAETSLTVPGIRYVIDTGTARISRYSYRAKVQRLPIEAVSQASA

NQRKGRCGRVEPGLCVRLYSEEDFNGRPEFTDPEILRTNLAAVILQMLHL
RLGEITDFPFIEPPDGKAISDGFNLLQELSAVNRENQLTPLGRQLARLPV
DPRMGRMLLEAAKQGSLQEVLIVASAMSVQDVRERPPERQQAADQAHAQW
KDADSDFAGLVNL WRGFEEQRQTLTASPLRNWCRRNFLNYLRLREWRDSH
RQLSLICRDLQLTVNKEPADYPKFHKAILSGLLSQIGQKADEGDYLGARQ
RRFWIHPSSGLGKKRPQWLMAAELVETTKLYARMVAKIDSDWIEPLAGHL
IKKNHFEPHWEKKRGQVVAFEQITLFGLIVVGRRPVHYGPVDPQVSRELF
IREGLVRGEILSRAKCLSANTRLLEQLDELEAKARRRDILADEDTLYSFY
EARIPAQIHQTATF DSWYKTESQNNPQLLIMREEDVLAREASEVTAAQYP
DTLNLGDLSLSLSYHFEPNHPRDGVTLRVPAPLLLSLPAERLEWLVPGLL
ETKCIALVRNLPKAVRKNFVPVPDFIKAALQRLTFGEGSLPQALGRELLR
MTGVRVSDEAWAEAAQQLEGHLRMNLEIVDGSGKFLGEGRDLAELTARFA
EASQAALAVPQTAKSQQPVQAKAFAAVAQKTQQNIAGLSMTVYPALVEEG
GAVKEGRFSTQAEAE YQHRRALQRLLLQQLAEPAKFLRNKLPGQTELALL
HRELGRIDALVEDILLASLDSCVLEGEAELPRDGAGLLSLAERKRADWTE
HAERFAKLALEILKLWHGLQKRFKGKIDLSQAVALNDIKAQLSKLVYPGF
VRETPAAWLKELPRYLKAIEMRLEKLPGQVQKDRVWSIELAGLWAQYQAR
ADKHAQEGKRDPELALYRWWMEEYRVSLFAQQLGTKMPVSDKRLSKQWSQ
VDS
> CP0132 icsB IcsB, invasion protein 114564:116048 reverse MW:56420
MILKISNFIDASNTKGPIRVEDTEHGPILIAQKFNLKDLFFRTLSTINAK
INSQILNEQLKNYRLENQKSLLLFLNTLASEKSAESAFAAYEAAKNSIQH
SFTGRDIKLMLNTAERFHGIGTAKNLERHLVFRCWGNRGITHLGHTSISI
KNNLLQEPTHTYLSWYPGGNVTKDTEINYLFEKRSGYSVDTYKQDKL NMI
SDQTAERLDAGQEVRNLLNSKQDQNNNKKIFFPRANQKKDPYGYWGVSAD
KVYIPLSGDNKTKDGKISHNLFGLDETNMSKFICKKKADAFRQLANYKLI
SKSENCAGMALNVLKAGNSEIYFPLPDVKLVATPNDVYAYANKVRQRIES
LNQSYNEIMKYIESDFDLSRLTQLRRSYLKSFNKINLIHTPKTFKPLSIS
LYKHPTENVSSEDFDAVINACHSYLVKSAPSNMTRVLNELKTEATDKK EE
IIEKSIKIIDYYNSLKSPDLGTKLYIHDLLQINKLLLNNSHSNI
> Q3BY51|Q3BY51_XANC5 Xanthomonas outer protein B - Xanthomonas campestris pv. vesicatoria (strain 85 -10).
MKAELTRSHSLSSLERIRHPESDMGNDCDLEVGAQPKHSGFCQSEDSRLP
TTRPPRRRSTSSGATSPKSTVQGSGHTDNSGLETPLLISLTGLQKPRHMG
LVRRESSRLVSADPVVHALLSFAQVDQPFPPQVASTDGVRLELVSRRDPE
KALEEFKDAFTVETAQLMPAANSSERTAEQIDADIHIPLLLKAIERGSAA
FGPSALIEMADGSQISAKAFLASCAPDVMSNDDVLSAFINQKLKGDEDLQ
VRLGAQELLYVATKKEFQLGGLAGSIGVSSVIGSVWELGASELLKKAIFG
KNFSPSQYALQLAGIDSVPPLIIETMDTMCVLAIIKGMKGEDWSLRDLLP
KAMKAGAISSIVSFPNNVLQYTGFKSGLGDLAANMTTTEAAIFGAASGIP
PEVKESEETMRAGLFQSIKDGVMACPSEKMGPEEAIEQMARHALDVAPGE
STAVKSMGLAAIVGMIPLIAGSKATGLLSEKVLRIFRSTVFNPIEAIALN
ALALGGRIHVPGLFESDNAKHARVVQTILARASEQMESEAREITAEELHH
ILAPRSEFLRHVGTAIVKGMNASFEVLPALARKLGYGETPLNKRIPYQDL
AVSNTSHQPAPEP
> Q8X3G1_ECO57
MPFSIKNRFSSSQVHYPEISGPIKDKPASKNCILTSTTCNVDSYTVYQKK
ACSFDMRPPGAGERTPKLKLSVTEMTWLSKTIETEIHNTKE
> NleC_Crod
MKIPSLQPSFNFFAPAGYSAAVAPNRSDNAYADYVLDIGKRIPLSAEDLG
NLYENVIRAVRDSRSKLIDQHTVDMIGNTILDALSRSQTFRDAVSYGIHN
KEVHIGCIKYRNEYELNGESP VKVDDIQSLTCTELYEYDVGQEPILPICE
AGENDNEEPYVSFSVAPDTDSYEMPSWQEGLIHEIIHHVTGASDPSGDSN
IELGPTEILARRVAQELGWTVPDFIGYAEPDREAHLRGRNLNALRQAAMR
HEDNERTFFERLGMISDRYEASPDFTEYSAVSNIEYGFIQQHDFPGLAID
DNLQDANQIQLYHGAPYIFTFGDVDKHNQR
> avrB4-1 Pph race 6-1448A|Genbank: AAX121 08
MGCITSKPLVSSPQWHNSATNSENLETGQRSHKASLYGAMSGSPERSELT
WHQQSLVGVARWPDKEYNRDHVPLQMEYGRSFWNESRKIGSALANGEIQN
FEDLWEKARDWRCSMANHDENIFKKPRNSYNEFPFTTPLINQYNYIKERY
SARTDGSLQKLDDEGLLPPAKEFLITDKIFGEPISLTKIVCSSDSSAHRD
QRRYSDLWSRGLDYGEPHYIQHTSSEEVPKILHHVNDLFNEVLQSNLST K
KALKFLGEIHWWLAHAMPDERGSAAKSELCVRAIAQAKGLDLPPMKSGIV

PDLEAMTMSREQFIKQYPSMFDD
> gi|18677797|gb|AAL78294.1| XopA [Xanthomonas campestris pv. vesicatoria]
MINSLNTSHLGVDSSFMQVNPDQFQKFDSNQSNQGISEKQLDQLLTQFIF
SMLLQDDNADDSPNSDKPTDFPSPRTQMLMNVIGDILQAKNGGRLGGLSD
GGLNTSLSLSGDTASMQ
> gi|15605307|ref|NP|220093.1| hypothetical protein CT578 [Chlamydia trachomatis D/UW-3/CX]
MSLSSSSSSDSSNLKNVLSQVIASTPQGVPNADKLTDNQVKQVQQTRQNR
DDLSMESDVAVAGTAGKDRAASASQIEGQELIEQQGLAAGKETASADATS
LTQSASKGASSQQCIEDTSKSLELSSLSSLSSVDATHLQEIQSIVS SAMG
ATNELSLTNLETPGLPKPSTTPRQEVMEISLALAKAITALGESTQAALEN
FQSTQSQSANMNKMSLESQGLKIDKEREEFKKMQEIQQKSGTNSTMDTVN
KVMIGVTVAITVISVVSALFTCGLGLIGTAAAGATAAAAGATAAATTATS
VATTVATQVTMQAVVQVVKQAIIQAVKQAIVQAIKQGIKQGIKQAIKQAV
KAAVKTLAKNVGKIFSAGKNAVSKSFPKLSKVINTLGSKWVTLGVGA LTA
VPQLVSGITSLQLSDMQKELAQIQKEVGALTAQSEMMKAFTLFWQQASKI
AAKQTESPSETQQQAAKTGAQIAKALSAISGALAAAA
> SOPB_SALBN
PAQEAHQVAVAAFREAQVQYLNNQPWQTIKNTLTHNGYRYTNTQCPAADM
KIGAQDIFPNAYQGKGVCSSDTTNTQHATNLWMSTLSVNENGKDKTLFCG
IRHGVLSPYHVKDPILRQVGAENRAREVLTAALFSQPALLTKALQDEV VS
LRLVSVGLLTTSTIVGNEDAMVQDQMRAWQSLTQPGNVIHLNIRNKEGEL
RTVKIKPEIAAFNTGVNELTLKLGLGHQASDNYNIGALHQLLGHDLRPEA
PPGGWVGEWLAQHPDNHAVVNTLVRQIKDIWNSKLHHTDGNEPYKFAQRL
AILAHEIGAVPAWNCKSGKDRTGMQDAEIKREVISLHQKATLTPLASLPD
SDGQEIFQKVLLNSGNLEIQKQNTGGAGNKVLKNLPPEVLNLSYQRRIG D
ANIWQLVKGLSSLVTS
> gi|21243950|ref|NP|643532.1| avirulence protein [Xanthomonas axonopodis pv. citri str. 306]
MGCTISTTNNAPHSPRQEDAPPLPPQTRQSFVGVVNGLLSDLPKRRRRGG
SLSDPDISLAGYLLSKAVIGDPVEPQDIPRLHKANNTMQETRARFPYGRG
NVATDIAVSDHASSQHAQAAHDVFVDLVRAAPPASMLTNPT LGHAVVSEF
VQGGHCAGYAAVATMRHVQKLQPEESVHYVQHNHQGHDWAESRVPDGHHK
TIVLDPWAQGPAVFASDSRFAANAQHTQERLALNAKDGDDIAAKTAAGAQ
YLLENCLPLTETHLKRLKAQEFHCAPEEVWQPQPVVSDAFRRRVRQSLAT
LTNSSELGLSRAEQSSKALKKMSIKSACALGFGKKAASAAAEGIAAAAYQ
LCEQSQ
> gi|28868061|ref|NP|790680.1| ribD C-terminal domain protein [Pseudomonas syringae pv. tomato str. DC3000]
MKVTVFSQISIDGKLTMGKGASSKPLFQNFDDDDMRFIHKFRGEVDAIMV
GRNTIVTDDPQLTNRYESGRNPIRIIPTTSLDLPTSASIFKSPEKTIIAT
SEQARDHEMVKHIRACGKEVLFAGAKHVDFTRLFPMLEARGINHIMVEGG
GHLNWQVFNLDLVDEIILMQVPIIIGGAAT ATLADGVGYRDINMANSFTL
HALEARPHYNLMHFKRESNNRSPY
> A2I8A2_PANAY
MWHNINPYGAPPFSNRAPDGEPPAADPPPQGTAGGTPPLRDDPRAAPYPE
DEVLTEWFSDCATIGGTDHGVIIDYGRALRRFSGFLSQNGLPALSAPGRL
DDPDRLRRDAHVYIDRFPQPTNKKYLIPSLNRLIDLVTSGADRITIRGPK
RPRRTVPDADEAQIKAAFPGRDGYSVNLTTSARAYSAWLHAN GRPGLSDT
DWLLSEQADADAAAYSWAHPGYAGRVNIVLAHLRARAGGEVPLIAKNQNT
RTIPLADQRLARAYRDVAEDRAGACGIVYKKGKDGRDAIDRSITLLRSFS
AWRTARGLPALTDHLHDPTLLTDAQMFVEEKSQAGRALNTTRRTLYMLNA
ALELLRASFPPGTPVTLPEEPADSFALPGSDWSGWGMSLSLGGHGPADSD
SVFGGLAPLDSRERFSSDGLSGTPASGLPETGEVNRPGPDGAP FVSMRDA
VPPFRPDGVGFASGEGLSCLLDSILQCYHNIRRGHGAPRGLTDWLDGEVR
RVREALSAQGVDLVPARGEIDIYGGPGSYLAGAMGLRLQVIQAEFQEEPD
GTYVRYTSHPEMGRSDAPLVRLLHTPGHFQPLWG
> VP1667 VP1667 putative outer membrane protein PopN 1783656:1784549 reverse MW:33232
MSIINSQIATNTKFDASVRNGLESS RADSAVKGSYRGETVRVHNATQSLF
DAMEELTSLGSEKAEKDLTKRKIKDGGVRVNEAHELVSDYLRKVPDLEKN
QKIKDLAAKMAGGNISTIAQLQAYLNGFSEEKSHQYLALKAVKKYLSANP
ESKHLLALIDQAILKIEQNPDSWSQIDTEIRVSHFADEFSKEQEFSSLHQ

LRGFYRDTVHSYQGLGSAYQDVVERFGEQEVSTAVDFMLQGMSADLSVQG
SNIDSVKLQLLMSDMQKLKTLNTLQD QVGRLFQMFKPERMSHGLSGF
> hopV1 Pph race 6 -1448A|Genbank: AAEZ01000000
MLNKPTLNGIAPTAQAAASHAVEGPTSPTPMRLDAAQGQRPLPPMDAPSS
LRLRASGTSSGGEPQAAQENSGSAAADNNGAARAQREPGIDPVRFSVKLM
VGTLALSTGCRLTRHPDDFAKDPGGSIWAAMSLKHRSSQNDLDQGNRTVL
ERYGAYIPKDSNCFKAKADVTHDIPPGVAGQWNV KTRQVKLNPNIALESH
PAEVAGHEFIHCYTHPEFRGRHIDHRHWKALNEGLTTHLTEKLPTPKRLL
PIPLAKDPYHGFKLATGDSWPAAAKRIEGAVGEDTLLKAFFGGDDDAISE
VAKAAAQIYPRLASSRTEQELYRAGMMRGSQQLAECYAGALLASGQPLPE
SWSRNMLPVFSFSDMQPEQAKKARLQAEQSQERMGIIFDAAFFSPDLKTQ
RQALGMLREDLLMHWENVVPDKGCPIEALALKATG KERCSSPPGRYRFHF
DQRTGQRQRHYLQSGARRFQGLLGGAEKLRVTGLQACKVKLAAGFLGPHQ
KHPQLHDIAQIKVLLPEHIAQFVEHRNSLDLSIPVCGRRTRRGVWVSEVR
QGAADEHQAGRGRHGHGLRDWKIADAGTLNAVAHGVTPQWMLMLQ
> Q8X834_ECO57
MKIPSLQSNFNFSAPAGYSAPIAPNRAENAYADYVLDIGKRIPLSAADLS
NVYESVIRAVHDSRSRLIDQHTVDMI GNTVLDALSRSQTFRDAVSYGIHN
EKVHIGCIKYRNEYELNEESSVKIDDIQSLTCNELYEYDVGQEPIFPICE
AGENDNEEPYVSFSVAPDTDSYEMPSWQEGLIHEIIHHVTGSSDPSGDSN
IELGPTEILARRVAQELGWSVPDFKGYAEPEREAHLRLRNLNALRQAAMR
HEENERAFFERLGTISDRYEASPDFTEYSAVSNIGYGFIQQHDFPGLAIN
DNLQDANQIQLYHGAPYIFTFGDVDKH NQQ
> Q3BW96|Q3BW96_XANC5 Xanthomonas outer protein P - Xanthomonas campestris pv. vesicatoria (strain 85 -10).
MPKIESTKQAPPDPAQLIQTAPTPSGSGSAASSSAASAAPSAQLGGLSIR
PRRLGRASRANLPAASSTNAQDVMAALFAAAPVPQGPDPFEVLSKASSRL
KRFNDLAQKRDAPADIRALEARLESGKSAIESARQALQALAEL NIQKRMP
VDNVEEHENFLVPHFAVAASLHYGACEKMIDQKMATEFGHVNTQRVEMVV
LSADAMRLQQPGPWLALRGHLLNALAAMEEVCQRIRSPMTGQGPRDSLND
HLPVVRAMMGSCHERMQTVRGMLVEIHSRRLADEASECGLPQVLDGLREL
EEIDSLHHAEVGEALKHVIQVHVERFSSTEHSLTPEGLARCKKVLVGYAE
IRGRAGMQLCVDAAALIEAGEHARDHLWPTMLDLAQALADQRQA IVDMCE
FAVQDRQLIGAAAASPGQAPASLTSSVPTTTTTTGKSSRPRRARVRTPDA
SSSAAAPARRVMDERSAAQKQADEILKGTRLESLPVAELGGDFIALAKRL
GKDTTDVERLIGDSRYDAATAFDSARITMQGWFGSSERVLQLKGKLRAGD
ARIDHLDTRLRLLQRIEHDFKRREADALKTDPQPRAPHLERLLAMNGLAR
VTSPRRLRSEDDIGDRGRLFEVRIDHTPQSNGNIPRPWFVHVHAK RPVTP
RRAARARL
> gi|53722553|ref|YP|111538.1| putative cell invasion protein [Burkholderia pseudomallei K96243]
MSSGVQGGPAANANAYQTHPLRDAASALGTLSPQAYVDVVSAAQRNFLER
MSQLASEQCDAQPAAHDARLDDRPALRAPQERDAPPLGASDTGSRASGAA
KLTELLGVLMSVISASSLDELKQRSDIWNQMSKAAQDNLSRL SDAFQRAT
DEAKAAADAAEQAAAAAKQAGADAKAADAAVDAAQKRYDDAVKQGLPDDR
LQSLKAALEQARQQAGDAHGRADALQADATKKLDAASALATQARACEQQV
DDAVNQATQQYGASASLRTPQSPRLSGAAELTAVLGKLQELISSGNVKEL
ESKQKLFTEMQAKREAELQKKSDEYQAQVKKAEEMQKTMGCIGKIVGWVI
TAVSFAAAAFTGGASLALAAVGLALAVGDEISRATTGVSFMDK LMQPVMD
AILKPLMEMISSLITKALVACGVDQQKAELAGAILGAVVTGVALVAAAFV
GASAVKAVASKVIDAMAGQLTKLMDSAIGKMLVQLIEKFSEKSGLQALGS
RTATAMTRMRRAIGVEAKEDGMLLANRFEKAGTVMNVGNQVSQAAGGIVV
GVERAKAMGLLADVKEAMYDIKLLGDLLKQAVDAFAEHNRVLAQLMQQMS
DAGEMQTSTGKLILRNARAV
> gi|29840710|ref|NP|82 9816.1| hypothetical protein CCA00955 [Chlamydophila caviae GPIC]
MTSGVSGSNNTDPSLAAQLAQNASQAASKSQGQQKSGNAQETQEAAAGFE
DLIQETQTQGTSKKEATSQTTKSSKTDKSAKSSSSTSVASASSTVTAQAV
KGPKGLQQNNYELPQLPMPENTEVNGVVIKKGMGTLALLGLIMTLLAQSS
AKSWSSQFQQQNQAIQNQVAMAPEIGNAIRTQANHQA SATEAQAKQSMIS
GIVNIVGFAVSVGGGLLSAAKSLGGIKSAAFAKETAGAAGSAASSAASQA
SKMATDAATTAAKTASTAASGVAGSAAQGAAKAAAGLTDDIAAATAKATA
GTAKGGGLFGKVLNTPGWKDKLTRGMNVVKTQGGRAAAFAGRALSTSMQM

SQMVHALTAGIDGIVGGVMGAEIAHHQRQAGMAEAHAEELKQLSSIQGQY
AGQAQQLQEQSQQSFNSALQTLQNIADSQTQTTSAIFS
> A1JQA7_YERE8
MAIGPVGGNSRFLVGQRDKSSSAAQASDDADEAARGQGVIDNSSEYASMS
MLAASHVRRRGPTTDSKEEWTRFSERILDAQADEKILRVEGALNNKLMTQ
QQLHAFLLQYFTDPSDLLMVLAALIHRSRLKRKQLEQLEALREQLEAEDF
DRSAQAGINIALVAKAFAQKMQQSAGNLRMLYREFLCYDGPAIYLYEQWA
EEMVAQERENIIRYLARALACDLQALPLGNINVSEF GALFNRVSRLREMK
SLDDTFRQRFSHTDFAFINQSGENMLSKLFISGIRGQENFNDSLLTFYSR
QLGALSTDMRARFLQILIIAFSILPTGIFSSLEEREHFIDGLKENMSHFM
EQELAMARRVLHDEEENIP
> Q3KHX5_PSEPF
MSDLNTLRASLKSGEHVFADTLSFIAAGYDYQPQAFNNGGVENAAGQNEG
SCKTLGLALLEGLSDEEALLAFGEHYRSVVATPEGSDHGNIRALIQHGLA
GVKFEAQPLTRR
> hopPmaA_Psyr
MSPAQIIRTPHSFPPSFTGTSSSAENSHAQSPQQVLTRAFVASGELNAAF
GRTSTASEQDFTSLLGTLQRELERKTLSFPDIAELANQLAEAAKGDQGGH
WLGRDEQQTLKGMIDRCKSQLAHTHASDASYDPLAQVCENLKTARLHQSI
SQMTGEAHAKVRGVPDLLALIQLDPDVLAEKPVGMTSYVNFGSFICMAKA
RTAELSEDLRSDPNEVALLLHPHADT ILELERLPDALAALTENCPDTPTR
DDLRSLAKETGELLQQLRANDLLPRSEEVSSYQGETSVRSREVVEPKLTL
CQAGGNGQGQLEASSARPESLRYAPTRAASSGSEARVPGQAVGGKIADDA
QKVAGLYAEKKRTNWTQANGVAGKISHKIQSLLGMRDAGSRVQAFVAFMA
DGKGRPGATMLDLGDGWMRATRVIKGEAALIDFQCDSDGKVVDARHPGRF
PVLPQGNEREAFKTVLQELKFRGAETL SKVPVYYVNRNTRGYVIPTHGYV
VAGHPNRGRKSGAVLYGVGGDPKRGPVALDEKLLGHLVGRSDSKTSSKLS
APVKAAISALAGASFATREDFYDAYCAVRGDAVDPLERHNEISSIYRLLP
LSTMEMWPKKADDYRVARPAAPERDLRAFENLPKDIGRKAQLKKVSNVDS
IDLLEAKRQFTLHQLYQDEMLGRNGTGVPSADFKPKVDAQRRDQLVASTP
KFQRLPPHTTDKVGNCNTGASSLLQRAV DTYTEKNNLPPEKVTAASIFGI
GSSHRLAIWDPLDGSSSNKSSKDR
> gi|34498029|ref|NP|902244.1| secretion system effector SseE
[Chromobacterium violaceum ATCC 12472]
MQEMNIKPDVVTQFLLSQGLVPEAEYFGNSHVLVGQRVKLLDCELVYRLE
DEELIICDFVAKQPVQGSASAVAAFIHLIHKIEKSVPTVKRVRGLFLESM
TRPELNQIRMRLSKVLEAQGATWRDIDGELWLVYEIGLAQAV
> gi|27376898|ref|NP|768427.1| hypothetical protein blr1787 [Bradyrhizobium
japonicum USDA 110]
MSAATPLQAWLNGRRARRRDRWTAISIDPRAKRRLDSLHCLQWVLVAEAY
PGHSCLSARLWDRTSLLVGRWRVSHSGGLAVLRCAAPAHSLHFATEETIS
MNTEQLVTRIPSATSDLESS EPGSPAAPLWGETLAGVHTSAAGAVASPEE
VLANWAAEDGQGQHGDRQQAVSRTSAWRGTGDLNQPLDLSSRSLTALPAP
ILAEVRRLNVDHNQLDSLPETLSTGLQRL
> A3HQ93_PSEPG
MMPAKRCTACPTPSGKPNTRKKPAPNNKRRSKKENLVTDLNTLRASLASG
QHVFADTLAFIADNYSYQPQAFNNGGVENAAGQNEGSCKTLGLALLEGLS
DQEALLAFGEHYRDVVATPEGSDHGNI RALIKQGLAGVKFAGQPLSRKA
> A0FZ98_9BURK
MAQALLGCIADDFTGATDLANMLVRGGMRTVQTIGVPDANIRIDADALVV
ALKSRTAPAREAVRQSLHALEWLRAQGCRQFFFKYCSTFDSTNKGNIGPV
ADALLDALKDDFTIACPAFPENGRTIFRGNLFVGDVLLSESGMEAHPLTP
MSDPNLVRVLQRQTKSKVGLIRYDTIAKGAEAIRARIEELKEDGVRIAIA
DALSDSDLYMLGKA CRNLPLVTGGSGIALGLPQNFHSADLLVYAGDAADL
QEIDGRSIVLAGSTSKATNAQVADWRASKPSFRIDPLALSRGEPVVERAI
DFTLKNDETVLIYATSAPDEVKAVQKELGAAKAGRLVEDALASIARGLRD
KGGVRKFIVAGGETSGAVVQALNVRSLRIGPQIDPGVPATQSIGRECELP
LGLALKSGNFGTVDFFTKALKALDGQTQMSEF
> STM1401 sseD translocation mach inery component 1486467:1487054 forward
MW:20592
MEASNVALVLPAPSLLTPSSTPSPSGEGMGTESMLLLFDDIWMKLMELAK
KLRDIMRSYNVEKQRLAWELQVNVLQTQMKTIDEAFRASMITAGGAMLSG
VLTIGLGAVGGETGLIAGQAVGHTAGGVMGLGAGVAQRQSDQDKAIADLQ

QNGAQSYNKSLTEIMEKATEIMQQIIGVGSSLVTVLAEILRALTR
> Q8XYF8_RALSO
MGYHFWAGWRPTSVADSAQASTSAPQAGAPASQPARPARAPAGSSILRGL
ARLRARWVAPPVPFFPPEIWTEIARGTKPADMQSLRAVSRAVKMGAEASI
PQLTITHRDSGKRPPSVAGCLELNRLTLVGHFTDAYLQRLPASLTALDLS
RCEGPITSAGIARLLALPLERLDVSGCGLDAESARLLASHPTLTVLNISR
NAIGDDGAAALAANPKLTSLNVGRNGISDAGARALVACAGLTKLD ISDNR
IGDAGARALAGSAKLNKLDAGDCGIGPEGARALAASQTLTRLDLRYNEIG
VGGAEALAANPRLTSLNVCGNALGSAGAALLAASAALTELDIGNNGIGNA
GARALGANATLVKLSVANNGIEEAGARALAASTTLTALDIGSNRIGDAGA
QALAANDTLVTLDASANWIGDPGALALADNTRLATLNVGANRIGEAGMRA
LEASTTLAVLKT
> Q8Y164_RALSO
MKRLFRALGVSQPTRTYT PTTDTTSPAAATPESSRNSSPESSRSPSPVRS
RSRNHPDLSDLPSRPRRKAEMLAESIQSARDRGVDPEFISYAETTLHNLA
SHTQPSVMETNLDIRHLDTIVQSYRYNNLNLQAFNSREAFVASLVEGQGS
GRAERGVVRDYPNLHHFAADVRHHEDGRTTVIILEPASAGNQENLPGYTE
LASALRYNLGSQCRMVVIEAEAQKSLSDCVAFALDFALAAYQERRTTFDQ
WHENLAAYGTIADNGVQDK KYGPFDRGLYHNYGIHLIKGWGVLPPVFYKH
AHSRETLKGVEKRQPGSLETDVSTGRNKDGAESLEERMEAFSDRHGFRPR
NISIEASRARKIRHALES
> Q8XE85_ECO57
MMKTITKQPILFTDVPVADLRNSMKQDLNQNLIERLWNKIRDFFLDSDKQ
KAFKSIHKYINTLSVLNYNSALTPDPNFNIDATSDLDSYLKLDFDRLSPK
QKQTTLCCFWNKIASSLPEPYNSTIKHNIIFYKDGEN LMIRGTISIVNEV
VKTYSLPIEKDDNGYYDFSGLYLAHSNISGKDPNKDPDIDFGIDMGNCNC
SNVNFEHTYFYGVKFTNANCTNANFNNCRFKKCDLTNMNCTGAILDNAMI
YGKEKEPEMQYPEADQIIQRITYQKSDGNETKGMILTNCSCVKTTFNWAD
LSESDCQNVDFSEANLSNTILPDIVRMKGTKLYRTDLFNPILKTEAESTE
EKDISPLAKIILDYIESDKNPESLNFEEKSTVIKIKQD IDNFIFYNQHLK
KIFNRAMNLQEKISRKKYNEFFKYIQAEAKQYFKDQYKLTKNDYLKKVPL
TAQLIAKYKMDDQLDQLLVTREIQDEIKSKIQDKIDELSKNLFNTMTETI
ENNFDDIFRQQSENMSNYYEFVD
> Q08678|AVRV_XANCV Avirulence protein avrRxv - Xanthomonas campestris pv. vesicatoria.
MCDSIRVQFRSIQKMVVKMKKFFRSLGVG GSSSSRFQHHIPEADSAPSSK
ASTPPASPPPDSPPSNSAFSALPTRPRKKAEALSDAVESRGHLAPPSLVS
YANATLDQLRRNEPISESLRLMDIENLPHLVRSYDNRLNNLNLRSFDTPG
QFLHDLSRWHKTGLPLRAVVRLDEDPRRWHRVAFDVRNHESGHTTIIALE
PASAYNPDHMPGFVKMRENLTSQFGRKISFAVIEAEALKSIGGCVIFSLD
YALAAYQERSTFDQWHKDLRKKGNIKGMTP ESQHLNELGVYLLKGTRLLP
ANFYKHAHSRRTIDELEADQPGASGTDVRSGRAAVYKESLSRRLEEFQVQ
RDKTYSMSIEASRARKIRHALES
> gi|17546893|ref|NP|520295.1| PROBABLE TRANSMEMBRANE ABC TRANSPORTER PROTEIN [Ralstonia solanacearum GMI1000]
MADVMHGSRLALPVSLELRALPSRAMAYASPLIALALTLVFGLLLFALL G
KDPVAGVRVFLIEPLATKRAIGEVLLKTVPLVLCALGLSVCYRANVWNIG
AEGQLIVGGIFAAATIIHFDTPTPAIPGGAALLLAVVAGLAGGMLWAGLT
ALLRDRFHANEILVSLMLTYIAQLLLLYMVNGPLKDPNGMNFPQSMVFDS
AFVLPTLVAGTRLHVGFLFMLAMAAAMTLFVFRSFAGYRLQVGGLAPQAA
RYAGFSSRAALWTALLVSGGLAGIAGAFEVAGPIGQLLPSISPGYGFAAI
VVAFVGRLHPLGTVLGAIVMSLFYIGGELAQSRLGLPAAITGVFQGMLLF
FLLACDTLIEYRLRWRAHR
> gi|16764754|ref|NP|460369.1| secreted effector protein [Salmonella typhimurium LT2]
MKIHIPSAASNIVDGNSPPSDIQAKEVSFPPPEIPAPGTPAAPVLLTPEQ
IRQQRDYAIHFMQYTIRALGATVVFGLSVAAAVISGGAGLPIAILAG AAL
VIAIGDACCAYHNYQSICQQKEPLQTASDSVALVVSALALKCGASLNCAN
TLANCLSLLIRSGIAISMLVLPLQFPLPAAENIAASLDMGSVITSVSLTA
IGAVLDYCLARPSGDDQENSVDELHADPSVLLAEQMAALCQSATTPALMD
SSDHTSRGEP
> gi|28871892|ref|NP|794511.1| type III effector HopPmaI(Pto) [Pseudomonas syringae pv. tomato str. DC3000]

MINLTHIASSLARAALSDSTKPKMERAINVASHIAGKVALQVTSSLLEQK
GLLNERQQKGLSMILKALSGKEPVNNVETHEGGGRFNLARAAFDVASVVW
ERDKSMHNVMSFLGVSDSKGKMLFSLGKKLADAMAKPEPGKDNSEATNAR
HAYFSSNLKLNKLMNDLTDQVFNKIRQSNGDRVRRPMPEPFWRPYGAQQQ
ARPQTPPGTRPQANSAPPPPPKAEPRPASGRP DGAQQQARPETPPRTRPQ
ANSTPPPPPKAEPRPASGRPDGAQQQARPETPPRTRPQANSTPPPPPKAE
PRPASGRPDGAQQQARPETPPRTRPQANSAPPPPPKAEPRPASGRPDGTQ
QQARPETPPRTRPQANSAPPPPPKAEPSAGGERPSTARPNNTSAADASAR
VGDSAPAKPPVKPLYEHLGLTDMSVDLSAVKKAYRDAAMKNHPDKNRGNE
AEAAERFKVISNAYKILSDPELRKAYDNGRINE AGNRA
> gi|34497721|ref|NP|901936.1| probable SPI2 translocated effector [Chromobacterium violaceum ATCC 12472]
MAENGRSGLFALALKNREVQPSPAGEGKTLDHIKRLVVIGDSLSDTEGRM
RSKTLGIMPSSRQYYEGRFTNGFAWPDFAASRAFLDKPLVNYAEGGAVAG
KYSKLNPTFMFISNMGRQIKKHDFKPGDMAVLELGANDYMTF GKTNVDKV
IHVYEKQIGKLVAKGVKNILVAGVPDLSKTVSARSPKQKPGHADKLAALS
RQHNEQLRQSVEKLRERHRGEGVEIRFFDIGGKLDYLMSLAGEVDYDTRF
NKHEGYIDLPRVFGFAGDTRPLDTSHRHVFHDEVHPSQEVHQILASHITD
FIREEYGRPEEAAAATDSRLERAGVYAGASSAEQPVR
> gi|28871729|ref|NP|794348.1| candidate type III effect or HolPtoZ [Pseudomonas syringae pv. tomato str. DC3000]
MKISGSTSPAHTSTNSAQKSSSKGLLSGLAKHFKGMLVSGNTSGHSALGH
YASSSSGSKGKAPVRDDYSNGPQTRLNNTPLKRALARELDRFGYGSSATE
SFDRSLQRKDKNPELGKV
> gi|4760784|dbj|BAA77400.1| translocated intimin receptor [Escherichia coli]
MPIGNLGNNNISNNLIPPAPPLPSQTDGATRGNGSSLISSTGSLGSRLLF
SPLRSSIVDTVDSRDVPGLPEHPLRFATSETCLHGGFEVLHDKGPLDTLN
QKIGSSVFRVEQQPDGTHAAIGVKDGVEVSVTLNSSELQSLQSLDTEGNG
RFVFTGGRGGSGHAMVTVASDISQAREKIIAKLDPDNHGGRQPKDIDTRS
VGVGSASGMGDGVVSETHTSTTTSSVRSDPKFWVSVGAIAAGLAGLAATG
IVQAVALTPAPDDPTTTDPDEAANAAEAATKDQLTKEAFQNPDNQKVNID
ELGNAIPSGELKDDVVAQIADQAKVAGEQARQQAVESNAQAQQRHDDQQA
KRQQELDLSSGIGYGLSSALIVGGGIGAGVTAMLHRRNPPTEQTIATTHS
VIQQQTGGNTRAQGGADTTGVENASLTRRDSQASVASTQWSDTSGDVVNP
YAEGWMSRNNPSLLAPEEPIYDEVAPDPNYSVIQHFSGNNPVTGRLVGSP
GQGIQSTYALLASSGGLRLGMGGLTGGGESAGSAANAATTPGVERFV
> gi|15596906|ref|NP|250400.1| translocator protein PopD [Pseudomonas aeruginosa PAO1]
MIDTQYSLAATQAAIPSEPIAPGAAGRSVGTPQAAADLPQVPAARADRVE
LNAPRQVLDPVRMEAAGSELDSSVELLLILFRIAQKARELGVLQRDNENQ
SIIHAQKAQVDEMRSGATLMI AMAVIAGVGALASAVVGSLGALKNGKAIS
QEKTLQKNIDGRNELIDAKMQALGKTSDEDRKIVGKVWAADQVQDSVALR
AAGRAFESRNGALQVANTVIQSFVQMANASVQVRQGESQASAREGEVNAT
IGQSQKQKVEDQMSFDAGFMKDVLQLIQQYTQSHNQAWRAAAGVV
> gi|50282917|ref|YP|052973.1| pilus subunit precursor [Rhizobium sp. NGR234]
MSKIGTLTSAVGAGAAAGQNVAAKGAGAAAFQAQIAELAAVSAEATARSM
LLRTVTTELQTTKKAADERVQ
> gi|50121145|ref|YP|050312.1| putative virulence effector protein [Erwinia carotovora subsp. atroseptica SCRI1043]
MAKLFLRSGSLDDFLALGENGQPVYASALQLRETLRLRKQQQIADCLAIP
QPNEHGDRIDWYSPVD GNVTSWIAASEEEREKALSLLETYKAAVADISQR
AQNAEKAGQKLFGVLLAKAIQFPGANHVYLVDGMPVLTFWGFVNLDKKSR
LDALDCLRPIIKEAEPLFVAPAPAASAPTLPLVDPVPEPEPQPIVQPDPV
EPTPAPATLPTRPAFFRLWWLLPAAALLAILSLQIRGCVSEQEKPTSEQA
ATVKPEKRALSSSTPAEPTPPPAPPVVEKEIVKAAEPPISAPPVVSPAPI
VEAAKPEAPAAVEPKEP VEPVVEQVPALPAGKDDLVMPADAVKIGSIKFL
NGNWRVIVDSKAPITGRPPSLRYQIQNGKGTARITHGDGVTCRANVEAGL
MGSGNLIVNSRSGARCSDNSRFQMPELVCKQGASGTAAECIGRYDADTVF
PMTIKRESK
> Q8XPN7_RALSO
MKIGRITGFALHGGDTASVSPPKSETASKGKSRAPAFAGLKPAAGTLEPT

QPSLRRTASVRMAVSDDAEPRRLERTLSCAGSLQRAPFGFDPQK YRARHG
KTLTEKYGSRQPARAKSGKKAIQPDEREKLPDGWWRTRVNPTRILVTSTG
IQTQADIEHCGLTKDAQAEPRSLDQLIDVARLRNEPRKFQYKWDMSANGT
FIIGNIHVQKPDGKGVHHLGHPTLVGGRRIPEARISGMLYADETGRLIIN
NDSGRFSEYPDRDPSQLEAVAAFLRQHGLIVEVEWIDMQNGKAKKRPPIP
LVNDPQLDW
> Q8X5W5_ECO57
MLGHISKFDGNNSLIKHGVV QGNNIVDFDLLRNFNGGPGLNRENFIYISN
IFLNIKQRNEKNHSINMFREVSISGDIVSVKFYRNEKIECACDFMMAKDA
QGYIDLSELDLTSCHFKGDVISKVSFISSNLQHVTFECKEIGDCNFTTAI
VDNVIFKCRRLHNVIFIKASGDYVDFSKNILDTVDFSQSQLTHSNFCECQ
IRNSNFDHCYLYASHFTRAEFLTDKEISFIKSNLTAVMFDHVRISTGNFK
DSVTQLMVLSIDYSDIFGNEY LDGYINNIIKMIDSLPDDPAILKSVLAVK
LVMQLKILNIVNKNFIENMKKIFSHGPYIKDPIIRSYIHPDEDNKFDNFM
RQNRFSKVNFDTQQMIDFINRFNMNKWLIDRNNNFFIQLIDQALRSTNDT
IKENAWHLYKEWIRSDDVSPLFIEIEDNLRTFNTNELTRNDNIFILFSSV
DDGPVMVVSSQRLHDMLNPTKDTNWNSTYIYKSRHEMLPVNLTPETLFGS
KSYDKHALFPIFTASWRANRIK NKGI
> Q4ZN85_PSEU2
MPSRFTHRPQLRQLIAASSLVALVACAEKPTAADATPLQSSKVQTNAPAV
APTPALVVDDNLTIQPAVSFSEWQAGFRAQALKAGIRADVFDLAFAGVTP
DMSVVKADRSQPEFSRPVWEYLDGAISAARVRKGQALLSQYADDLQKIEQ
QYGVDRQTLVAVWGMESNFGSLQGTQSVIRSLATLAYEGRRPGFAQSQLL
AALEIIQHGDITPDKMLGSWAGAMGQTQFIPT TYNTHAVDFDGDGRRDIW
NTPADALASTAHYLQSSGWQRGQPWGFEVVLGSGFDYSLADSTTRKSLVE
WQQLGLKQPDGSAIPVAASQQQAALLLPAGYRGPAFLVLDNFRAILKYNN
STSYALAISLLSDRFKGAGYVVGSWPRGDLPLSRSERIELQTLLSARQYD
AGAPDGIIGANTRKAIRSAQQSLGWPADGYPTHELLENLRKPVGQ
> A1HLP5_9FIRM
MQKLAVIADDLTGANDTGVQFAK QGLTVEVLLGSQDAAATQADIIVIDTN
SRAISSAEAYRRVAQAAAIVKASGIAAVYKKIDSTLRGNLGAEIDAIMDT
CGQAAAIVAPAYPQNGRCVIGGYHLLNNIPLEATEIARDPRCPVKESHIP
TLIAEQTRRPVGHLSFKTLLKGEDATQAALEELIGCGKEVIVCDAWEDKQ
LALIAAAAANLGKPVLWVGSAGLAQWLPTVLGLTARKQSSPVLVIAGSVS
GVTRGQVAKLKQNANVKGIEVDAR ALLAEPTRSGEVKRCCAEAAAALREG
YDVVIASGYAEEVVEHTKAQGAALGYTAQQVGDIVAGALGEIGKGLASEL
TLAGMVLTGGDIAVSVCRALGAASLSVASEVAPGIPVGFLTGGLCDGLKV
VTKAGAFGGEDALVKAVHCLKS
> Q7ACZ9_ECO57
MFPLNDLSLKTQSVQLNKITSNTESTIKQHELVSDDAIINELSSELVSCL
GNDKFTPVSEDSNLLNMLSEFKLLREQCFRWGNYTLLF ENYGAYDKTGSI
TIEKSQGEGTLPIRHKLEFISTNIAELLDKLTKITDARLCKGFSDWASSV
KEGASNDLKENVDRALVRMFKCVKLHSNELNLSSLSLGSVPPLPEWIEML
SLVYNELDSIQVPESCKELELDFNNLTEFPQVPDGITLISVNNNLISYID
SFPPKAKKIFICHNKLSEIPALPDTAKVFDCSENNIKEIRWFPKNLKEAY
IEYNKIEVIPAIPGNLKLLCMKCNPIKEAFLMPWTLTGI RYEISQRKYIV
MNPADYDKYSDMVKKHVIDGEEFIIKYYM
> gi|29171493|ref|NP|808677.1| type III effector HopO1 -1 [Pseudomonas syringae pv. tomato str. DC3000]
MGNICGTSGSNHVYSPPISPQHASGSSTPVPSASGTMLSLSHEQILSQNY
ASNIKGKYRTNPRKGPSPRLSDTLMKQALSSVITQEKKRLKSQPKSIAQD
IQPPNSMIKNALDEKDSHPFGDCFSDDEFLAIHLYTSCLYRPINHHLRYA
PKNDVAPVVEAMNSGLAKLAQYPDYQVSGQLHRGIKQKMDDGEVMSRFKP
GNTYRDDAFMSTSTRMDVTEEFTSDVTLHLQSSSAVNIGPFSKNPYEDEA
LIPPLTPFKVTGLHKQDDRWHVHLNEIAESSDE
> gi|28868090|ref|NP|790709.1| candidate type III effector Hop protein [Pseudomonas syringae pv. tomato str. DC3000]
MKINLPALRTTSSRVQICLTAVLLCTPLLFSAHAQAAGTASEQANVEVMI
RQLNALEAVAQRSVDLPQDPAQRYHLDYPRLVSDIARIRQGLQDYLSPSR
AQPRDPVELSGHYNVSGDHTP
> gi|12329070|emb|CAC05801.1| IpaD, secreted by the Mxi -Spa machinery, required for entry of bacteria into epithelial cells [Shigella flexneri]
MNITTLTNSISTSSFSPNNTNGSSTETVNSDIKTTTSSHPVSSLTMLNDT

LHNIRTTNQALKKELSQKTLTKTSLEEIALHSSQISMDVNKSAQLLDILS
RNEYPINKDARELLHSAPKEAELDGDQMISHRELWAKIANSINDINEQYL
KVYEHAVSSYTQMYQDFSAVLSSLAGWISPGGNDGNSVKLQVNSLKKALE
ELKEKYKDKPLYPANNTVSQEQANKWLTELGGTIGKVSQKNGGYVVSINM
TPIDNMLKSLDNLGGNGEVVLDNAKYQAWNAGFSAEDETMKNNLQTLVQK
YSNANSIFDNLVKVLSSTISSCTDTDKLFLHF
> gi|28868580|ref|NP|791199.1| type III effector HopPtoA1 [Pseudomonas syringae pv. tomato str. DC3000]
MHINRRVQQPPVTATDSF RTASDASLASSSVRSVSSDQQREINAIADYLT
DHVFAAHKLPPADSADGQAAVDVHNAQITALIETRASRLHFEGETPATIA
DTFAKAEKLDRLATTTSGALRATPFAMASLLQYMQPAINKGDWLPAPLKP
LTPLISGALSGAMDQVGTKMMDRATGDLHYLSASPDRLHDAMAASVKRHS
PSLARQVLDTGVAVQTYSARNAVRTVLAPALASRPAVQGAVDLGVSMAGG
LAANAGFGNRLLSVQSRDH QRGGALVLGLKDKEPKAQLSEENDWLEAYKA
IKSASYSGAALNAGKRMAGLPLDMATDAMGAVRSLVSASSLTQNGLALAG
GFAGVGKLQEMATKNITDPATKAAVSQLTNLAGSAAVFAGWTTAALTTDP
AVKKAESFIQDTVKSTASSTTGYVADQTVKLAKTVKDMGGEAITHTGASL
RNTVNNLRQRPAREADIEEGGTAASPSEIPFRPMRS
> A0WN29_9GAMM
MNSTVELAFYGDDFTGSTD VLESLSLRGKKTILLLRVPEKKDSIIYSDYD
CVGVAGISRSKDPVWMKAELPKVYQFLNSFDPKYIHYKVCSTFDSNLHKG
NLAVATQVGIDVLSPSWTSIVVGTPKIKRYVCFGELFADSKGETYRIDRH
PVMSCHPATPMTEANLVKHMHQLGEVDCASVNLSMYSDDSAATKLQQFIA
ENKVVIFDSVDHSSQAKVGALINEHCQEGLHFSVSSSGFEDAVYSQNKLV
ENINITSKSRILALSGSCSS ITSSQIDYALSHGFIPVELDIARLLMLESR
PQYLDEIVQTCIDLLSSNKSPLVYSALGPQKSGSTTASQDGDLHFDEMLG
SFLGAIALAVTKTGLIERLAVAGGDTSGYCIQSLNLDALTFIAPLCPGVP
LCRAHAQDSDIDGLELALKGGQMGSEDFFVRLCSGGE
> NOPP_RHISN
MYGRIDSSSDFHYTQSASKQMDAETQEFADTFARMHLDRSNGGSSSAARY
TLDHEPPVVPIDLETFRREIR KFHGKEITDIANNPQEYSDFVSAKARRTA
DVAQQYGIRRDSENARYFSYQLGNQCVGLMRTEGGFSMEEEFESKSWRDQ
FPGHQEITSTVDLQVAHPLVENAGDILLEHQLRRDGERPLLNWRAENPEA
KARAAMMGFVEVDDCDMVLDPKQHPDKWTQTSAAEWRRKDKPPLYLRKFE
DAETAQCSTSCSYETYEDDFM
> gi|34498035|ref|NP|902250.1| probable secreted pr otein EspA [Chromobacterium violaceum ATCC 12472]
MSTAISSISSFSNGQVDSVGKAGDGDNIDYLRAARNYSLLGQAIATMEEV
MLLFTELSNAKFAQMSKKMEVSRDAQEMANKVEAVLAGITDPKDTKKLPQ
DVLDYLKANGISVDSVENLEGDLSQADLTAVKSALESFSGRASDFVQQSQ
LKMQQLIQNFNTAVTMANSLQSMNAESTKSIAQAIR
> gi|28868613|re f|NP|791232.1| type III helper protein HrpK(Pto) [Pseudomonas syringae pv. tomato str. DC3000]
MRISSSPFVIVNQPTPGELALAVESPLAKALPTPVGGGGQAGVQFGQPAG
NTQGAPTGAEQTASSILALLLQGSGGAAANANVNNQVQPQPCADSPAEAA
PTEATAPAAPVEQVAAPTNTDATDASNAPKAADAAFLDNSEYSSPEALKR
WEPMVAHL PPEEREQAAKELNRPIAAAWMARDDGPDAGKAMDFINANPAL
KTAVDVAQGGGNADGKITNKDLKTFAKNMEKAADSADKDLANYTKDNPDA
DPQSLEMVRSAAVMRANMPLAIAADPHHAVDAADKTKVDGNVDAEDLKGL
AQSNPGLSGALKQSCSTWSQPGFLGQVDEAGMSGRKKAAHSPDKMFDAKN
LSEWIKKSAPTNGGQFASMLSDSATLNAVAGIDISKLDKDVFDKPKSYSG
AQKAAVMVK LQQTQQSVIAGRSLRNTDKTEQGLNDRISQLQADPDVQAYL
NKSIPEQERNLVRSDASLQKAVVEQTKNVNSGQALQTDMDKADKAVNKHN
PNADYSGAISGLSAQLQLQKDLFPDSKVPTTNQVLENKPDLQDKIATSYV
TNFSEGGALKQLLGQKKSDASQSLQTADNQKAIYDSVLPPDFTENQHENY
MNSTLGTLQDSKKGRKLLEGKSDEGGPSMLTQLAEAGVGGKALNSIVGFA
SVSSLIAHGD KLGATQAIYDSTRMGAEAIKGGIDAGAKMMGREASAGLGR
LGGQMIGRAVGMVAGEATGLAAGAALGAAIPVVGWAIDGALAVGFGISMI
IDAVKKHKAQKAFDHNVDPVLDQFGIAKAH
> gi|28871842|ref|NP|794461.1| type III effector HopPtoA2 [Pseudomonas syringae pv. tomato str. DC3000]
MHINQSAQQPPGVAMESFRTASDASLASS SVRSVSTTSCRDLQAITDYLK
HHVFAAHRFSVIGSPDERDAALAHNEQIDALVETRANRLYSEGETPATIA

ETFAKAEKFDRLATTASSAFENTPFAAASVLQYMQPAINKGDWLATPLKP
LTPLISGALSGAMDQVGTKMMDRARGDLHYLSTSPDKLHDAMAVSVKRHS
PALGRQVVDMGIAVQTFSALNVVRTVLAPALASRPSVQGAVDFGVSTAGG
LVANAGFGDRMLSVQSRDQLRGGAFVLGMK DKEPKAALSEETDWLDAYKA
IKSASYSGAALNAGKRMAGLPLDVATDGLKAVRSLVSATSLTKNGLALAG
GYAGVSKLQKMATKNITDSATKAAVSQLSNLVGSVGVFAGWTTAGLATDP
AVKKAESFIQDKVKSTASSTTSYVADQTVKLAKTVKDMSGEAISSTGASL
RSTVNNLRHRSAPEADIEEGGISAFSRSETPFQLRRL
> gi|58583878|ref|YP|202894.1| avirulence/vi rulence protein [Xanthomonas oryzae pv. oryzae KACC10331]
MGDVAGGVEGCQDPPKLCTAMRPRKLCRDHRPLVWRRTCKEVCLMDPIRS
RTPSPARELLPGPQPDRVQPTADRGGGAPPAGGPLDGLPARRTMSRTRLP
SPPAPSPAFSAGSFSDLLRQFDPSLLDTSLLDSMPAVGTPHTAAAPAECD
EVQSGLRAADDPPPTVRVAVTAARPPRAKPAPRRRAAQPSDASPA AQVDL
RTLGYSQQQQEKIKPKVRSTVAQHHEALVGHGFTHAHIVALSQHPAALGT
VAVTYQDIIRALPEATHEDIVGVGKQWSGARALEALLTKAGELRGPPLQL
DTGQLLKIAKRGGVTAVEAVHAWRNALTGAPLNLTPDQVVAIASNIGGKQ
ALETVQRLLPVLCQAHGLTPDQVVAIASHGGGKQALETVQRLLPVLCQDH
GLTPAQVVAIASNIGGKQALETVQRLLPVLCQDHGLTPAQVMAIAN NNGG
KQALETVQRLLPVLCQDHGLTPAQVVAIASNIGGKQALETVQRLLPVLCQ
DHGLTPTQVMAIANNNGGKQALETVQRLLPVLCQAHGLTPDQVVAIASHD
GGKQALETVQRLLPVLCQAHGLTPDQVVAIASHDGGKQALETVQRLLPVL
CQDHGLTPAQVVAIASHDGGKQALETVQRLLPVPVLCQAHGLTPDQVVAI
ASHDGGKQALETVQRLLPVLCQDHGLTPDQVVAIASNSGGKQALETV QRL
LPVLCQDHGLTPDQVVAIASNSGGKQALETVQRLLPVPVLCQAHGLTPDQ
VVAIASHDGGKQALETVQRLLPVLCQDHGLTPDQVVAIASNIGGKQALET
VQRLLPVLCQDHGLTPDQVVAIASNIGGKQALETVQRLLPVLCQDHGLTP
DQVVAIASHDGGKQALETVQRLLPVLCQDHGPDPGPGRGHRQQWRRQAGA
GDGATAVAGAVPGPWPDPGPGRGHRQPRWRQAGAGDGAAAVAGAVPGP WP
DPGPSGGHRQPRWRQAGAGDGAAAVAGVVPGPWPDPGPGGGHRQPRWRQA
GAGDGAAAVAGAVPGPWPDPGPGGGHRQQWRRQAGAEDGAAAVAGAVPGP
WPDPGPGGGHRQQWRRQAGAGEHCCPVILP
> STM1172 flgM anti -FliA factor 1257046:1257339 reverse MW:10568
MSIDRTSPLKPVSTVQTRETSDTPVQKTRQEKTSAATSASVTLSDAQAKL
MQPGVSDINMERVEALKTAIRNGELKMDTGKIADSLIREAQSYLQSK
> gi|33600606|ref|NP|888166.1| putative outer protein B [Bordetella bronchiseptica RB50]
MTVMSTTISTAPSGAALAPSRIDMRAPEPGSAGEGAGILAPVTTLALAAG
RPAFPASPSLRTAPVLDPPVRDLSPADLADLLRVLRSRAVDGQLATAREN
LQDAQVKAKQNTQAQLDKLDA WFRKAEEAESKGWLSKVFGWIGKVLAVVA
SALAVGFAAVASVATGAAATPMLLLSGMALVSAVTSLADQISQEAGGPPI
SLGGFLSGLAGRLLTALGVDQSQADQIAKIVAGLAVPVVLLIEPQMLGEM
AQGVARLAGASDATAGYIAMAMSIVAAIAVAAINAAGTAGAGSASAIKGA
WDRAAAVATQVLQGGTAVAQGGVGVSMAVDRKQADLLVADKADLAASLTK
LRAAMEREADDIKKILAQFDEA YHMIAKMISDMASTHSQVSANLGRRQAV

> A4VIM7_PSEST
MTDLKDFRASLRNRNHAFSDTLAFIASNYDYQPSRFVNGTLENAAGENEG
SCKTLGLALLEGFSTEEALLAFGEHYQAVLANPNGSDHRNIRALMETGLP
GVRFDSQPLHRK
> A0FNY1_9BURK
MAAKTIRPSSRFSPASTCRRSAEREDRDDMTTSASKPLLGCIADDFTGAT
DLANMLVRGGMRTVQTIGVPAADTRVEADA LVVALKSRTIDAADAVAQSL
AALEWLRAQGCRQFFFKYCSTFDSTDKGNIGPVADALLDALSPIGGGTPF
TIACPAFPENGRTIYRGHLFVGDVLLNESGMEHHPLTPMTDANLVRVLQR
QTRSTVGLVRYDAVARGAEAVRASIDALRHDGVRMAIADAVSDADLYTLG
EACADLPLITGGSGVALGLPANFRRAGLLGDAAADAGELPRIEGASAVLA
GSASKATNAQVAAWRETRPAFRIDPLAAARG EPVVEQALSFAREHMNARE
KQPVLIYATASPDEVKATQRELGVAQAGELVERTLAAIARGLHQMGVRKF
VVAGGETSGAVVQALDVRMLRIGKQIDPGVPATATIGAEPLALALKSGNF
GAVDFLEKALRHLDGGTQ
> gi|57434439|emb|CAI43856.1| EspF protein [Escherichia coli]
MLNGISQAVSTLGRHITSAASRVSSAGLSGFSVSPQAVRLNPIRAQSPFS

PGTSNINARTTFNVSSPATSFTPSRPAPPPPTSGQASGASRPLPPIAQAL
KDHLAAYEKSKSLEASGLKPSRPAPPPPTSGQASGASRPLPPIAQALKDH
LAAYEKSKSLEASGLKPARPAPPPPTSGQVSGSSRSLPPIAQALKDHLAA
YEKSKSLDTSSLKPSRPAPPPPTSGLVSGSSRSLPPIAQALKDHLAAYEK
SKSLDTSGLKPSRPAPPPPTSGQASEASRPLPPIAQALKDKNRY
> gi|15835055|ref|NP|296814.1| adherence factor [Chlamydia muridarum Nigg]
MPPSAISKISNSTTPQVQSSSAPNLTMLEGKGISVEKSFRVYSEEENQNH
TTEDVVRIGAMLQQQFYNMTTGRNESFTVSPAHHSGNWKTSLLYNFAQLV
AHIFSLNTVPAKIIHHENGTKQNWESKQPTVSSEKSSNKRDVSESIAVVQ
RVNPEVITKSSLKATKSIRRRKPRSVEEKREQLVKDP ADKIVYDLRADTI
ASRLKLTEEQIQKCRISLNNLKKAIQRYLNIDESERNSRKGQDLLVKQSI
FLNNILENAKMEESEEFKNVMSVIKTEFNSHRVKIDSHFHGIWIAGAPPE
GTDVYIKTFLQTYEEFDFLFWVDRQAFGAAKFSSILKKIAFDSSLKELRS
ITSQETQDFVKEYDELQAKYQTSGRREERRELEKDLRKLFDRYKHLSQEI
RTNFDALFLKNMVLSQDGFFNYCLLKGIGTIDDQTRIA YLQDELHLPEEE
IQQYKDLIKANEQKIKDIVGKVNTDLGKERVFIKNIRDLSSMQDRTNTYN
YEMEMLLRWNYPAASDQIRMYMLKELGGIYTDLDMMPQYSPDVLQMINEI
GGDRFFEELPIRRAVSDGVLRLANGESGITIDNIAQDIDISKLTRSDRTQ
LEKLLTDIENKQKSSGTSKFSLFQRMAPDSVRDFMPILQRHHKWSTGWNV
RGLNGLMMAHKGSAMVDAVIKGQRQAYRELKSLRETVLS GEFFKTLDDLK
HLNHKALIGGHLVEDFLGKSLFSEFRQDTIIAGALSTLGITGPDLIVDEM
KTYFRSLGPIGKDYLEGKKLGNKAFLGSYNKIVKEGKQTFDWLHPVTVGA
NDVTPADESTWCGIKQRCLAELLFSDESKFRPETPKGISRTKIDKKTFTK
LWSEQSKRALSPELLDRFNELIEAKNFDLIKFTDVDRALYVLRNQVCDDA
SASAAVFSLQLQLAELLRGTSFPIANQLNVFPDLNRNIAS SLEKTIKLYL
QSHSQTETVIWHSAISDRVMFLRDMLATAERMRLVKESIFSKEETPLTTE
EIKLLNSWAELQSKDNLGILSLEEVDKLLDVTSSIMENSKLKKRVSAIED
SIVAGYFYLRLEETLSEWVRIPPKDLKKRVIRFMEGMDANQQHPDERGGR
WYSQLYDESFKKRVTAPVKKIQELAKKYLNEQRVHVLEIDDYLTKNPLFN
RLHEEGYAFSDLTEITRYMLAEYGISGIFSEGNILPSPSAR LVNIIKTYV
GGDYHDMQDVLPKIYDWLASGGAADLTNERFAAIPESLRKNLEGLHGTDL
LTPPVDASVSGWGMSFGVENGVESDHTMISIAPGFFNGASYSMQHYLSAL
YEIHRHIHLGSLTSDLIKKELESKGAGCFVHEERFDSLLKASSEKQYLSL
TEIHKSLSNQVHLAEAVSHLMNTALPGVGKIIEREGDFGRLGITTTETSV
AMRSYDYRGIGVSKDLFSLPQEVPTIQNVVEQAKYTLLSWPE FYNRYANA
WSDLATHYGAEILEAHPQSFLYEVEGRCMGLSLLYMSIEDEGGYRTLQGN
LDTVSALYQQKERDHLPLSSKDQSLLSRDLSLINWLQYQGNKVLLENRGF
NHAKWDVLQLTSTFEKSLTKSLLITTPTHSLTLNFMGSFFRVTDPNFGHV
DFPSLAAALYFIEDMVQVSPHIKQRYGFSETLSVREQLQVYFLDAPEGIQ
SLLSFTDAGLLSSHQATTLEKMRERGPVIIERIQTTWGKLYEI GGSIHHR
RIDEFTSETDLDEMKLNGDVLTDFLTKHVLDSETVSLILVLLETRGLEEG
TKHVSRSLIVETPDEAASLFQFLKNKTAKTREMLVSLLSEVTDKLKSAQL
DGEDVEVSRVSFSDNSDEITVEVKKGSLLKKLSIRGGGIFDSFKQFSAKA
NELGATGVMDLELGMSIVSIVQYMRLLEEGKGKDPLAVANLVMDVKETAE
MTVGAVIQGLSKKFITQEGVDGFRLETAIASQLKKASSRVGGSV GKVFSK
TAAVLELPVLETALGAWNLYDSVVSLKEAQTHSDRMAARVDIAFNSITLG
ITIASVALPSLMLAVGPLAAIGMGASAIARNVALKEERYDQWLVYKKFLT
EGGSHILFADPDRHLLDFSGNYVLGNFLLDLRQNPPILKGDRSYNSNWRI
GNKAGWTDWQIRDRIGYGYRISPTSALARGHANSFWPPELPTIPKGDYRT
VILGYGTTYEAVTEVVYLSNKVVWREAVMESSSRYYRPPLTAKSK LSTII
GGDSPLTVIPVRLIEKDEKENIDHAASYKDYKINIKGGKGGLTVQIGGAG
FYNLTGTLPATNTISFRAIPAPFGVKFNLSLVEQDVPLIRPNGTEEKFLK
IRQTGFNTILGSSEGNDTLTGGNDTKFYSSPGGGTIYSGKGKVEYHIPQL
THPLHIILSKGSSAHQLVTEINADQVKPVLGSLSLTSSMELISKQEIHVS
SEDTKEDLGVWKERYEVRLGDGITATPIDQMNAGTRLVTMGVKTCD VAKW
QKQHPTETSYPEDILDWLTQKSWFLAPEVLFVLENGTAKFITNRKHIVYS
PKPYSELAVTQSRDIKAFVQGNTGCSYVFSTIYRSSQKTQQTELTLVDDD
VYAQTIDVSSILPTLILAKKSGDNQIDFEFSSSHHLMAIRVSWKGVIPER
TRVKINNETILRLGWLNAKLMASRNPSYWNLLYYNRNVIPERVEDVKSLN
NTVILMPPNTKRNGEHVFAIENQEGVDVKVLGFLQSGHVLGEYEEDL MYK
DVFARKITHFAVTVPAHKTKYLFFTDEESLANPEDNVLFYSKLGSNTLQA
SQKPLTQFARQLWASYDEISVSDTSLKLENFVRYLMEAETIELYRQLLYA
QNLLRIHNRDLVLKLFYVRQSEGMGAVLITFKNFFIESMEGISAPTLERE

AKPILASDPMGLINPAYREHLDLFLGEDKLNLAILVQEFSMLQYIVSMQE
GKALGSLLTPPRYKDMNLAVLTYTVASSWEGRREASWLKAPIEYKLPL ET
TLTASYYLXPVSGDLYVTCLMNRRPQNQAFVIRFKGYKFQKGAFRSIALI
SSHSVGESRIAKTVLQFRGQELFHLEKSAPNKSAVLQLDSKLLSLSRMLF
YGGDQVIRYTPLSVQQFLSLDDLWLADLKERVMNANEEGFKLISQMHDSY
LMESCMNLRDAVPQWKINPNVINNAITYYRVVVPFWIRNRLTKGSLIRIP
AHSIPIALTTTQNDVFKTIYAPGFSIYFSFLGLDAFLKPRTHNRVGDML L
ELEKEVVVTVKKVDETDYNKKRVYIVTELTKEEERVLKANKNVITIPMKS
SYTKK
> A0RRK8_CAMFF
MKISSNIFILSFALFITLLNYKFFEFAINKAGFYENKIVILTLPVLFFAL
LIIAFSLLFLPYLTKPLSIFIGISGIAGAYFMNTYGTIIDSDMIRNAVQT
DVKEVKDLLNWDIILWILGAVFVVIIVVKTKIIYSKVLYEIKIRSIFIVA
ALAIFGASFGIFSKNFIPFFRNYPEIRFF TTPFYPIYSIIKFTKSLTPKA
EFQKIGLDATLKDDKKRLFVLVVGETARAANYSLNNYMKNDTNPYSKENG
VLSFTNFYSCGTSTAISVPCMFSNLTKKTFSPSKANNTGNLLDVFTVANI
DVSWFGNNSGWCKGVCDRLQNAKNYGGEGFDEVMLKDINSKIQNASKNSF
IVVHLQGSHGPTYFKRYPKEFDKFNPTCDTAVLKDCTHDEIVNTYDNTIL
YTDYVMNKIINMLKDSKFETGLLYVSDHGE SLGENGLYLHGMPYAFAPDF
QIHVPAILWLNDNKKLDELSRLKDENLSQDYVFHSMLGFFSIDSNVYDNN
LDLFAKDIK
> gi|34498072|ref|NP|902287.1| pathogenicity island 1 effector protein
[Chromobacterium violaceum ATCC 12472]
MYGIQTSASLPLTRPQALETQAAAPQSDAPDASAGQTRAAPQGSAAPALA
AARAKADELGQAA REVRASVERQTAYETRLAAQRSAAAFSGGEPPQARRE
APGAELDEARNAQTVSARLFEGNLKGVAQSGHAMSAEQKQALQSGLDDVF
ADAPPQARSAGAPMLYSANAAAGQGMADSDLWDMISDQIGKIKDNYLGVY
ENVVGQYTDFYKAFSDILSQMANWIKPGGDGNKVKLNVDALKAALEKLKK
DFSLGDNLDNKKAVLFPAQSKDGGIQGGSESDARKWAKEMGLPDAPPPGF
SCVQKAADGNWVVV VDMTPIDTMIRDVGALGSGTELELDNAKFQAWQSGF
KAQEENLKNTLQTLTQKYSNANSLFDNLVKVLSSTISSCLETAKSFLQI
> gi|53722546|ref|YP|111531.1| putative G -nucleotide exchange factor
[Burkholderia pseudomallei K96243]
MTYNPRIGGFTHVKQASFDVHVKRGEAQPRTSFAQQIKRIFSKIGETLGQ
LFRHRAPDSAPGRV RLQGVRYVGSYRPTGDAKQAIRHFVDEAVKQVAHAR
TPEIRQDAEFGRQVYEATLCAIFSEAKDRFCMDPATRAGNVRPAFIEALG
DAARATGLPGADKQGVFTPSGAGTNPLYTEIRLRADTLMGAELAARPEYR
ELQPYARQQAIDLVANALPAERSNTLVEFRQTVQTLEATYRRAAQDASRD
EKGATNAADGA
> A1JU90_YERE8
MSRIIALIISFLLVGCATPPMPAQRIVGEVRMSRPLSRT AHIDVSIFGLY
EGKVREVQRTRFETGNLPLFFSIKLNPAQRGEGELYLRSTLSFPERGVQA
VAQQKLIGKNKVVLQMIPKTCYPNCQSPNTR
> gi|53722545|ref|YP|111530.1| putative intercellular spread protein
[Burkholderia pseudomallei K96243]
MINVDAFVASARSGARVVVGGDARGPVVSAARLGMKERLFAFLAHVPLLK
HCDAVRRYAEQVRMENRRSLEVFVLALSKRYGPEGAKAAFDYGARRDGAP
LDQRRVRNMVSIAEHFHGTGDAKPLARQMVFRSWECRGLDHPGHASLTIK
NQADADAGRHVYEHVSWWPNQRLGSKEHFDRIEPKTLDGYRIDKRSEISS
ATEQRLREGDAARRKILADGFKYANQDERHDARFFPRAGQKLDKDAEWGL
SARKVYFPAIGFNHDRRDTDRPRAFVLFGLNEAAMLRDARTVKEGAKSGE
LMYQMISKKENCASMALRVLRAGGAEHFVPYTAAWISEDPNHAHAYALAV
QARIDALNQRRADVERRCERLRDSASVRQAWRAFSEAGGASASPLAEDAG
RGRASAHMRQARLDEHAREVERIGAYFAELSAGRSGKHRDRADAALADAM
KRCAPSARDDVAALTRKASVLVETLGRHLDAPPPSDSSALRRLAAHAMIG
RIEAFMAAAIAA
> Q3BYL8|Q3BYL8_XANC5 Xanthomonas outer protei n F1 - Xanthomonas campestris
pv. vesicatoria (strain 85 -10).
MKLSSDIGTAASRGAASHPPVQPTQAEDVAAPREERAPTGPLAGLASSSA
ALRGRRASLAGRASPHADEEGAMLGGSHRSDSSQSSQASDATFYTAQVVS
PAREIDTPDVPAAAATYAERSTAAAAEVKAQLRARLDALPFAAPSEEQTA
LQASYLEWADARVQERINAFGPDAGYQAVGDMKTAGTKAAL PIAYECLRS
FIIGAMRIPVGLATGAAYASRRPADAEELSTAMVAGVAAGSASYTSDTLL

IPAMDRRAPVSNLPRFQAIDPKILVPDPPPVILEITAEGKRFTRPGENNA
PTLADLKAQTYDRRLGITQRQSTLDDKSLDTLLLKPVINAGFNAARRTGG
EPGLLTPVGQWGLSALAIGGAGVVQKALLETGKAVARTGQMRVPDLVGGE
QRLNLFALALPDKARRPAQWSDAVHFPPTYLLDTGKEALALA RQGFNSAN
AVATTARDLLTRHMLSNVMASFGPMGAGRIITAPLRRGSHIRVAGEAVNS
TAVVVQQAVQTLFNDTFWNALKAKNGANTSQAARLDNERAALAAEHQRTI
ERTLEALAEPVDQAIARLSGPTPAEAERAMEEGMRAAPGPGPLGQTLRTA
LQTLRTEIGTQSVSIATIDAVRTALREGWSQTFSGVPRDAATQALDGQLQ
TLKRALHDSEALRQWQSGRA
> gi|12054313|emb|CAC2 0124.1| harpin protein [Erwinia chrysanthemi]
MQITIKAHIGGDLGVSGLGLGAQGLKGLNSATSSLGSSLDKLSGTIDKLT
SALTSMMFGGALSQGMGSGLARGAGNQLGASLNTFGSGAQGAGNVLSKPQ
SGSDALSKMFDKALDDLLGHDTVTKLTNQSNQLANSLLNASQMTQANMNA
FGSGLNDALSSILGNGLGQAMGGFSPLSLGAGGLQGLNGAGAFSQLGNAI
GMGVGQNAALNALSNVSTHVDGNNRNFVDKEDRGLAKEIGQFMDQYPEIF
GKPQYQKDGWSSAKTDDKSWAKALSKPDDDGMTGASMDKFRQALGMIKSA
VAGDTGNTNLNLRGAGGASLGIDAAVVGDKIANMSLGKLAANAV
> VP1698 VP1698 hypothetical protein 1808583:1809581 reverse MW:38503
MRRRTQMKKQHWRRRSLFPDSIVTQRKVTVLQRGARYESASQPLQ DLNVV
HVNHRQLLSEGVLNDDQLSLLQRLLDRSVVDSLCASQLVKTYLRLGTSID
RFAMRLFLEIGAQLSDSQRVATFEQRLEYINSRLGFRFNLATPKTLILCC
YLALTEWIHRQTDQSALHASVKVEQLMNQLDIQKEYWSKLSGEDTSAIFV
EQQLALIESQQTQLKAQLNTLNEQQSQVIESHKALVDKWQPSLSNLKELA
DYTSTTDMFISDWKTWCSEARLQAPDLNEVWDACDVVYNDLNAVAK VWQW
FKDMQIVGDVDHYYFDIQSGQCGQACNHLSQI
> gi|29840317|ref|NP|829423.1| putative cytotoxin [Chlamydophila caviae GPIC]
MTLPEKTTSVASSAQTHSNYTAASNSSSLRQNNTTRAASLDERLALYDEA
VNQNNSTETVVEIGKKLQEEFYNLTTSQPTRTTASPSNHTGNWKTSFLYN
LAQLIAHIVPPQILPAKIAKPTIIPPAANATGNSTTEI SQRASNASISQN
TTSLSQPVIGGGGRPTAKKKVLGTLVKKSASYRYKKTLKTTAKRAPRHIS
KSSSVIASSAALEPSFIEKTENQLIEKTVSADSVQNVEDSPSFSRRKRAL
EEEAQEAGAEKYDLTPENIVDKLGLTEAQKKTCRASINNIKKAIGYYNNL
AEKNSRKGQDLLVKQSVFLETIQKKAGLPETHASAKVMTTIKTEFLSHRV
AVNKHLHGIWIAGSPPDDVSPYIKVFLQTYEDFDFFFWV DESAYGAAKFS
STLKRIAFDASITEIKEKTPEAAKDFIQNYEELKKKYDSAEDAEQKEQYQ
KDLHDFSEKYDKLNKEVKENFNALFLKNMITSQDGFFNFCMLKGTSTVTD
ALRIEYLEQNLKLPQEEIEQYKKTIEDNKKKIQDIVAKANKGLGSERVKI
KDIKELTSMKDKTNLYNYEMEMFLRWNYAAASDQIRMYMLKEYGGIYTDL
DIMPSYSQEATDAIFSKGGNRFFENLQIRRVISTAALKIA TEETPVTLEE
ISKEIKTSQLTREDKKKLKELIPELEKLHKDEGGGGKGKSKKGLFQTMAP
DTVRDTMPVLRRYHKWSTGWNVRILNGLMMAHKDSAAVDAVIKGQQRAYA
ELKDLRENVLSGTFFNTLDDLTHLDQKDRVGHYLVKDYLGKSLFYNFRQD
SIIPGAVSTLGITGPDLIKKELVKYFKDFGPMGKEFLNENGRNLGDSAYL
GSYKQIKDEHGETTYDWKNPLSVGANDVTPGDESSWCNVRQ KCAGELLFS
DSSKLRVETPKGIERTKVNEADFTKLWKEESRKKLPNRLLARFNELIDNP
NIDVAKLSDLDYDILKAKLAITNDPAATTSLFSLQLQLANLIRGVQLPVG
NQMNFFPDLYKNVEDDLEKAIKLYLKSHSQTTITLWYSSSGNLSLFLRDM
LSVAERQLAVGNLIDSVGQAPLSEKQINLLTTYVELKSKEILSSFFSSDED
LTNFLETLNSITEDAQLRPQIEKIEEKISSGHFYQDLEELIN KCLGLPEH
KRRKLILDTMKDITRFSGLDKQEQQSAQKWYEKIYDTTLEKRVLNPQKKL
EALLKKFESSERVRLENLDLFLRDRSLFSRMHRDGYAFADSNDLYRFMVA
EAGISGIFSVDSVLPPPSKHLVNMMKTTLNADYEDMHDTLGAVYNYLALD
PETEEAKAALDKIPEGLREKLQNSHIPDLLIPPIDSHVSALGMQYGVEEG
GESERIMASIMPGEFNPTSYIMSSYLDTLYQLHQRIHDGSLTL ESAKKLL
NENHIYCFVEDTRLQELVNLAQEKKYLSLTEINKLLSKKKNFAQAASALL
DGVLPGANQILQRDADFGRPLATTMLEPTAMNPYDYRGAGLSKDLFSTPP
DVPTLPNVVERAKYSLMAWPDFSREHIHPWSVLARTFGSNIVHVHPQTFL
YDLEGRCMGLAMLYMSAKTPVEYAFLTQNLMTVSSLFQTKERDHLPLSEA
DNKFLLKSQSYIDWLQYRGNKEIKTQGILTPHTWDIAALSEKFS KSTGSK
SLLVTTPTHSLVVQHLDWGYRFTDPNFGHCDFATLQQALSFLESSVQLSE
EVKLRYGISEHKDIKEQLKVYTLDSQKAENSWFPSTNLKFLSYDQHMITL
DKMILRGDVYVGRKKISWRHLYAAGATIDHKRIDEKTRETDLDKLQINGE

ILSDFLSRNTLDAPLASTIQYLLDTYGTEPGTKEVSRSLIVETPRDFASI
IGGFKTKAHQMSAMLRNVMDSIRSSIKDIPPGNQDRVSVTDIEVD GEDRI
SFKFKCGSSPEKKIQIPSHGLVKVFRQLGGMLNDLAGTGVMDMELGMSAV
SIIQYARLVEAGKGGSALAIFNLSLDVKEMAEMTLGAIVQGVGKKFITEK
GIDGLRLESVLSKQLIKVSNKVGGTVGKALFRVGQMLELPILETVAGVWG
LYNSVEELMQASSHSDMMGARVQVAFDVISLALTLSAVVAPAAMLAAGPI
AAIGMGASSIARNVAKTEGRHKEWGKYKKFLEDGSEHVVTAFPERG LIDL
SGNQILGNIYLDLTHNPPIFKGERSYNANRWIGHKPHLSDHQIREKLGYA
FRITPTYALALGHANSYWPPEVPHIPAGTYNTVILGYGITYKGTTEVVYL
SNKIVWREAIMDPESRYFVSPLTAQNHRSTVITGKTHTTIVPVRLLDDDS
KERLDQALEYKNYEIRIQGGSGGVSVQIGGAGYYNLTGAPGAENVISFRA
IPPPLAVQFNLSQGLQDVPLIRPNGTKINMLKIKQKGFSTIIGSSGG QDT
LTGHRDTKFYVSPGGGKVYSGAGTNWYYIPKLNENLTIVLATNSTDHSLH
LNMHSYELHSVENNLNLLGLNGDKHKGIYIENADKSSLFDRWIGHFIVKF
SDGITVEAVERPIPGNHTNATTLGFTKCDQSTWALKHPEEPGFVDNIVKW
MKKYLWWFAPEVSITQQHGHVSYFDQEKLFVYKPDKHSELEIRAQEEFLT
TVQGSVGSSYILSSAPNKQAKRIDIVLAKDEDAPQYLDLSSLIPSLVK GR
ITNGSNSSIDLEISSPRYTIPLSLQWDPEALPEKTVIEVHPQNRPTLGEW
YKILKKDPTQWHTLYRYSVLIPERLEGILSMNNTATLMLSESRKNEEHIL
GVENKGDVNLKIWGTLWAGHIKGAMRHLHWKTFNNYRALEKFGITIDAHG
FQYLAFEGTEKSGDNILFHSVLESYPFKAKQTPQTELSHHKWKFYDEIQV
FATTLHLQDFNRYHIASENKALSRQLLYAKNLVSISNRDFIVKLFYVRE G
FGIGAMRLVFKNFFNEHMEDITEKTLEREAKPWMASNPHAFIDPSYQNHL
ELILGKERLNLATLVREFCASSHILPLSEDEDHRLILPERYQPANLAVLT
YTIDSNKIHNGPANTLRFFDNTMKEYRLPLTTILKSSYYLDPVTGDLYIT
RLVAEQTDIQHQAFVIKLKGFKRHWESYKHIFISGAHVGLTRSKGTALTF
IGPELRHLEIDFPRNTTHHVFPERLVSRSGLVFPTNDQVVNYDPRIDKQF
HTILDYMLWNLRDRAWGSKRAKAYDSYLLESAMHIYDKNPQWKIPESMLH
YAIGYYKVQVPRWVRSHMRPYTLVKMLKGSITLSLITTQNEIFAHKRGSG
FNIYFALLGLNKHVEPHSNKPGDMTLRLKQDVILKVRKVDESEYQKKRVY
VVAEIATEEDRSLRPNSQVLIFPGGEKIRYRRSLKNPKESNSTITD
> gi|12329086|emb|CAC05817.1| MxiL, secreted by and put ative component of the Mxi-Spa secretion machinery [Shigella flexneri]
MINQINASNALQQRLNSEEFVNLNERLSSSQSFDEDIIYEIMQYFSQSEL
NSIDNDELHNKIEQLFNSRFPYLTAAQKSSLLNKLIDANQYVDLHEGFYA
SLSIYNNIDFYIKTTTFDSLISVFEAGREADDSTW
> gi|15618211|ref|NP|224496.1| Inclusion Membra ne Protein B [Chlamydophila pneumoniae CWL029]
MSAPIPTPQELSDQITCLNVQYQQVSELARENKGDIEGLKTLTAALTADA
GIQPSADEIYSLQTAAALILSASEKPGSGPSGSTEGSVTVQSPCKFKKVL
AVVLTIIALIAIAVLIACIIAACGGFPLLLSALNLYTIGACVSLPIIAST
SVALICLCTFVANSLIKPVITVRTTR
> gi|15835057|ref|NP|296816.1 | adherence factor [Chlamydia muridarum Nigg]
MEQALCTMTLSNTASSTVSPKSTQLNHTLSNQTHPVAQSNRNIDFQIYDE
QIHNNHSTEDVVAIGRRIQQEYANLTTSKQVNFSASPSNHTGNWKISLLY
NLSMLVANLFPTTIQPVQPQKVIFKNQDSSKETQLTKSSKVVSSRSSLKE
IFSKKSPPLRKSFSSSKISRAIPRRKKRSNDDPVQATNTYDLTAENILEK
LSLTQEQQIKHDNLISNLKEAINRYSDLNRKNSRKGQSLLVRQAKILDEI
LSQTKSTEERASNSVMTTIKKEFTSHRVPVEKNIHGIWIAGSPPEGTDEY
IKLFLHTYPEFSFLFWVDKTAYGAAKFSSTLKRIAFDAAVNSLREATPEP
VKQFVQRYDKLKKSYDTSRDFDEKQRLSEQLVELYDNYNKFSKEIQSNFD
VLLLHEMITIQDSFFNYCQLKGVGAITDETRIEYLEKVLKVKEEDLSHYK
ETIKRNKESIEKLVKEINDSTGRERVVIKDIRDLKSLQDLTNSYNYETEM
LLRWNYAAATDQLRMYMLKEYGGIYTDLDIMPQYSQDVLQKIMDVGGSRF
FEHDKLRRTLSFAALKLGSGKQTTVSFEEAKKAMTLPTFTLQDKSQISEI
FKYLETETQAKKSLFQPMDVTVVRDFMPILQRYHKWQTGWNVRGLNGLMM
AHKDSAVVDAVIARQRAAYDEMRALRQNVVSGEFFRSLGDLEHVNREKNI
GGYLAKNY LGGSLFFDFRQDSVIPGAISTLGISGPDIIMDTMSDYFTNLG
PVGEDFLYEGKLGKAAFLGAYQAQKTPKGELTYDWLHPLSIGANDVTPAD
ASTWCETRQHCAAELLLSDSISSDEHPKGIRRERVNPNDFSKLWSKEAQG
ILSSDFADLLPRFNLLIESSALDIHTLSALDRDIQHLFTKVQKDPVASVA
VFSLQLQLAEMIRAIPFPIRNQVHILPEAQAHFEADWKKAIQLYLHSHPQ
TEVVIWYSS THTQIVFGKDLLAVAERVAAAKSLMSDHDPSLITSYLKYKT

QSHLGVLTEFDQEDFFELMVDIAEEPELHKQLLKIEEQVNSGLYSHVEHS
LGEWLKLSKEERKSKFLKILKETFQEEEREDSQQQHKTWFEELYEKRHQE
RVKDPAKKIQELITVFQESQRVQAQDIDTYFAHKPFYQDLMKDGYAFEDI
SVITKYLLASDGVSGIITTDPIFPPPSKQLIDAMKQSLGEDFGELHYTLQ
MVYDWLSKETNSVTSEQAKQKLPQKLHEKLEGYTTHDLLIPPIDGSVSAL
GLRFSTEEGKVSDRVLTSIAPGVPNSASYAMTSYLYGLFLITKDIQSGRL
THEILKERLQTYGGAYFINESKIDVLLALSRKKAQISLIDAHKALTGFSS
FSEASLALLTGRMPGTSRVLSREVEFGRPSAIVMEGATAIRAQSYDAVGL
RKDFFLLPHTVPSIQSIVEQAKYTVLSWPEFYENHADKWNDLANRFGAED
LSVHPQTFLYDTEGRCMGLALLYMLADDSVSYRLLQQNLMTLASLFDEQN
RRNIPLTPADQKFLNKGLSLIEWLQFKGNQQLQTEGFFHTLDWDIPQLMK
HFASSTVKSWLITTPAHSLVLSLMGNFFRVTDPNYGHTDFPSLEAAITFL
ERMVQVSPAVLERYGFDKEKSVTSQLKVHSLETSELQNAVFASSDLGFTS
RYFTTTLEEMTVRGPITMNQRHTDWATLYKIGGTVQGKRIDSRTRESDLN
FLKINGDILEEFLTRTVLDSDLVELIQSLLKTHGLEPGTTLISPSSIVET
AIDHVSLLQAVKTKTSRMHTILQSLGERIFKLFKNSGVQDSDKISIDRVQ
LVDESDSATIDFTVIKDKQRSQKKSITVGIESLAGSFRKFSASMHEVIGT
GVLDLDLGMTVVSLVQYVRLVEAGQGKDALAVANLVMNLKIALEVSIGNV
IQALGKKLLTQEGLNTFRLETELARQLHKVGARVGGTVGKTLTRVAHVLE
LPILESAIGTWNLYNSVNELLHADSWSDQVAARVQVAFDSISLGITIASV
VSPALMMAAGPIAAIGMGAASIARNVARKEERHSQWLKYKSFLDNGSKHT
VAAFPHKGLIDLSENLVLGNIVLNLRVYPPLLTGDRSYNANRWFGNKPGW
SDWQVRERLGYAYRSSPSYALARGHANSFWPLSMPSIDKGVYRTVILGYG
IQYKAVTEVVYLSNEMVWREAVMQFDSRYYVEPLTAEKECATVLAGDTPL
SVIPVRLLEEESLEREKNAASYKNYQIIIEGGKGGLTVQIGGAGYYKLTA
QPGKGNILSFRAIPGYLSVTFDLSRLEQEVPLTKQNGTALKILKVRQTGF
DTIVGSSGGSDNLTGNHNTKFYLSTGGGHVTSGSGKNWYNIPSLTRSLGF
DFTSNAIEHKASVEMLLADFSPANNLSLIVERFANVSIHVFNYSNKTAPY
TVNLKDGVSLQASKQGFSGSLLEVASFDQTLWQKSYPEDRGFVEDILSWL
LKLQWSLASRVRIFLQGGTAQYETTQKSLIYRPDPHSSISIQATDSYNTQ
VYGNVGCSYILFSSPGVTAKTLDILLFEDSGLPQIIDLSTLVPTSIQGFL
YPGGFINFQVSSARYAFPFSVSWRRHHYTFPAQTIIQVLPRLRFELGDWF
TILQKSVGKWVTLYQHDMIIPERIEGVLSLNNTATLMSHHNQTTHMLGVE
NRGDLSLKVLGILQSGQIKGSKSENSASGHFSKLLSVPAHTIKNLSFKEE
EGTKSKNILFYSVLEETFLKATDKPLTIIKRDKWSLYDEIQVFATTLNLQ
NFLRYHISEETPMLSRLLMYAQKRVSIQNRDLALKFFYVRERSGIGAIRL
VFKNFFEESLQGILHGTLEREVKPMLAENPNTLIHSSYRNHLELILGEET
LDLAIIVQEFSSSKNIVELQKDLATHRLIYPQGKEALSLAIYTHTFSKES
LVTSKQRSEGLKFIDPSMQEYRLPETTILENSYYLDPSTGDLYLTKIIAQ
PSQSRAFLIKFKKYKRHWLDFQKLVLSGSHLELIASTGTALTFVGPELRH
LEIDFPGALNGTIAKEHISSRASIVIPTNNQVTHYDPRIAKQHYSSINYM
LWDLRDRATLSKRAKTLDSYLLEVCMNLDSSNPTWNIPNDVLNYAVGYYR
TILPSWVKNKIRVGSLIKVEADTAKTLSFSLITTQNDLFQPVVDKGFYIY
YSVSKLANHVKLRNVTGDMLLDIDKETTFIVRGVDESDYDKKQIYVVLDL
TTEEERKLRTDKNIIIIPGGERLNR
> Q8X5G6_ECO57
MLPTTNISVNSGVISFESPVDSPSNEDVEVALEKWCAEGEFSENRHEVAS
KILDVISTNGETLSISEPITTLPDLLPGSLKELVLNGCTELKSINCLPPN
LSSLSMVGCSSLEVINCSIPENVINLSLCHCSSLKHIEGSFPEALRNSVY
LNGCNSLNESQCQFLAYDVSQGRACLSKAELTADLIWLSANRTGEESAEE
LNYSGCDLSGLSLVGLNLSSVNFSGAVLDDTDLRMSDLSQAVLENCSFKN
SILNECNFCYANLSNCIIRALFENSNFSNSNLKNASFKGSSYIQYPPILN
EADLTGAIIIPGMVLSGAILGDVKELFSEKSNTINLGGCYIDLSDIQENI
LSVLDNYTKSNKSILLTMNTSDDKYNHDKVRAAEELIKKISLDELAAFRP
YVKMSLADSFSIHPYLNNANIQQWLEPICDDFFDTIMSWFNNSIMMYMEN
GSLLQAGMYFERHPGAMVSYNSSFIQIVMNGSRRDGMQERFRELYEVYLK
NEKVYPVTQQSDFGLCDGSGKPDWDDDSDLAYNWVLLSSQDDGMAMMCSL
SHMVDMLSPNTSTNWMSFFLYKDGEVQNTFGYSLSNLFSESFPIFSIPYH
KAFSQNFVSGILDILISDNELKERFIEALNSNKSDYKMIADDQQRKLACV
WNPFLDGWELNAQHVDMIMGSHVLKDMPLRKQAEILFCLGGVFCKYSSSD
MFGTEYDSPEILRRYANGLIEQAYKTDPQVFGSVYYYNDILDRLQGRNNV
FTCTAVLTDMLTEHAKESFPEIFSLYYPVAWR
> hopAL1 Pma ES4326|Genbank: AF458049

MDVRSAPSNHATAVPTAQPSAPSIKSTLSKLFRAQCSDPVPLSSSSSTER
IAPPRVTANYSAVVDISKTASHSKTNADTAEKNSFSWLKNKLSTVSENQQ
KKQAIQNFIAQVSQQYNVEEDVAKLAVAASGAYRQLRFEETQQQRVHACL
QDTEALTAMIDL ATRKPLTYKMVMGLDSSDCSFDPIARISDHDILTEAEQ
CQVVRSAFASADKVCVVTSYGIKPLGAPKSDALSPTIASVLQGFASKQHD
KDFTGVFFYQKSDAIQNYYTEDKRTNISIKENDWAKAVTLFNAAVERGEI
EGPKEGLK
> PA1698 popN outer membrane protein PopN 1847227:1848093 forward MW:31319
MDILQSSSAAPLAPREAANAPAQQAGGSFQG ERVHYVSVSQSLADAAEEL
TFAFSERAEKSLAKRRLSDAHARLSEVQAMLQEYWKRIPDLESQQKLEAL
IAHLGSGQLSSLAQLSAYLEGFSSEISQRFLALSRARDVLAGRPEARAML
ALVDQALLRMADEQGLEIELGLRIEPLAAEASAAGVGDIQALRDTYRDAV
LDYRGLSAAWQDIQARFAATPLERVVAFLQKALSADLDSQSSRLDPVKLE
RVMSDMHKLRVLGGLAEQVGALWQVLVTGERG HGIRAF
> Q8XQF0_RALSO
MLRTSLDRASGVASVPTHPQEATGSTPAPAHTPSGRAPSAELSALRTLSS
QSSRTESGRLSIGSQRQPASAQESAIEMASHHSPTSPASPRALLSGQSER
SNSDVSQWFSAAASIRAQSIGSIESNSPPGGSPPHTADPGQTGRGIDIPH
WMPIADQLRDIFRPHFDGTNDAQFEILIQQRAERLQAMGETPATVAAVLA
KGANRDRLAQTTVGFVRSVPFGIASRLFDV KQALTAFAKTTARVGATVGA
GSGTADAFGGTLLGKATSNTQWLAASPDHLEPVMAQAHKAVQPSLGRLAA
EVSLAFQTYSLRNVIRTGVAPLATHALGAARAANVDSWIAAVGGPVAGAA
AYMAMQHMNETHHRTGAEYLLGRTDWEDQFKQLKQSTWTDPLVGAGKRTA
KLLVADLATETLAATRSLFTATNLIKNMGALAGGFAGVLTAQTAAGKAAT
EAGYTEAAVAAVRRAVSTVLSAPVYAAWTTA DVMAGPAIDAAAGHIQQAF
TPLMPHAAPPAHTAIDMV
> hopG1 Pph race 6-1448A|Genbank: AAZ34904
MQIRNSHLYSASRLVQNTFNASSTKEAANTIAKNNESAALSATQTARTHE
GDSKGKSSNGSRSHLRSMRYAAYLAGSAYLYDKTVNDFFLSTTSLNDGKQ
GFTSDARLQKAEKKAEANYHAYHNRANFGGVAPTRSSLLPKLCGENLFVT
MLDFRAATKVHMKNLIDTKEA HDSIANNISCIMGERVKPELLKKHGIVQT
PPDFDITKGASYDCKNKYSLSGVPNQETGSYGYASRSVSNPFIEKGENHH
ARAINSERALTPRNCVDALQPLLQKSERLSPEAQFRAGQALLILRPLYCS
SENWGDAHKVLMPFLEKKGLVSSQENQKLGATRPFTQTDMEKGIARRNTS
LAGPTLNKLNIFLQKSIYNKDEKAISDLTAKKLGEMKYIPIAHFKMNEDV
SSFEDSSGLADSFTGYNVSAYI NHARLLSGEDRLSKKDVIAIVGCLNAIY
DNASSERHTLREIAHGCFVGAGYTVEDAEDFYNDACKAAATEFYGGRAIN
VQSQLYSSSVDKPDS
> NleH_Crod
MLSPAPVNLGCSWNSLTRNLTSPDNAVLSSVRDAAACSDNGTQVKVGNRI
YRVVVTDNKFCVARENHSGCFTNMLHRLGWPKGEITRKIEAMLNTSPVNL
AMERGSVHLKRPDLPPVDYMQPELPRVDYNKSPVPGNVIGKGGNAV VYED
MDDTTKVLKMFTTSQNPEEVTNEVSCFNQYYGSGSAEKIYDANGDIIGIR
MNKINGESLFNIQSLPTQAEQAIYDMFDRLEQKRILFVDTTETNVLYDRV
RNEFNPIDISSYNISDVSWREGQIMQSYHGGKQDLIRVVLSRI
> Q7DB50_ECO57
MILVAKLFITNQIGESLMINGLNNDSASLVLDAAMKVNSGFKKSWDEMSC
AEKLFKVLSFGLWNPTYSRSERQSFQELLTVLEPVYPLP NELGRVSARFS
DGSSLRISVTNSELVEAEIRTANNEKITVLLESNEQNRLLQSLPIDRHMP
YIQVHRALSEMDLTDTTSMRNLLGFTSKLSTTLIPHNAQTDPLSGPTPFS
SIFMDTCRGLGNAKLSLNGVDIPANAQKLLRDALGLKDTHSSPTRNVIDH
GISRHDAEQIARESSGSDKQKAEVVEFLCHPEAATAICSAFYQSFNVPAL
TLTHERISKASEYNAERSLDTPNACINISISQSSDGNIYV TSHTGVLIMA
PEDRPNEMGMLTNRTSYEVPQGVKCIIDEMVSALQPRYAASETYLQNT
> NleE_Crod
MINPITNTQNLLSINTKHAEYVVKNIYPEIEHNYFNEPPNVYDKKYISGV
TRGVAELKQEGFINEKARRLAYMQAMYSVCPEEFKPISRNEASTPEGSWL
TVISGKRPMGQFSVDSLYHPDLHALCELPDICCKIFPKENNDFLYIVIVY
RNDSPLGEQRANRFIELYNIKRDIMQELNY ESPELKAVKSEMIIAREMGE
IFGYVLEEIDSYMKNMNNKLAQIEARRSAT
> hopAH2-2 Pto DC3000|Genbank: AAO56771
MNIHTARSYDPLMATSEKPATADRTTRAVDAGATEPTPEKQAKLDTYTAS
DSRSAETAEGSPVYTRMMLSRNTRSLESEDSSSDTALLASQKALVSQNDT

KAVGSEVAAVKDSTALSDAEKKAKEAEKAASTEAAKTAQTVARGGANVNP
DDIATPAAATKMIQDLKAIGGGTLRIQMTHDQIRDVKQMDKLRALLNEGG
KQDVKVQFTFRDNANQGSGNVLTGDKLKQAAADISSVVKKFGSRPGFVLD
TFHQGGNSASQEWSDMQTTLIKSARNAGFKGSIVVEDSNWGGGLTAGPQS
GLVKFADQLKAANGEGNPALIGGFHVYARESEASSRLGKQIKALQEAGYK
LQIGEVGNASLLIGSTFRQKDDATKAMTDNMAALKAAGADILPGKDQFQD
GKLRRRVGFSKSDQYS
> gi|15596905|ref|NP|250399.1| translocator protein PopB [Pseudomonas aeruginosa PAO1]
MNPITLERAGLPYGVADAGDIPALGRPVARDVESLRVERLAAPAAASASG
TGVALTPPSAASQQRLEVANRAEIASLVQAVGEDVGLARQVVLAGASTLL
SAGLMSPQAFEIELAKITGEVENQQKKLKLTEIEQARKQNLQKMEDNQQK
IRESEEAAKEAQKSGLAAKIFGWISAIASIIVGAIMVATGVGAAAGALMI
AGGVMGVVSQSVQQAAADGLISKEVMEKLGPALMGIEMAVALLAAVVSFG
GSAVGGLARLGAKIGGKAAEMTASLASKVADLGGKFGSLAGQSLSHSLKL
GVQVSDLTLDVANGAAQATHSGFQAKAANRQADVQESRADLTTLQGVIER
LKEELSRMLEAFQEIMERIFAMLQAKGETLHNLSSRPAAI
> Q4ZX76_PSEU2
MTIMSSLAGAGRGVVNTIGGAAQGINSVKSSADRNAALVSNTGSTDSIDA
TRSSISKGDAKSAELDGTANEENGLLRESSMLAGFEDKKEALSNQIVASK
IRNSVVQF
> hopAR1 Pph race 3|Genbank: M86401
MKIGTQATSLAVLHNQESHAPQAPIAVRPEPAHAIPEIPLDLAIRPRTRG
IHPFLAMTLGDKGCASSSGVSLEDDSHTQVSLSDFSVASRDVNHNNICAG
LSTEWLVMSSDGDAESRMDHLDYNGEGQSRGSERHQVYNDALRAALSNDD
EAPFFTASTAVIEDAGFSLRREPKTVHASGGSAQLGQTVAHDVAQSGRKH
LLSLRFANVQGHAIACSCEGSQFKLFDPNLGEFQSSRSAAPQLIKGLIDH
YNSLNYDVACVNEFRVS
> A1JRY5_YERE8
MKITPTIIQTPFPFENNNSHTDIVTQPILGKLIGEGSTAEVFEDVNDSSA
LYKKYDLVGNQYNEILEMAKQESDLFNAFYGDEASVVIRHGGDVYLRMLR
VPGTPLSDIDTADIPDNLESLYLQLICKLNELGIIHYDLNTGNMLYDKES
ESLFPIDFRDIYTEYYSATKNDKEIIDRRLQMRTNDFYSLLNRKYL
> Q8XQ11_RALSO
MVFLVRSINVAQSSHDPSTSDDQPFAMSPPSPGERPARRSPAAVLAGLVG
FSRRRGAGETAHGHEGGRSLLRRFRAPRYMNLRAQGPSSPREPLAVWAQA
CELQPETVEALRALRDQEGSGPFMSLLAKLDACESFEKEPHRADSVQELG
AVLKLAAHNDAYRAFCFDVAGGADADCYDNAEVIFGNLRLAARDPTYHGN
ASLEQVLNYHKRCVPWSLVDDFVSERFPLFAESLENVLALRIRLSDILPI
RTPAMTFDNMTSVNQGVEAQARAYIARHCDREAKLQRNLCRSPAWRQFME
RQHPVEFTANTLLWASALQAVMEQRPEGAAMAVPPEVNTVSFGSRTEALA
RARAMPGIGTGHAFRHLQQNATVLLSEDLTRRLVVEKRPPRTEAKAYAHL
LRDPDWLTYLEQEHPDDPAFSSDGIGTPDRHERLMRLTQQEIVAARGG
> gi|20139268|sp|Q9RBS1|POPB|RALSO PopB protein
MSHSKIKAGGHGSSGIGNDFTPAKTPAPATPAPQSQQVNDLLGRGVGNAL
NKSNLGSDSQTWTPGSTMVSLKSRSSSSHKPDTGGDTKPDSTSGGKRKRD
DETDPNAETEGGKKKKKRDDENDSSQAGGAGSSAGSSGSPEDALMNIALQ
RAIQRQTQTRQKMQEAMKIKDDDD
> Q4ZV89_PSEU2
MITPSRYPGIYIAPLSSEPTAAHTFKEQAEKALDHISAGPSGRELLQEIS
KLASKKDRKVTLKEIEINNQCYTDAVLSRRQLEKYEPEDFNENRRIASRL
SRKGTFTKGEGSNAIIGWSPDKASIRLNQNGSPLRLGMDNDDKITTLAHE
LVHVRHVLSGSSLADDGDRYNPRTGSGKEELRAVGLDKYSYS LTKEPSEN
SIRAEHGLPLRMKYRPHQ
> Q2RKJ5_MOOTA
MEQISIIADDLTGANDTGVQFCQHGFRTMVIIDAANVERVGQDKDVWAIN
TDTRHLAAPEAYQRVYEITLKLKKAAISRVYKKIDSTLRGHPGAELEAVM
DAWQADLALVVPAYPANRRLVVDGHLLISEGMETAAASVSLTPGDARAAL
CHIPTVLQGEMGRRVGQINLATVRQGVKELVAALEAARTNSQVLVLDAAD
EEDLRNIARAISRFQRDVIVAGAAGMAAHLPLAWNLKPVPNNPLNKKGAI
LLVAGSRNPVTAAQVQRLAEVSACQAVKVETEAILTGEPAVEIERVLQEV
TTQDAGAGLIIIAVDSLFQTIDRDRVSNSGSKAIALALGTITSRLLNMRR
ISALVVTGGDTAVHVCRALEARGINLAADLLPGIPLGYLEGGRGDGLPIV

TKAGGFGSPDSLIKVNEFLQQRMKSEMELV
> Q8X4X3_ECO57
MPVDLTPYILPGVSF LSDIPQETLSEIRNQTIRGEAQIRLGELMVSIRPM
QVNGYFMGSLNQDGLSNDNIQIGLQYIEHIERTLNHGSLTSREVTVLREI
EMLENMDLLSNYQLEELLDKIEVCAFNVEHAQLQVPESLRTCPVTLCEPE
DGVFMRNSMNSNVCMLYDKMALIHLVKTRAAHPLSRESIAVSMIVGRDNC
AFDPDRGNFVLKN
> STM2879 sicP secretion chaparone 3023689:3024081 re verse MW:14449
LQAHQDIIANIGEKLGLPLTFDDNNQCLLLLDSDIFTSIEAKDDIWLLNG
MIIPLSPVCGDSIWRQIMVINGELAANNEGTLAYIDAAETLLLIHAITDL
TNTYHIISQLESFVNQQEALKNILQEYAKV
> Q8XQT8_RALSO
MDAGRLPPSFVPPATLSPDATAGDHTIQTQNEPPRSPRKRASDVTSNLPR
PARQPSAEPLEPASPGQAPSGFRTVPLPASTSSTVTQTEAIQ PAPARIDP
PLLQQSMAAWLPADVYDNARFTARSIREYAQEQLGLPNDADVVAHLFNAL
PEDQRTEPNRELLDKAMQHSVNASGAAMLMVNTDAGLQLVAANSQRHKIV
IQTNGACEKGESIRQTVRREFKEELGNPAPNGILLGTLSEANLRAVNGLN
YIGHTAAEIAAHIVKVEADPSELFLNVTSLFVNRAPVTMQALEAEVAHLN
ERLARAKPFYQEAVHYIYGDAKTTFQQDAQVRGEAANVVKRFR QACPDNI
TENFAQCLDAIKADGTDDMDALKQALAAIIDLAENDAIKLIDEPTFAQAM
RLATRMDSDEAAKTALENDYFDMSFIGGALHLGDAEPEAFMAQLKAGETA
PAIGRPVLNKDDAAQPEAAIRQAA
> gi|34498093|ref|NP|902308.1| hypothetical protein CV2638 [Chromobacterium violaceum ATCC 12472]
MTPDTVAILHNLSRQLAEGSGES LDIRECARLSLQMEKLAGKLPADSPLA
ELSERLALQGQAAWAQLARAEAETQLLPQFQRYDRPGAGSEKHAGASVGA
GLGLGEGGAGAKATFSVGLGWGRSMDNDDEGFVFQNKSHSASASAGVKGG
VGIASLSGGVEGRVQKTRYREYNSAKAFVHLNAERLRHGSRRDTLGVGAR
AAVGALIRLFRPGHGNELQHYHRLQQQAADQQQRLGVLQGWLGQRDASSA
LPGGRAPVVPSGDVVSVKGSANAR AAAAGVSAGAAVSVERIDIQADSLTP
YWQALSAPEGAARSAVVAAQRQAGIEAKAQALFDGPKDKAGTRPLSRLTG
GGAASSAALRECSPAALRQAAERLGAELDHFCAVAQQLDAGVGKRQGGAQ
IEKSIVSSWQGKRREDALANMALAHAALLIAARSRGDAGEAERALKDMAA
RLYAPPVRHNAEALSKRVAFRDTLELQIRNRSYALDLGGGVGPLGVKLEA
SLTEQHRVHLNPVRAGDYKDLRVTL SGSLGDAGALNKLKDALAAQLAPHG
LADALPAALAGMETALTGQLGAGATLLLRFYRPQYQGQADFPQEAAGFRL
QLARVVASGDASVALSGGVPLQPGVSLELGLSAGGSASVVAYERWGDNTL
TAPMMHYLHLQGVGEPERWQGMADMQKPALSGLFRQLADPGSAVRKEAEY
FLKRQPQDGFGERFFAAMSGFSSGEVDFDGARTALDEMMQRQLPLWQADK
LAFPGLVEQPLAA
> gi|34498037|ref|NP|902252.1| probable secreted protein EspA [Chromobacterium violaceum ATCC 12472]
MNTTVNQTTPGVNALTTGAADSSDNNDYLRAARNYSLLGQAITTMEEVML
LFTELSNAKFAQMSKKMEVSRDAAEMANKVEALLASITDPNGKASLPEDV
IEYMRKNGVAINGKSIDEFLRDDADLDSRWALNNIHSYTIGHTYFCLDSA
QAIAKNLDDAGVKI DGQKASDWLKNQENVDGSYSKEAMAKLFSNGRQLLG
RADLTAVKSALETFSGRASDFVQQSQLKMQQLIQNFNTAVTMANSLQSMN
AESTKSIAQAIR
> gi|53722547|ref|YP|111532.1| putative invasion protein [Burkholderia pseudomallei K96243]
MSARRAEAARPRGYAARIARVAAAFAIGIVSAGGVAHADCIDDAARRYGV
NADLLRSIAYYE SGLNPRALHRNGDGSTDIGLMQINSVHLPALRDQGIDR
LRLYDASINARVGAALLRRQIDQYGDTWRAVGAYHSRTPGLSERYARAVH
DVYVARPWAAKAAARRDAAHTMPARAPGLVIEEASVQ
> Q8XAL7_ECO57
MLPTSGSSANLYSWMYVSGRGNPSTPESVSELNHNHFLSPELQDKLDVMV
SIYSCARNNNELEEIFQELSAFVSGLMDKRNSVFEVRNENTDEVVGALRA
GMTIEDRDSYI RDLFFLHSLKVKIEESRQGKEDSKCKVYNLLCPHHSSEL
YGDLRAMKCLVEGCSDDFNPFDIIRVPDLTYNKGSLQCG
> hopX2 Pma ES4326|Genbank: AF458041
MGLCVSKGSTASSPQHYAVRYTEQVTPSPSPSVASTSPSLHDSTAIGSPL
ARRSVLLPHEVQQAAYQLAMRLNGRAIEDASDRKRLVDATATVHETRLAL
HRGRGKVDSDLRLSNGRSATYSSLSYCLGEDDENLLA GSALAAGAGNCDH

NAAINTRRHAVRMEDGGQMMTVRDYEQTHLYALYQPPNSAEADESTVVLD
SWGDGPAVLLRDSHWAESYGTSTHVIERFDKPDATDALARTNAFRAEIEN
PQTALHANARELETAFLANPAPGDIFSAMPVIAPHLAESTRRRLQECSPR
TRNVLAADAARDAYGLDDAQANSPRTTKAILKEAKRLDALGRPPLSW
> Q8Y1T1_RALSO
MRRIGNFFRTRSHASGSSRGRTHERE EGSSGRAARSPSPALSDLPSSPRS
RHVSTVASPTRFDDDGRVTYPGHIQYQTSESERERYPDLAEAVGHSRYTG
PGSPRPERFPISGYFPDGQYFSVTGLRGPAIRSGDDAVIDRQHASHVIDH
GEMSDAQAGRFMDWQNRSIYPTYETSGWEYSNCVHGHAEVGRSVFGWDVY
PDQHARPQDLADQMGNLRVRNYNSDSDSDG
> gi|28867816|ref|NP|790435.1| type III effecto r HopPtoH [Pseudomonas syringae pv. tomato str. DC3000]
MITPSRYPGIYIAPLSNEPTAAHTFKEQAEEALDHISAAPSGDKLLRKIS
TLASQKDRKVTLKEIEINNQCYTEAVLSRRQLEKYEPENFNENRHIASQL
SRKGTFTKGEGSNAIIGWSPDKASIRLNQNGSPLHLGMDNDDKITTLAHE
LVHARHVLGGSSLADGGDRYNPRTGSGKEELRAVGLDKYRYSLTKKP SEN
SIRAEHGLPLRMKYRAHQ
> gi|28872465|ref|NP|795084.1| type III effector HopPsyA(Pto) [Pseudomonas syringae pv. tomato str. DC3000]
MNPIQSRFSSVQELRRSNVDIPALKANGQLEVDGKRYEIRAADDGTISVL
RPEQQSKAKSFFKGASQLIGGSSQRAQIAQALNEKVASARTVLHQSAMTG
GRLDTLERGESSSATTAIKPTAK QAAQSTFNSFHEWAKQAEAMRNPSRMD
IYKIYKQDAPHSHPMSDEQQEEFLHTLKALNGKNGIEVRTQDHDSVRNKK
DRNLDKYIAESPDAKRFFYRIIPKHERREDKNQGRLTIGVQPQYATQLTR
AMATLIGKESAITHGKVIGPACHGQMTDSAVLYINGDVAKAEKLGEKLKQ
MSGIPLDAFVEHTPLSMQSLSKGLSYAESILGDTRGHGMSRAEVISDALR
MDGMPFLARLKLSLSANGYDPDNP ALRNTK
> Q8X9A5_ECO57
MPKISSVVSSCYHLFSEHQQLSNETTMTNPVSRRIVHKEYGISLKSVPVW
LATAKTPLALLNGRHTRSHSFIIAGTPGMGSRSGAQYYAINSDDKRSRID
IDSLFLKKLNNVRNQNKFPIDVKETVIKLQGQKFTCIEDFYKRYNETRLK
ANTNIQQEQIADEVKSLTYLIPSEKKEMWIYKNNGKDNAKPNLGERDVRM
FENISSDDTDKITGRKFSELGEYLYSGNVI KLSQLSIRYLPNISSISLIE
TKQSLLLHRLYSDEVLQRNGTLIPTPLHEEKSIPADNIKTMLNNIPTYKM
LPPFTETQGNCSSGAATFLRKSGAEEKDILACSPRNYGLHHNIKTWDPLV
RN
> gi|12329056|emb|CAC05787.1| IpaH7.8, member of the IpaH family, secreted by the Mxi-Spa secretion machinery, function unknown [S higella flexneri]
MFSVNNTHSSVSCSPSINSNSTSNEHYLRILTEWEKNSSPGEERGIAFNR
LSQCFQNQEAVLNLSDLNLTSLPELPKHISALIVENNKLTSLPKLPAFLK
ELNADNNRLSVIPELPESLTTLSVRSNQLENLPVLPNHLTSLFVENNRLY
NLPALPEKLKFLHVYYNRLTTLPDLPDKLEILCAQRNNLVTFPQFSDRNN
IRQKEYYFHFNQITTLPESFSQLDSSYRINISGN PLSTRVLQSLQRLTSS
PDYHGPQIYFSMSDGQQNTLHRPLADAVTAWFPENKQSDVSQIWHAFEHE
EHANTFSAFLDRLSDTVSARNTSGFREQVAAWLEKLSASAELRQQSFAVA
ADATESCEDRVALTWNNLRKTLLVHQASEGLFDNDTGALLSLGREMFRLE
ILEDIARDKVRTLHFVDEIEVYLAFQTMLAEKLQLSTAVKEMRFYGVSGV
TANDLRTAEAMVRSREENEFTDWFSLWGPWHAVLK RTEADRWAQAEEQKY
EMLENEYSQRVADRLKASGLSGDADAEREAGAQVMRETEQQIYRQLTDEV
LALRLSENGSRLHHS
> Q471Y7_RALEJ
MTAIQQQDRPLLGCIADDFTGATDLANTLVRNGMRTVQTIGVPDAHAVRD
VGQADAIVVALKSRTIPAADAVSQSLAALEWLRAQGCRQFVFKYCSTFDS
TDAGNIGPVAEALLAALDSDFTIACPAFPENGRTIFRGHLFVGDGLLNES
GMENHPLTPMRDANLVRVLQRQSKAKVGLLRYDAVARGPAATTARIEALR
ADGNHLAIADAVSDADLFTLGEACANLPLITGGSGIALGLPENFRRAGLL
PQRGDAASVPRVDGHGVVLAGSASRATNGQVARWLEQGRPALRIDPLALA
RGEAIVDTALAFAANHDEPVLIYATSSPDEVKAVQAELGVERAGHLVEQA
LAGIAAGLLARGTRRFVVAGGETSGAVVQALGVRALRIGAQIAPGVPATV
TLDAQPLALALKSGNFGGEDFFAEALAQLGGQ
> Q8XUH6_RALSO
MRVLSRFSFTAQPRADSAEPKKAPVAGSRGAAARPAALEKLTAFSRSNAA
KQANSFVRSPLPLRGDRYSSEPGVLPSAGQFDAHIWDELPTQMAQCAVAR

TRQGLAFRARFGAESAQHYSGSCVGLSAVWIRLHEAAPATHAVNRVNTAG
SFDGMAHAKVYQRAYEANQSDMLQGRASKRFGKSDMARLDAIAQEQPSQI
LGLTIGTEAY SHKSVGSTARVLTEFDGYGLLALRMAGSRGAINGHAAALH
RQPGSSHITFFEPNLGEFHIPLHDTKDFLQAYAGMQKSLGQPVSQFDLLP
VGVHGSIHDTPLQTLAHSLVS
> A3M1Q5_ACIBT
MAQELLAQLQAGTAKFSDVLAYIEARYQHTPTAFQNGAQHNAATENQGSA
KVFSFAKLQGLDQVQTLSLFAEHYASVLATPEGTDHQNIRQFMQNGWDGV
KFEGQALTEK
> Q8XTS6_RALSO
MGNGFSVNRHGPVVPQDVGPRSASADVDTPAASPAHPARTASGPLLGLRS
AQAQHAVAAASGQAADGEIGPAIGAPDRLPEPRTEMAPPSMRAEQHLPAV
AGDEASRTDAEPLSGARKLPPEMWTVIAHRDPVAQQRLRVVSRTTRAAAE
VGIRQLIVKTPEGLAAVKRPGSYPNLRELTLVGNFWGNDLIGLPATLREL
DLSGCSGLRVTGLDPLLALPLDQLNVSGCRLGAEGARQLANHPTLTSLDM
RGNQIGDEGATYLADNPRLTVLDVGDNRIGDPGVIALARNTVLASLHLDG
NTFGLDGIRALAKSATLTTLNVGKNRIGPEGAEALAEHAALTTLLIEENA
IGDRGAQRLAANKILTTLNVRNNGIGPDGGLALAASTSIVSLAIGHNRIG
SGAVLALAANRKLTSLDVEYTEISAVADAADAQAAAAAVAEVASALAAST
SLVHLNVGTNCLGDEGARRLAASKTIKTLNISYNKIRLDGARALAANRMI
ASLDLAGNVIGTPGVKALAANTALATLNLRQSQIEPEGVVALAANKTLAS
LDVGANKLDPDSLRMLLTQMNLAVLDATFSGCEPGAGAELTALAARIGVT
LRI
> gi|34498074|ref|NP|902289.1| cell invasion protein [Chromobacterium violaceum ATCC 12472]
MSDASAISRSAYLHNPKLAETAFESVRKDGTFLDAADKALKAVLATKAGE
ADGKAPIRERDLATPALTQPTAKAKEELDGAGKLTLLLGQLMSLLGNVSL
SQLESRIATWRAMMEAQVAMGEKLSQEFQDAFKAASEATEALKDAVGNYD
SAKQAADAAQKKADAAQAKVDGMSPDEPGYAEAQAASQQAAADALQAKQK
ADKAYDAAEKARVVAVEKGKVADDLAGKVQGANVNADVARKTGEDNLSNV
AKLTLLMAMFVELVGKNSEESLKNDLALFQALQEGRQKEMDKKSAEYQEE
VRKAEELNRTMGCIGKILGALLTVVSVVAAAFTGGASLALAAVGVALMVA
DEVVKAATGVSFMQEALKPLMDKVLKPLMDLIGKAITKALEGMGVDKKTA
ELAGSIAGAVLAAVAMVVVMVVVAVVGKGAAGKLGGALSKLLGDAIKKIV
PNVLKELAKGGSKMLTQGFQRLANSLGLQTDAAAKQMMANTLGRVVVGGE
VAQATSQAGGNVAQGVFMKNASDALADFTLARAAMEQIEQWLKQAVEVFA
STQKVTQELTTAMSAAVQQNAEAGRFVLRQSHA
> Q8XB12_ECO57
MSVGNEEKYSITHKDITNLIAGDRQSFLKLTLWEFILDLFPMYHIKEAKE
TIVEFVTKTEDKVKRFERIKSLAIPEEKWRFSTTTEFTVDENKDIIVSRV
FKININSKDNHNNAEENTMRSVLSEERNYNSYKHDIHFDEKAFQSGILKM
QFPNDNEKLSVYLKNKIPFENMIIDLSALKKDVLVSLKQCCFKKMTFTGN
ISYENLNGPVFENCFFEECNFESVSLVGFDKVTCESYDVLCNVKPNNKIP
IYGMFKGCFLYQCEMKNFKIETSKIYSINQDPQRGDKKVGAYLFMQSFVY
ASNLQDGVCKEASVIASSLLSCNIAKLNGVGMDFIETSFYGKTRGGFRDS
NNFYDCDFRYVNMTCKRDGIREDLRDYDPRKYFGESENKNKLNLDKALLN
FIHDDIFQNIKTHYNIKDDIDKHLNLNHCCLYAACLGELTGGNPLYDCAI
DTHTTILSGNLAANTQVKIPVYMYHRGAIAEKDILPFMEKVTNIYSRIED
IYKYSNNMKNNYKKALADLQSLLVNLHNIMPGYDVKDFQLNDETIKYIID
NVLYNRKKDIASEIDTDKVNFMHKIYNNLYWYRHYDENGNEKTYRTANPN
GNRFYFDFAGLQVLYYDYENKNKPTKEKFLEKQNLFLTVKNFKAEEKINQ
EKYIEKPANVAHEIASFFDIEQYLLGGIDRNNNYINENFTYKFQDLANLS
GTRDAQLERKVYFNEKMLNLNDNDEKVKIENDIKKIDKKIEEIDNAIRDL
SSETERFKIKYQQENTLNDVVQNIRTWLKENQNMVKAIKKIDTE
> CNF1_Ypse
MKNQWQHQYFLSYSELVANFPSPEKVVSDYIKHKFSTTLPWFGWADPDNL
YFIRFTQSRSNNKSYTGWDHLGKYAIETLTLTQAAIVNIGSRFDIFDEAN
STAGIYKTNNADSFDETNEAKMLPSEYLYFLRDCDFSNLYNKALSDYWAE
NYEKFSTLLQNYYISSAYYLYKDSAISKDEYEFSIDAIFNKKSKILRYYF
DVYGYYSSDMFVAMNDNKTMLFIPGATNPFIFADNITDLRDKIKALISDK
NTRELFSKHFSLYDRQDGNTYLGVNSMLEQIVSGVVDTNYIMYSNKNIRE
RNVFGSMAFSTRERSFNDGDVIIKSNAEVQRDYALNVLQTILSLSPIFDI
VLPEVSIPISLGITASSVGISFDELINGDTYEERRSAIPGLATNTVLLGI

SFAIPFLISKA EENKLIINNLVGSDENILNKNNLGDFLEKYNISESDIPE
NGSLVINLKNTNVPVRLVKLNDEEGEIVAIKGSTLSGIYYEVDTETGYEI
LSRRVFRTEYNEKIYWTRGGGLKGGQPFNFEGLDIPVYFIDKPYSELASS
VELSFVNDDSPLLFPEMDSRLPKPTPELDIKYYSSNLSSFKEDTVILMRG
TTEEEAWNIANYKTAGGSNKDLEENFIEAGPQFNLSFSEYTSSINSADTA
SRKHFLVIIKVQ VKYISNDNVLYANHWAIPDEAPVEVLAVVDRRFIFPEP
PVKPKLSFIQKIANRFLTENVAEISSINFRRLNSGNINVLKGRGVFSSRR
LREIYLRFDAANADELRPGDVYVKKTKFDSMGYDSHFYNEGIGINGAPTL
NTYTGEYVADSSSQGATYWLKYNLTNETSIIKVSNSARGANGIKIALEEI
EENKPVVITSGTLTGCTVVFARKGEYFYAVHTGNSESLIGFTSTSGVAKA
IEVLSSLSELEVP ALPDVINNNTLVEYLSDNFDSALISYSSSSLKPNSMI
NISRENVSTFSYYTDDIQLPSFGTSVTILVRTNDNTVVRSLSESYTMNSN
SSKMVVFNVLQKDF
> CP0125 ipaA IpaA, secreted by the Mxi -Spa machinery, modulates entry of bacteria into epithelial cells 107178:109079 reverse MW:70065
MHNVNNTQAPTFLYKA TSPSSTEYSELKSKISDIHSSQTSLKTPASVSEK
ENFATSFNQKCLDFLFSSSGKEDVLRSIYSNSMNAYAKSEILEFSNVLYS
LVHQNGLNFENEKGLQKIVAQYSELIIKDKLSQDSAFGPWSAKNKKLHQL
RQNIEHRLALLAQQHTSGEALSLGQKLLNTEVSSFIKNNILAELKLSNET
VSSLKLDDLVDAQAKLAFDSLRNQRKNTIDSKGFGIGKLSRDLNTVAVFP
ELLRKVLNDILEDIKDS HPIQDGLPTPPEDMPDGGPTPGANEKTSQPVIH
YHINNDNRTYDNRVFDNRVYDNSYHENPENDAQSPTSQTNDLLSRNGNSL
LNPQRALVQKVTSVLPHSISDTVQTFANNSALEKAFNHTPDNSDGIGSDL
LTTSSQERSANNSLSRGHRPLNIQNSSTTPPLHPEGVTSSNDNSSDTTKS
SASLSHRVASQINKFNSNTDSKVLQTDFLSRNGDTYLTRETIFEASKKVT
NSLSNLISLIGTKSGTQE RELQEKSKDITKSTTEHRINNKLKVTDANIRN
YVTETNADTIDKNHAIYEKAKEVSSALSKVLSKIDDTSAELLTDDISDLK
NNNDITAENNNIYKAAKDVTTSLSKVLKNINKD
> gi|46400191|emb|CAF23640.1| putative CPAF (chlamydia protease -like activity factor) [Parachlamydia sp. UWE25]
MNNFFLNTFRSCAIFVLFYASLSFAS TEDIKKESMIEDLKMIKHAFDVGY
APLEWKKNYTGLNLDEELNKSIDLILSNPTLTHKDFQRIVKNFLASTKDY
HVDVIFFSTETATLPFDVKGVNNRYFITWIDELKLPPSTYTIKVGDEIIE
FDGRPLGDVIEELKQHGSKNSNPLTDQALAEIKLTNRLGWLGDIIPQGPV
TIKVHSRSKEVPLSYQLIWDYRPELIFSPSFFLQTVESFFPNKKISVRSP
CMTMMNLTHRKMMSETKRDGTLCARKS FIPTLGPRVWMFDKIDKEKEISW
YAYIYKISEEEKIGYIRIPHYIGYKEESKEFGELLNYLEQKTDALVIDQV
HNGGGYASFQYELASMLALNPLKTPKHQMKITQKDVLTAYQILDVIEKIQ
QGIDDEIEEGPIDYQHLLFLKAFYEFTIQEWDGQRTLTDPTHLEGCDWIN
PHSDYRYTKPILMLINELDFSGGDFMPAIMQDNQRAVLFGTRTSGAGGFV
LQASFPNNNGIAAFSYTGSIAERPETLL KIENLGVTPDIVYSLTVDDLQN
GYQGYKAAVNEAIQALIKKK
> Q8XAL6_ECO57
MLSPSSINLGCSWNSLTRNLTSPDNRVLSSVRDAAVHSDSGTQVTVGNRT
YRVVVTDNKFCVTRESHSGCFTNLLHRLGWPKGEISRKIEAMLNTSPVST
TIERGSVHSNRPDLPPVDYAQPELPPADYTQSELPRVSNNKSPVPGNVIG
KGGNAVVYEDMEDTTKVLKMFTISQSHEEVTSEVRCFNQYYGSG SAEKIY
NDNGNVIGIRMNKINGESLLDIPSLPAQAEQAIYDMFDRLEKKGILFVDT
TETNVLYDRMRNEFNPIDISSYNVSDISWSEHQVMQSYHGGKLDLISVVL
SKI
> gi|16764975|ref|NP|460590.1| secreted effector protein [Salmonella typhimurium LT2]
MPLSVGQGYFTSSISSEKFNAIKESARLPELSLWEKIKAYFFTTHHAEAL
ECIFNLYHHQELNLTPVQVRGAYIKLRALASQGCKEQFIIESQEHADKLI
IKDDNGENILSIEVECHPEAFGLAKEINKSHPKPKNISLGDITRLVFFGD
SLSDSLGRMFEKTHHILPSYGQYFGGRFTNGFTWTEFLSSPHFLGKEMLN
FAEGGSTSASYSCFNCIGDFVSNTDRQVASYTPSHQDLAIFLLGANDYMT
LHKDNVIMVVEQQIDDIEKIISGGVNNVLVMGIPDLSLTPYGKHSDEKRK
LKDESIAHNALLKTNVEELKEKYPQHKICYYETADAFKVIMEAASNIGYD
TENPYTHHGYVHVPGAKDPQLDICPQYVFNDLVHPTQEVHHCFAIMLESF
IAHHYSTE
> Q1LMB1_RALME
MNRPLLGCIADDFTGATDLANTLVRNGMRTVQTIGVPASAVEADAIVVAL

KSRTIPASNAVAQSLAALRWLREQGCRQFVFKYCSTFDSTDAGNIGPVAE
ALLDALGSDFTIACPAFPENGRTIFRGHLFVADAL LNESGMEHHPLTPMT
DANLVRVLQGQSQAKVGLLRYDTVAQGANAVRARIDALRGEGVKLAIADA
ISDTDLFALGEGCAALPLITGGSGIALGLPENFRRAGLLPARTDAAEVPP
VHGHGVVLAGSASRATNGQVAHWIAQKRPALRIDPLRLARGDTVVENALA
FAGNHDEPVLIYATAQPDEVKAVQAELGVARAGELVEHALASIAASLKAR
GTRRFVVAGGETSGSVVQALGVQSLRIGPQIAPGVP ATVTLDDAPLGLAL
KSGNFGGENFFDEALQALGAPQ
> gi|21231833|ref|NP|637750.1| avirulence protein [Xanthomonas campestris pv. campestris str. ATCC 33913]
MLESADGCRRRYQLNSFGEPDRRYVANVAKAHPIRKDTRRYVTEAEPETV
TAAMLANGSVQKYETQHFALWYGIDRNGESYRDVASRGLVWKDFVAQSAR
WLEQVWDMNA QALGAPMPYATAAKPKRINVYMCATGLPYVQGGDLTECGA
SGGQAIGASSWALGYGSFSIAHEFTHVIQFYSGGYRNKQAAGWFWETNAN
WSAYQTTRMDDQWLAYYYSNLENGPFFPQSRYGAHPWLMYLDENDATRSL
VWDMWLKNLRNADGDTLELPVETLVRLGQQGGQFPNGYRSFADTVGRYGA
RLAAFDFVSQKAMLDIGNGNAAGKRYVPLKPLAAPGRYASSPERPLNIYG
THVIPLTPLSS ATSIKVSLTGKTRADQASWRFTVVSLDAQAHPRYAPLVA
VDGTASATASFPLQGGKGITHYLVVTATPYRYSEVPTGEEQLAGQAAARF
PYEVSIQGATPLVGPAATCYAYKGDDGLDRNWNTNGHQFEPQPCR
> A2A0X5_ECOLI
MINSINSFFSGLPRSISSAIRSSTFTVSQHKSTPNTVKTSSPFSPSNSPA
SATTIFKVKNSYTESGLQRPTSYTQSSIEKNALHRPLPDVAQRLMQHLAE
HGIQPARNMAEHIPPAPNWPAPTPPVQNEQSRPLPDVAQRLMQHLAEHGI
QPARNMAEHIPPAPNWPAPPPPVQNEQSRPLPDVAQRLMQHLAEHGIQPA
RNMAEHIPPAPNWPAPTPPVQNEQSRPLPDVAQRLMQHLAEHGIQPARNM
AEHIPPAPNWPAPTPPVQNEQSRPLPDVAQRLMQHLAEHGIQPARNMAEH
IPPAPNWPAPTPPVQNEQSRPLPDVAQRLMQHLAEHGIQPARNMAEHIPP
APNWPAPTPPVQNEQSRPLPDVAQRLMQHLAEHGIQPARNMAEHIPPAPN
WPAPPPPVQNEQSRPLPDVAQRLMQHLAEHGINTSKRS
> hopT2 Pto DC3000|Genbank: AAO58036
VFCRRSCMNGCRITPARVGSPKTEASGLQLNSRIEKVVVEHISGHPAAKI
TMQSLVGTLAHTFVMAHGEIKGWAEIVQSPSRPHESNRKGSGVFSACVDL
MACVGWNAALTRATADPATTTANKASW
> Q8X831_ECO57
MLSPYSVNLGCSWNSLTRNLTSPDNRVLSSVRDAAVHSDNGAQVKVGNRT
YRVVATDNKFCVTRESHSGCFTNLLHRLGWPKGEISRKIEVMLNASPVSA
AMERGIVHSNRPDLPPVDYAPPELPSVDYNRLSVPGNVIGKGGNAVVYED
AEDATKVLKMFTTSQSNEEVTSEVRCFNQYYGAGSAEKIYGNNGDIIGIR
MDKINGESLLNISSLPAQAEHAIYDMFDRLEQKGILFVD TTETNVLYDRA
KNEFNPIDISSYNVSDRSWSESQIMQSYHGGKQDLISVVLSKI
> Q8XQ98_RALSO
MRPDHHAIDIDPPAPQAEPPRAGAIAAPPVVPAWRRPATLRALSHVQATV
SAVASAGFLAHRHLPGYGLDKAALGLALANVPLTWAIVHGQLQAIRAEAE
GLRHPELRGQALDQVVKGFCAPDSAAARFDWNASGLFVNTMGMDMADEVS
HILARAFAAARTPAGSAIAPELRVYADNLASS ATLRAALAQTAYEANVAC
DDRVGVRLGELMLAGIMHMARDTTTEPRTVVGTLVLHAATRAMEQRISAL
LAQGRSPETAAPQPSAELMLAGCHAVQAALVRRGLAVPELFPEHLFGMDD
LHVHRNAVMATAEAIAEACHPSRAPGQPAGHAIVRFLERHGGEAAQAVLS
ARLAHLAKPLQATAQRELATLDATLETLSDQQYRDDADKVKQDYDTAVAQ
MRAHAIDGALAGDDADWVIPDAPAPTSEPEPAQ SHGETSADAGAAPVPDG
YDPTRGKWKTG
> gi|2865308|gb|AAC38400.1| EspF [Escherichia coli]
MLNGISNAASTLGRQLVGIASRVSSAGGTGFSVAPQAVRLTPVRVHSPFS
PGSSNVNARTIFNVSSQVTSFTPSRPAPPPPTSGQASGASRPLPPIAQAL
KDHLAAYELSKASETVNFKPTRPAPPPPTSGQASGASRPLPPIAQALKDH
LAAYELSKASETVSFKPTRQA PPPPTSGQASGPGGLPPLAQALKDHLAAY
EQSKKG
> Q4ZX75_PSEU2
MQSLSLNSSSLQTPAMALVLVRPETETTGASSSSKALQEVVVKLAEELMR
NGQLDDSSPLGKLLAKSMAADGKAGGGIEDVIAALDKLIHEKLGDNFGAS
ADNASGTGQQDLMTQVLNGLAKSMLDDLLTKQDGGTSFSEDDMPMLNKIA
QFMDDNPAQFPKPDSGSWVNELKEDNFLDGDETAAFRSALDIIGQQLGNQ

QSGTGGLAGTGGGLGTPSSFSSNSSGVKGDPLIDANTGPGDSGTTSGEAG
QLIGELIDRGLQSVLAGGGLGTPVNTPQTGTAANGGQSAQDLDQLLGGLL
LKGLEATLKDAGQTATDVQSSAAQIATLLVSTLLQGTRNQAAA
> gi|55977843|sp|Q06131|YOPD|YERPS Protein yopD
MTINIKTDSPIITTGSQLDAITTETVKQSGEIKKTEDTRHEAQAIKSSEA
SLSRSQVPELIKPSQGINVALLSKSQGDLNGTLSILLLLLELARKAREMG
LQQRDIENKATITAQKEQVAEMVSGAKLMIAMAVVSGIMAATSTVASAFS
IAKEVKIVKQEQILNSNIAGREQLIDTKMQQMSNIGDKAVSREDIGRIWK
PEQVADQNKLALLDKEFRMTDSKANAFNAATQPLGQMANSAIQVHQGYSQ
AEVKEKEVNASIAANEKQKAEEAMNYNDNFMKDVLRLIEQYVSSHTHAMK
AAFGVV
> ECs4555 ECs4555 EspD 4592002:4593126 reverse MW:39083
MLNVNNDTLSVTSGVNTASGTSGITQSETGLSLDLQLVKSMNSSAGWTES
SPLPTPPAGHSLVTPSAAEDVLSKLFGGISGEVTSRTEEAEPQRTSYPYL
SQVNTVDPQQMMMMVTLLSLDTSAQKVSSLKNSNEIYMDGQTKALENKTQ
EYKKQLEEQQKAEEKSQKSKIVGQVFGWLGVALTAVAAVFNPALWAVVAI
GATAMALQTAVDVMGENAPQGLKTAAQVFGGISMAASILTAGVGGVSSLL
SKFGNVANKIGSSVVKVVEKAAEALVKNVFAKISTVAEGVTNGIRSAGTT
ALNNEAAQLQMLSQLAAFAVQNLTRQSESLGESAKLELDKAASELQNQAS
YLQSVSQLMSDSARVNSRIVSGRI
> A4B9U2_9GAMM
MTLQELMATLHGHPDDLKFADVLETIDAEFEFTPCAFVNGDVHNSADENQ
GSCKVLCFAHKAGLAEGVALKLFAEHYRSVLADPKGTDHANIRQFMKRGW
MGVSFNGSPLKKK
> gi|15834669|ref|NP|296428.1| serine/threonine -protein kinase [Chlamydia muridarum Nigg]
MLELGVSFPSKTKYLLTRELSRKVGLTVYQGVDESSSRPVVIKALVSPGI
HDQRFLRAFEEEARIMQLVDHPAFVRLEEKGEWEQGRYFVSEYILGHSLR
DIILSSHLALDKAVSIVLQVAQAITALHKHHVLHLDIKPENIMISRLGEV
KLIDYGLSAWQFNHWGSPAYMSPEQSRQEKLSPASDVYALALLAYELIMG
QLSLGKVYLSLLPVKISKVLTQALQPDPEARFPSMQEFATALQDYLMHDV
HEDYRKKDRVIMQFEQLQQQNMWLAPDKLCMPEGMALHIYSQKEPCDLHN
VYYDILRSEDIVELWFCYAQGHCSFALSMIKQFLNQRTEKAQDIPTVIKT
LDTLCKTMHIPLCEKGISCCCFIFFQQELMCFSCGKTDFSLKKQTRGVQR
FQAESQGIGEEGPLEIHKQSFLWEPGDELIVHTPRARDLVYLYCPSFLKL
QDRGQMDIFCQTDYLQKEVRQKYDGSLYPSTLISLKRVR
> gi|15834868|ref|NP|296627.1| hypothetical protein TC0248 [Chlamydia muridarum Nigg]
MKMNRILLLLLLTFSSAIHSPLHGESLVCQNALKDLSFLEHL LQVKYAPKT
WKEQYLGWDLSKSSVFAEQKLRSEDNPSTSFCQQVIADFIGALSDFHAGV
SFFAVESAYLPYSVQKSSDGRFYFVDVMTFSSDIRVGDELLEVDGQPVAE
ALATLYGTNHKGTLAEESAALRTLFSRMASLGHKVPSGRITLKVRRSSGS
VKDVRAKWRYTPESVGDLATIAPSIKAPQLQKSMRGAFPKKESVFHQSST
LFYSPMVPHFWSEFRNHYATSGLKSGYNIGDTDGFFPVMGPV IWESDGIF
HAYIFPLVDENGRSHNVGFIRIPTYGWQEMEDLDSIGTPPWEEFGKIITL
FSEKTEALIIDQTNNPGGSVMYLYGLLSMLTDKPLDLPKHRMILTQDEVV
DALDWLNLLENVDTNAEARIALGDNMEGYPIDLQAAEYLKSFAHQVLACW
KNGDIELSTPIPLFGFEKIHPHPRVQYTKPICVLINEQDFSCADFFPAIL
KDNDRALVVGTRTAGAGGFVFNVQFPNRTGIKSCSLTGSLAVR EHGDLIE
NVGVEPHIEIPFTANDIRYRGYSEYIQKVQKLVAQLINNDSVIILSEDGS
F
> Q8XTA1_RALSO
MGCFNVTGTSGTASNYVAREHSVEASPTHTPQQTGRRRAPGTPPSRASNT
DAAEMRALRNQAQALQQRAIQELPNPRTDAASTLLDSLLTLVTSINRMDP
STAQNRLAQLEGRVNSFYTTNPVGHDQLDQSDRFSDTASHASDIYHPSDS
FRFMSYRPGR
> gi|15834667|re f|NP|296426.1| hypothetical protein TC0042 [Chlamydia muridarum Nigg]
MEVNKTTESLFSAKVEHNHAQAESHEPRDQRDVKVFSLGGKSSSKQEKLD
HGSGRTSSRHETSRSSKEGSIEDKSAEVSSREEEEENRDGFLSGGNLTAG
VAFTDTPMAVASEVMIETNAVTMSQIDLQWVEQLVLSTVESLLVADVDGK
QLVEIVLDNNSTVPEAFCGANLTLVQTGEDVAV TFSNFVDQDQVAEAMQL
VQQNPEQLTSLVESLKSRQLNLTELVVGNVAVSLPVLEKVETPLHMIAAT

IRHHDQEGDQEGEGRQEQHQGRQQEKKLEEAQI
> YPCD1.06 yopE putative outer membrane virulence protein 3600:4259 forward MW:22987
MKISSFISTSLPLPTSVSGSSSVGEMSGRSVSQQTSDQYANNLAGRTESP
QGSSLASRIIERLSSVA HSVIGFIQRMFSEGSHKPVVTPAPTPAQMPSPT
SFSDSIKQLAAETLPKYMQQLNSLDAEMLQKNHDQFATGSGPLRGSITQC
QGLMQFCGGELQAEASAILNTPVCGIPFSQWGTIGGAASAYVASGVDLTQ
AANEIKGLAQQMQKLLSLM
> gi|50121031|ref|YP|050198.1| harpin [Erwinia carotovora subsp. atroseptica SCRI1043]
MLNSLGGGTSLQI TIKAGGNGSLFQSQSSQNGASPSQSGFGGQRSNIAEQ
LSDIMTTMMFMGSMMGGGMGGGLGGMGGGLGGALGGLGSSLGGLGGGLLG
QGLGGGLAGGLGSSLGSGLGGALGGGLGGALGAGMNAMNPSAMMGSLLFS
ALEDLLGGGMSQQQGGLFGNKQPASPEISAYTQGVNDALSAILGNGLSQA
QGQNSPLQLGNNGLQGLSGAGAFNQLGSTLGMSVGQKAGLQELNNISTHN
DSPTRYFVDKEDRG MAKEIGQFMDQYPEVFGKPEYQKDNWQTAKQDDKSW
AKALSKPDDDGMTKGSMDKFMKAVGMIKSAVAGDTGNTNLNARGNGGASL
GIDAAMIGDRIVNMGLQKLAS
> gi|17428371|emb|CAD15058.1| GALA PROTEIN 4 [Ralstonia solanacearum]
MVSRIPTGNRGQGNGIEQSASSHATEATPWLGVNLPYGHWLPSSARSVVS
RASSLLGGLANFRWGGRPASARPAP NIRSAAIAPAQQTLPPELWQQIATL
AGARPRRAMREVSLELRNASRAVVTHLTISDPAMFRQLSLYPALKSVRFK
GELTLEALKALPPELEHLEIGRCTGSAISAEGLAHLASMPLKSLNLNGIE
IGVEGARTLAASKSLVSLSLIGCGIGDRAAQALVASRSIQCLDLSVNRIG
RDGAQALAGAPLVSLNLHNNEIGNEGARVLATSRTLTSLDVSNNGVGNAG
AEAFAGNTVLKQLSLAGGMISGDGAQ ALADNKSLTDLDLSNNRLGDAGAQ
ALADSESFVSLKLGGNEIGADGAEALARNVVLQSLNLSYNPIGFWGVNAL
GRAKLRKLDLCACAIDSDGASALARNTSLASLYLGSNRIGDDGARALAKN
STLTLLNLSGNNIHAVGAQALASNDSLITLDLSRNGIGDDGTAALACHPR
LTSLNLSRNQIGSTGAQQLAKSATLAELDLSENRIGPEGAEALARSTVLT
TLNVSYNAIGEAGARALAESVSLTSLD ARRNGIGEDGAKVLEANTRITGT
PQNPNFLAEDVPRPDVRWRD
> Q8XC43_ECO57
MSGTSGSSSDAALATRYAAEYFCKTWTAPGLNQAEGYKAISDLSHHYFRA
EGSSPPQSFL
> A1WKP8_VEREI
MSRLSLGCIADDFTGATDLANNLARAGMRVLQTIGVPGAAPDTEADAIVV
ALKSRTIAASVAVARSLAALQWLRAQGAQQIYFKYCSTFDSTAQGNIGPV
TEALMQALGSDFCIATP AFPDNQRTVFNGYLFVGERLLNESGMQNHPLTP
MTDPNLVRVLQAQTTHPVGLIDYRVVVQGEAAIRARMAALRADGVRIAVA
DAVCNADLLRLGAALKGMPLVTAGSGLAIGLPGNFGLAPGNEADRLPAAQ
GLQAIVSGSCSVATNQQVMDFIAAGKPALAIDPLRIASGMDMVAEALAWA
DAHIAHGPVLLYSSAEPSAVRAIQGRLGSDQAGALVEATLAAIACALVQR
GVRQLIVAGGETSGACVQ ALGIAQLRIGAQIDPGVPWCHARSPLAPEGLH
IALKSGNFGRSDFFRAAFARLPA
> gi|28867285|ref|NP|789904.1| type III effector HopPtoK [Pseudomonas syringae pv. tomato str. DC3000]
MNRISTSSVNSSFNYTAPTEEAQNRFASAPDNSPLVVTTTSIAQASEGLQ
RPGATLSMQAQRLRQLMGSPSEQCRRDTMLAKAFDAQRLNINTQA GSSNS
PHLNALNTLQQRHFKPAAGGLEIPVTSNSLLGGGRQVYQIGSSSRELSHR
PVNDQDRAPFRALERLHAELFRGGPIEFVPRGSNVLASNVRDVDMDEFDV
INSKDGCQGIGTTGLGPCIAVCARGMDREGLPVLGVYHHSGIGSPEDTMA
TLDQAMRDKGALQIKYSLVGGMIMPKEEEAGSYDDEQSFLALKGSYSIEG
ARLHVSEGEEDVHTGEDNSVNVLLMPDRVLYGRDTLYC
> NleB_Crod
MLSPLNVLQFNFRGETALSDSAPLQTVSFAGKDYSMEPIDEKTPILFQWF
EARPERYGKGEVPILNTKEHPYLSNIINAAKIENERVIGVLVDGDFTYEQ
RKEFLSLEDEHQNIKIIYRENVDFSMYDKKLSDIYLENIHEQESYPASER
DNYLLGLLREELKNIPYGKDSLIESYAEKRGHTWFDFFRNLAVLKGGGLF
TETGKTGCHNISPCGGCIYLDADMIITDKLGVLYAPDGIAVYVDCND RKS
LENGAIVVNRSNHPALLAGLDIMKSKVDAHPYYDGVGKGLKRHFNYSSLQ
DYNVFCNFIEFKHKNIIPNTSMYTNSSW
> gi|15723940|gb|AAL06387.1|AF311901|39 EspF [Citrobacter rodentium]

MLNGIGQAVSTLGRQLANVASRVGSVGGASFSVSPQAVRLTTIRIQSPFS
PAVSHINARTNFNVSGVTTSLTPSRPAPSPPTSGQSTGRSQSLPPIAQAL
KDHLAAFEASKSLEASSLKPARPAPQSPTNGQSTGRSQSLPPIAQALKDH
LAAFEASKSLEASNLKPARPAPQPPTNGQSTGRAQSLPPIAQALKDHLAA
FEASKSLEASSLKPARPAPQPPTSGQSTGRSQSLPPIAQALKDHLAAFEA
SKSLEASSLKPARPAPQPPTSGQSTGRAQSLPPIAQALKDHLAAYQQFKN
G
> gi|12329057|emb|CAC05788.1| IpaH4.5, member of th e IpaH family, probably secreted by the Mxi -Spa secretion machinery, function unknown [Shigella flexneri]
MKPINNHSFFRSLCGLSCISRLSVEEQCTRDYHRIWDDWAREGTTTENRI
QAVRLLKICLDTREPVLNLSLLKLRSLPPLPLHIRELNISNNELISLPEN
SPLLTELHVNGNNLNILPTLPSQLIKLNISFNRNLSCLPSLPPYLQSL SA
RFNSLETLPELPSTLTILRIEGNRLTVLPELPHRLQELFVSGNRLQELPE
FPQSLKYLKVGENQLRRLSRLPQELLALDVSNNLLTSLPENIITLPICTN
VNISGNPLSTHVLQSLQRLTSSPDYHGPQIYFSMSDGQQNTLHRPLADAV
TAWFPENKQSDVSQIWHAFEHEEHANTFSAFLDRLSDTVSARNTSGFREQ
VAAWLEKLSASAELRQQSFAVAADATESCEDRVALTWNNLRKTLLVHQA S
EGLFDNDTGALLSLGREMFRLEILEDIARDKVRTLHFVDEIEVYLAFQTM
LAEKLQLSTAVKEMRFYGVSGVTANDLRTAEAMVRSREENEFTDWFSLWG
PWHAVLKRTEADRWAQAEEQKYEMLENEYSQRVADRLKASGLSGDADAER
EAGAQVMRETEQQIYRQLTDEVLA
> gi|17427254|emb|CAD13773.1| HYPOTHETICAL/UNKNOWN PROTEIN [Ralstonia solanacearum]
MAMKAVTRSPAYPSGLRDLAATAKTDATTYPLRPHNSMPPGTAGEQKPPG
KSSAGGPLAGLSKTGLGVVLPERALHLPAMESLAAPARLLPRTRPASADA
VRRALLTAFWGSHGAPEDYAEQQLLNQLRSHKPAVPMPCILFTDPNKDPD
DVVAFTLAKPLQAFRLVKMAHAVATLGERDVRTRRAGVARGAFDRLDLQE
VRVTVGLDYAIKPEHAKDHAKFLDQGEALHVKLKPEAASEDSVA ALRESL
TQAAAPVTLVVIAGMTDPCALVTAEPGLVKQKVGRVVIMGGVRPDKDEDG
YVQPDDRAYNNTTDFAAACRFYRRVQELGIPLRIVTREAAYRAAVPRTFY
EDMAQSGHVVGQYLKDVQKHALNTLWDGIDQGRIAKLDKHWFFNTFIARE
IGQGDAAAWAARNHTFDAIWEQVVRLNLYDPMTLLAAVPASAQMLFRPKA
VQDAGRGLVEVIGADEVHCPSDARQLMSGLVKMALANTPGTR
> Q8XQE6_RALSO
MNIRKYLPVCFTGRTVEDDTLSSTPAQTEPRKKSGKRSTGMLTGLISRSR
KASEPDLAVRRKPLAEPGSPIKAAGTRESGNSLQQLFDADPNVASTAGAA
QVSQPPAGHMSDLAPPAGSVAPSPFSGARTWATASAAAIHEITPVPVSPA
IEVEAPAGAKPAEAAAASGAVAGGGVPLRQEPLFDLAFKKGKIRVKARHP
AQDRKQAHQLERLLNLRDNRVTSLTWNQATGGYLIDYQK HGVRYWVGMHG
KSLPAIVLPAHPGMKQGCSPPPIGLLANLSGSPDGALWREHHGQLYQWDT
QQAQWKPHALPGKMRAPITLLGRQLDGEAWARAGSMLLKLGAGGIRAYEV
AGLERFSAVRMDAAGRPLALDGEGRLCRPDSPSAQPRPIQLQLADGRPAF
EPVQLGKPDKPIQLARARDFVLAPDGHTLFARDQQGHLYQGDLHAADAAS
GEIKARRVGVPVRMPGSADGWGVETLATSPGPSDKGAALH AVFRSSEGQR
VTAAWDGQQWQPQWHVEQPLLLMSERGLQAPAMRTVCTYNDGAALGISAA
GQASQKDGAGHWRPLLQADGTPLADLRDLKLGPLGLADAKPVYALQSTPA
GGSQLLALELGGNMARLPARPGVAVAAVPAQRYVSQVSVLAASAAPIRDF
AVDGAGVAYHLTDDHALMRTPPGGQPQHLPAPPGQARLEQIAVSSDQHQV
FALAHSPSQDGGAAPQLSLLRYDHASASWADCQAGLSVLDP ETVRLGISR
LGTLQLTTGGADGEQRTHRVVPPLGAQKRYRLLATGTTEPTVSQALTGNR
PKRVRIPGTGVVATLHRTAGGVTERVQSNFRTAVGHVERVLAMPKGAVNR
AMEHLRGRRDMAEEYDDMKATHLELEPLLHHRHWPLPPTVAAGPAVAPVV
PEATAQACAALRGDAIDKMLGALAQIGVAARVLGPDLTLDAAAGRRKQRR
PAKAKPDPDDLLPKMHSWLDEFVRRAEAQAKPSPEAGAKAPA PVFDPARL
GDLHRIVELMHTLSEHGVKLPAPDLTGQRDTRDRFAILTGAVGRHLLTLD
LATRLVTLPQTVAVIGSDSAVEVLRQSVETDKVLRLARFGFSNWHDAEAF
WETAQTFKKEVMKPGSPFNRKFAESHAVRDASSPAEMALKFAEAMKGLSN
RSTLFGIESKGASAGVTSAGVVLPKKRVGAFGFAVLGVDRTTMVGVERTA
DKLAEGPLVAFFVRQSNKGLTMGGGMGMDFKPLRKVLGFRLQG GAQASAS
VMHGKGAAMLVAPDNIDEFARRLFDPASHDPGRLLELGLNQGAIGLDMIE
QQLNLSANAGGLGGYADKIPGFGPAHRHGDGSSTSAFQQTGLQRGFVGAN
VNWGVRDFFLKLQHAWEPISGYEYQGGRGWSANAFASLVQQGGLLPHVSD

AFTLVLRSLNITLLGASVELSGVESFKRTLDWKTAARVTPQEWSQLAALA
REVFPSQAIGHFDGPHLKAIIATTLQAAKSTWAARTEHERASFV DRAEQL
LLQDQLASGGRAMLLPGAKIEFNIPNFRSLVDTRKNSKAHRSLGALMEAA
ERAREAVPGLADAMRAMSERDGVNDVRFVFQMDPSYINAVNRLMLEGKLS
WAEFNTMARTVPAPYRLTEICAKDSDSNRSAFTLNPLPLLAFNDSAEVSR
SLFAAEVHLRYGLNGRLLGADLLPGAQRAVAGQKVLQPFVDAGVQPVPAA
GGAAPAQAPDLPRLQRSQSLPSERPAGPALKARSLERSKSLE
> Q8XC86_ECO57
MNTIDNTQVTMVNSASESTTGASSAVAASALSIDSSLLTDGKVDICKLML
EIQKLLGKMVTLLQDYQQKQLAQSYQIQQAVFESQNKAIEEKKAAATAAL
VGGIISSALGILGSFAAMNNAAKGAGEIAEKASSASSKAAGAASEVANKA
LVKATESVADVAEEASSAMQKAMATTTKAASRASGVADDVAKASDFAEDL
ADAAEKTSRINKLLNSVDKLTNTTAFVAVTSLAEGTKTL PTTISESVKST
HEVNEQRAKSLENFQQGNLELYKQDVRRTQDDITTRLRDITSAVRDLLEV
QNRMGQSGRLAG
> SIPA_SALEN
MVTSVRTQPPVIMPGMQTEIKTQATNLAANLSAVRESATTTLSGEIKGPQ
LEDFPALIKQASLDALFKCGKDAEALKEVFTNSNNVAGKKAIMEFAGLFR
SALNATSDSPEAKTLLMKVGAEYTAQIIKDGLKEKSAFGPWLPETKKAEA
KLENLEKQLLDIIK NNTGGELSKLSTNLVMQEVMPYIASCIEHNFGCTLD
PLTRSNLTHLVDKAAAKAVEALDMCHQKLTQEQGTSVGREARHLEMQTLI
PLLLRNVFAQIPADKLPDPKIPEPAAGPVPDGGKKAEPTGININININIDSS
NHSVDNSKHINNSRSHVDNSQRHIDNSNHDNSRKTIDNSRTFIDNSQRNG
ESHHSTNSSNVSHSHSRVDSTTHQTETAHSASTGAIDHGIAGKIDVTAHA
TAEAVTNASSESKDG KVVTSEKGTTGETTSFDEVDGVTSKSIIGKPVQAT
VHGVDDNKQQSQTAEIVNVKPLASQLAGVENVKTDTLQSDTTVITGNKAG
TTDNDNSQTDKTGPFSGLKFKQNSFLSTVPSVTNMHSMHFDARETFLGVI
RKALEPDTSTPFPVRRAFDGLRAEILPNDTIKSAALKAQCSDIDKHPELK
AKMETLKEVITHHPQKEKLAEIALQFAREAGLTRLKGETDYVLSNVLDGL
IGDGSWRAGPAYESYL NKPGVDRVITTVDGLHMQR
> gi|34498032|ref|NP|902247.1| secretion system effector [Chromobacterium violaceum ATCC 12472]
MPGAGLPADWAEKEEGKSGAARKVGDAVKMVSRPLAEVQSQQLGQLASPR
AAKEPVSRQQAADALGRIMGTIDQASGSDGAAKVPAFSLSEIEKIPMDSM
IMASTLMTSQMLGDTAAVKSKALDIMSTKQEALRKKEV ENIREQMNKAIE
QQDKAKKAGILSVVFDWVVAAVEVVTGVAKMIGGVMTGNVMQAAGGAMDL
VAGMAGLVKAMANTMALIDPENAEKYQKVADIAGKIQLSFEIAGAVVDVT
SAARNLLVTKMIPKVAGKVLKEGAEQMVSTAIKKGTAGAAKNAANAVGKQ
VADQVATQVAQALGKAAVDAAKATTKKTVEKFAQKFTNQMLERFTHEAIE
KMVSKSVEKVIKKAVKEGVELTAQEVTKRVVNQVFADVV KATIKATLKAP
ALVVSSTVRGVAAGAKEIMVGEIEKQRAKLQKEIDLLILDQQWLQSFFEF
YEQEKKEVIKKTRELLDSKSQVIDDGLQAMSQAASTQAQIASSMV
> gi|58579689|ref|YP|198705.1| HrpF protein [Xanthomonas oryzae pv. oryzae KACC10331]
MRFFRQNSSSARVHVAQMGNIRLHRMGGSVRPYKYKLIRRCSANSVIREA
AYMSLNMLST GSNPSQLLGTSSNESSSSELFGSDSSNDGSDLPSTMDTIF
QQIYLLLAALQANTQTSASGDTPANTASGDADTQMSASDWQATQPIEKRT
SWPSLGYDFDPKNIKGKDAPPALEGSTVTWNDGTLTKSELQIVSTLNAHK
DQMPIEYKNLDDKINDPSTPPDLKAALQGLKQDPRLFFAIGSQGDGKCGG
KVSAQDLWDFSDSHPQVKDLGGKNDEFNPKDIKGSNPPQAAEGSTVTWND
GQLNQSELEIV SVLDRHKDQVDSLSFDQLDAKINDPSTQPDLKKALKGLQ
KDPRLFFAIGSQKDGKCGGKIKAQDLTDFSYYHPQIAEYNDKKAKSYTQN
YIASDSPDKTKASVMTKSDALREMYRYSDYLPGNLSEDEFAKIVDGDSKT
GKCPPQLIAAAQYFRDHPDEWKEFSGDAGTMSTPDFLQKSTSEMHLTADE
QKTLDTINSHQDAFYGDGKELTRDKLDAISKDNKADPAVKEAATQLASDP
LLFGLLNNSITG YKKPHHFFGGGHVVDSGKISQNDFRQFYDHMSAANKTV
NTPATHEASSPDQQKAVADMLMGKDDPPAIKKPKKDVGTFQQGLHEFLKW
DSKILDWMSVGLSALNGIPVIGEIADAAAIALESEAQAAQVVDTAIQGGD
MSLALKLAGINMAGAVVGAVGGPTARIGAKGAAKGVAEVAAKEAAEGAAK
GTAKGAAKGAGKTAAERPSAAAFAKGYVAGSTISKSTEILKKPVMAGLHY
EEYQLDKQKDGEI HQKLDNAGGAPVGRQIIPTGIADNFEGDVRQNLRNVR
IRRR
> Q8XR46_RALSO

MERISTNAISKTVARSDLSNGSSAPPRKKNGSPPRRAHAPLLPLADRSLT
AISKPNETLGSRLAPVPQRAVHASAVPSAAGNWNPLSRLKPEELAGIPET
VVEKLGKDLHDLYEDVQSKGLNYVPVFGYLSLRYKNFHELGKRSQDEVRE
GKDWVPASLDNHALDFVMSTQYRGHHQHPGVVAGLSQSEGSAMDG VILKL
PVGNAEELLARVIRRELLDENDLIHPVPATPPAGTTGLAGSATAGPATPL
SPKKPRSNLMYSSAVRPVTLPNGAKVRALVFVTNPDSAKSLASVFKDREG
VSPQRLAYLMTSTSKGDLGGPAIDYWKRFVETCETAKTAVPPIVSQAIRL
ASDWPDAFTGEPAAPDDMPRQHQEAAWFRAMAGAAAPRRVWHERKPEPSP
DGASAGGTLGTQPKPD
> gi|53722549|ref|YP|111534.1 | hypothetical protein BPSS1528 [Burkholderia pseudomallei K96243]
MPPSIHRTSSINSTPAVTAAGARRASGPGGVLSADVARVASARRHTMPEI
GARRGVDGDRAAPAPRESFRRRLETVSSRAPTPPPEADSRAANARTTGAA
DGAGATGAAAGPSHGATSLDAARAYLAELAGDRGGALTTLVAQLNGHDRR
ALDHLALTVHALHLSVDDASHAKTFAGIGDALASFL AAAARANAKPGSTP
RDLCVEGNKGYRKLCECVGALLRSDGIANALFGRARERGDAAADTLVSER
LGGRMDGHVRTNGMVNRHGGDAQRRMADAAQRFALGARHATNLVDMVGTA
LELLGRTDQLLNDVSLKRPAAEPGGPRAPGGGRAAPAGPASGAAQQPAAP
VVVNNHNENNVNVNLDGLERLVGDLGKMMGALLERMGAYDPSHTRPAAPD
ANGPAPVRTGGHTTVVKSPVSVARTSNNGAPGSPAAD GTRGDAPMPPTTP
ALPATPATPTTPPAGGPSMDERTPATNTLERGAGNGGASPTGAWRRGDDG
GWRMARGNPVVRTTEGATRIDPFSRVGARAASGAGAARAWRDAAADATQR
SLASGTSGGAADAIAPRAGAGAAAGGDAARVARRMPDVRAGGLERDAPGE
RAAGPNGSNGSAVGQPIAGHAPAGGPDAADFSDASGTGGTGARAPGIPPA
TGSWSRDPASGRWLRAGGGVPLRSTEGFGRIDPFAGVQ RNASATGASAVS
SPSFASASMSVGSTGALPAGSSIASPDTSGIAPAVASGGALDVASNVASN
VTSNVTSNVASGVTSGVTSGVAPDGTAWTRPSALAPRNIDPRRMNRAQLE
RAGLYPLVDDALPPSSQSAPADAGDAGDAAARRASRPDTGAPQPGAAKTF
PSVAPAHRIVTTEGAARVGWNDSIANGTKR
> Q8X2D5_ECO57
MINNVSSLFPTVNRNITAVYKKSSFSVSPQKITLNPVKISSPFS PSSSSI
SATTLFRAPNAHSASFHRQSTAESSLHQQLPNVRQRLIQHLAEHGIKPAR
SMAEHIPPAPNWPAPPPPVQNEQSRPLPDVAQRLVQHLAEHGIQPARNMA
EHIPPAPNWPAPPLPVQNEQSRPLPDVAQRLVQHLAEHGIQPARSMAEHI
PPAPNWPAPPPPVQNEQSRPLPDVAQRLMQHLAEHGIQPARNMAEHIPPA
PNWPAPTPPVQNEQSRPLPDVAQRLMQHLAEHGIQPARNMAEHIP PAPNW
PAPTPPVQNEQSRPLPDVAQRLMQHLAEHGINTSKRS
> hopAC::ISPsy5 Pto DC3000|Genbank:
MRPIVAGLQVVAQTSAPQARNGKPVMTQKLPPLAITPTPLTPADTWPAAS
AALKRLDELRTLLASELETQPGPSEALLTALGGSDASERELEIFSLLQQI
DDYWTAPGETAESRRDRLVPALQRALRDEASVRIHERDLESGYLACLPDS
SGQPQSPALTYASLHVQLHDDE HVEMAGVLAISQEQERTLLMLPGLGIIG
FATQALMLATVARWLNTPTLQDALLNTLEQDHQDPLFTISQEADLYLEPF
TAADLQLQAVTTAPFMHALDRLLNKQRNDIRHACERPDIEDREARRGLIQ
KAIDMRGLLGPSYMLELRELTNRQRQYHRDMPDWLKIASEADLQTYADHL
RRYDDAHAAMLSVLGSAASPEQFAETHLRTRLANDLGHDLHPRALTIATR
RTLPSTDKTYLMTRSLVELALYG LHPDDEVAGSDFLEQTVITLNDKPLDA
AYAPLNPAYLAGLIDELDLRAVFGEFQQAAYQQEHSQQLLRALARTRLTA
QGWAAKMQGHIQPEDFAIVAQLASPTQGASDPALCVQQIRLNGHNVMARL
LAFRKQDAQGRTERLIMVAMDAPGQPFKAFDTETQLLHEVVGWSASPSMT
AYLLDQVEVSARPELEQKLAALALKPQPEKDFLQFIDHPDCETALRGFTD
EQTRVMLSEQARHTPDWYLRATRA QRRELQALEEAIGGALGHYQAQPHTQ
VQPFKNYVHQRASQQIAQLLNVPAANVDPDLIVITSERESLTYTDMLLNG
YNDSIDPVRTSADTQATFSGPDGIDLSALSPAAVAGSVRGRWLADDYIAL
IRSTLLNPESEGYAYRRQINTLITQLQMKAAALRSVLKGHIEAAQYAWLK
QSLDNAHLNDPASREQYPLYPLQVHVDKPLIASGLNGVDQLVIPSPLLTH
VETVQGCLVILPTQIRHAALLYTPQ APDGIEFRLFSAFVRSLDSDGMIDY
YKDRCRIKARRTLSFFLRDMQQGKANKPPVIPKAFISDFAETCFNRPLER
RLRDVEETTTGRNDMLNQLIWVSVEIIATALTLPFPPASFAVGSLLSLHD
SGRAIKALTEGEHGRATAYMLSALFNGIGAGSDLLVGLKGFGGVLHHLEH
GRESXXXXXwcwllkengstasgvsqwlemwgswigyresapvlrsfqrq
sslphyedlypvelqeqvflisrpna nghaavfqvpnrssappkatgqfa
arqaggawqpllpspnlaprapsglrielavnlslhdvprivaghaegvc

vvngrhyvelsgrtfqvhydthlrgwqivdpenpfaffgqqpvrldeqgq
wqlvarrrlrgggvgdsshahlpeetpgsstgsipsdyempaamqagldv
vlsnkpydptgigmesyfesyfvdlrqsfvarreklyedartffagfspp
pkpqlpplappvaidtliehvfaqgng lvlseapksvaskrllllnmpll
aeqrvkilyiehlltdkhlsklaryrqlgkksrsgshelkhylhdlnrgt
lnnsstdydyyhlikaahrygievrpfsssisypfldhpvlsaandttav
qkmsnffghtlissdvasaptkrwvalldqklatthdgvlgiaemqgvvs
vhvrdipagrptritkgtgelpregtqarcdftiafsdptlivpqaphph
gtklddmllrelrgqsagaggerwagqy gfirdedgawrwiapedwpads
pmtaiqqsltdpvyempldtrttlhtlanferrgldmeyffeesqyetvr
nvfalhrkklqqdaalisavqlpprptmpavnprtttaqlfetlyqhtdg
ivigeshfsvaskkmiidnlpllsqqnvrtlymehlltdlhqadldrffe
tgqmsktllhdlkvldrghrtdpdkvytfeqlvikaqqhgmevraidcaa
syhlsgldndgsitrqqmmnyfasrtlrr hqdvmgshkwialvgnshsnv
yqgvvpgiaeleggiglrvidvapgqskgvmhdlgelvsadisrtkvhik
gdyrveieiprakdairppqpvtleqrlarpglflveesegnlltivhra
rdtwihrtpvlvnaegklylervrwprihlkpfddmdalvaaleemnltr
vg
> gi|15723927|gb|AAL06374.1|AF311901|26 unknown [Citrobacter rodentium]
MNEQFLTDLAHTFGITLEEPTDALAFHDNDGHEWILECAPQSEILFFYCY
LSNTEPEQMNKILEINSNREMLGMFLLSLKENKVLLNIAFPANKIDITEF
TNLMENGYIIKKEIINSFLL
> Q7ADX5_ECO57
MPVTTLSIPSISQLSPARVQSLQDAARLESGIRISIGSGQYSVHYVQLLD
GFSVEPVRGGLLDRLLGREHRMDRRAVALERQLNGGVDFLSSVNNYFQSV
MAEHRENKTGNKILMEKINSCV FGTDSNHFSCPESFLTCPITLDTPETGV
FMRNSRGAEICSLYDKDALVQLVETGGTHPLSREPITESMIMRKDECHFD
AKREAFCCK
> STM1398 sseB translocation machinery component 1483934:1484524 forward
MW:21495
MSSGNILWGSQNPIVFKNSFGVSNADTGSQDDLSQQNPFAEGYGVLLILL
MVIQAIANNKFIEVQKNAERARNTQEKSNEMDE VIAKAAKGDAKTKEEVP
EDVIKYMRDNGILIDGMTIDDYMAKYGDHGKLDKGGLQAIKAALDNDANR
NTDLMSQGQITIQKMSQELNAVLTQLTGLISKWGEISSMIAQKTYS
> gi|28867731|ref|NP|790350.1| type III effector HoptoS2 [Pseudomonas
syringae pv. tomato str. DC3000]
MNINRQLPVSGSERLLTPDVGVSRQACSERHYSTGQD RHDFYRFAARLHV
DAQCFGLSIDDLMDKFSDKHFRAEHPEYRDVYPEECSAIYMHTAQDYSSH
LVRGEIGTPLYREVNNYLRLQHENSGREAEIDNHDEKLSPHIKMLSSALN
RLMDVAAFRGTVYRGIRGDLDTIARLYHLFDTGGRYVEPAFMSTTRIKDS
AQVFEPGTPNNIAFQISLKRGADISGSSQAPSEEEIMLPMMSEFVIEHAS
ALSEGKHLFVLSQI
> avrB2 Pph race 6-1448A|Genbank: AAZ37967
MGNVCFRPSRSHVSQEFSQSEFSAASPVRTSERPSDASLDAGLESSSACH
RSGLRGPAKHSMLSLEEIGLVGAARWPDDAPGLNISNKSNTQENKRYCES
LYQAARIAGGSIASGRVTSFDGLWRNATKWRLSRILSGDASKIDFATVRM
PNTRFVTSLRRPYHSVIERVRNHSDANSEIYEGEYLGGIETKVYRQHGTI
SSTTIPMTIVSAVADDDDIHERLKSLPKNERR HLKDLMAASHPNMITHTD
AVYLPMIKDHLESLYLQAIDPSLEQHEALELIARIHWWAASAAPDRRGSA
AKAEFAARSIAFAHGIELPPFEHGAVPDIEAMLRSEEQFVEDYPNLFERP
PQ
> NleA_Crod
MNIQPNIHSGITTQNNQQHHHAEQVPVSSSIPRSDLPPNCEAGFVVHIPE
DIQQHVPECGETTALLSLIKDEGLLSGLDKYLAPHLEEGSLGKKALDTFG
LFNVTQMALEIPSSVPGI SGKYGVQMNIVKPDIHPTTGNYFLQLFPLHDE
IGFNFKDLPGPLKNALTNSSISATASTVAPTPNDPMPWFGLTAQVVRNHG
VELPIVKTENGWKLVGETPLTPDGPKANYTEEWVIRPGEADFKYGTSPLQ
ATLGLEFGAHFKWDLDNPNTKYAILTNAAANAIGAAGGFAVSKVPGIDPM
LSPHVGAMLGQAAGHAVQCNTPGLKPDTILWWAGATFGAADLNKAEFDKV
RFTDYPRIWFHAREGALFP NKQDIARVTGADIKAMEEGVPVGHQHPKPED
VVIDIEGGNSPHHNPSNYVDTFEIIQETRV
> hopAH2-1 Pto DC3000|Genbank: AAO56770
MNINSIRSYDLSMAAPEKSAVTNKATLPVNAGNNDAPTTRQSKLDTYTAS

HKYDSSGRPAEAPETAPVYTKALLNRNAQLQKSAAPSDTAPAGQQQVATA
VTKNALQSNAVTVEEKVSGPTPNASNADTGSHGGANVDPGDIATPAAATK
MIQDLKGIGGGTLRIQMTHDQIQDTKEMDKLTALVNEGDKQGVKVQFTFR
DNANGGGGSVLTGDKLNQASTDISNVVKKFGGHSSFVLDTFNEGGTSATQ
GWADMESTLIKSARNAGYKGSIVVEDSNWGGGLTAGPESGLVKYADQLKA
ANGKGNPGLIGSIHEYASGADASARLGNEIKALQNAGYKPQIGEVGNANW
LGGDKFEERDGATKAVRDNLAALKAAGADILPWKDQFQDGKLRHHVGFSK
SDQY
> AvrA_Psyr
MWNVSKSSNNLGAYKLPLEAQTPPEKISPFDAMSAAQPEGKAPHDQLQND
QYPIQQAEDRGRHLVEQAEIQAHVQHCHSKAPEIGDATKTQSVSEKLGTA
KNSSCDASQILIGSKNDDFHKNKAGSNGDINKSSDPSALRCSLSPAPRRV
PKSKKSYGAATIGGKVYHPHEKTDSTIADFLSRSLSNNAYRSERHLRKRA
LAYLNHISAEKEITSNACFAMKDVNSFAHKQSEWLC HLERSLWRDEPALQ
FHDRQQLGNEVLGLKKPDDQSPYFKPRAWKISDEAASAFAMMLKGESGPF
TQDQVKVGFEICQEGELLAGRLKIQPRMAFRLKNRHDANRSGTHSVKSLS
GLDLSADVGTDIREFFQVPVMSGTSGTSSDVVIAARYAAMHAGLKWSAPE
LTIDQAKHALIDLSMDFFRRNGPAVVMALRMNSLRKNQGLPYKEVDRCEV
FTHSYAEIHGAISLTIDGVDPADKVEVKNRLYGYTLD AKATLMKIADRSI
RRGVRSKVDIRSTSTSLQTPQLRRVLEKKKIVQKVAELYSEMGKAGNSAT
LKEAITKSSVKELLVNDKPVVSRDYALGEPLMVRSLRFSHDHEATSSFGS
AGKTPAKREVDTLCDNSTAFDIVMTPFSVINAKAKGDTISEMKVPHRPKW
KGLPSVLYKVTASVDLPEYAVARPGFGDIHSFNSNKAFSSEFSSVRNSLS
HAEKMGFIENSLKPYIKHDPDRESFDFKHSIDELADAQ CMLQSRKPNSTL
RHNEYCAKLELWDAKAIEVGMSRPVAVATLIEFNLEMLSAARYIEDEGYD
GKLITNFLERQLSWFGQNAALNKEVTLKKLWGLPFDERKAVAEKVCEALR
QGVSLCVYEKNVEGSRIRELSLLNFNAYDIMRGIELFLSSKLLQPPTGAG
PTVKSRL
> A1HAK7_BURPI
MSAAPTSRPVLGCIADDFTGATDLANMLVRAGMRTVQTIGVPQAGGEAIA
ADADAIVVALKSRTIA AADAVAQSLSALRWLRAQGCSRFFFKYCSTFDST
DAGNIGPVAEALMAELGTDFTLACPAFPENGRTIFRGHLFVGDVLLNASG
MEHHPLTPMRDPNLVPVLARQSASKVGLLRYDTIMRGAQAARDRAQQLRA
DGVKLAIADAISNDDLIVLGEAFADLPLLTGGSGLALGLPAQYRRAGLIT
AHGNTAQLPIVADSAIVLSGSCSRATNAQVAHWRASRPAFQVDPLALAEG
KPVVVEALDFVHRHAGQ TVLVYATSAPEQVARVQQTLGVERAGKLVEDAL
GRIARTLHADGVRRFVVAGGETSGAVVQALDVRALRIGPQIDPGVPWTAT
IDSDPLALALKSGNFGTVDFFEKALAQLAHATTEPIA
> CP0127 ipaC IpaC, secreted by the Mxi -Spa secretion machinery, required for entry into epithelial cells 110137:111228 reverse MW:38 861
MEIQNTKPTQILYTDISTKQTQSSSETQKSQNYQQIAAHIPLNVGKNPVL
TTTLNDDQLLKLSEQVQHDSEIIARLTDKKMKDRSEMSHTLTPENTLDIS
SLSSNAVSLIISVAVLLSALRTAETKLGSQLSLIAFDATKSAAENIVRQG
LAALSSSITGAVTQVGITGIGAKKTHSGISDQKGALRKNLATAQSLEKEL
AGSKLGLNKQIDTNITSPQTNSSTKFLGKNKLAPDNISLSTEHKTSLS SP
DISLQDKIDTQRRTYELNTLSAQQKQNIGRATMETSAVAGNISTSGGRYA
SALEEEEQLISQASSKQAEEASQVSKEASQATNQLIQKLLNIIDSINQSK
NSTASQIAGNIRA
> avrB3 Psy W4N 15|Genbank: AAN85189
MGCVSSKASVISSDSFRASYTNSPGAFRAESSSVHNRARTPRYGELQGPQ
ASRLMPYQQALIGVARWPDSHFNRDDAPHQMEYGQSFYNKSRALGGSVA N
GEIGSFQELWDEARDWRASRAGADADVFLSPRDPNSYREYATPLAEQYSY
IKDRFANRKDGEVGSPADFLPKSKTFRISGKIDGEQIPLTRITVSKDRHA
DRMADPYPRLRNQGHGDLGEPNLMSHTSAEYVPQIMSHVESLHQSATDPS
VSDSHALKTLAEMHWWMAHAMPDKRGSAAKTELCVRSIAQARGMDLPPMR
LGIVPDLEALTMPLKDFVKSYQGFFENN
> gi|1330244|gb|AAB00 675.1| putative
MDPIRSRTPSPARELLPGPQPDGVQPTADRGVSPPAGGPLDGLPARRTMS
RTRLPSPPAPSPAFSAGSFSDLLRQFDPSLFNTSLFDSLPPFGAHHTEAA
TGEWDEVQSGLRAADAPPPTMRVAVTAARPPRAKPAPRRRAAQPSDASPA
AQVDLRTLGYSQQQQEKIKPKVRSTVAQHHEALVGHGFTHAHIVALSQHP
AALGTVAVKYQDMIAALPEATHEAIVGVGKQWSGAR ALEALLTVAGELRG
PPLQLDTGQLLKIAKRGGVTAVEAVHAWRNALTGAPLNLTPEQVVAIASH

DGGKQALETVQRLLPVLCQAHGLTPEQVVAIASNIGGKQALETVQALLPV
LCQAHGLTPEQVVAIASNGGGKQALETVQRLLPVLCQAHGLTPEQVVAIA
SHDGGKQALETVQRLLPVLCQAHGLTPEQVVAIASHDGGKQALETVQRLL
PVLCQAHGLTPEQVVAIASNIGGKQALETVQRLLPVL CQAHGLPPEQVVA
IASHDGGKQALETVQRLLPVLCQAHGLTLDQVVAIASNIGGKQALETVQR
LLPVLCQAHGLTPQQVVAIASNSGGKQALETVQRLLPVLCQAHGLTPEQV
VAIASHDGGKQALETVQRLLPVLCQAHGLTPEQVVAIASHDGGKQALETV
QRLLPVLCQAHGLTPEQVVAIASHDGGKQALETVQRLLPVLCQAHGLTPA
QVVAIASNNGGKQALETVQRLLPVLCQAHGLTPEQVVA IASNGGGRPALE
SIVAQLSRPDPALAALTNDHLVALACLGGRPALDAVKKGLPHAPALIKRT
NRRIPERTSHRVADHAQVVRVLGFFQCHSHPAQAFDDAMTQFGMSRHGLV
QLFRRVGVTELEARSGTLPPASQRWDRILQASGMKRAKPSPTSTQTPDQA
SLHAFADSLERDLDAPSPMHEGDQTRASSRKRSRSDRAVTGPSAQQSFEV
RVPEQRDALHLPLSWRVKRPRTSIGGGLPDPGTPTAADL AASSTVMREQD
EDPFAGAADDFPAFNEEELAWLMELLPQ
> Q84H14_PHOLU
MSPISGINHQRIFPTQQLDTTAVNQPQGELSGKSLKVSSPRPGLLERLSA
TIQRNLPGHSMLDRQLTTDGKKNQESRFSFSMIKDRIVHFAVSTKLGSVR
DSASKHGGEVTFKFAQTKGAFLDQIMKHQDTSGGVCESISAHWISAHAKG
ESVFNQLYVGGQKGQFHIDSLVSIKQLQMDGLDPYAEQSQITESWLR ENG
IQPRSPLEVSGETGSKGTKDLLNAILDTGDKGSGYKKISFEGQMAGHTVA
AYVDDQKGVTFFDPNFGEFSFPDKTSFSNWFSQDFWSKSMYNKEIGLGQN
FYVSNFEPKTR
> gi|53722631|ref|YP|111616.1| hypothetical protein BPSS1608 [Burkholderia pseudomallei K96243]
MRGRARQSVRFPMTEEPPMNAFRPPELIETERLRMRPLTEGD APEAFDAL
FGDRATTLDLPWPVHRSVDESRASIRRLAEGWRSGAFFSWGLFEKRGGRL
IGTLEMHGRLPRVEIGLLTSFAPGRVRTRAWSEMLHRFLGWLIAQPGVYR
IEAFCSVTGKAAPLMPHFGFVCEATVRNYEARPNRGMSAGDSYLFAITKA
PQALRGAVEDEARAPSRQAA
> hopAX1 Psy 61|Genbank: AY349161
MNPIRNSPSFIPPAHSPATQAPSPGTALHSAVVSRDSKA VAQLRNEGARA
NKLDAQGHSPLDVLDTMRDIDERSRSSLRMALLQSLNPTAQLGYTKPEAL
HGTPWGLEILQSGALRGGVNDAKGGTQSLEGKVFFSDRTRESASAETTRA
DFRSKPRVYARGEGMHPSNAYSRAQQHRMAQVILHALDNGRSLSTNELAP
SIEVSSPETLYIEGAAWLQRLLHGGYINKLGGLPFINAPLGEHLDSLRLP
GSIELRVDGQVKKLQGEELNRFYHQAASELQRSLENGKAP YLGLLNKGAI
VPLVFGFEKINNLSTHEIKLRSKTTQHSYQDTEHPLAGSPENGGKLKEVE
VRSLGDFATLCLGCAGQGALNCLPT
> gi|19749309|gb|AAG45730.2|AF229441|1 Y4yB [Sinorhizobium fredii]
MEHCPLSAAQLIPTEEVNPDRVDALQAQILQTGSWTVPITAEKDALFVID
DYHRLTAAHRLRLTRMPVLLLDNDSVRVESWRPGGNITPAEIFAMARSGR
KFPYKTTRHVFAHGLPTCDVPLELLSSPTPMDIAPVFSAGAL
> gi|22658444|gb|AAN01357.1| avirulence protein [Xanthomonas oryzae pv. oryzae]
MRPGKLCRDHRPLVWRRPCKEVCLMDPIRSRTPSPARELLPGPQPDRVQP
TADRGGAPPAGGPLDGLPARRTMSRTRLPSPPAPSPAFSAGSFSDLLRQF
DPSLLDTSLLDSMPAVGTPHTAAAPAEWDE AQSGLRAADDPPPTVRVAVT
AARPPRAKPAPRRRAAQPSDASPAAQVDLRTLGYSQQQQEKIKPKVRSTV
ARHHEALVGHGFTHAHIVALSQHPAALGTVAVTYQDIIRALPEATHEDIV
GVGKQWSGARALEALLTEAGELRGPPLQLDTGQLLKIAKRGGVTAVKAVH
AWRNALTGAPLNLTPAQVVAIASHDGGNQALETVQRLLPVLCQDHGLTPA
QVVAIASHDGGKQALETVQRLLPVLCQDHGL TPDQVVAIASHDGGKQALE
TVQRLLPVLCQDHGLTPAQAVAIASNGGGKQALETVQRLLPVLCQDHGLT
PDQVVAIASNGGKQALETVQRLLPVLCQDHGLTPDQVVAIASNNGGKQAL
ETVQRLLPVLCQDHGLTPDQVVAIASHDGGKQALETVQRLLPVLCQDHGL
TPDQVVAIASHDGGKQALETVQRLLPVLCQDHGLTPDQVVAIASNGGGKQ
ALESIVAQLSRPDPALAALTNDHLVALACLGG RPALDAVKKGLPHAPELI
RRVNRRIGERTSHRVADYAQVVRVLEFFQCHSHPAQAFDDAMTQFGMSRH
GLVQLFRRVGVTEFEARCGTLPPASQRWDRILQASGMKRAKPSPTSAQTP
DQASLHAFADSLERDLDAPSPMHEGDQTRASSRKRSRSDRAVTGPSTQQS
FEVRVPEQRDALHLPLSWRVKRPRTRIGGGLPDPGTPIAADLAASSTVMW
EQDAAPFAGAADDFPAFNEEELAWLMELLPQSG SVGGTI

> gi|16764411|ref|NP|460026.1| secreted effector protein [Phage Gifsy -2]
MPFHIGSGCLPAIISNRRIYRIAWSDTPPEMSSWEKMKEFFCSTHQAEAL
ECIWTICHPPAGTTREDVVSRFELLRTLAYDGWEENIHSGLHGENYFCIL
DEDSQEILSVTLDDVGNYTVNCQGYSETHHLTMATEPGVERTDITYNLTS
DIDAAAYLEELKQNPIINNKIMN PVGQCESLMTPVSNFMNEKGFDNIRYR
GIFIWDKPTEEIPTNHFAVVGNKEGKDYVFDVSAHQFENRGMSNLNGPLI
LSADEWVCKYRMATRRKLIYYTDFSNSSIAANAYDALPRELESESMAGKV
FVTSPRWFNTFKKQKYSLIGKM
> Q4ZYH0_PSEU2
MGCVSSTSRSTGYYSGYESHEEPRAASRPTNSDDRGYETDSEPSSGNLIP
NARSVYSESVLWHGTSMQSKIDLRSKGFDAGRKTGGA TAGGNANMFMNLS
REAQSETRRHHYLSSSQKEAKDFAMFADTSNPTLVRTIGVRNNLSLITDP
RTGGTALMTDQSIPRKFVLGSKSSAPGENAKVFRDEMRAAGHNVSTKQAG
ELLREVQSDSDDDNFPDPDDFIMSRFTG
> gi|11611727|gb|AAG39033.1|AF221058_1 avirulence protein AvrXv4
[Xanthomonas campestris pv. vesicatoria]
MKNIFRSLGLKSSRSSAPEPSYQAPSSSDNSPQSSPDRSSPARPTTFSGL
ASRPRRKAELLASELQKAQSSSANNSSLQQYARNTLNNLENGIQPTPGDT
MIDIENLHEVVASYRYEDLNLRAFNSIENFIDSLEAGRSSQSRQRAIVRD
YPNVHHFAVDVKHHENGASTLIVLESASAGNEIALPGYTKLASMLRSKFG
GSARMVVIEAEAQKSLNDCVIFALDFALAAYQKRNSVFEGWHDNLASHGS
IADQGERHRKYGPFDSGLFRNHGVFLIKGWGVLPPIFYKHCQSREVLQGV
EKRQPGSLDTNVSTGGNREQDESLSERMEAFTDSRGYRPRNISIETSRAT
KIRHALSRS
> avrRpt2 Pto JL1065|Genbank: L11355
MKIAPVAINHSPLSREVPSHAAPTQAKQTNLQSEAGDLDARKSSASSPET
RALLATKTVLGRHKIEVPAFGGWFKKKSSKHETGGSSANADSSSVASDST
EKPLFRLTHVP YVSQGNERMGCWYACARMVGHSVEAGPRLGLPELYEGRE
APAGLQDFSDVERFIHNEGLTRVDLPDNERFTHEELGALLYKHGPIIFGW
KTPNDSWHMSVLTGVDKETSSITFHDPRQGPDLAMPLDYFNQRLAWQVPH
AMLYR
> A2UK49_ECOLI
MIKIGVIADDFTGATDIASFLVENGLPTVQINGVPTGKMPEAIDALVISL
KTRSCPVVEATQQSLAALSWLQQQGCKQIYFKYCSTFDSTAK GNIGPVTD
ALMDALDTPFTVFSPALPVNGRTVYQGYLFVMNQLLAESGMRHHPVNPMT
DSYLPRLVEAQSTGRCGVVSAHVFEQGVDAVRQELARLQQEGYRYAVLDA
LTEHHLEIQGEALRDAPLVTGGSGLAIGLARQWAQENGNQARKAGRPLAG
RGVVLSGSCSQMTNRQVAHYRQIAPAREVDVARCLSIETLAAYAHELAEW
VLGQESVLAPLVFATASTDALAAIQQQYGAQKASQAVETLFSQ LAARLAA
EGVTRFIVAGGETSGVVTQSLGIKGFHIGPTISPGVPWVNALDKPVSLAL
KSGNFGDDAFFSRAQREFLS
> STM1184 flgL flagellar hook -associated protein 1267144:1268097 forward
MW:34175
MRISTQMMYEQNMSGITNSQAEWMKLGEQMSTGKRVTNPSDDPIAASQAV
VLSQAQAQNSQYALARTFATQKVSLEESVLSQVTTAIQTAQEK IVYAGNG
TLSDDDRASLATDLQGIRDQLMNLANSTDGNGRYIFAGYKTEAAPFDQAT
GGYHGGEKSVTQQVDSARTMVIGHTGAQIFNSITSNAVPEPDGSDSEKNL
FVMLDTAIAALKTPVEGNNVEKEKAAAAIDKTNRGLKNSLNNVLTVRAEL
GTQLSELSTLDSLGSDRALGQKLQMSNLVDVDWNSVISSYVMQQAALQAS
YKTFTDMQGMSLFQLNR
> Q1LK60_RALME
MTWLIIADDLS GAADCAIGFAAHGARAVVTLDVADSVGIADAEIVAADVD
SRRMTPEDATTSNIDAWHRGDGRHRRLYKKIDSTLRGNWAAETVALQPLA
GLAIIAPAFPATGRTTRGGRMFVNGQPLEDTDIWRLEGLNGHADMVALLA
ARGLATALLPLDVVRAGAPAVLAELRARQADGIAAVVCDAETEQDLVTLA
DASNQLDTPAYWVGSGGLARSLSTVAQVTRAETTSPVTYHQGPVLTMVGS
MSGISGKQAAYL CERARMEALVVAPQTLRDGPGHPAWTTTRQSITNCLSA
GRDLLLSIGRDDAFDPAEGPRLSTALAQLALPSFEHVGGLIATGGETARA
MLGAAGIAALALRHEVEPGVPLSDTIESLPGAPARRVATKAGAFGTEAAL
WLAWQAMRQN
> A1JQC0_YERE8
MKTIIYYLSIILFLSPVIAQASCLSSAAIRWDVPEIILEAIIINESGGKP
DARNINKNGSHDYGLMQINTINIDPLKSQGIIKNKQAL MQPCTNIEAGAY

LLSQKFKKHGYSWRAVGAYHSETAHYRDKYASKIMKIVNSGPDFAQRNAS
LLRSTPTY
> gi|4494850|gb|AAD21323.1| HpaA [Xanthomonas campestris pv. vesicatoria]
MIRRISPGTLRPSVSIEHGGSHHHGDHHADASGTSAGSPTDIAARAPRLR
PAPPRRRRRGIRSLDGQEDEFDANEQEEIEAKRECALRGRVSVAITPAQS
REHGQGDQHGGDRSTHTDDPQAGPWHSAAPAQLSDSIRTCIDAIVDRYVT
RRDADPVAKRHALAAALVELRAVGVSHPAVAPLTATIWRLMREHLRAYDK
ATAAENLLALRTRLLELMPSELEPVPALRNFHLLLPLILLNAEKPRRRVD
RTHAITRLNTLLIEQPQQAAQEVRP
> hopAP1 Psy B728A|Genbank: AAY36934
MDPEKRVVISKTDQGSEANMPGHWTGCARPTRDPFVPFVAGRGESVA VRY
NSRFGRRFDDQSVNGQPDPSLAYVFLGHELIHASRTLQGTQYRVSRGRPD
VDEDSGAAEEELRTVGIGQYEDEYPSENAIRREHGLAPRTSYSGNTGTNL
REPPERNSQYTEARLRSLNARRWSDM
> hopF3 Pph race 6-1448A|Genbank: AAZ37227
MGICASSSRNQYSPAVSNTCSPQHVASHGNLASSGGNRITSVDQLNSTER
KRFLERQDPMRMFNFKKDTPVYRTMSPEF LVDGRVSGNPISRTWVRDHES
LRPNPNGGFPEGTSNAYWPVIREARDLGPSLNVMTGGPSYSRDGDINVRM
RLGDFIDRGGKVYLDNSAAGGDRQKTIPLVITLPEGQSVPAEKIVSAS
> Q8Y2V4_RALSO
MVSRHCGDRPALVRTIGVMNHFAIEEDPDAGTSCLRTNEDIPSSHVLGSK
GSPAEGNARIFKGEMEADGHSISLEEAGDLLRAVQSDSDSDG
> Q8XAN6_ECO57
MPVILNFSSE RVLSESELEALRHVGRVSQSEQLVVRGRTMRLHHISFMDS
FSVEPVSGGLLDRLSARGHRLLAENLEIQLNRGHTFLQAFRLYMEQSRAT
PCTRQNVSSAIQNKINSHAFTVSHQDFSCHEQHLNCPITLCIPETGVFVR
NAKNSEICSLYDHNALTELIRRNAPHPLSREPFVPEMIVSKDECHFNLIE
QYFCILATQNICTRI
> Q8Y166_RALSO
MGCVNVTGASGKASDYVAERHANQPSTEQAT QRPAARSAGQGVLQGLQRQ
NSASRHSDTDAESAIQYAVTQARLGDRFRPGPLPEQKELMGHKIGRSD
> hopAS1' Pto DC3000|Genbank: AAO54018 (N-term), AAO54017 (C-term)
MTLRINTRSATPVVPLETGSTSQPTPPPVTARATEPPPVANPAAPKSAPG
VQQAHGLKTRIAGKLSERQTNFSLGIPGTGRTLNRPLRSGIPEEGEQVSN
EESHDPLLKEAHELQRM VESALTHLKAAPTSLWERPAPSTVRRITTKIFP
WLKPAPLREVASNGSNAKTKIKINSQQSPETIAAAVKELSTRLDHQSKVL
ATATHALVAAREHLESLEQATPPSSTEPLDHARARVQQADSTTRLASQQL
RELIQGTDVLQLGALSEGQDQVEQKAEFSXXXXXXXXXXqwlselrtrmq
avdvrfadrhtaieielqlkvaqtrelisldhdmtaaehtaaqantqlsa
lraqrqetpegsdeadrl dtaigqtlatrsqamqthqqlakrlvrvdadv
arlndelgeihqriaivnderfalktldqklpaseqvaapaedqtqerid
ktalknvreqylasqigeahafaatgadeiqaalqrigdrlqgtppptpl
pafavievvtsaladvtgndatranrvldllnqhplehwprvaeemsksv
rtrsannnsvqrdtldlcrklanvprgmemlqvlssdgtapivaerakpl
rvfwnadeaqqkepdgkvk awlqtakqvarskldgdeksfddvdhaafna
vrngyfsnapgtpyaqhdqrlkkatmewvmravaantpeqatatatptkss
lprrlvptlnktpfakstldraysvgesmglqsprkqvdqvisarismle
etlknakgapglqleisaahamldhlkslekkgthlsqvtlkkrdgksmq
sllkarvqqqrlsqiwdkpdangkravnvlhkaqhielpplyselanskl
svyealnrvdehlrevlppv lqpaesveevldqdlsaaikllktrhlsek
sdivtffkpfilesrlrdrlrlggggtlgvglpslpyslispivspifsa
eksrsdeafaqlfmpilgmemsfgkarteaaeatigvaagsavadgvaiq
aaltgrftaqetqtsstlmrffrtrhkddemrgnmltaldsmvrwdiidp
qkgqryagpleailarnpevsvsqidatsstrnvaarvalrvpavrfkdg
ashasqtltpepsvyveadri ketrtetggfisvvgakgdtaqqrmgvta
nlnfapiassatpaekganhgvqgqglglqlgmsrdlawalekneispfl
igdkqdadldrhystprdmqaeitanrdlwlmrcietlepdetgnkntpd
nrlraamhldafeatikrlgkdskycqynvnysmkgragaeidgyrgiqa
lalqrgdvqaaekaqqaiddilltketwrplvlivrerardstvvgwrnl
lrwqklsnvdgqrtaaqfppp
> Q8XRU9_RALSO
MIPKTLPTHTAPISAPDGSPPEAGEQASPRRSSDVQAPRTASPDLRILAD
KGHGGRVGRSLSDIGAQSMLRRHATLPRSASQGGMAPMSVPDVPDVPDVA

ASGVSLSGGVGSADSTGSVAQGPAPTGSARRSELLGDYQAAIHRPLDLQR
ALTAIQLRSAAEIYCEKKNNLARFMPRLPRLPASERHFQALLDKPVDDLI
ADVQKHPHRYSRTQLTDLAQHHLLAALNIDVKQYREG IHKAALYEGLHSV
AGRVVSAAASGISIGVSELPGVKHATSAVVKGIKKGVKILSHELPPNVIN
PALTGSLRSITDVKEAFKRVGGLPVVAPQIDQSHDMGRIVQSAKEKRRQL
DEAIAHFRSAHRTDPQALGRLVDAFLALHNVADRQYRRRIGLNRTQTYSK
GWGMAVNGVAATGAVVTATVPVVGQIAGPAIIGATIPLQWGAGYLDERRS
KHRYNLRANTKWGDFLKENAAHIHFMDLKPKHVSEPAL RRSFMTQPEVQI
AAIREVYEDGLGELMRQHAELGNKIAEQENKIDAQRRTGRPARALVPQRE
QLRELEQRRGDLEHRINEAKQHAGHFESFDIERWHSIPADSLIGRCLDDL
NQLEKANRHARLRKPGEGAQIVQRYVQAFHGGLSTGTALPIVDAITSIDA
FYTHDAQGNTGALQPAPEVAAVGAGVVGGTVFTAATGEVRMSKAYNKKLM
SELRVSPETYAAHQDQWVFRADNRSVDLRTTAGYRQHVH SKRDELALVGQ
ALKHGLTSGPVGLKNLLLAKGELRHARASLRSALNALVEARLPRTETAGK
RAPTISAMKDALYDYPAVRQHLGVDAEAVG
> gi|12620462|gb|AAG60738.1|AF322012|43 ID84 [Bradyrhizobium japonicum]
MYGRVVGSSSQSTSANQVDEPGDSPRFAETLAGMEPGGSSSETRAYYLNS
GPPIVEIDQLSFDRGLQRFLGRDIMNIAINPQEYSDFVS KKAERAATVAG
SYSATHYDPARPVRFFSYQLGDETVGLLRAGGPVRIKGETFREKFGRNDL
TSVVDLRVTHPLVENAGDILLEYQLREDGDDPLILSKPGLPGMEPRLAEM
GFVHVGRNHWVLDPHQHPEVWTKNENDQWQRVGKPTKYLSKVEDDDAAAE
STVQADYSDEDDPSVYLERVLTGLSME
> Q8Y2L4_RALSO
MLKTRITPATQPASTSTVAHPDGATESQTQPPQVSGPRARTADRNFTN LP
TRHASARALKQPVKLTRAASKVDTRALAAAAIQPGLSARLESMRQYLAEL
KTAAHADPNLRVETPGHRDAEFLDLLVAVENARHPKLALSAHTISLNTLN
TTNPAAVASLARNLETSMRSREPWHAVVDIGGHAMALSVRHHPEQPKQIS
MIVVNSAGEHLSVPEWNLMATLLAGHLNHKLEQAGDPKGVRIVLHCLNTG
IQKTNHGGAIFALSAVKAMPGDEDIGKLHAHALSEAGKHADAVDVHVID E
TPLLGPRFFKHMTHAFGLERMLTNRPDLRTAPVNKKTGLPLAQYQSQHLR
PHRLLQHVRETSSTSYEDKRLQLYERAITHLASARVEALTDHLDALRRAC
KGKADMPAPRDAEFLDLLVEVENKLDPQLRLSAHKIDAPRLAVRDPGAIA
SLAPVVADGVRGGGDWHATLDIGDGHHVAVAARHDAAHPTHVSLAVLDGA
GSPFSPANWRDLASTVAKCLDAAGNGRTGKVWLTYVDASSQATTSNSALF
ALRATKEMKESGLQHDGLIDQMHKNALNLARSAQTAVSLGKYGGPAPLDP
EYGTGGGADRYSLDGILRQEHIKMYKHAIRHFERAKEPAPERH
> gi|51591623|emb|CAF25427.1| yscH, yopR, lcrP; putative type III secretion protein [Yersinia pseudotuberculosis IP 32953]
MTVTLNRGSITSLMSSSQAVSTLQPVASELKTQLENK LKSESAEKTREVL
WQQYYASNPPDHAVLEVLAMPVREALLARFGQHQGSVVPAIDLPELRSVL
QQFDSFGKRWEAILLQVLEGIKPNESQVGLPYLSELINKELMILLPSNSI
VDSLLHNSHQIDMDT
> NleG_Crod
MQPVGLSNTPASSLLNETAELRHQTTPRQLGGIRIVGEIVDISYEPNDVV
FFGVGRGVMIGDILFPGSSADIMRELLMAHSGVRGILQQQENAPSSLQTR
ISECTFSVSQ EKLQCPEEEMRCPVTLETPEKGVFVKNSGDSSVCTLFDSS
SFSRLVRDGGVHPLTREPIKASMIVSSDKCVYDNAKGNFVIKNS*
> Q7A9J1_ECO57
MIYMGLWLKIFKIVVGENENYLKDVMMQLEAKNNEGKYVISKANGNPVFK
ELFWKAIDEFNFPQEELNRLKQYRSL
> gi|28868062|ref|NP|790681.1| candidate type III effector Hop protein [Pseudomonas syringae pv. tomato str. DC3000]
MEQEKSSCLRYGVTLNEKDLSRFLGTTQHYMWSTIKNEYALTESIDHLMA
QHQQQLMRSISFELFQSMPGVEALLNLLEHTGVPCAVASSSPRNLVELIL
KKTKLRRFFKEVICGTDVKESKPNPEIFLTAAKGLGVSPRACLVIEDSHH
GVTAAKAAHMFCIGLRHSSSFQQDLSAADLIANNHYDIKQWFAEK
> gi|17431349|emb|CAD18028.1| POPA PROTEIN [Ralstonia solanacearum]
MSVGNIQSPSNLPGLQNLNLNTNTNSQQSGQSVQDLIKQVEKDILNIIAA
LVQKAAQSAGGNTGNTGNAPAKDGNANAGANDPSKNDPSKSQGPQSANKT
GNVDDANNQDPMQALMQLLEDLVKLLKAALHMQQPGGNDKGNGVGGANGA
KGAGGQGGLAEALQEIEQILAQLGGGGAGAGGAGGGVGGAGGADGGSGAG
GAGGANGADGGNGVNGNQANGPQNAGDVNGANGADDGSEDQGGLTGVLQK
LMKILNALVQMMQQGGLGGGNQAQGGSKGAGNASPASGANPGANQPGSAD

DQSSGQNNLQSQIMDVVKEVVQILQQMLAAQNGGSQQSTSTQPM
> gi|28871145|ref|NP|793764.1| avirulence protein AvrPto(DC3000)
[Pseudomonas syringae pv. tomato str. DC3000]
MGNICVGGSRMAHQVNSPDRVSNNSGDEDNVTSSQLLSVRHQLAESAGVP
RDQHEFVSNQAPQSLRNRYNNLYSHTQRTLDMADMQHRYMTGASGINPGM
LPHENVDDMRSAITDWSDMREALQYAMGIHADIPPSPERFVATMNPNGSI
RMSTLSPSPYRNWQ
> Q6RK52_ERWCT
MADMTITLSIQPGAGLSSTGLNSPLSGSNGGASPRSGQQDSQLMEALSTL
LSALLPNSQGNTPSSTDGSS QGQAGIGNGGSSALGQNGSAADMLMKLLEA
LIPSKNGQEAGNPLSSGSSGAAGGNSGASPLTGSSGAGGVGGAQNPEDLS
RSLLQDSAGSALNNAINPTADGGGQLSGNDLLKALLELIGNLMDSQKGEF
GQPQGSGQSQGGGSPSTGAPQASSGGGGGSPAAPSAPSSSVGGNGGAASA
PLTAAPTGVDGGSAASPTASTAGAGPVSFPTASANPTVVNDTIKVGPGEV
FDGGGKTFTASSKLGDGGQAE GQKPLFELAQGATLKNVVFGDNAADGVHV
RGDAKIDNVHWTNVGEDALTVKSNSGKPANVEITNSSAQGASDKIFQLNA
DANLTIDNFKAKDFGTFVRTNGGQQGDWNLNLSNIDAENGKFSFVKSDSE
GLNVKGNNINLTNVNNHYKVPDSANLQVN
> Q8XQI6_RALSO
MGRITSTPGPQNPAGIGASPDVQTSGHATPAPQPLPVGRRSPIQSALRAG
LAQAPRTSATALPQSSVARLPSSGEKLT PPREITAPGANEASGPRSAPAH
AADLKPVVVLDWDDCLRYEKGMNYQLVHNALEIAASMHAQSLPELRAAVD
RLRGRMARGEQPGEGDPLIMQSQEDCANYLAANPGIYKRGMVEDFVRTML
PDVDSGKAADIVSAVYTQCVQEYSRLAASPRLRTARPCEVPFPGVRIALM
PGARELLDTSHAAGSPVMLISNRAHSDLEKEVRYLDMQQDFDVVSGAPTV
TRPRSNASAAPMPEALQQRLTAALQGDDD GALRAALEDASAYAHPNTTSV
TQTDRKPHAARLLDSLQRLSVPSEAPIILYGDQPSDISQAAKLAETGRRI
EGVLIDPGHNDVGRQIDVEGIPTRVIRSLADPDAPWKSAARAVHEPPLFP
AMRTVPFILSRPEQSAPPLPVAAGDGVVYSRAKQALGGSDVGMLAWMDGH
EHLVKTGLSRSLLGQIMSCRARGQESEAEKRVGYVVDIWARLSGDDTQKD
ALKDQLKKTLHAFIQSEKGSRLFEKARSGD LDLTDVSGLHRALTDACPQL
KNPLGMPVIFDLVNGVASQALANALQRTYLAGSHVPDATLLVSPDNVLFA
SRILQDAVPMDAFLTRAFLPQGVSLRDAKLAAARIKTGGAESAELRGARE
LIARICAPRQLDAGRQGLTAALQAQGMDGFFTSVLARLTIGESHNDLGPD
NMLIVPGADGRHKAVNIDVTGFWESRRDSIPAIDQAPPRPGWGEVMAHPE
QAADILLDANVLSGRYARGFEAVHEVVVDVL RNTLRDQAAPEAASVRAWY
AAQNEAHVLASMSELQHGLRDVAQLGWMVDPEMLEKGFSRNAGFIRDVIG
QAKAAQQAASTAGHAKAPGETASPRTTPGTIAQAPQRHPGSHG
> gi|15595242|ref|NP|248734.1| exoenzyme T [Pseudomonas aeruginosa PAO1]
MHIQSSQQNPSFVAELSQAVAGRLGQVEARQVATPREAQQLAQRQEAPKG
EGLLSRLGAALARPFVA IIEWLGKLLGSRAHAATQAPLSRQDAPPAASLS
AAEIKQMMLQKALPLTLGGLGKASELATLTAERLAKDHTRLASGDGALRS
LATALVGIRDGSRIEASRTQAARLLEQSVGGIALQQWGTAGGAASQHVLS
ASPEQLREIAVQLHAVMDKVALLRHAVESEVKGEPVDKALADGLVEHFGL
EAEQYLGEHPDGPYSDAEVMALGLYTNGEYQHLNRSLRQGRELDAGQALI
DRGMSAAFEKSGPAEQVV KTFRGTQGRDAFEAVKEGQVGHDAGYLSTSRD
PGVARSFAGQGTITTLFGRSGIDVSEISIEGDEQEILYDKGTDMRVLLSA
KDGQGVTRRVLEEATLGERSGHGEGLLDALDLATGTDRSGKPQEQDLRLR
MRGLDLA
> Q1QSJ7_CHRSD
MKQLLAAFYGDDFTGSTENLAQFCRHGLRGRLFFTSSNREAILEMASELD
VVGLAGTARGLAPSAMTEEVDPALALLDTLSPRLKQFKVCSTFDSSP TVG
SIGHVMDLARRRWPECALPILPATPAFGRYTAFSHLFTRYENEICRLDQN
PAMAYHPSTPMGEADLRRHLMAQTAIPPAAVTLLDYQQPDGGWQCLASAV
EEQRFVVMDATQAAEIHHAAKLILKLAERHPTLAVAAQGLADALGGLVTR
DMEQREPLPTTYPGVDRLLVLSGSCSPQTRKQLAHMEAQGGCIVAISPME
ALRSPSALSERLAQEVMEAFTQSQTVGIATTFQDTDLVDGLASDRLSL AV
GEAYALLAQHLRRETAIPRIVFAGGDTSSHAMRRIGADALELVAFDEAQG
GHLCRLIAPGSPIEGLEVVLKGGQIGGEDFFSRAQQGTSPRV
> Q8X9A7_ECO57
MPVILNFSNGSVLPENELEALRHIARSNQNDTITIGGRNMRLHYIQFMDG
FSVEPILGGLWDHLGAREAHHLADRLTRQLNGGNTFLQAYSLYLEQRQAA
PLVQESVIKTLLDRINSNAFPVSLQDFSCTEEHLNCPITLHI PETGVFVR

NARNSEICALYDQEALTELILRNALHPLSRDPFAPEMIISKDKCHFNITK
QCFYALPIYPLQQNSI
> gi|15618615|ref|NP|224901.1| hypothetical protein CPn0705 [Chlamydophila pneumoniae CWL029]
MELKKTAESLYSAKTDNHTVYQNSPEPRDSRDVKVFSLEGKQTRQEKTTS
SKGNTRTESRKFADEEKRVDDEIAEVGSKEEEQE SQEFCLAENAFAGMSL
IDIAAAGSAEAVVEVAPIAVSSIDTQWIENIILSTVESMVISEINGEQLV
ELVLDASSSVPEAFVGANLTLVQSGQDLSVKFSSFVDATQMAEAADLVTN
NPSQLSSLVSALKGHQLTLKEFSVGNLLVQLPKIEEVQTPLHMIASTIRH
REEKDQRDQNQKQKQDDKEQDSYKIEEARL
> gi|1772618|gb|AAC31975.1| HrcC [Erwinia chrysanthemi]
MRNALYALLFNLYRLFCWSLVLMGMLPGAHAATPPDWSKGAYAYSAEQTP
LSAVLQDFANGHGVDLVLGNIEDVNIEAKIRADNAVAFLDRLALEHRFQW
FVYNNVLYVSPQDEQTSVRIEVSQDAAPDMKQALTGIGLLDSRFGWGELP
EEGVVLVTGPTEYINLIRNFSQQRETKEDRRKVMIFPLRYASVSDRTIQY
RDQRLVIPGVATILNELMDGNRNTPAGAAGAGSGIGSDGGMATADGAGGG
LDASKSIEMNARSMLARLASRNSGAAASASRKKDQLNGRVSADVRNNALL
VRDDEKRRDEYQQLIDKIDVPQNLVNIDAIILDVDRTALSRLEANWQGKL
GAVTGGANMMSGSSTLFVSDFKRFSADIQALEGEGTASIVANPSVLTLEN
QPAVIDFSRTAFITATGERVANVQPVTAGTSLQVTPRVVGHGSTSSIQLV
IDIEDGQVETNTEGTASGVKRGTVSTQALIGENRALVLGGFHVEESGDRD
KRVPLLGDIPWIGKLFTSRQHEVSRRERLFILTPHLIGDQNDPTRFVSAD
NRRQLNDAMGRVAQRNSKTDLFGTVENALRDLASGQTPAGFTIDGSGPKL
SEICRTSPSMSYQSNRYQWYSNLSLSLTVGVVKNTGTQPLRFDEASCANS
RTLAVAAWPKTTLAPGESTEVYLALLPAKNAQPSRASLIKP
> Q0FF52_9PROT
MVTFLGCIADDFTGATDLASMLARSGVNVSLRIGVPRTEPKNTADIE VIA
LKTRSIPASDAVKESLSALKWLKQAGAKKFFLKYCSTFDSTSKGNIGPVS
EALMGQLGINQTIYCPAFPENGRSIFMGNLFVGQKLLSESSMKDHPLTPM
KDSNLVRLLSAQVTNSVGLADHMIVNNGADILKEKLIALKKDGVAHVVVD
AVAEKDLEIIANACEDMDFITGGSALAMPLAELYKLSGKISENNNIYRNH
ELTFGSVVLSGSCSEMTILQVKDFIERGAVAFQLDPIDMAENGIKKVL DW
LTEQDFTKNIIIYATADPNIVRTVQDRLGVENAGRIVEQGLSECAVKAKE
IGAKNFIIAGGETSGAITKALNISQLDIGIEIAPGVPWTFFGTKNNQMAL
SLKSGNFGKVDFFTEALSKLENL
> Q8XZK9_RALSO
MAGGRVGARAVRESAQTPVSADASTSTSAAASPPAAAARQAARPAELRGL
ASAPSSRASSSSTGGLSRQASLPARAVARETPEAPVDVFNRTMRHVNAWR
AVAERERKLEALGPEHKGLSPEREMRYACDAIASIQTCLQALAAMKRQQE
IPPGWDVDGQEAELLQLLIVAADIGVCTRHETLNKLADQDAVFRAQRSGT
AEAETVSGPDGPRPDTIEEPEPAGATAPSPSEQAAISPLSALEREPGKIQ
AWLAEFTSCRSALDQIIRRAEVCGASRTVQTALDTMLPNAIYDKLRCCSG
MQALHGILAAASANRFVELSRQMALPDLAVRADELLAAWHKENAAMEAAV
DALVMHGAPDKPTQRLTPKALDGHQAVIEAYAEGLDRVGLNLCLDAAADI
EMDDADGVWRSMMEVVHAISAYKASLLELSETAGRAKTQPEAPQPEPESS
EAAPEPALSEPPPAARPAGASGGMRRKHRKPGMRAAGPSVPEPVSARALV
PADTRTVAQKQADTLLRRCRIDRATVAQLGGDIVELAQRLGEDTRLVLSA
LNDRTRDAVIAAKFIWGSVGRWFGEPDRVRSAKASLHAGDHDRIGRLTNW
LGALELIERHMAPLEADMLKRDRYPKAKHLEKLLQAKGIEFVGVPGRLPP
TEDVNAVGTLFGMRIQFKPLSNRDQVAPWFVHLHTNRPVTAQALSTLAFE
DFTAVHLKTDRDKNKGAQWEAAMRALGYTEAKVHRAAIGEALLHKLFVQA
AKQPRRNKGGVRPAG
> YPCD1.20 yopT Yop targeted effector 12610:13578 forward MW:36308
MNSIHGHYHIQLSNYSAGENLQSATLTEGVIGAHRVKVETALSHSNLQKK
LSATIKHNQSGRSMLDRKLTSDGKANQRSSFTFSMIMYRMIHFVLSTRVP
AVRESVANYGGNINFKFAQTKGAFLHKIIKHSDTASGVCEALCAHWIRSH
AQGQSLFDQLYVGGRKGKFQIDTLYSIKQLQIDGCKADVDQDEVTLDWFK
KNGISERMIERHCLLRPVDVTGTTESEGLDQLLNAILDTHGIGYGYKKIH
LSGQMSAHAIAAYVNEKSGVTFFDPNFGEFHFS DKEKFRKWFTNSFWGNS
MYHYPLGVGQRFRVLTFDSKEV
> gi|28898430|ref|NP|798035.1| putative translocator protein PopD [Vibrio parahaemolyticus RIMD 2210633]
MLDKIGGTGRGELYGLGDTIKTTKAEKTPETKLEAGAVKSNGESNVSGAG

RYQLDGPKAPAISNQAQVVANLMGELAPTIDLLMSTTAKVLNGEEVIKSP
SDAVSQSLSLLTLLYQVSKLSREQQVLQREIAVEANVASLQSQAAELNNS
ASAMIAMAVVSGVLAGATAIIGALGSFKAGKEIKTEMASNNVLKTQKAGF
DQVEELMNNGNLSKTQQDQVKRAHSLAKDSIADTTAQLTSGGRKFDKLMS
SNQAKNAILQALGQMANSASNVEQTKAQARSKDDEVQATRAQAAKQKADE
NIGFQEGLLKELRELFRSISDSQNQAWRASIPTV
> gi|58579719|ref|YP|1987 35.1| Hpa2 [Xanthomonas oryzae pv. oryzae KACC10331]
MINSTIACQYVGKACNWQLEASPGPSPKRRVHAQLSRCGRWAGTGPQRIL
FAAALACAAPFARADCFEEAAGYQHVNPWVLRAIAWQESRGRADAIHRNN
NGTVDYGKMQINSIHLRRLFGYGISKEALMQPCVSVYVAAWRLREMTNKY
GNTWAAVGAYHSETPGERDKYAHAIHSILLRRGVIGE
> CP0130 ipgB1 IpgB1, secreted by the Mxi-Spa machinery, function unknown 113520:114146 reverse MW:23684
MQILNKILPQVEFAIPRPSFDSLSRNKLVKKILSVFNLKQRFPQKNFGCP
VNINKIRDSVIDKIKDSNSGNQLFCWMSQERTTYVSSMINRSIDEMAIHN
GVVLTSDNKRNIFAAIEKKFPDIKLDEKSAQTSISHTALNEIASSGLRAK
ILKRYSSDMDLFNTQMKDLTNLVSSSVYDKIFNESTKVLQIEISAEVLKA
VYRQSNTN
> gi|27376899|ref|NP|768428.1| hypothetical protein blr1788 [Bradyrhizobium japonicum USDA 110]
MQTARLNADVEDGLYDGRLGELLQNDRVLFRLEALDGIARERVNSLRRAD
PDADVDEIEVYLAYQAQLRDALELRHNAPDMRFMNVSQVTEADVARAEAS
ARDGKRRNFGTI
> gi|15605308|ref|NP|220094.1| hypothetical protein CT579 [Chlamydia trachomatis D/UW-3/CX]
MTTGVRGDNAPDPSLLAQLTQNANSASAASTGKNGQVAGAKQENVDASFE
DLLQDAQGTGGSKKATANQTSKSGKSEKAQASSGTSTTTSVAQASQTATA
QAVHGARDSGFNSDGSATLPSPTGTEVNGVVLRKGMGTLALMGLIMTLLA
QASAKSWSSSFQQQNQAIQNQVAMAPEIGNAIRTQANHQAQATELQAQQS
LISGITNIVGFAVSVGGGILSASKSLGGLKSAAFTNETASATTSATSSLA
KTATSALDDVAGTATAVGAKATSGAASAASSAATKLTQNMAESASKTLSQ
TASKSAGGLFGQALNTPSWSEKVSRGMNVVKTQGTRAAKFAGRALSSAMN
ISQMVHGLTAGIDGIVGGVIGAQVAQEQRMAGMAEARAEELKSLNSVQAQ
YASQAQQLQEQSQQSFNSALQTLQSISDSALQTTASMFN
> A3LKP0_PSEAE
MICKRFAVNTPHPSLRRSCLAALACSALVAQGAFAASASEQANLEVMIRQ
LNALEDTARRSAQGADEPGQRFYFDYSRLAADLQRIRQGLQDYMTPSRAQ
PRDPSDLSGNYTLRGGPMP
> A0GC88_9BURK
MTATTKRALLGCIADDFTGATDLANMLVRGGMRTVQTIGVPASNETVEAD
ALVVALKSRTIPAADAVAQSLAALDWLRAQGCRQFFFKYCSTFDSTDAGN
IGPVTDALLDALSAEGKAGAFTIACPAFPENGRTIFRGHLFVGDTLLNAS
GMENHPLTPMRDANLVSVLQRQTESKVGLVRYDAVAQGVSAVRDAFDTLR
NEGVRMAIADAVSDADLYVLGEACADLTLITGGSGIALGLPANFRRAGLL
KEQADAAQLPQVKGLSAVLAGSASKATNEQVAQWRSARPAFRVDPLAAAR
GENVVEQALAFAQPYLDKAEPMLIYATATPDEVKAVQRQLGVNEAGHLVE
NTLASIARGLHERGVRKFVVAGGETSGAVVQALDVHTLRIGAQIDPGVPA
TATTGAEPLALALKSGNFGTADFFEKALRHLNGAAQ
> Q4ZX50_PSEU2
MATMRISSSPSPALGSIVNQPTSGELAAETPLAKASLTQGSAGGGQAFVQ
FGQANDSPSSFSGTEQNGSSLMSLLTRSSSSDSTSSVDQDSEVSPMTSVS
STASASPTTASNPANAPSATDAAFLDNSEYSSPEALKRWEPMVANLPPEE
REQAAKELNRPIAAAWMAREDGPNAEKAMAFINANPALKTAVDVGKDGGN
ADGKITNKDLKAFAKNMEKAADNADKDVAKYMEDNPGADPQSLEMVRSAA
VMRANMPLATAADPHHAVGAADKTDVDGNVSAEGLKALIKSNPGLSGTLK
QSSNMWSQAGFLSQVDEAGLTGRKKAAHSPDQVFDASNMSEWIRKSAPKN
GGQFASMLSDAATLNSVAGIDISKLNAQVFEKPKAYTGAQKAAVMIKLQQ
TQQSVIAGRDLRNTEKTEAGLNERIAQLQADPDVQEYLNKSIPEQERSLV
RSDSALQKAVTEQAQNVNSGKALQTDMATADKAVDKHNPNPDYSGAITGL
SVQLQLQKDLFPDAQVPTAQQVFNNQPDELQTKIADSYVRNFSEGGALKQ
LLGQKKSDAGESLQTADNQKAAYESVLPADFVNGERESYTASTLSELQNS
KKGRKLLEGKTDEEGGPSLAAQLAEQGIGGKAFNSVMGFASVSDRLASGD

KLGAAQSIIDSSRLGAESIKGGIDTGAKMMGREASAGLGRLGGQMIGRAV
GLVAGEATGLAAGAALGAAIPVIGWAIDGAMALGFGISAIIDAVKKHKAQ
KAFDHNVDPVLDQFGIDKAH
> gi|50121143|ref|YP|050310.1| putative virulence factor [Erwinia carotovora subsp. atroseptica SCRI1043]
MKRLTPKQLSTRLHGQLQAVAQGVEQAIDWVETTRQNAPRLDIEADRLIV
KLRRNHNKAQHLSDVAQKEIAIGFFGLSQAGKSYLISSLAGGENGKLETS
FEGQQLDFVDHINPSDRATALVTRFSRQSGVKNKSFPVQLQLLSELDIGK
IMANAFLNDLNQETTFEELDERHIAEHIKTLLMHRQPEPVEGISRDEVVE
LWDYLVRHDVKRQKQLEAHFWPVAVELAPYLTVDDRAQLFSVLWGELNSL
TSAYRHFNHTLQHLSGASKLLAPLRTLVDDELNPADGLVDGSALERLHST
DDPSVLVRPVQNGRAGKTAELSLAELTMLTVELLIPLQSPPKEALFEQVD
VLDFPGFGEVRETRAAPPAKGQNTLYPLAHTLLRAKRAYLLERYTDNQEM
NVLMVCSAAGDRADVKVVGKALDHWVKQTQGENAQVRSHRKPGLIWAVTR
HDRRITHGQNYDAAVQRYVGNPGDAWGTMLAMDKRGVTRMATWLGTEVHR
EVKLGRISEQLNEIQRELSDNLLGNWYLPVDVDDPAEKQRIAETLLKSLQ
TRTGVHGELLERLLPSRDELRRLYLQQKGASYGGFHTDAEDLSAPLANSD
PFGVGIEIDLFADEPIAIDQPVMPVLTIDHGYEADYAHGVYRYWINYLRG
LPENAPLLDLLNVPKSTIEMLVEELITGSIRLRIEEALVDMLVDGEQLGI
NRENKADRQVSRVLTILGDFVAWLGFQQLDESLRPASRINRGHKIFAKPE
KQSVSFGASQRLTKLSLTPTNNTAFYIYDWLVGLNEMIIQNAGYSAAREV
SAEQREQLGTILALIKPAEK
> Q8XT98_RALSO
MPTSYVNLDQLTTEQDTSPIDAMPQMYIMGAPMVHVSPFESGSAVINVTT
PECTYVENGEARSKETRSQVELSEHMRSVMTSAGKGPHLYRTNNMISELG
KLMPRMFGGAPEAFLGGHVCTVGSSDQRGTSITSVTDNALTIIAPKATSA
VTLDLAGKISTASRFNVH LPLKQEGDLQIRMDRDVLADKAAAKARKMGLP
VGMSRVLDRQPGQVKQSGRFASEFILPRGAEKMATGDVFDAAFRDRSGRM
HVVIDGIAYGQDRRLGSGFGGEAFIYRDPATSHEIVVKEYFRLRSQASPG
DMDVNPPKDAGKRAELEKIQMQDFMIEKSAFDALATHPAAKNIAHALRAG
TVDGRFTIVMPYFRGGSVRDLCKNLDKAVEDGIISVGQRRDSALYIMQGI
LNGMKYLSSTLIHRDLKPD NVLLHIEKERDGTRVLIPKIADFGTSVLGTS
SDLPVVTTPQYKSPEYLRAEQQGRGQHTAAQDVWSAGISLHELLTGHRPF
DGDLPKDEHKIYDAIKNYAAGNTEILPADQSKAADLIRIMMNPSSDRATP
AELLDHEAFHGINEKECQKTLSLIHQS
> Q8XUL4_RALSO
MKVGNQSNNVGHDFSVKGVAGSSRRDGAGLDIGGDTGGLVDAVQRPRQRP
TMLDVLPRLKFATRIKQQRKMMPSSHRL GRPSDLTEGGSSSGTEAGNGRF
KSRLANELASSGKPLKFNKWEPADRFQLSSESDSIVKRGARVQAADRNIP
SSNRFSMDLTSAQSTARALAKRPLQRLTLESRLDIHAHGNDSYVEDLTAA
ELAAKLHDAGLEEVGVLKIQACDVGKGHYLQALLEHLASRGVKVGYLCGP
KGALTDTRTVENLGGGEQYVKKDVSFLPGKYVGWLPETFGLNVIKGNVDV
AFGGTRYNLSR
> Q6FEZ2_ACIAD
MVQELLKQLQNGEAKFADVLAYIESRFEHQPTAFKNGQQQNAATENQGSA
KVFSLAQIEGLNQTETLSLFAEHYAAVLNTPEGTDHQNIRQFMQHGWDGV
TFEGEALQAK
> gi|29839807|ref|NP|828913.1| serine/threonine -protein kinase [Chlamydophila caviae GPIC]
MDCQSEMLSHNQVIGAYHIKKLLSKKEGSTVYQGVHPDTLQPAAIKVLAT
PLVTDTPRVHNFLKEARIIEQISHPNIVKLYQYGQCREGLYIAMEYIQGV
SLRHYILSQLIPLSRAIDIILHIAQAIEYLHSRGILHRDIKPENILITSQ
GNIKLIDFGLAISSIDKDARPLYLGTPSYMSPEQRQGDKISELSEIYSLG
LIAYELILGNLALGKVVLSLIPDRVSKILAKALQPSPTDRYASMKEFIID
LHRYRHGEDLQKDLRNKDHTAMVNDQLYHQRFWLSPLEIVVPEYLAVSIH
EHGYPTHPHVYYEAYMSGDTFRLWFCYSLSGNPTLVLTVMKSFVSQWGHE
ENIRSTIRKMHSELMRINVPINGKGISLFCVTIPKEKKELSWIACGKTSF
RLKKQGKVPEIFTTSSAGLGKISSLQIQETKVAWEIGDGAVLHTLQADDS
MSSLHSPLFTELKDRGQTAIFCPIESVRYGIEENHDGNLCPSTLISLKRI
R
> Q4ZNB6_PSEU2
MINLTNLASSLARVALSDSTKPTIDRAMNVVSHIAGKVA LQVNSSLLEQK
GRLNERQQKGLEVILDALLGKEPVTHVETHEGGGRFNLARAAFDVASVVW

ERDQSMRNVMSFLGINDSKGNLLFSLGKTLADAMARPEPENGKHNSEANY
AFFSSNLKLNKLMNDIADQVINEIRQQNTDRVRRPTPGPSWRPESTQQQA
RPQTPPSARPQANSAPPPPKAQPDAGAQRPSTERPPKTTPTGASAKVDDS
APAKPPVKQLYEHLGLSDMTADLSAVKKAYKKAALKYHPD KNAGNVAEAN
ERFKKISNAFRILSDPELRTKYDNGVIDENGNEFKT
> gi|28867817|ref|NP|790436.1| type III effector HopPtoC [Pseudomonas syringae pv. tomato str. DC3000]
MTIVSGHIGKHPSLTTVQAGSSASVENQMPDPAQFSDGRWKKLPTQLSSI
TLARFDQDICTNNHGISQRAMCFGLSLSWINMIHAGKDHVTPYASAERMR
FLGSFEGVVHARTVHNFYRTEHKFLMEQASANPGVSSGAMAGTESLLQAA
ELKGLKLQPVLEDKSNSGLPFLIACKQSGRQVSTDEAALSSLCDAIVENK
RGVMVIYSQEIAHALGFSVSSDGKRATLFDPNLGEFHTHSKALADTIENI
SSADGLPLIGVQVFASKIH
> gi|12620505|gb|AAG60781.1|AF322012|86 ID186 [Bradyrhizobium japonicum]
MFQNTKALSNSER ASELDEMGTSSDDGSHTDSPRFLDVFSALSLAPSSCS
VDASLAAVPPYALVDERPVVEISKSSFENKLADFYGGDIEDIAANPQRYS
RWVSEKAHAQRTLHFASARSLFRRNRAFSAISLGTRASGF
> Q3BYJ5|Q3BYJ5_XANC5 Xanthomonas outer protein D - Xanthomonas campestris pv. vesicatoria (strain 85 -10).
MEYIPRYEALEKVPVRVRF HAYLRGDGSFGPGLPGILRYMTPDQKKRLYL
ASERRKLALAAPKSKPTPKSKPLKGVFRTLHQKPNLLLEISSKFSNRAYS
INDSSSGYLSQADLEEMVDEETGELTRLGEAVISGASQGIQTAIRANFRM
RYQQPDLPPYSPPQAFHRPEETWNPHTPAGSSYSSLFPPTPSGGWPQNAS
GEWHPDTPAGYSHRAWPAQPEASSSTFDDLESLDYRQNYGYREFDLNTPQ
EIEQPGWWQQATPAQSTDST FDGLSSMSHYGSEFDLNIPQQEEYPNNHGT
QTPMGYSAMTPERIDVDNLPSPQDVADPELPPVRATSWLLDGHLRAYTDD
LARRLRGEPNAHLLHFADSQVVTMLSSADPDQQARAQRLLAGDDIPPIVF
LPINQPNAHWSLLVVDRRNKDAVAAYHYDSMAQKDPQQRYLADMAAYHLG
LDYQQTHEMPIAIQSDGYSCGDHVLTGIEVLAHRVLDGTFDYAGGRDLTD
IEPDRGLIRDRLAQAEQAPAE SSIRQVPARSNEQKKKKSKWWKKF
> Q8XR44_RALSO
MRGGTALISITRRRPAQPVAPAHHFSPGSATSAPAPENTPRQQRRAGSAL
QRLAEIQTAQSASRPVVTGPSASGTGDATRVHRSMRDFLAEAPELHAGDT
AGGASPAEPAAPQKSSKSGLAKFTGKVKKLVGKAKTAADRNFVSSQTSVF
GLHTETTVGKPVTMSATRPRPEVLQSRPVPAGTRPEDLPSVPATGPAQPA
ARQRKIDATVKA AFNPSSSIAESMRSHLGLGQDKVARQSVSIAEAMSSEA
GMRVHLARLLSQPLHAPAVRALHGALLDSLPQATESASGRGFAQHVLLAE
ALVNACGGDTVRATAALRALKAGEFLPTAHGTGDAASPRPSDAGAEDAAT
AQAEPAGQHARRIGNASANAIHPTRQTPVEHAMLDAMHAAQGLAGIGDVG
MQALLAVMPPLQPAKREPLEFVLQAVEQVSVETKGAQVLLPDIIGHAEKM
AGTRDDTLACKVL RAEVKALRAADTYDALSRADKSAVFAWRQGFLTDDKH
SLLSQTQQRLAKFRKYVSRAETRNALNEARQDPARSTAATARLVANNVQR
AFSRKKSPLLAMGKYGSLAAGTRHVPTDQVELDKHMRTAIDALKDRLQTR
SSEARFSLGRYGEVPTVALRGAILEHWSAASESKRPQGYTLDGNAMVDIA
ERLHRAIGESVVRADGRLPQQLERLIGTRLSHATLATWARDAAMPQRTET
GEETAFSSAMRRAR NILEPGKDKPVDMTADSMRAFLKNFTGEHNVGNAMT
FTDGGALGVNTGALTLNLASLVKRFKGGFALTPVFDAQGSVARSAAFNIG
TTAHGGEIFIGRQRQVVGQLGGGLTAGYTTPTDADENSFSAGVGISGNVT
LFGLEHTNPTGVRLRFTTQRKDDGSAMNSDAMRKQMSDMVDYMFEACGPD
KRHLSPSALWEDFAVHHFDQKNLSVAWEDYASVNSKMGASVTLTARAGVT
TQDGQTVRAGGSVGY GFTWNPFTIGQRQEKSGIAPILREDRGSAHLHAVT
VAASVQLPAGPLPDTPDGAANSLGVPSVPIASATYMLGNGGFNATIRTLM
ERGRLSEAFTYRDLSERNINDFIKFANDPARRKEWEALCSAEQGAYAAHG
EADAGPGKKRLDDFLDKIQEMARPNQAHYMRWRLGEQERLAMDDYMAAAR
MADRGGRGKDAKACLQAVEQIAAREASWRPAALFTMHGNSKQTDTGLNFG
LQATARTAAVSDRELI FIGLPLPISDAWTQAARDQADPTA
> gi|16519844|ref|NP|443964.1| Y4lO [Rhizobium sp. NGR234]
MQLSRRAEIGNPSSRNLSPRINEKVELLGQALEHARRAGMSSSLMEYGRQ
VARHLSANVQPDEKILSLDIRNLPLLAASYNRRYPDLDLRHMDSPARFFD
ALNDRSSDGAWRAVVRLADGEQHHVAADVRTRAGAAPTIIVMEGANFYTF
VASYFKLRGDSFRQLGTQA KWAFIEVGAQKSAADCVMFGVQFALAAYREL
PTFDAWHDNLHHHGTIAHEGDYSSDYMPRRHAGICANKPFSWGEVPPSDL
LQALSLQQCN

> hopAG::ISPssy Pto DC3000|Genbank: AAO54435 (N -term) AAO54438 (C -term)
MNPITHSFSHLGFSNAQSTSALAPGGNKVPNFVSRGRGKGVPLEHFNTAD
EYRLARQQGGVLKSIDGREFMLLLQKYTAAETSDEEFA DLRAAIPRYSID
LAEPGQTKVLYRGISLPEKTAARLLNISWGYESREIAHGLIHGLRVVKEG
LKXXXXXXVTLSESRLPLSVASKSEHEVILDITNRYEITHARREGAYIVVD
MNVLGRSKRGGELEVIETDKWSQLSGAKGSNPGGLFQAPDGVKWYVKTNP
STNRLRNEVLASKLYRAAGIDVPDIKMASRQGKPALISKLIGGNHKDIKT
IEGSGQLRSGFAVDAWLANWDVIGQTGDNIIFNDRNKPV RIDLGGSLLFR
AQGGHKGNQFGNTPMELVTMLSRKENTSSHAFRKIERNDIRMGIAAIERI
PDDRIKALCAEHGPGNYSERIELGKRLISRKHWLVNMKQALPHIHRQKNE
GGDVVIVKNPTSPSDVRALSDSSATAVFVPHRPVRGSLNNIPFKSFAPPD
TVDGWRRHRTHAVDFKEPEFKFSNHLAPASGAIIFEPDGRLWITEPTNHP
FGATHAFPKGKLEPGLNLRTNALKEVYEETGLKVELHGFI GDYDRTTSRT
RYYLAKRVDGTPSDMGFESQSVKLARITEAGKLLTRGASGISEIDHTILV
KAAEVFRLNPF
> gi|27735216|sp|Q04640|MXIC|SHIFL MxiC protein
MLDVKNTGVFSSAFIDRLNAMTNSDDGDETADAELDSGLANSKYIDSSDE
MASALSSFINRRDLEKLKGTNSDSQERILDGEEDEINHKIFDLKRTLKDN
LPLDRDFIDRLKRYFKDPSDQVLALRELLNEK DLTAEQVELLTKIINEII
SGSEKSVNAGINSAIQAKLFGNKMKLEPQLLRACYRGFIMGNISTTDQYI
EWLGNFGFNHRHTIVNFVEQSLIVDMDSEKPSCNAYEFGFVLSKLIAIKM
IRTSDVIFMKKLESSSLLKDGSLSAEQLLLTLLYIFQYPSESEQILTSVI
EVSRASHEDSVVYQTYLSSVNESPHDIFKSESEREIAINILRELVTSAYK
KELSR
> Q3BWS7|Q3BWS7_XANC5 Xanth omonas outer protein O - Xanthomonas campestris
pv. vesicatoria (strain 85 -10).
MINTSVKAINSHHTHSSSSEEPKRQSRQADAGADPTRIHPALEGLPSIRQ
GMQATRRDATKTSSLEHSRRTEAFKAQRQAISMQPGPSNNPHLTALDKLQ
ERDFEPALGGVKIPLTLNSLPGGGKRVQVAGTSSRDVHVVPAGIRSELQE
AITDKQLLIQTLRQDLQEARNQG NPALIAQNERLLEQANADLRSLLGQVA
VYGEESRRINR
> A0UH23_9BURK
MTASASRPLLGCIADDFTGATDLANMLVKSGMRTVQTIGVPAESASIDAD
AIVVALKSRTIPAADAVAQSLAAYEWLRAQGCRQFFFKYCSTFDSTDAGN
IGPVADALLDAAGGGFTIACPAFPENGRTIYRGHLFVGDVLLNESGMENH
PLTPMKDANLVRVLQRQTSSKVGLIRYDTIARGAADVRACIAQLRADG VR
IAIADALSDRDLYVLGEACAALPLVTGGSGIALGLPENFRRAAELAARDN
AASLPRIDGTATVLAGSASKATNAQVAAWRATRPSFRIDPLAAARGEPVV
DQALAFARSHLPEPVLIYATATPDEVKAVQQALGVEAAGELVERTLAAIA
HGLRALGVRKFVVAGGETSGAVVQALGVKSLQIGAQIDPGVPATATIDTE
PLGLALKSGNFGAVDFFDKALRALDGAA
> gi|16767411|ref|NP|463026.1| putative cytoplasmic protein [Salmonella
typhimurium LT2]
MIPPLNRYVPALSKNELVKTVTNRDIQFTSFNGKDYPLCFLDEKTPLLFQ
WFERNPARFGKNDIPIINTEKNPYLNNIIKAATIEKERLIGIFVDGDFFP
GQKDAFSKLEYDYENIKVIYRNDIDFSMYDKKLSEIYMENISKQESMPEE
KRDCHLLQLLKKELSDIQEGNDSLIKSYLLDKGH GWFDFYRNMAMLKAGQ
LFLEADKVGCYDLSTNSGCIYLDADMIITEKLGGIYIPDGIAVHVERIDG
RASMENGIIAVDRNNHPALLAGLEIMHTKFDADPYSDGVCNGIRKHFNYS
LNEDYNSFCDFIEFKHDNIIMNTSQFTQSSWARHVQ
